# Global, regional, and national disability-adjusted life-years (DALYs) for 315 diseases and injuries and healthy life expectancy (HALE), 1990–2015: a systematic analysis for the Global Burden of Disease Study 2015

**DOI:** 10.1016/S0140-6736(16)31460-X

**Published:** 2016-10-08

**Authors:** GBD 2015 DALYs and HALE Collaborators, Nicholas J Kassebaum, Megha Arora, Ryan M Barber, Zulfiqar A Bhutta, Jonathan Brown, Austin Carter, Daniel C Casey, Fiona J Charlson, Matthew M Coates, Megan Coggeshall, Leslie Cornaby, Lalit Dandona, Daniel J Dicker, Holly E Erskine, Alize J Ferrari, Christina Fitzmaurice, Kyle Foreman, Mohammad H Forouzanfar, Nancy Fullman, Peter W Gething, Ellen M Goldberg, Nicholas Graetz, Juanita A Haagsma, Simon I Hay, Catherine O Johnson, Laura Kemmer, Ibrahim A Khalil, Yohannes Kinfu, Michael J Kutz, Hmwe H Kyu, Janni Leung, Xiaofeng Liang, Stephen S Lim, Rafael Lozano, George A Mensah, Joe Mikesell, Ali H Mokdad, Meghan D Mooney, Mohsen Naghavi, Grant Nguyen, Elaine Nsoesie, David M Pigott, Christine Pinho, Zane Rankin, Nikolas Reinig, Joshua A Salomon, Logan Sandar, Alison Smith, Reed J D Sorensen, Jeffrey Stanaway, Caitlyn Steiner, Stephanie Teeple, Chris Troeger, Thomas Truelsen, Amelia VanderZanden, Joseph A Wagner, Valentine Wanga, Harvey A Whiteford, Maigeng Zhou, Leo Zoeckler, Amanuel Alemu Abajobir, Kalkidan Hassen Abate, Cristiana Abbafati, Kaja M Abbas, Foad Abd-Allah, Biju Abraham, Ibrahim Abubakar, Laith J Abu-Raddad, Niveen M E Abu-Rmeileh, Tom Achoki, Ilana N Ackerman, Akindele Olupelumi Adebiyi, Isaac Akinkunmi Adedeji, José C Adsuar, Kossivi Agbelenko Afanvi, Ashkan Afshin, Emilie Elisabet Agardh, Arnav Agarwal, Sanjay Kumar Agarwal, Muktar Beshir Ahmed, Aliasghar Ahmad Kiadaliri, Hamid Ahmadieh, Nadia Akseer, Ziyad Al-Aly, Khurshid Alam, Noore K M Alam, Saleh Fahed Aldhahri, Miguel Angel Alegretti, Alicia V Aleman, Zewdie Aderaw Alemu, Lily T Alexander, Raghib Ali, Ala'a Alkerwi, François Alla, Peter Allebeck, Christine Allen, Ubai Alsharif, Khalid A Altirkawi, Elena Alvarez Martin, Nelson Alvis-Guzman, Azmeraw T Amare, Alemayehu Amberbir, Adeladza Kofi Amegah, Heresh Amini, Walid Ammar, Stephen Marc Amrock, Gregory M Anderson, Benjamin O Anderson, Carl Abelardo T Antonio, Palwasha Anwari, Johan Ärnlöv, Valentina S Arsic Arsenijevic, Al Artaman, Hamid Asayesh, Rana Jawad Asghar, Euripide Frinel G Arthur Avokpaho, Ashish Awasthi, Beatriz Paulina Ayala Quintanilla, Peter Azzopardi, Umar Bacha, Alaa Badawi, Kalpana Balakrishnan, Amitava Banerjee, Aleksandra Barac, Suzanne L Barker-Collo, Till Bärnighausen, Lars Barregard, Lope H Barrero, Sanjay Basu, Tigist Assefa Bayou, Justin Beardsley, Neeraj Bedi, Ettore Beghi, Brent Bell, Michelle L Bell, Corina Benjet, Derrick A Bennett, Isabela M Bensenor, Adugnaw Berhane, Eduardo Bernabé, Balem Demtsu Betsu, Addisu Shunu Beyene, Neeraj Bhala, Anil Bhansali, Samir Bhatt, Sibhatu Biadgilign, Kelly Bienhoff, Boris Bikbov, Aref A Bin Abdulhak, Stan Biryukov, Donal Bisanzio, Espen Bjertness, Jed D Blore, Rohan Borschmann, Soufiane Boufous, Rupert R A Bourne, Michael Brainin, Alexandra Brazinova, Nicholas J K Breitborde, Traolach S Brugha, Rachelle Buchbinder, Geoffrey Colin Buckle, Zahid A Butt, Bianca Calabria, Ismael Ricardo Campos-Nonato, Julio Cesar Campuzano, Hélène Carabin, Jonathan R Carapetis, Rosario Cárdenas, Juan Jesus Carrero, Carlos A Castañeda-Orjuela, Jacqueline Castillo Rivas, Ferrán Catalá-López, Fiorella Cavalleri, Jung-Chen Chang, Peggy Pei-Chia Chiang, Mirriam Chibalabala, Chioma Ezinne Chibueze, Vesper Hichilombwe Chisumpa, Jee-Young Jasmine Choi, Lincoln Choudhury, Hanne Christensen, Liliana G Ciobanu, Valentina Colistro, Mercedes Colomar, Samantha M Colquhoun, Monica Cortinovis, John A Crump, Albertino Damasceno, Rakhi Dandona, Paul I Dargan, José das Neves, Gail Davey, Adrian C Davis, Diego De Leo, Louisa Degenhardt, Liana C Del Gobbo, Sarah Derrett, Don C Des Jarlais, Gabrielle A deVeber, Samath D Dharmaratne, Preet K Dhillon, Eric L Ding, Kerrie E Doyle, Tim R Driscoll, Leilei Duan, Manisha Dubey, Bruce Bartholow Duncan, Hedyeh Ebrahimi, Richard G Ellenbogen, Iqbal Elyazar, Aman Yesuf Endries, Sergey Petrovich Ermakov, Babak Eshrati, Alireza Esteghamati, Kara Estep, Saman Fahimi, Talha A Farid, Carla Sofia e Sa Farinha, André Faro, Maryam S Farvid, Farshad Farzadfar, Valery L Feigin, Seyed-Mohammad Fereshtehnejad, Jefferson G Fernandes, Joao C Fernandes, Florian Fischer, Joseph R A Fitchett, Nataliya Foigt, F Gerry R Fowkes, Richard C Franklin, Joseph Friedman, Joseph Frostad, Thomas Fürst, Neal D Futran, Belinda Gabbe, Fortuné Gbètoho Gankpé, Alberto L Garcia-Basteiro, Tsegaye Tewelde Gebrehiwot, Amanuel Tesfay Gebremedhin, Johanna M Geleijnse, Katherine B Gibney, Richard F Gillum, Ibrahim Abdelmageem Mohamed Ginawi, Ababi Zergaw Giref, Maurice Giroud, Melkamu Dedefo Gishu, Giorgia Giussani, William W Godwin, Hector Gomez-Dantes, Philimon Gona, Amador Goodridge, Sameer Vali Gopalani, Carolyn C Gotay, Atsushi Goto, Hebe N Gouda, Harish Gugnani, Yuming Guo, Rahul Gupta, Rajeev Gupta, Vipin Gupta, Reyna A Gutiérrez, Nima Hafezi-Nejad, Demewoz Haile, Alemayehu Desalegne Hailu, Gessessew Bugssa Hailu, Yara A Halasa, Randah Ribhi Hamadeh, Samer Hamidi, Mouhanad Hammami, Alexis J Handal, Graeme J Hankey, Hilda L Harb, Sivadasanpillai Harikrishnan, Josep Maria Haro, Mohammad Sadegh Hassanvand, Tahir Ahmed Hassen, Rasmus Havmoeller, Roderick J Hay, Mohammad T Hedayati, Ileana Beatriz Heredia-Pi, Pouria Heydarpour, Hans W Hoek, Daniel J Hoffman, Masako Horino, Nobuyuki Horita, H Dean Hosgood, Damian G Hoy, Mohamed Hsairi, Hsiang Huang, John J Huang, Kim Moesgaard Iburg, Bulat T Idrisov, Kaire Innos, Manami Inoue, Kathryn H Jacobsen, Alejandra Jauregui, Achala Upendra Jayatilleke, Panniyammakal Jeemon, Vivekanand Jha, Guohong Jiang, Ying Jiang, Tariku Jibat, Aida Jimenez-Corona, Ye Jin, Jost B Jonas, Zubair Kabir, Dan K Kajungu, Yogeshwar Kalkonde, Ritul Kamal, Haidong Kan, Amit Kandel, André Karch, Corine Kakizi Karema, Chante Karimkhani, Amir Kasaeian, Marzieh Katibeh, Anil Kaul, Norito Kawakami, Dhruv S Kazi, Peter Njenga Keiyoro, Andrew Haddon Kemp, Andre Pascal Kengne, Andre Keren, Chandrasekharan Nair Kesavachandran, Yousef Saleh Khader, Abdur Rahman Khan, Ejaz Ahmad Khan, Young-Ho Khang, Tawfik Ahmed Muthafer Khoja, Jagdish Khubchandani, Christian Kieling, Cho-il Kim, Daniel Kim, Yun Jin Kim, Niranjan Kissoon, Miia Kivipelto, Luke D Knibbs, Ann Kristin Knudsen, Yoshihiro Kokubo, Dhaval Kolte, Jacek A Kopec, Parvaiz A Koul, Ai Koyanagi, Barthelemy Kuate Defo, Ricardo S Kuchenbecker, Burcu Kucuk Bicer, Ernst J Kuipers, G Anil Kumar, Gene F Kwan, Ratilal Lalloo, Tea Lallukka, Anders Larsson, Asma Abdul Latif, Pablo M Lavados, Alicia Elena Beatriz Lawrynowicz, Janet L Leasher, James Leigh, Ricky Leung, Yichong Li, Yongmei Li, Steven E Lipshultz, Patrick Y Liu, Yang Liu, Belinda K Lloyd, Giancarlo Logroscino, Katharine J Looker, Paulo A Lotufo, Robyn M Lucas, Raimundas Lunevicius, Ronan A Lyons, Hassan Magdy Abd El Razek, Mahdi Mahdavi, Marek Majdan, Azeem Majeed, Reza Malekzadeh, Deborah Carvalho Malta, Wagner Marcenes, Jose Martinez-Raga, Felix Masiye, Amanda J Mason-Jones, Richard Matzopoulos, Bongani M Mayosi, John J McGrath, Martin McKee, Peter A Meaney, Alem Mehari, Yohannes Adama Melaku, Peter Memiah, Ziad A Memish, Walter Mendoza, Atte Meretoja, Tuomo J Meretoja, Yonatan Moges Mesfin, Francis Apolinary Mhimbira, Anoushka Millear, Ted R Miller, Edward J Mills, Mojde Mirarefin, Erkin M Mirrakhimov, Philip B Mitchell, Charles N Mock, Karzan Abdulmuhsin Mohammad, Alireza Mohammadi, Shafiu Mohammed, Lorenzo Monasta, Julio Cesar Montañez Hernandez, Marcella Montico, Maziar Moradi-Lakeh, Rintaro Mori, Ulrich O Mueller, John Everett Mumford, Michele E Murdoch, Gudlavalleti Venkata Satyanarayana Murthy, Jean B Nachega, Aliya Naheed, Luigi Naldi, Vinay Nangia, John N Newton, Marie Ng, Frida Namnyak Ngalesoni, Quyen Le Nguyen, Muhammad Imran Nisar, Patrick Martial Nkamedjie Pete, Joan M Nolla, Ole F Norheim, Rosana E Norman, Bo Norrving, Carla Makhlouf Obermeyer, Felix Akpojene Ogbo, In-Hwan Oh, Olanrewaju Oladimeji, Pedro R Olivares, Bolajoko Olubukunola Olusanya, Jacob Olusegun Olusanya, Eyal Oren, Alberto Ortiz, Erika Ota, Abayomi Samuel Oyekale, Mahesh PA, Eun-Kee Park, Mahboubeh Parsaeian, Scott B Patten, George C Patton, João Mário Pedro, David M Pereira, Norberto Perico, Konrad Pesudovs, Max Petzold, Michael Robert Phillips, Frédéric B Piel, Julian David Pillay, Farhad Pishgar, Dietrich Plass, Suzanne Polinder, Svetlana Popova, Richie G Poulton, Farshad Pourmalek, Noela M Prasad, Mostafa Qorbani, Rynaz H S Rabiee, Amir Radfar, Anwar Rafay, Kazem Rahimi, Vafa Rahimi-Movaghar, Mahfuzar Rahman, Mohammad Hifz Ur Rahman, Sajjad Ur Rahman, Dheeraj Rai, Rajesh Kumar Rai, Sasa Rajsic, Murugesan Raju, Usha Ram, Kavitha Ranganathan, Amany H Refaat, Marissa B Reitsma, Giuseppe Remuzzi, Serge Resnikoff, Alex Reynolds, Antonio L Ribeiro, Stefano Ricci, Hirbo Shore Roba, David Rojas-Rueda, Luca Ronfani, Gholamreza Roshandel, Gregory A Roth, Ambuj Roy, Ben Benasco Sackey, Rajesh Sagar, Juan R Sanabria, Maria Dolores Sanchez-Niño, Itamar S Santos, João Vasco Santos, Rodrigo Sarmiento-Suarez, Benn Sartorius, Maheswar Satpathy, Miloje Savic, Monika Sawhney, Maria Inês Schmidt, Ione J C Schneider, Aletta E Schutte, David C Schwebel, Soraya Seedat, Sadaf G Sepanlou, Edson E Servan-Mori, Saeid Shahraz, Masood Ali Shaikh, Rajesh Sharma, Jun She, Sara Sheikhbahaei, Jiabin Shen, Kevin N Sheth, Kenji Shibuya, Mika Shigematsu, Min-Jeong Shin, Rahman Shiri, Inga Dora Sigfusdottir, Diego Augusto Santos Silva, Jonathan I Silverberg, Edgar P Simard, Abhishek Singh, Jasvinder A Singh, Prashant Kumar Singh, Vegard Skirbekk, Jens Christoffer Skogen, Michael Soljak, Kjetil Søreide, Reed J D Sorensen, Chandrashekhar T Sreeramareddy, Vasiliki Stathopoulou, Nicholas Steel, Dan J Stein, Murray B Stein, Timothy J Steiner, Lars Jacob Stovner, Saverio Stranges, Konstantinos Stroumpoulis, Bruno F Sunguya, Patrick J Sur, Soumya Swaminathan, Bryan L Sykes, Cassandra E I Szoeke, Rafael Tabarés-Seisdedos, Nikhil Tandon, David Tanne, Mohammad Tavakkoli, Bineyam Taye, Hugh R Taylor, Braden J Te Ao, Teketo Kassaw Tegegne, Dejen Yemane Tekle, Abdullah Sulieman Terkawi, Gizachew Assefa Tessema, J S Thakur, Alan J Thomson, Andrew L Thorne-Lyman, Amanda G Thrift, George D Thurston, Ruoyan Tobe-Gai, Marcello Tonelli, Roman Topor-Madry, Fotis Topouzis, Bach Xuan Tran, Thomas Truelsen, Zacharie Tsala Dimbuene, Miltiadis Tsilimbaris, Abera Kenay Tura, Emin Murat Tuzcu, Stefanos Tyrovolas, Kingsley N Ukwaja, Eduardo A Undurraga, Chigozie Jesse Uneke, Olalekan A Uthman, Coen H van Gool, Jim van Os, Tommi Vasankari, Ana Maria Nogales Vasconcelos, Narayanaswamy Venketasubramanian, Francesco S Violante, Vasiliy Victorovich Vlassov, Stein Emil Vollset, Gregory R Wagner, Mitchell T Wallin, Linhong Wang, Scott Weichenthal, Elisabete Weiderpass, Robert G Weintraub, Andrea Werdecker, Ronny Westerman, Tissa Wijeratne, James D Wilkinson, Hywel C Williams, Charles Shey Wiysonge, Solomon Meseret Woldeyohannes, Charles D A Wolfe, Sungho Won, Gelin Xu, Ajit Kumar Yadav, Bereket Yakob, Lijing L Yan, Yuichiro Yano, Mehdi Yaseri, Pengpeng Ye, Paul Yip, Naohiro Yonemoto, Seok-Jun Yoon, Mustafa Z Younis, Chuanhua Yu, Zoubida Zaidi, Maysaa El Sayed Zaki, Hajo Zeeb, Sanjay Zodpey, David Zonies, Liesl Joanna Zuhlke, Theo Vos, Alan D Lopez, Christopher J L Murray

## Abstract

**Background:**

Healthy life expectancy (HALE) and disability-adjusted life-years (DALYs) provide summary measures of health across geographies and time that can inform assessments of epidemiological patterns and health system performance, help to prioritise investments in research and development, and monitor progress toward the Sustainable Development Goals (SDGs). We aimed to provide updated HALE and DALYs for geographies worldwide and evaluate how disease burden changes with development.

**Methods:**

We used results from the Global Burden of Diseases, Injuries, and Risk Factors Study 2015 (GBD 2015) for all-cause mortality, cause-specific mortality, and non-fatal disease burden to derive HALE and DALYs by sex for 195 countries and territories from 1990 to 2015. We calculated DALYs by summing years of life lost (YLLs) and years of life lived with disability (YLDs) for each geography, age group, sex, and year. We estimated HALE using the Sullivan method, which draws from age-specific death rates and YLDs per capita. We then assessed how observed levels of DALYs and HALE differed from expected trends calculated with the Socio-demographic Index (SDI), a composite indicator constructed from measures of income per capita, average years of schooling, and total fertility rate.

**Findings:**

Total global DALYs remained largely unchanged from 1990 to 2015, with decreases in communicable, neonatal, maternal, and nutritional (Group 1) disease DALYs offset by increased DALYs due to non-communicable diseases (NCDs). Much of this epidemiological transition was caused by changes in population growth and ageing, but it was accelerated by widespread improvements in SDI that also correlated strongly with the increasing importance of NCDs. Both total DALYs and age-standardised DALY rates due to most Group 1 causes significantly decreased by 2015, and although total burden climbed for the majority of NCDs, age-standardised DALY rates due to NCDs declined. Nonetheless, age-standardised DALY rates due to several high-burden NCDs (including osteoarthritis, drug use disorders, depression, diabetes, congenital birth defects, and skin, oral, and sense organ diseases) either increased or remained unchanged, leading to increases in their relative ranking in many geographies. From 2005 to 2015, HALE at birth increased by an average of 2·9 years (95% uncertainty interval 2·9–3·0) for men and 3·5 years (3·4–3·7) for women, while HALE at age 65 years improved by 0·85 years (0·78–0·92) and 1·2 years (1·1–1·3), respectively. Rising SDI was associated with consistently higher HALE and a somewhat smaller proportion of life spent with functional health loss; however, rising SDI was related to increases in total disability. Many countries and territories in central America and eastern sub-Saharan Africa had increasingly lower rates of disease burden than expected given their SDI. At the same time, a subset of geographies recorded a growing gap between observed and expected levels of DALYs, a trend driven mainly by rising burden due to war, interpersonal violence, and various NCDs.

**Interpretation:**

Health is improving globally, but this means more populations are spending more time with functional health loss, an absolute expansion of morbidity. The proportion of life spent in ill health decreases somewhat with increasing SDI, a relative compression of morbidity, which supports continued efforts to elevate personal income, improve education, and limit fertility. Our analysis of DALYs and HALE and their relationship to SDI represents a robust framework on which to benchmark geography-specific health performance and SDG progress. Country-specific drivers of disease burden, particularly for causes with higher-than-expected DALYs, should inform financial and research investments, prevention efforts, health policies, and health system improvement initiatives for all countries along the development continuum.

**Funding:**

Bill & Melinda Gates Foundation.

## Introduction

Summary measures of population health are crucial inputs to guide health system investments and set priorities at global, regional, national, and subnational levels. The Millennium Development Goals (MDGs), which sought to reduce extreme poverty and improve health, expired in 2015, and were replaced by the 2030 Agenda for Sustainable Development, or Sustainable Development Goals (SDGs).^1^ The shift from the MDGs to the SDGs reflects a broadening of the global development agenda,^2^, ^3^ expanding to include targets for non-communicable diseases (NCDs) and indicators that consider the interplay of environmental, societal, and economic factors on health.^4^ Within this context, summary population health measures are advantageous because they can easily be used to show progress toward SDG 3—to “ensure healthy lives and promote well-being for all at all ages”—and provide a metric by which comparative progress on other SDGs can be monitored.^5^ Summary measures also provide insights into whether, as societies live longer, they spend more or less of their time with functional health loss, known as the expansion or compression of morbidity, respectively, which has profound implications for societies and the financing of health systems.

Two types of population health summary measures exist: health expectancies and health gaps.^6^ Healthy life expectancy (HALE), which originates from Sullivan,^7^ provides a single summary measure of population health by weighting years lived with a measure of functional health loss experienced before death. Many health expectancy measures have been proposed, but HALE is the only one that captures a full range of functional health loss.^8^, ^9^, ^10^ Health gap measures capture differences between a population and some normative standard such as a maximum lifespan in full health. Disability-adjusted life-years (DALY) are a widely used gap measure,^6^, ^9^, ^10^, ^11^ representing the sum of years of life lost (YLLs) due to premature mortality and years lived with disability (YLDs). YLLs quantify the gap between observed mortality and a normative life expectancy,^12^ and YLDs capture the prevalence of conditions that lead to non-fatal health loss while accounting for the severity of those conditions. Health gap measures can be easily disaggregated to examine contributions of relative morbidity and mortality, individual diseases, injuries, and attributable risk factors.

The Global Burden of Diseases, Injuries, and Risk Factors (GBD) study is the most comprehensive source of comparable summary population health measures because of its inclusion of country-level results, uncertainty quantification, and its effort to maximise comparability across geography, time, and across different health conditions. Alternative summary health assessments are not as standardised or comprehensive, with studies reporting only incomplete time-series, no uncertainty measures, or only a subset of countries and causes.^13^, ^14^, ^15^ WHO published DALY estimates for 2 years (2000 and 2012), with 132 causes and 174 countries and without uncertainty intervals. These estimates were derived primarily from GBD 2010 results, but were modified in 60 countries and for 12 cause groups separately estimated by WHO and UN agencies.^13^, ^16^, ^17^ WHO applied the same approach for GBD 2013 results and used their own life tables to produce HALE estimates for 2015.^14^ The European Commission (EC) and the Organisation for Economic Co-operation and Development (OECD) also reported healthy life expectancy estimates for European countries from 2004 through 2014, but these were based on self-reported health status.^18^, ^19^

**Research in context**

**Evidence before this study**

Disability-adjusted life-years (DALYs), a summary measure of population health based on estimates of premature mortality and non-fatal health loss, originated from the initial Global Burden of Disease (GBD) study in 1993. DALYs, in combination with other summary measures such as healthy life expectancy (HALE), offer relatively simple yet powerful metrics against which progress and challenges in improving disease burden and extending healthy lifespans can be effectively monitored over time. Published in 2012, GBD 2010 provided updated estimates of DALYs due to 291 causes and HALE in 187 countries from 1990 to 2010. GBD 2013 extended this time series to 2013, with 188 countries, and 306 causes. Novel analyses for quantifying epidemiological transitions were introduced as part of GBD 2013, enabling a comparison of shifts in years of life lost (YLLs) and years lived with disability (YLDs) with increasing levels of development. WHO has produced estimates of DALYs and HALE largely based on GBD 2010 and GBD 2013; however, modifications were implemented for a subset of causes, disability weights, and countries, and a normative life table of 91·9 years at birth was used for calculating YLLs.

**Added value of this study**

For GBD 2015, we generated estimates of HALE and DALYs for 315 causes by geography, sex, and age group from 1990 to 2015 for 195 countries and territories. We constructed a summary metric referred to as the Socio-demographic Index (SDI) based on measures of income per capita, average years of schooling, and total fertility rate. We estimated SDI for each geography-year, and characterised the average relationship for each age, sex, and cause for DALYs and HALE with SDI. Using these relationships, we calculated expected levels of DALYs, life expectancy, and HALE for each geography over time. We compared observed patterns of both DALYs and HALE with those expected on the basis of SDI, allowing us to explore where health gains exceeded—or lagged behind—corresponding changes in development.

**Implications**

Since 1990, overall health has improved in most countries, with particularly large gains occurring in the past 10 years. Although improved health means longer lifespans, it also translates to more years of functional health lost. The fraction of overall life expectancy spent in poor health is generally constant or has slightly declined in some countries, a result driven by declines in DALYs due to communicable, maternal, nutritional, and neonatal causes and increases in others, mainly non-communicable diseases. Country-specific drivers of disease burden, particularly when observed DALYs are higher than expected on the basis of SDI, should inform country-specific inquiry and action.

Here we present GBD 2015 findings for DALYs and HALE, building upon updated estimates of mortality, causes of death, and non-fatal health loss.^12^, ^20^ Overall analytic approaches are similar to previous GBD studies,^9^, ^10^ but include new mortality and morbidity data, refined methods, and expanded geographies.^12^, ^20^ This report supersedes all previous GBD studies on DALYs and HALE through the estimation of a complete time-series for 1990 to 2015. To facilitate a more in-depth examination of the drivers of DALY and HALE trends, we assess how HALE, along with overall and cause-specific DALYs, change as geographies move through the development continuum. We use this analysis to benchmark overall progress and decompose observed disease burden compared with levels expected for specific causes on the basis of development alone, to highlight potential areas for policy investment or further research.

## Methods

### Overview

Detailed methods for estimating DALYs and HALE, including analytic approaches for mortality and non-fatal health loss estimation, are provided in related publications.^12^, ^20^ Additional detail on GBD metrics and definitions are found elsewhere.^21^ Interactive tools are also available to explore GBD 2015 results and data sources. This analysis follows the Guidelines for Accurate and Transparent Health Estimates Reporting (GATHER), which includes recommendations on documentation of data sources, estimation methods, and statistical analysis.^22^, ^23^

In brief, the GBD geographic hierarchy involves 519 total geographies within 195 countries and territories, 21 regions, and seven super-regions. This study reports results for all countries and territories. The GBD cause hierarchy has four levels of classification and causes reported within each level that are mutually exclusive and collectively exhaustive. The full GBD cause list with corresponding International Classification of Diseases (ICD)-9 and ICD-10 codes are available in our publications on cause-specific mortality^12^ and non-fatal health outcomes.^20^

### Estimation of mortality and non-fatal health loss

We estimated all-cause and cause-specific mortality with a multistep computation process, which included systematically addressing known data challenges, such as different coding schemes, different age-group reporting, variation in certification, misclassification of HIV/AIDS deaths in some countries, misclassification of maternal HIV/AIDS deaths, and incorporation of population-based cancer registry data, before computation of cause-specific mortality with analytic tools such as Cause of Death Ensemble Modelling (CODEm). Each death could have only one underlying cause. Additional detail, including model specifications and data availability for each cause-specific model, can be found in the supplementary material of the GBD 2015 mortality and causes of death publication.^12^ We calculated normative life tables based on the lowest death rates for each age group among geographies with total populations greater than 5 million. We computed cause-specific YLLs by multiplying cause-specific deaths by the life expectancy at the age of death (ie, 86·59 years at age 0 years; 23·79 years at age 65 years) from this normative life table, and then used the GBD world population age standard to calculate age-standardised mortality rates and YLL rates.^12^

Our most commonly used analytic approach to estimate non-fatal health loss was DisMod-MR 2.1, a Bayesian meta-regression tool that synthesises diverse data sources to produce internally consistent estimates of incidence, prevalence, remission, and excess mortality. The use of other methods to estimate non-fatal health loss was determined by cause-specific data availability and epidemiological characteristics.^24^ Additional detail, including model specifications and data availability for each cause-specific model, can be found in the supplementary material of the GBD 2015 non-fatal publication.^20^ Each non-fatal sequela was estimated separately. We then applied a microsimulation framework to assess the occurrence of comorbidity in each age group, sex, geography, and year separately. Disability from comorbid conditions was apportioned to each of the contributing causes. GBD disability weights were based on population surveys with more than 60 000 respondents, and previous studies show that disability weights do not significantly vary across geographies, income, or educational attainment.^25^, ^26^ In this study, disability weights are invariant over geography and time, although the distribution of sequelae, and therefore the severity and cumulative disability per case of a condition, can differ by age, sex, geography, and year.

### Estimation of DALYs, HALE, and corresponding uncertainty

DALYs are the sum of YLLs and YLDs as estimated in GBD 2015 for each cause, geography, age group, sex, and year.^12^, ^20^ Using methods developed by Sullivan,^7^ we calculated HALE by age group within abridged multiple-decrement life tables and estimates of YLDs per capita for each geography–age–sex–year from 1990 to 2015.^8^, ^10^, ^27^

For all results, we report 95% uncertainty intervals (UIs), which were derived from 1000 draws from the posterior distribution of each step in the estimation process. UIs are distinct from confidence intervals, because confidence intervals only capture the uncertainty associated with sampling error, whereas uncertainty intervals provide a method for propagation of uncertainty from multiple sources including sampling, model estimation, and model specification. 95% UIs represent the ordinal 25th and 975th draw of the quantity of interest. For mortality and YLLs, UIs reflect uncertainty that arises from sample sizes of studies used as data sources, adjustments to sources of all-cause mortality, parameter uncertainty in model estimation, and specification uncertainty for all-cause and cause-specific models. For prevalence, incidence, and YLDs, UIs reflect the uncertainty that arises from sample sizes of studies used as data sources, data adjustments from non-reference definitions, parameter uncertainty in model estimation, and uncertainty in the disability weights. In the absence of any direction information about the correlation between uncertainty in YLLs and YLDs, we assumed uncertainty in age-specific YLDs is independent of age-specific YLLs in DALYs and death rates in HALE.

### Epidemiological transition and relationship between DALYs, HALE, and SDI

We examined the relationship between DALYs, HALE, and the Socio-demographic Index (SDI).^28^ SDI was constructed based on the geometric mean of three indicators: income per capita, average years of schooling among people aged 15 years or older, and the total fertility rate. SDI values were scaled to a range of 0 to 1, with 0 equalling the lowest income, lowest schooling, and highest fertility rate observed from 1980 to 2015, and 1 equalling the highest income, highest schooling, and lowest fertility rate assessed during that time. The average relationships between each summary health measure and SDI were estimated using spline regressions. These regressions were used to estimate expected values at each level of SDI. Additional detail on SDI computation and geography-specific SDI values are available in the appendix (pp 4–5 and pp 74–80).

### Role of the funding source

The funder of the study had no role in study design, data collection, data analysis, data interpretation, or writing of the report. The corresponding author had full access to all the data in the study and had final responsibility for the decision to submit for publication.

## Results

### Global trends for DALYs and HALE

**Figure 1:**
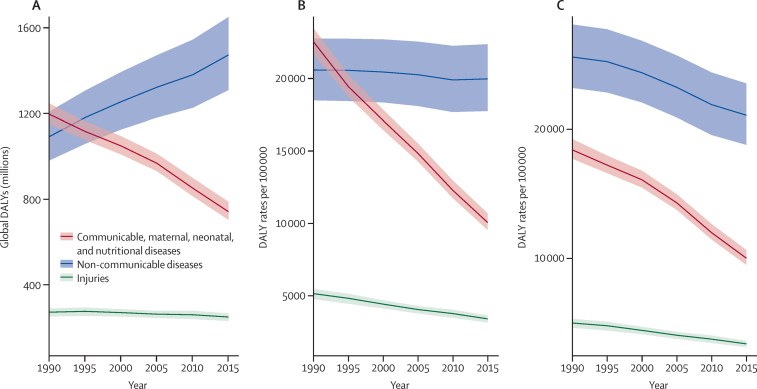
Trends from 1990 to 2015, by GBD Level 1 cause, in global DALYs (A), crude DALY rates (B), and age-standardised DALY rates (C) The difference in trends between (A) and (B) is caused by population growth and the difference between (B) and (C) is caused by changes in the percentage distribution of the population by age. Shaded areas show 95% uncertainty intervals. DALYs=disability-adjusted life-years.

Worldwide total DALYs due to Group 1 causes fell from 1·2 billion (95% CI UI 1·2–1·2) in 1990, to 741·6 million (703·9–787·7) in 2015, whereas total DALYs due to NCDs increased from 1·1 billion (1·0–1·2), to 1·5 billion (1·3–1·7; figure 1; table 1). Total injury DALYs were relatively unchanged between 1990 and 2015. All-age DALY rates for NCDs changed little between 1990 and 2015, whereas they declined substantially for Group 1 causes. Taking into account population ageing, DALY rates for Group 1 causes and NCDs both decreased (figure 1). For injuries, reductions in all-age DALY rates and age-standardised DALY rates were similar between 1990 and 2015.

HALE at birth increased to 60·9 years for men and 64·9 years for women in 2015, rising 2·9 years and 3·5 years since 2005, respectively (table 2). The gap between life expectancy and HALE, which represents years of functional health lost, widened between 2005 and 2015 from 7·7 years to 8·1 years for men, and from 9·4 years to 10·0 years for women. Life expectancy at age 65 years was 15·5 years for men and 18·5 years for women, while HALE was 11·9 years and 14·2 years for each sex, respectively (table 2).

### Global causes of DALYs

In 2015, Group 1 causes accounted for 30·1% (95% UI 28·6–31·7%) of global DALYs, with NCDs leading to 59·7% (57·8–61·5) and injuries to 10·1% (9·5–10·7; table 1). Since 2005, DALYs due to many of the world's leading communicable causes substantially declined, yet burden increased for a subset of infectious diseases. Age-standardised DALY rates from HIV/AIDS and malaria each fell by more than 40% and lower respiratory infections and diarrhoeal diseases had decreases in total and age-standardised rates of DALYs of more than 20% (table 1). From 2005 to 2015, reductions in both total and age-standardised DALY rates due to tetanus and measles surpassed 50% and 70%, respectively. African trypanosomiasis, a disease targeted for elimination, saw both total DALYs and age-standardised rates fall by more than 70% since 2005. DALY rates substantially fell for all types of hepatitis, with age-standardised DALY rates from acute hepatitis A declining by more than 35% by 2015. However, both total DALYs and age-standardised DALY rates from dengue increased by more than 50%. Although the west African Ebola virus outbreak peaked in 2014,^29^ Ebola virus disease still caused substantial DALYs in 2015 (table 1). Maternal disorders significantly declined from 2005 to 2015, with total DALYs and age-standardised rates each falling by more than 20%. Reductions in the global burden of neonatal disorders were somewhat less pronounced; for instance, the number of DALYs due to neonatal sepsis was largely unchanged (table 1).

In 2015, cardiovascular diseases, cancers, and mental and substance use disorders were among the leading causes of NCD burden (table 1). For many NCDs, including most cardiovascular causes and most cancers, total DALYs increased but age-standardised DALY rates declined. Nearly all neurological disorders increased in total DALYs, including Alzheimer's disease and other dementias, which rose by more than 30%, whereas age-standardised rates either moderately decreased (eg, Alzheimer's disease and other dementias) or were relatively unchanged (eg, Parkinson's disease). Total DALYs from low back and neck pain also increased, rising by more than 17%. Cirrhosis caused more DALYs in 2015 than in 2005, although age-standardised DALY rates significantly fell. A similar overall trend was found for diabetes and chronic kidney disease, with all aetiologies apart from chronic kidney disease due to diabetes, showing significant declines in age-standardised DALY rates amid rising total DALYs. For other NCDs, namely those associated with skin diseases, sensory conditions, and oral disorders, total DALYs significantly increased from 2005 to 2015, and age-standardised DALY rates either somewhat increased or did not significantly change since 2005. Age-standardised DALY rates due to chronic obstructive pulmonary disease fell by more than 20% from 2005 to 2015, while those due to asthma decreased by almost 17%. Peptic ulcer disease, a leading cause of digestive disease burden, saw marked reductions in total DALYs and age-standardised DALY rates, with the latter decreasing by nearly 30% (table 1).

A number of NCDs significantly increased in terms of total burden and age-standardised DALY rates. Osteoarthritis was the most notable example, with total DALYs rising by 35% and age-standardised DALY rates by 4% between 1990 and 2015. Major depressive disorders and drug use disorders, particularly of opioids and cocaine, both increased in total DALYs and age-standardised rates; however, age-standardised DALY rates from alcohol use disorders dropped by 19%. Total DALYs and age-standardised DALY rates from chronic kidney disease due to diabetes also significantly increased by 2015. Male and female infertility accounted for a relatively small fraction of NCDs, but burden due to both causes increased significantly since 2005. Oral disorders and sense organ diseases also had increased total DALYs, whereas age-standardised DALY rates were relatively unchanged since 2005.

Unintentional injuries and transport injuries each saw age-standardised DALY rates significantly decrease (20% and 17%, respectively). Road injury burden significantly declined from 2005, with age-standardised DALY rates falling by 18% by 2015. Among unintentional injuries, drowning had the largest reduction in both total burden (26%) and age-standardised DALY rates (32%). Age-standardised DALY rates from self-harm and interpersonal violence both fell by more than 16% since 2005. DALYs due to forces of nature, war, and legal intervention increased from 2005 to 2015, although not significantly; this rise was primarily driven by escalated violence and war in the Middle East. Despite still causing major health loss in 2015, forces of nature caused far fewer DALYs than in 2005, mainly because there were no large-scale losses of life like that seen in the 2005 earthquake that killed more than 70 000 people in India and Pakistan.

### Changes in leading causes of disease burden over time

**Figure 2:**
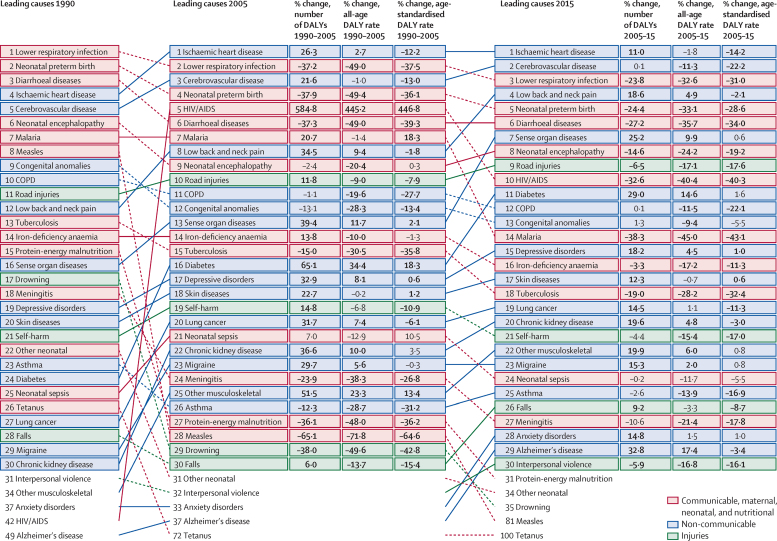
Leading 30 Level 3 causes of global DALYs for both sexes combined, 1990, 2005, 2015, with percentage change in number of DALYs, and all-age, and age-standardised rates Causes are connected by lines between time periods; solid lines are increases and dashed lines are decreases. For the time period of 1990 to 2005 and for 2005 to 2015, three measures of change are shown: percent change in the number of DALYs, percent change in the all-age DALY rate, and percent change in the age-standardised DALY rate. Statistically significant changes are shown in bold. DALYs=disability-adjusted life-years. COPD=chronic obstructive pulmonary disease.

In 1990, lower respiratory infections, preterm birth complications, and diarrhoeal diseases were the three leading causes of global DALYs; by 2015, only lower respiratory infections remained in the leading three (figure 2). Many Group 1 causes had significant declines for total burden, as well as all-age DALY rates and age-standardised DALY rates, for both time periods; these causes included tuberculosis, meningitis, diarrhoeal diseases, protein-energy malnutrition, preterm birth complications, tetanus, and measles. Such reductions across measures of DALYs contributed to downward shifts in relative ranks for most Group 1 causes over time. Malaria and HIV/AIDS both diverged from this trend, with each recording large increases in burden from 1990 to 2005, but by 2015, all measures of DALYs and relative ranks for malaria and HIV/AIDS had fallen markedly. Trends for NCDs and injuries, both in terms of ranks and changes in disease burden, were more varied. Between 1990 and 2005, total DALYs and all-age DALY rates significantly increased for many NCDs, including ischaemic heart disease, low back and neck pain, lung cancer, chronic kidney disease, and migraine. For these NCDs, their relative ranks also climbed by 2005, yet their age-standardised DALY rates either significantly decreased or remained relatively unchanged, reflecting the effects of changes in population age structure. This pattern continued through 2015 for many NCDs, and was further exemplified by Alzheimer's disease and other dementias as it rose to the 29th leading cause of global DALYs amid a significant decrease in age-standardised DALY rates. From 1990 to 2005, all three measures of DALYs significantly increased for a subset of NCDs (ie, sense organ diseases, diabetes, depressive disorders, and other musculoskeletal disorders), which contributed to their rises in relative ranking. More heterogeneous patterns emerged for injuries; for instance, road injuries and interpersonal violence each rose in reflective ranks from 2005 to 2015, despite significant reductions in total DALYs, all-age DALY rates, and age-standardised DALY rates from each cause.

**Figure 3:**
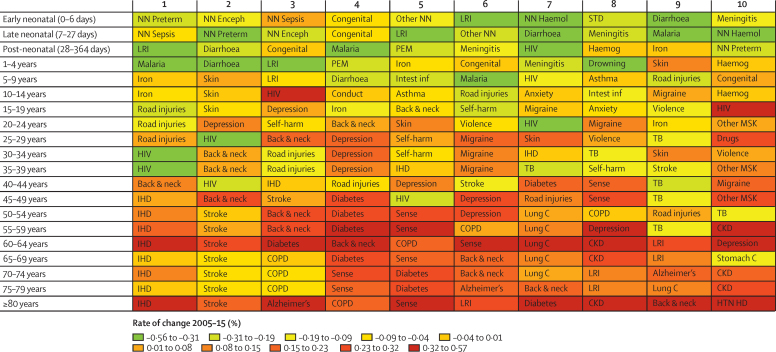
Leading ten Level 3 causes of global age-specific DALYs in 2015 Each cause is coloured by the percentage change in age-specific DALYs from 2005 to 2015. NN Preterm=neonatal preterm birth complications. NN Sepsis=neonatal sepsis and other neonatal infections. LRI=lower respiratory infections. Iron=iron-deficiency anaemia. HIV=HIV/AIDS. Back & neck=low back and neck pain. IHD=ischaemic heart disease. NN Enceph=neonatal encephalopathy due to birth asphyxia and trauma. Diarrhoea=diarrhoeal diseases. Skin=skin and subcutaneous diseases. Depression=depressive disorders. Stroke=cerebrovascular disease. Congenital=congenital anomalies. Diabetes=diabetes mellitus. COPD=chronic obstructive pulmonary disease. Alzheimer's=Alzheimer's disease and other dementias. PEM=protein-energy malnutrition. Conduct=conduct disorder. Sense=sense organ diseases. Other NN=other neonatal disorders. Intest inf=intestinal infectious diseases. Violence=interpersonal violence. NN Haemol=haemolytic disease and other neonatal jaundice. Anxiety=anxiety disorders. TB=tuberculosis. Lung C= lung, bronchial, and tracheal cancers. STD=sexually transmitted diseases excluding HIV. Haemog=haemoglobinopathies and haemolytic anaemias. CKD=chronic kidney disease. Other MSK=other musculoskeletal disorders. Drugs=drug use disorders. Stomach C=stomach cancer. HTN HD=hypertensive heart disease. GBD=Global Burden of Disease. DALYs=disability-adjusted life-years.

For at least one of their leading causes of DALYs in 2015, most age groups under 40 years old had a more than 31% decrease in total burden (figure 3). The causes for which DALYs largely declined included lower respiratory infections, diarrhoeal diseases, malaria, preterm birth complications, and drowning among children younger than 5 years, and HIV/AIDS and malaria for people between the ages of 5 years and 40 years. Increases in cause-specific DALYs varied more by age, with rising DALYs due to depressive disorders and drug use disorders for populations at age 20–30 years. DALYs from low back and neck pain increased since 2005 for many age groups. For people 60 years and older, several causes, including ischaemic heart disease, chronic kidney disease, diabetes, hearing loss, and Alzheimer's disease and other dementias, ranked among the leading causes of DALYs in 2015 and caused more DALYs than in 2005.

### Regional and country-specific HALE

HALE at birth was highest for men in Singapore (72·3 years [95% UI 70·1–74·2]) and for women in Andorra (76·3 years [72·8–79·4]) in 2015. It was lowest in Lesotho for both men (39·1 years [34·3–44·7]) and women (43·8 years [37·9–49·8]; table 2; appendix p 12). HALE at birth in 2015 exceeded 70 years in only 14 geographies for men, while 59 countries and territories surpassed this threshold for women. 13 countries and territories had HALE lower than 50 years for either sex. Since 2005, 121 countries and territories had significant increases in HALE at birth for men and 139 for women, led in both cases by Zimbabwe, whereas HALE at birth worsened for two countries (Syria and Libya) driven mainly by decreases in life expectancy (table 2). HALE at age 65 years was highest in Andorra for both men (15·2 years [UI 13·9–16·3]) and women (19·4 years [17·8–20·8]) in 2015, whereas the lowest HALE for men was in Lesotho (6·9 years [5·2–9·4]) and for women in Afghanistan (6·9 years [5·8–8·2]).

### Epidemiological transition

**Figure 4:**
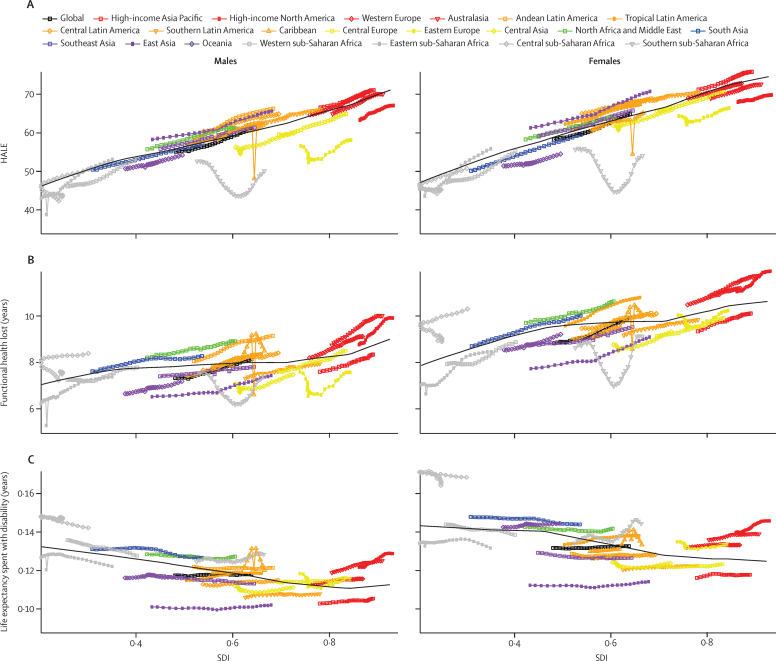
Co-evolution of HALE (A), functional health lost (life expectancy minus HALE; B), and life expectancy spent with disability (life expectancy minus HALE, divided by HALE; C) with SDI globally and for GBD regions, 1990 to 2015 Coloured lines show global and region values for each metric. Each point in a line represents 1 year starting at 1990 and ending at 2015. In all regions, SDI has increased year on year so progress in SDI is associated with later years for a given region. The black lines indicate trajectories for each geography expected on the basis of SDI alone. GBD=Global Burden of Disease. SDI=Socio-demographic Index. HALE=healthy life expectancy. DALYs=disability-adjusted life-years.

HALE at birth increased continuously and in a largely linear manner with increasing SDI for both sexes (figure 4A). At a SDI of 0·20, average HALE was 46·2 years for men and 47·1 years for women; by a SDI of 0·90, average HALE was 69·8 years for men and 73·8 years for women. Among high SDI regions, North America was the furthest below expected HALE at birth for both sexes, whereas high-income Asia Pacific remained above expected HALE at birth for both sexes and over time. In Australasia, male HALE was consistently close to expected levels, whereas female HALE remained below expected levels since 1990. With the exception of the Caribbean in 2010 (the year of the Haitian earthquake) HALE at birth throughout Latin America and the Caribbean was generally higher than expected for both sexes. HALE at birth also exceeded expectations in east Asia and north Africa and the Middle East. By contrast, Oceania steadily remained below expected levels of HALE, and all regions within the central Europe, eastern Europe, and central Asia GBD super-region had HALE lower than expected over time, particularly for men. HALE trends in sub-Saharan Africa were heavily influenced by the HIV/AIDS epidemic, particularly in southern sub-Saharan Africa, where HALE was well below expected levels. Notably, after lagging below expected levels of HALE before 2005, eastern sub-Saharan Africa posted average HALE for women that exceeded expected levels. Less pronounced increases occurred for men in eastern sub-Saharan Africa, as HALE essentially equalled expected levels only around 2010 (figure 4A).

Years of functional health lost on average increased as countries developed (figure 4B). Among high-income regions, all regions except for high-income Asia Pacific consistently exceeded expected levels of functional health loss over time, and women generally experienced a higher gap than men. South Asia, north Africa and the Middle East, and central sub-Saharan Africa had generally higher-than-expected functional health loss, whereas a number of regions, including Oceania, southeast Asia, and east Asia, all recorded smaller gaps between life expectancy and HALE than was expected.

The ratio of years of functional health loss to life expectancy, the proportion of life expectancy spent with disability, declined slightly with increasing SDI (figure 4C). Among men and women, central sub-Saharan Africa had the highest proportion of life spent with disability in 2015, although high-income North America had the largest difference between observed and expected levels for that year. For both sexes, several regions showed higher-than-expected proportions of life expectancy spent in ill health (eg, south Asia, north Africa and the Middle East, and Australasia), whereas others experienced lower-than-expected levels over time (eg, southeast Asia, east Asia, eastern sub-Saharan Africa, and southern Latin America).

**Figure 5:**
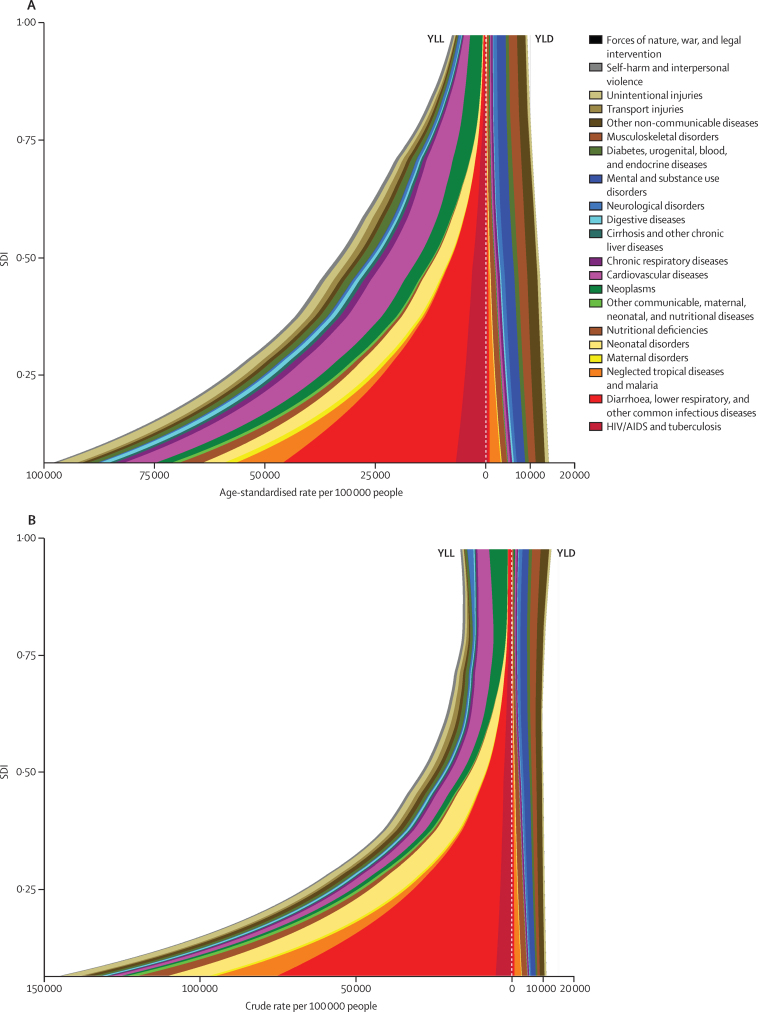
Expected relationship between age-standardised YLL and YLD rates and SDI (A) and all-age YLL and YLD rates (per 100 000) and SDI (B) for 21 GBD Level 2 causes These stacked curves represent the average relationship between SDI and each cause observed across all geographies over the time period 1990 to 2015. In each figure, the y axis goes from lowest SDI to highest SDI. The left side shows rates for YLLs and the right side shows rates for YLDs; higher rates are further from the midline. The difference between (A) and (B) is the effect of shifts in population age structure expected with SDI. GBD=Global Burden of Disease. SDI=Socio-demographic Index. YLDs=years lived with disability. YLLs=years of life lost.

Expected age-standardised YLL rates for many communicable causes and neonatal conditions declined profoundly as SDI increased (figure 5A). At the same time, age-standardised YLD rates for the leading causes of YLDs such as mental and substance abuse disorders and musculoskeletal disorders demonstrate relatively little change. At higher SDIs, the composition of disease burden shifted toward YLDs as the primary driver of burden, mainly due to the differential pace of change. The combined effect of the change in age-specific rates and age-structure change that occur with development is shown in figure 5B, which provides expected all-age YLL and YLD rates for Level 2 causes. Demographic shifts in age-structure potentiate and accelerate the transition from Group 1 conditions toward NCDs in terms of the composition of the burden of disease that health systems must handle. Of note, from an SDI of 0·8 onwards further declines in the age-standardised rates are matched or exceeded by increases in population age structure so that all-age rates for YLDs actually increase, as do YLL rates for some causes such as neurological conditions. These characterisations of the epidemiological transition demonstrate the double burden of communicable diseases and NCDs for populations with an SDI in the intermediate range.

### Observed versus expected total and cause-specific burden

**Figure 6:**
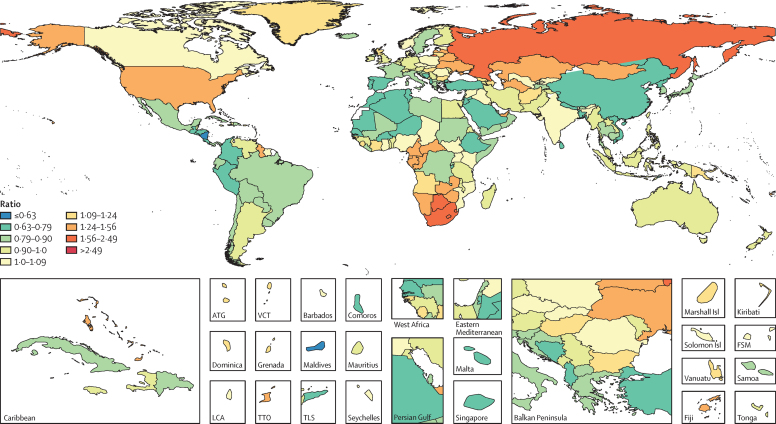
Ratio of observed versus expected age-standardised DALY rates (per 100 000) on the basis of SDI alone for both sexes combined, 2015 Ratios are colour-coded in terms of the magnitude of differences between observed and expected all-ages DALY rates. Blues indicate much lower observed DALYs than expected levels based on SDI, whereas reds reflect that observed DALYs far exceed expected levels given SDI. ATG=Antigua and Barbuda. VCT=Saint Vincent and the Grenadines. LCA=Saint Lucia. TTO=Trinidad and Tobago. TLS=Timor-Leste. FSM=Federated States of Micronesia. SDI=Socio-demographic Index. DALY=disability-adjusted life-years.

In 2015, the Maldives and Nicaragua had the lowest ratios of observed to expected all-ages DALY rates; many countries throughout Latin America also had lower-than-expected all-ages DALY rates (figure 6). Other regions where observed all-ages DALY rates fell below expected levels, included western Europe (eg, Portugal, Spain, France, Italy, and Sweden); western sub-Saharan Africa (eg, Burkina Faso, Niger, and Senegal); eastern sub-Saharan Africa (eg, Burundi and Ethiopia); north Africa and the Middle East (eg, Jordan, Saudi Arabia, and Turkey); east Asia (eg, China); and a subset of countries in South and Southeast Asia (eg, Bangladesh, Sri Lanka, and Vietnam). By contrast, observed all-ages DALY rates exceeded expected levels in southern sub-Saharan Africa, much of central Asia and eastern Europe, and a number of countries in central sub-Saharan Africa. Notably, observed all-ages DALY rates surpassed expected levels in the USA.

**Figure 7:**
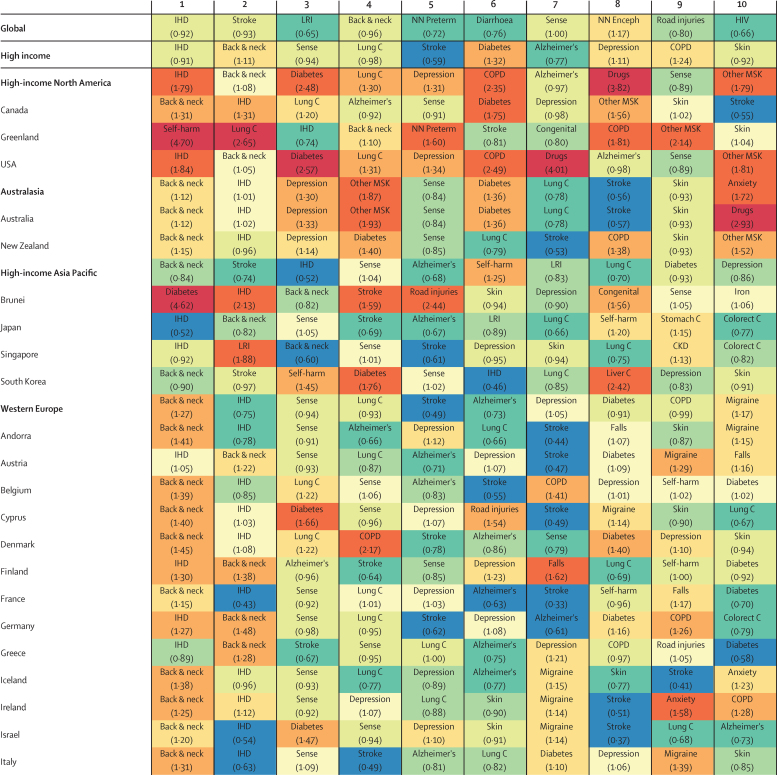

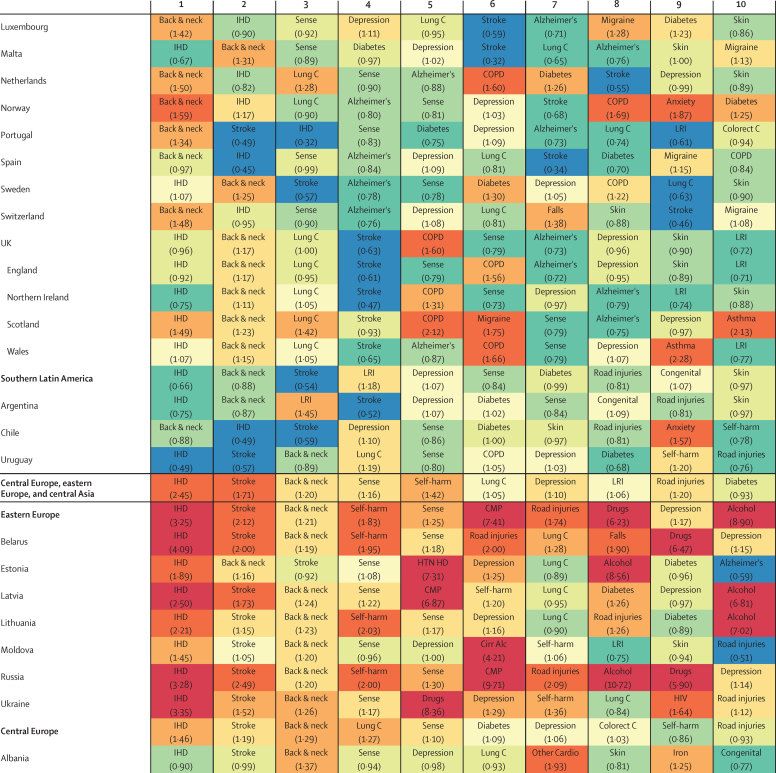

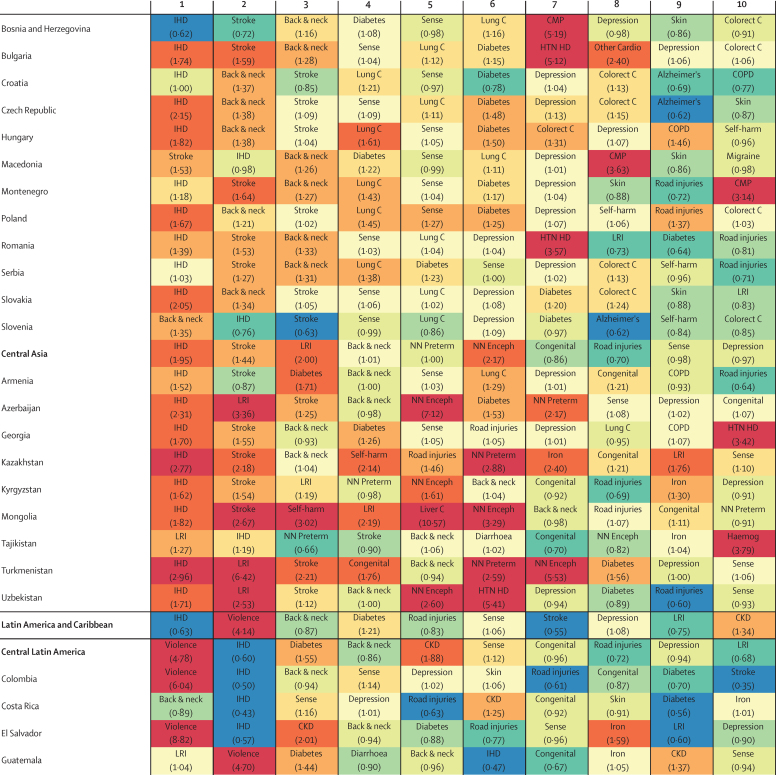

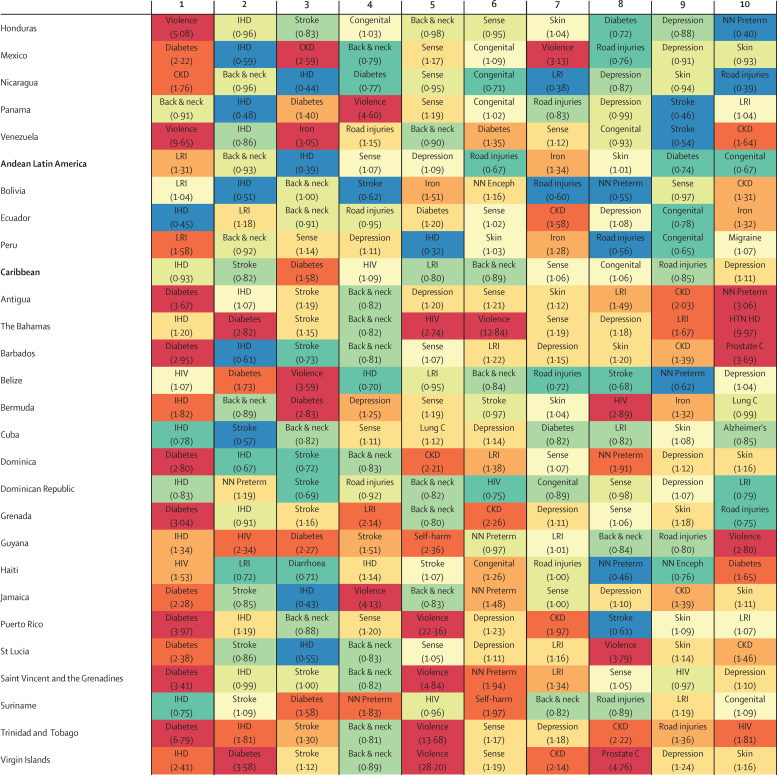

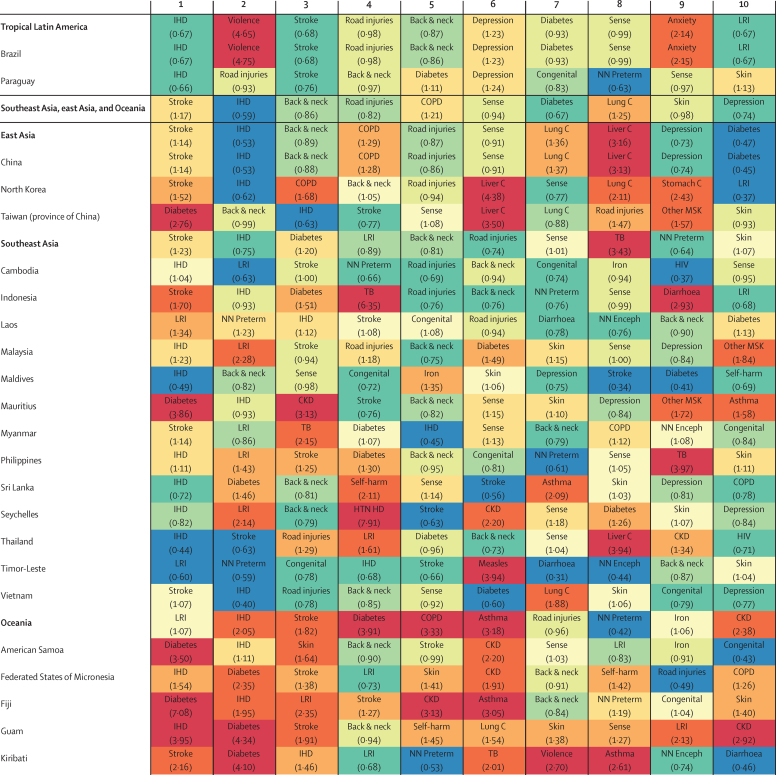

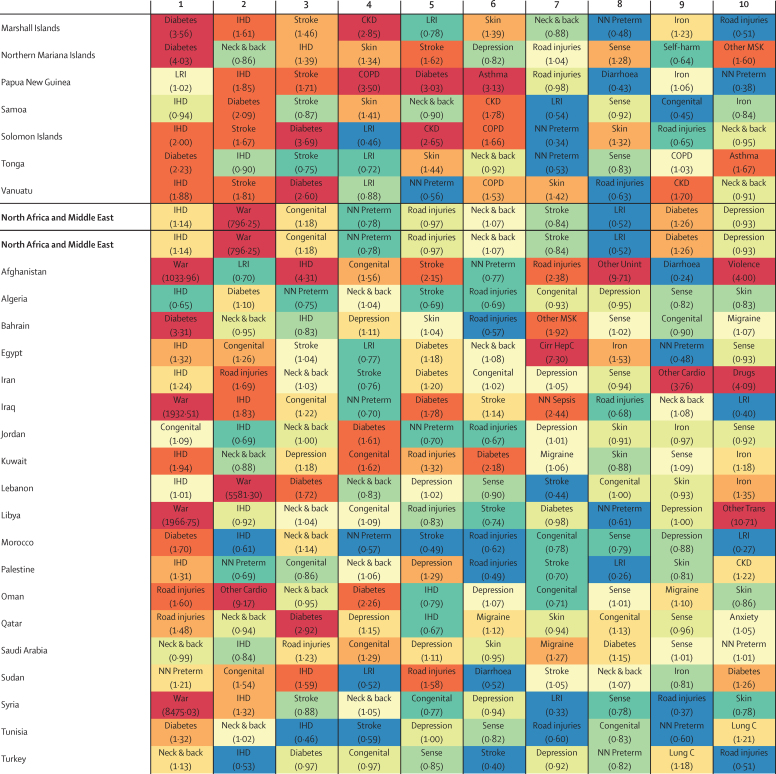

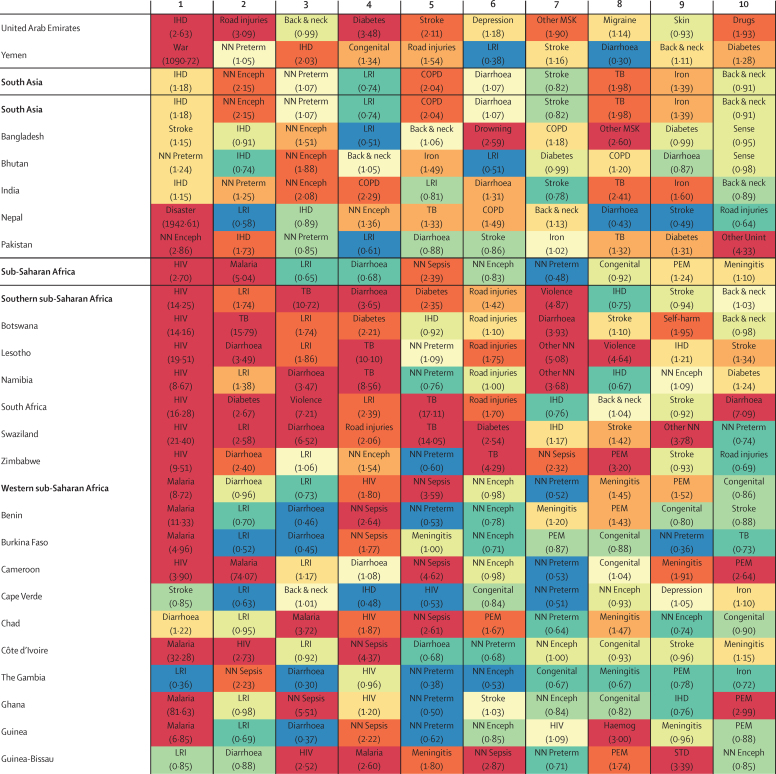

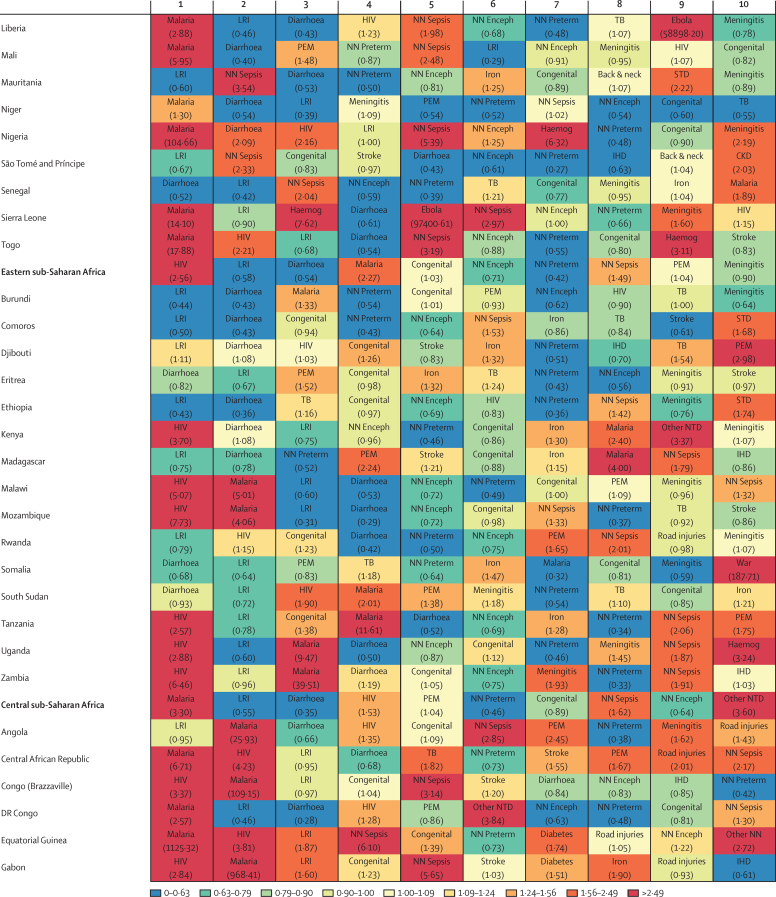
Leading ten causes of DALYs with the ratio of observed DALYs to DALYs expected on the basis of SDI in 2015, by location The ratio of observed DALYs to DALYs expected based on SDI is provided in brackets for each cause and cells are colour coded by ratio ranges (calculated to place a roughly equal number of cells into each bin). Shades of blue represent much lower observed DALYs than expected levels based on SDI, whereas red shows observed DALYs that exceed expected levels. IHD=ischaemic heart disease. Back & neck=low back and neck pain. Diabetes=diabetes mellitus. Stroke=Cerebrovascular disease. Lung C=lung, bronchus, and trachea cancers. Sense=sense organ diseases. Depression=depressive disorders. Alzheimer's=Alzheimer's disease and other dementias. Oth MSK=other musculoskeletal disorders. COPD=chronic obstructive pulmonary disease. NN Preterm=neonatal preterm birth complications. Diarrhoea=diarrhoeal diseases. Skin=skin and subcutaneous diseases. NN Enceph=neonatal encephalopathy due to birth asphyxia and trauma. Drugs=drug use disorders. Congenital=congenital anomalies. Liver C=liver cancer. Stomach C=stomach cancer. CKD=chronic kidney disease. Anxiety=anxiety disorders. Iron=iron-deficiency anaemia. HIV=HIV/AIDS. Colorect C=colon and rectum cancer. LRI=lower respiratory infections. Cirr HepC=cirrhosis due to hepatitis C. CMP=cardiomyopathy and myocarditis. Cirr alc=cirrhosis due to alcohol use. Other Cardio=other cardiovascular and circulatory diseases. Alcohol=alcohol use disorders. Violence=interpersonal violence. HTN HD=hypertensive heart disease. Haemog=haemoglobinopathies and haemolytic anaemias. TB=tuberculosis. Prostate C=prostate cancer. War=collective violence and legal intervention. Other Unint= other unintentional injuries. Oth trans=other transport injuries. Other NN=Other neonatal disorders. NN Sepsis=neonatal sepsis and other neonatal infections. PEM=protein-energy malnutrition. STD=sexually transmitted diseases excluding HIV. Ebola=Ebola virus disease. Other NTD=other neglected tropical diseases. GBD=Global Burden of Disease. SDI=Socio-demographic Index. DALYs=disability-adjusted life-years.

Ischaemic heart disease and stroke were the leading two causes of DALYs worldwide in 2015, and 106 geographies also had one of these diseases as the leading cause of DALYs that year (figure 7). Four GBD super-regions showed deviations from this trend: Latin America and the Caribbean, where diabetes and interpersonal violence often resulted in the most DALYs; north Africa and the Middle East, where war was a primary cause of burden; south Asia, where neonatal disorders often ranked among the leading causes of DALYs; and sub-Saharan Africa, where HIV/AIDS or malaria was the leading driver of disease burden in 29 geographies.

Stroke resulted in the most countries (94) having lower observed DALYs than expected based on their SDI. Other leading causes for which observed DALYs were well below expected levels included ischaemic heart disease particularly in Latin America, east Asia, and southeast Asia; road injuries in north Africa and the Middle East; and lower respiratory infections and diarrhoeal diseases in sub-Saharan Africa. Many high-income countries and territories also had lower-than-expected DALYs from ischaemic heart disease and Alzheimer's disease and other dementias. Road injuries accounted for lower burden than expected in 52 countries and territories, especially in Colombia. Although many Group 1 causes remained among the leading causes of DALYs, observed levels were often lower than expected on the basis of SDI (eg, lower respiratory infections in Ethiopia, diarrhoeal diseases in Afghanistan, and preterm birth complications in Kenya).

By contrast, diabetes was a leading cause for which observed burden exceeded expected levels in many geographies, especially in Oceania and the Caribbean. Observed DALYs due to chronic obstructive pulmonary disease were higher than expected in 30 geographies, as were those due to liver cancer and lung cancer for a subset of countries and territories. Drug use disorders led to more observed DALYs than expected in many high-income countries in 2015, particularly in the USA and Australia. A similar pattern occurred for self-harm, cirrhosis, alcohol use disorders, and drug use disorders throughout eastern Europe, and most prominently in Russia. Interpersonal violence was among the leading two causes of DALYs for six of 11 countries in central and tropical Latin America (Brazil, Colombia, El Salvador, Guatemala, Honduras, and Venezuela), and each had observed burden far surpassing expected levels. Throughout sub-Saharan Africa, HIV/AIDS and malaria resulted in far more DALYs than expected based on SDI.

Heterogeneous trends across and within regions emerged in terms of both leading causes of DALYs and ratios of observed to expected levels. For instance, south Asia's disease burden landscape diverged from global patterns, with both ischaemic heart disease and neonatal disorders ranking among some of the leading causes of burden and often resulting in higher-than-expected DALYs (figure 7). Stroke and lower respiratory infections resulted in fewer DALYs than expected for most countries in south Asia, yet other causes exacted more DALYs than expected on the basis of SDI (eg, tuberculosis in India and drowning in Bangladesh). Further, the 2015 Nepal earthquake resulted in forces of nature being its leading cause of DALYs that year. Many countries in central Asia experienced observed DALYs that surpassed expected levels due to both Group 1 causes (eg, lower respiratory infections, preterm birth complications, and neonatal encephalopathy) and NCDs such as hypertensive heart disease. War was the leading cause of DALYs in five countries in north Africa and the Middle East in 2015, including Afghanistan, Iraq, Libya, Syria, and Yemen. Although neonatal sepsis frequently led to higher-than-expected DALYs in sub-Saharan Africa, burden from preterm birth complications fell below expected levels, on the basis of SDI, in most countries. Notably, NCDs such as diabetes ranked among the leading ten causes of DALYs for a subset of countries in sub-Saharan Africa (eg, South Africa), whereas nutritional deficiencies also remained among leading causes of burden in others (eg, Ghana and Zimbabwe); in both instances, observed DALYs generally exceeded expected levels.

## Discussion

GBD 2015 results show that the world has become healthier in the past 25 years. Yet, this progress has not been universal. From 1990 to 2015, global HALE at birth increased from 56·7 years to 62·8 years, with 191 of 199 countries or territories recording improved HALE. Since 1990, global HALE at age 65 years also improved by 1·8 years, with an increase in 179 countries or territories. The global number of years of functional health lost grew during this time, from 8·2 years to 9·1 years. With YLL rates falling at a much faster pace than YLD rates, non-fatal health loss accounted for an increasing proportion of global DALYs, rising from 21·2% in 1990, to 32·1% in 2015. Worldwide progress was largely driven by rapid reductions in DALYs from communicable, maternal, neonatal, and nutritional diseases, although declines in age-standardised DALY rates from NCDs and injuries also contributed to overarching gains. Despite reductions in age-standardised DALY rates, 137 causes had statistically significant increases in total DALYs since 2005, a trend with extensive implications for health systems. Mental and substance use disorders, musculoskeletal disorders, and a range of other conditions including idiopathic developmental intellectual disability, vision and hearing impairment, and neurological disorders all saw rising disease burden since 2005, and few saw any evidence of declining age-specific YLD rates.

Considerable research and policy attention has considered the existence of compression of morbidity, or whether people live healthier lives as their lifespans extend.^30^ Beyond its profound consequences for financing health systems, compression of morbidity has considerable implications for societal structures and expectations about longevity of careers or timing of retirement. Compression can be interpreted in both absolute and relative terms. Absolute compression implies that as people live longer lives, they lose fewer years due to functional health loss, whereas relative compression implies that as people live longer lives, the ratio of years of functional health lost to life expectancy declines. Although some evidence shows that compression occurs among people with specific diseases such as diabetes and dementia,^31^, ^32^ national studies show more mixed results.^33^, ^34^, ^35^ This might not be surprising, as most national studies rely on self-reported health status and chronic conditions, which are then further complicated by variations in how individuals use the response scales and profound framing effects.^36^, ^37^, ^38^, ^39^ By contrast, the GBD study provides a more comprehensive and comparable assessment of changes in functional health status by synthesising many types of data, by cause, and applying standardised disability weights to reflect the public's average views of severity of different conditions. GBD 2015 results unequivocally show that as life expectancy increases, people spend more time with reduced functional health status, and thus absolute expansion of morbidity has occurred. This trend is driven by marked declines in age-specific mortality at the same time minimal improvement, if any, has occurred for age-specific YLDs per capita. The proportion of lifespans spent in ill health has remained comparatively constant since 1990, and did not vary as a function of SDI; thus, we found nominal evidence of relative compression at the global level.

Drawing from our empirical characterisation of epidemiological transitions on the basis of SDI, we found that life expectancy and HALE increased linearly with SDI, whereas years of functional health lost climbed with rising SDI. Historically, increases in SDI are associated with a rapid decrease in burden from communicable, neonatal, maternal, and nutritional diseases, the leading killers of children, adolescent girls, and women. Efforts to increase income, provide more years of education, and reduce adolescent and total fertility rates thus might catalyse additional gains for life expectancy, HALE, and reduced disease burden, emphasising the critical role of policy interventions beyond more traditional health service delivery.

At different locations in this continuous process of change, we see evidence of a so-called double burden of disease: from a SDI of approximately 0·35 to 0·60, we expect NCDs and Group 1 causes to each account for at least 20% of disease burden. The average relationships with SDI imply that within a country with wide inequalities in SDI, we should also expect wide variation in disease burden patterns. Subnational disparities might be consistent with variations in subnational SDI, either recording higher or lower burden than that of the national level. Use of average patterns can help to benchmark a country against others, but such assessments can also help to provide insights as to whether public action or other factors are helping make inequalities narrower than expected based on SDI alone. Given the complexity of health patterns identified for many causes and differential patterns by age and sex, providing some understanding of expected patterns on the basis of SDI alone can help to anchor the exploration of results and could provide some measure of the performance of health systems or the magnitude of avertable burden within each country or territory.

The *Lancet* Commission on Investing in Health^40^ galvanised considerable interest in the notion of a “grand convergence”, such that levels of under-5 mortality, maternal mortality, and some infectious diseases could converge across all countries within a generation. Convergence can be achieved through progress on increasing SDI (ie, increasing per-capita income and average years of schooling, and reducing fertility) and reducing or inverting high ratios between observed and expected (based on SDI alone) health expectancies and health gaps. The Commission has argued that, within a generation, preventable deaths in children and mothers could largely be avoided through increased investment of development assistance for health and expanded national expenditure on health. The vision inspired some of the absolute threshold SDG targets, including reducing under-5 mortality to 25 deaths per 1000 livebirths, neonatal mortality to 12 deaths per 1000 livebirths, and maternal mortality ratio to 70 deaths per 100 000 livebirths. The relationships between these health outcomes, broader health measures, and SDI offer a framework by which the likelihood of such a grand convergence can be assessed. Based on GBD 2015 results, the historical relationship between SDI and health suggests that convergence in the sense of reduced absolute differences in rates is likely to occur with faster improvements in SDI. However, if convergence means smaller relative differences, then improvements in SDI alone might not be sufficient. Our findings show that continued SDI improvement does not appear to be historically associated with absolute convergence in life expectancy or HALE. The *Lancet* Commission also emphasised the importance of hastening progress through strategic investments by donors and governments in effective health technologies, an approach that has the potential to catalyse faster progress than what would be expected on the basis of SDI alone. Convergence in this scenario could then be interpreted as reduction in the ratio of observed to expected burden for low-income and lower-middle-income countries (LMICs), and could be used as an indirect, but summary, metric to monitor health system performance and overall progress toward the SDGs. Since history provides a perspective for identifying which countries have been able to reduce their ratio of observed to expected health outcomes, the comprehensive and longitudinal approach of the GBD is optimally suited for monitoring health system-driven convergence at a macro-level. The same tools can also therefore be used to generate insights on progress, or lack thereof, on specific diseases and outcomes of interest. In some cases, there might be historical or geographical explanations for high burden for some conditions. In other cases, effective preventive and treatment measures have just not yet been implemented or are not functioning effectively.

Shifting from the MDGs to the SDGs dramatically broadened the global health agenda.^2^, ^3^ The SDGs include 17 goals, 169 targets, and 230 indicators; of these measures, 11 goals, 28 targets, and 46 indicators are health related.^41^ At present, 33 of the 46 health-related indicators are measured by the GBD study. Amid earlier discussions and negotiations over SDG 3 indicators, HALE was proposed as an indicator of overarching health status and progress;^2^ this proposal was not ultimately adopted in the final set of indicators. HALE provides a strong summary measure of overall health status because it accounts for functional health loss in addition to age-specific mortality. Other summary development measures, such as the Human Development Index, have considered replacing life expectancy with HALE as an input to the overall assessment.^42^ The GBD study measures health outcomes that are both amenable to intervention and could be risk standardised, thus offering a useful set of metrics to monitor progress toward specific SDG targets, such as the aim of target 3.8 to achieving effective universal health coverage.

DALYs and other health-gap metrics are one of many potential inputs when setting health policy and investment priorities, but major research organisations and funders such as the US National Institutes of Health and others note the use of DALYs to inform budgeting decisions.^43^, ^44^, ^45^, ^46^ Beyond health metrics, many other inputs are required for decision making, ranging from the effectiveness of different adoptable policies and programmes, to key social, cultural, and ethical considerations. Nonetheless, DALYs and other summary health measures might have an even more prominent role in the future in setting research and development priorities within the health sector, particularly in the absence of robust information on the effectiveness or likely success of various research projects and programmes.^47^ As global health research funders increasingly use DALYs to shape priority-setting processes, health challenges faced by populations with less health-care market power, namely poor people, will inevitably receive more attention. Shifting to the use of disease burden for programme design and evaluation would benefit poor people, but also potentially increase overall efficiency of health research.^48^ For instance, individuals who have historically underfunded conditions, such as mental health disorders, substance use, and musculoskeletal conditions, would benefit from the greater use of DALYs in decision-making processes.

Global progress has been especially rapid in reducing disease burden due to a number of communicable diseases, including diarrhoeal diseases, lower respiratory infections, tuberculosis, syphilis, typhoid, paratyphoid, and vaccine-preventable infections such as hepatitis B, measles, tetanus, and *Haemophilus influenzae* type b. To these successes, the last decade has seen profound declines in the burden from malaria and HIV/AIDS. NCD trends have been much more complicated. For the leading cause of disability, low back and neck pain, a lack of knowledge about risks limits the opportunity for prevention. Occupational ergonomic factors and high body-mass index (BMI) are estimated to be responsible for 30·9% (29·2–32·5) and 5·5% (3·4–7·6) of YLDs due to low back pain, respectively.^49^ The highest occupational risk is found in service industries and manual labour, especially agriculture.^50^, ^51^ The relatively small proportion of low back pain that is caused by high BMI is amenable to intervention, but the continued escalation of obesity rates indicates that these measures might have little effectiveness. With increasing SDI, the proportion of the workforce in agriculture would be expected to become smaller, which would have some effect on the burden of low back pain. Yet, based on our analyses, nearly 65% of the burden would remain. The management of most low back and neck pain is largely focused on pain relief and prevention of worsening outcomes through physical therapy and exercise; given the very large burden and the associated economic consequences of lost work time, low back and neck pain should be a priority for research to identify more effective preventive and therapy measures.^52^ Similarly, despite broad decreases in age-standardised rates of injury burden, the pace of progress for these causes has been comparatively slow and ultimately has led to minimal changes in the proportion of overall burden due to injuries during the past 25 years. Prevention of injuries requires strong public safety policies,^53^ but minimising mortality and long-term disability from injuries hinges upon having comprehensive trauma care systems^54^, ^55^ that provide timely, evidence-based care,^56^, ^57^, ^58^ including emergency surgical services.^59^, ^60^

In 2015, sense organ disorders were the second-leading cause of YLDs and resulted in more than 68 million DALYs. Reducing DALYs from vision impairment is achievable through vertically integrated programmes, including the delivery of eyeglasses for refractive error, curative surgery for cataracts, and onchocerciasis and trachoma prevention. Given the availability of cost-effective interventions, greater policy attention is needed for vision loss burden. Although interventions for hearing loss are less clear, the use of timely antibiotics for otitis media and meningitis and the provision of hearing aids for conductive hearing loss are likely to reduce its burden.

Reductions in age-standardised DALY rates due to some NCDs such as cardiovascular diseases, most cancers, chronic respiratory diseases, and many digestive diseases—some of which can be attributed to reductions in risk factors such as tobacco and improvements in cause-specific treatment and event survival—mask the effects of population ageing. This means more people had disease burden from these causes and total DALYs have remained largely unchanged (eg, chronic obstructive pulmonary disease) or significantly increased (eg, cardiovascular diseases, cancers, neurological disorders, diabetes, chronic kidney disease, and musculoskeletal disorders such as osteoarthritis and low back and neck pain) over time. As demographic transitions are widely expected to continue, the burden from NCDs is likely to continue expanding. Widespread efforts must continue to enact societal and environmental policies to reduce risk factor exposure, while national and local health systems must adapt to meet the prevention, screening, and treatment needs of their populations. As we now recognise many of the risk factors related to NCDs, low-SDI and middle-SDI countries could adopt policies to circumvent the mistakes made by other countries as they progressed along the SDI spectrum. Mental and substance use disorders are a particularly challenging group of conditions with non-trivial levels of disease burden in all geographies. Some countries provide excellent mental health resources, whereas others, particularly LMICs, do not have screening or treatment programmes. Addressing the growing burden and disparity in mental health disorders will be an especially pressing challenge during the SDG era.

A number of emerging and growing health threats also deserve special attention in policy planning, including infectious diseases such as outbreaks of dengue fever, Ebola virus disease, Zika virus, and pandemic influenza, and antimicrobial-resistant pathogens, which represent acute threats to life and highlight health-system deficiencies where they occur; substance abuse disorders, particularly of opioids and cocaine, in eastern Europe, Australia, and North America; and intentional firearm injuries, especially in Latin America, the USA, and South Africa. In the case of dengue fever and potentially other yet to be identified infectious diseases, urbanisation and global environmental change have contributed to an increased incidence and future climate change scenarios depict a rising trend in the coming years.^61^ Other emerging infectious diseases, including Zika and chikungunya viruses, have yet to be comprehensively analysed by the GBD.

A major change in the GBD 2015 assessment has been the closer integration of the assessments of mortality and disease sequelae prevalence in modelling. For cancers, HIV/AIDS, and injuries, previous iterations of the GBD modelled mortality, incidence, and prevalence in a coherent manner. For some other diseases, the modelling of disease prevalence and cause-specific mortality rates largely used different data sources and modelling techniques. Independent estimation of prevalence and mortality led in some cases to patterns across locations of excess mortality rates that were not consistent with expected relationships related to health-system access. For GBD 2015, we built the modelling of cause-specific mortality, excess mortality, incidence, and prevalence into nearly every cause. The effect of this approach has led to increases in the number of DALYs from injuries due to YLDs and changes in prevalence for other conditions, particularly those with minimal data for prevalence or incidence. More attention will be paid in future iterations of the GBD study to identifying unpublished data from cohort studies or linkage studies on levels of excess mortality by age and sex, especially in LMICs, to further strengthen modelling efforts.

A major development for the GBD 2015 has been adaptation of the GATHER guidelines endorsed by WHO, the Institute for Health Metrics and Evaluation (IHME), and other organisations.^22^, ^23^ GATHER compliance, including the sharing of statistical code for each of the many analytical steps in the GBD, provides a new level of transparency for the overall enterprise. We expect that many researchers will want to investigate, propose improvements, and provide alternative assessments for many components of the GBD. We welcome the debate that will follow on the best way to analyse different components of the GBD. We believe enhanced transparency will lead to healthy debate and exchange and to improved methods, data, and results for many aspects of the GBD. Transparency will not necessarily lead to consensus but it will broaden everyone's understanding of the available evidence on descriptive epidemiology. Adoption by the GBD of the GATHER guidelines will hopefully stimulate other organisations to adopt the guidelines in all aspects of their work as well.

Although the volume of input data to the GBD has continued to increase substantially, major data gaps remain.^12^, ^20^, ^62^, ^63^ Geographical and temporal coverage of all-cause and cause-specific mortality datasets are variable, as is the quality of the data contained in such systems. Development of methods to report overall evidence grades for each outcome–location–year combination would be valuable to help to guide strategies for improving data quality and closing data gaps. Investing to develop and improve cause of death and vital registration systems is crucial to improve the quality of insights from the GBD; incorporation of existing data from existing and new collaborators that are not currently in the GBD is another important aspect of this effort. Several countries have experienced significant recent turmoil, especially armed conflict in Syria, Yemen, and other countries in north Africa and the Middle East. Burden from many conditions is believed to have increased during and following those events, but due to disruption of data-collection systems, the full effect of such events has been difficult to quantify. For non-fatal health outcomes, some of the most notable data gaps pertain to aspects of individual diseases and injuries that are not typically included as part of standard epidemiological reports such as distribution of symptoms for those with chronic conditions at various stages of illness, duration of disability following acute events, or long-term disability after major acute injuries. Therefore, our recommendations regarding data gaps pertaining to non-fatal health loss are twofold. First, reports and scientific journals should strive to include reporting on functional health status including severity, distribution, and duration of symptoms with all epidemiological studies. Second, countries should work to centralise and compile existing non-fatal health data and invest to collect population-level epidemiological data on important causes of YLDs.

The iterative and now annual cycle of the GBD revisions provide opportunities to improve the estimation or scope of the GBD. Due to the high interest in Zika virus, we believe that we should try to quantify the burden related to Zika virus disease in the GBD 2016 analysis. Given the focus in the SDGs on various forms of sexual violence, we believe careful investigation of the evidence base for estimation is warranted. As noted in the GBD papers on mortality and non-fatal outcomes,^12^, ^20^ there are also a number of opportunities to improve data processing and estimation methods that will be explored for the next cycle of estimation. We also expect to include more subnational analyses, particularly for large countries.

Our analysis has several limitations. First and foremost, the calculation of DALYs and HALE reflects the limitations of all the underlying analyses of the GBD, including all-cause mortality, cause-specific mortality, prevalence, incidence, disability-weight derivation, and simulation of comorbidity. Second, as discussed above, data limitations are apparent in a number of facets of our analysis. Third, inherent to the GBD approach is the effort to quantify specific sequelae of each disease and injury. This means that the full disease burden of certain conditions such as heart failure, anaemia, vision and hearing loss, infertility, epilepsy, and intellectual disability are not as readily apparent in high-level review of the GBD results. However, the YLDs for these impairments are reported elsewhere.^20^ Fourth, our analysis of the relationship between SDI, DALYs, and HALE reflects the average historical relationship between SDI and each measure, so despite often strong correlation with SDI it cannot be interpreted as being causal in nature. In some cases, association of SDI with health indicators could be considered a confounder when the same elements (education, income, and fertility) are used to develop both the index and as a covariate in cause-specific models. SDI utility might be improved in the future through consideration of additional societal elements such as inequality in each component. Other measures that capture the status of women in society, such as the female labour-force participation, could be considered in future revisions. Fifth, we have assumed independence of uncertainty between YLLs and YLDs as well as between YLDs and life expectancy. Empirical evidence to guide alternative assumptions, however, is currently very limited. Sixth, recent events in Syria and Libya and the resulting mass migration have led to considerable health loss, including drownings of many migrants. New migrants have different health problems than the populations of the countries to which they have moved. Both the drownings and the change in health status in countries receiving migrants are not adequately captured in this assessment due to the time-lags in data collection and data capture intrinsic in all health data systems. Seventh, estimates of expected burden from SDI alone are based on the average levels of burden at each level of SDI. For endemic diseases, comparisons of observed rates to expected rates will lead to high observed over expected ratios in endemic countries and low ratios in non-endemic countries. Interpretation of the ratios for conditions that are endemic in only some countries needs to take this into account.

WHO has estimated DALYs by cause for the single years of 2000 and 2012.^13^, ^17^ They used published GBD 2010 results used to generate WHO 2012 DALYs for 132 causes with some modifications. First, WHO life tables were used instead of GBD life tables.^14^ WHO life tables are different from the UN Population Division life tables and use a set of methods developed by Murray and colleagues in the late 1990s;^11^, ^64^ this approach does not benefit from the many improvements in data processing and estimation methods that have emerged in the last 15 years.^12^, ^65^, ^66^ Second, WHO altered the empirical disability weights, which were derived from an international sample of more than 60 000 respondents from the GBD analysis^26^ for 32 outcomes using the opinions of 45 respondents.^13^, ^67^ Third, WHO calculated YLLs after changing from the GBD 2010 normative standard life expectancy of 86·0 years to 91·9 years.^68^ Fourth, rather than using GBD results, WHO elected to use alternative estimates for 12 causes of death, including tuberculosis, HIV/AIDS and other sexually transmitted infections, malaria, whooping cough, measles, schistosomiasis, maternal disorders, cancers, alcohol and drug use disorders, epilepsy, conflict and natural disasters, and road traffic accidents.^13^ Finally, WHO substituted prevalence estimates produced internally for vision loss, hearing loss, intellectual disability, infertility, anaemia, back pain, alcohol use disorders, headache, and skin diseases. The final hybrid estimates of DALYs do not provide uncertainty measures and have not been peer-reviewed.

WHO has also produced HALE estimates for three time periods, 2000, 2012, and 2015, using GBD 2010 results as described above for 2000 and 2012, and GBD 2013 results for 2015, also without uncertainty measures.^14^ Differences between their estimates and those for HALE from GBD 2015 reflect changes in age-specific YLDs per capita from GBD 2013 to GBD 2015 and differences in WHO life expectancy (appendix p 85). The EC and the OECD also report healthy life expectancy estimates based on self-reported health status from 2004 through 2014, but without specific consideration of prevalence or incidence of disease.^52^, ^53^ Comparison of 2014 estimates from the EC and GBD mostly show lower estimates from EC (appendix pp 86–87). EC estimates also report much wider ranges in HALE across countries in Europe than those estimated through GBD. Further, in a number of countries, EC estimates point to lower HALE among women than for men. These differences, both in terms of absolute estimates and those by sex, are probably due to the inclusion of non-health factors in self-reported assessments of disability.

In conclusion, HALE has increased steadily throughout the world over the MDG era, with a concomitant decrease in age-standardised DALY rates from most conditions. Declines occurred in overall health loss due to many communicable, maternal, neonatal, and nutritional diseases. Much of the evolution of health is consistent with the expected changes in disease burden with development that have been quantified in this study. Substantial variation in burden compared with levels expected on the basis of SDI suggests wide heterogeneity in the ability of governments and health systems to adequately meet the health needs of their populations. Progress in reducing these gaps will be crucial to achieving the ambitious SDG agenda. Demographic changes leading to increased population size and older average age have offset otherwise important gains in age-specific DALY rates leading to rising burden on health systems for many ailments of ageing. Emerging health threats and causes with lagging progress should be viewed as essential foci for investment in health infrastructure and health data systems to improve the global community's insights into the aggregate quality of care and the overall health of populations.

Correspondence to: Prof Christopher J L Murray, 2301 5th Avenue, Suite 600, Seattle, WA 98121, USA cjlm@uw.edu

**This online publication has been corrected. The corrected version first appeared at thelancet.com on January 5, 2017**

## Figures and Tables

**Table 1:** Global all-age DALYs and age-standardised DALY rates in 2005 and 2015 with median percentage change between 2005 and 2015 for all causes

				**All-age DALYs (thousands)**			**Age-standardised rate (per 100 000)**		
				2005	2015	Percentage change, 2005–15	2005	2015	Percentage change, 2005–15
**All causes**				**2 553 306·8 (2 373 137·6 to 2 756 328·0)**	**2 464 895·4 (2 259 889·0 to 2 696 510·8)**	−3·5 (−5·3 to −1·8)*	**41 561·7 (38 640·8 to 44 850·4)**	**34 445·7 (31 603·0 to 37 654·3)**	−17·1 (−18·7 to −15·7)*
**Communicable, maternal, neonatal, and nutritional diseases**				**968 014·5 (931 809·4 to 1 012 327·9)**	**741 595·9 (703 928·7 to 787 659·9)**	−23·4 (−25·2 to −21·7)*	**14 297·6 (13 766·0 to 14 955·1)**	**10 007·2 (9 498·5 to 10 629·3)**	−30·0 (−31·6 to −28·5)*
	**HIV/AIDS and tuberculosis**			**148 685·6 (141 013·7 to 160 635·2)**	**106 991·8 (99 680·8 to 116 789·9)**	−28·0 (−30·6 to −25·1)*	**2296·0 (2172·7 to 2490·5)**	**1 435·5 (1 336·0 to 1 569·2)**	−37·5 (−39·6 to −35·0)*
		Tuberculosis		49 769·6 (43 196·4 to 60 348·2)	40 302·2 (34 065·8 to 49 653·9)	−19·0 (−24·2 to −13·6)*	817·0(708·5 to 990·9)	552·4(467·2 to 681·3)	−32·4(−36·7 to −27·9)*
		HIV/AIDS		98 916·1(94 515·6 to 103 953·2)	66 689·5 (63 342·9 to 70 788·3)	−32·6 (−35·2 to −29·5)*	1478·9 (1414·0 to 1553·2)	883·1 (839·2 to 937·1)	−40·3 (−42·6 to −37·5)*
			HIV/AIDS—tuberculosis	19 327·0 (15 512·5 to 22 015·1)	11 621·9 (8 955·5 to 13 412·3)	−39·9 (−44·2 to −34·5)*	289·6 (232·8 to 329·8)	154·0 (118·7 to 177·7)	−46·8 (−50·7 to −42·1)*
			HIV/AIDS resulting in other diseases	79 589·0 (74 869·9 to 85 308·1)	55 067·6 (51 480·9 to 59 294·3)	−30·8 (−34·2 to −26·9)*	1189·3 (1119·8 to 1277·1)	729·1 (681·9 to 784·8)	−38·7 (−41·7 to −35·3)*
	**Diarrhoea, lower respiratory, and other common infectious diseases**			**333 534·0 (317 202·5 to 351 569·5)**	**242 875·8 (230 350·0 to 255 919·1)**	−27·2 (−30·2 to −24·2)*	**5005·5 (4769·5 to 5269·7)**	**3326·2 (3158·6 to 3503·4)**	−33·5 (−36·2 to −30·9)*
		Diarrhoeal diseases		98 394·2 (90 909·6 to 106 634·4)	71 589·5 (66 442·9 to 77 205·8)	−27·2 (−33·2 to −20·8)*	1479·8 (1371·9 to 1598·4)	976·9 (908·1 to 1 052·7)	−34·0 (−39·3 to −28·4)*
		Intestinal infectious diseases		15 062·4 (8 707·5 to 24 651·4)	12 632·0 (7 250·5 to 20 762·5)	−16·1 (−22·9 to −9·9)*	216·0 (124·9 to 353·2)	170·8 (98·2 to 280·7)	−20·9 (−27·3 to −14·9)*
			Typhoid fever	12 543·9 (6 953·1 to 21 143·4)	10 575·6 (5 896·1 to 17 598·0)	−15·7 (−22·7 to −9·3)*	179·8 (99·7 to 302·9)	143·0 (79·8 to 237·7)	−20·5 (−26·9 to −14·2)*
			Paratyphoid fever	2393·2 (1119·8 to 4569·5)	2014·8 (960·5 to 3848·4)	−15·8 (−24·2 to −7·4)*	34·4 (16·1 to 65·6)	27·2 (13·0 to 52·0)	−20·8 (−28·7 to −12·6)*
			Other intestinal infectious diseases	125·4 (39·3 to 243·9)	41·5 (16·3 to 81·3)	−66·9 (−78·0 to −47·0)*	1·8 (0·6 to 3·5)	0·6 (0·2 to 1·1)	−69·3 (−79·5 to −51·6)*
		Lower respiratory infections		135 293·2 (127 083·7 to 143 499·4)	103 048·6 (96 128·2 to 109 078·8)	−23·8 (−28·2 to −19·4)*	2 070·3 (1 939·4 to 2 189·6)	1428·5 (1330·5 to 1511·1)	−31·0 (−34·8 to −27·1)*
		Upper respiratory infections		2 661·2 (1 550·4 to 4 406·1)	2868·4 (1650·2 to 4785·2)	7·8 (5·2 to 9·7)*	40·1 (23·5 to 66·4)	38·7 (22·3 to 64·5)	−3·5 (−5·6 to −2·1)*
		Otitis media		3444·4 (2276·8 to 5004·0)	3497·6 (2284·9 to 5088·5)	1·5 (−1·3 to 4·1)	50·9 (33·7 to 74·1)	47·3 (30·9 to 68·7)	−7·2 (−9·9 to −4·9)*
		Meningitis		28 394·6 (24 170·2 to 32 237·2)	25 394·6 (21 653·2 to 30 649·0)	−10·6 (−20·1 to 3·8)	416·8 (355·2 to 471·9)	342·4 (292·3 to 412·8)	−17·8 (−26·4 to −4·6)*
			Pneumococcal meningitis	7826·5 (6404·7 to 9672·3)	7773·4 (6353·4 to 9900·2)	−0·7 (−11·2 to 14·9)	115·3 (94·8 to 141·6)	104·9 (85·9 to 133·4)	−9·0 (−18·5 to 5·3)
			*Haemophilus influenzae* type B meningitis	8507·6 (6656·5 to 10590·4)	5345·7 (4146·7 to 7103·2)	−37·2 (−45·8 to −25·0)*	123·3 (96·7 to 153·4)	71·9 (55·9 to 95·4)	−41·7 (−49·7 to −30·5)*
			Meningococcal meningitis	5239·8 (4012·0 to 6704·3)	5065·6 (3879·5 to 6721·8)	−3·3 (−16·9 to 16·3)	76·6 (59·0 to 97·6)	68·2 (52·3 to 90·3)	−11·0 (−23·4 to 6·9)
			Other meningitis	6820·7 (5665·6 to 8234·7)	7210·0 (5953·9 to 8958·4)	5·7 (−4·6 to 20·3)	101·7 (84·9 to 122·0)	97·4 (80·5 to 120·9)	−4·2 (−13·1 to 9·0)
		Encephalitis		8850·1 (8064·3 to 9665·8)	8452·5 (7668·6 to 9411·6)	−4·5 (−11·6 to 2·9)	132·1 (120·8 to 144·2)	114·6 (104·0 to 127·6)	−13·2 (−19·7 to −6·8)*
		Diphtheria		443·2 (229·3 to 905·0)	170·8 (83·4 to 388·7)	−61·5 (−86·1 to 3·5)	6·4 (3·3 to 13·1)	2·3 (1·1 to 5·2)	−64·2 (−87·1 to −3·7)*
		Whooping cough		8559·1 (3269·5 to 19234·7)	5070·5 (1815·7 to 10791·8)	−40·8 (−77·3 to 62·2)	123·2 (47·0 to 276·7)	68·0 (24·3 to 144·7)	−44·8 (−78·8 to 51·2)
		Tetanus		7213·2 (6251·3 to 9082·1)	3510·0 (3002·2 to 4502·9)	−51·3 (−58·0 to −43·8)*	105·1 (90·6 to 134·4)	47·1 (40·3 to 60·6)	−55·1 (−61·2 to −48·1)*
		Measles		24 602·4 (9 272·8 to 51 178·0)	6150·1 (2193·1 to 13467·4)	−75·0 (−84·4 to −59·0)*	355·1 (133·9 to 738·2)	82·7 (29·5 to 181·1)	−76·7 (−85·5 to −61·8)*
		Varicella and herpes zoster		616·0 (521·8 to 727·1)	491·2 (386·1 to 625·3)	−20·3 (−33·2 to −4·3)*	9·7 (8·2 to 11·5)	6·8 (5·3 to 8·6)	−29·9 (−40·6 to −16·7)*
	**Neglected tropical diseases and malaria**			**116 583·6 (96 865·6 to 137 551·5)**	**79 212·2 (63 820·2 to 97 299·0)**	−32·1 (−40·2 to −23·7)*	**1710·8 (1422·4 to 2019·1)**	**1066·4 (859·1 to 1310·2)**	−37·7 (−45·1 to −30·1)*
		Malaria		90 438·1 (73 336·5 to 107 546·9)	55 769·6 (42 478·4 to 69 078·5)	−38·3 (−48·1 to −27·8)*	1315·8 (1067·0 to 1562·5)	749·3 (571·0 to 927·9)	−43·1 (−52·0 to −33·4)*
		Chagas disease		243·6 (220·0 to 273·5)	236·1 (211·8 to 265·3)	−3·0 (−8·5 to 3·0)	4·4 (4·0 to 5·0)	3·4 (3·1 to 3·9)	−22·7 (−27·0 to −18·0)*
		Leishmaniasis		1367·1 (878·4 to 1979·8)	1418·9 (1005·8 to 1913·8)	3·8 (−8·6 to 19·3)	20·1 (13·0 to 28·9)	18·9 (13·4 to 25·5)	−5·9 (−16·9 to 8·0)
			Visceral leishmaniasis	1334·4 (844·9 to 1949·5)	1377·4 (965·4 to 1863·8)	3·2 (−9·2 to 19·1)	19·6 (12·4 to 28·5)	18·3 (12·9 to 24·8)	−6·3 (−17·4 to 7·9)
			Cutaneous and mucocutaneous leishmaniasis	32·6 (15·2 to 63·3)	41·5 (19·4 to 81·0)	27·3 (23·6 to 30·6)*	0·5 (0·2 to 1·0)	0·6 (0·3 to 1·1)	11·4 (8·2 to 14·5)*
		African trypanosomiasis		803·1 (425·6 to 1311·4)	202·4 (104·6 to 322·3)	−74·8 (−80·4 to −68·2)*	11·8 (6·3 to 19·3)	2·7 (1·4 to 4·3)	−77·2 (−82·3 to −71·3)*
		Schistosomiasis		3417·7 (1883·7 to 6044·0)	2613·3 (1409·9 to 4695·8)	−23·5 (−30·6 to −6·7)*	51·1 (28·4 to 90·0)	35·1 (19·0 to 62·9)	−31·3 (−37·7 to −16·2)*
		Cysticercosis		369·6 (260·6 to 489·8)	303·6 (211·4 to 410·9)	−17·9 (−22·7 to −13·5)*	5·9 (4·2 to 7·8)	4·1 (2·9 to 5·5)	−30·4 (−34·3 to −26·5)*
		Cystic echinococcosis		174·1 (141·3 to 212·4)	172·6 (132·1 to 220·6)	−0·8 (−7·4 to 4·5)	2·8 (2·2 to 3·4)	2·4 (1·8 to 3·0)	−14·7 (−20·2 to −10·2)*
		Lymphatic filariasis		2476·6 (1272·2 to 4008·8)	2075·0 (1120·5 to 3311·5)	−16·2 (−32·1 to −3·8)*	38·9 (20·0 to 63·1)	28·1 (15·2 to 44·9)	−27·7 (−41·5 to −17·0)*
		Onchocerciasis		1441·6 (835·2 to 2288·7)	1135·7 (545·8 to 2005·7)	−21·2 (−38·6 to −4·8)*	22·5 (13·2 to 35·5)	15·5 (7·5 to 27·3)	−31·2 (−46·8 to −16·0)*
		Trachoma		282·6 (194·3 to 396·1)	279·2 (192·5 to 396·2)	−1·2 (−5·6 to 3·0)	5·5 (3·8 to 7·7)	4·2 (2·9 to 5·9)	−23·9 (−27·6 to −20·3)*
		Dengue		1132·8 (795·9 to 1616·2)	1892·2 (1266·7 to 2925·2)	67·0 (16·8 to 166·1)*	16·6 (11·7 to 23·8)	25·5 (17·1 to 39·5)	53·5 (7·3 to 144·1)*
		Yellow fever		400·8 (83·3 to 1083·2)	329·8 (66·9 to 898·1)	−17·7 (−34·1 to 1·7)	5·8 (1·2 to 15·6)	4·4 (0·9 to 12·1)	−23·0 (−38·2 to −4·9)*
		Rabies		1760·6 (1525·6 to 2019·8)	931·6 (779·2 to 1120·9)	−47·1 (−54·2 to −39·3)*	26·4 (23·0 to 30·2)	12·6 (10·6 to 15·2)	−52·1 (−58·6 to −45·2)*
		Intestinal nematode infections		4404·5 (2719·6 to 6783·4)	3378·3 (2046·2 to 5294·5)	−23·3 (−27·4 to −18·2)*	65·8 (40·6 to 101·4)	45·6 (27·6 to 71·4)	−30·7 (−34·5 to −26·1)*
		Ascariasis		1693·0 (1061·5 to 2610·7)	1075·4 (685·1 to 1660·9)	−36·5 (−43·1 to −29·5)*	25·2 (15·8 to 39·0)	14·5 (9·2 to 22·4)	−42·5 (−48·6 to −36·2)*
		Trichuriasis		653·1 (357·9 to 1076·3)	544·1 (289·3 to 946·1)	−16·7 (−30·6 to 3·7)	9·8 (5·4 to 16·1)	7·3 (3·9 to 12·8)	−24·9 (−37·5 to −6·7)*
		Hookworm disease		2058·4 (1275·6 to 3171·3)	1758·8 (1085·8 to 2755·1)	−14·6 (−19·9 to −9·4)*	30·8 (19·0 to 47·4)	23·7 (14·7 to 37·1)	−22·9 (−27·7 to −18·1)*
		Food-borne trematodiases		1625·6 (793·1 to 3058·3)	1686·5 (855·0 to 3072·8)	3·7 (−0·5 to 10·1)	25·1 (12·3 to 47·0)	22·6 (11·5 to 41·0)	−10·0 (−13·5 to −5·2)*
		Leprosy		30·8 (20·6 to 43·0)	31·0 (20·8 to 43·6)	0·6 (−1·5 to 2·9)	0·5 (0·4 to 0·8)	0·4 (0·3 to 0·6)	−19·2 (−20·8 to −17·4)*
		Ebola virus disease		0·8 (0·7 to 1·0)	295·4 (238·3 to 353·8)	36 024·8 (36 010·3 to 36 054·6)*	0·0 (0·0 to 0·0)	3·9 (3·2 to 4·7)	31 654·0 (31 640·5 to 31 681·3)*
		Other neglected tropical diseases		6213·6 (3655·0 to 11309·8)	6461·0 (3516·3 to 13769·5)	4·0 (−18·2 to 40·2)	91·8 (53·8 to 167·4)	87·6 (47·7 to 187·2)	−4·6 (−24·9 to 28·6)
	**Maternal disorders**			**20 797·1 (19 462·4 to 22 290·3)**	**16 282·0 (14 542·5 to 18 451·2)**	−21·7 (−30·1 to −11·7)*	**299·0 (279·8 to 320·7)**	**212·6 (189·9 to 240·9)**	−28·9 (−36·5 to −19·8)*
		Maternal haemorrhage		5614·4 (4 902·4 to 6 402·8)	4645·8 (3 769·9 to 5 636·8)	−17·3 (−28·9 to −4·6)*	81·1 (70·8 to 92·3)	60·7 (49·3 to 73·6)	−25·1 (−35·6 to −13·5)*
		Maternal sepsis and other maternal infections		1443·1 (1195·4 to 1742·2)	1050·0 (792·7 to 1382·8)	−27·2 (−41·1 to −10·8)*	20·8 (17·3 to 25·1)	13·7 (10·4 to 18·0)	−34·1 (−46·8 to −19·0)*
		Maternal hypertensive disorders		3939·7 (3391·5 to 4579·2)	2938·0 (2318·0 to 3698·5)	−25·4 (−35·9 to −13·2)*	56·1 (48·3 to 65·2)	38·3 (30·2 to 48·2)	−31·7 (−41·2 to −20·7)*
		Maternal obstructed labour and uterine rupture		1905·3 (1592·5 to 2242·8)	1627·1 (1257·6 to 2025·1)	−14·6 (−25·4 to −2·4)*	27·7 (23·2 to 32·5)	21·2 (16·4 to 26·4)	−23·2 (−32·9 to −12·2)*
		Maternal abortion miscarriage and ectopic pregnancy		2366·6 (1992·0 to 2830·9)	1809·7 (1416·9 to 2283·6)	−23·5 (−34·2 to −12·1)*	34·0 (28·7 to 40·8)	23·6 (18·5 to 29·8)	−30·5 (−40·1 to −20·1)*
		Indirect maternal deaths		2192·6 (1803·5 to 2621·7)	1744·2 (1299·7 to 2286·8)	−20·4 (−33·2 to −3·4)*	31·4 (25·9 to 37·5)	22·7 (17·0 to 29·8)	−27·6 (−39·0 to −11·9)*
		Late maternal deaths		449·8 (295·7 to 652·2)	377·7 (244·8 to 562·2)	−16·0 (−26·8 to −2·6)*	6·5 (4·2 to 9·4)	4·9 (3·2 to 7·3)	−23·6 (−33·7 to −11·4)*
		Maternal deaths aggravated by HIV/AIDS		149·9 (96·3 to 204·8)	122·1 (73·3 to 176·5)	−18·6 (−35·2 to 4·2)	2·2 (1·4 to 3·0)	1·6 (1·0 to 2·3)	−27·1 (−42·1 to −6·8)*
		Other maternal disorders		2735·8 (2324·0 to 3245·0)	1967·5 (1578·3 to 2434·9)	−28·1 (−36·5 to −18·1)*	39·3 (33·4 to 46·6)	25·7 (20·6 to 31·7)	−34·7 (−42·4 to −26·1)*
	**Neonatal disorders**			**239 098·4 (232 872·7 to 246 048·9)**	**197 924·8 (191 388·9 to 204 751·8)**	−17·2 (−19·2 to −15·4)*	**3370·5 (3282·2 to 3468·1)**	**2638·3 (2551·0 to 2 729·5)**	−21·7 (−23·6 to −20·0)*
		Neonatal preterm birth complications		99 050·0 (92 118·6 to 110 250·8)	74 833·6 (68 500·3 to 83 015·4)	−24·4 (−29·6 to −19·2)*	1396·6 (1298·8 to 1554·2)	997·5 (913·1 to 1106·5)	−28·6 (−33·4 to −23·6)*
		Neonatal encephalopathy due to birth asphyxia and trauma		79 423·6 (72 215·4 to 87 185·6)	67 856·5 (61 497·7 to 75 824·7)	−14·6 (−21·7 to −6·7)*	1118·8 (1017·3 to 1228·6)	904·4 (819·6 to 1010·5)	−19·2 (−26·0 to −11·7)*
		Neonatal sepsis and other neonatal infections		30 510·1 (21 830·7 to 40 329·6)	30 454·5 (21 591·8 to 39 747·9)	−0·2 (−16·2 to 20·3)	429·6 (307·4 to 567·7)	406·0 (287·8 to 529·8)	−5·5 (−20·6 to 13·9)
		Haemolytic disease and other neonatal jaundice		6427·0 (4234·6 to 9708·3)	4504·0 (3202·7 to 6420·6)	−29·9 (−44·1 to −13·6)*	90·9 (60·0 to 137·0)	60·1 (42·7 to 85·6)	−33·9 (−47·2 to −18·6)*
		Other neonatal disorders		23 687·6 (17 420·2 to 30 795·3)	20 276·2 (15 531·0 to 25 385·9)	−14·4 (−32·8 to 7·1)	334·6 (246·3 to 434·7)	270·4 (207·1 to 338·5)	−19·2 (−36·6 to 1·1)
	**Nutritional deficiencies**			**84 133·3 (65 215·4 to 108 480·6)**	**76 517·1 (58 781·9 to 100 939·6)**	−9·1 (−15·2 to −3·5)*	**1242·7 (964·9 to 1598·6)**	**1034·4 (795·9 to 1 363·6)**	−16·8 (−22·4 to −11·7)*
		Protein-energy malnutrition		26 655·2 (21 449·8 to 32 723·9)	21 094·0 (16 844·2 to 26 299·5)	−20·9 (−34·5 to −4·8)*	395·1 (319·4 to 484·0)	286·5 (228·9 to 356·6)	−27·5 (−39·8 to −13·0)*
		Iodine deficiency		2320·5 (1500·7 to 3422·1)	2476·4 (1592·8 to 3662·1)	6·7 (3·4 to 9·9)*	35·3 (22·8 to 52·1)	33·4 (21·5 to 49·4)	−5·2 (−8·4 to −2·4)*
		Vitamin A deficiency		209·5 (130·8 to 309·2)	232·4 (143·4 to 346·3)	10·9 (7·5 to 14·6)*	3·1 (2·0 to 4·6)	3·1 (1·9 to 4·7)	0·2 (−2·7 to 3·3)
		Iron-deficiency anaemia		52 951·3 (36 342·0 to 74 873·5)	51 217·1 (35 014·4 to 72 661·0)	−3·3 (−4·8 to −1·8)*	778·6 (534·8 to 1 099·4)	690·8 (472·4 to 979·6)	−11·3 (−12·7 to −10·0)*
		Other nutritional deficiencies		1996·8 (1378·4 to 2848·4)	1497·2 (1135·7 to 1983·7)	−25·0 (−43·3 to −9·4)*	30·5 (21·2 to 43·5)	20·5 (15·7 to 27·1)	−32·7 (−48·9 to −19·0)*
	**Other communicable, maternal, neonatal, and nutritional diseases**			**25 182·6 (20 002·2 to 31 397·3)**	**21 792·2 (17 474·0 to 26 922·3)**	−13·5 (−18·6 to −7·8)*	**373·1 (298·4 to 462·5)**	**293·8 (235·7 to 362·6)**	−21·2 (−25·9 to −16·1)*
		Sexually transmitted diseases excluding HIV		12 378·7 (7 831·6 to 18 396·6)	10 330·9 (6 583·0 to 15 315·1)	−16·5 (−24·2 to −8·6)*	178·7 (113·6 to 264·7)	138·1 (87·9 to 205·0)	−22·7 (−29·6 to −15·5)*
			Syphilis	11 190·5 (6 607·1 to 17 280·8)	8957·1 (5273·2 to 13969·7)	−20·0 (−28·4 to −11·8)*	160·9 (95·4 to 247·9)	120·1 (70·8 to 187·2)	−25·4 (−33·3 to −17·7)*
			Chlamydial infection	337·0 (194·2 to 537·7)	369·8 (214·0 to 595·4)	9·7 (6·5 to 13·0)*	4·9 (2·8 to 7·8)	4·8 (2·8 to 7·7)	−2·3 (−5·0 to 0·5)
			Gonococcal infection	382·8 (238·6 to 573·0)	469·8 (283·4 to 716·8)	22·7 (15·9 to 28·1)*	5·6 (3·5 to 8·4)	6·1 (3·7 to 9·3)	8·8 (2·8 to 13·8)*
			Trichomoniasis	167·4 (67·1 to 354·8)	194·3 (77·8 to 412·1)	16·1 (15·0 to 17·2)*	2·5 (1·0 to 5·4)	2·6 (1·0 to 5·4)	1·0 (0·3 to 1·8)*
			Genital herpes	197·8 (61·5 to 468·3)	236·4 (74·3 to 555·8)	19·5 (17·4 to 23·0)*	3·2 (1·0 to 7·6)	3·2 (1·0 to 7·5)	0·7 (−1·5 to 5·2)
			Other sexually transmitted diseases	103·2 (74·9 to 139·2)	103·5 (74·3 to 141·2)	0·3 (−3·1 to 3·2)	1·6 (1·1 to 2·1)	1·4 (1·0 to 1·9)	−13·2 (−16·1 to −10·6)*
		Hepatitis		6042·4 (5738·7 to 6344·8)	4925·3 (4634·8 to 5229·3)	−18·5 (−22·7 to −14·1)*	93·0 (88·6 to 97·5)	66·7 (62·8 to 70·8)	−28·3 (−31·9 to −24·6)*
			Acute hepatitis A	1486·6 (1058·5 to 1896·9)	1030·3 (714·9 to 1335·9)	−30·7 (−39·7 to −20·8)*	21·5 (15·2 to 27·5)	13·8 (9·6 to 17·9)	−35·6 (−43·9 to −26·4)*
			Hepatitis B	2693·5 (2298·5 to 3080·0)	2449·1 (2083·5 to 2799·2)	−9·1 (−15·4 to −2·0)*	44·0 (37·8 to 50·0)	33·4 (28·5 to 38·1)	−24·1 (−29·2 to −18·6)*
			Hepatitis C	98·4 (26·2 to 220·4)	89·1 (25·5 to 201·9)	−9·5 (−23·3 to 7·6)	1·6 (0·4 to 3·7)	1·2 (0·3 to 2·8)	−25·9 (−37·5 to −11·0)*
			Acute hepatitis E	1763·9 (1304·1 to 2265·3)	1356·8 (972·0 to 1794·4)	−23·1 (−31·6 to −14·0)*	25·9 (19·0 to 33·7)	18·3 (13·1 to 24·2)	−29·6 (−37·1 to −21·6)*
		Other infectious diseases		6761·5 (4784·8 to 8106·7)	6536·0 (4527·7 to 7935·6)	−3·3 (−13·9 to 9·2)	101·3 (72·0 to 120·9)	89·0 (61·6 to 107·8)	−12·2 (−21·6 to −1·0)*
**Non-communicable diseases**				**1 322 207·9 (1 181 179·7 to 1 471 448·8)**	**1 473 508·2 (1 309 803·2 to 1 650 147·4)**	11·4 (9·7 to 13·0)*	**23 220·2 (20 898·5 to 25 689·0)**	**21 062·4 (18 790·6 to 23 518·0)**	−9·3 (−10·9 to −7·9)*
	**Neoplasms**			**187 562·1 (183 699·7 to 191 539·2)**	**209 359·2 (204 155·5 to 214 470·1)**	11·6 (9·3 to 14·2)*	**3424·3 (3356·2 to 3494·4)**	**3024·4 (2949·8 to 3098·9)**	−11·7 (−13·4 to −9·7)*
		Lip and oral cavity cancer		2954·1 (2866·9 to 3050·0)	3780·1 (3645·7 to 3928·9)	28·0 (23·9 to 32·6)*	53·7 (52·1 to 55·4)	53·7 (51·8 to 55·8)	0·1 (−3·1 to 3·7)
		Nasopharynx cancer		1805·8 (1477·1 to 1912·4)	1911·7 (1562·1 to 2040·2)	5·9 (−2·2 to 12·4)	30·9 (25·4 to 32·7)	26·5 (21·6 to 28·2)	−14·3 (−20·6 to −9·0)*
		Other pharynx cancer		1419·9 (1374·7 to 1468·8)	1715·7 (1635·4 to 1795·0)	20·8 (15·2 to 26·3)*	25·9 (25·1 to 26·8)	24·3 (23·2 to 25·4)	−6·3 (−10·7 to −2·1)*
		Oesophageal cancer		10 665·4 (10 313·5 to 11 043·9)	9 854·4 (9 465·3 to 10 270·8)	−7·6 (−12·4 to −2·1)*	201·0 (194·5 to 207·8)	143·6 (137·9 to 149·5)	−28·6 (−32·2 to −24·4)*
		Stomach cancer		18 665·9 (18 186·3 to 19 175·5)	17 439·6 (16 876·7 to 18 034·6)	−6·6 (−9·7 to −3·3)*	350·6 (341·9 to 360·0)	255·9 (247·9 to 264·5)	−27·0 (−29·4 to −24·5)*
		Colon and rectum cancer		14 409·5 (14 108·0 to 14 779·0)	17 026·6 (16 586·7 to 17 504·7)	18·2 (15·7 to 20·9)*	274·0 (268·2 to 280·8)	251·3 (244·9 to 258·4)	−8·3 (−10·2 to −6·2)*
		Liver cancer		19 643·6 (16 871·4 to 20 641·2)	20 578·0 (18 937·8 to 21 915·3)	4·8 (−1·4 to 15·5)	350·9 (302·8 to 368·5)	292·1 (269·1 to 311·0)	−16·8 (−21·4 to −8·7)*
			Liver cancer due to hepatitis B	8420·5 (7092·2 to 9020·2)	8029·5 (7279·6 to 8795·5)	−4·6 (−11·2 to 8·2)	143·4 (121·3 to 153·7)	110·6 (100·4 to 121·2)	−22·9 (−27·9 to −12·9)*
			Liver cancer due to hepatitis C	2897·4 (2607·7 to 3109·1)	3324·0 (3012·2 to 3573·6)	14·7 (10·3 to 20·2)*	55·5 (50·1 to 59·5)	49·3 (44·9 to 52·9)	−11·2 (−14·4 to −7·1)*
			Liver cancer due to alcohol use	4786·9 (4075·0 to 5168·7)	5888·5 (5368·4 to 6441·0)	23·0 (14·8 to 36·1)*	89·2 (76·2 to 96·3)	84·8 (77·5 to 92·7)	−4·9 (−11·1 to 4·8)
			Liver cancer due to other causes	3538·8 (3022·8 to 3829·6)	3336·0 (3019·1 to 3637·7)	−5·7 (−11·2 to 3·8)	62·9 (54·1 to 68·1)	47·4 (42·9 to 51·6)	−24·6 (−28·9 to −17·5)*
		Gallbladder and biliary tract cancer		2448·7 (2359·2 to 2524·3)	2615·7 (2454·6 to 2740·8)	6·8 (2·2 to 11·6)*	47·3 (45·7 to 48·7)	39·0 (36·7 to 40·9)	−17·5 (−21·0 to −13·8)*
		Pancreatic cancer		6528·1 (6433·9 to 6630·7)	8236·6 (8064·7 to 8420·0)	26·2 (23·3 to 29·0)*	125·2 (123·4 to 127·0)	121·8 (119·3 to 124·4)	−2·7 (−4·8 to −0·6)*
		Larynx cancer		2364·1 (2290·8 to 2443·5)	2608·5 (2518·3 to 2705·0)	10·3 (7·1 to 13·9)*	44·0 (42·7 to 45·5)	37·6 (36·3 to 39·0)	−14·5 (−17·0 to −11·8)*
		Tracheal, bronchus, and lung cancer		31 802·0 (31 117·2 to 32 516·2)	36 419·5 (35 356·6 to 37 615·6)	14·5 (11·0 to 19·1)*	604·5 (591·8 to 617·4)	535·9 (520·3 to 553·1)	−11·3 (−14·0 to −7·9)*
		Malignant skin melanoma		1304·0 (1086·8 to 1655·2)	1596·3 (1293·4 to 1982·7)	22·4 (15·6 to 27·5)*	23·3 (19·4 to 29·6)	22·8 (18·4 to 28·3)	−2·3 (−7·7 to 1·7)
		Non-melanoma skin cancer		794·3 (764·9 to 828·0)	1088·8 (1028·3 to 1150·1)	37·1 (32·0 to 41·9)*	15·2 (14·7 to 15·9)	16·3 (15·4 to 17·2)	6·8 (2·8 to 10·6)*
			Non-melanoma skin cancer (squamous-cell carcinoma)	789·6 (761·2 to 822·3)	1082·9 (1025·0 to 1142·0)	37·1 (32·0 to 42·0)*	15·1 (14·6 to 15·8)	16·2 (15·3 to 17·1)	6·9 (2·9 to 10·6)*
			Non-melanoma skin cancer (basal-cell carcinoma)	4·7 (2·2 to 8·7)	6·0 (2·8 to 11·1)	26·9 (23·4 to 30·2)*	0·1 (0·0 to 0·2)	0·1 (0·0 to 0·2)	−3·8 (−6·5 to −1·2)*
		Breast cancer		12 939·2 (12 181·7 to 13 828·7)	15 410·6 (14 415·5 to 16 217·8)	19·1 (11·6 to 25·8)*	231·4 (218·3 to 246·9)	217·1 (203·2 to 228·7)	−6·2 (−11·9 to −1·1)*
		Cervical cancer		6819·2 (6393·4 to 7236·4)	6963·0 (6526·2 to 7408·0)	2·1 (−4·5 to 10·5)	118·9 (111·8 to 126·1)	96·7 (90·7 to 102·7)	−18·7 (−23·9 to −12·2)*
		Uterine cancer		2077·7 (1963·6 to 2201·8)	2230·7 (2098·3 to 2386·9)	7·4 (0·3 to 15·8)*	38·7 (36·6 to 40·9)	32·2 (30·3 to 34·5)	−16·6 (−22·0 to −10·1)*
		Ovarian cancer		3496·3 (3401·0 to 3660·5)	4135·9 (3992·4 to 4298·0)	18·3 (13·4 to 23·1)*	63·2 (61·5 to 66·0)	58·6 (56·6 to 60·8)	−7·3 (−11·0 to −3·8)*
		Prostate cancer		4793·4 (3992·1 to 6079·2)	6281·2 (5233·9 to 7904·2)	31·0 (26·9 to 35·2)*	100·0 (83·5 to 126·8)	99·6 (83·0 to 125·7)	−0·4 (−3·5 to 2·6)
		Testicular cancer		411·3 (390·2 to 435·8)	442·6 (413·1 to 473·3)	7·6 (0·3 to 13·6)*	6·3 (6·0 to 6·7)	5·9 (5·5 to 6·3)	−6·4 (−12·6 to −1·2)*
		Kidney cancer		2649·3 (2539·2 to 2779·3)	3340·3 (3189·6 to 3513·2)	26·1 (21·2 to 30·6)*	48·5 (46·8 to 50·6)	48·4 (46·2 to 50·8)	−0·3 (−3·8 to 3·0)
		Bladder cancer		2832·7 (2754·6 to 2908·1)	3368·8 (3251·3 to 3496·5)	18·9 (15·4 to 22·4)*	56·3 (54·8 to 57·8)	51·3 (49·5 to 53·2)	−8·8 (−11·5 to −6·2)*
		Brain and nervous system cancer		6738·3 (6079·6 to 7270·6)	7624·4 (6975·3 to 8218·8)	13·2 (5·0 to 21·0)*	111·4 (100·8 to 119·7)	105·6 (96·7 to 113·7)	−5·2 (−11·7 to 1·2)
		Thyroid cancer		649·6 (605·3 to 707·6)	846·3 (754·1 to 929·0)	30·3 (19·2 to 37·4)*	12·1 (11·3 to 13·2)	12·4 (11·1 to 13·6)	2·2 (−6·2 to 7·8)
		Mesothelioma		544·9 (529·2 to 560·6)	701·9 (678·8 to 722·2)	28·8 (24·3 to 33·3)*	10·1 (9·8 to 10·3)	10·3 (9·9 to 10·5)	2·0 (−1·5 to 5·4)
		Hodgkin's lymphoma		949·3 (837·3 to 1120·9)	845·6 (758·4 to 1031·7)	−10·9 (−14·9 to −6·7)*	15·5 (13·7 to 18·3)	11·6 (10·5 to 14·2)	−24·7 (−28·2 to −21·1)*
		Non-Hodgkin lymphoma		5063·1 (4581·8 to 5581·3)	6283·8 (5449·8 to 6632·3)	24·1 (11·5 to 31·7)*	88·3 (79·7 to 96·6)	89·5 (77·5 to 94·3)	1·3 (−8·5 to 7·0)
		Multiple myeloma		1694·3 (1646·6 to 1751·4)	2182·4 (2096·5 to 2255·8)	28·8 (23·8 to 33·2)*	32·2 (31·2 to 33·2)	32·1 (30·8 to 33·1)	−0·4 (−4·1 to 2·9)
		Leukaemia		11 272·2 (10 956·3 to 11 683·7)	12 040·4 (11 609·8 to 12 499·3)	6·8 (2·9 to 10·5)*	181·6 (176·6 to 187·8)	167·7 (161·8 to 173·9)	−7·6 (−10·8 to −4·6)*
			Acute lymphoid leukaemia	4857·0 (4505·3 to 5452·8)	5057·8 (4658·4 to 5507·7)	4·1 (−1·7 to 9·9)	73·6 (68·3 to 82·4)	69·1 (63·6 to 75·2)	−6·1 (−11·3 to −1·0)*
			Chronic lymphoid leukaemia	1183·2 (1107·9 to 1256·6)	1268·6 (1194·2 to 1349·3)	7·2 (1·8 to 12·8)*	21·8 (20·5 to 23·1)	18·7 (17·7 to 19·9)	−14·1 (−18·3 to −10·0)*
			Acute myeloid leukaemia	4199·7 (3770·1 to 4553·5)	4769·0 (4345·7 to 5193·7)	13·6 (8·2 to 18·5)*	68·5 (62·1 to 74·0)	66·6 (60·8 to 72·5)	−2·8 (−7·0 to 1·1)
			Chronic myeloid leukaemia	1032·3 (957·8 to 1131·8)	945·1 (885·8 to 1032·9)	−8·5 (−12·3 to −3·8)*	17·7 (16·5 to 19·3)	13·3 (12·5 to 14·5)	−24·8 (−27·8 to −21·2)*
		Other neoplasms		9825·9 (9023·1 to 10 351·7)	11 789·9 (10 528·0 to 12 616·2)	20·0 (13·4 to 25·7)*	163·3 (150·1 to 171·6)	164·6 (146·8 to 175·8)	0·8 (−4·5 to 5·5)
	**Cardiovascular diseases**			**326 252·4 (318 365·8 to 334 285·8)**	**347 528·9 (337 220·3 to 358 093·5)**	6·5 (4·1 to 8·7)*	**6231·9 (6086·8 to 6378·1)**	**5179·7 (5026·3 to 5334·2)**	−16·9 (−18·7 to −15·3)*
		Rheumatic heart disease		11 594·7 (10 668·9 to 12 707·4)	10 513·2 (9 611·0 to 11 514·5)	−9·3 (−13·3 to −5·7)*	193·6 (178·4 to 210·6)	146·6 (134·2 to 160·1)	−24·3 (−27·5 to −21·3)*
		Ischaemic heart disease		147 780·0 (144 845·9 to 151 651·5)	164 020·4 (159 621·3 to 169 088·2)	11·0 (8·3 to 13·3)*	2860·0 (2805·1 to 2927·3)	2452·6 (2388·9 to 2526·8)	−14·2 (−16·2 to −12·5)*
		Cerebrovascular disease		118 566·2 (115 776·1 to 121 419·2)	118 626·7 (114 862·4 to 122 627·0)	0·1 (−2·5 to 2·7)	2283·2 (2230·3 to 2335·8)	1776·6 (1721·1 to 1834·8)	−22·2 (−24·1 to −20·1)*
			Ischaemic stroke	44 104·6 (42 363·3 to 45 822·3)	45 208·5 (43 150·2 to 47 386·8)	2·5 (−0·4 to 5·2)	903·6 (869·6 to 936·9)	706·2 (675·6 to 739·0)	−21·8 (−24·0 to −19·8)*
		Haemorrhagic stroke		74 461·5 (72 176·9 to 76 874·3)	73 418·2 (70 737·2 to 76 596·4)	−1·4 (−4·6 to 2·1)	1379·6 (1337·6 to 1423·6)	1070·4 (1032·1 to 1116·3)	−22·4 (−25·0 to −19·6)*
		Hypertensive heart disease		14 852·4 (13 919·4 to 16 052·6)	17 484·6 (16 286·9 to 18 593·6)	17·7 (11·6 to 22·9)*	286·5 (268·8 to 309·8)	262·2 (243·2 to 279·1)	−8·5 (−13·4 to −4·5)*
		Cardiomyopathy and myocarditis		9619·8 (9134·6 to 9993·7)	9220·3 (8765·6 to 9706·0)	−4·2 (−8·3 to −0·1)*	163·4 (155·8 to 169·5)	130·0 (123·7 to 136·7)	−20·5 (−23·7 to −17·2)*
		Atrial fibrillation and flutter		3463·2 (2764·5 to 4281·3)	4433·7 (3541·7 to 5489·1)	28·0 (26·6 to 29·3)*	73·8 (59·3 to 90·5)	70·6 (56·5 to 86·9)	−4·2 (−5·3 to −3·3)*
		Aortic aneurysm		2468·0 (2403·2 to 2547·3)	2930·2 (2846·0 to 3007·0)	18·7 (12·9 to 22·6)*	47·6 (46·4 to 49·1)	43·8 (42·6 to 45·0)	−7·9 (−12·3 to −5·0)*
		Peripheral vascular disease		913·0 (686·4 to 1279·3)	1198·8 (898·0 to 1682·6)	31·3 (27·7 to 35·2)*	19·3 (14·5 to 26·9)	19·1 (14·3 to 26·8)	−1·1 (−3·9 to 1·7)
		Endocarditis		1962·3 (1616·0 to 2190·5)	2210·3 (1838·9 to 2421·3)	12·6 (6·3 to 18·8)*	33·0 (27·7 to 36·7)	31·2 (26·1 to 34·2)	−5·5 (−10·2 to −0·9)*
		Other cardiovascular and circulatory diseases		15 032·7 (13 427·8 to 16 820·7)	16 890·7 (14 824·7 to 19 104·2)	12·4 (8·4 to 15·6)*	271·6 (243·4 to 303·7)	247·0 (217·2 to 279·2)	−9·0 (−12·1 to −6·5)*
	**Chronic respiratory diseases**			**97 356·3 (90 607·8 to 104 559·9)**	**97 451·8 (89 829·7 to 105 527·6)**	**0·1 (−2·4 to 2·6)**	**1802·0 (1694·4 to 1915·3)**	**1443·4 (1336·9 to 1556·8)**	−19·9 (−22·2 to −17·8)*
		Chronic obstructive pulmonary disease		63 773·8 (60 981·4 to 66 186·6)	63 850·4 (61 215·3 to 66 288·6)	0·1 (−3·1 to 3·8)	1246·9 (1195·2 to 1291·9)	971·1 (932·7 to 1 007·6)	−22·1 (−24·6 to −19·2)*
		Pneumoconiosis		976·7 (816·2 to 1 168·6)	1 099·6 (902·7 to 1 314·7)	12·6 (6·9 to 18·9)*	17·8 (15·0 to 21·1)	16·0 (13·2 to 19·1)	−9·9 (−14·8 to −4·7)*
			Silicosis	271·5 (240·1 to 308·1)	270·2 (235·8 to 310·1)	−0·5 (−10·1 to 11·5)	5·0 (4·5 to 5·7)	4·0 (3·5 to 4·5)	−21·3 (−28·7 to −12·0)*
			Asbestosis	76·3 (63·0 to 87·9)	92·0 (75·3 to 107·2)	20·5 (13·7 to 27·8)*	1·4 (1·2 to 1·6)	1·4 (1·1 to 1·6)	−3·8 (−8·9 to 1·7)
			Coal worker's pneumoconiosis	59·7 (52·8 to 67·4)	57·5 (49·0 to 67·2)	−3·6 (−17·4 to 9·4)	1·2 (1·0 to 1·3)	0·9 (0·7 to 1·0)	−25·1 (−35·6 to −15·1)*
			Other pneumoconiosis	569·3 (440·2 to 715·7)	680·0 (526·2 to 855·9)	19·5 (13·4 to 25·9)*	10·2 (8·0 to 12·7)	9·8 (7·6 to 12·3)	−3·4 (−9·0 to 2·3)
		Asthma		26 859·7 (21 268·1 to 33 122·5)	26 168·8 (20 501·4 to 32 583·0)	−2·6 (−9·2 to 4·2)	439·8 (351·4 to 537·5)	365·6 (287·5 to 453·5)	−16·9 (−23·5 to −10·3)*
		Interstitial lung disease and pulmonary sarcoidosis		1672·3 (1351·2 to 1954·1)	2352·6 (1933·8 to 2618·7)	40·7 (29·2 to 48·8)*	32·0 (25·7 to 37·2)	35·4 (28·9 to 39·3)	10·3 (1·5 to 16·7)*
		Other chronic respiratory diseases		4073·7 (3295·2 to 4805·9)	3980·4 (3225·6 to 4569·7)	−2·3 (−11·0 to 7·3)	65·5 (53·3 to 76·6)	55·4 (44·9 to 63·5)	−15·3 (−22·4 to −7·5)*
		**Cirrhosis and other chronic liver diseases**		**37 101·5 (35 722·1 to 39 385·8)**	**38 973·3 (37 202·0 to 41 702·9)**	5·0 (1·6 to 8·7)*	**632·5 (609·4 to 669·2)**	**539·6 (515·4 to 576·8)**	−14·7 (−17·4 to −11·8)*
		Cirrhosis and other chronic liver diseases due to hepatitis B		10 297·6 (9 495·9 to 11 233·5)	10 754·7 (9 876·8 to 11 957·6)	4·4 (0·2 to 8·6)*	178·0 (164·1 to 194·8)	149·5 (137·3 to 165·9)	−16·0 (−19·2 to −12·7)*
		Cirrhosis and other chronic liver diseases due to hepatitis C		8408·4 (7768·6 to 9069·5)	9161·7 (8443·3 to 9968·2)	9·0 (5·7 to 12·3)*	146·5 (135·6 to 157·9)	127·4 (117·6 to 138·4)	−13·0 (−15·6 to −10·4)*
		Cirrhosis and other chronic liver diseases due to alcohol use		10 093·5 (9 424·0 to 10 841·4)	10 997·4 (10 197·4 to 11 875·1)	9·0 (5·0 to 13·7)*	171·9 (160·5 to 184·3)	150·7 (139·7 to 162·6)	−12·3 (−15·5 to −8·6)*
		Cirrhosis and other chronic liver diseases due to other causes		8302·1 (7741·5 to 9165·9)	8059·5 (7452·2 to 8977·7)	−2·9 (−6·5 to 1·3)	136·1 (126·8 to 149·9)	112·0 (103·6 to 124·7)	−17·7 (−20·6 to −14·3)*
	**Digestive diseases**			**41 351·8 (37 701·8 to 45 778·3)**	**42 189·1 (38 101·1 to 47 087·5)**	**2·0 (−2·7 to 6·2)**	**716·3 (653·0 to 793·4)**	**600·3 (542·1 to 670·1)**	−16·2 (−19·7 to −12·9)*
		Peptic ulcer disease		10 164·0 (9220·6 to 11 279·2)	8894·4 (7950·0 to 10 039·2)	−12·5 (−17·7 to −5·9)*	180·8 (163·9 to 201·3)	128·5 (114·6 to 145·3)	−28·9 (−33·2 to −23·6)*
		Gastritis and duodenitis		5757·0 (4331·9 to 7638·9)	6145·5 (4517·3 to 8251·9)	6·7 (2·5 to 11·1)*	100·9 (76·0 to 134·0)	87·8 (64·6 to 118·0)	−13·1 (−16·4 to −9·7)*
		Appendicitis		2197·3 (1823·7 to 2661·6)	2015·9 (1631·6 to 2381·2)	−8·3 (−21·5 to 6·4)	34·4 (28·7 to 41·2)	27·6 (22·4 to 32·6)	−19·6 (−30·7 to −6·8)*
		Paralytic ileus and intestinal obstruction		6957·0 (6123·4 to 8236·1)	7135·5 (6464·3 to 8502·6)	2·6 (−5·5 to 11·2)	117·3 (104·1 to 138·5)	100·7 (91·4 to 119·7)	−14·2 (−20·1 to −7·5)*
		Inguinal femoral and abdominal hernia		1689·6 (1147·1 to 1996·2)	1645·7 (1089·7 to 1969·1)	−2·6 (−9·8 to 7·0)	29·2 (20·1 to 34·2)	23·4 (15·6 to 27·9)	−19·7 (−26·1 to −11·8)*
		Inflammatory bowel disease		3222·5 (2514·5 to 4041·9)	3588·0 (2797·3 to 4483·0)	11·3 (5·1 to 15·4)*	53·8 (42·3 to 67·1)	49·8 (39·0 to 62·0)	−7·5 (−12·2 to −4·3)*
		Vascular intestinal disorders		1475·6 (1358·6 to 1619·3)	1741·6 (1607·3 to 1903·4)	18·0 (12·8 to 23·7)*	28·8 (26·6 to 31·5)	26·5 (24·5 to 28·8)	−8·2 (−12·2 to −3·9)*
		Gallbladder and biliary diseases		2706·6 (2459·3 to 2994·3)	2878·7 (2597·4 to 3161·8)	6·4 (−0·2 to 11·9)	48·3 (44·1 to 53·0)	41·6 (37·6 to 45·6)	−13·8 (−18·7 to −9·6)*
		Pancreatitis		3765·2 (3501·1 to 4071·3)	4373·2 (4000·1 to 4766·0)	16·1 (9·2 to 23·2)*	62·8 (58·5 to 67·8)	60·2 (55·1 to 65·5)	−4·1 (−9·6 to 1·6)
		Other digestive diseases		3417·0 (2883·7 to 4228·0)	3770·5 (3180·0 to 4438·8)	10·3 (−0·1 to 22·2)	60·0 (51·0 to 73·9)	54·2 (45·9 to 63·7)	−9·7 (−18·0 to −0·4)*
	**Neurological disorders**			**74 456·9 (58 081·0 to 93 693·7)**	**87 082·1 (67 962·4 to 109 039·4)**	17·0 (15·5 to 18·5)*	**1291·0 (1025·5 to 1596·9)**	**1252·4 (987·9 to 1556·6)**	−3·0 (−4·2 to −2·0)*
		Alzheimer's disease and other dementias		17 905·9 (15 147·4 to 20 849·2)	23 779·2 (20 118·0 to 27 886·0)	32·8 (31·2 to 34·4)*	409·6 (348·0 to 479·2)	395·6 (333·5 to 463·8)	−3·4 (−4·4 to −2·5)*
		Parkinson's disease		1548·9 (1381·7 to 1747·5)	2059·3 (1831·8 to 2320·9)	32·9 (29·7 to 35·7)*	32·8 (29·4 to 36·8)	33·1 (29·5 to 37·2)	1·1 (−1·3 to 3·1)
		Epilepsy		12 892·1 (10 694·5 to 15 020·3)	12 417·9 (10 438·3 to 14 478·8)	−3·7 (−8·6 to 1·3)	194·1 (160·8 to 226·1)	167·6 (140·8 to 195·3)	−13·7 (−17·9 to −9·3)*
		Multiple sclerosis		1076·7 (913·9 to 1248·8)	1233·7 (1033·4 to 1436·9)	14·6 (9·9 to 18·4)*	18·1 (15·4 to 21·0)	16·9 (14·2 to 19·7)	−6·6 (−10·3 to −3·6)*
		Motor neuron disease		759·2 (728·7 to 806·3)	910·0 (872·2 to 958·5)	19·9 (13·6 to 23·2)*	13·6 (13·1 to 14·4)	13·1 (12·5 to 13·8)	−3·9 (−9·0 to −1·3)*
		Migraine		28 538·7 (17 584·9 to 42 479·8)	32 898·8 (20 294·8 to 48 945·4)	15·3 (14·0 to 16·6)*	435·2 (268·4 to 647·8)	438·7 (271·0 to 653·8)	0·8 (−0·1 to 1·8)
		Tension-type headache		1961·3 (915·9 to 3647·4)	2260·5 (1054·6 to 4193·3)	15·3 (14·0 to 16·7)*	30·0 (14·0 to 55·5)	30·2 (14·1 to 56·0)	0·6 (−0·1 to 1·3)
		Medication overuse headache		7705·4 (5100·2 to 10997·3)	9164·7 (6089·3 to 13080·8)	18·9 (15·4 to 22·8)*	123·0 (82·3 to 175·3)	123·7 (82·2 to 177·0)	0·6 (−2·3 to 3·5)
		Other neurological disorders		2070·4 (1941·6 to 2302·3)	2359·5 (2216·5 to 2565·4)	14·0 (9·4 to 17·5)*	34·6 (32·2 to 38·0)	33·6 (31·3 to 36·4)	−3·1 (−6·4 to −0·4)*
	**Mental and substance use disorders**			**141 375·1 (105 843·7 to 178 447·3)**	**162 442·3 (121 032·0 to 205 579·7)**	14·9 (14·1 to 15·7)*	**2189·2 (1646·0 to 2762·8)**	**2183·3 (1627·1 to 2766·3)**	**−0·3 (−1·0 to 0·3)**
		Schizophrenia		13 185·0 (9 635·3 to 16 203·0)	15 516·1 (11 279·2 to 19 137·0)	17·7 (16·5 to 18·8)*	210·4 (154·7 to 258·4)	207·5 (151·1 to 255·5)	−1·4 (−2·4 to −0·5)*
		Alcohol use disorders		11 566·7 (9 617·6 to 13 834·9)	11 194·3 (9 136·5 to 13 870·9)	−3·2 (−7·0 to 0·6)	183·4 (153·5 to 218·3)	149·4 (122·1 to 184·8)	−18·5 (−22·0 to −15·1)*
		Drug use disorders		13 671·4 (11 292·6 to 16 046·0)	16 909·5 (14 037·6 to 19 871·6)	23·7 (18·6 to 27·2)*	207·2 (171·5 to 242·7)	223·5 (185·8 to 262·2)	7·9 (3·5 to 10·9)*
			Opioid use disorders	9864·0 (8127·4 to 11516·8)	12 068·1 (9 878·0 to 14 145·1)	22·3 (17·5 to 26·1)*	150·2 (123·7 to 175·3)	159·3 (130·7 to 186·6)	6·0 (1·8 to 9·2)*
			Cocaine use disorders	729·3 (558·4 to 902·4)	999·3 (773·5 to 1 233·9)	37·0 (29·2 to 47·0)*	11·3 (8·7 to 14·0)	13·4 (10·4 to 16·6)	18·7 (12·1 to 27·3)*
			Amphetamine use disorders	1001·3 (706·2 to 1348·3)	1402·6 (1025·2 to 1846·9)	40·1 (26·1 to 55·2)*	14·6 (10·4 to 19·6)	18·4 (13·5 to 24·2)	25·8 (13·3 to 39·4)*
			Cannabis use disorders	548·1 (351·9 to 780·7)	577·2 (371·8 to 817·6)	5·3 (3·7 to 7·1)*	7·9 (5·0 to 11·2)	7·6 (4·9 to 10·7)	−3·7 (−5·0 to −2·3)*
			Other drug use disorders	1528·7 (1245·5 to 1861·2)	1862·2 (1501·6 to 2274·2)	21·8 (15·7 to 27·5)*	23·1 (19·0 to 28·0)	24·8 (20·0 to 30·2)	7·1 (1·7 to 12·0)*
		Depressive disorders		45 916·0 (31 684·6 to 61 591·2)	54 255·4 (37 513·6 to 72 968·9)	18·2 (17·2 to 19·2)*	726·9 (503·8 to 975·9)	734·2 (508·2 to 986·9)	1·0 (0·5 to 1·5)*
			Major depressive disorder	37 544·8 (24 983·0 to 51 134·6)	44 224·4 (29 542·6 to 60 430·5)	17·8 (16·6 to 19·0)*	590·9 (395·0 to 804·5)	597·2 (399·6 to 816·6)	1·1 (0·5 to 1·7)*
			Dysthymia	8 371·3 (5 537·6 to 11 983·7)	10 031·0 (6 604·4 to 14 289·3)	19·8 (18·3 to 21·5)*	136·1 (89·8 to 194·6)	137·0 (90·0 to 195·3)	0·7 (−0·2 to 1·6)
		Bipolar disorder		7838·8 (4787·8 to 11674·9)	9004·7 (5501·3 to 13396·4)	14·9 (13·9 to 15·9)*	119·0 (72·8 to 176·9)	119·6 (73·2 to 177·8)	0·5 (0·0 to 0·9)
		Anxiety disorders		21 474·8 (14 666·7 to 29 339·6)	24 643·0 (16 799·8 to 33 714·1)	14·8 (12·8 to 16·6)*	329·2 (225·4 to 448·4)	332·4 (227·0 to 453·8)	1·0 (−0·4 to 2·3)
		Eating disorders		1198·1 (806·7 to 1669·8)	1 421·7 (950·5 to 1 978·6)	18·7 (16·5 to 20·8)*	17·1 (11·5 to 23·7)	18·7 (12·5 to 26·0)	9·6 (7·7 to 11·4)*
			Anorexia nervosa	584·9 (396·7 to 829·1)	653·0 (439·3 to 929·4)	11·7 (8·7 to 14·6)*	8·2 (5·6 to 11·6)	8·6 (5·8 to 12·2)	4·6 (1·8 to 7·4)*
			Bulimia nervosa	613·3 (394·9 to 892·6)	768·7 (493·0 to 1113·8)	25·3 (23·2 to 27·6)*	8·8 (5·7 to 12·9)	10·1 (6·5 to 14·6)	14·2 (12·2 to 16·2)*
	Autistic spectrum disorders			8949·8 (5978·1 to 12 300·4)	10 051·5 (6718·9 to 13 804·4)	12·3 (11·9 to 12·8)*	134·7 (90·1 to 185·0)	135·5 (90·6 to 186·0)	0·6 (0·2 to 0·9)*
			Autism	5632·7 (3617·2 to 7920·0)	6335·9 (4070·1 to 8919·0)	12·5 (11·9 to 13·1)*	84·8 (54·5 to 119·2)	85·4 (54·9 to 120·3)	0·7 (0·2 to 1·2)*
			Asperger syndrome and other autistic spectrum disorders	3317·1 (2204·2 to 4849·0)	3715·6 (2467·7 to 5435·2)	12·0 (11·5 to 12·5)*	49·9 (33·2 to 72·9)	50·1 (33·3 to 73·2)	0·3 (−0·1 to 0·7)
		Attention-deficit hyperactivity disorder		617·9 (368·9 to 946·0)	620·1 (369·8 to 948·7)	0·4 (−0·6 to 1·2)	8·6 (5·1 to 13·2)	8·3 (5·0 to 12·7)	−3·5 (−4·4 to −2·8)*
		Conduct disorder		5740·5 (3459·2 to 8923·4)	5770·5 (3471·8 to 8956·9)	0·5 (−0·3 to 1·4)	78·2 (47·2 to 121·3)	79·3 (47·7 to 123·1)	1·4 (0·7 to 2·0)*
		Idiopathic developmental intellectual disability		3119·9 (1386·3 to 5416·5)	3442·1 (1503·1 to 5999·6)	10·3 (8·2 to 11·4)*	46·0 (20·4 to 80·0)	46·2 (20·1 to 80·5)	0·2 (−1·5 to 1·2)
		Other mental and substance use disorders		8095·9 (5632·9 to 10919·3)	9613·4 (6698·4 to 12966·0)	18·7 (17·7 to 19·8)*	128·4 (89·5 to 172·8)	128·8 (89·8 to 173·6)	0·3 (−0·6 to 1·1)
	**Diabetes, urogenital, blood, and endocrine diseases**			**122 917·1 (106 347·2 to 142 775·0)**	**146 780·6 (126 480·8 to 171 189·3)**	19·4 (16·4 to 22·5)*	**2123·6 (1847·2 to 2453·9)**	**2086·8 (1803·2 to 2422·8)**	**−1·7 (−4·0 to 0·6)**
		Diabetes mellitus		49 724·5 (41 868·2 to 58 982·1)	64 134·5 (53 489·7 to 76 112·8)	29·0 (26·2 to 31·7)*	911·2 (772·1 to 1 076·0)	925·8 (776·1 to 1 096·3)	1·6 (−0·5 to 3·7)
		Acute glomerulonephritis		412·3 (321·1 to 459·3)	337·9 (228·4 to 378·2)	−18·1 (−31·7 to −10·4)*	6·9 (5·4 to 7·7)	4·8 (3·2 to 5·3)	−30·6 (−42·4 to −24·1)*
		Chronic kidney disease		29 488·3 (26 950·4 to 31 604·2)	35 259·7 (32 008·1 to 37 763·0)	19·6 (16·0 to 23·2)*	524·4 (482·1 to 561·0)	508·8 (462·1 to 545·1)	−3·0 (−5·8 to −0·1)*
			Chronic kidney disease due to diabetes mellitus	8713·0 (7991·5 to 9466·3)	11 258·0 (10 302·9 to 12 225·2)	29·2 (25·9 to 32·5)*	159·0 (146·2 to 172·6)	163·3 (149·6 to 177·1)	2·7 (0·2 to 5·3)*
			Chronic kidney disease due to hypertension	10 366·1 (9 400·8 to 10 985·1)	12 737·4 (11 488·7 to 13 553·9)	22·9 (18·6 to 27·4)*	188·4 (172·1 to 199·7)	186·1 (168·1 to 197·9)	−1·2 (−4·6 to 2·3)
			Chronic kidney disease due to glomerulonephritis	7720·0 (6930·5 to 8332·1)	8136·0 (7294·2 to 8861·4)	5·4 (1·3 to 9·3)*	130·7 (117·3 to 140·8)	114·6 (103·0 to 124·6)	−12·3 (−15·6 to −9·0)*
			Chronic kidney disease due to other causes	2689·3 (2208·6 to 3237·9)	3128·3 (2517·9 to 3802·9)	16·3 (13·1 to 19·2)*	46·3 (37·7 to 55·8)	44·7 (36·0 to 54·1)	−3·4 (−5·6 to −1·5)*
		Urinary diseases and male infertility		7958·9 (6720·4 to 9447·1)	9653·3 (8096·4 to 11537·2)	21·3 (17·2 to 24·8)*	147·3 (123·4 to 175·7)	142·5 (119·0 to 170·6)	−3·3 (−6·3 to −0·5)*
			Interstitial nephritis and urinary tract infections	3399·6 (3134·3 to 3743·4)	3950·3 (3625·0 to 4408·2)	16·2 (11·4 to 21·5)*	60·6 (56·1 to 66·3)	57·6 (52·8 to 63·9)	−5·0 (−9·0 to −0·3)*
			Urolithiasis	448·5 (382·6 to 535·4)	456·1 (398·9 to 568·2)	1·7 (−4·7 to 10·8)	7·9 (6·7 to 9·4)	6·5 (5·7 to 8·1)	−17·3 (−22·5 to −9·6)*
			Benign prostatic hyperplasia	2922·0 (1867·0 to 4187·6)	3792·7 (2419·5 to 5453·5)	29·8 (27·6 to 32·2)*	58·6 (37·5 to 84·1)	57·9 (37·1 to 82·9)	−1·3 (−2·8 to 0·3)
			Male infertility	141·8 (57·7 to 293·6)	173·9 (69·7 to 365·9)	22·7 (19·2 to 25·9)*	2·1 (0·8 to 4·3)	2·3 (0·9 to 4·8)	9·8 (6·9 to 12·5)*
			Other urinary diseases	1047·1 (861·6 to 1213·3)	1280·2 (1044·9 to 1424·5)	22·3 (10·3 to 36·4)*	18·2 (15·1 to 21·1)	18·2 (14·9 to 20·2)	0·2 (−9·9 to 11·5)
		Gynaecological diseases		9292·8 (6396·3 to 13190·5)	10 255·1 (7 041·0 to 14 603·3)	10·4 (9·0 to 11·7)*	140·3 (96·5 to 199·6)	134·9 (92·6 to 192·2)	−3·8 (−4·9 to −2·8)*
		Uterine fibroids		2212·2 (1393·2 to 3481·1)	2452·6 (1514·9 to 3914·8)	10·9 (8·3 to 13·1)*	34·6 (21·7 to 54·4)	32·4 (20·0 to 51·8)	−6·1 (−8·1 to −4·4)*
		Polycystic ovarian syndrome		514·7 (245·2 to 972·0)	560·0 (263·4 to 1068·1)	8·8 (4·8 to 10·9)*	7·5 (3·6 to 14·2)	7·4 (3·5 to 14·0)	−2·6 (−6·5 to −0·6)*
		Female infertility		275·6 (106·1 to 606·6)	344·5 (133·4 to 748·2)	25·0 (19·7 to 30·1)*	4·0 (1·6 to 8·9)	4·5 (1·7 to 9·8)	11·7 (7·2 to 16·2)*
		Endometriosis		894·2 (580·2 to 1240·9)	999·0 (649·1 to 1390·3)	11·7 (10·4 to 12·9)*	13·4 (8·7 to 18·6)	13·0 (8·5 to 18·2)	−2·4 (−3·4 to −1·5)*
		Genital prolapse		449·1 (228·1 to 794·4)	519·3 (258·8 to 921·5)	15·6 (11·7 to 18·4)*	8·0 (4·1 to 14·0)	7·3 (3·6 to 12·9)	−8·4 (−11·8 to −5·9)*
		Premenstrual syndrome		3287·8 (2048·5 to 4917·5)	3621·6 (2248·2 to 5435·6)	10·2 (8·0 to 12·0)*	48·3 (30·1 to 72·2)	47·3 (29·4 to 71·0)	−2·0 (−3·9 to −0·4)*
		Other gynaecological diseases		1659·3 (1213·1 to 2208·2)	1758·0 (1278·9 to 2342·6)	6·0 (4·0 to 8·1)*	24·6 (18·0 to 32·6)	23·0 (16·8 to 30·6)	−6·3 (−8·1 to −4·5)*
	Haemoglobinopathies and haemolytic anaemias			20 211·2 (16 303·9 to 25 551·6)	20 604·4 (16 002·3 to 27 392·4)	1·9 (−11·4 to 19·6)	301·0 (243·6 to 378·6)	279·3 (217·4 to 370·5)	−7·2 (−18·9 to 8·7)
		Thalassaemias		1366·6 (1131·0 to 1683·8)	1034·6 (839·9 to 1272·9)	−24·3 (−39·0 to −2·4)*	19·8 (16·4 to 24·3)	14·0 (11·4 to 17·2)	−29·0 (−42·6 to −9·0)*
		Thalassaemias trait		3696·4 (2473·4 to 5333·5)	3922·2 (2605·5 to 5657·2)	6·1 (4·8 to 7·4)*	55·2 (36·9 to 79·6)	53·2 (35·3 to 76·7)	−3·6 (−4·7 to −2·6)*
		Sickle cell disorders		8662·7 (6150·3 to 13061·9)	8973·8 (5936·8 to 14752·8)	3·6 (−23·4 to 40·6)	124·4 (88·4 to 187·0)	120·3 (79·7 to 197·7)	−3·3 (−28·4 to 31·4)
		Sickle cell trait		1552·9 (1049·6 to 2211·1)	1720·3 (1153·9 to 2463·9)	10·8 (7·3 to 13·4)*	23·0 (15·5 to 32·7)	23·3 (15·6 to 33·4)	1·4 (−1·9 to 3·6)
		G6PD deficiency		1284·4 (1105·7 to 1525·4)	1440·5 (1225·4 to 1706·3)	12·1 (5·3 to 21·5)*	19·8 (17·0 to 23·4)	19·3 (16·4 to 22·9)	−2·4 (−8·4 to 5·3)
		G6PD trait		26·9 (18·4 to 37·1)	28·0 (19·2 to 38·9)	4·3 (0·1 to 8·8)*	0·4 (0·3 to 0·6)	0·4 (0·3 to 0·5)	−5·3 (−9·0 to −1·2)*
		Other haemoglobinopathies and haemolytic anaemias		3621·4 (2874·6 to 4634·1)	3485·1 (2770·9 to 4426·5)	−3·8 (−6·2 to −1·0)*	58·5 (47·2 to 73·7)	48·8 (39·1 to 61·7)	−16·5 (−19·2 to −13·8)*
		Endocrine, metabolic, blood, and immune disorders		5829·0 (5157·9 to 6691·1)	6535·7 (5812·5 to 7435·2)	12·1 (7·6 to 17·0)*	92·6 (82·5 to 105·6)	90·7 (80·9 to 103·0)	−2·1 (−5·8 to 1·7)
	**Musculoskeletal disorders**			**123 801·0 (90 494·0 to 163 616·5)**	**148 986·5 (108 880·7 to 197 118·2)**	20·3 (19·4 to 21·3)*	**2082·9 (1530·5 to 2750·7)**	**2065·1 (1510·9 to 2730·7)**	−0·9 (−1·5 to −0·2)*
		Rheumatoid arthritis		5204·7 (3777·3 to 6833·5)	6333·4 (4574·6 to 8332·9)	21·7 (18·7 to 24·7)*	90·9 (66·3 to 118·9)	89·7 (65·0 to 117·6)	−1·3 (−3·6 to 1·2)
		Osteoarthritis		9562·5 (6666·3 to 13 106·3)	12 886·2 (8 990·3 to 17 634·2)	34·8 (33·6 to 36·0)*	180·4 (125·9 to 247·2)	187·4 (130·8 to 256·4)	3·9 (3·0 to 4·8)*
		Low back and neck pain		80 053·5 (57 147·1 to 108 282·6)	94 941·5 (67 745·5 to 128 118·6)	18·6 (17·6 to 19·6)*	1337·3 (958·3 to 1802·5)	1309·7 (936·1 to 1765·1)	−2·1 (−2·7 to −1·4)*
			Low back pain	51 243·0 (36 375·0 to 70 714·6)	60 074·8 (42 682·1 to 82 419·2)	17·2 (16·4 to 18·1)*	852·0 (605·3 to 1 176·4)	829·5 (589·2 to 1 138·3)	−2·6 (−3·2 to −2·0)*
			Neck pain	28 810·5 (19 224·7 to 39 466·1)	34 866·7 (23 343·7 to 47 754·3)	21·0 (18·9 to 23·2)*	485·3 (327·2 to 662·1)	480·2 (322·0 to 653·6)	−1·1 (−2·3 to 0·1)
		Gout		1063·1 (719·2 to 1451·4)	1342·8 (908·5 to 1843·8)	26·3 (24·6 to 28·0)*	18·9 (12·9 to 25·8)	19·0 (12·9 to 26·1)	0·6 (−0·3 to 1·5)
		Other musculoskeletal disorders		27 917·2 (19 404·3 to 38 408·8)	33 482·6 (23 135·4 to 45 865·5)	19·9 (16·7 to 23·0)*	455·4 (316·9 to 623·9)	459·2 (318·0 to 630·7)	0·8 (−1·5 to 3·2)
	**Other non-communicable diseases**			**170 032·0 (132 096·5 to 221 007·5)**	**192 712·9 (148 719·0 to 251 205·4)**	13·3 (7·1 to 16·9)*	**2726·4 (2098·4 to 3558·8)**	**2687·2 (2068·1 to 3508·3)**	**−1·4 (−6·1 to 1·4)**
		Congenital anomalies		57 594·6 (49 489·5 to 69 397·4)	58 355·3 (52 597·9 to 64 983·3)	1·3 (−12·2 to 11·2)	826·5 (711·2 to 994·5)	781·1 (704·2 to 869·9)	−5·5 (−17·9 to 3·5)
			Neural tube defects	6925·7 (5218·4 to 9487·8)	5981·2 (4589·5 to 7574·7)	−13·6 (−32·7 to 5·7)	98·3 (74·1 to 134·8)	79·9 (61·3 to 101·2)	−18·8 (−36·7 to −0·6)*
			Congenital heart anomalies	26 778·2 (22 186·7 to 31 821·1)	25 706·4 (22 612·9 to 28 639·2)	−4·0 (−18·1 to 7·6)	382·7 (316·9 to 454·7)	343·9 (302·5 to 383·1)	−10·2 (−23·3 to 0·4)
			Cleft lip and cleft palate	355·3 (288·5 to 424·1)	196·0 (150·8 to 259·3)	−44·8 (−54·9 to −33·7)*	5·1 (4·1 to 6·1)	2·6 (2·0 to 3·5)	−48·3 (−57·6 to −38·1)*
			Down's syndrome	2379·1 (1571·3 to 3712·7)	2 401·1 (1 739·7 to 3 291·8)	0·9 (−23·2 to 29·4)	34·8 (23·1 to 53·9)	32·2 (23·3 to 44·0)	−7·5 (−29·2 to 17·8)
			Turner's syndrome	6·0 (2·1 to 12·9)	6·6 (2·4 to 14·4)	11·4 (8·7 to 14·0)*	0·1 (0·0 to 0·2)	0·1 (0·0 to 0·2)	−0·1 (−2·4 to 2·3)
			Klinefelter's syndrome	1·2 (0·4 to 2·8)	1·3 (0·5 to 3·2)	13·5 (10·6 to 15·6)*	0·0 (0·0 to 0·0)	0·0 (0·0 to 0·0)	0·2 (−2·2 to 2·0)
			Other chromosomal abnormalities	2110·0 (1124·3 to 4575·8)	2354·5 (1418·6 to 3905·9)	11·6 (−19·2 to 35·1)	30·4 (16·4 to 65·4)	31·5 (19·0 to 52·2)	3·6 (−24·3 to 25·0)
			Other congenital anomalies	19 039·2 (16 026·4 to 23 290·1)	21 708·1 (18 750·6 to 25 714·7)	14·0 (0·3 to 26·3)*	275·0 (231·4 to 336·5)	291·0 (251·2 to 344·6)	5·8 (−6·6 to 16·7)
		Skin and subcutaneous diseases		**42 069·1 (27 826·1 to 62 299·6)**	**47 222·7 (31 372·1 to 69 571·8)**	12·3 (11·5 to 13·2)*	**639·6 (426·1 to 942·5)**	**643·4 (428·2 to 946·7)**	0·6 (0·1 to 1·2)*
			Dermatitis	7733·8 (5244·5 to 10760·5)	8788·0 (5957·2 to 12275·5)	13·6 (12·9 to 14·4)*	117·8 (80·2 to 164·1)	119·7 (81·3 to 166·6)	1·6 (1·0 to 2·2)*
			Psoriasis	5478·8 (3816·1 to 7443·3)	6438·3 (4487·4 to 8755·9)	17·5 (16·6 to 18·4)*	87·4 (60·9 to 118·7)	87·9 (61·3 to 119·5)	0·6 (0·0 to 1·2)
			Cellulitis	406·1 (251·0 to 550·4)	498·2 (315·1 to 692·8)	22·7 (11·9 to 34·6)*	6·9 (4·3 to 9·4)	7·0 (4·5 to 9·8)	1·6 (−7·4 to 11·6)
			Pyoderma	1070·5 (714·3 to 1585·5)	1361·7 (932·3 to 2020·6)	27·2 (17·0 to 38·8)*	17·5 (11·7 to 25·6)	19·1 (13·1 to 28·2)	9·2 (0·6 to 18·9)*
			Scabies	4944·3 (2786·4 to 8050·4)	5268·9 (2947·0 to 8616·4)	6·6 (3·8 to 9·5)*	73·0 (41·2 to 118·8)	71·1 (39·8 to 116·0)	−2·5 (−4·5 to −0·5)*
			Fungal skin diseases	2457·6 (976·3 to 5214·9)	2783·3 (1101·3 to 5914·6)	13·3 (12·4 to 14·1)*	37·2 (14·7 to 79·0)	37·8 (15·0 to 80·3)	1·7 (1·4 to 2·0)*
			Viral skin diseases	4977·3 (3147·1 to 7362·6)	5396·9 (3412·0 to 7962·7)	8·4 (7·9 to 9·0)*	73·7 (46·7 to 109·3)	73·0 (46·2 to 107·9)	−1·0 (−1·4 to −0·6)*
			Acne vulgaris	6553·4 (3137·7 to 12159·0)	6854·0 (3271·3 to 12744·4)	4·6 (3·5 to 5·7)*	91·1 (43·7 to 169·0)	91·9 (43·9 to 171·0)	0·8 (−0·1 to 1·8)
			Alopecia areata	610·5 (376·1 to 911·7)	695·6 (427·5 to 1035·9)	13·9 (13·1 to 14·7)*	9·4 (5·8 to 14·0)	9·3 (5·7 to 13·8)	−1·0 (−1·7 to −0·4)*
			Pruritus	631·4 (306·3 to 1152·1)	741·6 (359·8 to 1349·6)	17·5 (15·5 to 19·4)*	10·1 (4·9 to 18·5)	10·2 (4·9 to 18·6)	0·5 (−0·7 to 1·7)
			Urticaria	3718·5 (2297·3 to 5286·3)	4115·7 (2560·6 to 5836·2)	10·7 (9·5 to 12·0)*	55·5 (34·5 to 78·7)	55·5 (34·6 to 78·7)	0·0 (−0·5 to 0·4)
			Decubitus ulcer	494·1 (328·7 to 602·5)	609·1 (415·1 to 738·7)	23·3 (17·3 to 33·2)*	9·8 (6·5 to 11·9)	9·4 (6·4 to 11·3)	−4·3 (−8·9 to 3·7)*
			Other skin and subcutaneous diseases	2992·7 (1486·8 to 5413·5)	3671·3 (1817·7 to 6638·7)	22·7 (21·9 to 23·5)*	50·2 (24·9 to 90·8)	51·5 (25·5 to 93·2)	2·6 (2·1 to 3·3)*
		Sense organ diseases		**54 708·4 (38 076·6 to 75 346·3)**	**68 515·2 (47 752·5 to 94 246·6)**	25·2 (24·2 to 26·4)*	**993·6 (694·9 to 1 361·8)**	**999·9 (697·1 to 1 370·3)**	**0·6 (−0·1 to 1·3)**
			Glaucoma	388·4 (268·1 to 538·9)	541·3 (369·5 to 748·0)	39·4 (37·0 to 41·9)*	8·0 (5·5 to 11·2)	8·4 (5·7 to 11·6)	4·3 (2·6 to 5·8)*
			Cataract	3091·3 (2213·8 to 4152·6)	3879·7 (2766·1 to 5232·4)	25·5 (24·1 to 26·9)*	62·9 (45·1 to 84·3)	60·3 (43·0 to 81·0)	−4·2 (−5·2 to −3·2)*
			Macular degeneration	313·2 (219·5 to 429·6)	462·4 (327·4 to 633·5)	47·7 (45·2 to 49·9)*	6·9 (4·9 to 9·5)	7·4 (5·2 to 10·1)	6·9 (5·3 to 8·6)*
			Refraction and accommodation disorders	12 046·9 (7737·6 to 18 905·4)	14 593·8 (9388·5 to 22 942·4)	21·1 (20·2 to 22·1)*	207·8 (133·9 to 326·0)	207·3 (133·7 to 324·8)	−0·3 (−0·9 to 0·4)
			Age-related and other hearing loss	32 119·0 (21 992·4 to 44 438·1)	40 596·8 (27 842·2 to 56 168·9)	26·4 (24·6 to 28·3)*	589·4 (405·6 to 817·1)	595·9 (409·6 to 825·0)	1·1 (0·0 to 2·1)
			Other vision loss	1352·0 (957·1 to 1852·2)	1756·4 (1247·4 to 2393·6)	29·9 (27·8 to 32·1)*	23·9 (17·0 to 32·5)	25·2 (17·9 to 34·1)	5·6 (4·3 to 6·7)*
			Other sense organ diseases	5397·7 (3358·7 to 7841·8)	6684·7 (4178·4 to 9722·8)	23·8 (22·9 to 24·8)*	94·7 (59·3 to 137·2)	95·5 (59·9 to 138·6)	0·9 (0·2 to 1·5)*
		Oral disorders		**13 868·2 (8387·2 to 21 285·6)**	**16 969·6 (10 280·0 to 26 059·7)**	22·4 (21·6 to 23·2)*	**241·3 (147·2 to 367·9)**	**240·8 (146·9 to 367·8)**	**−0·2 (−0·5 to 0·1)**
			Deciduous caries	141·4 (61·3 to 279·6)	147·2 (62·8 to 292·3)	4·1 (1·6 to 5·9)*	2·1 (0·9 to 4·1)	2·0 (0·9 to 4·0)	−2·7 (−4·9 to −0·8)*
			Permanent caries	1536·3 (681·5 to 2960·1)	1743·4 (773·5 to 3358·0)	13·5 (12·6 to 14·4)*	23·7 (10·5 to 45·7)	23·6 (10·5 to 45·5)	−0·4 (−1·2 to 0·4)
			Periodontal diseases	2808·4 (1084·9 to 5841·7)	3520·7 (1357·6 to 7337·3)	25·4 (24·1 to 26·5)*	48·0 (18·6 to 100·0)	48·6 (18·8 to 101·2)	1·2 (0·6 to 1·8)*
			Edentulism and severe tooth loss	5999·5 (4012·3 to 8293·2)	7640·3 (5092·8 to 10572·8)	27·3 (26·9 to 27·7)*	114·6 (76·7 to 158·5)	113·6 (76·0 to 157·3)	−0·8 (−1·1 to −0·6)*
			Other oral disorders	3382·7 (2093·2 to 5085·7)	3918·1 (2423·4 to 5894·1)	15·8 (15·3 to 16·4)*	53·0 (32·8 to 79·5)	53·0 (32·8 to 79·6)	0·0 (−0·2 to 0·3)
		Sudden infant death syndrome		1791·7 (1458·5 to 2904·4)	1650·1 (1366·4 to 2367·7)	−7·9 (−23·0 to 8·9)	25·5 (20·7 to 41·3)	22·0 (18·2 to 31·6)	−13·5 (−27·7 to 2·3)
**Injuries**				**263 084·3 (245 566·5 to 278 621·5)**	**249 791·3 (231 409·2 to 266 419·2)**	−5·1 (−8·5 to −0·9)*	**4044·0 (3764·8 to 4300·1)**	**3376·1 (3120·8 to 3607·0)**	−16·5 (−19·4 to −13·0)*
	**Transport injuries**			**77 146·4 (73 768·7 to 80 734·9)**	**72 365·5 (68 372·4 to 76 237·9)**	−6·2 (−11·1 to −1·8)*	**1175·5 (1121·9 to 1231·3)**	**971·4 (917·0 to 1024·0)**	−17·4 (−21·6 to −13·5)*
		Road injuries		71 941·1 (68 596·1 to 75 312·5)	67 270·4 (63 538·6 to 70 859·9)	−6·5 (−11·4 to −2·2)*	1095·3 (1042·8 to 1145·8)	903·0 (854·0 to 951·5)	−17·6 (−21·8 to −13·8)*
			Pedestrian road injuries	27 185·9 (25 567·5 to 29 487·1)	24 491·1 (22 747·9 to 26 770·6)	−9·9 (−16·4 to −3·8)*	417·2 (392·7 to 452·1)	330·1 (307·3 to 360·7)	−20·9 (−26·5 to −15·5)*
			Cyclist road injuries	3721·2 (3357·8 to 4119·6)	3399·8 (3022·9 to 3807·0)	−8·6 (−15·1 to −2·1)*	58·5 (52·7 to 65·1)	46·3 (41·1 to 52·0)	−20·9 (−26·3 to −15·4)*
			Motorcyclist road injuries	13 655·4 (12 181·3 to 14 651·4)	13 686·2 (12 440·3 to 15 369·7)	0·2 (−7·8 to 10·6)	204·9 (182·3 to 219·9)	182·4 (165·7 to 204·3)	−10·9 (−17·9 to −1·7)*
			Motor vehicle road injuries	26 257·7 (24 307·0 to 28 943·8)	24 460·3 (22 362·4 to 26 719·4)	−6·8 (−11·6 to −1·8)*	397·6 (368·7 to 437·0)	327·5 (299·3 to 357·3)	−17·6 (−21·8 to −13·2)*
			Other road injuries	1120·9 (782·6 to 1331·6)	1233·1 (858·6 to 1446·6)	10·0 (−5·2 to 31·3)	17·1 (12·1 to 20·2)	16·6 (11·6 to 19·4)	−2·9 (−16·0 to 15·4)
		Other transport injuries		5205·3 (4772·9 to 5728·4)	5095·1 (4507·0 to 5967·9)	−2·1 (−10·5 to 7·5)	80·2 (73·6 to 88·4)	68·4 (60·6 to 80·2)	−14·7 (−21·8 to −6·7)*
	**Unintentional injuries**			**118 001·9 (105 534·5 to 129 998·5)**	**107 990·9 (95 660·4 to 120 900·3)**	−8·5 (−11·9 to −3·4)*	**1840·5 (1642·4 to 2033·1)**	**1481·0 (1310·3 to 1660·5)**	−19·5 (−22·2 to −15·3)*
		Falls		23 912·3 (20 767·3 to 27 587·9)	26 101·6 (22 363·6 to 30 779·5)	9·2 (3·6 to 14·6)*	403·3 (349·6 to 467·0)	368·3 (315·1 to 435·0)	−8·7 (−13·1 to −4·4)*
		Drowning		24 207·3 (20 715·5 to 25 812·8)	17 864·3 (15 792·7 to 19 306·8)	−26·2 (−30·6 to −20·0)*	356·2 (305·1 to 379·5)	241·1 (213·0 to 260·6)	−32·3 (−36·3 to −26·7)*
		Fire, heat, and hot substances		11 737·6 (9 928·4 to 12 948·0)	10 274·1 (8 701·9 to 11 443·4)	−12·5 (−17·4 to −5·1)*	179·7 (153·4 to 198·0)	139·0 (117·7 to 155·0)	−22·6 (−26·8 to −16·6)*
		Poisonings		5746·4 (3978·7 to 7215·7)	4866·9 (3240·1 to 6070·3)	−15·3 (−28·9 to 3·6)	86·6 (60·4 to 107·8)	65·5 (43·6 to 81·5)	−24·4 (−36·0 to −7·5)*
		Exposure to mechanical forces		13 753·4 (12 020·2 to 15 236·7)	13 118·4 (10 878·2 to 14 847·6)	−4·6 (−11·8 to 1·7)	210·2 (183·6 to 234·3)	177·4 (146·9 to 201·3)	−15·6 (−21·7 to −10·2)*
			Unintentional firearm injuries	1655·9 (1188·2 to 1837·5)	1546·4 (1101·5 to 1727·2)	−6·6 (−11·9 to −0·6)*	24·9 (18·0 to 27·6)	20·7 (14·8 to 23·1)	−16·8 (−21·4 to −11·8)*
			Unintentional suffocation	2647·9 (2075·2 to 3023·5)	2495·6 (1885·3 to 2883·4)	−5·8 (−17·6 to 7·8)	39·3 (30·8 to 44·8)	33·9 (25·7 to 39·1)	−13·7 (−24·3 to −1·8)*
			Other exposure to mechanical forces	9449·6 (8051·7 to 10717·9)	9076·5 (7327·9 to 10524·0)	−3·9 (−11·4 to 3·1)	146·0 (124·4 to 166·3)	122·8 (99·1 to 142·7)	−15·9 (−22·2 to −10·1)*
		Adverse effects of medical treatment		5671·1 (4245·4 to 6758·2)	5384·9 (4213·4 to 6360·5)	−5·0 (−10·5 to 2·7)	89·8 (67·8 to 106·9)	74·1 (58·2 to 87·7)	−17·4 (−21·6 to −11·7)*
		Animal contact		6453·3 (4501·0 to 7223·6)	5661·2 (3760·0 to 7514·8)	−12·3 (−19·9 to 10·9)	97·6 (68·4 to 109·2)	76·6 (50·8 to 101·5)	−21·5 (−28·2 to −0·7)*
			Venomous animal contact	5306·6 (3504·8 to 5989·8)	4648·0 (2901·8 to 6463·8)	−12·4 (−20·6 to 12·9)	80·1 (53·1 to 90·4)	62·8 (39·2 to 87·3)	−21·6 (−29·0 to 1·1)
			Non-venomous animal contact	1146·7 (920·0 to 1358·6)	1013·2 (840·1 to 1180·4)	−11·6 (−20·1 to 5·0)	17·5 (14·1 to 20·6)	13·8 (11·4 to 16·1)	−21·2 (−28·5 to −6·9)*
		Foreign body		8668·4 (6684·4 to 10942·0)	8244·8 (6786·9 to 9635·2)	−4·9 (−14·1 to 4·8)	132·2 (103·4 to 165·2)	112·4 (92·8 to 131·1)	−15·0 (−22·5 to −7·3)*
			Pulmonary aspiration and foreign body in airway	6632·4 (4907·7 to 8950·1)	6321·8 (5088·3 to 7704·2)	−4·7 (−15·7 to 6·1)	101·0 (76·1 to 134·5)	86·2 (69·6 to 104·7)	−14·7 (−23·9 to −6·2)*
			Foreign body in eyes	48·8 (20·8 to 87·9)	56·2 (24·0 to 101·1)	15·2 (13·8 to 16·7)*	0·8 (0·3 to 1·4)	0·8 (0·3 to 1·4)	0·5 (−0·9 to 1·3)
			Foreign body in other body part	1987·3 (1331·1 to 2717·2)	1866·9 (1394·9 to 2247·3)	−6·1 (−19·8 to 6·4)	30·4 (20·8 to 41·4)	25·4 (19·1 to 30·6)	−16·4 (−28·8 to −6·5)*
		Environmental heat and cold exposure		4503·5 (3641·3 to 5457·2)	4234·7 (3350·6 to 5294·9)	−6·0 (−10·1 to −1·4)*	74·1 (59·9 to 89·9)	58·7 (46·4 to 73·6)	−20·7 (−24·0 to −17·1)*
		Other unintentional injuries		13 348·6 (11 406·7 to 15 619·1)	12 240·0 (10 234·9 to 14 692·0)	−8·3 (−12·9 to −3·8)*	210·9 (178·2 to 249·5)	167·9 (139·9 to 202·4)	−20·4 (−24·1 to −16·6)*
	**Self-harm and interpersonal violence**			**58 615·7 (52 794·8 to 60 379·5)**	**55 719·4 (50 909·8 to 58 127·7)**	−4·9 (−9·0 to −0·8)*	**889·6 (801·0 to 916·0)**	**741·1 (676·7 to 773·2)**	−16·7 (−20·3 to −13·0)*
		Self-harm		35 819·1 (31 467·1 to 37 112·4)	34 260·9 (30 852·1 to 36 042·4)	−4·4 (−10·4 to 1·6)	551·9 (484·7 to 571·2)	457·9 (412·2 to 481·5)	−17·0 (−22·3 to −11·9)*
		Interpersonal violence		22 796·5 (20 747·8 to 23 566·7)	21 458·5 (19 354·8 to 22 540·1)	−5·9 (−9·0 to −2·0)*	337·6 (308·1 to 349·1)	283·3 (255·6 to 297·4)	−16·1 (−18·8 to −12·7)*
			Assault by firearm	8856·3 (7747·9 to 9172·1)	9188·1 (7854·5 to 9689·2)	3·7 (0·0 to 8·2)	129·0 (113·0 to 133·6)	120·7 (103·2 to 127·3)	−6·4 (−9·9 to −2·4)*
			Assault by sharp object	5425·2 (5050·4 to 5731·6)	4546·1 (4223·1 to 4937·8)	−16·2 (−20·6 to −11·2)*	80·5 (75·1 to 85·0)	59·9 (55·6 to 65·0)	−25·6 (−29·6 to −21·2)*
			Assault by other means	8515·1 (7723·3 to 9065·4)	7724·3 (6927·0 to 8395·8)	−9·3 (−14·2 to −2·7)*	128·2 (116·6 to 136·2)	102·7 (92·2 to 111·6)	−19·8 (−24·2 to −14·1)*
	**Forces of nature, war, and legal intervention**			**9320·3 (6951·1 to 11 778·4)**	**13 715·5 (8561·9 to 18 537·5)**	**47·2 (−1·9 to 100·5)**	**138·4 (103·8 to 174·8)**	**182·6 (114·4 to 246·3)**	**31·9 (−11·6 to 79·5)**
		Exposure to forces of nature		6203·9 (4037·7 to 8412·0)	1435·5 (1092·6 to 1846·4)	−76·9 (−81·2 to −69·4)*	91·9 (60·2 to 124·2)	19·6 (14·9 to 25·2)	−78·7 (−82·7 to −72·0)*
		Collective violence and legal intervention		3116·4 (2504·2 to 3760·8)	12 279·9 (7208·0 to 17 075·2)	294·0 (175·8 to 394·3)*	46·5 (37·4 to 56·1)	163·0 (95·9 to 226·4)	250·5 (144·1 to 341·2)*

Data in parentheses are 95% uncertainty intervals. DALYs=disability-adjusted life-years. G6PD=glucose-6-phosphate dehydrogenase.

* Percentage changes that are statistically significant.

**Table 2:** Global, regional, and national or territory life expectancy and HALE at birth, by sex, in 2005 and 2015, and HALE at age 65, by sex, in 2015

			**2005, at birth**				**2015, at birth**				**2015, at age 65 years**			
			Females		Males		Females		Males		Females		Males	
			Life expectancy	HALE	Life expectancy	HALE	Life expectancy	HALE	Life expectancy	HALE	Life expectancy	HALE	Life expectancy	HALE
Global			70·73 (70·48–70·96)	61·37 (58·61–63·76)	65·69 (65.45–65·93)	57·96 (55·69–59·98)	74·83 (74·39–75·25)	64·88 (61·96–67·46)	69·04 (68·63–69·42)	60·91 (58·55–63·01)	18·46 (18·31–18·61)	14·19 (12·98–15·28)	15·45 (15·31–15·59)	11·90 (10·89–12·81)
	High SDI		80·26 (80·23–80·28)	69·62 (66·53–72·30)	73·25 (73·20–73·29)	64·82 (62·36–66·96)	82·34 (82·26–82·41)	71·33 (68·13–74·13)	76·21 (76·10–76·31)	67·27 (64·70–69·54)	20·96 (20·92–21·01)	16·18 (14·83–17·38)	17·66 (17·62–17·70)	13·55 (12·38–14·58)
	High-middle SDI		75·26 (75·02–75·51)	65·85 (63·11–68·23)	69·26 (68·96–69·53)	61·50 (59·22–63·59)	79·13 (78·80–79·43)	69·09 (66·18–71·63)	72·69 (72·25–73·10)	64·46 (62·01–66·60)	19·20 (19·01–19·40)	14·90 (13·69–16·00)	15·81 (15·58–16·01)	12·30 (11·29–13·21)
	Middle SDI		71·83 (71·49–72·19)	62·72 (60·08–65·04)	66·80 (66·43–67·19)	59·31 (57·11–61·26)	75·71 (75·21–76·19)	66·02 (63·24–68·52)	69·55 (68·99–70·14)	61·74 (59·46–63·75)	17·33 (17·03–17·62)	13·36 (12·19–14·40)	14·30 (14·01–14·59)	11·13 (10·24–11·95)
	Low-middle SDI		62·59 (61·97–63·21)	53·65 (51·01–56·01)	60·30 (59·69–60·86)	52·55 (50·22–54·66)	67·72 (66·80–68·61)	58·18 (55·33–60·84)	63·83 (62·99–64·62)	55·85 (53·44–58·01)	14·75 (14·34–15·16)	11·00 (9·95–12·05)	13·39 (13·06–13·70)	10·09 (9·18–10·94)
	Low SDI		56·19 (55·16–57·24)	48·18 (45·46–50·55)	54·49 (53·52–55·45)	47·34 (45·02–49·45)	62·30 (60·22–64·27)	53·64 (50·41–56·49)	59·13 (56·85–61·28)	51·66 (48·91–54·31)	13·35 (12·36–14·29)	9·88 (8·61–11·06)	12·78 (11·77–13·68)	9·55 (8·41–10·63)
	High-income		82·09 (82·07–82·11)	71·13 (67·95–73·87)	76·35 (76·33–76·37)	67·48 (64·90–69·75)	83·42 (83·38–83·46)	72·21 (68·97–75·02)	78·10 (78·05–78·15)	68·91 (66·27–71·25)	21·68 (21·65–21·70)	16·78 (15·39–18·00)	18·24 (18·22–18·27)	14·03 (12·82–15·09)
High-income North America			80·43 (80·39–80·47)	68·84 (65·64–71·64)	75·33 (75·28–75·37)	65·86 (63·17–68·24)	81·75 (81·68–81·82)	69·80 (66·51–72·75)	77·02 (76·95–77·10)	67·11 (64·32–69·65)	20·80 (20·74–20·86)	15·56 (14·12–16·84)	18·17 (18·12–18·21)	13·50 (12·21–14·67)
	Canada		82·53 (82·48–82·60)	71·39 (68·19–74·21)	77·81 (77·75–77·86)	68·29 (65·47–70·74)	83·86 (83·66–84·08)	72·49 (69·32–75·37)	79·50 (79·28–79·72)	69·64 (66·80–72·16)	21·87 (21·72–22·02)	16·77 (15·33–18·04)	18·81 (18·68–18·94)	14·29 (13·00–15·44)
	Greenland		71·07 (70·24–71·91)	61·63 (59·00–64·06)	67·44 (66·72–68·12)	59·74 (57·50–61·80)	74·90 (73·77–75·71)	64·81 (61·93–67·47)	70·00 (69·06–70·94)	61·90 (59·46–64·13)	16·76 (16·23–17·09)	12·65 (11·46–13·71)	14·26 (14·01–14·52)	10·79 (9·82–11·66)
	USA		80·21 (80·17–80·25)	68·57 (65·38–71·38)	75·06 (75·00–75·11)	65·60 (62·93–67·96)	81·52 (81·44–81·60)	69·50 (66·21–72·47)	76·74 (76·66–76·83)	66·82 (64·05–69·35)	20·68 (20·62–20·74)	15·42 (13·99–16·71)	18·09 (18·04–18·14)	13·41 (12·12–14·58)
	Australasia		83·25 (83·20–83·31)	71·66 (68·37–74·50)	78·57 (78·52–78·62)	68·83 (66·11–71·26)	84·32 (84·21–84·44)	72·57 (69·31–75·54)	80·09 (79·98–80·20)	70·08 (67·24–72·60)	22·01 (21·93–22·09)	17·06 (15·65–18·29)	19·11 (19·05–19·18)	14·66 (13·42–15·79)
	Australia		83·55 (83·49–83·61)	71·88 (68·59–74·74)	78·76 (78·70–78·82)	68·94 (66·19–71·38)	84·52 (84·41–84·65)	72·72 (69·43–75·71)	80·19 (80·08–80·31)	70·12 (67·25–72·66)	22·13 (22·04–22·22)	17·15 (15·75–18·40)	19·16 (19·08–19·23)	14·67 (13·43–15·82)
	New Zealand		81·80 (81·67–81·93)	70·59 (67·45–73·42)	77·61 (77·48–77·73)	68·32 (65·64–70·68)	83·29 (83·03–83·53)	71·83 (68·60–74·82)	79·58 (79·30–79·88)	69·94 (67·20–72·40)	21·39 (21·20–21·55)	16·57 (15·20–17·82)	18·89 (18·74–19·06)	14·59 (13·37–15·68)
High-income Asia Pacific			84·71 (84·67–84·76)	74·76 (71·79–77·29)	77·77 (77·70–77·83)	69·68 (67·35–71·75)	85·95 (85·85–86·05)	75·82 (72·83–78·39)	79·39 (79·24–79·53)	71·05 (68·64–73·16)	23·39 (23·32–23·46)	18·66 (17·29–19·85)	18·67 (18·61–18·73)	14·63 (13·48–15·62)
	Brunei		78·95 (78·49–79·31)	69·48 (66·76–72·02)	74·80 (74·36–75·15)	66·88 (64·59–69·00)	79·36 (78·84–79·87)	69·96 (67·21–72·39)	75·13 (74·43–75·87)	67·20 (64·83–69·39)	18·77 (18·50–19·01)	14·81 (13·69–15·83)	15·96 (15·61–16·33)	12·45 (11·43–13·43)
	Japan		85·34 (85·31–85·37)	75·43 (72·48–77·97)	78·46 (78·44–78·49)	70·32 (67·99–72·38)	86·35 (86·28–86·41)	76·28 (73·33–78·85)	79·94 (79·88–80·00)	71·54 (69·14–73·67)	23·78 (23·73–23·84)	19·04 (17·65–20·25)	18·94 (18·91–18·98)	14·85 (13·69–15·88)
	Singapore		82·17 (81·99–82·35)	73·34 (70·67–75·64)	77·88 (77·70–78·05)	70·62 (68·52–72·48)	83·99 (83·69–84·35)	75·02 (72·28–77·44)	79·83 (79·45–80·18)	72·31 (70·06–74·21)	21·19 (20·97–21·46)	17·24 (16·02–18·32)	18·19 (17·94–18·42)	14·75 (13·69–15·65)
	South Korea		81·97 (81·79–82·15)	71·92 (68·98–74·42)	75·22 (74·95–75·50)	67·35 (65·02–69·41)	84·28 (83·91–84·64)	73·97 (70·91–76·61)	77·53 (76·98–78·05)	69·38 (66·95–71·51)	21·66 (21·39–21·91)	16·97 (15·58–18·18)	17·33 (17·02–17·62)	13·49 (12·34–14·50)
Western Europe			82·60 (82·59–82·62)	71·55 (68·33–74·39)	76·99 (76·98–77·01)	68·13 (65·50–70·42)	83·97 (83·91–84·03)	72·75 (69·44–75·62)	78·93 (78·87–79·00)	69·82 (67·16–72·15)	21·64 (21·60–21·67)	16·79 (15·38–18·01)	18·29 (18·25–18·32)	14·20 (12·99–15·23)
	Andorra		87·91 (87·62–88·21)	75·88 (72·38–78·96)	80·38 (79·98–80·77)	70·89 (68·11–73·38)	88·44 (88·12–88·73)	76·30 (72·75–79·42)	81·20 (80·80–81·57)	71·57 (68·74–74·03)	24·99 (24·73–25·23)	19·38 (17·77–20·82)	19·53 (19·32–19·74)	15·16 (13·88–16·31)
	Austria		82·22 (82·15–82·30)	71·19 (67·98–74·02)	76·63 (76·55–76·71)	67·65 (65·05–69·96)	83·66 (83·52–83·79)	72·42 (69·15–75·31)	78·81 (78·66–78·95)	69·59 (66·86–71·97)	21·34 (21·25–21·43)	16·56 (15·16–17·79)	18·21 (18·13–18·29)	14·12 (12·93–15·18)
	Belgium		81·97 (81·87–82·07)	70·88 (67·69–73·65)	76·18 (76·08–76·29)	67·18 (64·52–69·46)	83·19 (82·82–83·56)	71·96 (68·66–74·88)	77·83 (77·37–78·27)	68·62 (65·99–71·05)	21·22 (20·97–21·49)	16·29 (14·84–17·59)	17·62 (17·36–17·88)	13·47 (12·27–14·58)
	Cyprus		82·15 (81·97–82·33)	71·02 (67·78–73·89)	75·90 (75·73–76·07)	67·09 (64·50–69·34)	85·05 (84·80–85·29)	73·41 (69·97–76·38)	78·69 (78·38–78·99)	69·38 (66·65–71·82)	22·49 (22·31–22·66)	17·33 (15·81–18·67)	17·95 (17·77–18·13)	13·78 (12·58–14·90)
	Denmark		80·50 (80·39–80·62)	69·73 (66·63–72·49)	76·08 (75·98–76·19)	67·24 (64·67–69·51)	82·44 (82·15–82·72)	71·39 (68·26–74·21)	78·31 (78·03–78·60)	69·21 (66·59–71·56)	20·43 (20·24–20·62)	15·83 (14·49–17·01)	17·64 (17·47–17·80)	13·74 (12·63–14·71)
	Finland		82·43 (82·30–82·55)	71·05 (67·72–74·00)	75·55 (75·41–75·68)	66·44 (63·69–68·82)	83·87 (83·60–84·15)	72·37 (68·92–75·31)	77·97 (77·62–78·32)	68·59 (65·86–70·98)	21·57 (21·39–21·76)	16·59 (15·12–17·84)	17·97 (17·79–18·16)	13·83 (12·60–14·90)
	France		83·90 (83·87–83·94)	72·66 (69·29–75·57)	76·81 (76·78–76·84)	68·01 (65·38–70·31)	85·17 (85·01–85·33)	73·83 (70·44–76·73)	78·39 (78·17–78·60)	69·30 (66·55–71·64)	23·00 (22·89–23·11)	17·91 (16·38–19·21)	18·69 (18·58–18·80)	14·32 (12·99–15·43)
	Germany		82·06 (82·03–82·10)	70·86 (67·65–73·74)	76·58 (76·54–76·61)	67·72 (65·12–70·01)	83·19 (83·07–83·30)	71·85 (68·59–74·75)	78·36 (78·23–78·49)	69·31 (66·67–71·65)	20·95 (20·88–21·03)	16·23 (14·87–17·40)	17·80 (17·73–17·87)	13·88 (12·74–14·88)
	Greece		82·34 (82·25–82·43)	71·62 (68·49–74·37)	76·82 (76·73–76·92)	68·22 (65·71–70·46)	83·49 (83·25–83·74)	72·64 (69·46–75·42)	78·42 (78·10–78·72)	69·60 (67·00–71·88)	20·96 (20·78–21·14)	16·38 (15·01–17·54)	18·30 (18·12–18·47)	14·30 (13·13–15·34)
	Iceland		83·64 (83·28–84·06)	72·37 (69·13–75·24)	79·65 (79·44–79·92)	70·50 (67·82–72·89)	85·82 (85·38–86·30)	74·35 (70·99–77·33)	80·93 (80·60–81·25)	71·60 (68·91–73·99)	23·00 (22·63–23·43)	17·83 (16·29–19·21)	19·25 (19·07–19·43)	14·93 (13·69–16·05)
	Ireland		81·77 (81·49–82·11)	70·80 (67·53–73·65)	77·65 (77·39–77·86)	68·43 (65·76–70·81)	84·31 (83·60–85·02)	72·94 (69·60–76·00)	79·18 (78·94–79·51)	69·74 (66·95–72·16)	21·98 (21·36–22·66)	17·09 (15·66–18·46)	18·30 (18·08–18·61)	14·14 (12·92–15·20)
	Israel		81·73 (81·57–81·87)	70·97 (67·90–73·67)	77·63 (77·46–77·77)	68·60 (65·94–70·92)	83·82 (83·55–84·07)	72·69 (69·47–75·47)	80·18 (79·92–80·47)	70·72 (67·94–73·16)	21·29 (21·09–21·47)	16·44 (15·05–17·64)	19·10 (18·93–19·28)	14·72 (13·43–15·83)
	Italy		83·73 (83·69–83·77)	72·34 (69·02–75·27)	78·29 (78·26–78·33)	69·36 (66·72–71·67)	84·57 (84·44–84·71)	73·10 (69·76–76·01)	79·64 (79·48–79·78)	70·58 (67·86–72·95)	21·90 (21·80–21·99)	16·87 (15·44–18·12)	18·37 (18·27–18·45)	14·27 (13·05–15·31)
	Luxembourg		82·51 (82·32–82·73)	70·98 (67·62–73·95)	77·24 (77·03–77·44)	67·78 (64·99–70·19)	84·29 (84·01–84·62)	72·47 (69·05–75·42)	79·86 (79·60–80·12)	70·09 (67·20–72·60)	21·79 (21·59–22·05)	16·67 (15·16–17·96)	18·69 (18·54–18·83)	14·31 (13·05–15·45)
	Malta		82·73 (82·33–83·10)	71·52 (68·23–74·41)	78·32 (77·92–78·69)	69·06 (66·33–71·43)	84·48 (84·15–84·84)	72·97 (69·61–75·95)	79·65 (79·26–80·06)	70·17 (67·29–72·59)	22·05 (21·80–22·33)	17·03 (15·60–18·34)	18·67 (18·43–18·92)	14·48 (13·25–15·55)
	Netherlands		81·63 (81·53–81·73)	70·43 (67·22–73·29)	77·33 (77·24–77·42)	68·31 (65·65–70·67)	83·40 (83·08–83·72)	71·93 (68·64–74·87)	79·17 (78·86–79·52)	69·89 (67·22–72·30)	21·22 (21·02–21·47)	16·28 (14·85–17·52)	17·78 (17·59–18·00)	13·75 (12·58–14·80)
	Norway		82·42 (82·27–82·58)	70·96 (67·71–73·85)	77·81 (77·66–77·95)	68·50 (65·80–70·87)	84·08 (83·81–84·34)	72·56 (69·27–75·55)	79·94 (79·68–80·21)	70·40 (67·62–72·90)	21·54 (21·35–21·73)	16·65 (15·28–17·91)	18·57 (18·41–18·74)	14·36 (13·14–15·48)
	Portugal		81·74 (81·66–81·83)	70·60 (67·34–73·41)	75·06 (74·96–75·14)	66·36 (63·75–68·59)	83·86 (83·69–84·02)	72·40 (69·05–75·36)	77·65 (77·47–77·83)	68·61 (65·93–70·95)	21·38 (21·26–21·49)	16·48 (15·07–17·73)	17·71 (17·62–17·81)	13·72 (12·59–14·76)
	Spain		83·63 (83·59–83·67)	72·81 (69·58–75·61)	76·91 (76·87–76·95)	68·29 (65·70–70·55)	85·27 (85·17–85·38)	74·27 (70·99–77·13)	79·84 (79·72–79·96)	70·91 (68·26–73·23)	22·50 (22·42–22·58)	17·58 (16·12–18·83)	18·86 (18·79–18·92)	14·72 (13·49–15·77)
	Sweden		82·61 (82·50–82·73)	71·97 (68·89–74·70)	78·42 (78·30–78·54)	69·24 (66·58–71·58)	83·98 (83·74–84·25)	73·04 (69·93–75·82)	80·25 (80·01–80·48)	70·70 (67·98–73·16)	21·39 (21·22–21·59)	16·59 (15·24–17·78)	18·72 (18·57–18·86)	14·36 (13·13–15·47)
	Switzerland		83·72 (83·59–83·85)	72·15 (68·81–75·09)	78·67 (78·54–78·80)	69·23 (66·47–71·66)	85·18 (84·89–85·50)	73·55 (70·15–76·54)	80·65 (80·33–81·01)	71·16 (68·40–73·63)	22·52 (22·31–22·74)	17·40 (15·85–18·73)	19·25 (19·06–19·47)	14·99 (13·77–16·09)
	UK		81·14 (81·11–81·18)	70·63 (67·46–73·34)	76·73 (76·70–76·77)	67·86 (65·24–70·12)	82·81 (82·71–82·93)	72·09 (68·88–74·81)	79·03 (78·94–79·13)	69·86 (67·26–72·19)	20·85 (20·77–20·95)	16·41 (15·13–17·54)	18·35 (18·28–18·41)	14·38 (13·28–15·39)
	England		81·38 (81·34–81·41)	70·91 (67·74–73·60)	77·05 (77·02–77·09)	68·20 (65·59–70·47)	83·05 (82·94–83·16)	72·37 (69·18–75·11)	79·34 (79·24–79·44)	70·19 (67·61–72·53)	21·01 (20·92–21·10)	16·57 (15·29–17·71)	18·51 (18·44–18·58)	14·54 (13·43–15·54)
	Northern Ireland		80·78 (80·57–80·97)	70·40 (67·34–73·09)	75·84 (75·64–76·04)	67·11 (64·54–69·40)	82·30 (81·91–82·68)	71·74 (68·69–74·38)	77·94 (77·50–78·37)	68·98 (66·37–71·31)	20·55 (20·27–20·82)	16·17 (14·89–17·30)	17·67 (17·45–17·89)	13·82 (12·71–14·82)
	Scotland		79·48 (79·35–79·61)	68·60 (65·44–71·42)	74·35 (74·23–74·47)	65·43 (62·83–67·70)	81·22 (80·86–81·62)	70·03 (66·77–72·90)	77·15 (76·79–77·50)	67·83 (65·20–70·28)	19·81 (19·57–20·06)	15·35 (14·05–16·49)	17·46 (17·28–17·65)	13·54 (12·45–14·58)
	Wales		80·66 (80·53–80·79)	69·95 (66·78–72·73)	76·19 (76·06–76·31)	66·91 (64·19–69·30)	82·16 (81·86–82·46)	71·20 (67·94–74·08)	77·98 (77·69–78·28)	68·39 (65·59–70·88)	20·38 (20·18–20·58)	15·88 (14·57–17·06)	17·63 (17·47–17·79)	13·62 (12·50–14·65)
Southern Latin America			79·29 (79·23–79·34)	69·60 (66·74–72·09)	72·64 (72·58–72·69)	64·83 (62·54–66·87)	80·41 (80·19–80·63)	70·54 (67·61–73·11)	73·98 (73·74–74·23)	66·02 (63·62–68·10)	19·81 (19·67–19·94)	15·78 (14·59–16·82)	15·83 (15·71–15·95)	12·47 (11·47–13·33)
	Argentina		78·69 (78·61–78·76)	69·07 (66·24–71·55)	71·78 (71·71–71·85)	64·04 (61·79–66·07)	79·76 (79·51–80·01)	70·00 (67·06–72·53)	73·05 (72·77–73·31)	65·20 (62·88–67·28)	19·55 (19·40–19·69)	15·58 (14·41–16·62)	15·37 (15·23–15·50)	12·10 (11·12–12·94)
	Chile		80·97 (80·87–81·08)	71·07 (68·14–73·59)	75·36 (75·26–75·47)	67·21 (64·82–69·31)	82·04 (81·53–82·54)	71·91 (69·01–74·53)	76·56 (75·93–77·19)	68·28 (65·76–70·51)	20·50 (20·15–20·84)	16·30 (15·06–17·39)	17·22 (16·88–17·57)	13·59 (12·47–14·60)
	Uruguay		79·15 (78·98–79·31)	69·51 (66·62–71·93)	71·80 (71·65–71·96)	64·33 (62·09–66·28)	80·51 (80·15–80·90)	70·65 (67·77–73·25)	72·93 (72·50–73·37)	65·30 (62·97–67·25)	19·85 (19·65–20·05)	15·80 (14·63–16·86)	15·05 (14·85–15·24)	11·86 (10·90–12·68)
Central Europe, eastern Europe, and central Asia			73·64 (73·53–73·75)	64·25 (61·48–66·73)	62·93 (62·80–63·06)	55·92 (53·84–57·72)	77·31 (77·09–77·50)	67·31 (64·36–69·91)	67·95 (67·71–68·21)	60·13 (57·76–62·14)	17·86 (17·77–17·94)	13·74 (12·56–14·77)	13·97 (13·90–14·04)	10·57 (9·60–11·43)
	Eastern Europe		72·72 (72·63–72·80)	63·16 (60·37–65·68)	59·78 (59·65–59·91)	53·18 (51·24–54·89)	76·70 (76·44–76·94)	66·44 (63·45–69·08)	65·73 (65·39–66·05)	58·15 (55·84–60·13)	17·40 (17·27–17·52)	13·30 (12·12–14·33)	13·10 (12·98–13·22)	9·84 (8·90–10·68)
		Belarus	74·89 (74·62–75·15)	65·58 (62·91–68·05)	62·76 (62·41–63·09)	55·92 (53·92–57·70)	77·07 (76·58–77·58)	67·38 (64·53–69·96)	65·02 (64·33–65·74)	57·85 (55·63–59·94)	17·05 (16·77–17·32)	13·19 (12·06–14·23)	11·75 (11·47–12·04)	8·86 (8·00–9·68)
		Estonia	77·67 (77·51–77·84)	67·79 (64·90–70·36)	67·12 (66·93–67·29)	59·50 (57·27–61·47)	81·43 (80·99–81·88)	71·08 (68·17–73·78)	73·44 (73·07–73·83)	64·89 (62·39–67·14)	19·70 (19·35–20·07)	15·32 (14·07–16·50)	15·61 (15·39–15·85)	11·87 (10·77–12·85)
		Latvia	76·63 (76·48–76·78)	66·98 (64·16–69·53)	65·47 (65·32–65·62)	58·12 (55·95–60·04)	79·73 (79·34–80·06)	69·63 (66·64–72·23)	70·59 (70·17–70·99)	62·48 (60·12–64·65)	19·01 (18·79–19·20)	14·70 (13·42–15·80)	14·55 (14·37–14·72)	10·99 (9·97–11·90)
		Lithuania	77·71 (77·60–77·82)	67·74 (64·85–70·37)	65·71 (65·60–65·82)	58·22 (55·95–60·15)	80·39 (80·16–80·61)	70·02 (67·03–72·76)	69·72 (69·47–70·02)	61·55 (59·11–63·70)	19·63 (19·49–19·76)	15·11 (13·80–16·26)	14·75 (14·64–14·87)	10·97 (9·87–11·94)
		Moldova	73·43 (73·13–73·69)	64·40 (61·73–66·76)	65·55 (65·21–65·82)	58·37 (56·23–60·27)	77·70 (77·29–78·06)	67·96 (65·13–70·54)	69·93 (69·47–70·35)	62·06 (59·81–64·18)	17·39 (17·21–17·58)	13·53 (12·39–14·53)	14·04 (13·91–14·18)	10·71 (9·76–11·57)
		Russia	72·21 (72·11–72·30)	62·43 (59·56–64·98)	58·71 (58·54–58·87)	52·21 (50·31–53·88)	76·57 (76·22–76·90)	66·02 (62·89–68·71)	65·30 (64·85–65·77)	57·71 (55·43–59·76)	17·48 (17·29–17·64)	13·26 (12·03–14·31)	13·12 (12·95–13·30)	9·83 (8·86–10·68)
		Ukraine	73·16 (72·91–73·37)	64·21 (61·62–66·53)	61·36 (61·08–61·60)	54·64 (52·67–56·37)	76·45 (75·94–76·93)	66·93 (64·21–69·44)	66·26 (65·56–66·93)	58·71 (56·38–60·69)	16·95 (16·73–17·18)	13·15 (12·05–14·14)	12·97 (12·77–13·18)	9·82 (8·90–10·63)
	Central Europe		77·90 (77·86–77·94)	68·36 (65·55–70·83)	70·48 (70·44–70·52)	62·43 (60·05–64·51)	80·45 (80·31–80·58)	70·52 (67·59–73·06)	73·43 (73·25–73·59)	64·92 (62·42–67·13)	19·05 (18·98–19·11)	14·80 (13·54–15·90)	15·33 (15·26–15·39)	11·65 (10·59–12·59)
		Albania	79·08 (78·63–79·52)	69·12 (66·18–71·74)	73·49 (72·96–74·00)	64·65 (62·01–66·95)	81·32 (80·71–81·95)	71·17 (68·10–73·74)	74·98 (74·10–75·87)	66·20 (63·43–68·64)	20·06 (19·65–20·47)	15·65 (14·28–16·89)	16·09 (15·36–16·99)	12·34 (11·10–13·63)
		Bosnia and Herzegovina	79·89 (79·75–80·05)	69·96 (67·03–72·56)	74·05 (73·91–74·23)	65·08 (62·52–67·36)	81·82 (81·45–82·21)	71·51 (68·53–74·24)	76·00 (75·72–76·30)	66·69 (64·02–69·04)	20·09 (19·81–20·38)	15·56 (14·25–16·76)	16·64 (16·51–16·81)	12·59 (11·45–13·61)
		Bulgaria	76·22 (76·11–76·34)	67·07 (64·38–69·46)	69·07 (68·95–69·17)	61·35 (59·08–63·34)	78·36 (77·90–78·80)	68·88 (66·09–71·34)	71·37 (70·84–71·87)	63·34 (60·91–65·43)	17·54 (17·26–17·79)	13·67 (12·53–14·68)	14·14 (13·91–14·37)	10·78 (9·80–11·69)
		Croatia	79·21 (79·07–79·35)	69·34 (66·46–71·92)	72·01 (71·89–72·12)	63·67 (61·24–65·84)	80·92 (80·61–81·26)	70·74 (67·69–73·39)	74·65 (74·31–75·00)	65·87 (63·30–68·12)	18·92 (18·71–19·15)	14·64 (13·35–15·72)	15·39 (15·20–15·58)	11·70 (10·64–12·64)
		Czech Republic	79·37 (79·28–79·47)	69·51 (66·58–72·10)	72·98 (72·90–73·06)	64·55 (62·06–66·73)	81·65 (81·47–81·83)	71·41 (68·37–74·06)	75·92 (75·73–76·11)	66·94 (64·35–69·27)	19·46 (19·33–19·59)	15·05 (13·78–16·19)	16·06 (15·96–16·17)	12·13 (11·00–13·14)
		Hungary	77·25 (77·14–77·34)	67·48 (64·65–70·00)	68·82 (68·73–68·91)	61·06 (58·81–63·06)	79·95 (79·55–80·34)	69·76 (66·77–72·32)	73·22 (72·78–73·68)	64·71 (62·24–66·94)	19·00 (18·75–19·23)	14·59 (13·27–15·70)	15·41 (15·20–15·63)	11·65 (10·56–12·61)
		Macedonia	76·41 (76·25–76·57)	67·48 (64·89–69·82)	71·36 (71·20–71·51)	63·45 (61·07–65·54)	79·14 (78·63–79·65)	69·66 (66·84–72·23)	73·92 (73·30–74·51)	65·54 (63·08–67·75)	17·94 (17·65–18·28)	13·95 (12·78–15·05)	15·06 (14·67–15·47)	11·50 (10·47–12·50)
		Montenegro	77·96 (77·70–78·22)	68·56 (65·82–71·10)	71·99 (71·77–72·22)	63·70 (61·25–65·88)	80·18 (79·69–80·68)	70·44 (67·53–73·02)	74·21 (73·61–74·86)	65·59 (63·03–67·89)	18·94 (18·63–19·25)	14·76 (13·53–15·90)	15·64 (15·26–16·09)	11·95 (10·83–12·96)
		Poland	79·36 (79·31–79·41)	69·57 (66·70–72·07)	70·78 (70·73–70·83)	62·67 (60·24–64·74)	81·61 (81·39–81·81)	71·53 (68·56–74·11)	73·45 (73·23–73·69)	64·96 (62·44–67·15)	19·98 (19·86–20·10)	15·51 (14·22–16·67)	15·56 (15·46–15·66)	11·82 (10·74–12·79)
		Romania	76·08 (76·00–76·17)	66·92 (64·24–69·30)	68·82 (68·75–68·90)	61·04 (58·74–63·06)	78·99 (78·59–79·36)	69·44 (66·68–71·90)	71·52 (71·03–72·00)	63·40 (60·98–65·54)	18·15 (17·92–18·37)	14·25 (13·12–15·26)	14·55 (14·34–14·77)	11·14 (10·15–12·04)
		Serbia	75·94 (75·71–76·15)	66·88 (64·26–69·26)	70·58 (70·36–70·80)	62·59 (60·22–64·67)	78·87 (78·69–79·05)	69·23 (66·44–71·75)	73·48 (73·20–73·71)	64·90 (62·35–67·11)	17·88 (17·77–17·99)	13·85 (12·68–14·89)	15·18 (15·02–15·31)	11·52 (10·46–12·47)
		Slovakia	78·18 (78·06–78·30)	68·72 (65·92–71·18)	70·28 (70·16–70·39)	62·14 (59·72–64·26)	80·92 (80·69–81·14)	70·92 (68·01–73·52)	73·98 (73·74–74·21)	65·21 (62·65–67·47)	19·18 (19·03–19·33)	14·90 (13·67–16·05)	15·38 (15·26–15·49)	11·60 (10·53–12·55)
		Slovenia	81·07 (80·92–81·20)	70·80 (67·77–73·46)	73·89 (73·76–74·01)	65·25 (62·75–67·47)	83·84 (83·62–84·05)	73·21 (70·12–75·94)	77·94 (77·71–78·18)	68·66 (65·91–71·09)	21·31 (21·16–21·47)	16·54 (15·13–17·77)	17·55 (17·42–17·68)	13·39 (12·17–14·47)
	Central Asia		71·47 (71·12–71·80)	62·79 (60·19–65·08)	63·62 (63·27–63·98)	56·73 (54·69–58·52)	75·30 (74·77–75·76)	65·96 (63·27–68·44)	67·41 (66·85–67·91)	59·93 (57·71–61·94)	16·95 (16·75–17·17)	13·14 (12·03–14·15)	13·48 (13·32–13·66)	10·35 (9·44–11·16)
		Armenia	75·32 (74·82–75·77)	65·81 (63·07–68·24)	68·18 (67·64–68·75)	60·65 (58·47–62·68)	78·37 (77·89–78·95)	68·40 (65·51–70·99)	70·75 (70·05–71·47)	62·84 (60·47–65·01)	18·06 (17·85–18·44)	13·83 (12·63–14·95)	14·37 (14·13–14·66)	10·99 (9·99–11·87)
		Azerbaijan	71·39 (70·61–72·13)	62·63 (59·91–65·16)	65·10 (64·27–65·92)	58·10 (55·95–60·12)	76·32 (75·59–77·07)	66·67 (63·73–69·41)	69·57 (68·51–70·58)	61·78 (59·33–63·98)	17·57 (17·25–17·90)	13·56 (12·32–14·64)	14·46 (13·77–14·96)	11·12 (10·05–12·10)
		Georgia	76·02 (75·39–76·63)	67·04 (64·39–69·40)	67·40 (66·60–68·21)	60·26 (58·09–62·26)	78·04 (77·27–78·74)	68·48 (65·63–71·10)	67·86 (66·87–69·05)	60·52 (58·23–62·66)	18·13 (17·73–18·55)	14·12 (12·89–15·23)	13·23 (12·89–13·73)	10·15 (9·21–11·06)
		Kazakhstan	71·06 (70·70–71·40)	62·23 (59·70–64·57)	59·79 (59·41–60·14)	53·24 (51·28–54·98)	75·03 (74·05–75·95)	65·52 (62·54–68·31)	65·29 (64·11–66·49)	57·91 (55·58–60·11)	16·49 (16·00–16·99)	12·70 (11·53–13·82)	12·66 (12·21–13·13)	9·60 (8·68–10·47)
		Kyrgyzstan	70·68 (70·16–71·20)	62·06 (59·52–64·39)	62·56 (61·96–63·23)	55·92 (53·99–57·76)	74·10 (73·44–74·71)	64·92 (62·25–67·31)	65·60 (64·83–66·41)	58·62 (56·46–60·57)	16·13 (15·81–16·47)	12·55 (11·47–13·49)	12·64 (12·31–12·98)	9·79 (8·91–10·58)
		Mongolia	67·72 (67·00–68·42)	60·10 (57·87–62·07)	59·95 (59·16–60·71)	53·54 (51·58–55·43)	71·87 (71·03–72·67)	63·55 (61·09–65·93)	62·78 (61·90–63·63)	55·92 (53·79–57·96)	15·12 (14·80–15·43)	11·73 (10·76–12·66)	11·78 (11·55–12·05)	8·94 (8·13–9·73)
		Tajikistan	70·16 (69·42–70·88)	61·79 (59·20–64·06)	65·92 (65·24–66·65)	58·68 (56·48–60·63)	74·80 (73·94–75·53)	65·67 (63·00–68·18)	70·12 (69·20–70·96)	62·17 (59·71–64·38)	16·91 (16·48–17·22)	13·20 (12·13–14·21)	15·04 (14·71–15·31)	11·61 (10·61–12·52)
		Turkmenistan	69·51 (68·21–70·74)	61·31 (58·75–63·67)	62·12 (60·77–63·27)	55·49 (53·35–57·52)	73·69 (72·71–74·46)	64·83 (62·09–67·28)	66·33 (65·35–67·15)	59·11 (56·86–61·15)	16·78 (16·53–17·01)	13·09 (11·99–14·06)	13·57 (13·32–13·79)	10·47 (9·58–11·31)
		Uzbekistan	71·50 (70·65–72·29)	62·93 (60·29–65·29)	65·48 (64·61–66·40)	58·39 (56·15–60·45)	75·20 (74·34–76·03)	66·03 (63·32–68·49)	68·37 (67·51–69·27)	60·85 (58·49–63·00)	16·77 (16·40–17·33)	13·10 (11·98–14·12)	13·69 (13·42–14·00)	10·59 (9·67–11·42)
Latin America and Caribbean			76·49 (76·28–76·69)	66·42 (63·49–69·02)	70·45 (70·24–70·68)	62·25 (59·82–64·38)	78·25 (77·87–78·62)	67·88 (64·80–70·50)	72·20 (71·73–72·62)	63·76 (61·28–65·91)	18·85 (18·69–19·02)	14·38 (13·08–15·51)	16·25 (16·11–16·40)	12·45 (11·37–13·42)
	Central Latin America		77·58 (77·40–77·75)	67·68 (64·81–70·20)	71·92 (71·72–72·11)	63·73 (61·30–65·83)	78·81 (78·44–79·15)	68·69 (65·67–71·26)	73·28 (72·84–73·72)	64·88 (62·36–67·07)	19·06 (18·95–19·19)	14·51 (13·21–15·68)	16·97 (16·86–17·09)	13·05 (11·91–14·05)
		Colombia	78·26 (78·06–78·46)	68·20 (65·23–70·78)	72·02 (71·80–72·23)	64·05 (61·65–66·08)	80·80 (80·35–81·25)	70·21 (67·06–72·90)	75·15 (74·58–75·65)	66·65 (64·15–68·88)	20·06 (19·85–20·28)	15·33 (13·95–16·52)	17·46 (17·28–17·64)	13·55 (12·44–14·57)
		Costa Rica	81·47 (81·22–81·69)	70·96 (67·85–73·65)	76·56 (76·27–76·80)	67·84 (65·19–70·12)	82·60 (82·12–83·05)	71·84 (68·61–74·60)	78·08 (77·50–78·58)	69·11 (66·46–71·44)	21·24 (20·99–21·51)	16·24 (14·85–17·48)	18·90 (18·66–19·12)	14·67 (13·44–15·77)
		El Salvador	77·02 (76·56–77·45)	66·63 (63·59–69·35)	68·79 (68·07–69·49)	60·84 (58·45–62·88)	78·94 (78·06–79·85)	68·24 (64·99–71·16)	70·57 (69·32–71·94)	62·56 (60·03–64·95)	19·55 (19·10–20·06)	14·80 (13·36–16·08)	17·79 (17·33–18·24)	13·74 (12·56–14·87)
		Guatemala	73·16 (72·79–73·54)	63·27 (60·37–65·90)	66·41 (66·08–66·75)	58·31 (55·91–60·44)	75·20 (73·54–77·05)	65·09 (61·95–68·07)	69·81 (67·75–71·79)	61·33 (58·45–64·12)	17·38 (16·41–18·60)	13·02 (11·59–14·44)	17·18 (16·45–17·95)	13·09 (11·85–14·29)
		Honduras	72·00 (69·46–74·83)	63·00 (59·75–66·13)	70·45 (67·22–72·91)	62·58 (59·11–65·60)	73·95 (71·41–76·68)	64·71 (61·55–68·01)	72·09 (68·69–74·71)	64·07 (60·73–67·10)	16·30 (15·18–17·86)	12·48 (11·19–13·99)	16·08 (14·54–17·25)	12·49 (11·04–13·84)
		Mexico	77·72 (77·45–77·95)	68·16 (65·42–70·62)	72·54 (72·25–72·81)	64·56 (62·22–66·64)	78·32 (77·98–78·60)	68·64 (65·82–71·11)	73·43 (73·04–73·75)	65·26 (62·82–67·38)	18·65 (18·59–18·70)	14·24 (12·98–15·37)	16·78 (16·74–16·83)	12·91 (11·78–13·91)
		Nicaragua	79·26 (78·69–79·76)	69·03 (65·97–71·74)	73·74 (73·07–74·37)	65·25 (62·65–67·57)	80·76 (79·93–81·68)	70·31 (67·22–73·07)	75·01 (73·80–76·26)	66·47 (63·72–69·01)	20·76 (20·25–21·30)	15·88 (14·42–17·17)	18·30 (17·75–18·85)	14·19 (12·90–15·41)
		Panama	79·72 (79·09–80·35)	69·27 (66·25–72·03)	74·77 (73·98–75·48)	66·08 (63·42–68·38)	81·01 (79·82–82·22)	70·21 (66·90–73·23)	75·48 (73·89–76·95)	66·63 (63·75–69·40)	20·93 (20·19–21·70)	15·90 (14·35–17·37)	18·34 (17·55–19·16)	14·11 (12·76–15·39)
		Venezuela	78·22 (78·11–78·32)	67·39 (64·28–70·19)	71·17 (71·06–71·27)	61·93 (59·15–64·31)	79·16 (78·00–80·36)	68·14 (64·68–71·24)	70·69 (69·02–72·41)	61·62 (58·62–64·44)	19·37 (18·64–20·13)	14·58 (13·09–15·96)	16·05 (15·37–16·77)	12·05 (10·85–13·25)
	Andean Latin America		76·76 (76·30–77·23)	66·92 (64·06–69·48)	73·09 (72·59–73·57)	64·22 (61·57–66·57)	79·01 (78·22–79·82)	68·86 (65·87–71·66)	75·40 (74·51–76·28)	66·26 (63·43–68·85)	19·24 (18·74–19·76)	14·90 (13·63–16·14)	17·61 (17·15–18·06)	13·44 (12·14–14·60)
		Bolivia	71·40 (69·86–72·67)	62·29 (59·64–64·88)	69·02 (67·91–70·15)	60·74 (58·14–63·17)	74·22 (72·07–76·43)	64·65 (61·20–67·81)	72·11 (69·61–74·23)	63·35 (60·22–66·40)	16·64 (15·46–18·07)	12·80 (11·36–14·26)	16·33 (15·07–17·17)	12·41 (11·10–13·67)
		Ecuador	76·85 (76·33–77·36)	66·94 (63·96–69·56)	71·53 (70·79–72·19)	62·95 (60·31–65·29)	78·56 (77·59–79·50)	68·39 (65·41–71·20)	73·35 (72·06–74·39)	64·57 (61·56–67·16)	18·94 (18·40–19·46)	14·57 (13·33–15·81)	16·49 (15·80–17·02)	12·48 (11·14–13·66)
		Peru	78·80 (78·19–79·39)	68·71 (65·77–71·33)	75·55 (74·73–76·36)	66·30 (63·53–68·76)	81·10 (80·01–82·23)	70·74 (67·67–73·66)	77·85 (76·38–79·13)	68·37 (65·44–71·13)	20·44 (19·71–21·22)	15·93 (14·52–17·30)	18·78 (18·02–19·53)	14·42 (13·06–15·70)
Caribbean			72·72 (71·92–73·44)	63·17 (60·25–65·76)	69·08 (68·51–69·68)	60·88 (58·42–63·12)	75·21 (73·88–76·31)	65·16 (61·96–67·91)	70·92 (69·92–71·88)	62·43 (59·83–64·66)	18·22 (17·84–18·59)	14·04 (12·77–15·10)	16·18 (15·97–16·43)	12·47 (11·39–13·43)
		Antigua and Barbuda	77·20 (76·51–78·00)	67·21 (64·20–69·87)	72·45 (71·58–73·41)	63·69 (61·07–66·03)	78·85 (78·10–79·78)	68·54 (65·44–71·37)	73·78 (72·68–75·33)	64·92 (62·16–67·47)	18·43 (18·03–19·10)	14·28 (13·02–15·46)	15·57 (14·73–16·84)	11·90 (10·54–13·37)
		The Bahamas	74·43 (73·70–75·22)	64·60 (61·75–67·14)	68·91 (67·92–69·86)	60·65 (58·19–62·97)	76·41 (75·13–77·73)	66·24 (63·14–69·13)	70·87 (69·42–72·31)	62·47 (59·69–65·04)	17·58 (16·97–18·24)	13·49 (12·26–14·70)	15·42 (14·86–16·14)	11·79 (10·61–12·96)
		Barbados	76·96 (76·19–77·73)	66·70 (63·63–69·54)	72·90 (72·17–73·53)	64·11 (61·48–66·54)	77·56 (76·24–78·67)	67·12 (63·92–69·93)	73·63 (72·28–74·85)	64·84 (62·02–67·46)	18·05 (17·34–18·64)	13·81 (12·54–15·06)	16·09 (15·12–16·78)	12·29 (10·96–13·53)
		Belize	73·08 (72·37–73·77)	63·66 (60·85–66·10)	67·27 (66·44–68·11)	59·58 (57·17–61·66)	74·73 (73·67–75·77)	65·00 (62·11–67·76)	69·05 (67·70–70·29)	61·18 (58·61–63·64)	16·75 (16·23–17·30)	12·97 (11·80–14·04)	14·44 (13·90–15·11)	11·19 (10·17–12·20)
		Bermuda	79·60 (79·07–80·17)	69·18 (66·21–72·01)	73·35 (72·73–74·01)	64·74 (62·22–67·06)	82·23 (81·24–83·29)	71·28 (68·14–74·12)	75·00 (73·93–75·97)	66·15 (63·61–68·57)	20·38 (19·75–21·11)	15·80 (14·45–17·03)	16·27 (15·84–16·62)	12·53 (11·43–13·52)
		Cuba	79·14 (79·04–79·22)	68·94 (65·93–71·55)	75·19 (75·09–75·27)	66·68 (64·14–68·92)	80·60 (80·24–80·97)	70·06 (66·98–72·80)	75·87 (75·52–76·25)	67·22 (64·76–69·45)	19·56 (19·34–19·79)	15·16 (13·88–16·31)	16·68 (16·50–16·87)	13·01 (11·96–13·95)
		Dominica	77·15 (76·26–77·93)	66·87 (63·91–69·60)	71·36 (70·25–72·27)	62·42 (59·76–64·84)	76·81 (75·46–78·06)	66·52 (63·28–69·40)	69·71 (67·83–71·50)	61·07 (58·09–63·74)	18·29 (17·55–18·86)	14·04 (12·66–15·26)	14·93 (14·01–15·94)	11·25 (9·97–12·50)
		Dominican Republic	76·16 (75·51–76·65)	66·32 (63·46–68·91)	70·75 (70·04–71·40)	62·43 (59·94–64·68)	77·93 (77·41–78·30)	67·89 (64·83–70·38)	72·82 (71·86–73·45)	64·36 (61·78–66·67)	19·04 (18·91–19·11)	14·78 (13·55–15·85)	16·79 (16·51–16·90)	12·99 (11·86–13·96)
		Grenada	73·36 (72·61–74·08)	63·76 (60·97–66·16)	67·21 (66·38–67·95)	59·21 (56·81–61·43)	74·28 (72·93–75·36)	64·40 (61·31–67·09)	68·78 (67·50–70·02)	60·56 (57·89–62·93)	16·30 (15·74–16·82)	12·50 (11·33–13·54)	13·99 (13·53–14·41)	10·61 (9·51–11·52)
		Guyana	67·83 (66·96–68·68)	58·96 (56·30–61·42)	61·28 (60·22–62·33)	54·11 (51·88–56·20)	70·47 (68·95–71·86)	61·22 (58·26–64·03)	63·47 (61·70–65·26)	56·08 (53·43–58·62)	14·88 (14·21–15·47)	11·43 (10·35–12·47)	12·55 (12·03–13·14)	9·57 (8·67–10·50)
		Haiti	59·86 (57·33–62·32)	51·93 (48·83–54·97)	60·30 (58·18–62·80)	52·84 (50·10–55·64)	64·74 (61·22–68·20)	55·81 (51·95–59·24)	63·91 (60·58–67·20)	55·75 (52·32–59·08)	13·37 (11·94–15·07)	10·09 (8·77–11·46)	14·01 (12·88–15·32)	10·54 (9·30–11·84)
		Jamaica	76·72 (75·84–77·43)	66·45 (63·40–69·28)	73·96 (72·94–74·84)	64·93 (62·19–67·48)	76·97 (75·72–78·20)	66·55 (63·15–69·48)	73·03 (71·74–74·36)	64·18 (61·35–66·78)	18·00 (17·35–18·77)	13·85 (12·48–15·08)	16·43 (15·55–17·11)	12·65 (11·39–13·78)
		Puerto Rico	80·24 (80·07–80·41)	69·47 (66·33–72·22)	72·85 (72·67–73·00)	63·94 (61·31–66·24)	81·77 (81·16–82·37)	70·70 (67·45–73·61)	75·03 (74·35–75·73)	65·70 (62·92–68·26)	20·43 (20·03–20·83)	15·71 (14·34–16·94)	17·42 (17·11–17·72)	13·17 (11·95–14·31)
		Saint Lucia	76·14 (75·26–77·06)	66·13 (63·23–68·75)	70·19 (69·24–71·13)	61·74 (59·30–63·99)	77·09 (75·75–78·29)	66·91 (63·83–69·68)	72·52 (71·02–73·92)	63·78 (61·01–66·41)	17·82 (17·13–18·53)	13·74 (12·49–14·93)	16·08 (15·41–16·73)	12·35 (11·12–13·45)
		Saint Vincent and the Grenadines	73·00 (72·20–73·75)	63·48 (60·67–65·95)	68·03 (67·29–68·71)	59·90 (57·52–62·10)	73·95 (72·82–75·00)	64·26 (61·26–66·93)	68·77 (67·61–69·89)	60·59 (58·00–62·87)	16·11 (15·64–16·62)	12·39 (11·28–13·41)	14·09 (13·51–14·61)	10·71 (9·59–11·71)
		Suriname	72·74 (71·94–73·56)	63·29 (60·51–65·80)	67·08 (66·13–68·21)	59·30 (56·87–61·54)	75·34 (73·89–76·55)	65·50 (62·51–68·45)	68·82 (67·09–70·46)	60·87 (58·15–63·47)	17·81 (16·90–18·46)	13·79 (12·48–15·07)	14·97 (13·93–15·94)	11·60 (10·37–12·83)
		Trinidad and Tobago	74·55 (74·07–74·94)	64·63 (61·71–67·22)	67·83 (67·34–68·24)	59·72 (57·26–61·89)	76·11 (74·90–77·24)	65·86 (62·82–68·63)	68·88 (67·40–70·26)	60·56 (57·82–63·02)	17·70 (17·11–18·26)	13·58 (12·32–14·73)	14·01 (13·42–14·55)	10·64 (9·59–11·68)
		Virgin Islands	78·01 (77·51–78·52)	67·81 (64·87–70·40)	70·91 (70·26–71·53)	62·41 (59·95–64·67)	78·72 (77·88–79·47)	68·35 (65·40–71·18)	70·34 (68·90–71·64)	62·04 (59·52–64·54)	18·47 (18·11–18·79)	14·26 (13·03–15·36)	14·52 (13·95–15·13)	11·03 (10·04–12·07)
Tropical Latin America			76·21 (75·81–76·58)	65·73 (62·71–68·45)	68·77 (68·38–69·19)	60·66 (58·25–62·82)	78·18 (77·61–78·70)	67·36 (64·20–70·06)	70·75 (70·08–71·38)	62·38 (59·89–64·58)	18·72 (18·37–19·08)	14·21 (12·88–15·37)	15·33 (15·06–15·65)	11·70 (10·66–12·65)
		Brazil	76·21 (75·81–76·59)	65·73 (62·70–68·46)	68·69 (68·28–69·12)	60·58 (58·18–62·74)	78·21 (77·63–78·74)	67·38 (64·24–70·09)	70·72 (70·02–71·35)	62·35 (59·87–64·54)	18·74 (18·38–19·11)	14·23 (12·89–15·40)	15·32 (15·05–15·65)	11·70 (10·65–12·65)
		Paraguay	76·10 (75·16–77·01)	65·86 (62·81–68·66)	72·03 (71·11–72·86)	63·47 (60·86–65·87)	76·95 (75·54–78·38)	66·44 (63·14–69·64)	72·09 (70·20–73·79)	63·54 (60·38–66·19)	17·79 (17·02–18·72)	13·48 (12·10–14·92)	15·74 (14·84–16·55)	12·01 (10·72–13·22)
Southeast Asia, east Asia, and Oceania			73·95 (73·58–74·33)	65·33 (62·87–67·58)	68·52 (68·12–68·95)	61·40 (59·27–63·32)	78·38 (77·84–78·93)	69·11 (66·39–71·55)	71·90 (71·26–72·50)	64·36 (62·09–66·41)	18·48 (18·12–18·87)	14·53 (13·45–15·60)	14·95 (14·63–15·29)	11·86 (10·97–12·70)
	East Asia		74·94 (74·46–75·43)	66·54 (64·09–68·73)	69·38 (68·86–69·89)	62·44 (60·36–64·35)	79·83 (79·19–80·49)	70·71 (67·99–73·12)	73·17 (72·45–73·88)	65·74 (63·45–67·78)	18·99 (18·51–19·50)	15·07 (13·98–16·18)	15·13 (14·73–15·54)	12·09 (11·18–12·92)
		China	74·90 (74·41–75·40)	66·53 (64·05–68·73)	69·33 (68·82–69·86)	62·40 (60·32–64·31)	79·89 (79·24–80·56)	70·78 (68·07–73·23)	73·19 (72·44–73·91)	65·75 (63·43–67·77)	19·01 (18·52–19·57)	15·11 (14·00–16·22)	15·10 (14·68–15·50)	12·07 (11·16–12·90)
		North Korea	72·55 (69·82–75·12)	64·12 (60·50–67·12)	67·53 (64·35–71·01)	60·93 (57·56–64·20)	74·89 (72·19–77·52)	66·20 (62·62–69·50)	68·94 (65·72–71·93)	62·22 (58·78–65·27)	16·59 (15·35–17·90)	13·02 (11·60–14·42)	13·94 (12·91–15·25)	11·14 (10·00–12·41)
		Taiwan (province of China)	80·69 (80·57–80·81)	70·76 (67·85–73·30)	74·48 (74·38–74·60)	66·78 (64·52–68·76)	82·50 (81·18–83·87)	72·18 (68·93–75·14)	76·64 (75·07–78·27)	68·54 (65·66–71·16)	20·75 (19·81–21·71)	16·11 (14·63–17·56)	17·83 (17·04–18·69)	14·14 (12·85–15·40)
	Southeast Asia		72·16 (71·46–72·89)	62·99 (60·28–65·42)	67·08 (66·24–68·00)	59·39 (57·12–61·51)	75·32 (74·30–76·32)	65·77 (63·04–68·45)	69·18 (67·96–70·46)	61·37 (58·95–63·67)	17·10 (16·64–17·58)	13·05 (11·88–14·14)	14·32 (13·85–14·99)	11·02 (10·03–12·01)
		Cambodia	66·24 (65·34–67·27)	57·67 (55·12–60·05)	61·53 (60·48–62·59)	54·41 (52·18–56·48)	72·44 (70·47–74·65)	63·20 (60·14–66·32)	66·21 (64·31–68·00)	58·76 (56·24–61·37)	15·49 (14·72–16·72)	11·80 (10·60–13·12)	13·09 (12·36–13·71)	10·05 (9·12–11·01)
		Indonesia	70·11 (69·16–71·14)	61·40 (58·93–63·80)	67·35 (66·18–68·63)	59·63 (57·19–61·78)	73·29 (71·23–75·39)	64·06 (61·07–66·97)	69·01 (66·64–71·91)	61·21 (58·27–64·28)	15·80 (15·03–16·90)	11·97 (10·81–13·15)	14·17 (13·25–15·79)	10·88 (9·66–12·36)
		Laos	62·05 (60·33–63·86)	54·26 (51·70–56·65)	58·08 (56·59–59·47)	51·31 (49·04–53·42)	69·48 (66·71–72·17)	60·75 (57·56–63·91)	64·73 (62·17–67·51)	57·20 (54·14–60·31)	15·26 (13·95–16·63)	11·60 (10·20–13·00)	13·78 (12·78–14·71)	10·49 (9·37–11·60)
		Malaysia	76·30 (76·22–76·37)	66·76 (64·04–69·16)	72·35 (72·27–72·44)	64·08 (61·75–66·23)	78·62 (77·72–79·60)	68·60 (65·64–71·42)	72·39 (70·75–74·04)	64·21 (61·50–66·73)	17·89 (17·46–18·39)	13·71 (12·45–14·86)	15·16 (14·29–16·00)	11·79 (10·61–12·90)
		Maldives	78·22 (77·80–78·65)	67·35 (64·31–70·02)	75·73 (75·17–76·25)	66·24 (63·45–68·64)	82·26 (81·00–83·52)	71·20 (68·08–74·17)	78·06 (76·40–79·63)	68·79 (65·73–71·60)	20·80 (19·93–21·72)	15·90 (14·43–17·29)	18·29 (17·22–19·27)	14·17 (12·75–15·45)
		Mauritius	75·67 (75·40–75·92)	66·47 (63·85–68·76)	69·17 (68·91–69·42)	61·68 (59·50–63·58)	77·70 (77·09–78·26)	67·92 (65·14–70·41)	71·40 (70·82–72·07)	63·38 (60·98–65·51)	17·89 (17·56–18·24)	13·70 (12·52–14·77)	14·77 (14·50–15·10)	11·39 (10·39–12·27)
		Myanmar	66·02 (61·76–70·84)	57·67 (53·57–61·94)	60·74 (56·24–65·94)	53·78 (49·53–58·22)	71·20 (66·85–75·28)	62·24 (58·07–66·28)	64·92 (60·29–69·96)	57·56 (53·22–61·91)	15·05 (13·25–17·14)	11·36 (9·68–13·07)	12·90 (11·42–15·11)	9·82 (8·39–11·59)
		Philippines	73·03 (72·88–73·18)	63·05 (60·16–65·57)	65·43 (65·27–65·58)	57·69 (55·43–59·66)	74·41 (73·36–75·31)	64·43 (61·55–67·10)	67·71 (66·63–68·76)	59·82 (57·37–61·95)	16·55 (16·00–17·05)	12·50 (11·34–13·58)	13·19 (12·72–13·67)	10·03 (9·09–10·89)
		Sri Lanka	77·97 (77·72–78·23)	68·07 (65·20–70·58)	70·10 (69·85–70·36)	62·08 (59·74–64·13)	81·19 (79·06–83·24)	70·84 (67·31–73·92)	74·05 (71·09–76·93)	65·47 (61·81–68·87)	19·88 (18·47–21·33)	15·31 (13·60–16·89)	16·64 (15·29–18·11)	12·85 (11·28–14·39)
		Seychelles	75·88 (75·41–76·46)	66·68 (64·02–69·12)	68·23 (67·63–68·81)	60·74 (58·52–62·75)	77·93 (76·78–78·97)	68·29 (65·52–70·95)	70·55 (68·82–72·14)	62·77 (60·08–65·25)	17·98 (17·48–18·44)	13·81 (12·63–14·94)	14·50 (13·82–15·26)	11·13 (10·05–12·20)
		Thailand	76·50 (75·95–77·06)	66·91 (64·21–69·39)	69·77 (68·82–70·76)	61·78 (59·27–64·01)	78·98 (77·47–80·46)	69·11 (66·04–72·23)	70·81 (68·74–73·24)	62·92 (60·29–65·67)	18·77 (18·08–19·63)	14·48 (13·16–15·80)	14·97 (14·25–16·20)	11·59 (10·51–12·79)
		Timor-Leste	68·04 (66·22–69·89)	59·73 (57·05–62·44)	66·97 (65·35–68·64)	58·74 (55·92–61·30)	72·96 (70·01–75·75)	64·02 (60·49–67·35)	71·98 (68·85–75·12)	63·08 (59·32–66·79)	16·40 (14·96–17·76)	12·60 (11·07–14·05)	16·61 (15·28–18·13)	12·61 (11·10–14·17)
		Vietnam	76·96 (74·35–79·23)	67·23 (63·76–70·40)	69·45 (66·39–72·95)	61·89 (58·50–65·51)	79·38 (77·02–82·25)	69·76 (66·41–73·20)	71·31 (67·83–74·84)	63·80 (60·25–67·14)	18·83 (17·64–20·57)	14·65 (13·11–16·41)	14·75 (13·69–16·34)	11·58 (10·33–13·06)
	Oceania		60·94 (56·86–65·01)	52·15 (48·22–56·06)	59·03 (55·23–63·02)	52·11 (48·32–55·82)	63·72 (58·96–67·73)	54·48 (49·99–58·41)	61·42 (57·02–65·32)	54·24 (50·11–57·86)	11·91 (10·33–13·38)	8·73 (7·39–10·01)	11·74 (10·65–12·99)	8·86 (7·77–10·03)
		American Samoa	74·63 (72·98–75·91)	64·66 (61·63–67·36)	69·55 (67·93–71·11)	61·14 (58·40–63·76)	74·85 (72·39–77·07)	64·86 (61·72–68·10)	70·87 (68·59–73·17)	62·33 (59·32–65·44)	16·17 (14·98–17·31)	12·17 (10·85–13·56)	14·46 (13·64–15·44)	10·94 (9·80–12·13)
		Federated States of Micronesia	69·93 (64·32–74·51)	61·17 (55·63–65·47)	65·36 (60·00–70·18)	58·15 (52·88–62·29)	71·54 (65·30–75·82)	62·52 (56·78–66·79)	66·52 (60·42–70·80)	59·17 (53·52–63·30)	14·60 (11·87–16·79)	11·12 (8·75–13·02)	12·96 (10·83–14·41)	9·94 (8·07–11·30)
		Fiji	67·13 (65·42–68·78)	58·07 (55·19–60·77)	62·69 (60·85–64·52)	55·18 (52·48–57·81)	68·13 (65·45–70·87)	58·97 (55·62–62·18)	63·69 (60·77–66·48)	56·19 (53·06–59·16)	13·44 (12·28–14·61)	10·05 (8·82–11·33)	11·87 (10·99–12·73)	8·97 (7·99–10·00)
		Guam	76·52 (75·37–77·64)	65·90 (62·55–68·94)	70·07 (68·71–71·26)	62·30 (60·02–64·56)	75·31 (73·53–77·29)	64·87 (61·61–67·99)	68·49 (66·20–70·79)	60·87 (58·19–63·57)	16·69 (15·95–17·72)	12·55 (11·35–13·79)	13·65 (12·85–14·37)	10·52 (9·51–11·52)
		Kiribati	64·94 (62·65–67·10)	56·02 (52·94–58·91)	56·94 (54·59–59·28)	50·14 (47·27–52·89)	67·44 (64·55–70·21)	58·34 (54·91–61·62)	58·85 (55·69–61·78)	52·04 (48·74–55·23)	13·39 (12·31–14·42)	10·04 (8·86–11·15)	10·74 (9·94–11·52)	8·13 (7·19–9·08)
		Marshall Islands	66·83 (64·59–68·99)	57·26 (54·03–60·39)	62·73 (60·79–64·89)	54·83 (52·07–57·66)	69·50 (66·96–71·91)	59·42 (55·77–62·90)	64·57 (61·96–67·29)	56·37 (53·19–59·53)	13·79 (12·65–14·83)	10·12 (8·77–11·43)	12·42 (11·59–13·18)	9·23 (8·14–10·25)
		Northern Mariana Islands	77·39 (76·65–78·14)	67·32 (64·34–69·95)	74·55 (73·60–75·36)	65·91 (63·22–68·16)	78·47 (77·15–79·64)	68·26 (65·13–71·21)	74·17 (72·71–75·49)	65·64 (62·87–68·11)	18·21 (17·55–18·80)	13·88 (12·59–15·11)	15·92 (15·26–16·49)	12·28 (11·15–13·36)
		Papua New Guinea	58·54 (53·42–63·88)	49·82 (45·14–54·61)	57·25 (52·39–62·71)	50·55 (45·95–55·57)	61·85 (55·97–67·04)	52·62 (47·33–57·26)	60·13 (54·55–65·30)	53·11 (47·99–57·67)	11·07 (9·04–13·12)	8·02 (6·41–9·61)	11·34 (9·73–13·22)	8·52 (7·14–10·11)
		Samoa	73·70 (71·66–75·83)	64·10 (60·84–67·21)	70·00 (67·91–71·97)	61·81 (58·87–64·79)	74·22 (71·79–76·62)	64·45 (60·99–67·91)	72·23 (69·74–74·55)	63·58 (60·54–66·60)	15·96 (14·76–17·15)	12·06 (10·65–13·56)	15·02 (14·00–16·06)	11·45 (10·19–12·72)
		Solomon Islands	62·69 (57·49–68·21)	54·94 (50·49–59·82)	60·93 (56·04–65·96)	54·44 (50·20–58·93)	65·12 (59·19–70·81)	56·98 (51·74–61·79)	62·86 (56·96–68·13)	56·07 (51·03–60·69)	11·78 (9·54–14·16)	8·95 (7·07–10·81)	11·83 (9·87–13·36)	9·10 (7·53–10·52)
		Tonga	72·70 (71·27–73·99)	62·79 (59·60–65·53)	67·29 (65·66–68·82)	59·53 (56·92–61·95)	74·68 (72·19–77·07)	64·43 (60·83–67·76)	68·40 (65·44–71·10)	60·50 (57·30–63·51)	16·40 (15·33–17·46)	12·25 (10·86–13·62)	13·64 (12·69–14·66)	10·39 (9·31–11·55)
		Vanuatu	64·21 (59·00–69·19)	56·64 (51·85–61·42)	61·37 (56·42–66·39)	54·95 (50·36–59·62)	66·64 (61·23–70·86)	58·58 (53·61–62·91)	63·22 (58·12–67·35)	56·41 (51·72–60·55)	12·72 (10·52–14·57)	9·79 (7·92–11·44)	12·03 (10·43–13·29)	9·32 (7·90–10·69)
North Africa and Middle East			72·55 (71·85–73·21)	62·33 (59·42–65·02)	68·64 (67·98–69·34)	59·98 (57·44–62·33)	74·94 (74·12–75·69)	64·29 (61·31–67·14)	70·07 (69·16–70·94)	61·16 (58·55–63·62)	17·58 (17·28–17·87)	13·07 (11·80–14·25)	15·37 (15·08–15·65)	11·55 (10·45–12·57)
		Afghanistan	51·40 (47·39–55·64)	44·45 (40·62–48·10)	52·67 (48·93–56·74)	45·55 (42·13–49·21)	54·46 (50·19–58·82)	47·11 (43·28–51·24)	53·34 (49·22–57·70)	46·27 (42·42–50·19)	9·41 (8·21–10·93)	6·91 (5·82–8·22)	10·57 (9·49–12·37)	7·68 (6·52–9·09)
		Algeria	76·07 (74·93–77·12)	65·55 (62·46–68·52)	73·51 (72·29–74·79)	64·39 (61·45–67·02)	77·44 (76·01–78·52)	66·66 (63·44–69·65)	75·37 (74·13–76·79)	65·91 (63·15–68·58)	18·29 (17·47–18·76)	13·73 (12·34–15·01)	17·48 (16·90–18·23)	13·26 (11·99–14·47)
		Bahrain	78·06 (77·24–78·93)	66·10 (62·55–69·30)	74·63 (73·80–75·59)	64·92 (62·04–67·48)	80·34 (78·92–81·83)	67·98 (64·36–71·35)	78·21 (76·45–79·99)	67·89 (64·67–71·12)	19·23 (18·60–20·06)	14·00 (12·51–15·46)	17·76 (16·79–18·90)	13·22 (11·71–14·75)
		Egypt	72·64 (72·38–72·87)	62·41 (59·38–65·08)	67·69 (67·45–67·94)	59·42 (57·08–61·59)	74·45 (73·88–74·95)	63·84 (60·71–66·58)	68·71 (68·13–69·28)	60·23 (57·62–62·46)	16·42 (16·23–16·61)	12·16 (10·97–13·28)	13·50 (13·34–13·66)	10·12 (9·16–11·03)
		Iran	74·58 (72·51–76·50)	64·28 (61·12–67·51)	69·64 (67·24–71·98)	60·98 (57·86–64·21)	77·98 (75·87–80·02)	67·13 (63·56–70·62)	71·85 (69·18–74·18)	62·99 (59·82–66·40)	17·85 (16·78–19·04)	13·34 (11·86–14·92)	15·06 (14·08–16·17)	11·40 (10·11–12·80)
		Iraq	68·24 (65·65–70·65)	58·57 (55·18–61·60)	63·26 (59·95–66·53)	54·71 (51·15–58·09)	70·66 (67·52–73·64)	60·53 (56·47–63·92)	64·33 (60·79–68·46)	55·65 (51·90–59·50)	14·80 (13·47–16·11)	10·88 (9·40–12·25)	13·05 (11·96–15·04)	9·55 (8·38–10·94)
		Jordan	74·44 (73·52–75·33)	63·69 (60·58–66·46)	74·66 (73·91–75·44)	65·14 (62·30–67·75)	80·69 (79·65–81·78)	68·76 (65·18–72·06)	76·46 (75·09–77·61)	66·68 (63·79–69·52)	19·84 (19·18–20·58)	14·67 (13·09–16·13)	17·46 (16·64–18·17)	13·12 (11·76–14·44)
		Kuwait	80·19 (79·91–80·52)	68·91 (65·71–71·77)	76·27 (75·99–76·54)	66·90 (64·25–69·36)	81·55 (80·47–82·71)	70·19 (66·81–73·29)	79·43 (77·94–80·67)	69·56 (66·56–72·40)	19·18 (18·38–20·06)	14·41 (12·88–15·84)	18·94 (18·09–19·69)	14·40 (12·97–15·80)
		Lebanon	77·64 (75·78–79·42)	65·80 (62·25–69·26)	77·03 (74·16–79·60)	65·45 (61·58–69·00)	79·87 (77·76–82·32)	68·01 (64·42–71·62)	77·90 (75·08–80·86)	66·81 (62·93–70·47)	19·01 (18·09–20·31)	14·11 (12·60–15·68)	17·80 (16·14–19·72)	13·07 (11·28–15·01)
		Libya	76·92 (76·07–77·62)	66·28 (63·11–69·04)	73·91 (73·06–74·78)	64·74 (61·95–67·21)	76·86 (75·26–78·22)	66·09 (62·84–69·25)	71·41 (68·93–73·78)	62·45 (59·39–65·54)	17·84 (16·99–18·55)	13·34 (11·96–14·72)	15·90 (14·76–16·92)	11·99 (10·61–13·33)
		Morocco	73·80 (70·89–76·16)	63·24 (59·71–66·72)	71·62 (68·72–74·08)	61·88 (58·64–65·13)	75·83 (72·69–79·05)	64·92 (61·12–68·62)	73·32 (69·45–76·58)	63·37 (59·35–66·89)	17·20 (15·61–19·00)	12·70 (11·04–14·44)	16·39 (14·35–18·01)	12·13 (10·31–13·70)
		Palestine	77·34 (76·55–78·10)	66·84 (63·82–69·70)	72·06 (70·51–73·06)	62·49 (59·81–65·14)	77·90 (75·78–79·78)	67·44 (63·98–70·52)	71·60 (68·64–74·52)	62·50 (59·06–65·67)	18·17 (17·04–19·22)	13·84 (12·39–15·21)	15·01 (13·92–16·52)	11·35 (9·94–12·77)
		Oman	78·38 (77·34–79·32)	66·81 (63·47–69·91)	74·07 (72·92–75·18)	64·15 (61·29–66·85)	79·64 (78·29–81·14)	67·75 (64·31–71·05)	75·71 (74·05–77·69)	65·38 (62·24–68·65)	18·79 (18·11–19·71)	13·71 (12·25–15·17)	16·32 (15·48–17·55)	12·03 (10·70–13·57)
		Qatar	78·31 (77·49–79·11)	67·01 (63·71–70·07)	75·24 (73·63–76·87)	65·43 (62·30–68·46)	81·20 (79·43–82·88)	69·31 (65·82–72·71)	79·11 (76·93–81·25)	68·57 (64·92–71·83)	19·76 (18·65–20·98)	14·58 (12·92–16·27)	18·59 (17·07–19·91)	13·80 (12·07–15·51)
		Saudi Arabia	80·00 (79·43–80·55)	68·99 (65·85–71·89)	75·95 (75·29–76·58)	66·78 (64·09–69·25)	82·21 (81·29–83·09)	70·87 (67·53–74·08)	77·45 (76·34–78·53)	68·15 (65·33–70·73)	20·68 (19·98–21·46)	15·72 (14·25–17·18)	17·77 (16·98–18·51)	13·67 (12·41–14·90)
		Sudan	66·07 (62·29–70·01)	56·47 (52·50–60·70)	63·09 (59·34–67·21)	54·72 (50·75–58·67)	69·27 (65·38–73·59)	59·38 (55·50–63·72)	65·81 (61·55–69·91)	57·16 (53·08–61·35)	15·03 (13·32–17·27)	11·20 (9·59–13·17)	14·27 (12·39–15·93)	10·56 (8·92–12·10)
		Syria	77·37 (76·74–77·97)	67·20 (64·25–69·78)	73·85 (73·09–74·68)	65·31 (62·81–67·63)	74·81 (72·12–77·75)	63·82 (59·86–67·73)	62·58 (56·48–70·19)	54·17 (48·63–61·05)	17·98 (17·37–18·82)	13·46 (12·11–14·74)	15·47 (14·56–16·66)	11·74 (10·46–12·98)
		Tunisia	78·95 (77·45–80·46)	68·16 (64·92–71·20)	73·66 (71·83–75·41)	64·38 (61·36–67·24)	80·73 (78·82–82·71)	69·61 (66·03–72·89)	74·63 (72·03–77·02)	65·26 (61·88–68·39)	19·77 (18·50–21·22)	14·95 (13·25–16·56)	16·18 (14·85–17·69)	12·24 (10·67–13·82)
		Turkey	79·52 (78·90–80·10)	68·04 (64·90–70·99)	72·91 (72·25–73·57)	64·01 (61·39–66·35)	82·37 (81·82–82·94)	70·49 (66·94–73·59)	75·90 (75·25–76·55)	66·61 (63·86–69·04)	21·21 (20·91–21·55)	15·75 (14·19–17·16)	17·35 (16·89–17·67)	13·17 (11·92–14·27)
		United Arab Emirates	77·32 (76·47–78·00)	66·22 (63·07–69·16)	74·16 (73·26–74·93)	64·47 (61·53–67·14)	78·02 (75·54–79·89)	67·00 (63·40–70·34)	74·52 (71·82–77·08)	65·04 (61·38–68·31)	17·99 (16·84–18·95)	13·46 (11·93–14·80)	15·92 (14·70–17·04)	11·98 (10·50–13·33)
		Yemen	64·72 (59·58–70·44)	56·24 (51·57–61·26)	64·41 (59·19–69·89)	56·78 (52·15–61·46)	66·57 (60·83–71·63)	57·71 (52·65–62·10)	65·39 (59·70–70·23)	57·40 (52·34–61·68)	13·55 (11·14–16·14)	10·28 (8·32–12·21)	14·03 (11·65–16·29)	10·71 (8·75–12·50)
South Asia			65·22 (64·66–65·79)	55·61 (52·88–58·11)	62·43 (61·92–62·97)	54·26 (51·89–56·37)	69·56 (68·83–70·27)	59·52 (56·53–62·18)	65·42 (64·74–66·10)	57·15 (54·76–59·35)	15·10 (14·74–15·47)	11·19 (10·11–12·26)	13·39 (13·11–13·69)	10·04 (9·15–10·90)
		Bangladesh	68·70 (67·58–69·89)	58·62 (55·71–61·43)	65·67 (64·60–66·76)	57·34 (54·71–59·68)	72·52 (70·56–74·46)	62·18 (58·79–65·30)	68·52 (66·50–70·35)	60·06 (57·26–62·74)	16·16 (14·93–17·32)	12·13 (10·66–13·46)	14·58 (13·76–15·33)	11·12 (10·01–12·19)
		Bhutan	70·15 (67·77–72·32)	59·82 (56·53–63·05)	68·68 (66·42–70·67)	59·58 (56·50–62·30)	74·26 (71·54–76·54)	63·08 (59·42–66·49)	71·50 (69·05–74·20)	61·95 (58·52–65·20)	17·80 (16·35–19·13)	13·11 (11·52–14·60)	16·35 (15·06–17·75)	12·22 (10·73–13·75)
		India	65·03 (64·41–65·66)	55·38 (52·60–57·89)	62·22 (61·65–62·83)	54·01 (51·64–56·15)	69·53 (68·68–70·43)	59·44 (56·46–62·17)	65·24 (64·46–66·00)	56·97 (54·53–59·20)	15·06 (14·65–15·51)	11·14 (10·03–12·17)	13·21 (12·89–13·53)	9·89 (9·00–10·75)
		Nepal	68·83 (66·99–70·57)	59·54 (56·41–62·32)	65·96 (64·42–67·66)	57·63 (54·90–60·03)	71·23 (68·75–73·92)	61·58 (58·13–64·95)	68·09 (65·20–70·91)	59·53 (56·39–62·74)	15·56 (14·22–17·16)	11·81 (10·30–13·35)	14·76 (13·48–16·01)	11·25 (9·97–12·59)
		Pakistan	63·24 (61·42–65·05)	54·31 (51·48–57·18)	61·29 (59·54–63·19)	53·51 (50·86–55·94)	67·32 (65·22–69·82)	57·93 (54·76–61·01)	64·54 (62·22–66·49)	56·40 (53·59–59·28)	14·39 (13·34–15·78)	10·73 (9·41–12·06)	13·59 (12·72–14·32)	10·21 (9·14–11·34)
Sub-Saharan Africa			55·92 (55·05–56·74)	47·94 (45·32–50·16)	54·26 (53·46–55·10)	47·13 (44·90–49·12)	63·61 (61·83–65·17)	54·63 (51·52–57·44)	60·02 (58·35–61·58)	52·35 (49·72–54·90)	14·28 (13·44–15·06)	10·57 (9·31–11·69)	13·33 (12·62–13·99)	9·96 (8·86–10·99)
	Southern sub-Saharan Africa		51·80 (50·49–53·23)	44·71 (42·42–46·86)	49·85 (48·74–51·12)	43·62 (41·66–45·44)	63·21 (61·67–64·69)	54·06 (51·28–56·71)	57·51 (56·00–58·95)	50·14 (47·75–52·40)	14·81 (14·01–15·56)	10·80 (9·59–11·98)	12·35 (11·68–13·03)	9·05 (8·05–9·98)
		Botswana	50·78 (39·38–64·33)	44·97 (34·78–54·50)	47·92 (38·74–60·22)	42·81 (34·01–51·74)	62·01 (47·70–72·30)	53·95 (41·57–61·73)	55·78 (43·89–66·62)	49·39 (39·01–57·39)	13·82 (7·51–18·54)	10·57 (5·57–13·91)	12·01 (7·31–16·37)	9·10 (5·40–12·22)
		Lesotho	44·77 (41·20–48·58)	38·92 (35·56–42·30)	41·21 (38·65–44·59)	36·45 (34·07–39·63)	50·41 (43·61–58·50)	43·83 (37·87–49·81)	44·11 (38·61–51·77)	39·13 (34·27–44·74)	11·49 (8·12–15·20)	8·57 (5·97–11·17)	9·26 (7·16–12·77)	6·87 (5·22–9·42)
		Namibia	55·05 (51·33–59·06)	47·65 (43·90–51·40)	51·07 (48·07–54·29)	44·91 (41·85–48·01)	68·44 (61·62–73·04)	58·70 (52·80–63·47)	59·98 (53·61–66·28)	52·56 (46·87–58·13)	16·51 (12·83–18·64)	12·31 (9·39–14·52)	13·01 (10·40–15·97)	9·79 (7·69–12·19)
		South Africa	53·62 (52·18–55·38)	46·19 (43·83–48·49)	51·67 (50·36–53·14)	45·17 (43·09–47·15)	63·99 (62·55–65·18)	54·63 (51·81–57·23)	58·64 (57·32–59·91)	51·07 (48·74–53·20)	14·97 (14·18–15·69)	10·88 (9·65–12·06)	12·56 (11·93–13·21)	9·15 (8·16–10·12)
		Swaziland	43·17 (39·24–47·45)	37·24 (33·86–40·73)	41·68 (38·42–45·84)	36·52 (33·44–39·83)	54·87 (46·71–63·97)	47·12 (40·22–53·87)	48·96 (42·27–58·26)	43·01 (37·10–49·82)	12·24 (8·24–16·65)	9·06 (5·84–12·09)	10·36 (7·55–14·68)	7·68 (5·39–10·79)
		Zimbabwe	45·56 (42·36–49·34)	39·75 (36·90–42·77)	44·61 (41·95–48·39)	39·24 (36·79–42·17)	62·54 (56·27–68·35)	54·29 (48·68–59·44)	56·28 (51·09–62·63)	49·52 (44·79–54·76)	14·81 (11·38–18·26)	11·23 (8·38–13·90)	12·20 (10·09–15·22)	9·24 (7·37–11·68)
	Western sub-Saharan Africa		57·51 (56·07–58·82)	49·35 (46·72–51·71)	55·80 (54·46–57·19)	48·44 (46·03–50·65)	64·15 (60·80–66·47)	55·25 (51·81–58·49)	60·97 (58·24–62·97)	53·19 (50·17–55·91)	14·98 (13·35–16·06)	11·21 (9·74–12·54)	14·16 (13·01–15·00)	10·62 (9·32–11·78)
		Benin	60·97 (56·43–65·32)	52·53 (48·15–56·53)	56·21 (52·01–61·29)	49·70 (45·45–53·99)	64·84 (55·71–72·41)	56·44 (49·10–62·89)	59·88 (51·34–68·32)	53·59 (45·88–60·42)	13·93 (10·08–17·93)	10·67 (7·51–13·80)	12·40 (9·47–15·93)	9·66 (7·11–12·34)
		Burkina Faso	56·63 (53·42–59·73)	48·94 (45·56–52·15)	55·04 (52·19–58·27)	48·19 (44·89–51·56)	61·85 (54·32–68·96)	54·25 (47·62–60·31)	60·01 (52·54–66·03)	53·28 (47·34–58·69)	13·59 (10·13–17·19)	10·51 (7·72–13·28)	13·54 (10·32–16·35)	10·47 (7·98–12·83)
		Cameroon	55·92 (52·80–59·52)	48·21 (44·66–51·56)	53·84 (50·74–57·25)	46·89 (43·74–50·19)	61·20 (53·95–68·55)	53·35 (46·88–59·61)	57·59 (50·44–64·22)	50·85 (44·49–56·32)	14·06 (10·39–17·58)	10·68 (7·68–13·40)	12·99 (9·83–16·07)	9·86 (7·25–12·30)
		Cape Verde	76·02 (73·41–76·91)	65·63 (62·25–68·60)	63·22 (59·21–68·01)	56·01 (52·27–60·21)	76·55 (74·33–77·59)	66·26 (62·96–69·24)	69·13 (66·18–71·78)	60·99 (57·64–64·19)	18·09 (16·79–18·64)	13·68 (12·17–15·01)	14·85 (13·40–16·22)	11·31 (9·78–12·82)
		Chad	54·49 (49·28–60·02)	47·07 (42·17–51·79)	51·52 (46·42–57·50)	44·78 (40·30–49·49)	58·88 (49·37–67·61)	51·31 (43·16–58·31)	55·92 (47·52–63·60)	48·99 (41·63–55·16)	13·57 (9·15–18·17)	10·39 (6·83–13·74)	12·82 (9·30–16·41)	9·68 (6·76–12·31)
		Côte d'Ivoire	54·81 (51·77–58·19)	47·31 (44·08–50·42)	52·17 (49·40–55·26)	45·74 (42·79–48·69)	61·41 (55·01–68·52)	53·25 (47·55–58·86)	57·21 (49·99–64·50)	50·62 (44·57–56·49)	13·45 (10·36–17·14)	10·18 (7·63–12·89)	12·52 (9·58–15·76)	9·58 (7·19–12·09)
		The Gambia	66·15 (63·52–69·30)	57·47 (53·89–60·95)	63·77 (60·62–66·93)	56·24 (53·02–59·47)	69·63 (64·33–74·55)	60·60 (55·53–65·49)	66·65 (61·40–70·72)	58·85 (53·84–63·05)	15·20 (12·74–17·85)	11·66 (9·40–13·93)	14·44 (12·06–16·15)	11·12 (9·08–12·84)
		Ghana	62·22 (58·39–66·35)	54·17 (50·38–58·28)	59·44 (55·06–63·57)	52·58 (48·64–56·59)	67·55 (59·65–74·25)	59·26 (52·16–65·27)	63·06 (54·99–69·81)	56·43 (49·27–62·23)	14·65 (11·01–18·42)	11·43 (8·29–14·33)	13·26 (10·33–16·14)	10·37 (7·79–12·80)
		Guinea	56·87 (54·19–59·83)	49·13 (46·04–52·16)	55·17 (52·35–58·20)	48·39 (45·37–51·39)	60·49 (54·52–67·09)	52·65 (47·07–58·36)	57·81 (50·80–64·49)	51·10 (45·32–56·48)	13·11 (10·29–16·46)	10·04 (7·59–12·67)	12·74 (9·77–15·74)	9·76 (7·38–12·08)
		Guinea-Bissau	53·45 (41·87–65·17)	47·62 (37·00–56·49)	52·32 (41·45–61·55)	46·95 (36·92–54·28)	56·44 (42·35–67·69)	50·29 (37·66–58·94)	54·86 (41·87–63·97)	49·30 (37·55–56·40)	12·49 (7·38–18·14)	9·86 (5·56–13·75)	12·60 (7·97–16·67)	9·81 (6·01–12·82)
		Liberia	58·41 (55·78–61·50)	49·34 (45·91–52·69)	58·42 (55·80–61·11)	49·59 (46·21–52·76)	63·41 (57·41–69·44)	54·28 (49·06–59·54)	63·08 (57·54–67·67)	54·32 (49·25–58·93)	13·46 (10·70–16·61)	9·89 (7·64–12·38)	14·11 (11·59–16·32)	10·32 (8·27–12·33)
		Mali	57·10 (54·19–59·62)	48·99 (45·63–52·25)	56·70 (54·17–59·25)	49·03 (45·80–51·97)	60·44 (54·41–66·68)	52·48 (46·95–57·55)	60·04 (54·18–64·35)	52·64 (47·53–56·71)	13·86 (10·84–16·93)	10·51 (8·06–12·88)	14·63 (12·17–16·71)	11·06 (8·94–12·93)
		Mauritania	66·08 (63·12–68·95)	56·59 (53·00–60·00)	66·76 (63·89–69·70)	57·74 (54·26–60·96)	69·81 (63·59–75·57)	60·13 (54·58–65·16)	69·83 (63·66–75·84)	60·72 (55·13–65·85)	15·43 (12·47–18·74)	11·57 (9·08–13·97)	16·23 (13·54–19·71)	12·20 (9·82–14·85)
		Niger	56·69 (53·93–59·39)	49·23 (46·04–52·19)	55·23 (52·52–57·72)	48·42 (45·56–51·12)	62·47 (56·63–68·18)	54·62 (49·53–59·73)	59·58 (52·81–65·38)	52·71 (46·88–57·41)	14·18 (11·30–17·09)	10·82 (8·50–13·07)	13·37 (10·41–15·90)	10·20 (7·88–12·23)
		Nigeria	57·27 (54·37–59·89)	48·85 (45·55–51·94)	56·32 (53·71–59·05)	48·49 (45·46–51·58)	66·33 (59·47–70·32)	56·70 (51·20–61·03)	62·95 (57·35–65·78)	54·38 (49·69–57·95)	16·69 (13·14–18·76)	12·42 (9·77–14·48)	15·56 (13·16–16·63)	11·54 (9·51–13·09)
		São Tomé and Príncipe	66·23 (62·95–69·55)	57·52 (53·96–60·98)	64·70 (61·02–68·11)	56·82 (53·12–60·37)	68·81 (61·31–75·85)	59·88 (53·33–66·12)	67·19 (59·36–74·84)	59·20 (52·56–65·53)	14·08 (10·84–17·67)	10·72 (8·12–13·39)	14·05 (11·23–17·55)	10·75 (8·30–13·43)
		Senegal	63·34 (58·73–68·24)	54·93 (50·49–59·50)	60·96 (55·78–65·54)	53·85 (49·19–57·99)	66·70 (58·22–74·46)	58·42 (50·73–64·76)	63·84 (54·36–70·63)	56·97 (48·79–63·01)	13·78 (10·12–17·89)	10·62 (7·54–13·61)	13·27 (9·98–16·37)	10·37 (7·55–12·81)
		Sierra Leone	53·25 (51·13–55·51)	45·84 (43·07–48·53)	51·31 (49·15–53·49)	44·43 (41·65–46·90)	57·73 (51·98–63·30)	50·57 (45·66–55·39)	56·54 (50·98–62·48)	49·86 (44·73–54·86)	12·49 (9·78–15·38)	9·48 (7·31–11·79)	12·90 (10·28–15·60)	9·74 (7·58–11·87)
		Togo	59·02 (55·78–62·50)	51·21 (47·63–54·69)	54·59 (51·23–58·28)	48·11 (44·83–51·43)	64·61 (58·52–70·49)	56·48 (50·97–61·52)	58·47 (52·25–65·69)	52·02 (46·39–57·51)	14·56 (11·46–17·65)	11·20 (8·60–13·65)	12·32 (9·91–15·47)	9·53 (7·35–11·85)
	Eastern sub-Saharan Africa		56·08 (54·83–57·34)	48·45 (45·86–50·76)	54·53 (53·31–55·72)	47·61 (45·40–49·71)	64·32 (61·38–67·07)	55·84 (52·48–58·99)	60·50 (57·72–63·16)	53·13 (50·12–56·22)	13·98 (12·61–15·37)	10·53 (9·15–11·90)	13·06 (11·85–14·21)	9·88 (8·61–11·14)
		Burundi	55·27 (52·28–58·06)	48·44 (45·16–51·56)	54·16 (50·88–57·52)	47·83 (44·43–51·02)	62·69 (54·73–70·23)	55·80 (48·75–62·27)	60·10 (53·06–67·68)	53·82 (47·80–59·82)	12·82 (9·59–16·50)	10·08 (7·37–12·88)	12·63 (9·89–15·75)	9·88 (7·68–12·18)
		Comoros	66·70 (63·68–69·99)	57·99 (54·66–61·50)	65·33 (62·26–68·60)	57·09 (53·78–60·39)	69·29 (63·40–75·47)	60·62 (54·98–65·59)	66·88 (59·97–73·41)	58·93 (52·88–64·41)	14·53 (11·92–17·65)	11·10 (8·78–13·54)	14·20 (11·45–17·21)	10·82 (8·68–13·10)
		Djibouti	63·91 (53·86–71·72)	55·49 (47·06–61·84)	57·84 (47·80–66·54)	51·35 (42·10–58·41)	66·98 (56·90–74·05)	58·00 (50·10–64·10)	60·88 (50·23–69·17)	53·93 (44·93–60·78)	14·82 (10·34–18·71)	11·17 (7·77–13·99)	12·68 (9·03–16·33)	9·67 (6·85–12·37)
		Eritrea	60·25 (51·43–69·82)	52·69 (45·09–59·93)	59·80 (49·75–67·61)	52·23 (44·29–58·27)	61·27 (51·78–71·86)	54·02 (45·38–61·97)	60·30 (50·84–69·23)	53·13 (44·87–60·06)	11·72 (8·22–16·59)	9·11 (6·24–12·36)	12·28 (9·13–15·98)	9·37 (6·69–12·12)
		Ethiopia	56·14 (53·45–58·72)	49·32 (46·48–52·07)	56·04 (53·51–58·96)	49·30 (46·65–52·30)	66·80 (59·33–73·42)	58·77 (52·50–64·20)	63·57 (55·62–69·63)	56·28 (49·52–61·63)	14·27 (10·72–17·84)	11·00 (8·27–13·63)	13·56 (10·54–16·34)	10·42 (7·90–12·58)
		Kenya	58·53 (57·14–59·88)	49·67 (46·13–52·44)	55·96 (54·64–57·25)	48·76 (46·34–50·93)	67·57 (65·77–69·31)	57·66 (53·64–61·01)	62·82 (61·09–64·62)	54·92 (52·29–57·47)	15·31 (14·17–16·53)	11·45 (9·89–12·97)	13·87 (13·01–14·77)	10·59 (9·37–11·68)
		Madagascar	62·33 (59·40–65·25)	53·64 (50·13–57·00)	60·13 (56·80–62·98)	52·03 (48·41–55·18)	65·04 (57·77–72·35)	56·75 (50·52–62·59)	61·91 (54·45–69·95)	54·43 (48·02–60·83)	13·15 (10·10–16·69)	9·99 (7·55–12·60)	12·64 (9·95–15·97)	9·56 (7·46–11·87)
		Malawi	49·32 (46·27–52·66)	42·60 (39·74–45·60)	47·72 (44·73–51·41)	41·63 (38·71–44·92)	62·98 (56·85–68·70)	54·97 (49·46–59·86)	58·15 (51·92–63·72)	51·35 (46·05–56·40)	14·81 (11·53–17·83)	11·32 (8·60–13·68)	13·51 (10·59–16·30)	10·37 (7·97–12·57)
		Mozambique	54·79 (51·04–58·72)	46·48 (42·43–50·10)	50·88 (47·90–54·71)	44·16 (41·06–47·64)	59·86 (52·11–67·28)	51·69 (45·15–57·69)	54·28 (47·35–61·94)	47·80 (41·60–53·86)	13·85 (9·99–17·92)	10·32 (7·24–13·27)	11·87 (9·20–15·65)	8·84 (6·56–11·72)
		Rwanda	60·97 (58·02–64·00)	52·57 (48·95–56·16)	58·08 (55·32–61·18)	50·39 (47·21–53·62)	67·68 (60·39–73·73)	59·11 (52·82–64·58)	63·73 (56·40–69·34)	56·16 (49·82–61·32)	15·06 (11·69–18·23)	11·56 (8·91–14·25)	13·95 (10·78–16·52)	10·74 (8·17–12·89)
		Somalia	52·51 (41·10–66·66)	45·86 (35·54–55·66)	51·66 (41·01–63·08)	46·33 (36·49–54·83)	54·98 (42·35–67·96)	47·96 (37·04–57·19)	53·40 (41·87–64·12)	47·76 (37·41–55·73)	10·69 (7·12–16·09)	8·10 (5·21–11·73)	11·26 (7·63–15·67)	8·71 (5·75–11·88)
		South Sudan	55·74 (43·07–67·56)	48·44 (37·79–57·07)	54·87 (43·09–64·34)	47·89 (37·96–55·43)	56·87 (43·88–68·72)	49·77 (38·27–58·38)	55·45 (43·16–64·87)	48·64 (38·05–56·08)	11·97 (7·37–17·66)	8·98 (5·34–13·00)	12·33 (7·95–16·51)	9·12 (5·83–12·18)
		Tanzania	57·17 (53·50–60·79)	49·44 (46·06–53·05)	57·12 (53·46–60·80)	49·74 (46·17–53·19)	65·47 (56·90–72·65)	57·40 (50·02–63·49)	62·69 (54·76–68·51)	55·32 (48·37–60·49)	14·46 (10·45–18·66)	11·18 (7·94–14·12)	13·93 (10·64–16·46)	10·71 (8·00–12·82)
		Uganda	55·34 (52·66–58·44)	47·78 (44·69–50·97)	51·71 (49·09–54·57)	45·36 (42·49–48·23)	64·35 (56·89–71·57)	56·25 (49·89–62·36)	58·36 (51·27–66·03)	51·76 (45·44–57·69)	14·11 (10·60–18·08)	10·90 (7·98–13·80)	12·40 (9·73–15·74)	9·54 (7·25–12·13)
		Zambia	48·46 (45·68–51·26)	42·41 (39·54–45·15)	45·78 (43·60–48·33)	40·36 (37·94–42·72)	59·83 (53·63–67·00)	52·38 (46·92–58·21)	53·97 (48·48–61·10)	47·77 (42·64–53·66)	12·74 (9·89–16·41)	9·81 (7·34–12·57)	10·61 (8·78–13·49)	8·07 (6·51–10·38)
	Central sub-Saharan Africa		56·41 (52·60–59·67)	46·71 (41·51–50·81)	54·60 (51·21–57·42)	46·50 (42·70–49·68)	61·04 (55·98–66·25)	50·71 (44·61–55·69)	58·94 (53·90–63·87)	50·56 (46·12–54·91)	12·79 (10·79–15·11)	8·87 (6·84–10·89)	12·61 (10·67–14·58)	9·12 (7·53–10·85)
		Angola	55·19 (43·52–67·89)	47·66 (37·79–56·61)	55·20 (44·08–64·69)	48·52 (38·95–56·00)	59·75 (45·75–71·66)	51·73 (39·66–60·53)	59·55 (46·05–68·63)	52·43 (41·43–59·55)	12·10 (7·38–17·92)	8·97 (5·42–12·88)	12·72 (8·13–16·57)	9·57 (6·13–12·44)
		Central African Republic	47·63 (39·73–57·62)	41·10 (34·21–48·26)	43·62 (37·13–52·61)	38·33 (32·47–45·21)	51·75 (42·48–63·18)	45·12 (37·16–53·72)	47·55 (39·54–59·15)	42·15 (34·69–50·66)	10·90 (7·53–16·52)	8·25 (5·45–12·08)	9·75 (7·41–14·86)	7·34 (5·32–10·57)
		Congo	55·61 (52·62–58·83)	47·40 (43·62–50·61)	58·08 (54·72–61·61)	50·39 (46·85–54·00)	60·01 (52·72–68·01)	51·32 (45·16–57·62)	63·40 (55·94–68·74)	55·23 (48·88–60·46)	11·54 (8·70–15·25)	8·42 (6·22–11·06)	14·00 (11·01–16·37)	10·51 (8·10–12·58)
		Democratic Republic of the Congo	57·64 (55·14–60·61)	47·07 (40·13–51·19)	55·25 (52·58–57·86)	46·61 (42·75–49·94)	62·14 (56·77–68·11)	51·04 (43·96–56·67)	59·37 (53·21–65·66)	50·66 (45·33–55·99)	13·20 (10·70–16·25)	9·04 (6·57–11·48)	12·70 (10·21–15·37)	9·14 (7·12–11·15)
		Equatorial Guinea	56·51 (44·74–67·67)	49·44 (38·84–57·56)	55·55 (44·50–63·43)	48·93 (39·34–55·10)	61·90 (48·89–71·63)	53·99 (42·57–61·32)	60·27 (48·44–67·38)	53·03 (42·56–58·90)	13·77 (8·32–18·74)	10·47 (6·04–13·86)	13·88 (9·07–16·60)	10·47 (6·73–12·86)
		Gabon	61·28 (58·52–64·16)	51·68 (47·81–55·07)	59·26 (55·55–63·45)	51·16 (47·61–55·01)	68·45 (61·48–74·32)	57·02 (50·37–62·57)	63·58 (56·80–69·02)	55·00 (48·91–60·04)	14·41 (11·30–17·40)	10·16 (7·60–12·55)	13·98 (11·11–16·34)	10·34 (8·00–12·41)

Data in parentheses are 95% uncertainty intervals. HALE=healthy life expectancy. SDI=Socio-demographic Index.

## References

[1] UN (Oct 29, 2015). Sustainable development goals. http://www.un.org/sustainabledevelopment/sustainable-development-goals/.

[2] Murray CJL (2015). Choosing indicators for the health-related SDG targets. Lancet.

[3] Murray CJL (2015). Shifting to Sustainable Development Goals—Implications for global health. N Engl J Med.

[4] Nilsson M, Griggs D, Visbeck M (2016). Policy: Map the interactions between Sustainable Development Goals. Nature News.

[5] UN United Nations Sustainable Development. http://www.un.org/sustainabledevelopment/health/.

[6] Murray CJL, Salomon JA, Mathers CD, Lopez AD (2002). Summary Measures in Population Health: Concepts, Ethics, Measurement, and Applications.

[7] Sullivan DF (1971). A single index of mortality and morbidity. HSMHA Health Rep.

[8] Mathers CD, Sadana R, Salomon JA, Murray CJ, Lopez AD (2001). Healthy life expectancy in 191 countries, 1999. Lancet.

[9] Murray CJ, Vos T, Lozano R (2012). Disability-adjusted life years (DALYs) for 291 diseases and injuries in 21 regions, 1990–2010: a systematic analysis for the Global Burden of Disease Study 2010. Lancet.

[10] Murray CJ, Barber RM, Foreman KJ (2015). Global, regional, and national disability-adjusted life years (DALYs) for 306 diseases and injuries and healthy life expectancy (HALE) for 188 countries, 1990–2013: quantifying the epidemiological transition. Lancet.

[11] Murray CJ (1994). Quantifying the burden of disease: the technical basis for disability-adjusted life years. Bull World Health Organ.

[12] GBD 2015 Mortality and Causes of Death Collaborators (2016). Global, regional, and national life expectancy, all-cause mortality, and cause-specific mortality for 249 causes of death, 1980–2015: a systematic analysis for the Global Burden of Disease Study 2015. Lancet.

[13] WHO (2013). WHO methods and data sources for global burden of disease estimates 2000–2011.

[14] WHO (2016). WHO methods and data sources for life tables 1990–2015.

[15] EHLEIS (April 2015). Bibliography on Health Expectancy in Europe. http://www.eurohex.eu/pdf/Reports_2015/2015_TR4%204_Bibliography.pdf.

[16] WHO (2014). WHO methods and data sources for country-level causes of death 2000–2012.

[17] WHO Estimates for 2000–2012. http://www.who.int/healthinfo/global_burden_disease/estimates/en/.

[18] European Commission Healthy Life Years (HLY). http://ec.europa.eu/health/indicators/healthy_life_years/hly_en.htm#fragment2.

[19] OECD (2012). Health at a Glance: Europe 2012.

[20] GBD 2015 Risk Factors Collaborators (2016). Global, regional, and national incidence, prevalence, and years lived with disability for 310 diseases and injuries during 1990–2015: a systematic analysis for the Global Burden of Disease Study 2015. Lancet.

[21] Murray CJ, Ezzati M, Flaxman AD (2012). GBD 2010: design, definitions, and metrics. Lancet.

[22] Stevens GA, Alkema L, Black RE (2016). Guidelines for Accurate and Transparent Health Estimates Reporting: the GATHER statement. Lancet.

[23] Stevens GA, Alkema L, Black RE (2016). Guidelines for Accurate and Transparent Health Estimates Reporting: the GATHER statement. PLoS Med.

[24] Stanaway JD, Shepard DS, Undurraga EA (2016). The global burden of dengue: an analysis from the Global Burden of Disease Study 2013. Lancet Infect Dis.

[25] Salomon JA, Vos T, Hogan DR (2012). Common values in assessing health outcomes from disease and injury: disability weights measurement study for the Global Burden of Disease Study 2010. Lancet.

[26] Salomon JA, Haagsma JA, Davis A (2015). Disability weights for the Global Burden of Disease 2013 study. Lancet Glob Health.

[27] Salomon JA, Wang H, Freeman MK (2012). Healthy life expectancy for 187 countries, 1990–2010: a systematic analysis for the Global Burden Disease Study 2010. Lancet.

[28] Calculating the Indices Human Development Reports. http://hdr.undp.org/en/content/calculating-indices.

[29] WHO Ebola Response Team (2015). West African Ebola epidemic after one year— slowing but not yet under control. N Engl J Med.

[30] Fries JF (2005). The compression of morbidity. Milbank Q.

[31] Langa KM, Larson EB, Karlawish JH (2008). Trends in the prevalence and mortality of cognitive impairment in the United States: is there evidence of a compression of cognitive morbidity?. Alzheimers Dement.

[32] Bardenheier BH, Lin J, Zhuo X (2016). Compression of disability between two birth cohorts of US adults with diabetes, 1992–2012: a prospective longitudinal analysis. Lancet Diabetes Endocrinol.

[33] Beltran-Sanchez H, Preston S, Canudas-Romo V (2008). An integrated approach to cause-of-death analysis: cause-deleted life tables and decompositions of life expectancy. Demogr Res.

[34] Jagger C, Matthews R, Matthews F (2007). The burden of diseases on disability-free life expectancy in later life. J Gerontol A Biol Sci Med Sci.

[35] van Gool CH, Picavet HSJ, Deeg DJ (2011). Trends in activity limitations: the Dutch older population between 1990 and 2007. Int J Epidemiol.

[36] King G, Murray CJL, Salomon JA, Tandon A (2004). Enhancing the validity and cross-cultural comparability of measurement in survey research. Am Polit Sci Rev.

[37] Salomon JA, Tandon A, Murray CJL (2004). Comparability of self rated health: cross sectional multi-country survey using anchoring vignettes. BMJ.

[38] Tandon A, Murray CJL, Salomon JA, King G (2002). Statistical Models for Enhancing Cross-Population Comparability.

[39] Bowling A (2011). Commentary: Trends in activity limitation. Int J Epidemiol.

[40] Jamison DT, Summers LH, Alleyne G (2013). Global health 2035: a world converging within a generation. Lancet.

[41] GBD 2015 SDG Collaborators (2016). Measuring the health-related Sustainable Development Goals in 188 countries: a baseline analysis from the Global Burden of Disease Study 2015. Lancet.

[42] Engineer MH, Roy N, Fink S (2009). ‘Healthy’ Human Development Indices. Soc Indic Res.

[43] National Institutes of Health (NIH) (Dec 15, 2015). NIH unveils FY2016–2020 Strategic Plan. http://www.nih.gov/news-events/news-releases/nih-unveils-fy2016-2020-strategic-plan.

[44] Gillum LA, Gouveia C, Dorsey ER (2011). NIH disease funding levels and burden of disease. PLoS One.

[45] Emdin CA, Odutayo A, Hsiao AJ (2015). Association between randomised trial evidence and global burden of disease: cross sectional study (Epidemiological Study of Randomized Trials—ESORT). BMJ.

[46] Álvarez-Martín E, Gènova-Maleras R, Morant-Ginestar C, Catalá-López Ferrán, García-Altés Anna (2010). Does the development of new medicinal products in the European Union address global and regional health concerns?. Popul Health Metr.

[47] WHO Investing in health research and development. http://www.who.int/tdr/publications/tdr-research-publications/investing-in-health/en/.

[48] Chalmers I, Bracken MB, Djulbegovic B (2014). How to increase value and reduce waste when research priorities are set. Lancet.

[49] GBD 2015 Risk Factors Collaborators (2016). Global, regional, and national comparative risk assessment of 79 behavioural, environmental and occupational, and metabolic risks or clusters of risks, 1990–2015: a systematic analysis for the Global Burden of Disease Study 2015. Lancet.

[50] Leigh JP, Sheetz RM (1989). Prevalence of back pain among fulltime United States workers. Br J Ind Med.

[51] Leino-Arjas P, Hänninen K, Puska P (1998). Socioeconomic variation in back and joint pain in Finland. Eur J Epidemiol.

[52] Waddell G, Burton AK (2001). Occupational health guidelines for the management of low back pain at work: evidence review. Occup Med (Lond).

[53] Haddon W (1980). Advances in the epidemiology of injuries as a basis for public policy. Public Health Rep.

[54] Moore L, Evans D, Hameed SM (2016). Mortality in Canadian trauma systems: a multicenter cohort Study. Ann Surg.

[55] Uthkarsh PS, Gururaj G, Reddy SS, Rajanna MS (2016). Assessment and availability of trauma care services in a district hospital of south India: a field observational study. Bull Emerg Trauma.

[56] Ghajar J (2000). Traumatic brain injury. Lancet.

[57] Cheatham ML, Malbrain ML, Kirkpatrick A (2007). Results from the International Conference of Experts on Intra-abdominal Hypertension and Abdominal Compartment Syndrome. II. Recommendations. Intensive Care Med.

[58] Stuke LE, Pons PT, Guy JS, Chapleau WP, Butler FK, McSwain NE (2011). Prehospital spine immobilization for penetrating trauma—review and recommendations from the Prehospital Trauma Life Support Executive Committee. J Trauma.

[59] Rickard JL, Ntakiyiruta G, Chu KM (2015). Associations with perioperative mortality rate at a major referral hospital in Rwanda. World J Surg.

[60] Odhiambo FO, Beynon CM, Ogwang S (2013). Trauma-related mortality among adults in rural western Kenya: characterising deaths using data from a Health and Demographic Surveillance System. PLoS One.

[61] Naish S, Dale P, Mackenzie JS, McBride J, Mengersen K, Tong S (2014). Climate change and dengue: a critical and systematic review of quantitative modelling approaches. BMC Infect Dis.

[62] GBD 2015 Child Mortality Collaborators (2016). Global, regional, national, and selected subnational levels of stillbirths, neonatal, infant, and under-5 mortality, 1980–2015: a systematic analysis for the Global Burden of Disease Study 2015. Lancet.

[63] GBD 2015 Maternal Mortality Collaborators (2016). Maternal mortality 1990 to 2015: a systematic analysis of the Global Burden of Disease 2015 Study. Lancet.

[64] Murray CJL, Ferguson BD, Lopez AD, Guillot M, Salomon JA, Ahmad O (2003). Modified logit life table system: principles, empirical validation, and application. Popul Stud.

[65] Wang H, Dwyer-Lindgren L, Lofgren KT (2012). Age-specific and sex-specific mortality in 187 countries, 1970–2010: a systematic analysis for the Global Burden of Disease Study 2010. Lancet.

[66] GBD 2013 Mortality and Causes of Death Collaborators (2015). Global, regional, and national age-sex specific all-cause and cause-specific mortality for 240 causes of death, 1990–2013: a systematic analysis for the Global Burden of Disease Study 2013. Lancet.

[67] Stouthard ME, Essink-Bot M, Bonsel G, Barendregt J, Kramers P (1997). Disability weights for diseases in the Netherlands.

[68] UN, Department of Economic and Social Affairs, Population Division (2013). World Population Prospects: The 2012 Revision, Key Findings and Advance Tables. Working Paper number ESA/P/WP.227.

