# Department of Error

**DOI:** 10.1016/S0140-6736(16)32607-1

**Published:** 2017-01-07

*GBD 2015 DALYs and HALE Collaborators. Global, regional, and national disability-adjusted life-years (DALYs) for 315 diseases and injuries and healthy life expectancy (HALE), 1990-2015: a systematic analysis for the Global Burden of Disease Study 2015.* Lancet *2016; **388:** 1603–58*—In this Article, Christine Allen, Stan Biryukov, Giorgia Giussani, Harish Gugnani, Simon I Hay, Anoushka Millear, and Thomas Truelsen should have been listed as authors. The affiliation details for Monica Cortinovis have been updated. A funding statement for Simon I Hay has been added. These changes have been made to the online version as of Jan 5, 2017.

