# Supplementary material for: Global, regional, and national disability-adjusted life-years (DALYs) for 315 diseases and injuries and healthy life expectancy (HALE), 1990–2015: a systematic analysis for the Global Burden of Disease Study 2015
Source: Lancet. 2016 Oct 8;388(10053):1603–58. doi: 10.1016/S0140-6736(16)31460-X (PMC5388857; doi:10.1016/S0140-6736(16)31460-X)

THE LANCET

Supplementary appendix

This appendix formed part of the original submission and has been peer reviewed. We post it as supplied by the authors.

Supplement to: GBD 2015 DALYs and HALE Collaborators. Global, regional, and national disability-adjusted life-years (DALYs) for 315 diseases and injuries and healthy life expectancy (HALE), 1990–2015: a systematic analysis for the Global Burden of Disease Study 2015. *Lancet* 2016; **388:** 1603–58.

Appendix to Global, regional, and national disability-adjusted life years (DALYs) for 315 diseases and injuries and healthy life expectancy (HALE) for 195 countries and territories, 1990-2015: a systematic analysis for the Global Burden of Diseases, Injuries, and Risk Factors (GBD) 2015 Study

This appendix provides methodological detail, supplemental figures, and more detailed results for disability-adjusted life years and healthy life expectancy. The appendix is organized into broad sections following the structure of the main paper.

Preamble

This appendix provides methodological detail and more detailed results for disability-adjusted life years and healthy life expectancy. This study complies with the Guidelines for Accurate and Transparent Health Estimates Reporting (GATHER) recommendations. It includes detailed tables and information on data in an effort to maximize transparency in our estimation processes and provide a comprehensive description of analytical steps. We intend this to be a living document, to be updated with each annual iteration of the Global Burden of Disease.

Table of Contents

Preamble 2

Section 1. Socio-Demographic Index (SDI) analysis & Epidemiological Transition 4

Section 2. GATHER Compliance Table 6

Appendix Table 1. GATHER checklist of information that should be included in reports of global health estimates, with description of compliance and location of information for GBD 2015 DALYs and HALE capstone 6

Section 2. Supplementary Results 11

Appendix Table 2. Life expectancy at birth and HALE at birth and at age 65 by location for GBD years 1990 - 2015, both sexes combined. 12

Appendix Table 3. DALYs for all causes and level 1 cause groups by location for 1990 - 2015, both sexes combined. 17

Appendix Table 4. Rate of DALYs per 100,000 individuals for all causes and level 1 cause groups by location for 1990 - 2015, both sexes combined. 39

Appendix Table 5. Socio-Demographic Index (SDI) values for all estimated GBD 2015 locations, 1980-2015. 74

Appendix Figure 1. Co-evolution of the proportion of DALYs due to YLDs with SDI for the globe and GBD regions from 1990 to 2015, with comparison to the value of expected proportion of DALYs due to YLDs based on SDI alone. 81

Appendix Figure 2. Years of functional health lost computed as life expectancy at birth minus HALE at birth for both sexes combined, 2015. 82

Appendix Figure 3. Observed HALE at birth minus expected HALE at birth for both sexes combined, 2015. 83

Appendix Figure 4. Ratio of observed versus expected age-standardised DALY rates (per 100,000) on the basis of SDI alone for both sexes combined, 2005. 84

Appendix Figure 5A. Comparisons of GBD 2015 HALE at birth with WHO 2015 HALE at birth. 85

Appendix Figure 5B. Comparisons of GBD 2014 HALE at birth with EC 2014 HALE at birth. 86

Appendix Figure 5C. Comparisons of GBD 2014 HALE at age 65 with EC 2014 HALE at age 65. 87

Section 1. Socio-Demographic Index (SDI) analysis & Epidemiological Transition

We began exploring the relationship between a composite indicator of socio-demographic development in GBD 2013 DALYs. We used lag distributed income per capita (LDI), average educational attainment over the age 15 years, total fertility rate (TFR), and mean population age and called it SDS, socio-demographic status. In response to feedback, we excluded mean population age due its strong relationship to mortality rates. We renamed the indicator Socio-Demographic Index (SDI). SDI has an interpretable scale: zero represents the lowest income per capita, lowest educational attainment, and highest TFR observed across all GBD geographies from 1980 to 2015 and one represents the highest income per capita, highest educational attainment, and lowest TFR.

SDI was calculated using the Human Development Index (HDI) methodology, wherein an index value was determined for each covariate input (log LDI, average educational attainment in the population over age 15, and TFR):

$$I_{Cly} = {(C_{ly} - \min(C))}\Big/{(\max(C) - \min(C))}$$

Where I_{cly} – the index for covariate *C*, location *l*, and year *y* – is equal to the difference between the value of that covariate in that location-year and the minimum observed value of the covariate (min(C)) in any location over the 1980-2015 time interval divided by the observed range (max(C)-min(C)). An additional innovation for GBD 2015 was to incorporate subnational locations where estimated (resulting in 519 unique administrative units) for the entire estimation period of 1980-2015. The Socio-Demographic Index is then the geometric mean of these three indices:

$$SDI = \sqrt[3]{I_{lnLDI} I_{educ} I_{TFR}}$$

In our mortality analyses, for LDI and TFR, we noted depreciating gains in life expectancy at birth and 5q0 at the higher and lower terminals, respectively. Due to the significance of these values in indexing, we aimed to identify the point at which increasing income or reducing fertility no longer resulted in improved child mortality or life expectancy. We tested various restrictions, and found that capping LDI at $60,000 and setting a TFR floor at 1 resulted in improved correlations with the resultant health indicators.

We further aimed to validate the use of SDI by regressing it in a variety of forms against life expectancy at birth, 5q0, 35q15, and 20q50. We found that SDI generally is as capable of predicting these demographic indicators as the previous SDS, and also as the inputs. We also found that in incorporating year, we did not substantially reduce the coefficients for SDI. Additionally, in testing lags of 2-10 years, we found the version with no lag to be the most predictive. Appendix Table 5. SDI values by GBD geography over time illustrates these results more in-depth.

We derived expected DALYs on the basis of SDI using the separate YLL and YLD models described in the

GBD 2015 mortality and non-fatal papers.[1] Additionally, HALE was calculated using the same methods described in the main text of this paper, with expected YLD rates and expected life tables as inputs.

[1] GBD 2015 Mortality and Causes of Death Collaborators. Global, regional, and national life expectancy, all-cause and cause-specific mortality for 249 causes of death, 1980–2015: a systematic analysis for the Global Burden of Disease Study 2015. The Lancet Under review.

GBD 2015 Diseases and Injury Incidence and Prevalence Collaborators. Global, regional, and national incidence, prevalence, and years lived with disability (YLDs) for 310 acute and chronic diseases and injuries, 1990-2015: a systematic analysis for the Global Burden of Disease Study 2015. The Lancet Under review.

Section 2. GATHER Compliance Table

Appendix Table 1. GATHER checklist of information that should be included in reports of global health estimates, with description of compliance and location of information for GBD 2015 DALYs and HALE capstone

#	GATHER checklist item	Description of compliance	Reference
**Objectives and funding**			
1	Define the indicators, populations, and time periods for which estimates were made.	Narrative provided in paper describing indicators, definitions, time periods, and populations.	Main text (Introduction, Methods)
2	List the funding sources for the work.	Funding sources listed in paper.	Main text (Summary, Funding)
**Data Inputs**			
For all data inputs from multiple sources that are synthesized as part of the study:			
3	Describe how the data were identified and how the data were accessed.	All data seeking methodology referenced in main text.	1. Main text (Methods)<br><br>2. Main text from citation 12 (Methods, Nonfatal outcome estimation)<br>3. Appendix from citation 12 (Parts 1-3)<br><br>4. Main text from citation 20 (Methods)<br>5. Appendix from citation 20 (Section 2. Nonfatal outcome estimation, Step 1. Data sources, identification and extraction)<br><br>*Citations*<br><br>12 GBD 2015 Mortality and Causes of Death Collaborators. Global, regional, and national life expectancy, all-cause and cause-specific mortality for 249 causes of death, 1980–2015: a systematic

			analysis for the Global Burden of Disease Study 2015. The Lancet Under review.<br><br>20 GBD 2015 Diseases and Injury Incidence and Prevalence Collaborators. Global, regional, and national incidence, prevalence, and years lived with disability (YLDs) for 310 acute and chronic diseases and injuries, 1990-2015: a systematic analysis for the Global Burden of Disease Study 2015. The Lancet Under review.
4	Specify the inclusion and exclusion criteria. Identify all ad-hoc exclusions.	All inclusion and exclusion criteria by data type referenced in main text.	1. Main text (Methods)<br><br>2. Main text from citation 12 (Methods, Nonfatal outcome estimation)<br>3. Appendix from citation 12 (Parts 1-3)<br><br>4. Main text from citation 20 (Methods)<br>5. Appendix from citation 20 (Section 2. Nonfatal outcome estimation, Step 1. Data sources, identification and extraction; Section 3a-d. Cause-specific estimation process)<br><br>See above for citations
5	Provide information on all included data sources and their main characteristics. For each data source used, report reference information or contact name/institution, population represented, data collection method, year(s) of data collection, sex and age range, diagnostic criteria or measurement method, and sample size, as relevant.	Interactive, online data source tool that provides metadata for data sources by component, geography, cause, or impairment has been developed.	Online data tools: http://ghdx.healthdata.org/global-burden-disease-study-2015
6	Identify and describe any categories of input data that have potentially important biases (e.g., based on characteristics listed in item 5).	Summary of known biases by cause included in methodological appendices of referenced papers.	1. Main text (Methods)<br><br>2. Appendix from citation 12 (Parts 1-3)

			3. Appendix from citation 20 (Section 3a-d. Cause-specific estimation process)<br><br>See above for citations
For data inputs that contribute to the analysis but were not synthesized as part of the study:			
7	Describe and give sources for any other data inputs.	Included in list of all data sources provided in submission materials for referenced papers as well as online data source tool.	Online data tools: http://ghdx.healthdata.org/global-burden-disease-study-2015
For all data inputs:			
8	Provide all data inputs in a file format from which data can be efficiently extracted (e.g., a spreadsheet as opposed to a PDF), including all relevant meta-data listed in item 5. For any data inputs that cannot be shared due to ethical or legal reasons, such as third-party ownership, provide a contact name or the name of the institution that retains the right to the data.	Downloads of input data will be available through online tools, including data visualization tools and data query tools. Input data not available in tools will be made available upon request.	Online data tools: http://ghdx.healthdata.org/global-burden-disease-study-2015
**Data analysis**			
9	Provide a conceptual overview of the data analysis method. A diagram may be helpful.	Flow diagrams of the overall methodological processes as well as cause-specific modelling processes have been provided.	Main text (Methods)
10	Provide a detailed description of all steps of the analysis, including mathematical formulae. This description should cover, as relevant, data cleaning, data pre-processing, data adjustments and weighting of data sources, and mathematical or statistical model(s).	Methodological write-up for the calculation of DALYs and HALE have been provided.	Main text (Methods)
11	Describe how candidate models were evaluated and how the final model(s) were selected.	Provided in the methodological write-ups within referenced appendix material.	1. Main text (Methods)<br><br>2. Appendix from citation 12 (Parts 2-3)<br><br>3. Appendix from citation 20 (Section 3a-d. Cause-specific estimation process)

			See above for citations
12	Provide the results of an evaluation of model performance, if done, as well as the results of any relevant sensitivity analysis.	Provided in the methodological write-ups within referenced appendix material.	1. Main text (Methods)<br>2. Appendix from citation 12 (Parts 2-3)<br>3. Appendix from citation 20 (Section 3a-d. Cause-specific estimation process)<br>See above for citations
13	Describe methods for calculating uncertainty of the estimates. State which sources of uncertainty were, and were not, accounted for in the uncertainty analysis.	Provided in the methodological write-ups within referenced appendix material.	1. Main text (Methods)<br>2. Appendix from citation 12 (Parts 2-3)<br>3. Appendix from citation 20 (Section 3a-d. Cause-specific estimation process)<br>See above for citations
14	State how analytic or statistical source code used to generate estimates can be accessed.	Access statement provided.	Online data tools: http://ghdx.healthdata.org/global-burden-disease-study-2015
**Results and Discussion**			
15	Provide published estimates in a file format from which data can be efficiently extracted.	GBD 2015 results will be made available through online data visualization tools, the Global Health Data Exchange, and the online data query tool (these tools are already available for GBD 2013 results).	Main text, appendix results section, and online data tools: http://ghdx.healthdata.org/global-burden-disease-study-2015
16	Report a quantitative measure of the uncertainty of the estimates (e.g. uncertainty intervals).	Uncertainty intervals are provided with all results.	Main text, appendix results section, and online data tools: http://ghdx.healthdata.org/global-burden-disease-study-2015

17	Interpret results in light of existing evidence. If updating a previous set of estimates, describe the reasons for changes in estimates.	Discussion of methodological changes between GBD rounds provided in the narrative of the papers and appendices of referenced papers.	Main text (Methods, Methods changes and data gaps)
18	Discuss limitations of the estimates. Include a discussion of any modelling assumptions or data limitations that affect interpretation of the estimates.	Discussion of limitations provided in the narrative of the main paper as well as in the methodological write-ups in the appendices of referenced papers.	Main text (Methods, Limitations)

Section 2. Supplementary Results

Appendix Table 2. Life expectancy at birth and HALE at birth and at age 65 by location for GBD years 1990 - 2015, both sexes combined.

Location	1990			2000			2005			2010			2015		
	Life expectancy	HALE		Life expectancy	HALE		Life expectancy	HALE		Life expectancy	HALE		Life expectancy	HALE	
	At birth	At birth	At age 65	At birth	At birth	At age 65	At birth	At birth	At age 65	At birth	At birth	At age 65	At birth	At birth	At age 65
Global	64.76 (64.49-65.01)	56.68 (54.31-58.75)	11.24 (10.27-12.08)	66.6 (66.4-66.8)	58.29 (55.84-60.38)	11.71 (10.72-12.57)	68.15 (67.95-68.36)	59.63 (57.12-61.83)	12.09 (11.06-13.0)	70.15 (69.9-70.42)	61.38 (58.8-63.66)	12.66 (11.59-13.62)	71.85 (71.46-72.19)	62.83 (60.2-65.19)	13.08 (11.98-14.07)
High SDI	74.58 (74.54-74.62)	65.4 (62.71-67.76)	12.74 (11.67-13.69)	75.76 (75.73-75.78)	66.39 (63.65-68.75)	13.35 (12.24-14.34)	76.75 (76.72-76.78)	67.23 (64.44-69.65)	13.9 (12.71-14.94)	78.45 (78.43-78.46)	68.64 (65.78-71.15)	14.63 (13.39-15.73)	79.29 (79.22-79.35)	69.32 (66.43-71.87)	14.97 (13.69-16.09)
High-middle SDI	69.13 (68.76-69.47)	60.92 (58.5-63.05)	11.53 (10.59-12.36)	70.97 (70.77-71.19)	62.57 (60.1-64.74)	11.99 (11.0-12.84)	72.16 (71.96-72.36)	63.61 (61.11-65.83)	12.37 (11.36-13.27)	74.03 (73.82-74.25)	65.22 (62.65-67.47)	13.06 (11.99-14.01)	75.8 (75.49-76.09)	66.69 (64.03-68.99)	13.64 (12.51-14.61)
Middle SDI	64.62 (64.08-65.14)	56.9 (54.62-58.91)	10.19 (9.33-10.96)	67.56 (67.17-67.93)	59.51 (57.17-61.52)	10.79 (9.92-11.56)	69.2 (68.92-69.48)	60.93 (58.55-63.06)	11.15 (10.21-11.97)	70.98 (70.67-71.29)	62.52 (60.08-64.72)	11.73 (10.75-12.59)	72.45 (72.06-72.89)	63.76 (61.23-65.96)	12.24 (11.25-13.15)
Low-middle SDI	57.19 (56.6-57.78)	49.39 (47.05-51.48)	9.2 (8.29-10.01)	59.71 (59.24-60.19)	51.6 (49.14-53.77)	9.53 (8.61-10.36)	61.41 (60.94-61.89)	53.08 (50.65-55.33)	9.83 (8.9-10.71)	63.44 (62.83-64.03)	54.95 (52.51-57.21)	10.18 (9.24-11.09)	65.69 (65.01-66.36)	56.96 (54.43-59.34)	10.54 (9.58-11.45)
Low SDI	50.64 (49.71-51.48)	43.67 (41.34-45.74)	8.48 (7.53-9.34)	52.24 (51.49-52.96)	45.05 (42.83-47.19)	8.68 (7.73-9.57)	55.34 (54.53-56.12)	47.75 (45.31-49.94)	9.09 (8.09-9.98)	58.57 (57.43-59.66)	50.66 (47.88-52.98)	9.52 (8.42-10.49)	60.7 (59.15-62.25)	52.63 (49.81-55.16)	9.72 (8.58-10.71)
High-income	76.03 (76.01-76.04)	66.71 (63.99-69.08)	13.21 (12.11-14.18)	78.17 (78.16-78.18)	68.45 (65.6-70.88)	14.14 (12.98-15.17)	79.28 (79.26-79.29)	69.35 (66.46-71.86)	14.75 (13.52-15.84)	80.32 (80.31-80.33)	70.21 (67.28-72.76)	15.29 (14.01-16.43)	80.79 (80.75-80.82)	70.59 (67.63-73.19)	15.49 (14.18-16.64)
High-income North America	75.62 (75.6-75.63)	65.69 (62.93-68.13)	13.09 (11.91-14.15)	77.19 (77.17-77.2)	66.9 (64.03-69.42)	13.52 (12.31-14.61)	77.93 (77.89-77.96)	67.39 (64.47-69.97)	13.92 (12.63-15.1)	79.1 (79.09-79.11)	68.22 (65.2-70.93)	14.43 (13.05-15.67)	79.41 (79.36-79.47)	68.48 (65.44-71.23)	14.6 (13.23-15.84)
Canada	77.43 (77.38-77.47)	67.67 (64.85-70.19)	13.6 (12.4-14.64)	79.24 (79.21-79.28)	69.02 (66.07-71.57)	14.2 (12.94-15.31)	80.22 (80.18-80.27)	69.88 (66.86-72.52)	14.82 (13.49-15.97)	81.34 (81.3-81.38)	70.82 (67.78-73.5)	15.48 (14.11-16.69)	81.7 (81.55-81.86)	71.08 (68.08-73.8)	15.59 (14.21-16.81)
Greenland	64.6 (64.02-65.26)	56.88 (54.65-58.94)	9.57 (8.7-10.37)	67.76 (67.21-68.32)	59.48 (57.11-61.66)	10.2 (9.26-11.06)	69.05 (68.5-69.61)	60.58 (58.17-62.77)	10.6 (9.63-11.45)	70.81 (70.27-71.3)	62.07 (59.63-64.29)	11.22 (10.2-12.13)	72.12 (71.39-72.81)	63.14 (60.59-65.46)	11.62 (10.56-12.58)
United States	75.39 (75.38-75.41)	65.46 (62.69-67.89)	13.01 (11.83-14.07)	76.94 (76.92-76.95)	66.65 (63.79-69.17)	13.41 (12.22-14.51)	77.65 (77.61-77.68)	67.1 (64.19-69.67)	13.8 (12.51-14.97)	78.82 (78.81-78.84)	67.91 (64.9-70.63)	14.28 (12.9-15.53)	79.12 (79.06-79.18)	68.16 (65.13-70.91)	14.46 (13.09-15.7)
Australasia	76.75 (76.7-76.79)	66.95 (64.19-69.42)	13.3 (12.23-14.28)	79.52 (79.48-79.56)	69.09 (66.13-71.69)	14.62 (13.43-15.68)	80.93 (80.89-80.97)	70.26 (67.26-72.91)	15.35 (14.11-16.46)	81.81 (81.78-81.85)	71.02 (68.01-73.74)	15.79 (14.49-16.94)	82.19 (82.12-82.27)	71.32 (68.26-74.07)	15.88 (14.56-17.08)
Australia	76.98 (76.93-77.03)	67.11 (64.33-69.6)	13.37 (12.29-14.35)	79.75 (79.71-79.79)	69.24 (66.26-71.86)	14.72 (13.52-15.79)	81.18 (81.13-81.22)	70.43 (67.43-73.09)	15.47 (14.22-16.6)	82.03 (81.99-82.07)	71.16 (68.14-73.91)	15.89 (14.58-17.05)	82.34 (82.26-82.43)	71.41 (68.36-74.17)	15.93 (14.61-17.13)
New Zealand	75.6 (75.5-75.7)	66.2 (63.49-68.59)	12.99 (11.94-13.92)	78.42 (78.33-78.51)	68.39 (65.54-70.92)	14.12 (12.96-15.15)	79.75 (79.66-79.85)	69.48 (66.54-72.08)	14.79 (13.58-15.87)	80.76 (80.67-80.84)	70.32 (67.38-72.98)	15.32 (14.03-16.42)	81.45 (81.24-81.64)	70.88 (67.91-73.61)	15.61 (14.31-16.78)
High-income Asia Pacific	77.43 (77.31-77.51)	68.94 (66.4-71.16)	14.07 (13.01-14.99)	80.04 (79.99-80.08)	71.14 (68.52-73.41)	15.47 (14.31-16.48)	81.3 (81.26-81.34)	72.27 (69.62-74.6)	16.13 (14.94-17.18)	82.24 (82.2-82.29)	73.11 (70.44-75.44)	16.61 (15.36-17.7)	82.69 (82.59-82.78)	73.46 (70.78-75.79)	16.76 (15.49-17.85)
Brunei	73.78 (73.45-74.16)	65.53 (63.08-67.63)	12.41 (11.38-13.29)	75.92 (75.63-76.2)	67.31 (64.85-69.5)	13.24 (12.21-14.18)	76.74 (76.43-77.02)	68.08 (65.61-70.37)	13.5 (12.49-14.47)	77.08 (76.77-77.35)	68.42 (65.87-70.65)	13.62 (12.56-14.59)	77.15 (76.68-77.62)	68.52 (65.97-70.77)	13.61 (12.56-14.59)
Japan	78.98 (78.96-79.0)	70.37 (67.83-72.58)	14.55 (13.47-15.5)	81.01 (80.99-81.03)	72.07 (69.45-74.36)	15.88 (14.7-16.9)	81.96 (81.94-81.98)	72.92 (70.3-75.25)	16.48 (15.27-17.53)	82.74 (82.72-82.75)	73.62 (70.96-75.95)	16.89 (15.64-17.99)	83.16 (83.11-83.2)	73.93 (71.27-76.29)	17.04 (15.76-18.16)
Singapore	74.89 (74.75-75.04)	67.35 (65.17-69.3)	12.6 (11.68-13.39)	78.32 (78.19-78.46)	70.35 (67.98-72.39)	13.93 (12.95-14.77)	80.04 (79.91-80.17)	71.99 (69.62-74.08)	15.02 (13.97-15.92)	81.3 (81.18-81.42)	73.17 (70.79-75.29)	15.72 (14.62-16.67)	81.97 (81.7-82.23)	73.71 (71.23-75.87)	16.08 (14.94-17.05)
South Korea	71.76 (71.42-72.01)	63.84 (61.47-65.94)	11.39 (10.47-12.19)	76.25 (76.09-76.41)	67.6 (65.04-69.84)	13.15 (12.07-14.09)	78.7 (78.54-78.87)	69.72 (67.06-72.02)	14.26 (13.11-15.27)	80.37 (80.21-80.53)	71.2 (68.48-73.55)	15.15 (13.91-16.21)	80.98 (80.65-81.3)	71.75 (69.0-74.08)	15.41 (14.13-16.48)
Western Europe	76.26 (76.25-76.28)	66.9 (64.11-69.32)	13.09 (12.02-14.04)	78.55 (78.53-78.56)	68.76 (65.85-71.28)	14.08 (12.92-15.12)	79.86 (79.85-79.87)	69.89 (66.93-72.46)	14.77 (13.53-15.85)	80.92 (80.91-80.93)	70.84 (67.86-73.43)	15.35 (14.07-16.47)	81.48 (81.43-81.53)	71.32 (68.32-73.93)	15.58 (14.26-16.71)
Andorra	81.7 (81.28-82.12)	71.29 (68.25-73.96)	15.79 (14.47-16.96)	83.72 (83.41-84.01)	72.97 (69.86-75.72)	16.83 (15.52-18.06)	84.28 (84.0-84.55)	73.49 (70.36-76.26)	17.14 (15.76-18.38)	84.71 (84.44-84.96)	73.9 (70.74-76.66)	17.34 (15.94-18.58)	84.82 (84.54-85.09)	73.94 (70.74-76.72)	17.35 (15.93-18.6)
Austria	75.7 (75.64-75.76)	66.39 (63.66-68.79)	12.86 (11.8-13.78)	78.18 (78.12-78.23)	68.4 (65.53-70.94)	13.92 (12.78-14.93)	79.53 (79.48-79.6)	69.51 (66.57-72.1)	14.61 (13.38-15.67)	80.54 (80.48-80.6)	70.38 (67.39-73.0)	15.14 (13.85-16.25)	81.29 (81.18-81.39)	71.05 (68.03-73.71)	15.44 (14.12-16.59)
Belgium	75.99 (75.91-76.08)	66.6 (63.86-69.03)	12.76 (11.68-13.72)	77.78 (77.7-77.86)	67.93 (65.05-70.45)	13.57 (12.38-14.62)	79.13 (79.05-79.2)	69.08 (66.13-71.64)	14.26 (13.0-15.36)	80.05 (79.97-80.13)	69.91 (66.95-72.54)	14.79 (13.49-15.92)	80.53 (80.21-80.84)	70.31 (67.37-73.01)	14.96 (13.66-16.16)
Cyprus	76.76 (76.58-76.96)	67.24 (64.44-69.68)	13.18 (12.07-14.17)	77.74 (77.58-77.91)	68.02 (65.19-70.5)	13.6 (12.52-14.62)	78.95 (78.82-79.09)	68.99 (66.07-71.53)	14.24 (13.06-15.31)	80.59 (80.43-80.75)	70.32 (67.3-72.94)	14.93 (13.66-16.06)	81.78 (81.56-81.98)	71.31 (68.24-74.02)	15.54 (14.19-16.74)
Denmark	75.08 (74.99-75.17)	65.92 (63.26-68.24)	12.59 (11.55-13.49)	77.09 (77.0-77.16)	67.53 (64.75-69.97)	13.18 (12.11-14.12)	78.33 (78.24-78.41)	68.52 (65.67-71.02)	13.78 (12.63-14.78)	79.37 (79.29-79.46)	69.46 (66.59-71.99)	14.33 (13.15-15.37)	80.38 (80.16-80.6)	70.32 (67.4-72.89)	14.83 (13.59-15.92)
Finland	75.12 (75.03-75.22)	65.8 (63.02-68.22)	12.51 (11.42-13.43)	77.83 (77.73-77.92)	67.97 (65.07-70.56)	13.73 (12.55-14.76)	79.05 (78.95-79.14)	68.79 (65.75-71.47)	14.49 (13.23-15.6)	80.04 (79.94-80.13)	69.69 (66.62-72.36)	14.93 (13.61-16.06)	80.93 (80.7-81.17)	70.5 (67.4-73.1)	15.31 (13.98-16.48)
France	77.16 (77.13-77.19)	67.64 (64.75-70.13)	14.14 (12.94-15.18)	79.08 (79.05-79.11)	69.25 (66.33-71.8)	14.88 (13.64-15.98)	80.42 (80.4-80.45)	70.39 (67.37-73.0)	15.58 (14.26-16.74)	81.38 (81.36-81.41)	71.22 (68.18-73.83)	16.13 (14.73-17.33)	81.82 (81.67-81.97)	71.6 (68.54-74.22)	16.24 (14.81-17.46)
Germany	75.42 (75.4-75.45)	66.06 (63.32-68.48)	12.61 (11.57-13.53)	78.12 (78.09-78.14)	68.27 (65.41-70.8)	13.81 (12.67-14.8)	79.41 (79.38-79.44)	69.36 (66.44-71.94)	14.46 (13.27-15.49)	80.26 (80.24-80.29)	70.15 (67.22-72.72)	14.91 (13.67-15.98)	80.79 (80.68-80.88)	70.6 (67.63-73.22)	15.12 (13.86-16.2)
Greece	77.05 (76.97-77.12)	67.76 (64.99-70.15)	13.18 (12.12-14.11)	78.58 (78.5-78.65)	69.07 (66.22-71.48)	13.98 (12.84-14.97)	79.56 (79.49-79.62)	69.91 (67.06-72.4)	14.6 (13.41-15.65)	80.43 (80.37-80.49)	70.68 (67.77-73.19)	15.2 (13.98-16.28)	80.96 (80.75-81.16)	71.13 (68.24-73.64)	15.38 (14.11-16.47)
Iceland	78.25 (78.03-78.44)	68.72 (65.89-71.15)	13.89 (12.72-14.9)	80.46 (80.2-80.7)	70.48 (67.58-72.99)	14.85 (13.6-15.94)	81.65 (81.41-81.89)	71.45 (68.46-74.06)	15.46 (14.17-16.61)	82.81 (82.53-83.07)	72.51 (69.52-75.17)	16.13 (14.78-17.34)	83.34 (83.08-83.61)	72.95 (69.88-75.66)	16.39 (15.02-17.61)
Ireland	76.27 (75.9-76.62)	66.83 (63.99-69.31)	13.15 (12.03-14.18)	77.84 (77.5-78.19)	68.08 (65.2-70.63)	13.8 (12.66-14.86)	79.74 (79.56-79.95)	69.64 (66.64-72.25)	14.62 (13.4-15.71)	81.05 (80.78-81.31)	70.78 (67.72-73.43)	15.33 (14.06-16.5)	81.72 (81.38-82.07)	71.31 (68.18-73.97)	15.64 (14.31-16.83)
Israel	76.66 (76.54-76.78)	67.41 (64.65-69.78)	13.13 (12.03-14.07)	78.61 (78.51-78.71)	68.87 (66.05-71.35)	13.92 (12.74-14.96)	79.75 (79.63-79.85)	69.84 (66.98-72.34)	14.53 (13.31-15.59)	81.58 (81.46-81.7)	71.37 (68.35-73.98)	15.47 (14.18-16.61)	82.06 (81.85-82.25)	71.75 (68.74-74.36)	15.64 (14.31-16.8)
Italy	76.99 (76.96-77.02)	67.51 (64.74-69.98)	13.31 (12.17-14.29)	79.58 (79.55-79.6)	69.57 (66.64-72.14)	14.37 (13.14-15.47)	81.12 (81.09-81.15)	70.93 (67.97-73.53)	15.18 (13.88-16.31)	81.93 (81.9-81.96)	71.72 (68.73-74.37)	15.61 (14.28-16.77)	82.18 (82.06-82.3)	71.9 (68.85-74.53)	15.66 (14.32-16.83)
Luxembourg	75.18 (75.02-75.35)	65.42 (62.59-67.9)	12.33 (11.26-13.3)	78.51 (78.34-78.67)	68.19 (65.17-70.81)	13.79 (12.61-14.87)	79.97 (79.81-80.13)	69.46 (66.4-72.14)	14.52 (13.27-15.67)	81.34 (81.18-81.49)	70.65 (67.54-73.36)	15.17 (13.85-16.36)	82.15 (81.95-82.37)	71.35 (68.2-74.12)	15.61 (14.21-16.83)
Malta	77.06 (76.79-77.35)	67.42 (64.57-69.87)	13.1 (12.01-14.08)	79.15 (78.83-79.5)	69.13 (66.17-71.75)	14.23 (13.05-15.31)	80.67 (80.4-80.95)	70.41 (67.39-73.04)	15.11 (13.86-16.23)	81.3 (81.11-81.51)	71.0 (67.98-73.67)	15.46 (14.2-16.61)	82.09 (81.85-82.35)	71.6 (68.52-74.28)	15.82 (14.5-16.99)
Netherlands	77.05 (76.97-77.12)	67.38 (64.59-69.87)	13.01 (11.91-14.01)	78.1 (78.03-78.18)	68.22 (65.36-70.74)	13.39 (12.24-14.41)	79.57 (79.5-79.63)	69.44 (66.49-72.04)	14.14 (12.93-15.2)	80.8 (80.73-80.87)	70.51 (67.48-73.11)	14.84 (13.54-15.93)	81.31 (81.08-81.56)	70.94 (67.97-73.59)	15.07 (13.76-16.21)
Norway	76.63 (76.5-76.76)	67.02 (64.25-69.45)	13.05 (12.0-14.0)	78.57 (78.45-78.69)	68.5 (65.61-71.1)	13.83 (12.68-14.89)	80.17 (80.05-80.28)	69.79 (66.79-72.41)	14.66 (13.45-15.74)	81.07 (80.96-81.18)	70.58 (67.56-73.25)	15.11 (13.85-16.22)	82.04 (81.86-82.23)	71.51 (68.48-74.24)	15.57 (14.27-16.74)
Portugal	74.18 (74.13-74.24)	64.83 (62.03-67.2)	12.32 (11.28-13.24)	76.77 (76.72-76.83)	67.05 (64.19-69.48)	13.38 (12.26-14.37)	78.43 (78.37-78.49)	68.51 (65.58-71.02)	14.11 (12.92-15.16)	79.87 (79.82-79.92)	69.78 (66.79-72.33)	14.79 (13.54-15.89)	80.81 (80.69-80.95)	70.56 (67.55-73.19)	15.22 (13.92-16.35)
Spain	76.92 (76.88-76.95)	67.56 (64.75-69.99)	13.55 (12.41-14.55)	79.15 (79.12-79.18)	69.51 (66.6-72.03)	14.46 (13.26-15.54)	80.27 (80.24-80.3)	70.55 (67.61-73.08)	15.03 (13.77-16.15)	81.82 (81.79-81.84)	71.91 (68.93-74.47)	15.85 (14.51-17.01)	82.6 (82.52-82.68)	72.63 (69.7-75.23)	16.24 (14.88-17.39)
Sweden	77.63 (77.55-77.72)	68.41 (65.68-70.78)	13.54 (12.48-14.51)	79.72 (79.63-79.81)	69.93 (67.09-72.46)	14.26 (13.09-15.31)	80.55 (80.47-80.64)	70.64 (67.76-73.18)	14.72 (13.49-15.79)	81.61 (81.53-81.69)	71.51 (68.6-74.1)	15.28 (14.03-16.39)	82.13 (81.96-82.32)	71.9 (68.99-74.5)	15.52 (14.23-16.67)
Switzerland	77.51 (77.4-77.6)	67.5 (64.54-70.05)	13.6 (12.42-14.65)	79.84 (79.75-79.93)	69.51 (66.45-72.16)	14.65 (13.4-15.75)	81.29 (81.2-81.39)	70.77 (67.66-73.46)	15.38 (14.04-16.52)	82.3 (82.21-82.39)	71.74 (68.6-74.39)	15.88 (14.52-17.04)	82.98 (82.74-83.22)	72.41 (69.28-75.14)	16.28 (14.88-17.47)
United Kingdom	75.72 (75.7-75.75)	66.65 (63.98-68.96)	12.67 (11.69-13.52)	77.79 (77.76-77.81)	68.3 (65.48-70.71)	13.62 (12.57-14.55)	78.99 (78.96-79.02)	69.29 (66.38-71.76)	14.36 (13.25-15.34)	80.23 (80.2-80.25)	70.39 (67.49-72.89)	15.13 (13.95-16.16)	80.95 (80.87-81.03)	71.0 (68.09-73.54)	15.45 (14.24-16.5)
England	75.94 (75.92-75.97)	66.9 (64.24-69.2)	12.78 (11.8-13.64)	78.03 (78.01-78.06)	68.58 (65.76-70.99)	13.75 (12.69-14.68)	79.26 (79.23-79.29)	69.59 (66.7-72.07)	14.51 (13.4-15.49)	80.52 (80.5-80.55)	70.72 (67.84-73.22)	15.29 (14.12-16.34)	81.22 (81.14-81.3)	71.31 (68.41-73.85)	15.6 (14.4-16.66)
Northern Ireland	74.94 (74.79-75.11)	66.18 (63.62-68.41)	12.32 (11.39-13.14)	77.57 (77.41-77.72)	68.21 (65.46-70.65)	13.55 (12.49-14.49)	78.38 (78.23-78.53)	68.81 (65.99-71.25)	14.12 (13.03-15.1)	79.1 (78.92-79.27)	69.38 (66.57-71.87)	14.65 (13.47-15.66)	80.15 (79.84-80.43)	70.39 (67.56-72.83)	15.07 (13.89-16.12)
Scotland	73.99 (73.89-74.07)	64.75 (62.05-67.1)	11.82 (10.87-12.65)	75.83 (75.74-75.91)	66.17 (63.36-68.66)	12.7 (11.65-13.63)	76.98 (76.89-77.07)	67.06 (64.15-69.66)	13.36 (12.27-14.34)	78.26 (78.18-78.34)	68.19 (65.23-70.78)	14.06 (12.89-15.07)	79.23 (78.98-79.47)	68.96 (66.04-71.58)	14.51 (13.31-15.61)
Wales	75.65 (75.55-75.74)	66.26 (63.53-68.67)	12.49 (11.5-13.38)	77.39 (77.29-77.48)	67.61 (64.71-70.1)	13.25 (12.16-14.21)	78.46 (78.36-78.55)	68.46 (65.54-71.01)	13.88 (12.76-14.88)	79.51 (79.4-79.6)	69.33 (66.33-71.95)	14.56 (13.36-15.62)	80.07 (79.86-80.29)	69.79 (66.86-72.47)	14.79 (13.6-15.89)
Southern Latin America	72.67 (72.63-72.71)	64.43 (61.97-66.54)	12.64 (11.68-13.47)	74.97 (74.94-75.01)	66.33 (63.74-68.55)	13.44 (12.42-14.33)	76.0 (75.97-76.04)	67.25 (64.66-69.51)	13.78 (12.72-14.7)	76.55 (76.51-76.6)	67.74 (65.11-70.01)	14.0 (12.92-14.93)	77.26 (77.09-77.43)	68.34 (65.64-70.67)	14.28 (13.2-15.24)

Appendix Table 2. Life expectancy at birth and HALE at birth and at age 65 by location for GBD years 1990 - 2015, both sexes combined.

	1990			2000			2005			2010			2015		
Location	Life expectancy	HALE		Life expectancy	HALE		Life expectancy	HALE		Life expectancy	HALE		Life expectancy	HALE	
	At birth	At birth	At age 65	At birth	At birth	At age 65	At birth	At birth	At age 65	At birth	At birth	At age 65	At birth	At birth	At age 65
Argentina	72.45 (72.4-72.5)	64.2 (61.74-66.32)	12.68 (11.73-13.51)	74.2 (74.15-74.24)	65.63 (63.05-67.84)	13.23 (12.22-14.11)	75.26 (75.22-75.31)	66.58 (64.03-68.83)	13.56 (12.5-14.47)	75.84 (75.79-75.88)	67.13 (64.53-69.38)	13.77 (12.72-14.68)	76.45 (76.26-76.65)	67.64 (64.95-69.94)	14.0 (12.92-14.96)
Chile	73.13 (73.04-73.21)	64.9 (62.46-67.0)	12.5 (11.55-13.33)	77.32 (77.24-77.4)	68.4 (65.78-70.72)	14.24 (13.14-15.18)	78.22 (78.14-78.3)	69.19 (66.51-71.5)	14.58 (13.46-15.53)	78.5 (78.38-78.62)	69.38 (66.66-71.75)	14.69 (13.55-15.66)	79.36 (78.96-79.77)	70.15 (67.51-72.55)	15.07 (13.93-16.1)
Uruguay	73.02 (72.88-73.14)	64.84 (62.4-66.93)	12.58 (11.62-13.4)	74.64 (74.52-74.76)	66.15 (63.59-68.29)	13.21 (12.2-14.08)	75.52 (75.4-75.63)	66.95 (64.41-69.14)	13.52 (12.47-14.42)	76.21 (76.06-76.38)	67.58 (64.98-69.84)	13.76 (12.71-14.68)	76.76 (76.45-77.1)	68.01 (65.36-70.24)	14.0 (12.92-14.95)
Central Europe, Eastern Europe, and Central Asia	69.19 (69.12-69.27)	60.64 (58.08-62.86)	11.13 (10.14-12.0)	67.61 (67.5-67.71)	59.41 (56.96-61.55)	10.76 (9.8-11.6)	68.15 (68.03-68.26)	59.98 (57.55-62.1)	10.96 (9.98-11.82)	70.96 (70.84-71.07)	62.36 (59.8-64.6)	11.79 (10.73-12.7)	72.68 (72.48-72.87)	63.76 (61.11-66.07)	12.44 (11.33-13.4)
Eastern Europe	69.5 (69.43-69.57)	60.64 (58.04-62.92)	11.12 (10.11-12.01)	65.99 (65.89-66.08)	57.83 (55.39-59.94)	10.28 (9.34-11.1)	65.99 (65.88-66.09)	57.97 (55.61-60.03)	10.38 (9.43-11.21)	69.53 (69.48-69.58)	60.93 (58.39-63.16)	11.31 (10.29-12.21)	71.29 (71.05-71.51)	62.35 (59.7-64.7)	11.96 (10.87-12.9)
Belarus	70.46 (70.11-70.8)	61.89 (59.4-64.18)	11.62 (10.6-12.53)	68.13 (67.83-68.42)	60.12 (57.77-62.22)	10.71 (9.8-11.52)	68.66 (68.39-68.9)	60.62 (58.3-62.73)	10.89 (9.93-11.72)	70.21 (69.94-70.48)	61.93 (59.51-64.11)	11.35 (10.36-12.22)	70.96 (70.47-71.45)	62.54 (60.05-64.85)	11.42 (10.41-12.35)
Estonia	70.07 (69.93-70.21)	61.41 (58.88-63.72)	11.24 (10.26-12.13)	70.43 (70.28-70.58)	61.86 (59.32-64.1)	11.51 (10.47-12.39)	72.52 (72.38-72.65)	63.75 (61.16-66.03)	12.2 (11.11-13.15)	76.37 (76.22-76.5)	67.01 (64.24-69.43)	13.67 (12.48-14.71)	77.77 (77.49-78.06)	68.26 (65.52-70.73)	14.01 (12.81-15.11)
Latvia	69.58 (69.46-69.72)	60.98 (58.45-63.2)	11.13 (10.11-12.02)	70.51 (70.39-70.63)	61.92 (59.39-64.12)	11.7 (10.66-12.63)	71.09 (70.97-71.21)	62.58 (60.08-64.8)	11.79 (10.74-12.71)	73.95 (73.83-74.06)	65.0 (62.42-67.32)	12.78 (11.65-13.76)	75.38 (75.05-75.7)	66.24 (63.58-68.63)	13.26 (12.07-14.3)
Lithuania	71.45 (71.37-71.54)	62.55 (59.96-64.88)	11.92 (10.84-12.88)	72.05 (71.97-72.14)	63.12 (60.45-65.45)	12.27 (11.15-13.25)	71.67 (71.59-71.75)	62.95 (60.35-65.22)	12.31 (11.17-13.3)	73.85 (73.76-73.94)	64.78 (62.12-67.13)	12.96 (11.77-13.98)	75.21 (75.02-75.41)	65.92 (63.18-68.32)	13.47 (12.25-14.56)
Moldova	68.15 (67.78-68.48)	59.83 (57.38-61.93)	10.61 (9.69-11.42)	68.57 (68.11-68.92)	60.36 (57.96-62.51)	10.68 (9.74-11.5)	69.53 (69.23-69.78)	61.42 (59.0-63.55)	10.6 (9.7-11.39)	70.5 (70.23-70.72)	62.36 (59.96-64.56)	11.01 (10.08-11.83)	73.91 (73.52-74.25)	65.09 (62.55-67.4)	12.34 (11.27-13.28)
Russia	69.24 (69.21-69.27)	60.25 (57.58-62.58)	11.03 (10.0-11.94)	65.13 (65.04-65.22)	56.93 (54.48-59.03)	10.06 (9.1-10.88)	65.12 (64.98-65.25)	57.06 (54.69-59.14)	10.18 (9.23-11.02)	68.94 (68.91-68.97)	60.23 (57.66-62.52)	11.18 (10.13-12.1)	71.0 (70.67-71.33)	61.92 (59.21-64.29)	11.93 (10.81-12.91)
Ukraine	70.02 (69.74-70.29)	61.43 (58.96-63.61)	11.22 (10.25-12.08)	67.3 (66.95-67.59)	59.31 (56.94-61.48)	10.56 (9.65-11.37)	67.11 (66.85-67.33)	59.31 (57.05-61.33)	10.56 (9.64-11.36)	70.54 (70.31-70.71)	62.2 (59.78-64.36)	11.4 (10.42-12.26)	71.45 (70.92-71.95)	62.9 (60.41-65.13)	11.84 (10.82-12.74)
Central Europe	70.99 (70.94-71.03)	62.6 (60.13-64.79)	11.11 (10.16-11.95)	72.91 (72.88-72.95)	64.29 (61.74-66.53)	11.7 (10.7-12.59)	74.18 (74.15-74.21)	65.38 (62.78-67.65)	12.21 (11.16-13.14)	75.51 (75.48-75.54)	66.57 (63.94-68.89)	12.77 (11.68-13.74)	76.96 (76.82-77.1)	67.74 (65.01-70.08)	13.4 (12.23-14.43)
Albania	73.25 (72.82-73.65)	64.11 (61.41-66.46)	12.5 (11.4-13.44)	74.65 (74.22-75.02)	65.33 (62.65-67.77)	12.89 (11.8-13.88)	76.16 (75.72-76.57)	66.78 (64.02-69.16)	13.57 (12.43-14.61)	77.19 (76.48-77.75)	67.84 (65.03-70.32)	13.84 (12.59-14.96)	78.01 (77.37-78.65)	68.55 (65.62-71.07)	13.95 (12.68-15.16)
Bosnia and Herzegovina	72.68 (72.39-72.99)	64.17 (61.66-66.37)	12.14 (11.13-13.0)	75.75 (75.58-75.92)	66.49 (63.81-68.83)	12.95 (11.86-13.92)	77.02 (76.9-77.16)	67.56 (64.8-70.0)	13.41 (12.25-14.43)	77.9 (77.78-78.03)	68.32 (65.52-70.78)	13.73 (12.52-14.78)	78.92 (78.67-79.17)	69.1 (66.26-71.6)	14.2 (12.97-15.31)
Bulgaria	71.37 (71.28-71.45)	63.01 (60.57-65.21)	10.86 (9.91-11.69)	71.58 (71.48-71.66)	63.25 (60.76-65.41)	11.0 (10.04-11.82)	72.55 (72.47-72.64)	64.14 (61.68-66.32)	11.38 (10.39-12.24)	73.74 (73.65-73.83)	65.19 (62.67-67.44)	11.97 (10.96-12.88)	74.81 (74.42-75.19)	66.07 (63.48-68.33)	12.36 (11.3-13.32)
Croatia	72.64 (72.53-72.74)	63.86 (61.25-66.14)	11.35 (10.34-12.22)	74.16 (74.07-74.26)	65.29 (62.69-67.6)	11.74 (10.73-12.64)	75.65 (75.55-75.74)	66.54 (63.91-68.91)	12.4 (11.33-13.36)	76.74 (76.66-76.84)	67.48 (64.73-69.86)	12.86 (11.72-13.85)	77.84 (77.59-78.09)	68.35 (65.54-70.76)	13.34 (12.16-14.35)
Czech Republic	71.77 (71.7-71.84)	63.39 (60.87-65.54)	10.61 (9.68-11.43)	75.03 (74.96-75.1)	66.12 (63.46-68.43)	12.0 (10.96-12.92)	76.21 (76.15-76.28)	67.07 (64.36-69.44)	12.54 (11.43-13.53)	77.51 (77.44-77.58)	68.18 (65.42-70.61)	13.2 (12.03-14.2)	78.82 (78.68-78.95)	69.21 (66.38-71.72)	13.73 (12.51-14.82)
Hungary	69.49 (69.42-69.58)	61.24 (58.86-63.36)	10.64 (9.69-11.48)	71.77 (71.69-71.84)	63.23 (60.75-65.43)	11.46 (10.44-12.37)	73.06 (72.99-73.13)	64.29 (61.74-66.57)	11.9 (10.82-12.85)	74.62 (74.55-74.71)	65.64 (63.01-67.98)	12.45 (11.34-13.44)	76.74 (76.41-77.06)	67.36 (64.64-69.72)	13.37 (12.19-14.41)
Macedonia	71.69 (71.52-71.86)	63.33 (60.9-65.49)	11.28 (10.29-12.15)	72.9 (72.76-73.02)	64.6 (62.18-66.79)	10.95 (10.0-11.78)	73.85 (73.72-73.97)	65.44 (62.96-67.65)	11.3 (10.33-12.15)	75.17 (75.03-75.3)	66.57 (64.05-68.84)	11.85 (10.83-12.76)	76.5 (76.0-77.01)	67.59 (64.95-69.95)	12.8 (11.69-13.82)
Montenegro	74.57 (73.93-75.18)	65.65 (63.04-68.09)	12.99 (11.91-13.99)	73.25 (73.01-73.49)	64.64 (62.14-66.89)	12.04 (11.02-12.94)	74.98 (74.79-75.17)	66.13 (63.54-68.48)	12.59 (11.5-13.57)	76.53 (76.27-76.81)	67.48 (64.82-69.84)	13.31 (12.19-14.33)	77.17 (76.77-77.64)	67.99 (65.24-70.4)	13.45 (12.27-14.51)
Poland	70.94 (70.9-70.97)	62.59 (60.14-64.76)	11.13 (10.17-11.98)	73.79 (73.75-73.83)	65.05 (62.48-67.31)	12.12 (11.07-13.04)	75.07 (75.04-75.11)	66.13 (63.48-68.4)	12.82 (11.7-13.78)	76.23 (76.19-76.26)	67.17 (64.51-69.5)	13.36 (12.23-14.37)	77.58 (77.41-77.77)	68.29 (65.55-70.66)	13.91 (12.7-14.97)
Romania	69.99 (69.93-70.05)	61.67 (59.23-63.83)	11.27 (10.33-12.1)	71.08 (71.02-71.14)	62.7 (60.25-64.9)	11.53 (10.57-12.38)	72.4 (72.34-72.46)	63.93 (61.45-66.15)	11.88 (10.9-12.76)	73.69 (73.63-73.75)	65.12 (62.62-67.37)	12.28 (11.26-13.19)	75.21 (74.87-75.55)	66.38 (63.77-68.64)	12.84 (11.79-13.78)
Serbia	72.55 (72.09-72.98)	63.96 (61.47-66.27)	11.67 (10.67-12.58)	72.13 (71.92-72.34)	63.76 (61.32-65.92)	10.83 (9.88-11.65)	73.25 (73.08-73.42)	64.72 (62.23-66.94)	11.16 (10.17-12.03)	74.84 (74.79-74.9)	66.06 (63.46-68.35)	11.91 (10.86-12.83)	76.19 (76.02-76.35)	67.08 (64.41-69.43)	12.79 (11.67-13.78)
Slovakia	70.95 (70.86-71.04)	62.69 (60.26-64.84)	10.84 (9.91-11.66)	73.27 (73.18-73.35)	64.62 (62.06-66.82)	11.55 (10.56-12.44)	74.26 (74.17-74.35)	65.45 (62.87-67.74)	11.97 (10.94-12.9)	75.66 (75.57-75.74)	66.67 (63.99-69.01)	12.62 (11.54-13.59)	77.56 (77.39-77.73)	68.17 (65.41-70.58)	13.51 (12.35-14.59)
Slovenia	73.65 (73.55-73.76)	64.77 (62.15-67.1)	11.86 (10.81-12.79)	76.06 (75.96-76.15)	66.81 (64.11-69.24)	12.84 (11.69-13.85)	77.56 (77.47-77.66)	68.1 (65.31-70.54)	13.5 (12.31-14.55)	79.5 (79.4-79.6)	69.8 (66.93-72.32)	14.43 (13.17-15.53)	80.93 (80.77-81.09)	70.98 (68.02-73.59)	15.12 (13.79-16.29)
Central Asia	67.1 (66.85-67.36)	59.16 (56.8-61.28)	11.31 (10.34-12.15)	66.52 (66.23-66.79)	58.79 (56.44-60.84)	10.75 (9.84-11.54)	67.5 (67.17-67.81)	59.72 (57.42-61.73)	10.8 (9.87-11.6)	69.56 (69.19-69.88)	61.49 (59.08-63.59)	11.3 (10.33-12.15)	71.35 (70.85-71.78)	62.94 (60.44-65.19)	11.89 (10.87-12.79)
Armenia	70.08 (69.62-70.52)	61.51 (59.05-63.75)	11.72 (10.7-12.63)	71.26 (70.82-71.68)	62.71 (60.26-65.01)	11.35 (10.38-12.24)	71.77 (71.33-72.19)	63.23 (60.81-65.44)	11.4 (10.45-12.29)	73.01 (72.59-73.44)	64.32 (61.81-66.56)	12.01 (10.95-12.97)	74.71 (74.21-75.25)	65.72 (63.07-68.13)	12.57 (11.46-13.54)
Azerbaijan	65.56 (64.84-66.2)	57.93 (55.64-60.02)	10.97 (10.04-11.79)	66.46 (65.78-67.09)	58.78 (56.48-60.88)	10.55 (9.64-11.36)	68.22 (67.56-68.85)	60.34 (57.88-62.61)	10.86 (9.91-11.71)	70.1 (69.29-70.81)	61.97 (59.5-64.3)	11.08 (10.12-11.94)	72.92 (72.13-73.7)	64.21 (61.61-66.61)	12.44 (11.33-13.41)
Georgia	69.89 (69.3-70.44)	62.0 (59.52-64.11)	11.87 (10.92-12.75)	71.39 (70.77-72.0)	63.39 (61.04-65.61)	11.94 (10.99-12.82)	71.76 (71.19-72.36)	63.68 (61.28-65.78)	11.91 (10.91-12.79)	72.2 (71.58-72.81)	63.99 (61.57-66.22)	12.19 (11.2-13.11)	72.87 (72.17-73.58)	64.43 (61.83-66.73)	12.31 (11.22-13.27)
Kazakhstan	68.0 (67.76-68.25)	59.69 (57.25-61.85)	11.25 (10.24-12.09)	64.93 (64.56-65.24)	57.25 (54.98-59.28)	10.17 (9.28-10.95)	65.26 (64.9-65.58)	57.61 (55.36-59.63)	10.2 (9.29-10.98)	68.48 (68.18-68.76)	60.32 (57.9-62.44)	10.82 (9.85-11.64)	70.18 (69.31-71.04)	61.74 (59.19-64.22)	11.42 (10.37-12.41)
Kyrgyzstan	65.97 (65.53-66.43)	58.1 (55.8-60.11)	11.18 (10.22-12.01)	65.91 (65.45-66.34)	58.25 (56.06-60.33)	10.69 (9.8-11.51)	66.48 (66.04-66.94)	58.88 (56.7-60.92)	10.38 (9.53-11.18)	67.91 (67.48-68.32)	60.14 (57.9-62.19)	10.72 (9.82-11.51)	69.78 (69.22-70.34)	61.71 (59.21-63.89)	11.31 (10.33-12.16)
Mongolia	62.3 (61.64-62.91)	55.11 (53.01-57.01)	10.02 (9.16-10.81)	62.47 (61.84-63.04)	55.54 (53.51-57.34)	9.29 (8.51-10.0)	63.63 (63.03-64.2)	56.64 (54.61-58.5)	9.62 (8.81-10.36)	65.21 (64.57-65.87)	57.96 (55.86-59.99)	9.9 (9.06-10.69)	67.1 (66.38-67.77)	59.54 (57.25-61.71)	10.4 (9.49-11.25)
Tajikistan	65.26 (64.55-65.95)	57.58 (55.26-59.76)	11.47 (10.5-12.35)	65.8 (65.18-66.42)	58.17 (55.87-60.24)	10.74 (9.84-11.55)	67.97 (67.39-68.52)	60.17 (57.85-62.28)	10.66 (9.75-11.45)	70.13 (69.55-70.68)	62.03 (59.54-64.17)	11.33 (10.36-12.19)	72.36 (71.65-73.04)	63.84 (61.26-66.2)	12.37 (11.35-13.31)
Turkmenistan	63.87 (62.94-64.77)	56.6 (54.05-58.77)	11.03 (10.12-11.83)	63.74 (62.37-64.86)	56.57 (54.21-58.76)	10.63 (9.74-11.38)	65.76 (64.47-66.88)	58.36 (55.98-60.53)	10.96 (10.05-11.77)	68.06 (66.94-69.0)	60.37 (57.95-62.54)	11.46 (10.49-12.33)	70.01 (69.02-70.78)	61.97 (59.51-64.21)	11.91 (10.92-12.83)
Uzbekistan	67.77 (67.18-68.33)	59.8 (57.27-62.0)	11.23 (10.25-12.07)	67.58 (66.94-68.18)	59.76 (57.35-61.91)	10.77 (9.86-11.58)	68.49 (67.74-69.2)	60.65 (58.18-62.85)	10.85 (9.91-11.67)	70.22 (69.47-70.9)	62.16 (59.65-64.32)	11.49 (10.51-12.37)	71.79 (71.08-72.47)	63.44 (60.88-65.7)	11.94 (10.93-12.84)
Latin America and Caribbean	69.28 (69.11-69.44)	60.79 (58.29-63.02)	12.16 (11.1-13.1)	72.09 (71.92-72.27)	63.16 (60.53-65.49)	12.67 (11.58-13.65)	73.44 (73.25-73.63)	64.31 (61.63-66.67)	13.06 (11.92-14.07)	73.16 (71.98-74.18)	64.01 (61.3-66.52)	13.27 (12.1-14.3)	75.2 (74.81-75.55)	65.8 (63.0-68.16)	13.46 (12.25-14.51)
Central Latin America	71.19 (71.04-71.35)	62.63 (60.13-64.84)	12.96 (11.83-13.96)	73.81 (73.66-73.97)	64.88 (62.24-67.2)	13.52 (12.34-14.56)	74.74 (74.56-74.91)	65.7 (63.04-68.0)	13.62 (12.44-14.66)	75.36 (75.12-75.59)	66.23 (63.52-68.57)	13.81 (12.6-14.87)	76.03 (75.68-76.38)	66.77 (64.0-69.15)	13.82 (12.59-14.89)
Colombia	71.5 (71.33-71.66)	62.96 (60.43-65.19)	12.89 (11.79-13.85)	73.34 (73.16-73.5)	64.57 (61.97-66.82)	13.2 (12.09-14.19)	75.14 (74.94-75.33)	66.12 (63.43-68.42)	13.47 (12.34-14.46)	76.4 (76.09-76.68)	67.19 (64.45-69.55)	13.93 (12.76-14.96)	78.0 (77.54-78.42)	68.45 (65.62-70.88)	14.5 (13.24-15.63)
Costa Rica	76.58 (76.34-76.81)	67.53 (64.82-69.9)	13.73 (12.58-14.73)	77.69 (77.4-77.93)	68.39 (65.61-70.82)	14.2 (13.01-15.26)	78.98 (78.73-79.19)	69.37 (66.49-71.87)	14.86 (13.6-15.99)	79.8 (79.56-80.01)	70.07 (67.18-72.55)	15.35 (14.03-16.49)	80.32 (79.91-80.69)	70.46 (67.51-72.94)	15.48 (14.16-16.64)
El Salvador	67.72 (67.15-68.29)	59.3 (56.88-61.55)	12.47 (11.41-13.46)	71.94 (71.4-72.46)	62.91 (60.27-65.3)	13.45 (12.29-14.52)	73.03 (72.55-73.48)	63.83 (61.13-66.2)	13.75 (12.55-14.82)	74.09 (73.5-74.62)	64.78 (62.03-67.23)	14.15 (12.89-15.26)	74.95 (74.13-75.77)	65.53 (62.74-68.1)	14.32 (13.03-15.48)
Guatemala	64.62 (64.33-64.92)	56.5 (54.11-58.63)	11.53 (10.51-12.45)	68.77 (68.51-69.03)	59.99 (57.43-62.32)	12.56 (11.41-13.57)	69.83 (69.51-70.15)	60.83 (58.18-63.21)	12.76 (11.55-13.81)	71.2 (70.81-71.54)	62.06 (59.3-64.4)	12.82 (11.62-13.87)	72.57 (71.25-73.86)	63.24 (60.28-65.99)	13.05 (11.73-14.27)
Honduras	70.33 (69.73-70.97)	61.48 (58.84-63.79)	13.45 (12.28-14.49)	70.45 (68.67-72.04)	61.97 (58.99-64.68)	12.0 (10.75-13.16)	71.21 (69.09-73.11)	62.76 (59.67-65.57)	12.06 (10.76-13.3)	72.03 (69.89-73.95)	63.55 (60.68-66.28)	12.26 (11.01-13.49)	73.01 (70.88-75.03)	64.36 (61.5-67.13)	12.47 (11.25-13.68)
Mexico	71.51 (71.22-71.79)	63.0 (60.51-65.21)	13.15 (12.0-14.18)	74.59 (74.33-74.85)	65.83 (63.29-68.18)	13.89 (12.67-14.97)	75.13 (74.85-75.38)	66.36 (63.82-68.61)	13.79 (12.59-14.85)	75.43 (75.14-75.68)	66.59 (63.96-68.84)	13.84 (12.63-14.91)	75.85 (75.5-76.14)	66.94 (64.31-69.24)	13.6 (12.41-14.68)
Nicaragua	73.33 (72.86-73.78)	64.0 (61.26-66.4)	14.92 (13.66-16.1)	75.97 (75.55-76.38)	66.49 (63.7-68.96)	15.08 (13.78-16.23)	76.56 (76.14-77.01)	67.17 (64.38-69.71)	14.95 (13.66-16.13)	77.21 (76.82-77.6)	67.84 (65.11-70.24)	14.93 (13.7-16.06)	77.95 (77.24-78.67)	68.43 (65.51-70.97)	15.09 (13.76-16.31)
Panama	74.86 (74.26-75.45)	65.94 (63.39-68.43)	13.53 (12.42-14.58)	76.47 (75.97-77.01)	67.11 (64.4-69.67)	14.29 (13.08-15.39)	77.17 (76.62-77.69)	67.62 (64.76-70.11)	14.63 (13.39-15.76)	77.32 (76.81-77.84)	67.75 (64.88-70.25)	14.81 (13.48-15.95)	78.17 (77.11-79.14)	68.36 (65.31-71.13)	15.01 (13.61-16.28)
Venezuela	72.52 (72.44-72.61)	63.73 (61.15-66.02)	12.3 (11.25-13.24)	73.75 (73.68-73.83)	63.95 (61.04-66.47)	12.72 (11.53-13.75)	74.61 (74.53-74.68)	64.59 (61.67-67.18)	13.21 (11.95-14.3)	74.87 (74.8-74.94)	64.84 (61.85-67.42)	13.49 (12.22-14.6)	74.79 (73.65-75.87)	64.77 (61.68-67.57)	13.37 (12.05-14.58)

Appendix Table 2. Life expectancy at birth and HALE at birth and at age 65 by location for GBD years 1990 - 2015, both sexes combined.

Location	1990			2000			2005			2010			2015		
	Life expectancy	HALE		Life expectancy	HALE		Life expectancy	HALE		Life expectancy	HALE		Life expectancy	HALE	
	At birth	At birth	At age 65	At birth	At birth	At age 65	At birth	At birth	At age 65	At birth	At birth	At age 65	At birth	At birth	At age 65
Andean Latin America	67.9 (67.52-68.27)	59.38 (56.81-61.65)	12.32 (11.23-13.28)	72.77 (72.41-73.13)	63.71 (61.11-66.07)	13.14 (12.03-14.15)	74.93 (74.57-75.27)	65.57 (62.88-67.97)	13.79 (12.58-14.84)	76.13 (75.7-76.53)	66.67 (63.89-69.12)	14.05 (12.84-15.16)	77.22 (76.61-77.81)	67.57 (64.7-70.11)	14.21 (12.97-15.36)
Bolivia	61.64 (60.79-62.56)	54.16 (51.81-56.28)	10.7 (9.65-11.65)	67.58 (66.91-68.27)	59.28 (56.74-61.63)	11.66 (10.59-12.64)	70.21 (69.29-71.09)	61.51 (58.92-63.93)	12.13 (11.04-13.22)	71.84 (70.5-73.12)	62.9 (60.1-65.58)	12.38 (11.22-13.58)	73.16 (71.43-74.74)	63.98 (60.94-66.82)	12.61 (11.34-13.87)
Ecuador	71.19 (70.84-71.53)	62.15 (59.47-64.48)	13.01 (11.82-14.05)	73.19 (72.72-73.62)	64.03 (61.32-66.45)	13.23 (12.09-14.27)	74.12 (73.63-74.55)	64.89 (62.1-67.4)	13.37 (12.14-14.45)	75.07 (74.48-75.55)	65.72 (62.91-68.21)	13.48 (12.29-14.61)	75.89 (75.14-76.62)	66.43 (63.49-69.01)	13.54 (12.26-14.7)
Peru	68.72 (68.23-69.25)	60.05 (57.47-62.44)	12.62 (11.53-13.64)	74.56 (74.04-75.09)	65.26 (62.6-67.65)	13.67 (12.53-14.71)	77.21 (76.69-77.74)	67.53 (64.73-70.02)	14.65 (13.4-15.76)	78.38 (77.84-78.91)	68.64 (65.84-71.14)	15.03 (13.8-16.12)	79.51 (78.6-80.32)	69.58 (66.7-72.25)	15.23 (13.9-16.4)
Caribbean	67.82 (67.45-68.18)	59.39 (56.83-61.57)	12.44 (11.39-13.34)	70.13 (69.7-70.56)	61.38 (58.8-63.73)	12.83 (11.73-13.8)	70.89 (70.37-71.39)	62.02 (59.34-64.39)	13.06 (11.92-14.04)	58.71 (49.14-69.49)	51.16 (42.77-60.17)	12.19 (10.99-13.32)	73.05 (72.11-73.91)	63.78 (60.88-66.19)	13.28 (12.1-14.29)
Antigua and Barbuda	73.53 (72.84-74.21)	64.55 (61.86-67.06)	12.51 (11.44-13.46)	73.76 (73.11-74.34)	64.59 (61.95-67.09)	12.58 (11.51-13.58)	74.85 (74.23-75.52)	65.46 (62.65-67.96)	12.83 (11.67-13.89)	75.81 (75.06-76.5)	66.29 (63.42-68.81)	13.13 (11.88-14.24)	76.38 (75.6-77.37)	66.77 (63.77-69.35)	13.18 (11.94-14.47)
The Bahamas	68.52 (67.5-69.43)	60.01 (57.52-62.33)	11.19 (10.23-12.04)	70.17 (69.45-70.86)	61.37 (58.8-63.74)	11.83 (10.78-12.81)	71.72 (70.98-72.43)	62.66 (59.95-65.03)	12.21 (11.12-13.18)	72.72 (71.85-73.58)	63.52 (60.85-66.0)	12.73 (11.59-13.81)	73.68 (72.46-74.83)	64.38 (61.46-67.01)	12.73 (11.6-13.84)
Barbados	73.05 (72.12-73.83)	63.97 (61.26-66.48)	12.48 (11.37-13.45)	74.0 (73.21-74.71)	64.68 (61.83-67.26)	12.68 (11.54-13.77)	75.02 (74.41-75.6)	65.47 (62.66-68.15)	13.08 (11.84-14.19)	75.24 (74.56-75.85)	65.69 (62.82-68.12)	13.05 (11.88-14.17)	75.68 (74.6-76.61)	66.03 (63.11-68.66)	13.13 (11.82-14.33)
Belize	71.37 (70.6-72.05)	62.65 (60.08-65.0)	12.61 (11.57-13.54)	68.99 (68.36-69.62)	60.69 (58.25-62.88)	11.19 (10.29-12.0)	70.03 (69.35-70.68)	61.51 (59.01-63.71)	11.53 (10.55-12.42)	71.2 (70.68-71.73)	62.51 (59.96-64.82)	11.94 (10.93-12.85)	71.73 (70.69-72.67)	62.97 (60.33-65.55)	12.02 (10.99-13.02)
Bermuda	71.06 (70.56-71.51)	62.54 (60.07-64.74)	11.6 (10.66-12.42)	74.13 (73.62-74.6)	65.05 (62.39-67.49)	12.44 (11.39-13.39)	76.37 (75.94-76.86)	66.88 (64.2-69.41)	13.35 (12.25-14.38)	77.65 (77.21-78.15)	67.96 (65.17-70.51)	13.82 (12.66-14.86)	78.46 (77.73-79.15)	68.59 (65.79-71.1)	14.15 (12.98-15.2)
Cuba	74.69 (74.62-74.75)	65.75 (63.1-68.09)	13.23 (12.16-14.16)	76.16 (76.09-76.23)	67.05 (64.32-69.42)	13.47 (12.38-14.43)	77.12 (77.06-77.18)	67.79 (65.01-70.22)	13.69 (12.53-14.69)	77.8 (77.73-77.87)	68.33 (65.56-70.76)	13.89 (12.74-14.89)	78.15 (77.91-78.42)	68.59 (65.79-71.06)	14.08 (12.91-15.1)
Dominica	72.79 (71.95-73.49)	63.58 (60.82-66.09)	12.35 (11.26-13.29)	73.73 (72.91-74.44)	64.28 (61.51-66.75)	12.91 (11.72-13.94)	74.19 (73.35-74.9)	64.59 (61.75-67.08)	13.17 (11.99-14.22)	73.65 (72.66-74.5)	64.13 (61.38-66.66)	12.91 (11.73-14.01)	73.11 (71.72-74.39)	63.66 (60.67-66.34)	12.63 (11.4-13.81)
Dominican Republic	71.8 (71.35-72.29)	62.84 (60.22-65.15)	13.78 (12.65-14.78)	73.34 (72.64-73.92)	64.25 (61.54-66.65)	13.73 (12.54-14.75)	73.33 (72.75-73.84)	64.28 (61.7-66.73)	13.76 (12.63-14.79)	74.45 (73.94-74.88)	65.3 (62.58-67.74)	13.82 (12.65-14.84)	75.31 (74.68-75.79)	66.07 (63.24-68.45)	13.88 (12.73-14.89)
Grenada	69.86 (68.71-70.76)	61.0 (58.31-63.48)	11.17 (10.22-12.05)	70.5 (69.82-71.1)	61.75 (59.18-64.13)	11.49 (10.5-12.39)	70.26 (69.53-70.9)	61.48 (58.91-63.76)	11.37 (10.35-12.27)	70.97 (70.29-71.59)	62.04 (59.3-64.36)	11.51 (10.5-12.42)	71.48 (70.38-72.48)	62.45 (59.69-64.97)	11.65 (10.55-12.57)
Guyana	64.18 (63.55-64.76)	56.1 (53.65-58.3)	9.9 (9.04-10.65)	64.18 (63.52-64.86)	56.13 (53.74-58.27)	10.34 (9.41-11.16)	64.41 (63.67-65.16)	56.42 (53.97-58.55)	10.0 (9.09-10.8)	65.47 (64.56-66.33)	57.38 (54.98-59.59)	10.21 (9.3-11.06)	66.76 (65.48-68.0)	58.48 (55.92-60.91)	10.51 (9.59-11.42)
Haiti	54.28 (53.05-55.49)	47.13 (44.65-49.2)	9.05 (8.18-9.87)	58.69 (57.26-60.27)	51.08 (48.44-53.58)	10.02 (8.97-11.06)	60.02 (58.25-61.87)	52.31 (49.58-54.9)	10.24 (9.13-11.32)	32.16 (21.15-54.51)	28.1 (17.86-44.68)	6.51 (4.7-8.5)	64.29 (61.75-66.85)	55.72 (52.48-58.62)	10.27 (9.18-11.39)
Jamaica	74.77 (74.05-75.39)	65.42 (62.66-67.87)	13.58 (12.44-14.58)	74.35 (73.69-74.94)	64.97 (62.24-67.59)	13.56 (12.42-14.56)	75.35 (74.5-76.04)	65.7 (62.84-68.36)	14.06 (12.85-15.14)	74.84 (74.01-75.49)	65.25 (62.37-67.88)	13.38 (12.21-14.48)	74.97 (73.97-75.97)	65.34 (62.4-68.05)	13.26 (12.04-14.37)
Puerto Rico	73.75 (73.63-73.88)	64.54 (61.81-66.97)	13.27 (12.14-14.27)	75.6 (75.47-75.73)	65.9 (63.04-68.41)	13.67 (12.51-14.72)	76.57 (76.44-76.68)	66.72 (63.8-69.23)	13.93 (12.73-15.01)	77.14 (77.02-77.25)	67.21 (64.31-69.75)	14.0 (12.77-15.08)	78.47 (78.0-78.94)	68.26 (65.29-70.87)	14.56 (13.28-15.74)
Saint Lucia	70.18 (69.02-71.1)	60.97 (58.21-63.53)	11.33 (10.3-12.29)	71.98 (71.31-72.6)	62.94 (60.38-65.34)	11.87 (10.79-12.84)	73.11 (72.3-73.88)	63.89 (61.22-66.28)	12.42 (11.29-13.47)	74.52 (73.61-75.25)	65.06 (62.27-67.51)	13.22 (12.04-14.28)	74.8 (73.52-75.89)	65.34 (62.53-67.88)	13.08 (11.88-14.17)
Saint Vincent and the Grenadines	69.67 (68.53-70.69)	61.01 (58.37-63.56)	11.58 (10.57-12.6)	69.1 (68.37-69.72)	60.47 (57.88-62.79)	11.03 (10.07-11.93)	70.42 (69.71-71.06)	61.63 (59.05-63.93)	11.38 (10.36-12.33)	71.07 (70.3-71.8)	62.17 (59.52-64.49)	11.56 (10.49-12.53)	71.24 (70.15-72.2)	62.34 (59.59-64.65)	11.56 (10.45-12.52)
Suriname	69.71 (69.05-70.33)	61.04 (58.53-63.39)	12.39 (11.35-13.34)	69.7 (68.98-70.35)	61.08 (58.54-63.45)	12.27 (11.19-13.19)	69.85 (69.11-70.62)	61.25 (58.71-63.58)	12.16 (11.08-13.17)	70.71 (69.86-71.6)	62.04 (59.42-64.47)	12.4 (11.32-13.45)	72.01 (70.8-73.16)	63.13 (60.38-65.72)	12.78 (11.59-13.91)
Trinidad and Tobago	69.11 (68.77-69.44)	60.62 (58.07-62.93)	10.84 (9.9-11.66)	69.46 (69.04-69.85)	60.89 (58.34-63.21)	11.21 (10.24-12.07)	71.12 (70.66-71.49)	62.12 (59.45-64.48)	11.92 (10.86-12.84)	71.79 (71.14-72.35)	62.65 (59.9-65.04)	12.05 (10.97-13.03)	72.39 (71.29-73.41)	63.13 (60.29-65.59)	12.17 (11.05-13.21)
Virgin Islands, U.S.	73.13 (72.68-73.57)	64.1 (61.45-66.45)	12.24 (11.21-13.14)	73.97 (73.57-74.36)	64.7 (61.93-67.06)	12.6 (11.53-13.54)	74.43 (73.99-74.87)	65.07 (62.4-67.46)	12.74 (11.66-13.74)	74.34 (73.78-74.87)	65.03 (62.36-67.47)	12.63 (11.52-13.62)	74.43 (73.54-75.22)	65.09 (62.47-67.69)	12.64 (11.56-13.72)
Tropical Latin America	68.03 (67.66-68.39)	59.56 (57.08-61.83)	11.18 (10.22-12.07)	70.77 (70.41-71.12)	61.74 (59.16-64.04)	11.73 (10.71-12.65)	72.43 (72.09-72.76)	63.15 (60.47-65.57)	12.38 (11.27-13.38)	73.64 (73.24-74.0)	64.16 (61.46-66.68)	12.88 (11.75-13.92)	74.4 (73.88-74.87)	64.82 (62.04-67.27)	13.02 (11.82-14.06)
Brazil	67.87 (67.49-68.24)	59.43 (56.94-61.68)	11.13 (10.17-12.02)	70.69 (70.32-71.05)	61.66 (59.09-63.96)	11.7 (10.68-12.62)	72.39 (72.04-72.73)	63.11 (60.43-65.53)	12.37 (11.26-13.36)	73.62 (73.21-74.0)	64.15 (61.45-66.68)	12.88 (11.75-13.92)	74.4 (73.88-74.87)	64.82 (62.05-67.26)	13.03 (11.83-14.07)
Paraguay	74.23 (73.65-74.83)	64.88 (62.11-67.39)	13.29 (12.12-14.37)	74.2 (73.58-74.77)	64.78 (62.01-67.32)	13.19 (12.04-14.26)	74.01 (73.27-74.66)	64.63 (61.79-67.18)	12.97 (11.77-14.1)	73.98 (73.14-74.69)	64.63 (61.76-67.11)	12.75 (11.54-13.84)	74.41 (73.16-75.61)	64.91 (61.87-67.57)	12.73 (11.49-13.89)
Southeast Asia, East Asia, and Oceania	66.36 (65.69-67.02)	59.01 (56.72-61.04)	10.63 (9.81-11.37)	69.17 (68.74-69.59)	61.57 (59.32-63.53)	11.28 (10.43-12.01)	71.1 (70.78-71.42)	63.26 (61.0-65.32)	11.68 (10.78-12.48)	73.2 (72.87-73.54)	65.11 (62.74-67.28)	12.45 (11.48-13.3)	74.93 (74.47-75.41)	66.58 (64.09-68.73)	13.16 (12.18-14.07)
East Asia	66.81 (65.9-67.72)	59.7 (57.45-61.73)	10.55 (9.74-11.27)	69.7 (69.07-70.31)	62.35 (60.16-64.34)	11.32 (10.49-12.03)	71.96 (71.53-72.37)	64.34 (62.08-66.36)	11.74 (10.87-12.52)	74.33 (73.97-74.65)	66.4 (64.07-68.53)	12.67 (11.72-13.51)	76.23 (75.66-76.77)	68.01 (65.59-70.16)	13.5 (12.52-14.42)
China	66.68 (65.73-67.59)	59.6 (57.36-61.62)	10.5 (9.7-11.22)	69.83 (69.25-70.39)	62.48 (60.27-64.47)	11.29 (10.48-12.02)	71.91 (71.46-72.34)	64.31 (62.06-66.33)	11.69 (10.83-12.47)	74.31 (73.93-74.65)	66.4 (64.07-68.53)	12.63 (11.7-13.48)	76.25 (75.66-76.8)	68.04 (65.62-70.21)	13.49 (12.51-14.41)
North Korea	68.88 (64.68-72.42)	61.39 (56.92-65.23)	12.06 (10.74-13.34)	59.05 (42.72-68.68)	52.93 (37.93-61.41)	10.49 (9.19-11.96)	70.24 (67.72-72.49)	62.64 (59.25-65.46)	11.84 (10.71-12.91)	71.23 (69.26-73.06)	63.56 (60.5-66.08)	12.07 (11.05-13.1)	72.06 (69.7-74.25)	64.3 (61.26-67.07)	12.32 (11.16-13.45)
Taiwan	73.75 (73.67-73.84)	65.43 (62.93-67.63)	12.12 (11.16-12.94)	75.99 (75.91-76.07)	67.53 (65.05-69.75)	13.4 (12.37-14.31)	77.37 (77.29-77.45)	68.63 (66.06-70.86)	14.13 (13.03-15.09)	78.98 (78.9-79.05)	69.93 (67.27-72.24)	14.86 (13.68-15.87)	79.47 (78.41-80.59)	70.29 (67.33-72.88)	15.11 (13.86-16.35)
Southeast Asia	65.55 (64.82-66.25)	57.46 (54.95-59.68)	10.95 (9.97-11.86)	68.09 (67.41-68.71)	59.86 (57.39-62.06)	11.17 (10.19-12.08)	69.6 (68.92-70.28)	61.18 (58.7-63.37)	11.51 (10.51-12.44)	70.95 (70.17-71.69)	62.42 (59.95-64.74)	11.8 (10.79-12.74)	72.19 (71.31-73.12)	63.52 (61.05-65.84)	12.1 (11.06-13.08)
Cambodia	57.61 (56.75-58.55)	50.17 (47.85-52.17)	9.05 (8.13-9.84)	60.0 (59.3-60.73)	52.49 (50.23-54.58)	9.39 (8.52-10.18)	63.97 (63.21-64.78)	56.09 (53.8-58.28)	9.9 (9.03-10.74)	67.01 (66.03-67.95)	58.93 (56.37-61.28)	10.51 (9.57-11.4)	69.43 (67.99-70.95)	61.04 (58.37-63.69)	11.03 (10.01-12.09)
Indonesia	64.0 (63.37-64.67)	56.21 (53.88-58.3)	10.46 (9.52-11.31)	67.47 (66.85-68.09)	59.46 (57.16-61.49)	10.73 (9.82-11.56)	68.72 (67.95-69.59)	60.5 (58.1-62.71)	10.89 (9.91-11.77)	69.83 (68.8-71.1)	61.49 (58.99-63.78)	11.09 (10.06-12.06)	71.11 (69.53-72.98)	62.59 (59.86-65.29)	11.45 (10.35-12.64)
Laos	51.5 (49.44-53.68)	45.34 (42.8-47.9)	8.66 (7.63-9.79)	56.07 (54.84-57.35)	49.31 (47.13-51.42)	9.28 (8.38-10.17)	60.07 (58.81-61.18)	52.77 (50.5-54.96)	10.01 (9.07-10.91)	64.14 (62.72-65.51)	56.39 (53.95-58.74)	10.63 (9.66-11.58)	67.1 (64.9-69.14)	58.95 (56.11-61.71)	11.09 (9.97-12.2)
Malaysia	71.82 (71.74-71.9)	63.3 (60.83-65.43)	11.18 (10.26-11.98)	73.31 (73.24-73.37)	64.55 (62.1-66.81)	11.6 (10.66-12.46)	74.25 (74.19-74.31)	65.37 (62.87-67.62)	11.95 (10.96-12.83)	74.45 (74.25-74.63)	65.6 (63.04-67.84)	12.25 (11.23-13.14)	75.32 (74.28-76.35)	66.28 (63.54-68.81)	12.68 (11.56-13.75)
Maldives	65.98 (65.5-66.46)	56.99 (54.45-59.25)	10.3 (9.31-11.18)	73.01 (72.58-73.45)	63.14 (60.27-65.59)	12.27 (11.14-13.26)	76.89 (76.51-77.25)	66.74 (63.78-69.24)	13.78 (12.56-14.84)	79.22 (78.76-79.67)	69.04 (66.15-71.59)	14.76 (13.51-15.88)	79.99 (78.92-81.11)	69.87 (66.85-72.63)	14.93 (13.56-16.2)
Mauritius	69.28 (69.09-69.47)	61.61 (59.43-63.57)	10.25 (9.45-10.98)	71.42 (71.24-71.6)	63.36 (61.02-65.41)	11.27 (10.34-12.07)	72.37 (72.18-72.55)	64.04 (61.66-66.11)	11.63 (10.69-12.47)	73.38 (73.17-73.57)	64.75 (62.23-66.9)	12.35 (11.3-13.25)	74.52 (74.09-74.97)	65.63 (63.07-67.86)	12.64 (11.54-13.58)
Myanmar	57.57 (52.99-62.75)	50.67 (46.42-55.14)	8.87 (7.26-10.79)	60.84 (56.44-65.81)	53.54 (49.27-57.97)	9.19 (7.63-10.9)	63.37 (58.94-68.39)	55.71 (51.57-60.08)	9.66 (8.06-11.53)	66.05 (61.1-70.71)	58.12 (53.6-62.41)	10.26 (8.64-12.02)	68.04 (63.47-72.62)	59.88 (55.58-64.01)	10.64 (9.07-12.37)
Philippines	67.55 (67.36-67.74)	58.54 (55.96-60.76)	11.43 (10.38-12.35)	69.14 (69.02-69.27)	60.23 (57.72-62.53)	11.12 (10.16-12.01)	69.07 (68.94-69.21)	60.26 (57.68-62.49)	10.83 (9.86-11.69)	70.07 (69.89-70.23)	61.22 (58.66-63.44)	11.18 (10.18-12.06)	70.96 (70.16-71.71)	62.05 (59.36-64.31)	11.34 (10.29-12.29)
Sri Lanka	70.46 (70.21-70.71)	61.53 (58.99-63.83)	12.73 (11.59-13.69)	72.41 (72.18-72.65)	63.66 (61.12-65.83)	12.58 (11.49-13.5)	73.86 (73.68-74.04)	64.94 (62.34-67.17)	12.46 (11.4-13.38)	75.04 (74.26-75.79)	65.85 (62.96-68.25)	13.24 (12.1-14.22)	77.65 (75.78-79.5)	68.16 (64.84-71.14)	14.15 (12.66-15.52)
Seychelles	69.06 (68.58-69.57)	61.39 (59.21-63.38)	11.14 (10.24-11.96)	70.88 (70.44-71.31)	62.75 (60.36-64.86)	11.66 (10.67-12.54)	71.95 (71.56-72.4)	63.62 (61.28-65.75)	11.96 (10.95-12.84)	73.12 (72.6-73.61)	64.63 (62.15-66.8)	12.34 (11.31-13.27)	74.08 (72.98-75.2)	65.41 (62.82-67.82)	12.55 (11.45-13.57)
Thailand	71.0 (70.48-71.54)	62.3 (59.77-64.63)	12.38 (11.32-13.32)	70.75 (70.1-71.41)	62.22 (59.71-64.41)	12.3 (11.26-13.24)	73.07 (72.45-73.64)	64.29 (61.73-66.56)	13.16 (12.02-14.16)	74.15 (73.56-74.75)	65.34 (62.76-67.6)	13.05 (11.95-14.05)	74.7 (73.33-76.18)	65.86 (63.17-68.5)	13.04 (11.88-14.21)
Timor-Leste	58.53 (56.53-60.57)	51.43 (48.81-53.78)	10.45 (9.41-11.45)	60.65 (57.92-63.46)	53.09 (49.78-56.21)	11.04 (9.99-12.05)	67.49 (66.19-68.81)	59.22 (56.67-61.72)	11.59 (10.5-12.75)	70.81 (68.98-72.62)	61.99 (59.12-64.78)	12.33 (11.11-13.5)	72.44 (70.04-74.73)	63.52 (60.2-66.64)	12.59 (11.3-13.88)
Vietnam	69.35 (67.72-70.71)	60.88 (58.2-63.42)	11.79 (10.64-12.78)	71.98 (70.52-73.66)	63.36 (60.64-66.01)	12.15 (10.98-13.36)	73.27 (71.23-75.56)	64.58 (61.57-67.57)	12.63 (11.31-13.96)	74.52 (72.02-76.84)	65.95 (62.9-68.88)	13.11 (11.82-14.43)	75.4 (73.25-77.77)	66.8 (63.6-69.84)	13.32 (11.99-14.69)
Oceania	58.62 (55.1-62.08)	51.04 (47.6-54.53)	8.27 (7.19-9.46)	59.67 (55.97-63.63)	51.86 (48.18-55.63)	8.38 (7.29-9.72)	59.95 (56.05-63.97)	52.12 (48.28-55.94)	8.4 (7.26-9.68)	60.97 (56.5-64.99)	53.04 (48.73-56.86)	8.5 (7.3-9.74)	62.53 (57.96-66.45)	54.35 (49.92-58.09)	8.78 (7.56-9.99)
American Samoa	70.23 (69.1-71.38)	61.49 (58.82-64.02)	10.93 (9.94-11.87)	71.67 (70.54-72.66)	62.55 (59.89-65.13)	11.37 (10.35-12.35)	72.0 (70.8-73.12)	62.83 (60.06-65.31)	11.47 (10.39-12.47)	72.76 (71.58-74.03)	63.47 (60.56-66.09)	11.68 (10.49-12.76)	72.87 (71.14-74.58)	63.58 (60.66-66.57)	11.65 (10.47-12.85)

Appendix Table 2. Life expectancy at birth and HALE at birth and at age 65 by location for GBD years 1990 - 2015, both sexes combined.

Location	1990			2000			2005			2010			2015		
	Life expectancy	HALE		Life expectancy	HALE		Life expectancy	HALE		Life expectancy	HALE		Life expectancy	HALE	
	At birth	At birth	At age 65	At birth	At birth	At age 65	At birth	At birth	At age 65	At birth	At birth	At age 65	At birth	At birth	At age 65
Federated States of Micronesia	64.33 (59.38-69.86)	56.87 (52.22-61.36)	9.55 (8.03-11.32)	66.6 (61.82-71.23)	58.75 (53.94-62.84)	9.97 (8.29-11.43)	67.58 (62.04-72.28)	59.61 (54.16-63.8)	10.21 (8.28-11.69)	68.29 (62.33-72.83)	60.28 (54.6-64.42)	10.41 (8.3-12.01)	68.94 (62.76-73.18)	60.78 (55.07-65.01)	10.56 (8.43-12.19)
Fiji	65.11 (63.07-67.0)	56.77 (53.94-59.49)	9.35 (8.34-10.29)	64.22 (62.84-65.66)	55.82 (53.08-58.36)	9.05 (8.07-9.94)	64.8 (63.39-66.03)	56.54 (53.91-59.02)	9.21 (8.29-10.07)	65.18 (63.73-66.65)	56.95 (54.18-59.43)	9.15 (8.2-9.99)	65.82 (63.7-67.83)	57.5 (54.61-60.23)	9.53 (8.51-10.53)
Guam	72.61 (71.66-73.54)	64.08 (61.51-66.38)	11.98 (10.96-12.88)	73.58 (72.58-74.43)	64.73 (62.18-67.13)	12.25 (11.22-13.18)	73.09 (72.13-73.88)	63.97 (61.28-66.54)	12.0 (10.96-13.0)	71.59 (70.56-72.68)	62.56 (59.7-65.17)	11.46 (10.39-12.45)	71.68 (70.09-73.21)	62.72 (60.04-65.37)	11.54 (10.48-12.63)
Kiribati	58.04 (56.3-59.78)	50.74 (48.19-53.14)	8.27 (7.45-9.09)	59.76 (58.37-61.17)	52.09 (49.54-54.48)	8.57 (7.71-9.36)	60.81 (58.89-62.6)	52.97 (50.23-55.59)	8.79 (7.88-9.64)	61.93 (59.73-64.1)	54.03 (51.05-56.86)	9.02 (8.08-9.9)	62.99 (60.6-65.29)	55.06 (51.9-58.05)	9.17 (8.21-10.12)
Marshall Islands	64.63 (63.2-66.05)	56.03 (53.18-58.66)	9.36 (8.41-10.27)	64.69 (63.0-66.24)	55.94 (53.07-58.76)	9.24 (8.23-10.15)	64.71 (63.2-66.38)	55.99 (53.14-58.77)	9.15 (8.16-10.1)	65.76 (63.92-67.67)	56.9 (53.97-59.74)	9.4 (8.37-10.43)	66.94 (65.06-68.92)	57.82 (54.76-60.68)	9.7 (8.57-10.7)
Northern Mariana Islands	72.3 (70.83-73.61)	63.57 (60.83-66.2)	11.66 (10.57-12.65)	75.5 (74.88-76.18)	66.25 (63.57-68.72)	12.63 (11.53-13.65)	75.95 (75.27-76.55)	66.63 (63.86-69.04)	12.88 (11.72-13.9)	76.38 (75.56-77.13)	67.03 (64.24-69.47)	13.18 (12.03-14.2)	76.17 (75.09-77.24)	66.87 (63.9-69.41)	13.12 (11.92-14.22)
Papua New Guinea	56.01 (51.55-60.87)	48.64 (44.52-52.87)	7.63 (6.39-9.22)	57.54 (52.78-62.9)	49.89 (45.45-54.54)	7.78 (6.48-9.42)	57.86 (52.82-63.19)	50.16 (45.53-54.99)	7.77 (6.43-9.44)	59.11 (53.55-64.53)	51.3 (46.17-56.09)	7.92 (6.45-9.5)	60.94 (55.28-66.13)	52.84 (47.62-57.41)	8.22 (6.69-9.76)
Samoa	69.11 (66.68-71.31)	60.79 (57.75-63.64)	10.72 (9.57-11.79)	70.81 (68.8-72.69)	62.11 (59.19-64.96)	11.13 (9.97-12.23)	71.8 (70.26-73.28)	62.91 (60.0-65.64)	11.42 (10.26-12.55)	72.61 (71.22-74.01)	63.55 (60.67-66.33)	11.61 (10.41-12.78)	73.22 (71.39-75.1)	64.01 (61.01-67.0)	11.78 (10.54-13.08)
Solomon Islands	60.07 (55.09-65.26)	53.2 (48.68-57.58)	8.29 (6.98-9.72)	61.74 (56.9-66.7)	54.61 (50.11-58.87)	8.59 (7.17-9.97)	61.79 (56.75-67.1)	54.69 (50.35-59.37)	8.57 (7.15-10.06)	62.72 (57.42-68.16)	55.51 (50.62-60.17)	8.76 (7.11-10.32)	63.96 (57.99-69.4)	56.51 (51.37-61.23)	9.02 (7.3-10.65)
Tonga	69.13 (67.26-70.94)	60.22 (57.08-62.94)	10.48 (9.42-11.48)	69.57 (68.5-70.6)	60.71 (57.88-63.2)	10.67 (9.64-11.62)	69.98 (68.75-71.17)	61.15 (58.29-63.59)	10.85 (9.77-11.85)	70.65 (69.05-72.34)	61.68 (58.75-64.46)	11.07 (9.96-12.16)	71.51 (69.44-73.49)	62.43 (59.39-65.34)	11.39 (10.19-12.54)
Vanuatu	61.92 (57.04-66.56)	55.07 (50.56-59.25)	8.81 (7.43-10.19)	62.52 (57.4-67.56)	55.54 (51.13-60.13)	8.98 (7.51-10.52)	62.73 (57.62-67.73)	55.76 (51.04-60.46)	9.1 (7.6-10.75)	63.91 (58.26-68.46)	56.64 (51.58-61.16)	9.33 (7.7-10.82)	64.88 (59.63-69.05)	57.47 (52.63-61.66)	9.55 (7.89-11.02)
North Africa and Middle East	65.93 (65.44-66.4)	56.99 (54.36-59.33)	11.02 (9.92-11.95)	69.39 (68.93-69.86)	60.05 (57.37-62.51)	11.7 (10.59-12.7)	70.53 (70.01-71.06)	61.12 (58.44-63.61)	11.86 (10.74-12.87)	71.93 (71.36-72.5)	62.35 (59.67-64.88)	12.16 (11.04-13.22)	72.4 (71.69-73.05)	62.66 (59.91-65.31)	12.33 (11.17-13.38)
Afghanistan	49.54 (46.89-52.65)	42.43 (39.65-45.41)	7.1 (6.14-8.18)	50.14 (47.36-52.96)	43.13 (40.27-46.08)	6.84 (5.98-7.82)	52.01 (49.19-54.98)	44.93 (42.03-47.97)	6.93 (6.08-7.92)	53.62 (50.66-56.5)	46.38 (43.44-49.32)	7.08 (6.16-8.09)	53.83 (50.7-56.94)	46.6 (43.6-49.88)	7.23 (6.28-8.31)
Algeria	70.18 (69.3-71.03)	60.99 (58.23-63.46)	12.03 (10.91-13.09)	73.28 (72.36-74.09)	63.65 (60.77-66.31)	12.7 (11.52-13.78)	74.78 (73.9-75.7)	64.96 (62.06-67.69)	13.14 (11.91-14.26)	76.01 (75.28-76.77)	65.96 (63.09-68.65)	13.51 (12.27-14.65)	76.4 (75.44-77.37)	66.28 (63.37-69.0)	13.5 (12.23-14.64)
Bahrain	71.83 (70.75-72.86)	61.99 (59.09-64.79)	11.21 (10.02-12.37)	73.7 (72.85-74.58)	63.38 (60.39-66.23)	11.62 (10.44-12.72)	76.09 (75.47-76.77)	65.47 (62.39-68.16)	12.31 (11.08-13.45)	78.68 (77.91-79.42)	67.6 (64.35-70.48)	13.41 (12.03-14.69)	79.14 (77.81-80.25)	68.0 (64.72-71.09)	13.59 (12.16-14.98)
Egypt	65.2 (64.88-65.53)	56.46 (53.93-58.67)	10.61 (9.6-11.51)	69.72 (69.48-69.96)	60.58 (57.95-62.95)	11.27 (10.18-12.23)	70.12 (69.9-70.36)	60.89 (58.2-63.3)	11.07 (9.99-12.01)	70.07 (69.81-70.32)	60.9 (58.23-63.34)	10.83 (9.78-11.76)	71.48 (70.94-71.96)	61.97 (59.14-64.44)	11.19 (10.11-12.2)
Iran	65.96 (64.22-67.77)	56.97 (53.92-59.74)	11.24 (10.14-12.33)	71.35 (70.15-72.32)	61.76 (58.93-64.35)	11.79 (10.63-12.82)	71.95 (70.29-73.7)	62.51 (59.5-65.44)	11.48 (10.28-12.64)	73.69 (71.65-75.61)	64.04 (61.08-66.93)	11.93 (10.65-13.22)	74.63 (72.93-76.29)	64.85 (61.73-67.93)	12.24 (10.98-13.57)
Iraq	65.74 (63.62-67.7)	56.43 (53.39-59.23)	9.95 (8.84-11.06)	65.79 (63.85-67.64)	56.58 (53.55-59.42)	9.62 (8.54-10.68)	65.68 (63.38-67.84)	56.57 (53.47-59.5)	9.72 (8.57-10.78)	67.35 (64.81-69.77)	58.1 (54.95-61.21)	9.98 (8.82-11.11)	67.39 (64.68-70.06)	57.98 (54.5-61.25)	10.23 (9.0-11.4)
Jordan	71.49 (69.92-72.73)	61.81 (58.9-64.61)	11.5 (10.27-12.67)	73.4 (72.46-74.37)	63.51 (60.64-66.27)	11.85 (10.67-12.96)	74.53 (73.98-75.12)	64.42 (61.49-67.02)	12.04 (10.83-13.09)	77.93 (77.66-78.2)	67.18 (64.09-70.0)	13.66 (12.32-14.85)	78.49 (77.61-79.3)	67.67 (64.61-70.54)	13.89 (12.54-15.14)
Kuwait	77.2 (76.17-78.16)	66.96 (63.98-69.66)	14.02 (12.71-15.15)	77.7 (77.51-77.92)	67.49 (64.61-70.13)	13.58 (12.34-14.7)	77.72 (77.49-77.93)	67.64 (64.78-70.27)	13.47 (12.25-14.6)	78.46 (78.09-78.77)	68.32 (65.33-70.92)	13.5 (12.24-14.62)	80.32 (79.34-81.21)	69.82 (66.8-72.69)	14.4 (13.03-15.68)
Lebanon	69.34 (67.55-71.09)	57.92 (54.56-60.99)	11.03 (9.74-12.16)	75.9 (74.44-77.23)	64.02 (60.43-67.2)	12.81 (11.47-14.13)	77.33 (75.63-78.85)	65.6 (62.15-68.77)	13.18 (11.79-14.52)	78.09 (76.17-79.96)	66.66 (63.18-69.96)	13.35 (11.85-14.81)	78.87 (76.86-80.89)	67.37 (63.74-70.64)	13.58 (12.02-15.04)
Libya	73.39 (72.31-74.39)	63.87 (61.02-66.48)	12.88 (11.69-13.97)	74.8 (74.0-75.55)	65.07 (62.24-67.63)	13.04 (11.84-14.12)	75.36 (74.71-75.98)	65.49 (62.62-68.07)	13.08 (11.87-14.14)	75.94 (75.15-76.7)	65.95 (63.03-68.65)	13.13 (11.9-14.27)	74.08 (72.49-75.63)	64.23 (61.16-67.05)	12.69 (11.41-13.93)
Morocco	67.72 (66.4-68.93)	58.3 (55.33-60.91)	11.25 (10.02-12.31)	71.48 (69.87-72.95)	61.49 (58.43-64.44)	11.86 (10.59-13.08)	72.73 (70.49-74.43)	62.55 (59.43-65.76)	12.08 (10.8-13.42)	73.72 (71.4-75.76)	63.42 (60.14-66.67)	12.26 (10.82-13.69)	74.58 (71.97-76.83)	64.12 (60.67-67.5)	12.41 (10.84-13.93)
Palestine	71.77 (69.38-73.72)	61.89 (58.79-64.8)	12.2 (10.92-13.44)	74.41 (73.44-75.37)	64.17 (61.26-66.98)	13.03 (11.87-14.09)	74.63 (73.67-75.32)	64.61 (61.73-67.29)	12.99 (11.84-14.09)	74.25 (72.73-75.71)	64.53 (61.55-67.37)	12.63 (11.39-13.83)	74.61 (72.76-76.64)	64.84 (61.62-67.65)	12.57 (11.27-13.85)
Oman	72.09 (69.9-74.66)	62.05 (58.63-65.5)	12.15 (10.75-13.61)	75.24 (73.77-76.79)	64.81 (61.66-67.93)	12.39 (11.08-13.69)	75.99 (75.16-76.76)	65.39 (62.28-68.17)	12.43 (11.17-13.59)	76.05 (75.33-76.77)	65.45 (62.41-68.24)	12.31 (11.08-13.47)	77.19 (75.94-78.74)	66.36 (63.15-69.49)	12.77 (11.44-14.17)
Qatar	74.52 (73.44-75.58)	64.42 (61.53-67.23)	12.31 (11.09-13.47)	74.74 (73.68-75.72)	64.63 (61.78-67.36)	12.14 (10.9-13.34)	76.27 (75.07-77.37)	66.01 (62.99-68.93)	12.68 (11.32-14.0)	79.22 (78.17-80.21)	68.4 (65.19-71.29)	13.97 (12.52-15.32)	79.74 (77.95-81.31)	68.83 (65.39-72.0)	14.06 (12.42-15.67)
Saudi Arabia	73.86 (72.19-75.25)	64.39 (61.45-67.23)	13.56 (12.25-14.83)	76.81 (76.13-77.47)	66.89 (63.97-69.64)	13.88 (12.64-15.02)	77.77 (77.21-78.28)	67.8 (64.95-70.46)	14.07 (12.83-15.21)	78.7 (78.12-79.26)	68.68 (65.77-71.41)	14.39 (13.11-15.59)	79.52 (78.7-80.29)	69.36 (66.27-72.09)	14.68 (13.32-15.98)
Sudan	58.85 (56.65-61.14)	50.75 (47.83-53.5)	10.07 (8.85-11.19)	62.37 (59.72-65.06)	53.61 (50.28-56.88)	10.24 (8.93-11.57)	64.56 (61.78-67.43)	55.54 (52.13-59.02)	10.47 (9.14-11.88)	66.09 (63.45-69.09)	56.86 (53.45-60.42)	10.66 (9.37-12.13)	67.52 (64.39-70.53)	58.21 (54.68-61.95)	10.87 (9.5-12.26)
Syria	70.49 (68.98-71.74)	61.72 (59.03-64.23)	11.8 (10.64-12.83)	74.39 (73.12-75.46)	65.18 (62.48-67.78)	12.7 (11.56-13.8)	75.5 (75.01-76.04)	66.19 (63.44-68.62)	12.93 (11.83-13.93)	76.51 (76.07-76.97)	66.99 (64.21-69.42)	13.26 (12.09-14.29)	68.17 (63.53-73.6)	58.57 (53.87-63.89)	12.61 (11.25-13.76)
Tunisia	71.75 (71.12-72.4)	62.32 (59.64-64.89)	12.08 (10.98-13.11)	74.98 (74.11-75.9)	65.12 (62.17-67.7)	12.93 (11.7-14.03)	76.22 (75.06-77.44)	66.2 (63.19-68.91)	13.34 (12.06-14.52)	77.11 (75.63-78.57)	66.98 (63.92-69.81)	13.61 (12.25-14.92)	77.61 (76.01-79.31)	67.37 (64.16-70.32)	13.6 (12.19-15.02)
Turkey	68.09 (67.35-68.85)	58.7 (55.88-61.21)	11.37 (10.22-12.44)	73.76 (73.14-74.36)	63.72 (60.83-66.37)	12.81 (11.6-13.93)	76.23 (75.71-76.74)	66.03 (63.11-68.61)	13.59 (12.31-14.74)	78.77 (78.31-79.17)	68.2 (65.16-70.9)	14.59 (13.22-15.81)	79.24 (78.68-79.77)	68.62 (65.5-71.36)	14.59 (13.15-15.82)
United Arab Emirates	70.55 (68.39-72.61)	60.99 (57.73-64.05)	10.93 (9.72-12.15)	73.32 (72.39-74.3)	63.45 (60.56-66.11)	11.67 (10.55-12.76)	75.18 (74.48-75.84)	65.09 (62.12-67.85)	12.4 (11.18-13.48)	75.56 (74.12-76.78)	65.61 (62.52-68.46)	12.55 (11.23-13.72)	75.37 (73.09-77.41)	65.52 (62.09-68.63)	12.4 (11.01-13.69)
Yemen	58.48 (53.85-63.76)	51.21 (46.9-55.54)	9.75 (7.95-11.55)	62.42 (57.4-67.61)	54.65 (49.99-59.04)	9.94 (8.06-11.81)	64.55 (59.39-70.15)	56.5 (51.88-61.33)	10.16 (8.26-12.28)	66.56 (60.72-72.03)	58.24 (53.05-63.09)	10.46 (8.39-12.5)	65.95 (60.2-70.93)	57.53 (52.54-61.81)	10.48 (8.53-12.31)
South Asia	58.44 (58.04-58.87)	50.3 (47.93-52.42)	8.97 (8.08-9.76)	61.94 (61.57-62.39)	53.31 (50.81-55.58)	9.47 (8.53-10.31)	63.75 (63.35-64.18)	54.89 (52.36-57.19)	9.86 (8.91-10.72)	65.59 (65.13-66.07)	56.68 (54.09-59.05)	10.21 (9.27-11.09)	67.36 (66.75-67.91)	58.26 (55.61-60.64)	10.61 (9.64-11.55)
Bangladesh	57.91 (56.98-58.95)	49.8 (47.36-52.12)	9.57 (8.61-10.48)	64.95 (64.0-65.94)	56.02 (53.28-58.47)	10.73 (9.68-11.73)	67.09 (66.32-67.91)	57.93 (55.22-60.44)	11.0 (9.91-12.0)	68.83 (67.85-69.74)	59.63 (56.95-62.09)	11.28 (10.18-12.28)	70.41 (69.02-71.73)	61.04 (58.16-63.73)	11.59 (10.43-12.68)
Bhutan	61.38 (59.41-63.46)	52.96 (50.15-55.56)	10.46 (9.34-11.55)	65.91 (64.29-67.45)	56.6 (53.65-59.24)	11.35 (10.12-12.5)	69.33 (67.76-70.82)	59.67 (56.66-62.51)	12.13 (10.85-13.38)	71.39 (69.72-73.02)	61.41 (58.34-64.4)	12.59 (11.26-13.91)	72.68 (70.88-74.56)	62.41 (59.19-65.39)	12.58 (11.23-13.87)
India	58.16 (57.67-58.66)	50.0 (47.65-52.18)	8.72 (7.85-9.53)	61.55 (61.11-62.05)	52.89 (50.39-55.18)	9.27 (8.35-10.12)	63.55 (63.08-64.05)	54.66 (52.14-56.98)	9.72 (8.77-10.57)	65.37 (64.82-65.9)	56.44 (53.86-58.81)	10.07 (9.15-10.96)	67.25 (66.57-67.93)	58.12 (55.48-60.54)	10.51 (9.54-11.45)
Nepal	58.45 (56.89-60.19)	50.89 (48.29-53.32)	9.89 (8.81-10.93)	65.21 (64.35-66.09)	56.69 (54.16-58.92)	10.92 (9.92-11.87)	67.39 (66.27-68.51)	58.58 (55.83-60.98)	11.29 (10.15-12.31)	69.04 (67.41-70.69)	60.19 (57.27-62.82)	11.46 (10.27-12.55)	69.71 (67.8-71.54)	60.57 (57.63-63.45)	11.53 (10.3-12.75)
Pakistan	62.11 (60.85-63.32)	53.83 (51.27-56.27)	10.49 (9.49-11.52)	62.32 (61.08-63.59)	54.17 (51.61-56.69)	9.89 (8.9-10.88)	62.22 (60.91-63.54)	53.88 (51.35-56.32)	9.9 (8.92-10.88)	64.53 (63.09-66.1)	55.96 (53.17-58.64)	10.25 (9.22-11.34)	65.87 (64.27-67.41)	57.11 (54.27-59.87)	10.45 (9.35-11.59)
Sub-Saharan Africa	53.37 (52.41-54.17)	46.03 (43.67-48.13)	9.07 (8.1-9.95)	53.16 (52.53-53.74)	45.86 (43.5-47.92)	9.07 (8.11-9.95)	55.09 (54.38-55.77)	47.53 (45.11-49.57)	9.45 (8.45-10.31)	58.76 (57.86-59.65)	50.72 (48.07-53.05)	10.01 (8.89-10.98)	61.8 (60.41-63.08)	53.48 (50.73-56.02)	10.28 (9.12-11.26)
Southern Sub-Saharan Africa	62.23 (61.38-62.98)	54.16 (51.84-56.39)	9.52 (8.62-10.38)	54.28 (53.06-55.56)	47.18 (44.94-49.21)	8.55 (7.64-9.42)	50.84 (49.69-52.05)	44.17 (42.06-46.08)	8.85 (7.86-9.82)	53.64 (52.41-54.79)	46.42 (44.18-48.44)	9.55 (8.45-10.54)	60.36 (59.07-61.48)	52.09 (49.6-54.47)	10.03 (8.97-11.07)
Botswana	62.32 (50.5-72.52)	54.99 (44.44-63.06)	9.78 (6.17-13.23)	47.65 (37.86-61.55)	42.71 (33.38-52.39)	8.91 (4.98-12.65)	49.29 (39.05-62.2)	43.83 (34.4-52.96)	8.9 (5.01-12.79)	55.61 (43.33-67.55)	49.06 (37.85-57.53)	9.62 (5.33-13.11)	58.7 (45.57-69.23)	51.51 (40.19-59.38)	9.88 (5.53-13.12)
Lesotho	60.47 (57.74-63.18)	52.93 (49.82-56.05)	9.55 (8.25-10.91)	50.65 (47.52-53.77)	44.17 (40.76-47.4)	8.74 (7.34-10.12)	43.07 (40.27-46.08)	37.72 (35.12-40.5)	7.27 (6.21-8.26)	46.42 (42.94-50.28)	40.44 (37.25-43.85)	7.68 (6.23-9.19)	47.11 (42.33-52.87)	41.19 (36.95-45.79)	7.75 (5.94-9.76)
Namibia	61.66 (60.12-63.34)	53.77 (51.07-56.25)	9.21 (8.24-10.21)	54.04 (51.4-56.69)	47.15 (44.29-49.88)	8.0 (7.0-9.1)	53.07 (50.18-56.01)	46.26 (43.26-49.23)	8.66 (7.37-9.9)	61.43 (57.88-65.01)	53.19 (49.7-56.87)	10.52 (8.93-12.29)	64.18 (59.3-68.78)	55.49 (51.0-59.71)	11.1 (9.04-13.08)
South Africa	62.03 (61.05-62.89)	53.95 (51.58-56.23)	9.41 (8.54-10.28)	56.43 (55.3-57.64)	49.02 (46.78-51.13)	8.55 (7.59-9.42)	52.65 (51.3-54.17)	45.68 (43.52-47.74)	9.12 (8.01-10.14)	54.86 (53.66-56.31)	47.41 (45.06-49.63)	9.84 (8.64-10.93)	61.34 (60.17-62.29)	52.86 (50.35-55.13)	10.13 (9.03-11.19)
Swaziland	60.67 (57.3-64.45)	52.88 (49.41-56.45)	8.98 (7.63-10.43)	48.8 (45.34-52.6)	42.27 (38.96-45.63)	8.27 (6.82-9.77)	42.45 (39.22-46.05)	36.85 (33.95-39.67)	7.15 (5.86-8.47)	46.42 (42.48-51.21)	40.08 (36.52-43.87)	7.73 (6.07-9.61)	51.81 (46.28-58.61)	44.72 (39.85-50.08)	8.31 (6.25-10.67)
Zimbabwe	63.04 (60.76-65.44)	55.0 (52.09-57.75)	10.4 (9.2-11.72)	47.88 (44.19-52.15)	41.82 (38.62-45.1)	8.84 (7.16-10.33)	45.07 (42.43-48.28)	39.46 (37.15-42.11)	8.17 (6.81-9.63)	49.11 (46.92-51.78)	42.94 (40.51-45.39)	8.65 (7.33-10.08)	59.25 (54.9-64.08)	51.68 (47.76-55.92)	10.19 (8.3-12.3)

Appendix Table 2. Life expectancy at birth and HALE at birth and at age 65 by location for GBD years 1990 - 2015, both sexes combined.

	1990			2000			2005			2010			2015		
Location	Life expectancy	HALE		Life expectancy	HALE		Life expectancy	HALE		Life expectancy	HALE		Life expectancy	HALE	
	At birth	At birth	At age 65	At birth	At birth	At age 65	At birth	At birth	At age 65	At birth	At birth	At age 65	At birth	At birth	At age 65
Western Sub-Saharan Africa	54.19 (52.3-55.85)	46.58 (43.9-49.04)	9.8 (8.66-10.93)	54.96 (54.01-55.94)	47.39 (44.95-49.57)	9.89 (8.87-10.85)	56.65 (55.6-57.65)	48.89 (46.43-51.12)	10.13 (9.06-11.1)	60.03 (58.8-61.31)	51.87 (49.26-54.36)	10.66 (9.52-11.74)	62.53 (60.39-64.22)	54.18 (51.26-56.94)	10.92 (9.69-12.01)
Benin	56.09 (54.4-57.85)	48.25 (45.49-50.89)	10.15 (9.04-11.22)	57.2 (54.93-59.43)	49.57 (46.71-52.43)	9.92 (8.69-11.09)	58.59 (55.4-62.04)	51.05 (47.46-54.37)	9.97 (8.43-11.42)	60.77 (55.42-65.78)	53.15 (48.26-57.77)	10.0 (8.07-11.99)	62.32 (55.9-68.92)	54.77 (49.23-60.26)	10.14 (8.01-12.45)
Burkina Faso	51.42 (49.62-53.22)	44.29 (41.75-46.81)	9.3 (8.15-10.46)	52.56 (50.61-54.42)	45.53 (42.96-47.95)	9.56 (8.41-10.65)	55.87 (53.6-58.01)	48.55 (45.6-51.21)	10.06 (8.77-11.24)	59.0 (55.57-62.74)	51.71 (48.18-55.29)	10.36 (8.71-11.97)	60.94 (55.6-66.19)	53.63 (48.76-58.46)	10.43 (8.28-12.59)
Cameroon	58.35 (56.72-60.06)	50.2 (47.33-52.76)	9.9 (8.74-10.97)	54.25 (52.12-56.64)	46.95 (44.28-49.59)	9.25 (8.03-10.59)	54.87 (52.45-57.48)	47.51 (44.6-50.24)	9.37 (8.03-10.72)	57.81 (54.47-61.72)	50.2 (46.84-53.9)	9.93 (8.36-11.67)	59.35 (53.95-64.53)	51.91 (47.03-56.51)	10.21 (8.1-12.38)
Cape Verde	68.66 (68.16-69.14)	59.73 (57.11-62.1)	11.59 (10.55-12.55)	68.44 (65.18-71.68)	59.73 (56.11-63.33)	11.37 (9.88-12.89)	69.3 (66.5-72.21)	60.55 (57.29-63.76)	11.57 (10.25-12.92)	71.06 (70.33-71.8)	62.1 (59.38-64.54)	11.96 (10.82-12.94)	72.9 (70.88-74.51)	63.64 (60.62-66.5)	12.59 (11.2-13.9)
Chad	53.67 (51.86-55.46)	46.13 (43.4-48.61)	10.0 (8.9-11.11)	51.5 (49.23-54.05)	44.54 (41.81-47.38)	9.17 (7.95-10.46)	52.99 (49.04-56.79)	45.83 (41.94-49.49)	9.43 (7.61-11.04)	55.75 (50.46-61.4)	48.33 (43.39-53.57)	9.73 (7.6-11.96)	57.39 (51.25-63.84)	49.88 (44.28-55.68)	9.93 (7.58-12.36)
Cote d'Ivoire	55.28 (53.31-57.19)	47.38 (44.64-50.12)	9.02 (7.87-10.25)	52.05 (50.0-54.22)	45.15 (42.53-47.74)	8.64 (7.35-9.91)	53.37 (51.13-55.7)	46.43 (43.8-48.88)	9.06 (7.83-10.25)	56.64 (53.26-60.02)	49.29 (45.77-52.68)	9.56 (7.97-11.16)	59.14 (54.11-64.28)	51.67 (47.0-56.23)	9.77 (7.82-11.77)
The Gambia	61.26 (55.7-66.43)	53.73 (48.9-58.4)	10.51 (8.39-12.6)	63.65 (61.31-66.14)	55.68 (52.77-58.61)	10.58 (9.34-11.89)	64.93 (62.92-67.13)	56.8 (53.99-59.72)	10.81 (9.62-12.03)	66.53 (64.18-68.98)	58.21 (55.1-61.28)	11.1 (9.81-12.48)	68.13 (64.24-71.66)	59.63 (55.76-63.3)	11.34 (9.68-12.96)
Ghana	58.04 (54.14-61.87)	50.59 (46.87-54.15)	9.44 (7.94-11.0)	60.04 (57.57-62.56)	52.5 (49.44-55.65)	10.04 (8.68-11.47)	60.82 (57.63-63.99)	53.32 (49.94-56.96)	10.22 (8.76-11.82)	62.92 (57.87-67.86)	55.3 (50.96-60.0)	10.54 (8.66-12.66)	65.3 (59.47-70.99)	57.64 (52.25-62.62)	10.85 (8.71-13.04)
Guinea	51.97 (50.43-53.63)	44.87 (42.42-47.36)	9.93 (8.82-11.03)	54.22 (52.67-56.0)	47.1 (44.38-49.48)	9.65 (8.52-10.69)	56.01 (54.04-58.12)	48.73 (45.98-51.28)	9.81 (8.65-10.95)	57.91 (55.35-60.71)	50.46 (47.45-53.55)	9.86 (8.56-11.17)	59.13 (54.56-63.71)	51.73 (47.38-56.07)	9.85 (8.05-11.72)
Guinea-Bissau	51.47 (41.4-60.34)	45.66 (36.52-52.6)	9.62 (6.14-12.9)	52.75 (41.9-62.66)	46.98 (37.0-54.69)	9.63 (6.02-13.2)	52.88 (41.72-63.29)	47.28 (36.94-55.21)	9.64 (5.85-13.25)	54.27 (42.6-64.65)	48.58 (37.57-56.56)	9.76 (5.82-13.29)	55.65 (42.14-65.78)	49.78 (37.62-57.62)	9.83 (5.76-13.3)
Liberia	48.55 (45.25-52.12)	40.96 (37.56-44.35)	9.1 (7.67-10.52)	55.83 (53.85-57.81)	47.16 (44.27-49.97)	9.71 (8.45-11.0)	58.38 (56.5-60.61)	49.42 (46.39-52.26)	9.51 (8.15-10.81)	60.82 (58.33-63.55)	51.83 (48.47-55.16)	9.6 (8.27-11.07)	63.21 (58.92-67.44)	54.2 (49.94-58.13)	10.05 (8.32-11.9)
Mali	50.01 (48.67-51.43)	43.06 (40.68-45.34)	9.02 (7.97-10.07)	53.48 (51.93-55.04)	46.07 (43.52-48.39)	9.79 (8.63-10.93)	56.87 (54.85-58.75)	48.96 (46.02-51.65)	10.43 (9.09-11.64)	59.21 (56.67-61.87)	51.28 (48.19-54.08)	10.76 (9.4-12.03)	60.19 (55.95-64.37)	52.43 (48.27-56.38)	10.7 (8.86-12.5)
Mauritania	59.47 (57.8-61.29)	51.11 (48.27-53.76)	9.29 (8.18-10.38)	63.78 (62.03-65.67)	54.91 (51.88-57.63)	10.41 (9.17-11.62)	66.32 (64.18-68.43)	57.06 (53.84-60.06)	11.25 (9.87-12.58)	68.6 (65.51-71.51)	59.15 (55.52-62.45)	11.69 (10.08-13.17)	69.75 (65.43-74.08)	60.26 (55.92-64.36)	11.76 (9.88-13.68)
Niger	47.15 (45.56-48.8)	41.07 (38.8-43.14)	9.87 (8.8-10.85)	52.41 (50.76-54.01)	45.66 (43.28-47.86)	10.19 (9.12-11.16)	55.94 (54.16-57.85)	48.8 (46.2-51.16)	10.41 (9.27-11.41)	59.18 (56.48-61.84)	51.78 (48.8-54.73)	10.55 (9.25-11.85)	60.97 (56.43-65.4)	53.53 (49.41-57.87)	10.48 (8.76-12.28)
Nigeria	54.14 (50.37-57.7)	46.35 (42.61-49.99)	10.06 (8.28-11.65)	55.2 (53.32-57.12)	47.29 (44.49-49.84)	10.3 (9.06-11.51)	56.78 (54.61-58.86)	48.64 (45.95-51.32)	10.52 (9.31-11.78)	61.19 (58.65-63.59)	52.39 (49.21-55.54)	11.46 (9.97-12.97)	64.59 (60.04-67.61)	55.45 (51.32-59.05)	11.96 (10.05-13.57)
Sao Tome and Principe	63.44 (61.83-64.94)	55.26 (52.53-57.8)	11.02 (9.88-12.05)	64.63 (62.75-66.64)	56.38 (53.5-59.1)	10.41 (9.29-11.55)	65.47 (63.03-67.85)	57.14 (53.94-60.19)	10.28 (9.12-11.52)	66.93 (62.92-70.66)	58.41 (54.34-62.39)	10.46 (8.85-12.13)	68.0 (62.7-73.51)	59.38 (54.25-64.31)	10.67 (8.69-12.76)
Senegal	57.76 (55.98-59.67)	50.46 (47.87-53.03)	10.08 (8.99-11.15)	59.72 (57.52-61.74)	52.14 (49.33-54.79)	10.08 (8.92-11.24)	62.18 (58.84-65.55)	54.33 (50.78-57.6)	10.32 (8.85-11.83)	64.16 (58.87-69.38)	56.25 (51.43-60.99)	10.36 (8.46-12.36)	65.32 (58.95-71.47)	57.49 (51.62-62.71)	10.42 (8.25-12.69)
Sierra Leone	51.62 (47.97-55.18)	44.37 (40.74-47.82)	10.22 (8.67-11.78)	51.48 (49.6-53.3)	44.22 (41.5-46.67)	9.33 (8.2-10.41)	52.28 (50.74-53.92)	45.12 (42.69-47.5)	8.85 (7.82-9.88)	54.96 (52.76-57.38)	47.9 (44.98-50.59)	9.14 (7.89-10.38)	57.13 (52.84-61.34)	50.11 (46.06-53.79)	9.56 (7.85-11.28)
Togo	57.76 (55.89-59.97)	50.38 (47.74-52.85)	10.02 (8.93-11.1)	56.71 (54.4-59.3)	49.48 (46.58-52.38)	9.71 (8.43-10.98)	56.8 (54.34-59.48)	49.62 (46.66-52.46)	9.82 (8.46-11.1)	58.66 (55.63-61.83)	51.41 (48.33-54.63)	9.99 (8.64-11.4)	61.48 (57.09-66.17)	54.09 (49.88-58.28)	10.34 (8.47-12.18)
Eastern Sub-Saharan Africa	50.86 (49.8-51.82)	44.21 (41.9-46.24)	8.44 (7.55-9.29)	51.71 (50.76-52.57)	44.9 (42.84-46.93)	8.74 (7.87-9.62)	55.31 (54.23-56.29)	48.03 (45.67-50.13)	9.3 (8.35-10.18)	59.77 (58.23-61.23)	51.96 (49.19-54.43)	9.93 (8.8-10.98)	62.4 (60.34-64.41)	54.45 (51.51-57.26)	10.22 (9.02-11.36)
Burundi	45.59 (41.47-51.0)	40.43 (36.61-44.9)	6.82 (5.79-8.45)	46.74 (44.64-49.01)	41.24 (38.82-43.69)	7.25 (6.33-8.26)	54.68 (52.42-57.04)	48.08 (45.19-50.88)	8.75 (7.66-9.88)	60.44 (56.83-64.19)	53.41 (49.93-56.78)	9.83 (8.43-11.3)	61.39 (55.91-67.15)	54.65 (49.67-59.42)	9.92 (7.96-11.94)
Comoros	57.1 (51.92-63.09)	49.94 (45.27-54.7)	9.0 (7.22-10.93)	62.14 (59.7-64.46)	54.16 (51.22-56.98)	9.7 (8.54-10.83)	65.99 (63.61-68.39)	57.51 (54.53-60.43)	10.7 (9.52-11.93)	67.71 (65.04-70.35)	59.19 (55.97-62.31)	11.01 (9.69-12.26)	68.07 (63.05-72.43)	59.65 (55.3-63.72)	10.92 (9.26-12.68)
Djibouti	60.99 (56.24-65.79)	53.22 (48.66-57.4)	10.38 (8.49-12.1)	59.97 (52.75-67.2)	52.43 (45.89-58.14)	10.12 (7.79-12.48)	60.72 (53.75-67.54)	53.03 (46.54-58.76)	10.15 (7.64-12.58)	62.35 (54.8-69.29)	54.41 (48.08-60.35)	10.26 (7.76-12.73)	63.77 (56.51-70.51)	55.61 (49.15-61.09)	10.33 (7.94-12.62)
Eritrea	52.43 (50.36-54.64)	45.69 (42.81-48.33)	7.85 (6.82-8.87)	37.61 (29.05-50.45)	32.26 (24.63-42.39)	8.06 (6.31-10.19)	59.94 (53.43-66.97)	52.19 (46.63-57.77)	9.2 (7.11-11.57)	60.13 (52.69-67.59)	52.55 (46.23-58.73)	9.07 (6.84-11.52)	60.74 (52.84-68.72)	53.28 (46.14-59.27)	9.11 (6.73-11.52)
Ethiopia	45.23 (43.27-47.14)	39.76 (37.47-41.96)	7.24 (6.42-8.11)	52.05 (50.28-53.86)	45.7 (43.49-47.97)	8.46 (7.54-9.43)	56.05 (54.36-57.93)	49.26 (46.85-51.61)	9.18 (8.18-10.22)	62.12 (58.83-65.78)	54.59 (51.12-57.95)	10.27 (8.78-11.83)	65.18 (60.04-70.12)	57.37 (52.59-61.81)	10.66 (8.74-12.56)
Kenya	61.84 (60.44-63.08)	53.45 (50.51-55.89)	10.22 (9.09-11.15)	55.6 (54.36-56.81)	47.83 (45.14-50.2)	10.01 (8.86-11.13)	57.25 (56.05-58.38)	49.22 (46.34-51.6)	10.36 (9.15-11.46)	62.92 (61.58-64.14)	54.0 (50.71-56.7)	10.83 (9.54-11.94)	65.14 (63.55-66.53)	56.25 (53.1-59.08)	11.03 (9.75-12.24)
Madagascar	55.95 (54.64-57.29)	48.1 (45.52-50.44)	8.93 (7.93-9.91)	59.15 (57.47-60.67)	50.9 (48.16-53.35)	9.08 (8.07-10.0)	61.22 (58.88-63.48)	52.8 (49.63-55.55)	9.2 (8.05-10.24)	62.44 (58.47-66.51)	54.14 (50.37-57.96)	9.51 (8.06-10.98)	63.46 (57.95-69.31)	55.41 (50.71-60.58)	9.72 (7.91-11.66)
Malawi	48.83 (46.69-50.94)	42.25 (39.6-44.56)	9.24 (8.0-10.49)	45.41 (42.74-48.27)	39.38 (36.78-42.0)	8.56 (7.08-10.25)	48.51 (45.99-51.24)	42.09 (39.44-44.79)	9.15 (7.61-10.71)	55.37 (52.72-58.16)	48.12 (45.27-51.06)	10.16 (8.64-11.7)	60.51 (55.64-64.88)	53.0 (48.86-57.02)	10.83 (8.84-12.84)
Mozambique	51.46 (49.75-53.02)	44.11 (41.47-46.52)	9.29 (8.1-10.31)	52.33 (50.23-54.47)	44.92 (42.17-47.57)	9.01 (7.71-10.33)	52.89 (50.05-56.1)	45.31 (42.21-48.44)	9.23 (7.64-10.76)	54.38 (51.05-58.46)	46.68 (43.26-50.18)	9.12 (7.52-10.84)	57.11 (51.85-62.72)	49.56 (44.86-54.18)	9.54 (7.54-11.71)
Rwanda	50.55 (48.49-52.73)	44.51 (42.07-46.84)	8.16 (7.16-9.12)	49.23 (47.52-51.0)	42.72 (40.36-45.06)	8.17 (7.2-9.2)	59.63 (57.38-61.81)	51.53 (48.51-54.45)	10.61 (9.26-11.96)	65.09 (61.82-68.41)	56.69 (53.06-60.41)	11.24 (9.66-12.84)	65.85 (60.27-70.55)	57.61 (52.77-62.15)	11.16 (9.17-13.15)
Somalia	48.83 (38.93-60.25)	43.17 (34.3-51.25)	8.28 (5.45-11.86)	50.71 (40.75-63.25)	44.91 (35.58-53.85)	8.19 (5.47-11.83)	52.06 (41.06-64.81)	46.07 (35.97-55.25)	8.24 (5.37-11.97)	51.61 (40.45-63.5)	45.62 (35.38-54.28)	8.16 (5.29-11.49)	54.17 (42.06-65.97)	47.84 (37.2-56.43)	8.37 (5.43-11.76)
South Sudan	50.88 (40.95-60.7)	44.01 (35.67-51.36)	8.56 (5.47-11.79)	54.4 (43.24-64.81)	47.15 (36.92-55.3)	8.68 (5.46-12.01)	55.29 (43.09-65.96)	48.15 (37.86-56.3)	8.85 (5.59-12.26)	55.99 (42.86-66.45)	49.01 (37.78-56.75)	9.04 (5.49-12.45)	56.14 (43.56-66.74)	49.19 (38.22-57.16)	9.04 (5.55-12.57)
Tanzania	55.23 (53.5-57.1)	47.8 (45.22-50.33)	9.6 (8.46-10.72)	54.29 (52.0-56.71)	47.07 (44.5-49.84)	9.68 (8.4-11.11)	57.13 (54.27-60.06)	49.53 (46.53-52.42)	10.34 (8.91-11.86)	61.39 (56.78-65.59)	53.37 (49.06-57.44)	10.74 (8.83-12.55)	64.06 (57.88-69.41)	56.17 (50.72-60.76)	10.87 (8.66-13.02)
Uganda	50.69 (48.52-53.34)	43.82 (41.15-46.44)	8.69 (7.52-10.15)	49.14 (47.33-51.25)	42.74 (40.46-45.21)	8.38 (7.29-9.77)	53.51 (51.47-55.62)	46.53 (43.74-49.17)	8.82 (7.59-10.09)	57.77 (54.52-61.59)	50.45 (47.2-54.07)	9.69 (8.17-11.41)	61.25 (55.98-67.23)	53.75 (48.88-58.62)	10.18 (8.21-12.4)
Zambia	51.95 (49.85-54.22)	45.42 (42.97-47.98)	9.48 (8.37-10.72)	44.84 (42.76-47.11)	39.34 (37.11-41.74)	7.79 (6.82-8.79)	47.11 (45.14-49.11)	41.36 (39.09-43.68)	7.72 (6.84-8.62)	53.08 (50.31-55.89)	46.55 (43.82-49.36)	8.38 (7.27-9.56)	56.75 (52.31-61.71)	49.81 (45.68-54.0)	8.92 (7.3-10.61)
Central Sub-Saharan Africa	52.68 (49.12-55.99)	44.42 (40.59-47.98)	8.33 (6.83-9.78)	53.2 (49.89-55.77)	44.66 (40.63-48.05)	8.42 (7.0-9.71)	55.5 (51.97-58.2)	46.58 (42.5-49.91)	8.62 (7.1-9.93)	57.8 (53.96-60.64)	48.63 (43.9-52.21)	8.81 (7.18-10.21)	59.98 (55.65-63.84)	50.58 (45.97-54.7)	8.96 (7.38-10.53)
Angola	48.63 (39.43-60.27)	42.45 (34.04-50.35)	7.97 (5.41-11.33)	52.26 (41.87-63.48)	45.5 (36.83-53.46)	8.46 (5.58-11.88)	55.15 (43.74-66.15)	48.04 (38.34-56.25)	8.74 (5.62-12.16)	57.63 (44.93-68.29)	50.1 (39.46-57.84)	9.03 (5.65-12.44)	59.62 (45.88-70.01)	52.04 (41.09-59.92)	9.22 (5.75-12.67)
Central African Republic	48.36 (46.24-50.5)	41.42 (38.74-44.05)	7.34 (6.39-8.4)	45.34 (40.83-51.65)	39.14 (34.84-44.48)	7.27 (5.67-9.5)	45.55 (40.5-52.69)	39.38 (34.58-44.81)	7.32 (5.59-9.6)	47.86 (41.85-55.26)	41.49 (36.15-47.36)	7.57 (5.79-9.9)	49.57 (43.29-57.56)	43.19 (37.37-49.36)	7.68 (5.84-10.09)
Congo	54.26 (51.45-57.47)	46.88 (43.58-50.03)	7.52 (6.45-8.68)	52.46 (50.38-54.76)	45.23 (42.4-47.94)	7.88 (6.78-9.03)	56.66 (54.23-59.09)	48.71 (45.39-51.56)	8.74 (7.51-9.95)	60.43 (56.97-63.6)	51.97 (48.05-55.49)	9.12 (7.66-10.56)	61.48 (56.01-66.68)	52.94 (48.08-57.78)	9.22 (7.36-11.21)
Democratic Republic of the Congo	54.5 (50.79-58.62)	45.47 (41.09-49.27)	8.76 (6.97-10.49)	54.34 (52.33-56.34)	45.08 (40.78-48.33)	8.66 (7.07-9.97)	56.44 (54.55-58.43)	46.82 (42.15-50.02)	8.78 (7.14-10.13)	58.45 (55.84-61.03)	48.71 (43.95-52.42)	8.89 (7.12-10.34)	60.75 (56.63-65.28)	50.75 (45.44-55.45)	9.04 (7.21-10.93)
Equatorial Guinea	48.87 (39.39-61.78)	43.31 (34.75-52.9)	7.76 (5.3-11.55)	52.87 (42.4-63.67)	46.69 (36.97-54.79)	9.17 (5.65-12.9)	56.01 (44.8-65.25)	49.16 (38.97-56.28)	9.96 (5.98-13.14)	58.55 (46.82-67.52)	51.3 (41.13-58.06)	10.31 (6.32-13.24)	61.05 (48.58-69.33)	53.48 (42.71-60.0)	10.46 (6.41-13.22)
Gabon	59.46 (57.52-61.62)	50.97 (47.81-53.78)	8.59 (7.5-9.71)	59.35 (57.13-61.5)	50.73 (47.4-53.62)	8.79 (7.57-9.92)	60.15 (57.73-62.65)	51.3 (47.94-54.25)	9.09 (7.77-10.28)	62.99 (59.72-66.08)	53.43 (49.41-57.0)	9.91 (8.35-11.41)	65.87 (60.38-70.27)	55.84 (50.63-60.39)	10.19 (8.16-12.05)

Appendix Table 3. DALYs for all causes and level 1 cause groups by location for 1990 - 2015, both sexes combined.

	Cause	1990	1995	2000	2005	2010	2015
Global	All causes	256,127,151,822,720<br>(241,872,302,073,120–272,432,975,821,480)	257,386,382,514,610<br>(241,650,359,790,680–275,147,037,613,690)	257,393,914,383,320<br>(240,870,372,829,980–276,372,895,979,900)	255,330,675,738,610<br>(237,313,761,874,420–275,632,803,804,820)	249,271,795,250,780<br>(229,837,871,214,860–270,300,259,830,960)	246,489,538,665,230<br>(225,988,897,725,520–269,651,075,114,390)
Global	Group I	119,624,949,926,150<br>(115,220,287,238,450–124,616,933,253,040)	111,663,597,764,660<br>(107,663,753,381,660–116,310,546,741,090)	104,912,535,812,850<br>(100,987,383,793,800–109,419,264,303,590)	96,801,454,581,035<br>(93,180,944,921,269–101,232,791,236,720)	85,198,698,182,969<br>(81,504,146,843,898–89,897,425,338,177)	74,159,590,972,266<br>(70,392,871,460,406–78,765,986,756,643)
Global	NCD	109,276,406,573,910<br>(98,157,176,438,389–120,816,603,781,800)	118,106,901,699,470<br>(105,935,576,435,710–130,634,381,991,590)	125,456,737,768,840<br>(112,576,448,278,950–139,290,464,926,530)	132,220,794,473,300<br>(118,117,974,914,640–147,144,882,737,280)	138,048,582,817,790<br>(122,660,899,067,380–154,398,072,326,310)	147,350,822,496,590<br>(130,980,319,206,280–165,014,744,539,370)
Global	Injuries	27,225,795,322,663<br>(25,215,306,105,564–28,926,645,196,850)	27,615,883,050,491<br>(25,481,054,819,787–29,418,740,525,310)	27,024,640,801,629<br>(25,196,696,388,347–28,617,461,123,587)	26,308,426,684,278<br>(24,556,647,135,395–27,862,150,863,350)	26,024,514,250,028<br>(24,007,986,666,328–27,888,894,961,309)	24,979,125,196,369<br>(23,140,916,281,771–26,641,923,381,308)
High SDI	All causes	32,641,825,275,553<br>(29,439,729,905,067–36,248,797,927,826)	34,895,622,852,159<br>(31,552,738,317,182–38,704,474,550,201)	34,701,943,510,957<br>(31,245,045,344,067–38,698,237,688,073)	35,108,603,205,066<br>(31,517,372,372,129–39,263,470,681,246)	34,257,000,500,169<br>(30,455,146,820,798–38,596,638,389,028)	35,513,523,727,965<br>(31,403,226,026,818–40,079,398,227,469)
High SDI	Group I	2,602,918,922,224<br>(2,346,492,832,489–2,922,046,247,530)	2,583,240,394,325<br>(2,324,841,040,452–2,894,685,531,155)	2,335,426,982,451<br>(2,081,200,120,976–2,649,686,195,716)	2,258,068,618,493<br>(2,001,318,304,761–2,584,059,253,016)	2,130,192,652,676<br>(1,873,668,457,923–2,474,420,057,421)	2,094,423,918,516<br>(1,836,538,406,130–2,449,158,297,822)
High SDI	NCD	25,890,766,287,370<br>(23,272,011,816,430–28,855,396,116,943)	27,697,053,034,198<br>(24,933,896,246,849–30,804,922,011,277)	28,044,093,492,676<br>(25,139,737,553,345–31,298,235,772,880)	28,632,470,468,454<br>(25,591,132,716,026–32,076,703,022,151)	28,515,999,953,420<br>(25,268,393,682,814–32,169,520,255,373)	29,959,560,422,880<br>(26,419,826,319,340–33,873,544,806,569)
High SDI	Injuries	4,148,140,065,958<br>(3,863,452,243,889–4,487,143,685,281)	4,615,329,423,636<br>(4,308,843,750,097–4,977,324,786,136)	4,322,423,035,830<br>(4,023,462,365,180–4,672,677,741,904)	4,218,064,118,120<br>(3,908,185,485,904–4,582,947,986,579)	3,610,807,894,072<br>(3,320,313,146,995–3,975,001,709,178)	3,459,539,386,569<br>(3,149,182,378,651–3,848,902,158,952)
High-middle SDI	All causes	44,917,765,278,805<br>(41,604,978,407,209–48,693,476,356,465)	45,363,658,108,448<br>(41,651,617,488,581–49,343,329,739,288)	45,867,599,072,311<br>(41,857,247,155,141–50,300,676,974,200)	46,861,958,536,259<br>(42,584,630,912,951–51,695,950,115,236)	46,645,697,850,046<br>(42,135,299,087,210–51,725,663,812,886)	46,535,130,813,187<br>(41,693,470,986,956–52,105,242,568,572)
High-middle SDI	Group I	12,109,314,020,702<br>(11,460,459,073,423–12,897,880,887,267)	10,142,949,567,372<br>(9,511,548,914,636–10,859,281,758,323)	9,178,721,453,048<br>(8,584,143,394,838–9,880,273,978,528)	8,800,104,740,792<br>(8,238,779,326,452–9,511,395,176,876)	7,962,396,450,335<br>(7,349,588,685,642–8,705,672,390,354)	6,472,512,464,338<br>(5,875,802,354,487–7,190,160,750,445)
High-middle SDI	NCD	26,294,345,095,185<br>(23,610,105,623,552–29,248,035,601,882)	28,682,479,345,016<br>(25,761,140,683,419–31,879,720,068,015)	30,516,212,951,644<br>(27,325,419,537,530–34,116,928,302,391)	32,179,236,676,018<br>(28,706,837,319,488–36,016,461,670,980)	33,209,221,558,776<br>(29,327,976,145,935–37,401,781,907,184)	34,939,363,130,139<br>(30,844,678,632,283–39,623,272,814,132)
High-middle SDI	Injuries	6,514,106,162,917<br>(6,116,408,224,163–6,896,568,529,304)	6,538,229,196,060<br>(6,199,015,658,742–6,859,510,568,492)	6,172,664,667,620<br>(5,882,455,944,428–6,474,553,313,820)	5,882,617,119,449<br>(5,587,455,983,200–6,191,785,867,145)	5,474,079,840,935<br>(5,171,834,580,672–5,797,257,042,650)	5,123,255,218,711<br>(4,774,247,456,764–5,493,945,731,455)
Middle SDI	All causes	67,829,537,842,725<br>(63,906,906,673,071–72,409,512,240,138)	64,086,431,114,473<br>(59,786,978,707,053–68,903,407,239,563)	61,892,934,426,038<br>(57,505,417,988,045–66,926,188,690,438)	61,001,177,882,041<br>(56,118,756,738,577–66,431,114,455,880)	60,061,406,731,658<br>(54,897,343,240,695–65,837,585,626,479)	60,100,383,907,796<br>(54,670,295,817,247–66,139,683,356,005)
Middle SDI	Group I	28,997,640,839,514<br>(27,670,348,858,525–30,482,111,045,828)	23,769,243,575,201<br>(22,594,500,956,146–25,106,003,848,122)	19,849,743,960,141<br>(18,776,990,716,982–21,099,805,756,101)	17,031,524,809,399<br>(16,067,304,234,133–18,169,878,459,512)	14,690,221,501,690<br>(13,796,484,709,677–15,755,024,607,818)	12,524,489,071,064<br>(11,631,475,757,101–13,570,869,105,847)
Middle SDI	NCD	30,642,740,070,397<br>(27,623,895,037,625–33,800,431,709,796)	32,380,869,692,444<br>(29,130,260,402,832–35,847,706,675,211)	34,548,061,339,674<br>(31,171,330,209,526–38,277,347,576,363)	36,770,140,079,937<br>(32,948,204,751,363–40,818,286,070,378)	38,547,399,669,696<br>(34,363,343,102,457–43,049,858,045,560)	40,655,109,105,275<br>(36,223,480,356,454–45,425,567,671,610)
Middle SDI	Injuries	8,189,156,932,813<br>(7,418,786,963,743–8,717,769,749,730)	7,936,317,846,829<br>(7,176,843,267,088–8,403,436,662,052)	7,495,129,126,223<br>(6,947,543,723,099–7,866,332,589,084)	7,199,512,992,704<br>(6,653,159,572,800–7,565,399,814,907)	6,823,785,560,272<br>(6,250,626,903,331–7,188,271,332,137)	6,920,785,731,458<br>(6,290,523,019,148–7,449,172,452,867)
Low-middle SDI	All causes	81,719,949,690,686<br>(78,368,325,668,930–85,520,164,946,857)	81,539,095,087,761<br>(77,878,644,293,995–85,684,797,855,658)	81,674,247,576,411<br>(77,606,658,645,507–86,132,137,015,765)	80,087,363,253,606<br>(75,715,277,404,968–84,865,434,296,359)	77,140,818,602,803<br>(72,306,758,823,041–82,290,159,501,082)	73,528,319,823,518<br>(68,309,372,456,251–79,399,036,887,869)
Low-middle SDI	Group I	54,245,653,852,188<br>(52,477,034,113,486–56,086,224,718,477)	51,485,024,594,253<br>(49,775,304,204,155–53,322,435,718,708)	49,131,056,244,963<br>(47,419,808,535,707–50,946,021,248,476)	45,565,753,251,129<br>(43,973,652,815,036–47,417,300,911,968)	39,410,274,133,815<br>(37,819,406,881,088–41,328,995,297,309)	34,065,669,730,238<br>(32,374,363,103,001–35,943,979,415,562)
Low-middle SDI	NCD	21,261,930,545,820<br>(19,018,552,671,901–23,668,631,152,654)	23,567,757,558,689<br>(21,088,884,528,457–26,047,828,059,898)	25,967,897,069,494<br>(23,152,783,003,038–28,800,177,562,313)	27,650,091,973,208<br>(24,594,856,213,046–30,633,005,639,378)	29,941,966,111,635<br>(26,643,910,321,851–33,408,557,878,404)	32,712,926,177,107<br>(29,110,564,824,757–36,436,680,933,612)
Low-middle SDI	Injuries	6,212,365,292,678<br>(5,494,785,613,482–6,857,586,470,606)	6,486,312,934,819<br>(5,669,945,107,493–7,111,847,531,341)	6,575,294,261,953<br>(5,715,991,733,939–7,184,241,290,172)	6,871,518,029,269<br>(6,091,993,870,591–7,435,195,826,506)	7,788,578,357,353<br>(6,533,296,208,683–9,038,713,115,304)	6,749,723,916,172<br>(5,996,114,864,366–7,254,478,723,598)
Low SDI	All causes	28,878,328,680,145<br>(27,876,976,173,366–29,916,679,188,427)	31,358,134,967,395<br>(30,212,017,462,326–32,556,288,686,377)	33,108,352,593,534<br>(31,819,279,712,204–34,407,346,088,827)	32,115,333,165,513<br>(30,809,496,154,392–33,675,824,345,590)	30,962,479,434,928<br>(29,390,266,491,315–32,872,657,763,151)	30,647,543,896,158<br>(28,654,131,447,024–32,912,226,394,709)
Low SDI	Group I	21,615,177,503,949<br>(20,743,097,257,167–22,505,083,149,872)	23,631,523,941,136<br>(22,721,323,252,778–24,571,991,708,700)	24,367,906,078,105<br>(23,459,986,429,801–25,428,406,561,277)	23,098,330,644,664<br>(22,246,641,697,242–24,137,394,091,030)	20,965,046,368,825<br>(20,107,129,352,418–22,007,045,264,596)	18,967,285,555,827<br>(17,961,420,518,983–20,218,754,879,541)
Low SDI	NCD	5,116,290,221,675<br>(4,486,560,905,551–5,856,820,870,511)	5,701,869,149,524<br>(5,011,247,125,828–6,474,163,091,437)	6,296,380,318,916<br>(5,532,696,813,166–7,078,961,634,971)	6,895,586,959,472<br>(6,073,708,822,957–7,738,170,021,466)	7,730,649,247,234<br>(6,829,901,566,665–8,709,851,964,565)	8,970,331,114,315<br>(7,898,657,094,444–10,197,579,719,468)
Low SDI	Injuries	2,146,860,954,520<br>(1,728,347,333,070–2,483,299,300,845)	2,024,741,876,735<br>(1,654,641,393,477–2,317,992,018,241)	2,444,066,196,513<br>(2,059,137,725,800–2,791,840,862,984)	2,121,415,561,378<br>(1,876,953,128,607–2,358,359,877,921)	2,266,783,818,869<br>(2,027,353,814,771–2,480,543,285,378)	2,709,927,226,016<br>(2,410,513,141,875–3,007,233,326,621)
High-income	All causes	25,034,546,235,796<br>(22,453,705,484,402–27,974,955,263,988)	25,481,439,129,768<br>(22,769,535,843,695–28,600,411,955,403)	25,460,343,421,426<br>(22,645,749,750,406–28,736,742,173,698)	25,680,230,571,545<br>(22,712,913,553,169–29,115,807,892,234)	26,043,356,282,486<br>(22,934,722,202,933–29,640,047,027,825)	27,367,973,807,885<br>(24,021,193,308,112–31,189,298,035,388)
High-income	Group I	1,905,462,829,328<br>(1,724,843,797,195–2,135,811,357,815)	1,951,598,364,377<br>(1,770,190,946,589–2,184,076,993,118)	1,697,055,615,564<br>(1,510,195,689,202–1,936,654,676,513)	1,636,355,729,137<br>(1,446,275,602,688–1,887,484,330,156)	1,577,828,918,416<br>(1,384,466,023,274–1,836,021,018,605)	1,554,917,138,867<br>(1,360,202,620,757–1,818,315,627,747)
High-income	NCD	20,225,535,950,923<br>(18,070,482,622,348–22,666,742,498,940)	20,689,569,769,437<br>(18,410,348,835,737–23,270,533,988,057)	21,077,603,774,472<br>(18,680,415,411,644–23,799,884,342,633)	21,443,623,019,660<br>(18,897,575,705,089–24,319,193,605,781)	22,039,080,488,838<br>(19,338,324,190,458–25,081,918,874,355)	23,410,644,687,232<br>(20,534,020,542,272–26,647,531,291,820)
High-income	Injuries	2,903,547,455,546<br>(2,665,439,351,122–3,189,593,191,692)	2,840,270,995,953<br>(2,596,600,159,858–3,127,159,779,623)	2,685,684,031,390<br>(2,442,587,343,926–2,985,093,805,395)	2,600,251,822,748<br>(2,348,520,056,720–2,912,518,978,945)	2,426,446,875,232<br>(2,181,225,057,891–2,747,464,269,128)	2,402,411,981,786<br>(2,153,053,320,633–2,732,832,584,548)
High-income North America	All causes	8,040,614,886,649<br>(7,234,739,700,869–8,963,977,735,718)	8,429,848,852,425<br>(7,566,809,996,418–9,413,173,603,687)	8,555,432,147,694<br>(7,624,323,410,768–9,611,865,859,805)	8,924,156,136,424<br>(7,936,514,717,100–10,050,988,945,150)	9,141,393,395,061<br>(8,061,593,102,395–10,344,607,747,374)	9,723,670,287,216<br>(8,552,380,925,540–11,015,470,721,686)
High-income North America	Group I	667,640,596,072<br>(613,898,237,225–732,038,194,047)	736,835,810,295<br>(680,264,939,533–809,234,330,763)	598,299,589,759<br>(535,731,807,947–680,005,983,256)	588,889,645,245<br>(522,740,462,390–674,252,992,250)	549,354,451,841<br>(481,595,386,097–639,534,778,102)	537,219,008,502<br>(467,961,903,614–629,117,256,399)
High-income North America	NCD	6,423,921,000,472<br>(5,721,937,458,980–7,223,150,068,500)	6,765,085,183,347<br>(6,012,750,498,440–7,613,634,389,636)	7,057,510,254,978<br>(6,248,441,908,697–7,976,554,427,987)	7,375,034,693,104<br>(6,507,770,890,264–8,358,709,348,240)	7,698,774,382,129<br>(6,760,360,914,468–8,756,929,855,802)	8,285,454,834,580<br>(7,268,167,319,091–9,409,204,375,308)
High-income North America	Injuries	949,053,290,105<br>(889,803,578,864–1,021,072,202,384)	927,927,858,784<br>(864,313,067,330–1,004,468,347,046)	899,622,302,957<br>(833,197,061,173–980,043,151,436)	960,231,798,074<br>(884,415,168,415–1,052,024,522,508)	893,264,561,092<br>(819,111,712,343–987,616,396,603)	900,996,444,135<br>(821,448,594,469–1,002,700,608,673)
Canada	All causes	685,058,751,283<br>(605,075,834,245–774,879,369,037)	721,478,387,268<br>(636,842,807,786–818,449,960,122)	736,707,939,685<br>(646,115,654,938–840,417,791,278)	766,207,819,261<br>(668,471,167,811–878,157,172,052)	798,054,970,630<br>(692,864,631,135–917,076,702,470)	859,596,482,232<br>(744,379,302,508–984,880,824,569)
Canada	Group I	47,666,958,249<br>(41,235,799,495–56,108,098,763)	51,726,685,343<br>(45,048,394,535–60,711,211,501)	44,514,611,402<br>(37,540,234,548–53,808,905,314)	44,059,734,156<br>(37,090,270,250–53,517,618,823)	46,340,037,932<br>(38,854,616,239–56,625,446,724)	46,912,864,653<br>(38,811,301,360–57,280,125,763)
Canada	NCD	559,240,186,101<br>(491,611,288,202–635,173,195,100)	595,106,629,484<br>(522,367,496,250–676,591,611,653)	621,451,389,560<br>(544,026,172,360–710,294,432,958)	651,463,682,134<br>(566,787,140,561–747,191,738,459)	682,930,845,593<br>(591,357,650,434–783,655,818,408)	742,614,678,968<br>(641,414,265,154–851,433,540,123)
Canada	Injuries	78,151,606,933<br>(72,429,427,538–85,345,608,965)	74,645,072,441<br>(68,630,454,531–82,116,806,253)	70,741,938,723<br>(64,516,454,839–78,493,526,966)	70,684,402,971<br>(63,958,783,485–78,879,322,799)	68,784,087,105<br>(61,749,082,642–77,642,138,509)	70,068,938,611<br>(62,361,617,324–79,751,730,784)
Greenland	All causes	2,437,382,127<br>(2,282,755,647–2,602,723,130)	2,125,039,106<br>(1,971,766,184–2,282,952,286)	1,929,802,609<br>(1,770,572,246–2,097,016,193)	1,870,952,237<br>(1,708,705,204–2,036,521,463)	1,770,048,811<br>(1,618,606,754–1,935,173,671)	1,703,304,835<br>(1,541,509,562–1,881,018,232)
Greenland	Group I	596,215,764<br>(542,694,056–658,366,251)	446,831,220<br>(409,546,847–493,131,456)	306,234,368<br>(277,562,550–339,652,021)	276,353,118<br>(247,445,096–305,344,848)	245,579,490<br>(220,462,533–274,723,406)	208,557,190<br>(184,542,197–235,439,718)
Greenland	NCD	1,259,227,786<br>(1,125,493,563–1,400,032,938)	1,192,991,215<br>(1,070,149,830–1,321,695,755)	1,191,496,692<br>(1,068,019,832–1,325,789,224)	1,203,308,837<br>(1,075,633,930–1,338,618,407)	1,181,213,725<br>(1,054,132,563–1,316,581,631)	1,187,842,950<br>(1,051,374,190–1,326,763,337)
Greenland	Injuries	581,938,577<br>(539,620,406–618,034,275)	485,216,671<br>(456,554,860–515,254,192)	432,071,550<br>(405,342,632–460,105,021)	391,290,281<br>(367,536,773–415,210,434)	343,255,596<br>(320,476,429–366,617,704)	306,904,696<br>(278,754,737–336,592,507)
United States	All causes	7,350,015,442,292<br>(6,623,427,479,283–8,180,643,254,780)	7,703,091,525,010<br>(6,924,557,338,705–8,588,552,779,191)	7,813,678,295,122<br>(6,970,240,724,600–8,765,479,789,182)	8,152,838,215,596<br>(7,262,002,123,290–9,166,927,792,132)	8,338,359,682,223<br>(7,362,599,703,462–9,419,923,201,225)	8,859,067,173,611<br>(7,804,222,509,554–10,015,378,193,748)
United States	Group I	619,079,783,004<br>(571,155,905,787–674,909,140,283)	684,344,148,497<br>(634,509,254,860–747,185,573,388)	553,244,236,257<br>(497,377,665,026–624,837,203,465)	544,329,432,018<br>(485,043,832,608–619,728,328,022)	502,572,526,499<br>(441,989,888,133–582,448,400,823)	489,918,189,070<br>(428,182,122,776–572,004,694,357)
United States	NCD	5,861,047,534,485<br>(5,225,136,802,264–6,583,280,902,040)	6,166,339,656,604<br>(5,487,992,465,165–6,933,721,454,528)	6,432,339,156,586<br>(5,700,591,423,376–7,262,488,784,090)	6,719,719,960,145<br>(5,935,192,952,596–7,607,533,689,491)	7,011,975,690,312<br>(6,161,761,617,064–7,966,465,530,515)	7,538,838,435,303<br>(6,620,394,449,634–8,552,021,276,342)

Appendix Table 3. DALYs for all causes and level 1 cause groups by location for 1990 - 2015, both sexes combined.							
	Cause	1990	1995	2000	2005	2010	2015
United States	Injuries	869,888,124,803 (816,800,369,033–934,117,950,146)	852,407,719,909 (794,771,054,723–921,205,473,226)	828,094,902,278 (767,867,450,366–901,243,374,399)	888,788,823,432 (819,658,002,971–972,326,634,033)	823,811,465,412 (756,828,401,826–909,178,004,576)	830,310,549,238 (758,332,652,018–921,941,214,011)
Australasia	All causes	525,345,180,716 (466,249,036,013–591,583,197,812)	534,560,992,564 (471,543,452,157–605,910,246,842)	546,152,951,931 (477,582,085,102–623,923,008,109)	557,753,181,922 (484,235,857,918–640,693,111,938)	591,642,207,213 (510,900,810,518–682,145,617,742)	638,421,243,989 (548,984,915,169–736,087,165,935)
Australasia	Group I	31,265,812,036 (27,317,806,339–36,744,824,909)	30,313,136,463 (26,055,129,783–35,852,093,206)	29,345,322,932 (24,768,328,123–35,070,231,043)	30,152,604,075 (25,390,923,211–36,485,292,883)	30,920,866,765 (25,834,199,432–37,531,414,533)	30,690,295,564 (25,210,243,148–38,032,206,476)
Australasia	NCD	436,632,581,949 (385,281,528,994–495,357,787,579)	451,107,103,103 (395,485,424,542–514,426,086,638)	465,139,343,109 (405,392,090,202–534,185,026,372)	479,256,441,314 (414,728,178,611–552,773,314,601)	512,987,041,772 (441,495,373,932–592,687,467,610)	558,558,750,828 (480,075,678,643–646,653,894,669)
Australasia	Injuries	57,446,786,731 (53,386,749,794–62,037,795,990)	53,140,752,997 (49,017,080,595–58,037,591,676)	51,668,285,890 (47,430,184,512–56,750,222,038)	48,344,136,533 (43,939,045,625–53,775,948,332)	47,734,298,676 (43,265,560,803–53,569,253,139)	49,172,197,597 (44,328,922,910–55,440,551,174)
Australia	All causes	433,712,465,285 (384,197,443,547–489,329,268,010)	441,076,457,972 (388,567,156,682–500,800,133,693)	452,283,599,038 (394,899,085,961–517,700,428,849)	461,337,417,300 (400,076,480,492–530,810,484,428)	491,957,899,504 (424,185,790,845–567,750,286,502)	534,770,489,754 (460,191,959,430–617,021,582,031)
Australia	Group I	25,731,039,467 (22,245,866,585–30,343,901,496)	25,136,834,897 (21,484,736,644–29,874,188,481)	24,652,437,880 (20,742,126,082–29,548,897,596)	25,437,157,702 (21,402,115,359–30,776,875,285)	26,113,755,479 (21,780,354,136–31,801,528,444)	26,116,707,685 (21,346,575,161–32,501,864,955)
Australia	NCD	361,666,065,553 (318,475,372,148–410,909,485,731)	373,108,645,653 (326,705,027,316–426,048,975,116)	385,358,558,135 (335,665,009,521–443,023,765,607)	396,695,217,070 (342,964,355,608–458,156,278,407)	426,950,223,281 (366,835,309,353–494,265,158,007)	467,974,732,360 (402,167,635,702–542,035,896,946)
Australia	Injuries	46,315,360,265 (42,964,198,877–50,159,687,896)	42,830,977,422 (39,434,994,699–46,900,764,969)	42,272,603,024 (38,703,910,757–46,495,507,019)	39,205,042,527 (35,482,858,399–43,707,083,774)	38,893,920,744 (35,170,346,369–43,793,989,356)	40,679,049,708 (36,600,670,877–46,007,150,393)
New Zealand	All causes	91,632,715,431 (82,297,533,372–102,303,724,806)	93,484,534,592 (83,131,703,725–105,304,241,237)	93,869,352,893 (82,792,001,892–106,343,059,105)	96,415,764,622 (84,159,377,426–109,990,818,227)	99,684,307,709 (86,730,917,061–114,110,363,560)	103,650,754,235 (89,381,637,013–119,040,529,792)
New Zealand	Group I	5,534,772,568 (4,908,716,698–6,323,231,455)	5,176,301,567 (4,495,403,437–6,037,147,839)	4,692,885,052 (3,974,277,526–5,642,347,881)	4,715,446,373 (3,949,004,269–5,728,504,385)	4,807,111,286 (4,009,788,079–5,834,732,743)	4,573,587,879 (3,769,769,182–5,618,181,275)
New Zealand	NCD	74,966,516,396 (66,655,730,028–84,312,909,818)	77,998,457,450 (68,774,999,347–88,457,851,644)	79,780,784,974 (69,998,998,847–91,067,851,142)	82,561,224,244 (71,763,918,057–94,578,955,982)	86,036,818,491 (74,610,752,558–98,899,487,034)	90,584,018,468 (77,761,161,824–104,241,378,593)
New Zealand	Injuries	11,131,426,466 (10,435,074,839–11,930,768,130)	10,309,775,575 (9,602,828,136–11,159,045,056)	9,395,682,867 (8,694,053,262–10,248,705,390)	9,139,094,006 (8,405,242,363–10,028,933,691)	8,840,377,932 (8,086,312,298–9,781,771,424)	8,493,147,888 (7,659,766,844–9,502,243,584)
High-income Asia Pacific	All causes	3,865,778,662,597 (3,447,599,542,922–4,341,480,979,944)	4,009,718,802,198 (3,564,753,555,938–4,523,720,450,665)	4,016,309,332,361 (3,552,586,242,475–4,552,211,686,732)	4,066,295,806,736 (3,581,179,906,900–4,623,833,003,552)	4,147,685,191,929 (3,645,596,536,560–4,726,466,391,711)	4,385,578,104,279 (3,853,249,571,799–4,992,015,108,816)
High-income Asia Pacific	Group I	317,168,832,386 (277,629,441,029–366,437,431,820)	306,174,925,942 (268,781,237,931–354,175,449,841)	295,956,139,812 (259,097,595,229–341,089,154,190)	284,336,847,212 (248,333,705,798–331,089,107,945)	288,088,587,575 (253,628,819,049–336,462,487,406)	300,188,240,078 (265,132,746,600–348,617,618,778)
High-income Asia Pacific	NCD	3,055,850,466,276 (2,707,425,176,533–3,449,618,945,047)	3,160,626,129,595 (2,791,288,427,402–3,578,856,011,874)	3,229,120,446,120 (2,838,612,839,371–3,671,437,871,999)	3,319,101,107,107 (2,906,120,883,976–3,785,434,800,154)	3,421,582,183,319 (2,987,421,014,897–3,916,558,251,247)	3,659,289,621,360 (3,189,912,788,263–4,181,701,328,586)
High-income Asia Pacific	Injuries	492,759,363,936 (455,649,801,672–535,564,982,391)	542,917,746,660 (482,810,679,489–613,095,645,244)	491,292,746,430 (452,823,743,534–538,252,023,640)	462,857,852,418 (424,741,120,213–512,345,966,688)	438,014,421,034 (400,455,965,727–486,622,540,958)	426,100,242,841 (386,399,904,734–477,919,559,494)
Brunei	All causes	5,072,307,381 (4,530,985,866–5,686,806,198)	5,584,690,144 (4,961,946,242–6,265,753,339)	5,823,318,558 (5,115,975,295–6,634,993,843)	6,343,619,084 (5,541,789,253–7,249,315,474)	7,063,747,916 (6,187,513,583–8,060,581,733)	8,059,529,876 (7,045,738,161–9,209,834,717)
Brunei	Group I	668,457,860 (590,362,355–767,232,152)	674,265,170 (595,065,517–777,610,598)	663,649,778 (576,418,088–777,159,257)	676,962,554 (583,601,383–806,012,211)	703,174,788 (601,105,095–837,469,355)	738,314,641 (631,182,551–874,741,477)
Brunei	NCD	3,540,891,626 (3,115,135,676–4,021,757,118)	4,012,070,577 (3,512,406,958–4,567,467,037)	4,298,412,531 (3,725,700,329–4,945,162,587)	4,835,395,397 (4,185,548,669–5,553,573,511)	5,496,944,172 (4,764,897,600–6,324,113,966)	6,421,323,270 (5,566,882,272–7,354,108,414)
Brunei	Injuries	862,957,895 (774,707,597–950,051,698)	898,354,397 (820,834,209–987,346,229)	861,256,249 (786,313,984–940,814,029)	831,261,133 (750,213,868–923,451,654)	863,628,956 (778,649,137–961,623,018)	899,891,965 (793,734,809–1,023,934,435)
Japan	All causes	2,727,374,594,530 (2,414,143,369,422–3,081,676,041,459)	2,885,719,966,082 (2,556,192,567,674–3,262,356,864,586)	2,928,451,464,081 (2,582,952,123,803–3,320,729,580,616)	2,998,917,382,742 (2,643,221,041,449–3,403,164,743,862)	3,056,462,395,813 (2,689,117,908,208–3,473,562,490,430)	3,214,956,642,013 (2,826,725,789,262–3,654,977,949,664)
Japan	Group I	203,458,150,952 (179,195,387,462–234,223,974,358)	214,780,824,212 (190,829,417,510–245,762,376,919)	213,192,800,839 (189,267,610,474–243,984,251,111)	210,754,570,695 (187,536,228,920–241,284,977,614)	214,919,284,120 (192,810,039,426–246,128,566,920)	226,897,843,522 (204,487,492,050–257,870,791,659)
Japan	NCD	2,234,619,662,860 (1,967,536,482,932–2,533,076,170,039)	2,334,317,772,537 (2,056,895,034,029–2,650,763,517,851)	2,400,889,786,629 (2,108,521,807,308–2,734,629,495,977)	2,482,897,469,679 (2,177,331,281,741–2,831,585,618,364)	2,556,101,700,507 (2,237,644,748,726–2,920,442,417,184)	2,710,630,609,692 (2,365,934,622,734–3,093,781,826,050)
Japan	Injuries	289,296,780,719 (265,712,328,489–317,604,555,165)	336,621,369,332 (288,131,836,840–397,907,882,988)	314,369,076,214 (287,216,097,805–347,760,338,131)	305,265,342,368 (278,954,541,669–337,677,638,061)	285,441,411,186 (258,912,328,613–319,620,192,626)	277,428,188,798 (250,293,288,954–314,548,341,441)
Singapore	All causes	56,784,111,462 (50,878,915,406–63,421,141,285)	58,415,204,983 (51,870,008,695–65,923,623,729)	60,047,172,302 (52,891,517,248–68,426,050,060)	62,909,665,504 (54,925,620,852–71,969,389,495)	66,593,466,416 (57,926,514,914–76,254,570,224)	70,507,064,642 (61,264,950,022–81,334,240,771)
Singapore	Group I	6,552,626,701 (5,868,446,047–7,426,308,116)	6,263,678,215 (5,546,330,777–7,207,237,594)	6,422,262,560 (5,637,369,852–7,472,774,857)	6,422,023,249 (5,650,331,151–7,526,116,766)	6,866,480,390 (6,062,613,807–7,960,270,162)	7,445,991,393 (6,564,285,739–8,537,522,630)
Singapore	NCD	43,894,846,932 (39,075,214,899–49,291,868,033)	46,317,724,358 (40,894,088,946–52,376,452,458)	48,125,439,184 (42,131,458,418–54,876,033,388)	51,176,117,119 (44,496,142,877–58,686,429,162)	54,874,926,581 (47,241,145,644–63,256,634,183)	58,288,384,486 (50,018,284,184–67,592,950,793)
Singapore	Injuries	6,336,637,828 (5,785,405,139–7,001,943,406)	5,833,802,409 (5,210,416,740–6,576,217,413)	5,499,470,558 (4,888,516,231–6,222,264,050)	5,311,525,136 (4,697,290,181–6,102,268,109)	4,852,059,445 (4,239,905,469–5,659,002,987)	4,772,688,763 (4,121,312,956–5,644,343,724)
South Korea	All causes	1,076,547,649,224 (971,657,815,241–1,191,592,096,656)	1,059,998,940,988 (955,705,226,343–1,179,461,542,516)	1,022,047,377,420 (907,127,791,150–1,152,873,861,833)	998,125,139,406 (878,064,903,004–1,135,777,163,040)	1,017,565,581,784 (887,226,214,483–1,165,044,680,422)	1,092,054,867,748 (954,433,211,197–1,249,270,868,908)
South Korea	Group I	106,489,596,873 (90,837,422,045–126,159,320,656)	84,456,158,345 (71,580,768,367–100,688,729,444)	75,677,626,236 (64,200,016,712–90,237,014,939)	66,483,290,714 (54,936,538,278–81,546,890,270)	65,599,648,277 (53,795,553,880–81,633,289,342)	65,106,090,521 (53,554,773,955–80,726,678,996)
South Korea	NCD	773,795,064,858 (694,092,661,017–865,443,184,347)	775,978,562,123 (690,094,911,253–872,093,260,536)	775,806,807,775 (683,186,464,046–880,178,354,939)	780,192,124,919 (679,989,296,677–890,300,059,387)	805,108,612,059 (696,092,024,690–927,653,822,738)	883,949,303,912 (767,435,822,656–1,014,731,722,221)
South Korea	Injuries	196,262,987,493 (182,882,323,629–211,683,317,137)	199,564,220,521 (185,454,965,445–216,165,107,925)	170,562,943,409 (158,653,763,852–185,981,190,882)	151,449,723,781 (139,314,892,541–165,589,192,948)	146,857,321,447 (136,049,514,286–160,520,470,723)	142,999,473,315 (130,436,529,379–157,875,121,361)
Western Europe	All causes	11,181,328,271,182 (10,017,133,716,959–12,515,174,594,271)	11,077,408,416,040 (9,868,393,694,942–12,455,807,351,951)	10,890,229,681,189 (9,651,406,951,701–12,306,419,772,295)	10,660,731,547,074 (9,379,417,279,229–12,133,562,423,403)	10,623,080,425,443 (9,295,624,326,214–12,141,621,171,905)	11,018,979,718,475 (9,629,793,440,039–12,602,529,037,768)
Western Europe	Group I	643,293,744,601 (573,748,712,450–734,429,461,606)	657,690,229,830 (589,791,886,445–750,987,961,765)	570,369,682,962 (499,946,126,644–663,059,979,330)	541,136,837,279 (471,643,600,644–635,834,921,335)	523,198,024,600 (452,609,663,877–621,947,536,340)	511,590,801,819 (439,347,327,833–612,047,621,303)
Western Europe	NCD	9,304,523,100,049 (8,332,494,602,083–10,391,322,134,145)	9,282,147,674,321 (8,266,535,542,028–10,415,672,347,299)	9,258,456,351,628 (8,209,017,075,773–10,432,936,355,384)	9,163,766,576,954 (8,071,172,024,282–10,402,788,016,480)	9,233,399,694,256 (8,098,999,435,292–10,519,080,788,330)	9,660,986,604,489 (8,455,579,844,562–11,020,055,198,222)
Western Europe	Injuries	1,233,511,426,533 (1,105,885,052,265–1,388,158,913,176)	1,137,570,511,889 (1,011,880,771,764–1,289,285,842,749)	1,061,403,646,599 (937,493,158,481–1,209,906,325,118)	955,828,132,841 (836,237,001,028–1,096,708,055,819)	866,482,706,588 (750,448,509,660–1,015,148,677,692)	846,402,312,167 (730,143,832,745–997,442,997,054)
Andorra	All causes	1,098,127,029 (945,901,080–1,275,103,706)	1,281,623,758 (1,097,308,236–1,488,497,628)	1,290,748,157 (1,099,072,090–1,503,291,629)	1,486,403,736 (1,261,527,671–1,742,780,408)	1,642,541,856 (1,396,614,988–1,926,191,677)	1,723,613,546 (1,465,334,478–2,014,963,787)
Andorra	Group I	71,433,938 (51,599,037–153,591,272)	86,458,638 (56,277,284–220,439,295)	74,959,138 (52,672,869–159,265,085)	80,474,316 (58,964,075–144,214,880)	84,274,996 (62,170,191–140,912,527)	82,909,152 (61,951,480–125,209,802)
Andorra	NCD	910,431,160 (777,415,697–1,057,177,660)	1,068,690,632 (904,121,862–1,246,580,270)	1,092,196,116 (926,882,841–1,276,646,266)	1,270,353,890 (1,071,798,555–1,486,259,532)	1,420,719,232 (1,202,963,947–1,662,633,696)	1,504,124,282 (1,279,740,367–1,758,213,621)
Andorra	Injuries	116,261,931 (92,351,437–137,982,960)	126,474,488 (95,689,681–152,334,919)	123,592,903 (98,637,636–148,952,677)	135,575,530 (111,562,085–164,917,395)	137,547,629 (111,011,280–168,542,629)	136,580,112 (110,777,001–168,995,611)
Austria	All causes	234,572,967,920 (210,830,258,365–261,323,144,289)	229,274,666,903 (204,989,267,659–257,123,343,435)	222,120,048,204 (197,116,958,281–250,773,683,213)	216,429,872,727 (190,168,990,839–246,299,718,415)	216,291,654,974 (189,441,965,077–246,773,343,245)	223,316,989,384 (194,786,185,727–255,530,238,884)
Austria	Group I	10,631,722,395 (9,258,387,577–12,402,957,886)	9,711,436,040 (8,320,302,174–11,578,134,615)	8,437,559,327 (7,060,558,031–10,200,738,612)	8,218,044,562 (6,855,471,278–9,975,345,211)	7,771,529,870 (6,400,791,871–9,615,441,226)	7,383,506,123 (5,977,089,749–9,308,497,449)

Appendix Table 3. DALYs for all causes and level 1 cause groups by location for 1990 - 2015, both sexes combined.

	Cause	1990	1995	2000	2005	2010	2015
Austria	NCD	194,533,004,551<br>(174,624,899,670–216,693,581,821)	193,105,556,391<br>(172,127,429,846–216,306,204,452)	189,801,072,018<br>(168,435,124,838–213,473,764,985)	186,453,084,213<br>(163,956,762,037–210,978,130,737)	188,605,214,898<br>(165,592,848,007–214,632,680,888)	196,870,856,574<br>(172,340,398,603–224,341,967,391)
Austria	Injuries	29,408,240,974<br>(26,519,690,774–32,745,384,024)	26,457,674,501<br>(23,726,378,229–29,724,945,898)	23,881,417,160<br>(21,182,479,867–27,076,564,372)	21,758,743,951<br>(19,147,780,169–25,115,327,766)	19,914,910,206<br>(17,307,466,951–23,187,240,340)	19,062,626,687<br>(16,496,151,280–22,349,441,733)
Belgium	All causes	300,693,980,374<br>(269,991,196,247–335,566,964,275)	299,226,509,044<br>(267,834,689,972–335,186,943,534)	297,793,319,432<br>(265,422,645,248–334,536,653,535)	287,880,986,702<br>(254,856,967,120–325,945,087,410)	289,260,708,318<br>(254,276,327,244–328,641,766,604)	300,545,301,791<br>(262,236,742,648–342,050,995,156)
Belgium	Group I	14,752,167,339<br>(13,196,962,638–16,669,617,858)	14,798,233,444<br>(13,307,413,244–16,742,895,258)	14,976,778,919<br>(13,473,398,927–16,900,153,560)	14,842,156,833<br>(13,236,929,605–16,816,066,485)	14,678,434,262<br>(13,067,375,233–16,767,479,455)	14,440,887,877<br>(12,664,360,076–16,550,906,427)
Belgium	NCD	249,683,934,663<br>(222,994,294,389–279,216,294,455)	249,043,360,524<br>(221,839,403,394–279,819,796,126)	247,813,958,058<br>(220,332,311,978–278,913,332,993)	241,381,916,040<br>(212,715,481,378–273,484,176,539)	244,420,870,323<br>(214,471,125,199–278,495,763,348)	256,184,296,872<br>(223,025,606,455–292,640,937,880)
Belgium	Injuries	36,257,878,371<br>(32,902,485,972–40,346,189,798)	35,384,915,075<br>(32,119,316,751–39,529,363,605)	35,002,582,455<br>(31,421,959,773–39,244,449,282)	31,656,913,829<br>(28,139,938,260–36,039,164,044)	30,161,403,734<br>(26,694,477,623–34,493,116,885)	29,920,117,042<br>(26,322,550,315–34,347,348,509)
Cyprus	All causes	16,608,095,033<br>(14,773,251,140–18,701,443,181)	17,338,064,093<br>(15,405,360,664–19,532,692,318)	17,282,842,012<br>(15,263,298,147–19,596,754,438)	17,885,723,484<br>(15,693,573,327–20,403,054,560)	18,824,878,965<br>(16,319,670,180–21,767,000,582)	19,071,686,972<br>(16,345,178,008–22,178,336,173)
Cyprus	Group I	1,553,232,311<br>(1,382,961,434–1,746,094,771)	1,305,570,347<br>(1,154,110,197–1,497,616,106)	1,096,009,136<br>(953,462,263–1,277,156,042)	979,508,293<br>(838,633,149–1,160,827,666)	940,067,638<br>(788,941,219–1,134,850,046)	894,532,429<br>(742,502,621–1,098,566,986)
Cyprus	NCD	13,066,761,525<br>(11,532,590,194–14,789,567,061)	13,999,409,600<br>(12,355,730,321–15,887,369,139)	14,173,663,918<br>(12,479,505,238–16,080,401,122)	14,781,349,780<br>(12,884,571,125–16,940,018,215)	16,036,625,648<br>(13,841,559,842–18,584,559,515)	16,448,586,094<br>(14,041,530,623–19,196,136,763)
Cyprus	Injuries	1,988,101,197<br>(1,788,573,709–2,234,444,712)	2,033,084,147<br>(1,823,940,472–2,294,606,322)	2,013,168,957<br>(1,804,413,912–2,276,811,752)	2,124,865,410<br>(1,887,433,243–2,412,983,362)	1,848,185,679<br>(1,611,889,660–2,155,366,248)	1,728,568,449<br>(1,485,488,734–2,050,139,632)
Denmark	All causes	165,557,694,395<br>(149,845,027,080–183,551,987,920)	165,885,350,409<br>(149,630,081,202–184,069,273,475)	156,530,896,743<br>(139,949,856,076–175,240,792,345)	151,073,382,829<br>(134,098,911,381–170,403,471,200)	149,007,580,527<br>(131,718,867,419–168,266,551,655)	149,049,008,678<br>(131,111,774,809–169,306,735,274)
Denmark	Group I	7,897,786,003<br>(7,072,754,637–9,029,651,973)	8,471,592,972<br>(7,615,740,801–9,663,280,242)	7,113,188,666<br>(6,174,064,457–8,387,846,389)	7,340,018,479<br>(6,359,993,510–8,668,076,720)	7,307,485,203<br>(6,308,630,461–8,705,825,975)	6,931,627,581<br>(5,925,081,664–8,295,188,407)
Denmark	NCD	138,815,769,342<br>(125,455,622,781–153,750,996,028)	141,173,810,668<br>(127,324,535,932–156,691,282,980)	135,785,245,146<br>(121,569,698,213–151,854,477,032)	132,196,462,205<br>(117,397,464,798–148,420,266,181)	131,468,181,464<br>(116,232,190,138–148,216,486,279)	132,420,066,521<br>(116,089,988,198–149,876,045,553)
Denmark	Injuries	18,844,139,050<br>(17,047,134,402–21,045,441,771)	16,239,946,769<br>(14,639,644,762–18,117,395,957)	13,632,462,932<br>(12,166,326,680–15,422,757,520)	11,536,902,145<br>(10,219,053,922–13,230,858,888)	10,231,913,860<br>(8,948,814,373–11,852,965,881)	9,697,314,575<br>(8,397,757,962–11,301,081,142)
Finland	All causes	152,579,718,174<br>(137,092,618,842–170,409,221,474)	147,065,857,484<br>(130,772,849,098–164,980,646,022)	146,090,308,744<br>(129,308,348,127–164,781,135,427)	145,681,142,365<br>(128,497,927,691–165,436,345,913)	146,328,760,899<br>(128,662,016,739–166,846,739,015)	148,428,688,316<br>(130,307,577,550–169,701,183,786)
Finland	Group I	9,101,821,469<br>(7,903,456,491–10,659,963,558)	8,448,555,719<br>(7,288,944,970–9,950,340,705)	7,358,549,110<br>(6,309,339,087–8,729,790,225)	5,987,723,565<br>(4,975,702,185–7,357,556,615)	5,262,518,448<br>(4,225,827,141–6,620,587,543)	4,994,472,156<br>(3,963,362,073–6,288,477,062)
Finland	NCD	121,359,633,188<br>(108,562,265,465–135,894,568,242)	119,385,546,799<br>(105,847,811,764–134,106,334,230)	120,921,348,415<br>(106,892,439,458–136,534,483,756)	122,205,172,553<br>(107,588,218,632–138,154,396,124)	125,101,013,105<br>(110,032,142,557–141,720,381,758)	128,517,653,619<br>(112,652,907,429–145,779,758,313)
Finland	Injuries	22,118,263,517<br>(20,320,712,404–24,222,964,002)	19,231,754,966<br>(17,593,146,899–21,195,246,080)	17,810,411,219<br>(16,181,288,265–19,812,521,385)	17,488,246,247<br>(15,583,039,269–19,843,014,288)	15,965,229,345<br>(14,017,459,125–18,371,282,087)	14,916,562,541<br>(12,977,879,620–17,366,476,951)
France	All causes	1,561,005,290,498<br>(1,391,046,473,761–1,757,575,105,389)	1,556,131,916,177<br>(1,381,164,640,351–1,759,396,897,199)	1,561,163,562,154<br>(1,379,996,220,108–1,768,793,662,774)	1,537,557,333,385<br>(1,349,246,577,660–1,756,667,730,995)	1,547,579,127,200<br>(1,353,537,539,687–1,774,228,625,195)	1,618,463,114,358<br>(1,415,134,887,823–1,855,887,031,610)
France	Group I	103,868,078,492<br>(93,391,617,894–117,882,362,148)	106,180,090,394<br>(95,713,049,803–120,950,228,045)	87,144,857,382<br>(76,740,208,720–101,807,667,326)	81,984,341,320<br>(70,955,612,850–97,345,063,769)	80,723,013,603<br>(69,363,037,730–96,514,704,839)	79,277,333,340<br>(67,357,048,494–95,594,981,148)
France	NCD	1,211,716,615,568<br>(1,073,611,197,588–1,366,577,917,991)	1,221,764,398,572<br>(1,077,509,563,674–1,382,816,410,022)	1,261,335,255,154<br>(1,113,298,452,656–1,425,510,869,298)	1,266,662,885,399<br>(1,109,532,459,760–1,441,742,930,911)	1,291,799,715,085<br>(1,127,319,817,986–1,478,017,646,393)	1,365,310,765,210<br>(1,192,827,443,274–1,561,622,486,105)
France	Injuries	245,420,596,438<br>(222,592,895,931–273,432,094,185)	228,187,427,212<br>(204,925,148,906–255,556,678,182)	212,683,449,619<br>(190,798,954,630–238,600,718,180)	188,910,106,666<br>(167,348,693,110–215,472,911,622)	175,056,398,511<br>(153,685,068,500–201,492,600,145)	173,875,015,808<br>(152,081,742,631–200,579,025,146)
Germany	All causes	2,541,145,292,558<br>(2,287,070,359,098–2,827,433,888,760)	2,485,317,263,105<br>(2,221,464,865,855–2,779,690,226,999)	2,392,206,177,104<br>(2,127,609,745,669–2,693,038,067,894)	2,315,056,656,719<br>(2,044,353,602,699–2,622,992,786,933)	2,287,755,868,808<br>(2,017,680,299,392–2,593,626,086,120)	2,390,573,023,996<br>(2,102,358,334,613–2,712,290,452,026)
Germany	Group I	119,744,471,419<br>(104,562,034,430–139,937,310,914)	109,958,921,011<br>(94,381,985,475–130,336,013,063)	100,698,668,439<br>(85,400,875,435–121,113,842,433)	98,158,068,609<br>(83,526,492,497–117,890,514,323)	96,858,615,439<br>(82,501,565,107–115,713,432,226)	97,435,332,844<br>(83,163,759,587–117,377,643,882)
Germany	NCD	2,175,627,885,897<br>(1,965,167,946,826–2,415,175,900,507)	2,146,375,873,050<br>(1,922,578,304,944–2,395,021,633,622)	2,086,715,486,603<br>(1,861,483,759,540–2,342,984,266,845)	2,037,396,134,886<br>(1,806,495,314,137–2,300,265,161,414)	2,028,140,836,556<br>(1,791,830,059,342–2,294,815,056,075)	2,130,912,055,128<br>(1,876,801,087,088–2,409,242,929,803)
Germany	Injuries	245,772,935,243<br>(219,317,742,340–277,616,621,947)	228,982,469,044<br>(202,716,280,999–259,834,644,497)	204,792,022,062<br>(179,910,134,464–235,047,055,979)	179,502,453,224<br>(155,623,378,834–209,175,059,652)	162,756,416,813<br>(139,649,989,287–190,644,431,404)	162,225,636,024<br>(137,903,518,106–191,817,103,335)
Greece	All causes	276,460,899,142<br>(246,490,897,419–310,837,935,043)	286,630,952,464<br>(254,849,396,621–323,763,756,455)	294,452,462,299<br>(261,563,571,006–333,043,613,150)	294,193,410,168<br>(259,997,567,868–333,210,239,965)	291,892,460,149<br>(257,000,869,147–332,418,478,526)	294,661,443,855<br>(260,094,848,751–334,043,920,379)
Greece	Group I	13,746,858,539<br>(11,883,622,726–16,318,540,292)	13,511,593,524<br>(11,668,098,811–16,072,987,167)	12,772,213,737<br>(10,937,578,865–15,216,949,885)	12,268,515,703<br>(10,405,957,263–14,576,957,909)	11,740,923,422<br>(9,945,306,285–14,183,485,478)	10,587,869,940<br>(8,764,555,082–12,875,630,174)
Greece	NCD	231,878,744,465<br>(206,767,262,537–259,677,586,917)	242,479,346,076<br>(215,509,813,232–272,767,288,591)	252,088,071,252<br>(223,849,462,315–283,892,692,754)	255,223,734,574<br>(226,003,774,853–287,771,594,617)	257,018,401,380<br>(226,969,800,020–290,936,029,468)	263,529,619,240<br>(232,339,171,379–298,756,345,101)
Greece	Injuries	30,835,296,137<br>(27,158,122,262–35,200,040,995)	30,640,012,864<br>(26,973,036,561–34,967,189,544)	29,592,177,310<br>(25,967,149,560–33,964,107,802)	26,701,159,891<br>(23,268,421,613–30,901,789,966)	23,133,135,347<br>(19,902,262,896–27,250,654,188)	20,543,954,675<br>(17,489,158,452–24,441,853,575)
Iceland	All causes	5,691,877,237<br>(5,023,996,307–6,470,507,530)	6,235,637,724<br>(5,400,410,028–7,181,132,448)	5,793,271,787<br>(5,027,087,637–6,660,181,473)	5,931,746,698<br>(5,091,158,659–6,885,077,654)	6,079,603,253<br>(5,201,625,341–7,063,914,952)	6,294,124,500<br>(5,343,078,668–7,348,134,462)
Iceland	Group I	408,879,811<br>(362,415,611–469,834,160)	401,779,680<br>(352,029,406–464,887,977)	346,181,449<br>(294,454,491–413,519,132)	329,913,685<br>(277,471,547–400,273,864)	326,250,225<br>(268,714,221–400,173,192)	317,916,297<br>(262,027,861–393,232,857)
Iceland	NCD	4,587,665,419<br>(4,012,888,154–5,243,530,764)	4,721,479,080<br>(4,101,147,401–5,408,727,230)	4,821,968,232<br>(4,164,024,253–5,559,520,743)	4,992,883,418<br>(4,285,125,377–5,777,916,145)	5,203,822,434<br>(4,420,775,353–6,047,566,120)	5,448,598,677<br>(4,606,910,265–6,352,198,695)
Iceland	Injuries	695,332,007<br>(621,822,645–782,186,001)	1,112,378,963<br>(886,191,913–1,395,466,130)	625,122,106<br>(551,638,092–716,983,352)	608,949,594<br>(529,675,333–706,147,846)	549,530,595<br>(479,039,000–634,011,073)	527,609,525<br>(455,469,578–615,089,021)
Ireland	All causes	89,401,388,891<br>(79,305,683,173–101,005,208,038)	89,120,372,089<br>(78,919,999,163–101,043,162,889)	92,633,424,792<br>(81,514,647,762–105,189,291,765)	92,430,953,739<br>(80,211,806,289–106,555,686,772)	94,668,918,054<br>(81,191,375,940–109,549,584,359)	98,887,054,927<br>(85,056,737,357–114,812,193,890)
Ireland	Group I	6,467,453,308<br>(5,629,663,614–7,543,272,056)	6,260,338,749<br>(5,463,169,526–7,320,516,205)	6,248,116,967<br>(5,445,591,286–7,308,334,244)	5,810,293,389<br>(4,930,080,797–6,990,056,837)	5,805,199,324<br>(4,851,586,046–7,165,299,248)	5,632,628,733<br>(4,633,820,502–7,012,657,051)
Ireland	NCD	73,318,435,159<br>(64,586,324,652–82,943,623,603)	73,920,658,425<br>(65,125,725,659–83,962,020,589)	77,057,991,936<br>(67,637,294,599–87,632,890,641)	78,380,321,181<br>(67,808,622,282–90,295,618,548)	81,133,101,773<br>(69,547,431,196–93,971,310,959)	85,709,431,892<br>(73,159,649,030–99,271,620,799)
Ireland	Injuries	9,615,500,424<br>(8,580,589,628–10,799,449,785)	8,939,374,916<br>(8,013,756,109–10,044,815,092)	9,327,315,889<br>(8,387,274,628–10,486,907,692)	8,240,339,168<br>(7,250,133,730–9,429,511,618)	7,730,616,957<br>(6,760,313,051–8,929,941,893)	7,544,994,303<br>(6,548,570,214–8,809,365,071)
Israel	All causes	101,208,196,819<br>(89,880,273,217–114,552,831,276)	118,489,486,926<br>(104,727,355,115–134,149,397,170)	128,630,064,025<br>(112,979,946,188–146,560,021,473)	134,740,795,078<br>(117,599,702,036–154,023,478,343)	141,330,725,838<br>(121,709,449,110–163,694,006,028)	150,360,280,845<br>(128,964,852,976–174,506,647,508)
Israel	Group I	10,097,671,284<br>(8,835,645,589–11,663,886,328)	10,111,496,600<br>(8,698,664,264–11,826,214,857)	10,655,747,557<br>(9,216,191,480–12,671,287,312)	10,658,070,567<br>(9,132,522,575–12,837,871,446)	11,180,249,156<br>(9,374,100,786–13,677,985,709)	11,203,897,262<br>(9,250,556,184–13,839,258,106)
Israel	NCD	78,719,654,570<br>(69,571,428,365–89,382,993,400)	94,135,208,756<br>(82,837,976,131–106,836,697,109)	102,607,893,961<br>(89,802,695,421–117,168,477,584)	108,173,606,639<br>(94,004,522,949–124,026,392,531)	115,633,351,172<br>(99,144,936,541–134,031,217,601)	124,488,218,260<br>(106,448,978,596–145,218,131,480)
Israel	Injuries	12,390,870,965<br>(11,078,975,316–13,947,643,546)	14,242,781,570<br>(12,755,542,735–16,030,717,765)	15,366,422,507<br>(13,679,568,076–17,331,218,068)	15,909,117,873<br>(14,138,236,796–18,040,386,308)	14,517,125,509<br>(12,812,274,675–16,787,611,075)	14,668,165,323<br>(12,825,122,763–17,072,784,731)
Italy	All causes	1,620,506,343,270<br>(1,443,987,853,694–1,821,621,093,428)	1,605,800,915,597<br>(1,425,437,842,573–1,809,752,220,741)	1,564,026,219,542<br>(1,379,275,984,369–1,773,240,956,618)	1,512,362,219,384<br>(1,323,933,047,263–1,727,484,022,079)	1,532,894,609,774<br>(1,333,322,952,777–1,756,359,935,350)	1,633,736,276,505<br>(1,423,216,346,286–1,875,247,040,039)

Appendix Table 3. DALYs for all causes and level 1 cause groups by location for 1990 - 2015, both sexes combined.

	Cause	1990	1995	2000	2005	2010	2015
Italy	Group I	78,884,407,388<br>(70,856,821,370–89,134,382,715)	81,938,051,439<br>(74,090,566,659–92,047,454,992)	59,933,268,304<br>(52,506,741,360–69,682,766,335)	55,835,366,634<br>(48,706,275,217–65,269,927,492)	54,964,715,908<br>(47,268,740,956–64,764,373,481)	52,866,052,647<br>(45,015,662,555–63,294,824,237)
Italy	NCD	1,372,485,104,105<br>(1,223,343,036,412–1,541,236,295,875)	1,364,979,052,881<br>(1,210,563,271,421–1,538,689,683,267)	1,356,621,825,297<br>(1,198,942,829,978–1,536,272,328,788)	1,325,849,051,594<br>(1,160,942,996,754–1,515,349,522,647)	1,362,061,620,063<br>(1,188,001,472,891–1,561,416,726,055)	1,465,463,713,286<br>(1,281,440,828,858–1,679,753,473,518)
Italy	Injuries	169,136,831,776<br>(149,836,328,660–192,618,923,257)	158,883,811,277<br>(139,721,236,070–182,359,404,560)	147,471,125,941<br>(128,933,906,390–170,343,200,816)	130,677,801,156<br>(113,047,019,081–153,124,339,089)	115,868,273,803<br>(98,874,848,293–137,758,729,148)	115,406,510,571<br>(98,232,605,230–137,410,618,960)
Luxembourg	All causes	11,964,656,260<br>(10,757,904,842–13,381,072,353)	11,666,197,570<br>(10,350,252,180–13,154,504,709)	11,413,673,364<br>(10,011,751,630–12,971,760,451)	11,233,414,494<br>(9,791,417,573–12,865,822,131)	11,585,195,924<br>(9,997,184,730–13,409,896,700)	12,298,263,002<br>(10,546,824,590–14,288,638,402)
Luxembourg	Group I	549,952,945<br>(481,038,459–640,370,590)	545,233,841<br>(470,312,081–641,949,653)	517,336,774<br>(440,876,214–617,129,291)	508,966,685<br>(426,650,526–613,535,972)	530,291,967<br>(440,353,444–647,820,281)	552,239,970<br>(455,128,426–682,553,887)
Luxembourg	NCD	9,943,777,337<br>(8,922,123,725–11,132,895,178)	9,776,676,743<br>(8,659,335,725–11,040,072,006)	9,671,141,149<br>(8,471,477,196–10,981,736,879)	9,650,806,792<br>(8,402,021,124–11,051,643,566)	10,057,554,044<br>(8,672,017,951–11,640,074,893)	10,729,443,688<br>(9,197,210,704–12,476,369,856)
Luxembourg	Injuries	1,470,925,978<br>(1,316,964,524–1,663,690,725)	1,344,286,986<br>(1,189,996,255–1,530,053,673)	1,225,195,441<br>(1,072,897,846–1,406,463,290)	1,073,641,017<br>(933,538,359–1,247,995,026)	997,349,913<br>(854,196,283–1,171,387,027)	1,016,579,343<br>(867,413,253–1,206,368,400)
Malta	All causes	8,462,413,648<br>(7,461,416,762–9,622,196,673)	8,564,743,091<br>(7,505,016,456–9,837,725,236)	8,904,750,525<br>(7,724,161,887–10,218,378,491)	9,037,611,356<br>(7,823,466,302–10,434,628,312)	9,598,137,801<br>(8,293,097,758–11,086,222,165)	9,936,937,927<br>(8,594,876,595–11,474,985,368)
Malta	Group I	622,492,181<br>(545,091,465–721,770,317)	628,787,023<br>(547,426,810–731,168,106)	550,833,825<br>(473,922,035–650,044,213)	495,872,104<br>(423,320,168–589,392,745)	488,699,483<br>(417,116,166–585,219,499)	476,338,834<br>(404,880,812–568,816,207)
Malta	NCD	7,077,923,455<br>(6,237,112,758–8,032,064,645)	7,207,855,272<br>(6,306,797,255–8,292,405,185)	7,620,795,796<br>(6,610,393,535–8,776,210,194)	7,860,591,731<br>(6,810,600,871–9,088,004,163)	8,457,498,139<br>(7,299,026,307–9,732,847,931)	8,831,601,759<br>(7,651,941,275–10,199,932,479)
Malta	Injuries	761,998,013<br>(660,729,406–883,654,508)	728,100,795<br>(624,141,030–852,753,840)	733,120,903<br>(623,966,548–861,848,145)	681,147,521<br>(572,289,605–813,919,301)	651,940,180<br>(543,594,477–791,656,450)	628,997,335<br>(515,982,554–771,830,008)
Netherlands	All causes	397,166,881,974<br>(352,384,391,591–446,886,084,462)	408,801,468,102<br>(362,640,059,019–461,690,642,391)	420,263,103,430<br>(371,112,514,604–475,697,991,267)	408,079,281,302<br>(357,067,021,552–465,926,381,728)	404,843,838,616<br>(353,010,100,989–465,172,521,219)	421,096,690,434<br>(366,123,368,713–482,206,473,060)
Netherlands	Group I	20,792,709,778<br>(18,390,637,509–23,980,394,211)	22,427,300,022<br>(19,992,134,776–25,676,354,046)	22,915,882,664<br>(20,438,957,132–26,210,481,524)	21,373,390,653<br>(18,914,692,646–24,729,199,006)	19,707,687,421<br>(17,251,606,498–23,064,809,831)	19,480,658,770<br>(17,002,070,778–22,947,132,494)
Netherlands	NCD	342,500,273,481<br>(304,004,681,817–385,272,406,884)	353,680,183,246<br>(312,863,283,354–399,670,796,138)	365,351,774,209<br>(321,388,459,898–412,994,532,617)	357,568,541,244<br>(312,400,385,640–407,890,899,570)	357,553,294,952<br>(312,003,861,109–408,863,107,992)	373,467,386,326<br>(323,603,048,940–427,321,794,200)
Netherlands	Injuries	33,873,898,715<br>(30,366,580,101–38,099,310,912)	32,693,984,834<br>(29,074,886,161–37,120,648,975)	31,995,446,557<br>(28,450,805,828–36,287,194,060)	29,137,349,405<br>(25,451,821,627–33,550,486,790)	27,582,856,244<br>(23,896,842,802–32,314,241,203)	28,148,645,339<br>(24,224,868,158–33,139,228,498)
Norway	All causes	125,509,534,272<br>(112,600,916,275–140,431,186,029)	120,859,307,073<br>(107,496,065,031–136,093,047,796)	120,918,991,148<br>(106,650,732,090–136,460,034,253)	115,173,779,343<br>(100,756,147,351–131,538,144,570)	116,814,671,464<br>(101,611,525,832–134,037,055,294)	117,628,519,121<br>(101,538,894,249–135,696,905,369)
Norway	Group I	7,524,469,067<br>(6,831,143,937–8,382,618,644)	6,636,888,607<br>(5,881,629,428–7,585,282,156)	6,106,122,761<br>(5,346,282,937–7,108,424,445)	5,710,322,856<br>(4,915,718,117–6,697,193,917)	5,618,084,458<br>(4,758,169,203–6,729,688,586)	5,503,483,900<br>(4,578,963,851–6,645,677,529)
Norway	NCD	104,245,613,775<br>(93,068,663,100–116,806,936,964)	102,948,357,430<br>(91,183,091,322–116,062,261,806)	103,569,129,774<br>(91,196,800,768–117,412,698,581)	99,374,455,322<br>(86,547,571,947–113,573,927,390)	101,480,329,632<br>(88,202,015,023–116,523,122,651)	103,182,881,616<br>(88,561,772,909–118,689,072,816)
Norway	Injuries	13,739,451,430<br>(12,408,317,212–15,362,256,837)	11,274,061,036<br>(10,108,425,860–12,776,805,962)	11,243,738,613<br>(10,008,227,884–12,714,620,744)	10,089,001,165<br>(8,896,360,336–11,595,151,322)	9,716,257,375<br>(8,525,397,626–11,208,137,051)	8,942,153,605<br>(7,796,273,395–10,395,104,450)
Portugal	All causes	317,234,865,652<br>(286,641,653,093–352,776,931,523)	315,681,139,125<br>(284,500,725,978–352,511,422,861)	312,414,483,706<br>(280,034,355,029–350,408,984,952)	299,015,456,243<br>(265,493,400,334–338,196,741,741)	288,074,762,991<br>(254,236,597,079–328,055,674,911)	286,494,410,720<br>(250,818,863,960–327,101,518,847)
Portugal	Group I	21,892,502,595<br>(20,001,505,955–24,444,642,854)	24,405,861,393<br>(22,472,257,333–26,992,136,568)	24,508,287,155<br>(22,478,285,839–27,240,912,316)	21,933,837,206<br>(19,891,914,510–24,658,257,500)	19,514,564,641<br>(17,437,443,708–22,250,255,784)	18,092,890,403<br>(15,950,416,336–20,971,814,145)
Portugal	NCD	249,362,016,336<br>(224,185,761,280–278,613,310,208)	250,721,448,207<br>(224,773,492,581–281,285,695,814)	252,800,089,484<br>(225,491,926,712–284,819,319,924)	247,612,096,827<br>(218,798,684,472–281,135,051,447)	243,602,167,225<br>(213,683,520,051–277,455,110,838)	245,517,019,004<br>(214,255,069,570–281,173,705,335)
Portugal	Injuries	45,980,346,721<br>(41,739,051,157–51,056,235,775)	40,553,829,525<br>(36,577,435,316–45,435,719,041)	35,106,107,067<br>(31,339,583,715–39,740,045,691)	29,469,522,210<br>(25,862,697,518–33,756,178,972)	24,958,031,125<br>(21,780,817,314–28,929,980,362)	22,884,501,314<br>(19,841,650,292–26,781,782,616)
Spain	All causes	1,050,199,051,379<br>(935,074,327,998–1,182,144,826,707)	1,060,910,792,156<br>(942,275,422,881–1,196,743,828,612)	1,053,481,362,392<br>(929,746,218,244–1,196,207,540,690)	1,077,429,950,103<br>(945,065,475,354–1,230,833,040,187)	1,070,861,869,882<br>(930,608,986,407–1,233,104,412,999)	1,102,159,239,695<br>(952,653,113,990–1,270,806,762,918)
Spain	Group I	73,579,536,149<br>(64,306,140,944–84,767,915,106)	84,699,436,096<br>(75,987,422,221–96,060,404,793)	62,816,257,019<br>(54,315,296,398–74,263,276,784)	63,464,035,162<br>(54,285,438,380–75,644,580,390)	60,388,308,528<br>(50,447,560,208–73,774,152,772)	58,140,841,913<br>(47,582,536,763–71,712,608,448)
Spain	NCD	848,359,997,271<br>(756,888,022,485–954,080,623,719)	865,750,142,350<br>(768,848,968,716–976,544,757,635)	883,696,830,659<br>(782,337,429,303–999,848,332,391)	915,274,286,748<br>(806,095,804,594–1,040,038,869,283)	927,394,059,958<br>(808,155,981,135–1,062,196,390,666)	964,292,503,876<br>(835,602,457,494–1,106,974,181,310)
Spain	Injuries	128,259,517,960<br>(115,017,569,692–144,512,142,860)	110,461,213,709<br>(97,576,568,413–126,225,268,417)	106,968,274,714<br>(94,260,863,343–122,429,028,174)	98,691,628,193<br>(85,951,143,511–115,168,723,523)	83,079,501,397<br>(70,689,358,492–98,856,648,964)	79,725,893,907<br>(67,912,605,529–95,727,998,851)
Sweden	All causes	246,672,533,302<br>(220,805,359,928–275,744,007,976)	238,298,629,169<br>(212,044,460,398–268,317,251,487)	234,605,278,677<br>(207,150,081,043–265,512,001,151)	231,034,918,587<br>(203,332,341,587–262,476,072,548)	228,473,172,040<br>(199,452,192,259–260,610,015,474)	236,046,229,924<br>(205,721,181,106–269,482,077,547)
Sweden	Group I	12,781,325,993<br>(11,344,371,248–14,714,760,622)	11,831,812,394<br>(10,275,565,000–13,911,339,035)	10,803,863,461<br>(9,131,747,739–13,056,097,896)	10,403,573,727<br>(8,773,896,246–12,724,351,952)	10,390,558,845<br>(8,621,025,321–12,675,160,682)	10,354,768,252<br>(8,511,915,423–12,718,439,343)
Sweden	NCD	210,925,166,612<br>(188,368,725,377–235,558,935,889)	205,382,433,975<br>(182,365,162,258–230,796,788,651)	204,286,809,023<br>(180,386,186,587–230,573,725,188)	201,747,210,424<br>(177,871,739,669–229,050,819,461)	200,192,588,880<br>(175,045,251,031–227,769,801,178)	207,923,274,092<br>(181,128,657,109–237,022,337,425)
Sweden	Injuries	22,966,040,696<br>(20,753,328,369–25,613,945,002)	21,084,382,799<br>(18,878,525,832–23,777,856,554)	19,514,606,193<br>(17,376,347,956–22,277,435,353)	18,884,134,436<br>(16,739,649,730–21,597,892,281)	17,890,024,316<br>(15,715,904,395–20,705,013,510)	17,768,187,580<br>(15,572,194,853–20,700,630,910)
Switzerland	All causes	189,291,963,309<br>(168,140,112,569–213,742,386,231)	189,143,358,634<br>(167,477,607,078–214,709,413,753)	183,710,069,590<br>(160,665,369,327–209,877,539,006)	179,464,215,476<br>(155,768,073,033–206,366,152,851)	181,978,339,834<br>(157,764,258,888–210,418,067,605)	188,363,751,562<br>(161,797,233,566–218,426,191,365)
Switzerland	Group I	10,208,790,233<br>(9,032,665,131–11,752,312,956)	11,714,089,486<br>(10,406,286,240–13,329,655,099)	9,072,844,383<br>(7,813,297,968–10,760,511,176)	8,121,323,529<br>(6,879,846,104–9,811,589,359)	8,009,747,594<br>(6,735,529,806–9,735,562,512)	8,113,976,808<br>(6,695,099,174–9,944,391,245)
Switzerland	NCD	150,740,811,339<br>(133,386,800,996–170,108,181,105)	154,087,102,482<br>(135,928,849,739–174,736,556,422)	153,936,157,971<br>(134,388,555,512–175,499,326,244)	153,198,893,518<br>(132,909,548,245–176,287,203,499)	157,352,431,204<br>(135,729,884,527–181,677,223,284)	163,740,086,111<br>(140,664,897,583–189,833,161,310)
Switzerland	Injuries	28,342,361,737<br>(25,265,027,848–32,066,118,343)	23,342,166,666<br>(20,638,226,197–26,602,411,972)	20,701,067,237<br>(18,199,734,049–23,670,136,655)	18,143,998,429<br>(15,800,938,057–21,008,572,050)	16,616,161,035<br>(14,343,810,584–19,626,510,322)	16,509,688,644<br>(14,240,694,418–19,550,454,455)
United Kingdom	All causes	1,757,306,446,355<br>(1,587,976,190,344–1,953,133,533,817)	1,704,948,041,379<br>(1,530,298,922,169–1,908,114,419,702)	1,653,802,370,361<br>(1,475,148,325,240–1,860,536,986,549)	1,606,895,200,220<br>(1,422,264,006,559–1,821,064,597,395)	1,576,615,505,131<br>(1,387,923,302,984–1,794,141,964,815)	1,599,271,121,470<br>(1,401,958,676,200–1,821,824,769,177)
United Kingdom	Group I	117,472,516,595<br>(106,552,062,811–132,193,673,171)	122,969,517,070<br>(111,767,414,792–137,950,399,891)	115,651,337,382<br>(104,224,457,102–131,023,932,919)	106,080,268,231<br>(94,097,221,246–121,714,328,581)	100,379,578,231<br>(88,575,062,304–116,536,804,784)	98,328,309,336<br>(85,956,054,244–114,694,097,868)
United Kingdom	NCD	1,505,541,075,634<br>(1,357,546,346,915–1,669,363,085,168)	1,457,466,904,377<br>(1,306,297,524,461–1,625,267,090,446)	1,417,617,589,985<br>(1,263,049,132,524–1,589,981,520,399)	1,387,390,840,980<br>(1,225,866,074,609–1,567,901,770,067)	1,370,009,395,942<br>(1,203,875,406,867–1,555,378,327,814)	1,401,246,414,807<br>(1,228,308,887,667–1,592,767,841,754)
United Kingdom	Injuries	134,292,854,125<br>(118,686,635,574–153,255,930,003)	124,511,619,932<br>(109,280,354,993–142,760,463,873)	120,533,442,993<br>(105,041,745,368–139,362,813,804)	113,424,091,010<br>(97,833,742,102–132,612,597,169)	106,226,530,958<br>(91,226,610,795–124,947,556,394)	99,696,397,327<br>(85,115,934,788–118,178,455,586)
England	All causes	1,445,933,130,810<br>(1,305,419,960,165–1,608,249,007,375)	1,401,941,052,351<br>(1,257,701,772,391–1,569,933,409,770)	1,359,514,511,148<br>(1,211,095,715,329–1,531,013,942,794)	1,318,539,854,647<br>(1,165,295,186,216–1,495,820,053,709)	1,292,404,040,820<br>(1,136,343,839,334–1,472,486,138,590)	1,313,643,379,896<br>(1,149,442,779,549–1,498,607,302,859)
England	Group I	97,200,740,071<br>(88,053,347,304–109,569,721,501)	102,834,709,085<br>(93,250,133,423–115,729,094,526)	97,381,563,749<br>(87,746,622,321–110,450,483,941)	89,025,493,963<br>(79,004,876,083–102,130,206,683)	84,292,543,451<br>(74,140,472,500–98,100,584,952)	82,664,635,439<br>(71,992,935,086–96,639,107,049)
England	NCD	1,240,509,686,307<br>(1,117,592,563,268–1,376,053,726,387)	1,199,138,643,355<br>(1,073,685,428,355–1,338,149,054,887)	1,165,169,615,020<br>(1,037,267,076,309–1,308,727,027,715)	1,138,509,787,668<br>(1,004,602,421,024–1,288,259,713,669)	1,123,763,561,557<br>(985,847,082,362–1,277,582,855,454)	1,151,080,920,508<br>(1,007,489,787,201–1,310,043,246,497)
England	Injuries	108,222,704,432<br>(95,425,284,663–123,789,416,350)	99,967,699,911<br>(87,479,611,468–115,154,651,743)	96,963,332,379<br>(84,210,127,111–112,366,161,521)	91,004,573,016<br>(78,352,475,319–106,767,932,208)	84,347,935,811<br>(72,183,844,882–99,869,729,787)	79,897,823,949<br>(67,876,200,230–94,958,009,811)

Appendix Table 3. DALYs for all causes and level 1 cause groups by location for 1990 - 2015, both sexes combined.

	Cause	1990	1995	2000	2005	2010	2015
Northern Ireland	All causes	45,645,353,897 (41,215,909,872–50,704,073,541)	44,445,345,394 (39,851,558,254–49,722,203,691)	43,251,714,195 (38,353,127,187–48,872,016,910)	43,904,435,856 (38,832,585,808–49,673,364,073)	45,362,930,554 (40,012,172,943–51,325,545,115)	45,157,217,462 (39,750,232,410–51,248,968,406)
Northern Ireland	Group I	3,906,713,483 (3,595,300,282–4,358,623,903)	3,817,585,799 (3,463,865,657–4,285,620,406)	3,374,862,944 (3,011,069,194–3,837,211,975)	3,111,871,585 (2,765,352,611–3,563,463,002)	3,031,829,190 (2,689,143,582–3,469,992,292)	2,916,118,439 (2,535,095,266–3,410,816,576)
Northern Ireland	NCD	37,391,482,187 (33,656,101,578–41,583,603,019)	36,517,167,795 (32,519,400,985–40,969,824,070)	35,918,111,683 (31,739,368,069–40,633,609,982)	36,640,565,497 (32,309,783,149–41,522,183,355)	37,576,803,754 (33,118,355,747–42,625,007,636)	38,452,651,888 (33,746,063,954–43,664,031,355)
Northern Ireland	Injuries	4,347,158,227 (3,905,022,728–4,918,415,669)	4,110,591,800 (3,659,491,956–4,652,375,458)	3,958,739,568 (3,501,919,944–4,522,205,635)	4,151,998,774 (3,637,591,905–4,757,171,636)	4,754,297,611 (4,059,207,917–5,524,197,231)	3,788,447,136 (3,275,414,416–4,454,209,024)
Scotland	All causes	173,089,178,122 (157,283,659,265–191,274,508,628)	167,539,844,463 (150,874,443,319–186,358,857,327)	162,598,182,673 (146,235,276,312–181,390,345,321)	157,913,023,541 (140,817,367,644–176,688,811,088)	153,535,006,594 (136,239,800,609–173,025,642,182)	153,986,187,764 (136,137,537,115–173,287,472,149)
Scotland	Group I	10,475,521,258 (9,567,417,036–11,627,963,283)	10,084,034,405 (9,188,910,284–11,240,996,478)	8,941,787,889 (8,058,867,134–10,069,810,745)	8,504,082,971 (7,644,437,772–9,612,736,434)	7,808,040,461 (6,948,145,629–8,952,940,129)	7,581,539,521 (6,674,421,353–8,710,385,105)
Scotland	NCD	147,630,351,741 (133,979,319,419–163,057,182,884)	143,438,836,073 (129,268,556,691–159,267,844,971)	140,261,894,519 (126,247,270,916–156,206,086,637)	137,069,306,793 (121,968,734,026–153,166,748,297)	134,261,759,059 (119,009,057,928–151,051,884,293)	135,732,916,194 (119,442,977,632–153,075,148,572)
Scotland	Injuries	14,983,305,123 (13,408,863,340–17,005,870,733)	14,016,973,984 (12,495,215,928–15,896,162,385)	13,394,500,265 (11,901,732,579–15,260,415,651)	12,339,633,777 (10,843,429,135–14,193,840,782)	11,465,207,074 (9,986,652,910–13,274,048,035)	10,671,732,049 (9,272,038,279–12,482,024,828)
Wales	All causes	92,638,783,526 (83,562,388,544–102,861,333,606)	91,021,799,171 (81,791,017,408–101,387,661,956)	88,437,962,344 (79,110,777,873–99,102,740,115)	86,537,886,176 (76,908,992,103–97,564,164,863)	85,313,527,162 (75,348,407,651–96,499,128,235)	86,484,336,348 (76,016,437,590–97,869,428,579)
Wales	Group I	5,889,541,783 (5,247,316,919–6,667,465,248)	6,233,187,780 (5,576,273,609–7,079,217,347)	5,953,122,800 (5,303,767,968–6,728,535,354)	5,438,819,712 (4,766,723,392–6,217,396,658)	5,247,165,128 (4,581,605,887–6,045,355,199)	5,166,015,937 (4,508,549,996–5,988,170,005)
Wales	NCD	80,009,555,400 (72,156,641,125–88,716,419,190)	78,372,257,154 (70,320,064,128–87,258,296,954)	76,267,968,763 (68,215,312,055–85,312,426,519)	75,171,181,022 (66,801,087,003–84,441,266,672)	74,407,271,572 (65,624,966,089–83,931,695,382)	75,979,926,217 (66,735,372,778–85,877,838,492)
Wales	Injuries	6,739,686,343 (5,932,501,108–7,738,382,939)	6,416,354,237 (5,614,625,670–7,386,340,107)	6,216,870,781 (5,415,324,676–7,214,494,438)	5,927,885,443 (5,104,648,766–6,943,016,577)	5,659,090,462 (4,840,671,853–6,682,855,989)	5,338,394,193 (4,575,064,929–6,325,267,241)
Southern Latin America	All causes	1,421,479,234,651 (1,300,747,348,236–1,562,889,153,740)	1,429,902,066,541 (1,298,737,906,044–1,580,125,899,957)	1,452,159,308,250 (1,310,204,154,190–1,615,867,544,370)	1,471,293,899,389 (1,320,773,919,026–1,642,084,216,030)	1,539,555,062,841 (1,379,297,595,270–1,721,327,538,341)	1,601,324,453,925 (1,425,849,850,417–1,799,737,354,916)
Southern Latin America	Group I	246,093,844,233 (231,232,389,814–265,726,315,981)	220,584,261,847 (205,398,183,975–239,875,428,870)	203,084,880,100 (188,105,787,027–221,295,137,311)	191,839,795,326 (176,387,872,458–211,498,871,907)	186,266,987,636 (170,446,321,508–205,687,949,053)	175,228,792,905 (159,515,190,995–194,545,565,651)
Southern Latin America	NCD	1,004,608,802,177 (906,791,580,968–1,114,572,528,416)	1,030,603,679,071 (924,838,573,186–1,151,691,023,797)	1,067,377,378,637 (952,444,205,970–1,197,975,693,114)	1,106,464,201,180 (982,257,964,637–1,246,656,377,909)	1,172,337,187,363 (1,038,793,099,391–1,322,904,978,239)	1,246,354,875,975 (1,100,069,625,415–1,410,773,153,722)
Southern Latin America	Injuries	170,776,588,241 (158,867,875,172–184,830,170,554)	178,714,125,623 (165,932,912,759–194,067,383,412)	181,697,049,513 (168,534,856,514–197,789,526,628)	172,989,902,883 (159,724,494,173–189,974,570,083)	180,950,887,842 (167,072,479,684–199,100,405,246)	179,740,785,046 (164,483,333,375–199,219,389,644)
Argentina	All causes	986,620,944,272 (904,651,746,574–1,082,544,104,832)	1,000,999,313,057 (912,733,722,151–1,102,824,401,953)	1,027,543,627,447 (931,726,917,657–1,137,466,129,833)	1,032,384,564,672 (931,117,821,978–1,146,817,926,921)	1,065,963,867,657 (958,835,153,416–1,186,768,120,105)	1,103,848,848,953 (987,422,101,178–1,238,012,377,735)
Argentina	Group I	180,366,773,609 (168,619,963,786–195,533,590,990)	167,603,066,251 (155,824,286,355–181,962,446,528)	159,118,684,911 (147,656,917,798–172,925,888,509)	152,891,940,445 (141,094,795,159–168,177,340,763)	148,000,683,957 (135,925,014,474–162,588,638,946)	138,495,670,590 (126,547,750,129–153,184,111,529)
Argentina	NCD	696,619,939,079 (630,452,072,385–770,940,999,469)	716,270,134,702 (645,685,170,609–797,080,081,927)	742,939,944,133 (665,902,646,545–829,038,708,346)	760,555,664,269 (678,250,819,075–853,659,456,227)	797,219,523,570 (709,754,015,721–896,981,784,540)	842,650,441,855 (745,711,879,520–952,216,064,051)
Argentina	Injuries	109,634,231,585 (101,504,788,816–118,859,786,764)	117,126,112,103 (108,429,195,194–127,701,985,118)	125,484,998,402 (116,278,590,956–136,591,760,808)	118,936,959,958 (109,654,459,800–130,662,225,399)	120,743,660,130 (111,283,624,706–133,118,249,147)	122,702,736,508 (112,321,092,773–136,231,779,634)
Chile	All causes	337,360,379,513 (306,150,390,778–372,948,370,901)	328,578,990,532 (294,441,518,264–367,457,591,616)	327,042,585,160 (289,338,534,274–369,841,965,017)	344,068,257,385 (303,823,550,943–390,317,171,584)	378,815,745,394 (333,788,193,340–429,208,214,448)	401,341,362,414 (351,631,959,391–454,136,669,117)
Chile	Group I	53,570,161,170 (50,343,875,267–57,753,025,969)	41,256,375,045 (38,226,641,524–45,080,598,585)	33,793,606,071 (30,846,130,909–37,703,995,744)	29,561,178,324 (26,540,013,502–33,685,091,998)	29,861,969,772 (26,677,795,509–33,984,148,951)	28,776,730,951 (25,562,945,481–32,761,296,269)
Chile	NCD	233,228,452,445 (207,468,161,409–262,224,064,997)	237,604,663,349 (209,480,675,527–269,404,895,171)	248,569,277,813 (216,652,510,791–284,135,677,983)	271,117,224,463 (236,658,221,230–310,415,291,904)	299,781,346,507 (262,286,044,002–342,121,664,833)	326,392,301,303 (283,147,650,556–371,903,108,879)
Chile	Injuries	50,561,765,897 (47,559,791,430–54,363,173,550)	49,717,952,138 (46,380,312,640–53,487,267,640)	44,679,701,277 (41,513,619,441–48,560,354,781)	43,389,854,598 (40,187,882,018–47,520,938,489)	49,172,429,115 (44,479,978,586–54,979,603,745)	46,172,330,160 (41,444,107,988–51,557,040,053)
Uruguay	All causes	97,439,247,973 (89,495,732,015–107,123,759,179)	100,251,709,274 (91,709,444,026–110,165,857,042)	97,488,086,177 (88,697,863,197–107,502,578,475)	94,754,101,885 (85,687,933,959–105,059,663,079)	94,687,725,245 (85,401,798,063–105,355,574,659)	96,043,411,526 (86,354,282,982–107,078,091,016)
Uruguay	Group I	12,149,287,738 (11,250,168,249–13,287,884,771)	11,716,268,135 (10,826,508,389–12,791,996,902)	10,163,342,412 (9,257,183,703–11,298,127,210)	9,377,834,998 (8,426,957,801–10,575,200,584)	8,395,661,938 (7,457,968,108–9,546,016,269)	7,948,320,252 (6,953,755,966–9,168,538,065)
Uruguay	NCD	74,716,603,512 (68,068,902,734–82,365,766,724)	76,674,110,860 (69,558,910,783–84,705,521,027)	75,802,212,118 (68,431,438,874–84,089,172,387)	74,722,210,883 (67,175,521,481–83,178,037,661)	75,265,949,708 (67,415,224,687–84,030,420,842)	77,237,783,160 (69,035,608,131–86,373,523,777)
Uruguay	Injuries	10,573,356,723 (9,768,118,861–11,569,367,698)	11,861,330,280 (11,032,437,220–12,858,101,805)	11,522,531,647 (10,722,979,873–12,541,589,768)	10,654,056,003 (9,856,316,456–11,620,593,002)	11,026,113,598 (10,225,112,531–12,072,923,900)	10,857,308,114 (10,005,913,816–11,944,471,860)
Central Europe, Eastern Europe, and Central Asia	All causes	15,744,933,780,975 (14,507,193,284,417–17,138,869,801,941)	18,250,227,155,159 (17,013,959,253,315–19,661,904,408,249)	17,445,674,699,501 (16,199,458,716,513–18,880,981,101,361)	17,462,518,725,164 (16,213,525,542,669–18,869,113,090,696)	15,577,448,152,755 (14,321,181,972,461–17,013,490,780,201)	15,306,856,696,659 (13,971,909,936,579–16,795,661,556,075)
Central Europe, Eastern Europe, and Central Asia	Group I	2,231,873,084,911 (2,094,895,745,197–2,405,927,555,070)	2,121,053,853,278 (1,980,017,225,409–2,293,964,828,122)	1,860,291,418,709 (1,732,079,332,985–2,014,302,268,499)	1,707,857,485,412 (1,583,096,365,362–1,864,560,034,830)	1,509,563,254,269 (1,385,094,325,527–1,658,577,295,682)	1,355,291,093,888 (1,228,876,099,025–1,509,077,293,745)
Central Europe, Eastern Europe, and Central Asia	NCD	11,254,094,914,459 (10,281,803,818,959–12,331,735,135,236)	13,145,912,586,305 (12,165,802,964,891–14,238,932,173,166)	12,940,404,194,559 (11,919,546,277,583–14,065,838,390,398)	13,244,489,580,271 (12,217,264,563,779–14,381,163,381,020)	12,158,802,262,512 (11,102,630,088,490–13,328,025,769,114)	12,187,932,061,400 (11,058,997,740,473–13,456,207,646,023)
Central Europe, Eastern Europe, and Central Asia	Injuries	2,258,965,781,604 (2,133,319,708,586–2,399,269,498,312)	2,983,260,715,576 (2,845,603,558,181–3,134,079,159,202)	2,644,979,086,233 (2,517,850,996,637–2,786,617,731,960)	2,510,171,659,481 (2,393,649,765,225–2,649,061,799,004)	1,909,082,635,974 (1,808,926,117,073–2,029,824,174,765)	1,763,633,541,371 (1,658,462,269,472–1,896,082,307,576)
Eastern Europe	All causes	8,737,330,924,323 (8,028,963,582,013–9,536,363,051,483)	11,017,818,185,569 (10,315,485,689,264–11,827,308,123,033)	10,737,840,599,462 (10,023,378,547,249–11,557,535,520,090)	10,904,074,424,406 (10,206,708,621,913–11,694,924,457,430)	9,219,119,918,627 (8,522,607,871,668–10,007,106,412,652)	9,098,775,579,020 (8,337,747,999,151–9,939,137,979,657)
Eastern Europe	Group I	760,779,671,070 (685,420,955,625–854,744,058,200)	792,490,310,535 (714,399,010,226–883,473,634,606)	828,267,809,691 (757,867,692,799–918,326,424,084)	827,291,358,068 (758,012,868,790–914,176,653,156)	722,758,226,885 (651,604,992,613–808,480,485,067)	675,623,852,051 (605,612,487,444–766,768,802,878)
Eastern Europe	NCD	6,542,588,779,272 (5,991,645,875,186–7,155,054,863,726)	8,161,549,328,110 (7,613,196,855,737–8,781,207,052,591)	8,004,373,273,955 (7,439,536,077,282–8,641,961,714,397)	8,246,083,813,704 (7,688,850,186,233–8,870,727,484,805)	7,207,818,951,783 (6,629,989,299,335–7,835,856,178,551)	7,241,509,036,337 (6,600,354,372,046–7,936,357,063,586)
Eastern Europe	Injuries	1,433,962,473,980 (1,354,783,639,958–1,525,130,870,733)	2,063,778,546,924 (1,975,998,784,612–2,160,517,300,454)	1,905,199,515,816 (1,819,236,536,097–2,003,255,756,005)	1,830,699,252,633 (1,745,954,861,527–1,922,380,728,716)	1,288,542,739,959 (1,226,343,647,029–1,364,907,171,226)	1,181,642,690,633 (1,113,471,638,107–1,267,726,596,690)
Belarus	All causes	375,557,789,801 (343,895,886,491–410,141,081,305)	429,418,321,377 (397,426,967,800–463,813,793,387)	439,950,671,295 (407,757,361,180–476,281,371,952)	432,785,267,139 (402,723,448,072–467,593,909,827)	405,156,765,127 (373,501,637,287–439,266,223,146)	412,943,454,692 (378,558,787,713–449,051,195,936)
Belarus	Group I	29,352,701,020 (25,367,118,140–34,088,547,608)	25,868,616,300 (22,434,238,820–30,220,791,373)	23,088,936,208 (20,060,038,505–26,740,842,193)	21,379,500,975 (18,390,798,620–24,837,426,359)	19,367,682,890 (16,338,613,556–22,657,443,319)	17,439,195,433 (14,692,912,913–20,811,655,808)
Belarus	NCD	286,111,161,837 (260,142,013,580–314,207,135,155)	330,061,260,897 (304,229,744,487–358,401,058,725)	341,794,532,671 (315,690,860,502–370,611,961,496)	342,074,866,961 (316,185,081,937–371,060,114,202)	325,515,461,707 (299,246,864,082–353,676,119,956)	337,318,218,058 (307,981,693,832–367,973,062,477)
Belarus	Injuries	60,093,926,945 (56,152,174,669–64,219,952,755)	73,488,444,180 (69,150,617,185–77,726,884,475)	75,067,202,416 (70,266,335,764–79,507,486,840)	69,330,899,203 (65,482,898,194–73,594,979,159)	60,273,620,529 (56,751,940,947–64,415,017,287)	58,186,041,201 (53,155,290,942–64,450,971,772)
Estonia	All causes	61,061,542,063 (56,104,459,754–66,529,861,406)	65,856,305,787 (61,270,313,372–70,942,228,220)	57,154,315,302 (52,697,925,082–62,194,113,953)	51,974,658,063 (47,597,040,090–56,919,390,955)	42,895,846,667 (38,587,414,358–47,721,523,794)	41,509,357,654 (37,030,489,327–46,417,891,752)
Estonia	Group I	4,128,459,403 (3,671,874,767–4,701,422,096)	4,000,833,584 (3,594,687,960–4,503,150,780)	3,086,045,368 (2,759,040,464–3,522,661,675)	2,558,372,863 (2,257,662,864–2,963,903,791)	2,063,038,434 (1,747,648,079–2,454,030,267)	1,781,314,875 (1,473,971,916–2,174,818,188)
Estonia	NCD	45,552,690,912 (41,651,479,718–49,844,681,084)	49,033,024,429 (45,324,592,485–53,136,706,652)	45,027,453,978 (41,295,867,342–49,158,680,675)	42,495,243,772 (38,796,648,630–46,745,840,378)	36,483,698,824 (32,645,981,763–40,610,860,258)	36,143,179,497 (32,012,294,969–40,418,340,673)

Appendix Table 3. DALYs for all causes and level 1 cause groups by location for 1990 - 2015, both sexes combined.

	Cause	1990	1995	2000	2005	2010	2015
Estonia	Injuries	11,380,391,748<br>(10,696,433,577–12,135,066,674)	12,822,447,774<br>(12,173,354,820–13,525,633,860)	9,040,815,956<br>(8,516,856,157–9,640,824,013)	6,921,041,429<br>(6,511,656,381–7,399,468,061)	4,349,109,409<br>(4,021,240,128–4,761,366,330)	3,584,863,283<br>(3,246,647,041–4,016,525,638)
Latvia	All causes	108,178,426,810<br>(99,914,159,937–117,590,614,179)	121,186,796,861<br>(113,325,237,722–130,312,074,102)	98,272,346,811<br>(90,561,446,868–107,091,647,305)	95,788,568,787<br>(88,086,109,690–104,325,023,947)	82,118,238,995<br>(74,822,963,534–90,226,079,561)	77,804,585,803<br>(70,321,368,319–86,153,731,763)
Latvia	Group I	7,311,435,587<br>(6,533,948,075–8,228,528,963)	7,330,092,378<br>(6,614,898,121–8,205,934,578)	5,548,079,134<br>(4,946,024,044–6,331,237,640)	4,859,624,370<br>(4,332,629,509–5,539,743,280)	4,211,127,281<br>(3,682,009,606–4,855,435,719)	3,663,244,797<br>(3,126,138,244–4,339,342,844)
Latvia	NCD	80,564,217,168<br>(73,830,224,194–87,967,626,362)	89,539,196,879<br>(83,073,812,823–96,640,066,372)	76,338,764,603<br>(69,695,783,378–83,551,459,308)	77,070,977,843<br>(70,724,412,544–84,250,238,002)	68,243,224,818<br>(61,873,363,137–75,156,460,698)	65,778,186,854<br>(59,204,286,128–73,010,487,146)
Latvia	Injuries	20,302,774,055<br>(19,221,871,121–21,585,315,540)	24,317,507,604<br>(23,147,504,090–25,584,392,675)	16,385,503,074<br>(15,534,732,334–17,467,246,781)	13,857,966,575<br>(13,080,699,654–14,764,784,564)	9,663,886,895<br>(9,048,741,122–10,409,069,264)	8,363,154,153<br>(7,761,032,453–9,148,259,583)
Lithuania	All causes	130,697,816,530<br>(119,435,965,268–143,191,093,651)	148,504,812,153<br>(137,330,699,879–161,266,304,199)	127,328,060,145<br>(116,290,998,535–140,014,555,559)	132,481,472,716<br>(121,667,239,293–144,725,970,013)	118,511,336,928<br>(107,901,144,470–130,447,194,130)	111,406,704,952<br>(100,713,162,237–123,105,523,457)
Lithuania	Group I	8,213,120,782<br>(7,217,418,324–9,431,397,139)	8,859,736,275<br>(7,900,587,482–10,072,975,675)	7,113,539,978<br>(6,291,651,980–8,225,622,808)	6,811,156,191<br>(6,011,764,660–7,839,345,009)	5,837,876,763<br>(5,104,237,169–6,769,075,746)	4,950,618,535<br>(4,261,985,821–5,775,439,469)
Lithuania	NCD	97,321,484,814<br>(88,228,136,623–107,303,050,476)	110,069,023,149<br>(101,020,300,164–120,198,950,816)	97,148,980,148<br>(88,083,678,848–107,210,045,749)	102,977,937,379<br>(93,875,791,694–113,131,530,477)	95,427,535,226<br>(86,476,763,449–105,627,070,195)	91,314,496,860<br>(81,910,711,926–101,300,435,344)
Lithuania	Injuries	25,163,210,934<br>(23,795,872,891–26,726,263,481)	29,576,052,729<br>(28,275,153,991–31,117,965,465)	23,065,540,019<br>(21,852,637,437–24,521,151,430)	22,692,379,146<br>(21,593,172,489–24,043,022,700)	17,245,924,939<br>(16,331,719,691–18,414,861,353)	15,141,589,557<br>(14,197,191,466–16,295,725,912)
Moldova	All causes	165,783,578,545<br>(152,863,380,180–180,334,833,260)	187,715,371,812<br>(174,927,302,706–201,922,716,148)	154,558,521,877<br>(142,125,879,551–168,331,577,704)	151,442,477,835<br>(139,351,122,422–165,092,219,471)	147,259,610,977<br>(135,225,535,281–160,640,791,890)	127,316,282,357<br>(114,900,205,785–140,923,026,995)
Moldova	Group I	21,450,703,122<br>(18,874,553,522–24,362,689,073)	21,796,646,374<br>(19,388,349,142–24,738,027,309)	18,447,469,480<br>(16,487,723,758–20,900,765,276)	13,519,987,417<br>(12,063,203,717–15,311,591,429)	12,605,278,837<br>(11,217,841,664–14,441,315,504)	9,693,378,937<br>(8,293,720,632–11,400,987,633)
Moldova	NCD	117,115,227,261<br>(107,411,681,331–128,210,968,533)	137,256,384,155<br>(127,205,994,938–148,357,199,314)	115,589,860,398<br>(105,813,647,983–126,509,521,954)	119,931,522,481<br>(110,012,712,329–131,078,062,804)	118,588,936,466<br>(108,516,008,268–129,858,812,105)	105,017,414,006<br>(94,382,145,762–116,439,609,797)
Moldova	Injuries	27,217,648,161<br>(25,481,629,429–29,047,051,172)	28,662,341,283<br>(27,048,939,160–30,423,379,909)	20,521,191,999<br>(19,292,487,785–21,969,550,160)	17,990,967,937<br>(16,930,617,146–19,207,703,799)	16,065,395,674<br>(15,152,804,721–17,146,719,656)	12,605,489,414<br>(11,652,236,978–13,661,089,878)
Russia	All causes	5,862,322,642,489<br>(5,388,403,449,439–6,404,011,979,590)	7,603,579,511,324<br>(7,124,617,042,628–8,156,521,933,012)	7,541,144,290,038<br>(7,054,743,369,710–8,106,974,681,155)	7,697,411,671,799<br>(7,217,819,610,178–8,241,256,668,204)	6,457,819,763,621<br>(5,973,179,638,177–7,004,556,582,602)	6,334,867,968,331<br>(5,799,490,052,783–6,935,210,254,097)
Russia	Group I	545,798,097,710<br>(488,042,468,033–621,465,831,577)	570,618,476,769<br>(508,465,980,184–641,683,938,236)	609,809,173,808<br>(555,329,070,755–680,631,890,070)	608,149,528,485<br>(552,711,728,931–676,424,432,402)	534,350,126,954<br>(477,661,417,393–602,784,021,393)	515,669,971,650<br>(455,985,587,799–592,039,123,302)
Russia	NCD	4,320,387,066,379<br>(3,958,054,774,126–4,719,744,455,569)	5,528,971,397,237<br>(5,165,165,046,137–5,944,040,428,417)	5,516,818,434,176<br>(5,132,432,301,376–5,947,889,052,264)	5,695,188,854,572<br>(5,319,858,602,198–6,115,278,147,428)	4,944,081,891,500<br>(4,550,785,799,747–5,370,311,434,779)	4,952,359,074,489<br>(4,517,522,044,003–5,437,490,238,891)
Russia	Injuries	996,137,478,400<br>(938,757,932,672–1,059,557,356,743)	1,503,989,637,318<br>(1,438,965,127,999–1,577,237,545,221)	1,414,516,682,053<br>(1,349,821,546,983–1,493,153,671,275)	1,394,073,288,742<br>(1,323,964,986,249–1,465,161,418,408)	979,387,745,168<br>(929,435,385,181–1,036,343,876,587)	866,838,922,191<br>(813,324,850,811–930,567,115,137)
Ukraine	All causes	2,033,729,128,085<br>(1,872,002,655,334–2,211,869,110,028)	2,461,557,066,254<br>(2,300,987,546,549–2,639,727,270,414)	2,319,432,393,993<br>(2,161,879,024,526–2,491,552,578,842)	2,342,190,308,066<br>(2,190,019,098,259–2,510,344,100,812)	1,965,358,356,312<br>(1,816,567,270,962–2,131,842,470,207)	1,992,927,225,232<br>(1,831,265,588,671–2,171,047,103,747)
Ukraine	Group I	144,525,153,446<br>(128,245,778,101–163,069,052,893)	154,015,908,854<br>(140,196,634,276–170,692,988,989)	161,174,565,715<br>(146,964,678,549–176,333,302,341)	170,013,187,768<br>(156,901,417,745–183,918,003,299)	144,323,095,726<br>(133,433,642,017–157,144,026,185)	122,426,127,825<br>(110,719,011,805–137,157,938,097)
Ukraine	NCD	1,595,536,930,902<br>(1,462,465,713,361–1,742,969,151,851)	1,916,619,041,363<br>(1,783,295,875,089–2,062,894,530,018)	1,811,655,247,979<br>(1,679,258,639,001–1,958,746,825,643)	1,866,344,410,697<br>(1,737,710,799,400–2,010,221,807,799)	1,619,478,203,241<br>(1,488,768,046,880–1,765,672,737,301)	1,653,578,466,572<br>(1,513,177,392,530–1,813,005,495,853)
Ukraine	Injuries	293,667,043,737<br>(276,892,919,717–313,189,929,554)	390,922,116,037<br>(372,305,822,874–411,708,312,759)	346,602,580,299<br>(329,291,176,515–366,413,032,284)	305,832,709,601<br>(291,212,400,963–322,546,971,431)	201,557,057,345<br>(189,118,937,250–217,304,906,027)	216,922,630,835<br>(195,085,908,521–241,237,174,092)
Central Europe	All causes	4,240,823,599,767<br>(3,887,302,702,001–4,640,705,427,798)	4,279,021,883,902<br>(3,925,044,164,445–4,679,969,647,475)	3,999,407,324,576<br>(3,645,390,345,012–4,402,843,342,513)	3,862,962,461,828<br>(3,506,779,722,228–4,269,743,342,666)	3,725,468,264,952<br>(3,365,847,582,298–4,134,820,945,721)	3,590,881,463,424<br>(3,222,080,462,524–4,010,398,079,205)
Central Europe	Group I	405,956,701,593<br>(375,072,060,928–442,139,087,175)	319,604,825,160<br>(294,765,498,110–351,418,587,405)	251,670,960,995<br>(227,846,528,750–280,859,271,650)	209,748,677,660<br>(188,318,949,781–236,632,829,339)	180,288,986,936<br>(159,507,918,295–207,437,642,119)	157,415,582,405<br>(137,897,527,637–183,068,521,945)
Central Europe	NCD	3,348,847,406,171<br>(3,056,454,606,375–3,671,798,139,519)	3,436,305,688,605<br>(3,135,669,714,540–3,769,966,010,244)	3,344,916,280,820<br>(3,042,683,369,320–3,684,793,029,589)	3,298,760,597,647<br>(2,986,392,719,848–3,648,289,014,481)	3,231,411,083,628<br>(2,912,492,997,202–3,587,217,729,588)	3,151,543,251,796<br>(2,822,593,804,540–3,514,636,477,454)
Central Europe	Injuries	486,019,492,003<br>(453,706,313,639–524,118,768,821)	523,111,370,137<br>(486,838,500,346–562,176,749,747)	402,820,082,761<br>(374,873,331,342–436,002,994,387)	354,453,186,521<br>(329,680,959,844–384,960,347,088)	313,768,194,389<br>(290,984,082,002–341,872,515,006)	281,922,629,223<br>(259,850,310,953–311,531,495,963)
Albania	All causes	92,017,603,439<br>(82,931,030,494–102,173,775,198)	86,744,926,307<br>(77,969,385,263–96,636,799,944)	79,156,330,968<br>(70,660,234,336–88,755,979,580)	73,828,104,526<br>(65,387,762,648–83,446,334,814)	71,345,307,699<br>(62,797,507,576–80,787,381,641)	72,418,734,733<br>(63,317,046,398–82,386,489,839)
Albania	Group I	29,321,517,388<br>(25,082,864,540–32,920,486,911)	22,696,602,442<br>(19,966,744,997–25,613,294,010)	14,030,512,156<br>(12,165,829,792–16,090,860,355)	8,989,292,808<br>(7,618,267,996–10,592,857,549)	6,343,669,084<br>(5,186,049,916–7,699,607,338)	5,439,164,383<br>(4,369,123,666–6,750,239,639)
Albania	NCD	53,210,650,976<br>(46,606,167,245–60,260,726,330)	53,789,658,973<br>(47,197,825,530–60,892,244,483)	56,029,893,380<br>(49,594,349,645–63,320,358,879)	58,887,509,539<br>(52,059,806,692–66,918,371,529)	60,091,876,401<br>(52,761,896,055–68,267,715,890)	62,482,824,463<br>(54,358,626,573–71,211,168,644)
Albania	Injuries	9,485,435,075<br>(8,669,467,624–10,359,875,251)	10,258,664,892<br>(9,359,104,132–11,094,556,738)	9,095,925,431<br>(8,169,460,577–9,839,754,701)	5,951,302,179<br>(5,426,638,394–6,556,889,990)	4,909,762,214<br>(4,424,246,587–5,465,422,967)	4,496,745,887<br>(3,968,129,956–5,067,322,929)
Bosnia and Herzegovina	All causes	125,063,809,514<br>(113,187,140,091–138,504,391,437)	172,283,901,806<br>(153,820,246,595–191,710,652,138)	102,341,938,447<br>(91,731,036,438–114,630,249,639)	102,627,974,540<br>(91,155,944,522–115,404,686,329)	103,818,640,542<br>(92,281,541,316–117,078,680,916)	103,781,471,058<br>(91,782,724,750–117,246,093,612)
Bosnia and Herzegovina	Group I	14,437,687,825<br>(12,893,841,272–16,414,401,358)	10,664,503,749<br>(9,340,350,130–12,156,307,551)	6,764,945,918<br>(5,859,183,387–7,895,046,656)	5,522,581,512<br>(4,660,034,691–6,530,043,026)	4,971,908,719<br>(4,166,018,023–5,936,534,708)	4,423,287,846<br>(3,611,715,508–5,385,646,713)
Bosnia and Herzegovina	NCD	93,057,246,152<br>(82,976,503,062–103,524,223,600)	88,708,006,781<br>(79,645,584,274–98,492,097,198)	84,778,273,034<br>(75,524,355,505–95,107,453,234)	87,617,973,557<br>(77,716,651,244–98,727,093,537)	89,707,860,157<br>(79,613,893,991–101,166,875,971)	90,901,602,027<br>(80,040,521,758–102,728,588,523)
Bosnia and Herzegovina	Injuries	17,568,875,536<br>(14,763,386,391–20,645,750,481)	72,911,391,277<br>(60,544,880,080–85,277,347,320)	10,798,719,494<br>(9,621,943,166–12,191,748,592)	9,487,419,471<br>(8,457,995,580–10,819,548,173)	9,138,871,666<br>(8,094,253,575–10,445,449,179)	8,456,581,185<br>(7,370,598,128–9,781,229,570)
Bulgaria	All causes	325,905,666,916<br>(299,289,957,105–355,646,042,693)	331,411,868,048<br>(305,280,020,419–360,771,447,211)	318,191,269,037<br>(292,853,915,912–346,892,159,381)	303,687,597,955<br>(278,969,934,996–331,667,902,787)	283,758,491,404<br>(259,579,345,848–310,910,600,582)	274,923,665,259<br>(250,141,343,909–302,920,623,264)
Bulgaria	Group I	25,188,606,912<br>(23,110,350,596–27,746,698,271)	20,588,211,332<br>(18,671,072,047–22,889,673,168)	17,690,002,703<br>(15,981,898,863–19,784,463,378)	14,555,681,255<br>(13,010,258,859–16,425,862,726)	12,956,795,117<br>(11,500,793,990–14,704,239,628)	10,943,937,590<br>(9,407,513,727–12,823,313,179)
Bulgaria	NCD	268,539,282,791<br>(246,262,723,919–293,428,181,198)	279,053,038,295<br>(257,538,094,126–303,789,010,672)	273,335,584,077<br>(252,316,712,412–297,576,345,375)	266,690,955,183<br>(245,324,181,486–290,381,090,744)	252,373,238,692<br>(231,255,883,610–276,131,878,583)	247,186,063,564<br>(224,864,066,280–271,590,304,325)
Bulgaria	Injuries	32,177,777,213<br>(29,420,620,042–35,255,288,851)	31,770,618,421<br>(29,171,265,979–34,734,136,666)	27,165,682,257<br>(24,761,957,309–29,802,624,308)	22,440,961,516<br>(20,367,087,467–24,885,335,999)	18,428,457,595<br>(16,687,315,675–20,611,711,505)	16,793,664,105<br>(15,089,267,806–18,925,162,020)
Croatia	All causes	161,164,700,053<br>(147,104,261,226–177,743,303,683)	160,990,325,744<br>(146,255,388,385–177,045,815,805)	150,373,917,164<br>(136,435,431,868–165,777,358,544)	142,535,156,359<br>(128,569,326,684–158,162,509,135)	138,111,748,511<br>(124,409,981,683–154,092,491,497)	132,932,374,545<br>(119,184,750,101–148,837,676,327)
Croatia	Group I	9,807,067,380<br>(8,748,657,350–11,181,329,990)	8,857,028,278<br>(7,823,332,955–10,123,337,493)	7,735,260,246<br>(6,784,578,727–8,929,146,302)	6,320,070,085<br>(5,435,028,250–7,460,628,689)	5,443,029,719<br>(4,613,759,810–6,526,830,684)	4,791,808,035<br>(3,983,456,112–5,807,835,525)
Croatia	NCD	131,609,097,274<br>(119,712,752,413–144,942,616,674)	130,543,343,122<br>(118,239,892,985–144,162,010,835)	128,935,644,129<br>(116,929,333,201–142,132,167,248)	124,341,610,683<br>(112,075,449,711–138,092,115,788)	122,305,312,739<br>(110,170,271,601–136,181,004,512)	118,854,895,209<br>(106,369,804,667–133,030,707,136)
Croatia	Injuries	19,748,535,399<br>(18,281,966,390–21,504,998,197)	21,589,954,345<br>(17,609,967,616–26,047,427,666)	13,703,012,789<br>(12,683,821,638–14,871,261,556)	11,873,475,592<br>(10,961,863,787–12,925,129,119)	10,363,406,054<br>(9,530,483,215–11,367,510,945)	9,285,671,301<br>(8,487,681,790–10,291,375,719)
Czech Republic	All causes	361,661,456,256<br>(331,766,496,657–396,368,811,247)	334,948,993,086<br>(304,818,021,591–369,982,695,914)	318,189,207,565<br>(287,474,523,023–353,298,408,948)	310,641,104,235<br>(278,823,801,037–346,515,038,770)	305,009,703,601<br>(272,266,837,875–342,372,792,745)	302,870,693,371<br>(268,128,524,015–341,099,735,905)
Czech Republic	Group I	18,990,849,825<br>(16,821,608,227–21,720,412,279)	15,666,419,980<br>(13,689,430,103–18,318,994,206)	14,156,754,253<br>(12,215,706,907–16,813,002,898)	14,123,550,371<br>(12,175,884,739–16,668,188,615)	14,036,506,635<br>(12,106,470,225–16,579,620,202)	13,243,904,935<br>(11,232,226,479–15,814,908,180)

Appendix Table 3. DALYs for all causes and level 1 cause groups by location for 1990 - 2015, both sexes combined.

	Cause	1990	1995	2000	2005	2010	2015
Czech Republic	NCD	304,387,073,859 (279,200,856,079–333,075,177,608)	283,681,236,876 (257,687,784,323–313,010,270,342)	272,576,344,179 (246,319,010,694–302,226,327,617)	268,155,558,190 (240,714,739,064–298,878,976,482)	265,943,104,653 (237,140,395,888–298,163,615,249)	267,387,093,904 (236,619,837,788–301,261,210,344)
Czech Republic	Injuries	38,283,532,572 (35,265,343,118–41,696,128,771)	35,601,336,230 (32,885,539,360–38,748,526,224)	31,456,109,133 (29,015,588,762–34,342,988,981)	28,361,995,673 (26,071,317,668–31,049,145,686)	25,030,092,313 (22,973,791,592–27,574,879,819)	22,239,694,532 (20,168,658,631–24,765,128,263)
Hungary	All causes	432,412,577,540 (400,252,227,631–467,513,146,583)	427,164,808,004 (395,737,428,749–463,309,540,763)	398,612,185,822 (366,425,646,578–434,785,000,876)	380,238,648,662 (347,599,298,870–416,504,117,954)	357,986,154,951 (325,695,069,309–393,989,991,956)	330,518,480,632 (297,728,235,022–366,940,523,961)
Hungary	Group I	23,685,421,257 (21,662,072,343–26,137,348,443)	18,812,166,965 (16,961,985,973–21,198,440,377)	15,577,173,889 (13,750,809,565–17,827,511,794)	13,000,106,967 (11,198,036,459–15,136,849,718)	11,450,874,697 (9,664,626,748–13,608,271,855)	10,504,278,346 (8,618,706,258–12,698,778,497)
Hungary	NCD	358,063,017,041 (330,641,871,026–388,070,423,705)	363,377,928,083 (336,497,703,017–394,551,484,803)	345,565,852,440 (317,415,331,264–376,771,025,355)	335,137,625,464 (306,162,613,117–367,373,913,144)	319,991,287,468 (290,604,181,329–352,124,259,732)	297,453,439,022 (267,865,873,525–330,328,853,301)
Hungary	Injuries	50,664,139,243 (47,528,786,428–53,964,438,396)	44,974,712,956 (42,015,239,838–48,194,001,461)	37,469,159,494 (34,921,236,036–40,286,687,041)	32,100,916,231 (29,870,310,939–34,559,841,908)	26,543,992,787 (24,656,880,811–28,782,084,693)	22,560,763,264 (20,550,158,521–24,907,137,718)
Macedonia	All causes	58,592,714,591 (53,349,653,595–64,521,616,112)	56,673,920,110 (51,733,866,154–62,361,073,139)	56,792,491,913 (51,490,718,080–62,769,509,937)	57,457,049,964 (51,784,077,150–63,853,066,345)	56,790,107,124 (51,017,321,593–63,273,180,052)	56,350,443,032 (49,953,080,903–63,232,344,692)
Macedonia	Group I	10,912,584,219 (10,071,690,990–11,770,751,200)	7,311,818,043 (6,756,453,161–7,963,285,380)	4,527,113,044 (4,074,413,559–5,032,609,221)	3,648,428,538 (3,239,713,696–4,147,325,895)	2,909,582,088 (2,539,688,740–3,365,864,829)	2,795,266,782 (2,229,402,037–3,465,805,310)
Macedonia	NCD	42,511,030,969 (38,061,484,389–47,390,035,288)	44,655,751,410 (40,231,147,709–49,542,430,517)	47,890,720,693 (43,298,482,253–53,049,503,731)	50,146,410,184 (45,117,427,243–55,808,497,403)	50,525,847,305 (45,298,248,195–56,358,065,427)	50,071,442,911 (44,254,089,544–56,382,804,549)
Macedonia	Injuries	5,169,099,403 (4,698,501,734–5,725,074,422)	4,706,350,657 (4,268,186,799–5,225,333,977)	4,374,658,175 (3,998,700,057–4,811,391,210)	3,662,211,241 (3,325,950,436–4,057,967,681)	3,354,677,731 (3,034,290,845–3,730,491,641)	3,483,733,339 (3,120,572,636–3,871,616,493)
Montenegro	All causes	16,395,650,646 (14,692,076,689–18,345,461,972)	18,111,834,850 (16,350,307,641–20,078,264,217)	19,037,070,263 (17,314,941,172–21,023,599,074)	18,079,846,446 (16,296,853,206–20,063,022,672)	17,103,486,744 (15,294,247,763–19,191,905,990)	17,258,282,143 (15,306,686,564–19,455,437,509)
Montenegro	Group I	1,955,520,447 (1,552,563,170–2,415,473,264)	1,654,797,050 (1,458,282,836–1,896,548,592)	1,767,079,607 (1,571,812,538–1,975,270,902)	1,143,690,775 (993,336,917–1,333,548,761)	900,984,740 (763,007,110–1,078,114,184)	777,129,373 (631,726,346–956,813,203)
Montenegro	NCD	12,506,053,570 (11,152,932,191–14,039,375,282)	14,384,756,822 (12,880,969,658–15,975,697,041)	15,344,377,587 (13,901,226,472–17,023,231,567)	15,256,279,985 (13,712,218,237–16,967,252,281)	14,659,049,661 (13,074,491,407–16,451,130,888)	15,060,286,047 (13,321,341,648–16,991,614,163)
Montenegro	Injuries	1,934,076,630 (1,747,418,366–2,150,923,129)	2,072,280,978 (1,915,911,325–2,266,567,828)	1,925,613,069 (1,778,161,768–2,104,714,426)	1,679,875,685 (1,540,205,255–1,844,601,126)	1,543,452,343 (1,404,744,900–1,695,961,214)	1,420,866,723 (1,266,481,270–1,589,606,703)
Poland	All causes	1,288,706,572,129 (1,182,728,185,246–1,409,798,029,978)	1,254,875,419,839 (1,145,857,594,936–1,376,803,785,000)	1,184,890,529,486 (1,074,833,998,064–1,310,000,131,557)	1,164,410,940,591 (1,052,650,374,824–1,293,575,802,968)	1,151,131,822,669 (1,036,717,596,982–1,281,872,482,710)	1,136,008,115,564 (1,016,116,222,833–1,272,561,702,479)
Poland	Group I	104,416,683,823 (96,254,799,786–114,873,004,759)	81,652,344,595 (74,296,194,812–91,049,790,803)	58,478,846,311 (52,123,937,718–66,424,283,105)	53,311,373,996 (47,602,031,325–61,006,087,957)	51,300,838,171 (45,447,217,607–58,868,745,277)	45,685,120,006 (39,832,225,771–53,031,181,253)
Poland	NCD	1,032,914,711,414 (943,864,835,834–1,135,273,808,363)	1,027,522,514,384 (934,040,202,353–1,132,237,299,322)	991,944,314,565 (896,402,536,084–1,099,642,677,391)	985,096,239,053 (885,867,333,895–1,099,071,179,211)	984,718,774,804 (881,550,542,388–1,100,661,043,201)	984,689,983,018 (877,800,736,544–1,108,343,346,686)
Poland	Injuries	151,375,176,892 (141,226,787,761–162,370,050,472)	145,700,560,860 (136,156,560,556–156,460,925,862)	134,467,368,610 (125,512,020,297–144,742,054,870)	126,003,327,542 (117,596,552,978–135,426,541,020)	115,112,209,694 (107,514,614,316–123,962,472,717)	105,633,012,540 (97,675,698,479–115,361,948,966)
Romania	All causes	848,220,328,223 (780,448,304,415–924,588,167,204)	883,197,811,801 (815,624,737,597–959,106,920,573)	812,802,728,714 (746,138,242,728–888,072,437,419)	770,121,956,076 (705,349,006,033–843,666,541,758)	725,449,605,033 (661,627,894,713–796,194,536,572)	674,979,185,606 (611,822,104,687–747,004,468,462)
Romania	Group I	117,641,923,034 (104,839,019,349–126,455,993,583)	90,789,142,984 (83,595,484,966–98,564,828,943)	79,772,162,867 (72,882,040,883–86,796,897,875)	62,773,620,006 (57,337,128,558–69,077,214,932)	46,238,321,877 (41,546,705,191–52,158,365,295)	37,069,694,028 (32,260,275,945–43,003,350,278)
Romania	NCD	631,635,850,597 (575,957,423,485–692,036,423,047)	695,116,442,474 (638,669,228,032–757,090,933,230)	654,856,838,337 (599,144,414,555–716,482,007,217)	639,594,849,584 (584,020,096,225–702,173,780,146)	619,525,608,260 (564,655,548,677–680,325,627,498)	587,332,235,855 (531,531,840,160–649,794,877,186)
Romania	Injuries	98,942,554,592 (91,520,520,006–106,989,850,229)	97,292,226,342 (90,665,708,210–105,093,828,801)	78,173,727,510 (72,240,522,509–84,978,406,260)	67,753,486,487 (62,589,426,735–73,689,457,813)	59,685,674,896 (55,105,003,185–65,079,329,604)	50,577,255,723 (46,163,775,485–56,251,367,670)
Serbia	All causes	295,466,898,059 (267,778,834,975–326,389,729,746)	326,861,351,996 (298,631,825,366–357,614,625,710)	334,684,743,432 (306,222,831,105–366,321,421,096)	318,155,077,296 (290,097,121,874–349,451,545,873)	300,144,464,225 (271,989,304,403–331,947,501,744)	281,901,265,998 (253,809,650,449–313,654,028,375)
Serbia	Group I	31,064,162,641 (26,718,287,858–35,871,517,974)	25,482,518,337 (23,079,778,284–28,380,933,721)	18,196,316,008 (16,130,889,858–20,616,332,637)	14,314,305,439 (12,511,767,741–16,592,656,787)	12,540,460,843 (10,931,564,256–14,537,645,839)	11,382,696,055 (9,848,567,315–13,345,569,657)
Serbia	NCD	233,550,321,712 (210,149,103,400–258,874,922,390)	271,790,714,751 (246,910,556,447–298,249,671,187)	286,551,479,633 (261,779,741,912–313,876,454,799)	280,345,339,829 (255,116,918,669–307,776,905,734)	267,095,190,984 (241,592,562,412–295,394,779,186)	250,635,413,023 (224,968,059,847–278,380,722,482)
Serbia	Injuries	30,852,413,706 (27,816,222,384–33,747,007,557)	29,588,118,908 (27,212,360,667–32,030,886,586)	29,936,947,791 (27,079,375,001–32,964,449,542)	23,495,432,028 (21,704,870,904–25,478,336,905)	20,508,812,397 (18,913,668,593–22,524,609,489)	19,883,156,920 (18,247,569,052–21,942,767,812)
Slovakia	All causes	174,375,755,399 (159,818,473,066–190,823,358,668)	165,622,395,896 (150,186,667,075–182,803,242,712)	165,371,448,067 (150,139,628,945–182,650,907,861)	164,011,554,708 (148,144,783,467–181,936,518,048)	160,016,158,401 (143,848,857,023–178,521,029,560)	153,489,496,367 (136,412,554,562–172,511,643,710)
Slovakia	Group I	15,003,102,215 (13,395,220,072–16,800,098,459)	12,355,917,402 (10,903,348,901–13,962,750,529)	10,119,322,885 (8,911,755,941–11,574,129,121)	9,374,387,866 (8,193,238,412–10,837,356,553)	8,679,044,249 (7,579,450,721–10,102,493,083)	7,925,552,387 (6,775,729,460–9,367,770,910)
Slovakia	NCD	138,569,939,086 (126,412,646,348–151,785,991,967)	134,896,771,237 (121,793,554,889–149,083,766,928)	138,251,433,645 (125,095,235,153–152,992,462,625)	139,308,927,071 (125,385,439,455–154,807,684,563)	137,341,445,691 (123,124,848,190–153,272,890,073)	132,891,730,576 (117,763,362,812–149,190,814,102)
Slovakia	Injuries	20,802,714,097 (19,126,985,093–22,463,893,412)	18,369,707,257 (16,926,747,818–19,966,731,275)	17,000,691,537 (15,707,339,747–18,439,193,863)	15,328,239,771 (14,144,537,643–16,742,728,413)	13,995,668,460 (12,836,044,649–15,398,481,699)	12,672,213,404 (11,440,994,908–14,189,490,933)
Slovenia	All causes	60,839,867,002 (54,997,103,953–67,407,959,046)	60,134,326,416 (54,107,969,071–66,929,878,077)	58,963,463,700 (52,870,632,576–65,829,344,305)	57,167,450,473 (50,916,283,865–64,209,199,745)	54,802,574,050 (48,324,868,388–62,125,340,822)	53,449,255,114 (46,738,689,172–61,042,579,328)
Slovenia	Group I	3,531,574,627 (3,062,284,835–4,116,308,780)	3,073,354,004 (2,635,175,095–3,647,057,738)	2,855,471,109 (2,455,465,327–3,351,584,182)	2,671,588,042 (2,276,768,770–3,175,339,846)	2,516,970,997 (2,124,950,517–3,043,974,636)	2,433,742,637 (2,041,105,401–2,944,044,223)
Slovenia	NCD	48,293,130,732 (43,395,525,568–53,628,516,451)	48,785,525,398 (43,664,279,692–54,377,599,937)	48,855,525,121 (43,538,417,209–54,690,433,058)	48,181,319,325 (42,782,703,413–54,268,957,152)	47,132,486,813 (41,397,004,715–53,531,571,139)	46,596,242,178 (40,600,847,512–53,298,446,089)
Slovenia	Injuries	9,015,161,644 (8,377,544,509–9,701,561,739)	8,275,447,014 (7,714,454,475–8,898,725,752)	7,252,467,471 (6,740,985,359–7,822,929,414)	6,314,543,106 (5,870,894,657–6,821,772,090)	5,153,116,240 (4,734,054,994–5,651,568,217)	4,419,270,299 (4,004,809,476–4,957,533,174)
Central Asia	All causes	2,766,779,256,884 (2,591,292,243,606–2,967,709,928,568)	2,953,387,085,688 (2,765,847,564,176–3,158,006,023,020)	2,708,426,775,463 (2,516,711,656,919–2,909,005,328,748)	2,695,481,838,930 (2,499,503,288,671–2,913,700,991,687)	2,632,859,969,176 (2,427,443,664,922–2,865,106,294,649)	2,617,199,654,215 (2,389,811,089,672–2,864,840,299,047)
Central Asia	Group I	1,065,136,712,248 (1,015,751,547,844–1,123,631,120,778)	1,008,958,717,583 (960,191,751,843–1,067,543,699,099)	780,352,648,023 (735,722,571,572–829,410,774,889)	670,817,449,684 (621,083,339,608–724,327,456,625)	606,516,040,449 (559,136,190,375–657,661,346,609)	522,251,659,433 (472,898,151,877–572,749,312,286)
Central Asia	NCD	1,362,658,729,016 (1,234,291,825,710–1,505,873,327,486)	1,548,057,569,590 (1,411,249,288,902–1,694,725,610,678)	1,591,114,639,784 (1,449,308,286,798–1,747,784,079,324)	1,699,645,168,920 (1,548,155,695,955–1,870,722,725,346)	1,719,572,227,102 (1,556,768,685,902–1,903,319,695,027)	1,794,879,773,268 (1,619,326,597,740–1,995,919,837,590)
Central Asia	Injuries	338,983,815,621 (321,705,290,585–356,983,180,952)	396,370,798,515 (369,227,559,899–424,172,040,344)	336,959,487,656 (320,093,641,662–354,127,005,217)	325,019,220,327 (309,319,633,639–342,645,490,638)	306,771,701,626 (290,373,762,063–326,290,370,842)	300,068,221,514 (279,787,712,388–321,483,955,678)
Armenia	All causes	118,565,351,232 (109,197,802,980–129,204,951,748)	113,204,305,436 (104,387,721,079–123,039,468,222)	98,150,564,533 (89,438,644,060–107,886,754,875)	97,362,123,395 (88,882,117,718–106,745,480,337)	91,342,709,925 (83,073,179,434–100,948,361,657)	90,630,488,892 (81,732,140,914–100,464,593,810)
Armenia	Group I	29,846,980,870 (27,362,514,076–32,482,577,625)	22,069,699,927 (20,032,144,017–24,351,721,757)	13,216,465,233 (11,745,317,121–14,883,916,126)	11,097,171,590 (9,713,971,892–12,595,400,787)	8,949,305,383 (7,485,918,529–10,347,905,275)	7,171,973,125 (5,907,770,277–8,571,706,223)
Armenia	NCD	70,100,321,133 (63,028,688,396–77,707,339,499)	75,502,964,183 (68,368,113,630–83,013,096,685)	73,980,440,085 (66,769,865,075–81,695,237,386)	76,219,131,273 (69,302,425,044–83,792,943,162)	72,877,580,812 (65,945,283,340–80,678,156,772)	75,105,434,749 (67,761,404,829–83,521,726,115)
Armenia	Injuries	18,618,049,229 (16,713,758,519–20,451,499,362)	15,631,641,326 (14,212,603,428–17,098,930,567)	10,953,659,215 (9,829,323,878–12,200,197,595)	10,045,820,532 (9,001,688,517–11,107,673,020)	9,515,823,729 (8,566,751,434–10,464,155,693)	8,353,081,017 (7,411,619,559–9,369,804,211)
Azerbaijan	All causes	313,481,526,551 (291,061,071,936–338,814,567,405)	331,387,511,649 (306,213,172,823–356,430,143,194)	291,727,873,913 (268,963,980,309–316,965,183,481)	291,402,988,555 (265,879,209,615–318,664,028,344)	290,832,761,062 (260,986,575,008–322,214,488,814)	285,026,877,362 (254,270,533,264–318,180,376,347)

Appendix Table 3. DALYs for all causes and level 1 cause groups by location for 1990 - 2015, both sexes combined.

	Cause	1990	1995	2000	2005	2010	2015
Azerbaijan	Group I	136,696,023,426 (124,211,344,670–150,419,613,201)	130,626,568,905 (118,657,526,466–143,976,378,285)	94,404,456,238 (86,159,369,959–103,540,368,299)	82,267,243,152 (74,050,145,363–91,089,827,497)	71,917,189,130 (60,075,759,951–85,188,944,201)	56,653,552,875 (46,892,809,629–67,260,361,647)
Azerbaijan	NCD	143,749,839,023 (129,302,663,050–159,479,100,722)	162,500,489,655 (147,477,515,234–179,377,915,437)	170,213,031,410 (153,317,700,590–188,872,671,359)	184,229,884,548 (165,318,677,904–205,368,449,421)	195,792,347,208 (175,519,089,475–219,074,621,504)	204,546,712,435 (181,707,348,960–229,961,193,908)
Azerbaijan	Injuries	33,035,664,102 (30,307,720,296–35,790,838,474)	38,260,453,089 (35,080,597,212–41,399,363,170)	27,110,386,266 (24,701,499,070–29,803,972,154)	24,905,860,855 (22,617,321,854–27,358,237,938)	23,123,224,724 (20,629,039,190–25,765,680,885)	23,826,612,051 (21,063,589,163–27,030,456,968)
Georgia	All causes	196,689,040,221 (180,309,966,574–214,240,853,036)	174,697,718,253 (159,689,838,866–191,614,794,935)	161,621,616,148 (147,237,027,230–177,324,345,392)	158,287,967,900 (144,171,373,812–173,880,779,636)	153,082,253,231 (139,512,805,952–168,096,806,420)	142,969,941,944 (129,652,484,682–157,202,689,586)
Georgia	Group I	38,857,286,673 (35,570,800,964–42,277,850,210)	31,817,886,458 (29,166,764,669–34,639,803,242)	24,031,049,108 (21,603,093,158–26,749,855,966)	18,633,144,302 (16,412,473,902–21,058,087,977)	15,513,454,587 (13,473,697,806–17,840,805,014)	12,335,486,357 (10,593,231,221–14,397,656,144)
Georgia	NCD	136,106,280,312 (122,839,917,279–150,197,616,292)	125,722,142,329 (113,150,697,893–139,932,019,909)	124,529,459,788 (112,915,909,015–137,585,513,651)	127,118,753,708 (115,516,945,648–140,149,317,504)	124,421,509,003 (112,952,658,320–137,246,848,043)	117,515,519,190 (106,387,888,027–129,755,397,840)
Georgia	Injuries	21,725,473,236 (19,882,938,804–23,725,643,298)	17,157,689,466 (15,520,835,772–18,794,667,912)	13,061,107,252 (11,708,288,443–14,685,189,481)	12,536,069,889 (11,380,331,415–13,867,614,078)	13,147,289,642 (12,074,938,543–14,392,919,277)	13,118,936,397 (11,945,984,768–14,450,112,803)
Kazakhstan	All causes	609,614,419,332 (564,143,595,118–663,431,596,320)	720,605,339,713 (670,564,583,397–775,246,610,862)	628,551,550,520 (585,856,544,806–677,781,603,859)	657,214,189,012 (611,164,169,989–707,079,876,003)	593,183,801,241 (545,482,194,569–645,846,021,863)	592,329,263,534 (533,007,493,453–652,313,105,608)
Kazakhstan	Group I	149,401,165,485 (136,218,548,237–164,513,056,454)	141,435,894,900 (128,312,257,890–154,952,939,471)	108,419,822,405 (98,471,448,725–120,487,597,915)	103,145,696,975 (92,127,610,710–116,101,638,659)	92,336,017,851 (80,796,252,829–105,184,757,046)	81,430,939,397 (68,861,647,479–95,067,147,109)
Kazakhstan	NCD	363,275,249,134 (330,788,281,182–400,452,985,955)	436,400,222,176 (402,779,980,844–473,467,124,395)	410,998,616,486 (379,604,958,773–447,102,754,433)	441,515,844,399 (408,304,546,370–478,068,169,776)	409,444,667,163 (374,775,684,037–448,545,440,192)	422,165,597,253 (377,209,549,545–469,892,861,897)
Kazakhstan	Injuries	96,938,004,713 (92,298,450,459–101,811,382,055)	142,769,222,637 (123,661,234,045–161,897,129,523)	109,133,111,630 (104,306,660,347–113,852,545,224)	112,552,647,638 (108,144,147,859–117,372,349,534)	91,403,116,226 (87,304,687,446–96,294,381,354)	88,732,726,885 (81,825,673,707–96,234,346,555)
Kyrgyzstan	All causes	191,691,210,401 (180,070,399,875–204,965,309,341)	187,982,059,337 (175,717,387,180–200,734,636,860)	183,462,186,830 (170,386,719,953–197,269,835,484)	180,498,146,867 (166,501,243,441–195,017,311,415)	184,306,466,046 (170,376,070,544–200,459,230,064)	181,996,891,956 (166,563,140,255–199,948,238,690)
Kyrgyzstan	Group I	77,795,247,322 (73,968,753,981–82,325,886,285)	65,437,917,388 (61,987,946,356–69,167,959,519)	54,658,950,557 (51,220,630,421–58,121,601,984)	47,585,371,704 (44,269,265,444–50,982,022,206)	48,427,182,973 (44,733,210,538–52,384,458,539)	44,457,292,925 (40,040,116,522–49,610,150,843)
Kyrgyzstan	NCD	88,604,376,801 (80,255,373,837–97,170,020,786)	97,949,847,224 (88,598,982,663–107,065,267,539)	106,045,271,484 (96,267,322,022–116,076,419,099)	112,227,521,690 (101,832,087,091–123,457,892,815)	112,882,736,463 (101,967,528,244–124,872,727,148)	117,701,078,317 (105,547,105,465–131,212,056,126)
Kyrgyzstan	Injuries	25,291,586,278 (23,287,323,066–27,042,083,694)	24,594,294,725 (22,686,803,962–26,188,191,003)	22,757,964,789 (21,253,343,285–24,228,993,506)	20,685,253,473 (19,364,214,283–22,186,900,630)	22,996,546,609 (21,750,902,472–24,500,964,707)	19,838,520,714 (18,338,308,905–21,631,287,905)
Mongolia	All causes	111,337,553,428 (104,970,357,329–118,043,958,870)	100,811,524,745 (94,639,609,147–107,338,542,101)	93,282,352,783 (86,928,803,643–100,187,883,368)	95,197,729,824 (88,019,507,116–102,722,184,426)	100,873,338,339 (93,105,749,458–109,138,063,656)	102,783,784,623 (94,197,454,175–112,415,201,001)
Mongolia	Group I	51,765,685,198 (47,574,324,126–56,011,080,954)	37,574,149,037 (34,482,297,724–40,654,229,239)	26,682,067,132 (24,502,894,163–28,964,793,663)	21,864,467,011 (19,803,202,455–24,264,906,715)	20,803,238,946 (18,058,994,578–23,765,992,861)	17,456,168,255 (14,782,441,098–20,854,790,675)
Mongolia	NCD	47,084,859,289 (42,260,995,535–51,927,175,815)	50,625,043,501 (46,083,058,775–55,177,988,275)	53,243,330,871 (48,474,496,205–58,418,521,072)	57,644,880,654 (52,266,461,435–63,238,125,623)	62,879,861,910 (57,040,619,429–68,867,736,090)	68,057,506,683 (61,271,450,567–74,964,094,545)
Mongolia	Injuries	12,487,008,941 (11,163,458,971–14,030,390,094)	12,612,332,207 (11,469,451,407–13,865,452,365)	13,356,954,780 (12,088,909,769–14,442,573,412)	15,688,382,159 (13,729,170,628–17,045,388,068)	17,190,237,484 (14,783,624,905–18,662,923,302)	17,270,109,684 (14,902,790,700–18,952,312,652)
Tajikistan	All causes	279,085,084,081 (260,434,873,574–299,253,059,991)	295,708,745,357 (274,923,941,642–318,729,244,281)	255,400,574,999 (236,267,279,984–273,894,422,738)	228,770,447,141 (211,496,261,852–248,684,475,696)	228,329,428,327 (208,485,286,717–250,262,626,832)	230,106,525,024 (203,677,683,002–257,217,077,567)
Tajikistan	Group I	162,797,427,006 (149,489,680,955–177,076,585,430)	163,551,564,644 (149,920,085,798–178,158,797,554)	126,995,474,209 (116,954,408,475–137,847,701,494)	96,653,528,438 (88,045,510,615–106,190,485,974)	88,397,377,418 (79,301,235,416–98,087,694,824)	81,202,644,516 (68,563,593,851–95,530,603,164)
Tajikistan	NCD	92,101,651,871 (82,151,809,163–103,751,311,068)	101,561,680,248 (91,142,712,959–112,870,053,211)	104,020,441,244 (93,547,626,209–115,796,302,865)	110,892,984,842 (98,831,135,505–124,960,689,039)	118,326,417,864 (105,095,602,680–133,767,323,587)	127,205,252,623 (111,444,640,609–144,533,754,476)
Tajikistan	Injuries	24,186,005,204 (21,851,473,483–26,840,950,067)	30,595,500,464 (27,665,198,445–33,510,269,993)	24,384,659,545 (22,483,949,769–26,672,238,358)	21,223,933,861 (19,520,182,852–23,127,721,550)	21,605,633,045 (19,645,218,548–23,759,404,699)	21,698,627,884 (19,563,136,049–24,315,607,379)
Turkmenistan	All causes	188,568,706,315 (172,069,914,703–206,332,359,242)	195,711,292,705 (178,243,585,553–215,047,861,383)	191,455,772,341 (173,054,347,062–212,415,353,388)	179,379,632,817 (161,068,677,701–199,816,812,372)	170,048,959,162 (152,241,122,490–189,575,785,671)	165,598,600,613 (148,490,912,328–183,992,855,230)
Turkmenistan	Group I	99,798,472,106 (87,654,448,291–112,559,118,463)	92,531,193,479 (80,297,303,398–105,911,005,003)	77,757,407,957 (64,922,501,471–93,096,015,456)	62,786,838,303 (50,327,243,419–76,503,695,105)	50,296,897,531 (40,235,470,129–61,985,904,202)	41,400,489,872 (33,354,399,474–51,410,249,579)
Turkmenistan	NCD	69,517,197,591 (63,107,863,034–76,399,278,444)	82,534,068,999 (75,109,954,978–90,271,651,817)	92,372,850,063 (84,205,086,617–101,066,320,745)	97,019,536,698 (88,086,790,816–107,441,940,552)	102,450,600,540 (92,312,157,134–114,194,782,252)	108,078,985,496 (96,470,494,574–120,906,103,480)
Turkmenistan	Injuries	19,253,036,618 (17,299,848,895–21,415,997,806)	20,646,030,227 (18,661,295,780–23,007,588,808)	21,325,514,320 (19,143,170,454–23,567,017,499)	19,573,257,815 (17,521,270,424–21,641,193,959)	17,301,461,090 (15,736,483,334–19,347,399,880)	16,119,125,245 (14,555,556,432–18,467,832,811)
Uzbekistan	All causes	757,746,365,324 (695,250,714,640–822,947,060,416)	833,278,588,495 (769,866,074,040–908,225,920,601)	804,774,283,396 (734,514,920,629–875,341,835,048)	807,368,613,420 (733,361,642,301–891,827,528,554)	820,860,251,845 (736,745,131,027–907,164,129,773)	825,757,280,268 (742,436,121,137–924,766,475,827)
Uzbekistan	Group I	318,178,424,161 (287,433,871,542–351,322,219,867)	323,913,842,845 (296,514,325,168–357,368,068,888)	254,186,955,183 (228,953,682,914–283,851,957,351)	226,783,988,208 (196,929,099,802–264,513,600,776)	209,875,376,630 (178,285,822,913–247,721,862,249)	180,143,112,110 (150,795,481,591–214,387,335,934)
Uzbekistan	NCD	352,118,953,862 (316,368,340,471–393,980,668,420)	415,261,111,275 (374,111,354,478–460,120,186,219)	455,711,198,354 (410,494,885,044–504,868,538,754)	492,776,631,108 (442,846,917,071–548,141,007,453)	520,496,506,138 (466,086,716,871–582,133,378,104)	554,503,686,520 (493,710,662,132–625,277,435,581)
Uzbekistan	Injuries	87,448,987,301 (79,937,342,427–95,129,502,797)	94,103,634,375 (86,856,757,409–101,955,557,688)	94,876,129,859 (87,332,030,599–102,157,850,244)	87,807,994,104 (80,815,515,305–95,416,709,057)	90,488,369,077 (82,344,753,475–99,806,060,446)	91,110,481,637 (81,714,178,058–101,883,128,237)
Latin America and Caribbean	All causes	13,775,950,512,726 (12,818,035,623,300–14,885,523,261,662)	13,665,968,913,044 (12,594,535,178,267–14,860,559,596,150)	13,506,763,032,913 (12,315,330,617,665–14,829,722,374,097)	13,583,212,392,528 (12,292,042,192,929–15,023,920,279,201)	15,482,124,703,829 (13,715,316,566,281–17,407,247,887,688)	14,652,968,447,997 (13,118,151,861,380–16,311,588,360,302)
Latin America and Caribbean	Group I	5,098,956,574,042 (4,902,501,630,754–5,336,487,426,443)	4,271,846,947,125 (4,098,376,698,539–4,490,266,820,969)	3,563,928,234,924 (3,379,630,956,905–3,788,046,279,484)	3,054,638,138,066 (2,865,309,471,284–3,289,192,827,352)	2,659,045,025,514 (2,458,701,054,003–2,903,959,902,594)	2,364,643,645,415 (2,157,896,437,753–2,622,210,492,649)
Latin America and Caribbean	NCD	6,630,972,690,416 (5,829,534,344,216–7,460,872,148,307)	7,284,181,204,607 (6,409,558,461,458–8,222,955,194,420)	7,864,816,588,789 (6,891,165,705,173–8,938,406,021,576)	8,492,177,101,280 (7,416,667,786,654–9,657,840,311,133)	9,285,049,434,705 (8,088,522,182,271–10,575,867,436,378)	10,227,394,542,368 (8,929,554,527,910–11,646,891,266,070)
Latin America and Caribbean	Injuries	2,046,021,248,268 (1,974,192,533,934–2,115,459,575,742)	2,109,940,761,312 (2,048,206,131,362–2,167,656,237,030)	2,078,018,209,200 (2,021,641,196,105–2,141,317,762,548)	2,036,397,153,182 (1,974,805,631,816–2,098,437,645,472)	3,538,030,243,611 (2,372,202,529,851–4,788,686,503,929)	2,060,930,260,215 (1,985,259,651,982–2,141,328,560,342)
Central Latin America	All causes	5,253,304,683,317 (4,898,501,663,091–5,681,126,004,734)	5,260,135,613,963 (4,842,981,377,220–5,726,158,359,874)	5,231,217,583,861 (4,763,451,436,971–5,752,949,048,674)	5,321,334,174,516 (4,819,056,837,707–5,900,847,277,108)	5,653,276,646,161 (5,092,224,347,153–6,282,714,929,979)	5,937,928,713,117 (5,316,029,110,437–6,640,583,680,738)
Central Latin America	Group I	1,858,673,571,851 (1,792,288,900,589–1,943,122,092,975)	1,557,054,627,536 (1,486,070,986,832–1,642,980,435,723)	1,308,465,501,045 (1,231,763,534,191–1,401,652,476,800)	1,134,680,393,602 (1,057,415,050,568–1,232,401,364,278)	1,001,553,665,191 (921,423,953,929–1,106,325,324,378)	893,907,109,216 (810,970,470,612–998,320,588,833)
Central Latin America	NCD	2,548,182,231,741 (2,239,637,209,042–2,885,308,008,118)	2,836,377,321,591 (2,491,494,069,022–3,220,414,895,662)	3,063,855,682,599 (2,678,068,994,971–3,499,620,699,992)	3,355,992,629,838 (2,929,574,898,625–3,842,235,690,757)	3,748,099,177,496 (3,269,611,694,053–4,289,261,007,299)	4,174,899,090,553 (3,638,549,329,458–4,776,923,548,582)
Central Latin America	Injuries	846,448,879,725 (828,352,448,656–866,125,929,842)	866,703,664,837 (851,690,534,803–883,539,500,481)	858,896,400,218 (841,707,129,177–876,724,691,431)	830,661,151,076 (813,736,510,126–849,727,549,555)	903,623,803,475 (885,236,461,936–925,018,446,757)	869,122,513,348 (838,556,271,175–900,612,635,550)
Colombia	All causes	1,001,587,367,843 (924,892,654,785–1,092,803,923,385)	1,054,850,595,424 (966,683,549,645–1,151,800,295,640)	1,061,302,504,920 (965,285,046,303–1,168,966,119,801)	1,034,719,944,260 (929,990,798,478–1,157,046,162,696)	1,054,999,291,267 (938,042,574,474–1,183,764,160,253)	1,056,466,042,480 (927,246,068,397–1,195,199,225,084)
Colombia	Group I	251,630,322,500 (234,614,129,165–270,638,370,943)	226,035,748,042 (208,318,884,179–246,036,691,212)	192,722,149,291 (176,585,637,791–212,151,864,453)	176,579,995,531 (160,098,248,563–197,347,412,629)	158,069,763,276 (137,733,497,339–181,943,003,795)	133,376,888,554 (112,346,192,479–158,754,733,295)
Colombia	NCD	501,405,391,082 (437,562,680,628–572,443,702,967)	553,002,754,492 (482,076,984,959–634,436,187,031)	595,176,835,526 (515,473,220,208–686,167,871,269)	643,635,476,004 (554,802,832,329–747,405,015,745)	700,445,386,847 (600,171,247,780–812,345,588,893)	748,463,052,084 (640,286,219,025–870,908,494,113)
Colombia	Injuries	248,551,654,261 (242,345,339,156–254,528,424,295)	275,812,092,890 (269,833,347,113–282,067,711,708)	273,403,520,104 (266,519,709,890–280,114,665,162)	214,504,472,725 (209,462,240,233–220,127,544,423)	196,484,141,144 (191,028,958,761–202,053,260,726)	174,626,101,842 (165,624,730,774–183,616,833,156)

Appendix Table 3. DALYs for all causes and level 1 cause groups by location for 1990 - 2015, both sexes combined.

	Cause	1990	1995	2000	2005	2010	2015
Costa Rica	All causes	64,907,330,117 (57,729,487,828–73,166,889,460)	72,903,788,308 (65,055,302,836–82,312,117,339)	78,627,363,437 (69,206,801,475–89,658,245,456)	83,198,141,249 (72,718,242,582–95,193,375,690)	89,374,679,967 (77,577,651,937–102,467,022,303)	96,378,301,062 (83,492,613,041–111,085,247,545)
Costa Rica	Group I	14,235,008,189 (12,462,663,273–16,441,239,027)	13,684,980,219 (11,825,826,872–15,933,097,748)	11,957,057,351 (10,264,156,370–14,231,069,165)	10,662,223,960 (9,102,304,450–12,605,945,278)	9,835,220,599 (8,249,010,261–11,812,138,327)	9,199,666,925 (7,601,398,198–11,326,866,153)
Costa Rica	NCD	43,174,955,024 (37,368,728,656–49,976,863,007)	50,436,736,701 (43,925,449,798–58,312,646,234)	56,958,597,476 (49,167,298,495–66,036,325,990)	62,668,678,283 (53,873,022,965–72,923,044,288)	69,110,022,097 (59,186,843,721–80,191,642,131)	76,950,071,179 (65,555,054,272–89,923,643,182)
Costa Rica	Injuries	7,497,366,903 (7,173,688,607–7,860,854,609)	8,782,071,388 (8,446,912,592–9,177,374,352)	9,711,708,610 (9,321,296,272–10,112,879,282)	9,867,239,007 (9,470,274,058–10,269,449,490)	10,429,437,272 (10,015,303,303–10,847,502,610)	10,228,562,958 (9,576,971,437–10,880,942,540)
El Salvador	All causes	213,036,171,173 (197,097,081,462–230,397,239,324)	191,969,046,752 (176,154,059,515–208,774,877,238)	176,629,388,317 (161,018,816,625–192,884,541,677)	168,912,854,251 (153,390,103,555–187,533,411,516)	165,942,352,217 (149,170,275,800–185,418,458,452)	166,518,148,601 (147,788,046,271–186,638,260,003)
El Salvador	Group I	71,118,143,820 (64,799,103,853–77,979,804,178)	56,332,593,562 (50,508,083,960–62,337,079,795)	43,248,198,774 (38,560,046,744–48,124,443,444)	30,786,653,240 (27,038,357,472–35,538,607,772)	24,648,151,343 (20,837,087,615–29,492,651,365)	22,252,292,357 (18,724,594,826–26,660,864,658)
El Salvador	NCD	96,440,951,930 (85,488,348,137–108,340,764,779)	95,639,428,597 (84,360,339,628–107,718,919,106)	95,144,660,514 (83,473,387,439–108,101,750,921)	99,027,774,726 (86,958,536,884–112,973,807,059)	103,648,412,414 (90,586,724,064–118,601,790,738)	108,210,930,975 (93,809,475,499–124,546,786,130)
El Salvador	Injuries	45,477,075,422 (41,337,793,825–49,414,996,119)	39,997,024,593 (36,934,228,072–42,128,703,168)	38,236,529,029 (36,137,581,736–40,383,061,154)	39,098,426,284 (36,635,629,868–41,067,128,207)	37,645,788,459 (34,811,934,217–39,942,923,679)	36,054,925,268 (32,476,720,066–39,652,772,713)
Guatemala	All causes	471,121,534,265 (446,485,859,621–500,154,394,307)	450,211,406,809 (423,183,756,206–481,522,888,000)	444,468,928,445 (413,357,309,878–479,356,289,517)	453,418,226,329 (416,506,253,532–494,275,408,663)	450,332,588,538 (407,612,670,369–497,030,901,751)	456,680,527,364 (403,602,167,127–513,443,543,736)
Guatemala	Group I	271,888,439,962 (258,302,047,686–286,330,965,258)	230,517,735,816 (219,305,511,918–243,427,500,193)	213,995,489,871 (201,038,783,424–227,908,121,310)	187,171,130,124 (171,647,330,580–202,622,844,547)	155,221,598,691 (138,603,230,788–173,654,127,017)	132,153,799,552 (114,444,854,580–151,505,440,788)
Guatemala	NCD	151,736,591,781 (134,799,321,464–169,510,766,225)	168,560,823,776 (149,007,946,640–188,524,569,439)	174,917,729,758 (153,527,503,862–198,067,960,918)	195,789,601,923 (170,996,408,649–223,034,615,322)	221,988,185,234 (193,916,909,659–253,446,619,787)	248,522,852,350 (213,103,932,960–286,737,752,916)
Guatemala	Injuries	47,496,502,523 (44,697,816,362–50,370,629,349)	51,132,847,216 (49,173,005,890–53,011,861,573)	55,555,708,816 (53,790,062,353–57,541,501,986)	70,457,494,282 (68,186,495,144–73,029,629,194)	73,122,804,613 (70,915,803,708–75,502,264,192)	76,003,875,462 (67,183,612,334–85,242,327,893)
Honduras	All causes	191,822,611,788 (179,213,017,592–205,631,859,487)	187,585,833,407 (172,154,319,612–204,992,323,325)	188,739,271,172 (170,197,248,636–210,156,603,785)	189,661,553,332 (168,143,541,238–214,263,245,496)	194,872,388,340 (171,472,290,278–222,454,594,206)	201,293,991,535 (174,955,957,190–231,076,257,007)
Honduras	Group I	77,292,571,172 (71,392,485,959–83,123,820,007)	66,268,052,802 (61,036,710,591–72,097,002,141)	58,250,228,701 (53,142,094,089–63,947,133,378)	50,223,800,029 (45,556,608,358–55,553,572,830)	43,691,495,418 (38,977,356,110–48,640,906,151)	38,230,943,263 (33,488,430,604–43,378,640,152)
Honduras	NCD	95,680,215,159 (85,622,284,388–106,346,631,095)	101,249,604,686 (89,869,985,017–113,496,716,556)	106,212,592,682 (92,928,155,480–120,855,933,698)	112,327,707,819 (96,568,416,559–129,504,210,360)	120,218,789,335 (102,820,815,666–138,716,473,017)	132,741,560,494 (113,284,608,773–153,042,079,596)
Honduras	Injuries	18,849,825,457 (16,862,208,152–20,637,393,250)	20,068,175,919 (17,107,121,986–22,972,727,657)	24,276,449,789 (19,003,772,909–29,216,144,677)	27,110,045,483 (20,015,712,368–33,260,271,052)	30,962,103,587 (22,396,684,534–39,077,021,534)	30,321,487,778 (22,143,067,725–39,785,594,064)
Mexico	All causes	2,582,217,714,156 (2,406,697,171,553–2,788,257,871,227)	2,512,963,868,613 (2,313,322,435,533–2,740,460,719,813)	2,455,026,755,892 (2,231,033,018,698–2,700,597,712,846)	2,509,007,680,702 (2,263,862,330,161–2,773,547,023,188)	2,733,572,279,425 (2,456,009,201,945–3,039,140,504,304)	2,908,724,900,037 (2,603,337,218,500–3,244,865,873,074)
Mexico	Group I	920,717,947,288 (886,958,395,750–957,660,898,239)	718,168,210,325 (687,442,624,495–753,076,016,466)	568,664,777,147 (540,137,634,320–603,076,656,933)	475,644,876,199 (447,610,722,887–509,356,461,875)	415,257,766,971 (386,775,020,534–449,537,522,275)	367,190,013,588 (337,982,239,115–403,454,364,804)
Mexico	NCD	1,286,398,406,190 (1,135,396,048,229–1,456,986,214,422)	1,444,024,003,767 (1,271,269,106,254–1,638,259,712,415)	1,569,746,748,265 (1,376,623,557,840–1,784,764,084,211)	1,725,934,151,060 (1,514,277,487,058–1,960,982,666,506)	1,949,679,970,610 (1,708,044,993,543–2,216,348,541,642)	2,192,787,231,958 (1,923,111,388,916–2,490,190,156,278)
Mexico	Injuries	375,101,360,679 (365,943,839,310–386,664,633,474)	350,771,654,521 (344,082,109,330–359,996,036,933)	316,615,230,479 (309,230,897,655–325,505,727,669)	307,428,653,444 (300,267,784,017–315,640,141,098)	368,634,541,844 (361,014,325,889–377,681,497,285)	348,747,654,492 (340,835,636,090–358,066,063,774)
Nicaragua	All causes	150,628,546,694 (140,198,520,501–161,913,466,985)	136,856,907,449 (125,478,599,614–148,964,164,626)	123,965,264,672 (112,277,215,489–137,190,405,143)	119,665,271,239 (107,030,884,420–133,825,493,374)	119,558,107,646 (106,436,068,254–134,694,807,136)	124,008,738,795 (108,241,552,450–141,361,113,584)
Nicaragua	Group I	78,903,580,385 (73,933,578,427–83,960,510,408)	61,136,980,092 (57,419,154,368–65,259,479,652)	44,240,967,489 (40,930,394,604–47,972,385,402)	33,445,201,536 (30,305,728,306–36,833,967,743)	26,368,083,842 (23,193,445,292–29,839,763,738)	21,648,722,776 (18,486,314,731–25,159,212,951)
Nicaragua	NCD	54,870,295,179 (46,899,344,215–62,980,747,341)	59,550,562,480 (50,836,170,413–68,975,922,555)	64,287,978,943 (54,838,734,331–74,488,420,319)	71,244,238,954 (60,612,437,341–83,006,432,866)	78,908,710,141 (67,682,810,637–91,838,550,945)	88,026,035,967 (74,436,483,559–102,377,699,507)
Nicaragua	Injuries	16,854,671,130 (15,708,964,591–18,149,718,286)	16,169,364,877 (15,021,757,958–17,314,478,381)	15,436,318,240 (14,482,106,497–16,421,351,658)	14,975,830,749 (14,005,875,497–15,883,509,044)	14,281,313,663 (13,409,771,968–15,248,307,295)	14,333,980,052 (12,874,475,311–15,943,656,810)
Panama	All causes	60,064,883,715 (53,761,140,737–66,915,511,993)	65,986,305,210 (58,964,382,708–74,168,609,919)	70,035,525,331 (62,029,944,141–79,106,972,443)	75,716,522,479 (67,055,016,573–85,259,510,786)	84,626,864,175 (75,002,834,090–95,492,926,040)	90,024,907,098 (78,198,908,697–103,048,971,433)
Panama	Group I	15,832,492,379 (13,873,323,809–18,327,656,014)	16,013,599,876 (13,979,274,143–18,654,737,110)	16,295,352,136 (14,164,792,795–18,872,470,330)	17,162,923,987 (14,969,680,643–19,729,718,357)	17,220,607,783 (14,956,154,220–19,852,372,118)	16,266,460,075 (13,707,891,811–19,341,563,809)
Panama	NCD	35,882,796,554 (30,757,989,165–41,454,020,130)	40,397,804,103 (34,644,484,586–46,827,172,311)	44,087,440,979 (37,709,901,246–51,261,241,285)	49,000,576,491 (41,781,642,607–57,093,828,366)	55,617,575,110 (47,559,440,390–64,781,093,739)	62,267,997,647 (52,566,463,513–72,965,492,071)
Panama	Injuries	8,349,594,782 (7,747,867,085–8,944,257,265)	9,574,901,231 (8,891,269,137–10,236,518,358)	9,652,732,215 (8,933,975,331–10,385,140,265)	9,553,022,000 (8,726,858,831–10,353,709,822)	11,788,681,282 (10,790,991,351–12,745,384,769)	11,490,449,376 (9,946,752,826–13,126,134,717)
Venezuela	All causes	517,918,523,565 (472,293,662,613–569,993,335,311)	586,807,861,991 (529,000,102,951–649,522,507,112)	632,422,581,675 (568,391,352,376–705,278,373,933)	687,033,980,675 (614,967,845,015–770,101,337,602)	759,998,094,585 (679,820,888,459–852,176,612,283)	837,833,156,146 (729,572,287,588–951,876,578,483)
Venezuela	Group I	157,055,066,156 (146,378,831,955–170,734,128,821)	168,896,726,802 (154,161,762,461–186,414,438,446)	159,091,280,284 (140,952,691,329–182,625,234,511)	153,003,588,997 (133,901,437,111–179,584,838,399)	151,240,977,267 (131,190,322,697–179,523,060,361)	153,588,322,127 (130,492,462,423–182,494,229,003)
Venezuela	NCD	282,592,628,842 (246,078,568,110–321,320,346,896)	323,515,602,987 (281,658,488,161–368,518,020,786)	357,323,098,456 (310,769,411,488–409,023,461,648)	396,364,424,578 (342,915,782,628–454,345,263,534)	448,482,125,707 (389,000,411,465–513,251,815,402)	516,929,357,897 (441,975,498,910–594,495,806,823)
Venezuela	Injuries	78,270,828,568 (75,513,390,308–80,897,183,395)	94,395,532,202 (91,209,802,609–97,608,371,764)	116,008,202,935 (112,946,437,186–118,982,827,117)	137,665,967,100 (134,355,962,316–140,947,897,409)	160,274,991,610 (156,440,605,904–164,010,821,311)	167,315,476,121 (147,435,856,994–188,416,938,184)
Andean Latin America	All causes	1,658,455,386,800 (1,556,827,207,879–1,773,626,516,033)	1,527,796,423,001 (1,419,001,791,831–1,647,923,596,746)	1,372,051,064,454 (1,255,340,173,893–1,498,731,170,194)	1,299,946,056,755 (1,174,777,182,446–1,441,976,549,748)	1,296,537,492,822 (1,159,603,884,502–1,449,249,672,373)	1,317,495,900,287 (1,158,724,196,718–1,492,373,315,990)
Andean Latin America	Group I	775,699,539,972 (739,890,351,394–816,232,586,313)	633,704,995,640 (605,147,611,413–665,403,558,519)	482,992,268,393 (456,922,337,346–513,182,070,305)	390,694,675,933 (364,755,669,650–421,843,562,131)	327,926,262,010 (300,890,152,065–359,934,371,373)	285,341,018,616 (255,825,703,073–319,053,909,850)
Andean Latin America	NCD	656,575,744,397 (578,808,386,818–738,964,250,411)	684,367,242,714 (599,763,046,517–776,535,968,280)	703,171,724,699 (611,709,961,372–800,920,718,885)	740,932,542,801 (640,455,839,098–852,230,251,028)	800,131,951,293 (688,051,147,017–921,473,940,504)	871,429,894,012 (741,388,415,237–1,010,432,626,677)
Andean Latin America	Injuries	226,180,102,432 (200,277,381,920–248,344,984,497)	209,724,184,647 (194,057,742,593–226,036,897,182)	185,887,071,362 (174,128,502,935–196,373,660,593)	168,318,838,021 (156,662,959,202–178,906,895,093)	168,479,279,519 (156,530,044,649–180,833,278,101)	160,724,987,660 (146,053,025,274–176,843,467,571)
Bolivia	All causes	416,720,452,025 (395,473,052,312–439,085,830,838)	384,141,903,368 (361,483,995,346–408,578,485,339)	346,663,692,362 (322,887,677,214–372,199,814,817)	315,880,311,625 (288,859,934,908–344,208,188,370)	302,982,243,321 (270,172,565,430–338,143,301,497)	302,176,010,322 (264,131,304,057–344,445,764,196)
Bolivia	Group I	218,170,608,137 (203,641,485,820–231,826,152,761)	185,543,881,567 (174,012,720,951–197,742,424,967)	150,104,536,309 (141,134,871,294–158,714,058,521)	118,454,267,582 (109,276,748,776–127,945,327,628)	96,291,313,984 (85,614,045,186–107,834,232,173)	80,823,952,181 (69,512,187,063–92,750,603,177)
Bolivia	NCD	131,819,801,025 (116,089,020,402–148,088,581,515)	139,309,913,881 (123,164,314,011–155,805,828,024)	144,729,291,850 (127,757,273,311–163,160,084,513)	150,488,462,723 (131,427,625,429–169,663,828,763)	162,623,279,173 (139,427,904,944–186,729,813,717)	179,573,210,833 (153,443,922,382–207,426,636,699)
Bolivia	Injuries	66,730,042,863 (55,292,347,185–79,492,426,718)	59,288,107,920 (51,956,325,864–67,235,948,378)	51,829,864,202 (47,714,327,619–56,268,101,573)	46,937,581,320 (42,085,004,459–52,551,033,742)	44,067,650,164 (38,566,288,047–51,255,680,563)	41,778,847,308 (35,808,357,191–49,629,508,379)
Ecuador	All causes	342,554,440,846 (315,699,492,708–372,149,482,903)	348,559,475,287 (320,624,285,612–380,203,406,181)	351,679,092,265 (319,900,573,962–386,978,063,248)	359,930,690,583 (323,922,515,853–401,349,448,020)	370,964,166,918 (329,061,028,674–416,986,087,248)	386,571,674,792 (339,500,645,944–439,830,994,152)
Ecuador	Group I	136,152,661,721 (128,263,277,815–145,088,346,608)	122,848,819,288 (115,122,648,764–132,078,815,607)	109,583,851,389 (101,009,905,868–119,377,142,490)	94,886,957,981 (86,273,054,700–105,091,109,266)	80,950,882,315 (71,644,581,873–92,215,830,559)	71,828,420,722 (62,154,124,711–83,443,507,869)
Ecuador	NCD	159,685,050,114 (140,061,750,850–180,721,011,870)	173,332,095,857 (151,811,092,055–197,759,354,298)	190,435,614,838 (166,211,242,178–218,006,233,670)	211,894,693,084 (183,995,697,296–243,119,744,916)	234,388,431,572 (202,714,715,474–270,323,265,246)	260,202,679,281 (223,596,075,127–302,824,486,609)

Appendix Table 3. DALYs for all causes and level 1 cause groups by location for 1990 - 2015, both sexes combined.

	Cause	1990	1995	2000	2005	2010	2015
Ecuador	Injuries	46,716,729,010 (44,320,357,120–49,482,789,454)	52,378,560,142 (49,443,421,813–55,431,097,861)	51,659,626,038 (48,481,662,519–55,087,203,995)	53,149,029,519 (49,325,532,780–56,908,005,804)	55,624,853,032 (51,296,597,939–59,881,399,262)	54,540,574,789 (49,310,693,756–60,392,632,658)
Peru	All causes	899,180,493,929 (840,153,629,956–965,350,112,737)	795,095,044,346 (732,845,710,575–864,441,270,238)	673,708,279,827 (607,527,983,660–743,902,395,496)	624,135,054,547 (555,193,474,910–702,375,730,096)	622,591,082,583 (549,522,508,845–704,707,743,189)	628,748,215,173 (542,868,455,702–721,452,762,653)
Peru	Group I	421,376,270,114 (398,391,981,071–447,820,171,432)	325,312,294,786 (306,603,323,221–345,258,417,542)	223,303,880,696 (206,874,507,485–241,286,873,555)	177,353,450,370 (164,032,071,302–193,261,738,636)	150,684,065,711 (137,467,128,530–166,152,154,590)	132,688,645,713 (116,697,203,220–150,203,818,884)
Peru	NCD	365,070,893,258 (320,140,305,650–413,937,802,625)	371,725,232,977 (323,790,577,412–424,318,745,651)	368,006,818,010 (316,248,310,970–424,703,197,579)	378,549,386,995 (322,888,765,456–440,610,263,897)	403,120,240,549 (341,421,456,848–469,994,062,711)	431,654,003,898 (362,766,219,863–505,932,167,848)
Peru	Injuries	112,733,330,558 (95,388,517,136–127,836,871,142)	98,057,516,584 (85,309,518,315–109,424,387,415)	82,397,581,121 (74,709,463,184–89,057,661,663)	68,232,217,182 (62,432,785,428–73,330,528,920)	68,786,776,323 (63,005,220,633–74,463,357,002)	64,405,565,563 (57,142,781,741–72,555,817,278)
Caribbean	All causes	1,469,216,552,511 (1,369,825,081,534–1,581,123,803,911)	1,452,232,341,988 (1,344,930,477,418–1,571,449,753,114)	1,411,908,952,966 (1,289,847,243,383–1,541,727,010,703)	1,435,658,727,894 (1,315,686,994,027–1,574,658,835,890)	2,849,973,943,832 (1,687,061,748,884–4,094,677,891,692)	1,433,813,175,436 (1,285,938,337,460–1,596,450,642,322)
Caribbean	Group I	576,292,333,092 (538,591,365,647–620,114,849,197)	530,906,522,438 (495,129,488,819–569,722,895,048)	480,867,725,640 (448,261,933,743–517,171,284,995)	462,421,154,173 (432,315,845,227–496,413,038,576)	397,861,788,549 (367,289,428,214–432,759,842,118)	331,695,796,674 (292,271,866,131–375,183,930,331)
Caribbean	NCD	722,226,751,130 (644,949,552,493–802,617,195,585)	758,638,627,456 (675,177,145,494–848,744,381,436)	782,248,104,556 (691,393,250,331–880,626,823,873)	824,841,044,466 (728,225,596,893–933,025,488,101)	887,530,108,961 (781,080,329,684–1,002,477,391,402)	950,736,530,542 (837,057,639,431–1,075,404,246,695)
Caribbean	Injuries	170,697,468,290 (146,267,844,663–187,977,353,243)	162,687,192,095 (143,992,530,862–176,960,101,635)	148,793,122,771 (133,423,578,863–161,041,037,708)	148,396,529,255 (133,621,712,756–160,508,375,139)	1,564,582,046,322 (419,740,445,762–2,832,914,697,803)	151,380,848,220 (137,330,808,256–166,338,598,481)
Antigua and Barbuda	All causes	1,629,750,798 (1,455,274,154–1,818,400,783)	1,856,853,824 (1,659,189,655–2,073,255,323)	2,051,156,498 (1,827,395,660–2,295,113,815)	2,066,503,126 (1,836,502,546–2,328,994,288)	2,101,241,815 (1,855,387,088–2,388,872,190)	2,209,628,423 (1,923,948,553–2,521,424,062)
Antigua and Barbuda	Group I	291,426,511 (249,029,732–341,534,641)	358,333,477 (312,668,905–413,370,087)	432,768,482 (380,679,981–492,412,150)	354,865,314 (307,033,763–412,013,905)	320,717,784 (276,231,753–377,327,738)	313,466,037 (265,911,096–377,975,691)
Antigua and Barbuda	NCD	1,195,321,711 (1,053,274,184–1,347,567,584)	1,344,836,985 (1,185,813,500–1,521,236,932)	1,459,760,440 (1,271,564,971–1,656,485,290)	1,540,775,073 (1,347,204,456–1,756,583,305)	1,616,085,165 (1,409,718,332–1,852,653,062)	1,740,343,095 (1,505,165,716–2,003,067,722)
Antigua and Barbuda	Injuries	143,002,576 (131,738,568–156,007,239)	153,683,361 (141,151,505–167,988,492)	158,627,576 (144,271,066–173,623,622)	170,862,739 (157,159,279–184,377,381)	164,438,866 (148,952,283–179,974,122)	155,819,292 (137,930,924–175,889,103)
The Bahamas	All causes	8,436,815,943 (7,512,700,298–9,466,247,539)	8,921,412,906 (8,053,114,690–9,909,396,335)	9,339,219,423 (8,431,152,383–10,349,089,197)	9,918,640,220 (8,920,666,118–11,044,796,013)	10,937,906,909 (9,764,078,203–12,210,633,671)	11,538,814,587 (10,164,706,991–12,987,570,319)
The Bahamas	Group I	2,203,971,668 (1,750,857,629–2,719,327,105)	2,159,202,072 (1,889,200,117–2,495,436,313)	2,151,060,289 (1,915,110,912–2,405,364,986)	1,997,390,487 (1,728,182,075–2,324,598,077)	2,102,118,147 (1,802,533,071–2,489,059,736)	1,808,156,287 (1,461,893,298–2,357,038,445)
The Bahamas	NCD	5,080,184,664 (4,506,234,951–5,704,911,574)	5,592,772,758 (4,973,270,619–6,291,395,970)	6,008,460,506 (5,295,633,653–6,707,423,239)	6,680,330,265 (5,882,092,982–7,557,839,213)	7,453,166,597 (6,517,302,198–8,441,261,484)	8,435,580,381 (7,349,812,342–9,540,389,734)
The Bahamas	Injuries	1,152,659,611 (1,053,009,737–1,259,679,197)	1,169,438,076 (1,051,882,058–1,301,756,155)	1,179,698,628 (1,037,938,218–1,301,781,546)	1,240,919,468 (1,106,158,372–1,383,163,066)	1,382,622,165 (1,210,899,138–1,559,571,672)	1,295,077,918 (1,109,335,186–1,481,831,214)
Barbados	All causes	7,684,343,305 (6,816,892,033–8,587,359,680)	7,960,523,982 (7,093,423,096–8,905,475,687)	8,155,027,012 (7,250,374,419–9,122,730,601)	8,091,084,282 (7,169,153,318–9,075,445,630)	8,474,726,715 (7,561,025,251–9,482,419,250)	8,821,752,401 (7,754,467,881–9,936,651,740)
Barbados	Group I	1,337,764,697 (1,085,504,133–1,633,706,828)	1,329,578,498 (1,103,081,697–1,603,421,279)	1,434,424,977 (1,231,943,284–1,673,304,197)	1,187,515,544 (1,026,403,372–1,376,637,006)	1,125,674,716 (968,830,458–1,309,813,218)	1,052,486,729 (868,816,182–1,286,344,774)
Barbados	NCD	5,727,294,371 (5,096,018,867–6,418,985,721)	6,046,222,849 (5,356,657,157–6,811,331,366)	6,176,620,813 (5,457,705,633–6,975,477,931)	6,304,513,328 (5,523,209,582–7,152,374,805)	6,755,044,720 (5,962,564,017–7,633,622,098)	7,192,434,170 (6,280,052,173–8,207,848,851)
Barbados	Injuries	619,284,237 (578,888,427–662,624,519)	584,722,635 (544,100,990–628,587,047)	543,981,222 (499,369,389–589,586,768)	599,055,410 (560,457,821–639,533,987)	594,007,279 (550,007,395–637,999,451)	576,831,503 (511,423,150–642,710,622)
Belize	All causes	5,870,969,678 (5,274,307,651–6,524,448,516)	6,063,251,257 (5,463,628,159–6,789,175,936)	7,298,293,308 (6,618,149,081–8,081,120,527)	7,669,135,836 (6,906,816,571–8,572,841,740)	8,516,887,099 (7,654,237,201–9,445,443,863)	9,365,419,557 (8,192,737,806–10,602,110,842)
Belize	Group I	2,215,692,357 (1,923,809,939–2,530,679,581)	1,858,156,229 (1,610,812,925–2,159,020,151)	2,075,447,751 (1,814,657,920–2,371,017,228)	2,078,218,649 (1,816,392,436–2,376,034,869)	2,001,703,056 (1,728,116,352–2,348,969,627)	2,084,555,907 (1,676,194,663–2,627,606,359)
Belize	NCD	2,907,659,140 (2,534,064,008–3,311,343,607)	3,384,932,738 (2,993,038,998–3,823,951,198)	4,032,257,957 (3,559,683,567–4,532,553,256)	4,320,648,121 (3,772,097,889–4,946,713,639)	5,150,453,223 (4,470,015,112–5,857,534,694)	5,854,355,747 (4,996,208,199–6,687,298,288)
Belize	Injuries	747,618,181 (670,925,929–827,058,578)	820,162,290 (754,926,968–892,500,176)	1,190,587,600 (1,073,732,705–1,314,983,920)	1,270,269,066 (1,153,197,900–1,404,649,262)	1,364,730,820 (1,207,941,123–1,507,861,856)	1,426,507,903 (1,203,537,119–1,645,219,537)
Bermuda	All causes	1,547,515,318 (1,400,860,560–1,707,740,869)	1,531,487,997 (1,373,947,543–1,697,845,408)	1,506,900,149 (1,341,331,003–1,695,020,331)	1,452,470,917 (1,274,510,691–1,641,248,269)	1,452,476,937 (1,272,985,747–1,654,130,783)	1,494,135,612 (1,303,148,403–1,699,659,294)
Bermuda	Group I	184,309,893 (161,160,301–214,234,710)	196,701,904 (173,435,488–225,858,613)	223,227,951 (199,693,824–251,481,001)	174,603,882 (151,897,467–202,820,880)	160,676,602 (137,602,761–189,217,725)	154,620,698 (130,869,261–183,494,615)
Bermuda	NCD	1,190,012,817 (1,068,195,676–1,320,076,531)	1,186,137,342 (1,053,191,538–1,318,381,954)	1,164,244,666 (1,023,776,600–1,315,824,020)	1,147,919,915 (1,000,286,623–1,309,176,809)	1,171,292,515 (1,017,552,878–1,338,509,374)	1,232,238,722 (1,067,488,179–1,409,850,890)
Bermuda	Injuries	173,192,608 (161,970,561–185,261,034)	148,648,752 (138,185,742–159,662,614)	119,427,532 (106,742,363–132,092,761)	129,947,120 (118,694,805–142,093,458)	120,507,820 (108,585,283–133,781,374)	107,276,192 (93,312,719–121,383,112)
Cuba	All causes	278,252,355,775 (250,190,254,734–310,327,283,805)	286,792,250,137 (257,214,326,351–320,869,377,517)	283,229,122,671 (252,666,899,291–318,536,028,403)	284,941,364,892 (252,710,270,180–321,841,232,031)	295,281,185,383 (262,163,697,187–332,935,544,412)	313,195,032,803 (278,574,920,033–351,925,314,327)
Cuba	Group I	34,952,211,245 (30,909,774,985–40,232,547,975)	32,376,765,722 (28,222,235,123–37,852,690,450)	29,120,124,370 (25,210,745,656–34,155,200,190)	26,732,826,551 (23,139,009,335–31,652,301,140)	25,851,546,445 (22,318,057,807–30,451,625,776)	25,956,600,347 (22,315,976,495–30,594,830,604)
Cuba	NCD	201,175,228,108 (177,728,823,794–226,766,285,787)	214,461,111,746 (190,006,805,998–241,671,963,301)	221,768,947,953 (195,519,698,908–250,868,739,365)	231,487,920,209 (204,144,085,808–262,179,245,064)	244,703,549,972 (216,219,060,175–276,727,905,609)	262,049,811,329 (232,212,172,634–295,717,987,926)
Cuba	Injuries	42,124,916,423 (40,423,735,052–43,968,445,095)	39,954,372,670 (38,244,190,365–41,875,028,761)	32,340,450,348 (30,788,573,720–34,191,846,453)	26,720,618,131 (25,290,484,448–28,450,337,332)	24,726,088,966 (23,256,746,126–26,543,950,203)	25,188,621,127 (23,454,870,502–27,288,372,962)
Dominica	All causes	1,933,651,932 (1,720,770,777–2,167,698,005)	1,986,536,321 (1,771,070,616–2,227,275,036)	1,883,666,441 (1,671,645,122–2,104,954,159)	1,922,321,445 (1,710,485,631–2,155,992,081)	2,058,467,821 (1,827,473,865–2,309,745,037)	2,214,778,508 (1,943,398,313–2,500,788,590)
Dominica	Group I	443,244,464 (366,082,598–526,190,049)	421,419,216 (357,170,770–494,155,968)	367,547,548 (315,832,056–423,717,871)	351,265,669 (300,810,624–412,270,654)	356,688,075 (299,619,582–430,475,567)	363,862,810 (290,312,861–454,940,354)
Dominica	NCD	1,327,958,676 (1,175,098,081–1,507,928,772)	1,396,905,683 (1,233,045,749–1,577,927,677)	1,350,131,250 (1,183,893,899–1,526,396,178)	1,393,001,091 (1,220,085,933–1,578,479,385)	1,505,521,217 (1,319,829,692–1,707,408,475)	1,650,787,759 (1,442,300,600–1,874,085,326)
Dominica	Injuries	162,448,792 (148,653,771–177,913,793)	168,211,421 (154,467,971–183,743,085)	165,387,643 (151,059,501–180,262,950)	178,054,684 (161,824,160–194,062,832)	196,258,530 (176,721,879–214,087,409)	200,127,939 (175,797,770–227,564,145)
Dominican Republic	All causes	247,666,552,826 (228,620,136,647–268,725,481,686)	240,948,281,588 (221,432,028,019–264,662,031,947)	245,142,052,406 (221,537,514,786–270,872,779,679)	265,887,393,624 (242,040,846,526–293,138,188,684)	265,792,646,395 (239,422,205,117–296,645,632,095)	269,204,303,857 (240,149,731,662–301,436,267,326)
Dominican Republic	Group I	112,230,264,634 (104,011,767,110–120,068,578,288)	95,727,167,888 (89,024,535,049–103,453,343,799)	91,228,684,828 (82,597,227,470–100,670,426,885)	94,435,311,149 (86,431,986,477–103,487,361,846)	77,805,012,463 (69,864,139,812–86,717,359,556)	64,024,320,579 (55,103,586,199–72,816,000,843)
Dominican Republic	NCD	110,297,979,963 (96,001,887,477–125,423,784,975)	120,003,033,284 (103,560,830,549–137,584,689,296)	126,826,833,509 (109,612,229,243–146,342,749,321)	140,965,332,074 (121,667,502,211–161,526,927,159)	156,262,513,093 (135,355,856,720–179,695,043,693)	173,107,838,287 (150,515,914,032–198,081,225,751)
Dominican Republic	Injuries	25,138,308,228 (23,326,812,255–27,746,800,339)	25,218,080,417 (23,277,220,322–27,094,605,337)	27,086,534,069 (25,381,539,548–29,032,005,438)	30,486,750,401 (28,576,928,319–32,416,195,967)	31,725,120,838 (28,945,465,981–33,925,512,739)	32,072,144,990 (29,562,145,900–35,019,662,027)
Grenada	All causes	3,222,541,859 (2,818,317,059–3,689,312,502)	2,977,507,380 (2,659,452,770–3,320,864,775)	3,049,660,949 (2,737,395,175–3,408,325,037)	3,198,761,652 (2,893,257,852–3,563,180,749)	3,198,001,507 (2,874,881,049–3,567,529,241)	3,267,791,397 (2,900,202,886–3,669,676,485)
Grenada	Group I	848,721,480 (640,869,735–1,114,303,557)	623,197,684 (507,435,931–748,733,711)	588,495,177 (496,647,888–706,010,081)	543,414,685 (455,035,284–644,342,727)	520,660,225 (434,972,767–631,460,313)	520,698,083 (412,990,743–664,913,944)

Appendix Table 3. DALYs for all causes and level 1 cause groups by location for 1990 - 2015, both sexes combined.

	Cause	1990	1995	2000	2005	2010	2015
Grenada	NCD	2,085,452,276<br>(1,856,886,373–2,314,737,487)	2,076,260,209<br>(1,855,871,715–2,316,531,318)	2,142,570,725<br>(1,916,669,364–2,398,211,002)	2,272,162,368<br>(2,033,163,996–2,548,677,500)	2,309,912,109<br>(2,064,278,297–2,586,959,095)	2,408,566,161<br>(2,130,130,759–2,707,493,318)
Grenada	Injuries	288,368,103<br>(259,655,811–323,934,224)	278,049,487<br>(256,182,801–301,358,746)	318,595,047<br>(293,348,035–345,913,640)	383,184,599<br>(345,057,785–424,421,050)	367,429,174<br>(329,240,268–405,576,359)	338,527,153<br>(292,507,018–380,826,923)
Guyana	All causes	32,034,811,044<br>(29,781,370,588–34,523,223,444)	30,825,331,935<br>(28,573,664,283–33,164,202,261)	30,657,031,807<br>(28,359,079,514–33,025,763,325)	29,883,936,381<br>(27,577,562,751–32,467,495,318)	29,174,216,322<br>(26,711,942,550–31,715,827,407)	29,024,706,125<br>(26,161,972,425–32,143,780,435)
Guyana	Group I	12,473,654,336<br>(11,604,893,539–13,362,732,979)	10,766,298,757<br>(10,044,159,500–11,540,203,592)	10,373,272,211<br>(9,644,897,645–11,156,153,524)	9,078,761,391<br>(8,255,990,361–10,262,538,910)	8,219,270,944<br>(7,191,567,920–9,576,529,220)	7,311,545,127<br>(6,250,180,801–8,570,433,520)
Guyana	NCD	15,502,792,117<br>(13,953,322,066–17,102,679,600)	16,082,913,456<br>(14,585,451,872–17,786,452,221)	15,848,012,327<br>(14,237,313,660–17,581,100,784)	16,318,631,701<br>(14,670,272,720–18,266,095,318)	16,899,231,485<br>(15,015,314,449–18,842,584,823)	17,762,959,251<br>(15,620,458,124–20,090,631,063)
Guyana	Injuries	4,058,364,590<br>(3,801,897,645–4,414,140,260)	3,976,119,722<br>(3,728,348,882–4,312,560,227)	4,435,747,269<br>(4,065,241,628–4,843,893,446)	4,486,543,289<br>(3,944,026,422–4,982,917,740)	4,055,713,892<br>(3,536,622,771–4,557,629,173)	3,950,201,747<br>(3,368,994,560–4,528,343,833)
Haiti	All causes	600,966,969,726<br>(568,324,447,395–634,838,001,584)	566,261,572,228<br>(530,617,486,560–603,408,667,274)	520,902,391,835<br>(481,240,710,379–558,403,012,491)	520,519,151,105<br>(478,526,489,169–567,664,434,960)	1,870,360,334,463<br>(769,579,219,068–3,091,226,558,916)	472,194,808,810<br>(406,553,414,103–541,068,684,791)
Haiti	Group I	341,517,390,563<br>(312,947,339,637–376,587,508,463)	318,379,916,273<br>(291,029,621,486–350,573,390,834)	281,932,057,107<br>(258,180,568,041–310,414,537,279)	270,528,338,839<br>(248,838,669,170–294,093,273,333)	229,740,966,505<br>(207,591,213,454–254,938,052,543)	183,746,655,023<br>(152,150,340,018–221,777,931,400)
Haiti	NCD	193,336,156,046<br>(173,418,825,984–216,125,984,204)	189,778,315,537<br>(168,739,349,264–212,854,432,653)	188,152,494,754<br>(164,870,881,984–213,669,891,370)	199,757,415,979<br>(173,057,406,484–229,074,402,938)	218,974,952,790<br>(187,145,944,403–251,192,468,285)	233,211,817,794<br>(196,549,457,661–272,107,941,489)
Haiti	Injuries	66,113,423,117<br>(43,227,823,850–82,999,115,912)	58,103,340,418<br>(40,693,678,472–71,468,071,354)	50,817,839,974<br>(37,312,679,944–60,915,133,432)	50,233,396,286<br>(37,660,642,045–60,899,094,383)	1,421,644,415,167<br>(315,206,925,406–2,649,857,242,240)	55,236,335,993<br>(43,080,132,331–68,360,791,160)
Jamaica	All causes	63,353,179,556<br>(56,124,286,194–71,689,831,239)	65,073,553,558<br>(57,471,248,085–73,548,853,245)	69,949,412,873<br>(62,173,868,666–78,835,727,688)	70,852,512,967<br>(62,422,066,373–80,550,146,548)	74,879,660,232<br>(65,799,958,495–84,264,439,755)	78,501,310,801<br>(68,666,740,902–89,375,107,232)
Jamaica	Group I	17,386,824,790<br>(14,462,796,160–20,959,405,931)	15,761,068,556<br>(13,085,732,337–19,207,578,190)	15,864,904,265<br>(13,248,303,911–19,023,883,488)	14,547,182,304<br>(12,055,284,303–17,863,402,853)	13,890,491,092<br>(11,191,398,225–16,912,317,016)	12,816,721,562<br>(10,175,330,397–16,214,886,874)
Jamaica	NCD	42,349,230,708<br>(37,186,468,930–48,242,254,436)	44,442,327,955<br>(39,014,419,507–50,568,930,914)	48,217,778,737<br>(42,273,025,635–54,735,510,895)	48,973,315,750<br>(42,584,150,262–55,882,876,153)	53,663,992,920<br>(46,763,579,179–60,876,456,284)	58,504,316,965<br>(50,555,853,606–66,979,258,530)
Jamaica	Injuries	3,617,124,058<br>(3,289,609,169–4,133,855,444)	4,870,157,047<br>(4,356,672,178–5,322,915,679)	5,866,729,871<br>(5,070,875,173–6,454,423,099)	7,332,014,913<br>(5,854,134,194–8,132,613,015)	7,325,176,220<br>(5,511,258,617–8,113,713,931)	7,180,272,273<br>(5,477,222,495–8,207,318,594)
Puerto Rico	All causes	103,340,966,796<br>(93,331,476,985–114,642,478,929)	114,559,369,767<br>(103,996,593,129–126,602,763,176)	108,702,497,493<br>(97,496,944,724–121,461,950,030)	105,364,810,368<br>(94,089,188,099–118,380,752,724)	104,048,585,053<br>(92,934,643,317–116,714,906,764)	100,323,813,965<br>(88,719,436,798–113,259,143,816)
Puerto Rico	Group I	17,839,448,829<br>(16,571,133,617–19,511,710,592)	19,981,902,509<br>(18,686,856,857–21,660,568,522)	14,394,986,573<br>(13,113,409,716–16,103,774,687)	12,323,396,576<br>(11,131,800,803–13,889,395,550)	10,610,374,101<br>(9,471,878,194–11,981,289,283)	9,487,897,185<br>(8,356,426,716–10,926,181,087)
Puerto Rico	NCD	72,318,395,958<br>(63,874,878,371–81,636,203,865)	80,173,795,816<br>(71,366,539,262–90,339,373,417)	82,144,358,406<br>(72,646,074,632–92,897,774,879)	81,815,613,215<br>(71,947,024,806–93,023,290,558)	82,178,431,594<br>(72,519,967,155–93,132,509,539)	80,957,671,549<br>(70,349,873,630–92,383,064,960)
Puerto Rico	Injuries	13,183,122,009<br>(12,627,898,730–13,732,180,343)	14,403,671,443<br>(13,787,810,042–15,011,903,957)	12,163,152,515<br>(11,615,494,754–12,726,402,353)	11,225,800,577<br>(10,743,963,656–11,817,498,617)	11,259,779,359<br>(10,810,998,746–11,809,825,681)	9,878,245,231<br>(9,172,908,141–10,640,507,456)
Saint Lucia	All causes	4,643,480,534<br>(4,054,700,052–5,369,323,632)	4,513,463,191<br>(4,024,333,564–5,049,441,894)	4,511,622,263<br>(4,037,471,369–5,035,168,140)	4,573,094,358<br>(4,058,807,355–5,162,068,951)	4,952,031,927<br>(4,398,549,877–5,548,296,299)	5,193,574,223<br>(4,564,331,214–5,904,486,908)
Saint Lucia	Group I	1,284,838,821<br>(968,402,597–1,663,749,171)	980,821,572<br>(808,707,107–1,189,302,139)	863,726,070<br>(724,396,997–1,017,929,442)	803,772,970<br>(659,522,716–980,752,843)	782,523,182<br>(639,317,165–957,513,702)	715,571,142<br>(559,519,308–937,168,913)
Saint Lucia	NCD	2,916,043,037<br>(2,601,902,563–3,269,873,888)	3,089,085,274<br>(2,761,450,035–3,448,298,363)	3,176,028,245<br>(2,813,458,159–3,575,461,806)	3,247,522,336<br>(2,869,920,467–3,674,233,204)	3,625,683,122<br>(3,195,205,925–4,103,186,699)	3,975,151,786<br>(3,484,222,203–4,516,405,502)
Saint Lucia	Injuries	442,598,676<br>(398,193,179–495,942,775)	443,556,346<br>(410,791,171–481,177,693)	471,867,948<br>(431,212,695–510,096,369)	521,799,053<br>(486,652,564–562,919,710)	543,825,622<br>(507,583,254–582,231,734)	502,851,294<br>(449,828,829–562,166,942)
Saint Vincent and the Grenadines	All causes	3,476,773,225<br>(3,059,065,466–3,930,149,931)	3,487,095,232<br>(3,112,130,424–3,882,355,864)	3,594,476,435<br>(3,248,507,630–3,973,577,141)	3,433,430,581<br>(3,107,659,178–3,803,117,361)	3,398,852,369<br>(3,064,426,807–3,770,009,259)	3,510,534,487<br>(3,138,955,077–3,912,399,273)
Saint Vincent and the Grenadines	Group I	1,047,613,551<br>(848,765,525–1,296,890,667)	921,188,340<br>(776,727,249–1,078,877,771)	941,115,141<br>(826,825,788–1,073,031,237)	722,962,691<br>(629,359,245–843,440,003)	644,475,127<br>(550,282,564–771,129,385)	573,339,689<br>(471,599,619–708,964,890)
Saint Vincent and the Grenadines	NCD	2,077,815,188<br>(1,833,974,040–2,327,407,767)	2,230,745,331<br>(1,989,644,134–2,499,557,769)	2,326,836,305<br>(2,077,987,097–2,596,559,432)	2,338,658,525<br>(2,081,637,824–2,625,034,564)	2,381,263,618<br>(2,119,854,180–2,671,631,061)	2,568,862,221<br>(2,281,018,362–2,911,712,959)
Saint Vincent and the Grenadines	Injuries	351,344,485<br>(315,378,366–391,670,706)	335,161,561<br>(303,971,827–368,232,256)	326,524,988<br>(299,994,840–355,537,735)	371,809,364<br>(342,450,010–403,989,420)	373,113,625<br>(342,927,809–403,381,992)	368,332,577<br>(327,656,321–408,359,950)
Suriname	All causes	14,403,823,193<br>(13,185,472,250–15,701,346,182)	15,070,583,504<br>(13,737,651,566–16,484,401,478)	16,107,878,924<br>(14,638,276,600–17,699,078,158)	16,526,826,063<br>(15,036,454,684–18,226,687,205)	16,798,854,328<br>(15,116,016,893–18,560,966,985)	16,763,746,631<br>(14,890,606,330–18,643,969,459)
Suriname	Group I	4,864,366,979<br>(4,419,117,082–5,385,165,471)	4,831,321,898<br>(4,326,704,997–5,319,337,319)	4,812,576,918<br>(4,314,866,235–5,366,962,093)	4,326,101,002<br>(3,827,735,952–4,880,908,653)	4,003,027,381<br>(3,509,629,970–4,592,883,638)	3,594,309,792<br>(3,108,537,741–4,182,558,231)
Suriname	NCD	7,678,522,904<br>(6,815,845,155–8,607,588,135)	8,299,619,995<br>(7,365,186,435–9,329,399,002)	9,219,683,207<br>(8,175,980,619–10,399,452,320)	9,952,440,506<br>(8,790,014,490–11,234,430,383)	10,509,428,192<br>(9,232,061,129–11,865,013,658)	10,997,327,688<br>(9,546,691,302–12,515,633,291)
Suriname	Injuries	1,860,933,310<br>(1,735,675,536–2,000,017,350)	1,939,641,612<br>(1,793,582,366–2,088,739,234)	2,075,618,799<br>(1,903,949,816–2,257,307,784)	2,248,284,555<br>(2,061,969,310–2,461,520,276)	2,286,398,755<br>(2,064,363,919–2,521,055,715)	2,172,109,151<br>(1,923,515,633–2,462,604,946)
Trinidad and Tobago	All causes	38,531,331,265<br>(34,959,623,410–42,321,759,098)	39,413,443,479<br>(35,805,795,778–43,287,106,436)	41,588,125,628<br>(37,880,737,522–45,604,442,418)	42,007,461,333<br>(38,129,132,271–46,436,505,673)	43,704,366,757<br>(39,356,467,103–48,182,063,352)	45,005,251,712<br>(39,990,021,088–50,504,710,212)
Trinidad and Tobago	Group I	7,982,719,339<br>(7,125,589,944–8,906,788,390)	7,559,793,304<br>(6,805,421,112–8,370,159,762)	8,491,619,628<br>(7,785,910,169–9,327,699,303)	6,666,892,004<br>(5,926,189,802–7,505,989,511)	6,042,874,647<br>(5,175,527,812–7,070,155,778)	5,626,638,198<br>(4,632,924,548–6,878,288,873)
Trinidad and Tobago	NCD	26,078,749,271<br>(23,355,674,068–28,999,763,103)	27,608,087,761<br>(24,771,580,902–30,721,721,655)	28,965,262,605<br>(25,967,568,579–32,377,046,104)	29,996,921,272<br>(26,764,569,249–33,610,559,374)	32,137,812,183<br>(28,487,570,007–36,038,648,115)	34,328,733,507<br>(30,214,271,666–38,754,614,361)
Trinidad and Tobago	Injuries	4,469,862,655<br>(4,247,655,934–4,694,979,577)	4,245,562,414<br>(4,052,103,905–4,461,382,548)	4,131,243,395<br>(3,932,024,136–4,318,720,575)	5,343,648,057<br>(5,131,703,511–5,568,185,942)	5,523,679,927<br>(5,232,741,107–5,801,472,523)	5,049,880,007<br>(4,523,241,784–5,637,727,550)
Virgin Islands, U.S.	All causes	2,842,426,144<br>(2,552,013,107–3,169,035,082)	3,011,876,057<br>(2,705,699,979–3,346,287,666)	3,153,621,031<br>(2,837,370,866–3,517,422,747)	3,338,108,386<br>(3,002,285,665–3,713,230,840)	3,589,128,673<br>(3,222,389,887–3,972,526,872)	3,820,516,485<br>(3,402,858,908–4,242,952,354)
Virgin Islands, U.S.	Group I	426,200,822<br>(378,358,001–483,217,521)	368,078,874<br>(320,865,449–424,503,091)	337,267,682<br>(294,899,619–390,067,348)	307,232,354<br>(269,318,744–357,037,631)	288,741,778<br>(248,792,624–338,572,781)	275,688,694<br>(236,201,916–324,184,839)
Virgin Islands, U.S.	NCD	2,078,539,467<br>(1,843,865,758–2,334,848,623)	2,277,210,430<br>(2,025,654,868–2,549,564,525)	2,480,514,208<br>(2,206,420,329–2,788,422,961)	2,667,687,951<br>(2,369,861,945–2,983,298,934)	2,930,940,238<br>(2,616,748,362–3,268,668,066)	3,187,270,900<br>(2,823,856,541–3,556,885,975)
Virgin Islands, U.S.	Injuries	337,685,855<br>(311,980,827–370,617,472)	366,586,752<br>(333,567,841–407,028,631)	335,839,142<br>(310,044,950–363,493,418)	363,188,081<br>(335,484,018–390,155,261)	369,446,658<br>(332,170,785–400,806,383)	357,556,890<br>(318,347,005–398,856,176)
Tropical Latin America	All causes	5,394,973,890,098<br>(4,990,308,957,959–5,840,015,531,272)	5,425,804,534,092<br>(4,989,655,415,617–5,904,783,980,872)	5,491,585,431,632<br>(5,009,539,415,925–6,031,878,249,063)	5,526,273,433,363<br>(4,998,600,347,162–6,106,629,983,369)	5,682,336,621,014<br>(5,096,648,598,622–6,316,799,812,577)	5,963,730,659,158<br>(5,332,096,657,733–6,653,319,216,044)
Tropical Latin America	Group I	1,888,291,129,128<br>(1,795,056,309,598–1,984,152,275,344)	1,550,180,801,511<br>(1,475,018,187,562–1,640,867,479,012)	1,291,602,739,847<br>(1,215,750,461,720–1,386,622,465,588)	1,066,841,914,358<br>(994,296,415,287–1,157,671,529,763)	931,703,309,764<br>(854,195,830,346–1,024,977,756,091)	853,699,720,910<br>(771,797,115,544–952,316,502,296)
Tropical Latin America	NCD	2,703,987,963,149<br>(2,372,424,561,772–3,049,180,800,186)	3,004,798,012,846<br>(2,639,570,693,049–3,381,418,445,451)	3,315,541,076,936<br>(2,912,627,852,490–3,764,137,824,452)	3,570,410,884,174<br>(3,118,599,029,639–4,059,187,270,760)	3,849,288,196,955<br>(3,355,927,809,227–4,383,703,805,894)	4,230,329,027,261<br>(3,692,160,631,346–4,811,554,386,952)
Tropical Latin America	Injuries	802,694,797,821<br>(775,103,665,462–833,250,846,889)	870,825,719,735<br>(839,975,723,257–903,831,823,059)	884,441,614,849<br>(851,306,032,454–921,283,278,091)	889,020,634,831<br>(853,787,079,787–924,803,888,775)	901,345,114,295<br>(861,734,229,563–938,639,286,984)	879,701,910,986<br>(832,351,814,795–927,907,823,729)
Brazil	All causes	5,282,728,327,000<br>(4,887,613,354,718–5,717,452,899,691)	5,304,043,899,279<br>(4,879,693,693,438–5,770,881,546,811)	5,357,952,036,792<br>(4,889,097,856,324–5,882,587,289,506)	5,381,439,395,598<br>(4,870,296,499,042–5,948,097,342,119)	5,527,371,789,872<br>(4,958,843,642,868–6,144,943,152,577)	5,798,345,400,561<br>(5,184,742,890,558–6,464,034,111,981)

Appendix Table 3. DALYs for all causes and level 1 cause groups by location for 1990 - 2015, both sexes combined.

	Cause	1990	1995	2000	2005	2010	2015
Brazil	Group I	1,846,099,138,956 (1,754,727,943,750–1,941,156,405,368)	1,510,228,948,437 (1,436,857,241,312–1,597,965,460,728)	1,252,062,861,313 (1,178,607,613,073–1,344,662,173,631)	1,031,009,176,728 (960,704,744,673–1,118,657,142,818)	899,896,001,709 (824,461,330,546–990,820,663,395)	824,460,262,254 (745,672,303,757–919,961,096,888)
Brazil	NCD	2,645,265,243,284 (2,321,179,438,676–2,981,379,153,371)	2,937,050,306,346 (2,581,978,126,462–3,304,470,637,126)	3,238,283,486,450 (2,846,742,428,457–3,674,643,765,033)	3,480,204,921,671 (3,039,924,783,032–3,954,855,988,518)	3,746,914,748,584 (3,269,493,636,615–4,266,783,392,060)	4,116,154,967,121 (3,594,495,353,236–4,681,614,638,828)
Brazil	Injuries	791,363,944,760 (763,877,563,475–821,663,274,888)	856,764,644,497 (826,284,751,854–889,577,755,393)	867,605,689,029 (835,389,911,765–904,280,415,690)	870,225,297,200 (836,200,363,565–905,553,236,895)	880,561,039,580 (841,818,807,706–916,780,775,959)	857,730,171,185 (811,672,498,604–904,659,191,769)
Paraguay	All causes	112,245,563,098 (101,181,694,921–123,953,714,549)	121,760,634,812 (109,041,899,650–135,608,853,194)	133,633,394,840 (119,902,697,984–149,024,614,051)	144,834,037,765 (129,108,478,102–162,556,553,890)	154,964,831,141 (137,102,148,415–174,019,940,257)	165,385,258,597 (144,480,126,377–188,676,375,811)
Paraguay	Group I	42,191,990,172 (38,935,464,207–45,514,346,132)	39,951,853,074 (36,704,478,272–43,596,958,754)	39,539,878,534 (35,880,932,694–43,847,677,221)	35,832,737,631 (31,998,655,454–40,208,172,502)	31,807,308,055 (27,429,512,422–36,713,616,029)	29,239,458,656 (24,868,873,692–34,392,569,444)
Paraguay	NCD	58,722,719,865 (50,398,393,985–67,492,291,687)	67,747,706,500 (57,808,888,175–78,077,310,389)	77,257,590,486 (66,408,377,339–88,929,360,109)	90,205,962,503 (77,840,921,927–104,163,642,659)	102,373,448,371 (88,897,623,599–118,283,690,558)	114,174,060,140 (98,450,214,020–132,228,922,584)
Paraguay	Injuries	11,330,853,061 (10,497,287,105–12,171,038,200)	14,061,075,238 (12,961,003,025–15,169,696,162)	16,835,925,820 (15,616,486,679–18,117,950,316)	18,795,337,631 (17,397,911,149–20,294,266,432)	20,784,074,716 (18,847,397,385–22,877,953,107)	21,971,739,801 (18,888,970,460–26,138,813,644)
Southeast Asia, East Asia, and Oceania	All causes	63,736,352,199,278 (59,617,541,905,217–68,547,842,432,654)	60,428,840,157,281 (56,072,109,258,136–65,246,414,826,686)	58,565,650,014,860 (54,155,535,161,367–63,567,253,087,315)	56,913,104,320,107 (51,964,058,354,936–62,335,635,890,600)	55,368,391,105,168 (49,993,202,841,869–61,245,429,624,425)	55,017,452,596,870 (49,562,546,996,539–61,249,648,054,824)
Southeast Asia, East Asia, and Oceania	Group I	22,356,339,795,209 (21,034,673,828,962–23,857,836,692,501)	17,362,645,466,954 (16,239,674,041,295–18,604,194,177,288)	13,635,525,583,913 (12,691,510,124,753–14,699,739,324,673)	10,427,003,489,076 (9,649,674,868,401–11,352,636,899,932)	8,623,876,941,348 (7,902,053,248,736–9,473,297,358,454)	7,434,503,013,783 (6,758,607,097,427–8,213,343,259,710)
Southeast Asia, East Asia, and Oceania	NCD	33,089,246,793,620 (29,819,743,906,310–36,556,955,463,483)	35,073,986,616,176 (31,558,685,746,019–38,757,365,386,158)	37,523,438,527,266 (33,939,229,723,873–41,490,325,451,847)	39,595,181,346,392 (35,508,729,628,473–43,929,621,431,853)	40,425,529,707,111 (35,903,392,594,991–45,178,303,696,915)	41,935,874,991,617 (37,351,357,778,979–47,024,556,817,075)
Southeast Asia, East Asia, and Oceania	Injuries	8,290,765,610,449 (7,659,444,809,453–8,780,746,829,353)	7,992,208,074,152 (7,451,323,781,824–8,406,366,975,143)	7,406,685,903,682 (7,074,745,229,710–7,734,754,616,370)	6,890,919,484,639 (6,537,530,731,534–7,214,810,077,070)	6,318,984,456,709 (5,920,569,520,812–6,658,127,219,905)	5,647,074,591,470 (5,217,449,611,517–6,049,361,507,751)
East Asia	All causes	43,830,439,976,618 (40,833,451,582,301–47,337,909,791,249)	41,269,867,229,810 (38,119,555,977,500–44,713,980,438,842)	39,456,290,499,888 (36,246,026,085,428–42,872,433,144,125)	37,951,240,235,655 (34,436,862,874,825–41,714,377,968,254)	36,409,504,437,233 (32,852,882,753,903–40,456,479,672,997)	35,862,561,471,974 (32,150,411,518,527–40,059,178,243,013)
East Asia	Group I	12,404,256,846,351 (11,490,912,588,898–13,338,276,870,741)	9,144,396,388,541 (8,391,298,822,803–9,943,462,184,930)	6,574,217,126,937 (5,956,086,210,466–7,260,125,585,214)	4,279,085,502,850 (3,850,411,047,475–4,796,891,534,982)	3,365,409,233,953 (2,998,649,271,454–3,823,817,609,679)	2,939,569,304,984 (2,583,774,907,398–3,378,653,233,965)
East Asia	NCD	25,031,526,972,916 (22,749,627,772,697–27,672,318,119,773)	26,097,813,161,307 (23,625,988,407,236–28,744,151,525,913)	27,497,942,288,593 (24,904,983,924,398–30,479,876,229,780)	28,765,205,437,557 (25,866,957,376,514–31,943,296,635,049)	28,673,717,086,027 (25,603,370,925,653–32,046,421,624,106)	29,133,515,318,170 (25,973,462,540,505–32,794,697,624,394)
East Asia	Injuries	6,394,656,157,351 (5,830,008,802,563–6,812,576,587,662)	6,027,657,679,962 (5,564,163,086,926–6,375,683,944,859)	5,384,131,084,358 (5,135,329,875,287–5,623,464,378,902)	4,906,949,295,248 (4,629,865,419,087–5,153,202,782,327)	4,370,378,117,253 (4,059,350,763,294–4,642,177,021,840)	3,789,476,848,820 (3,464,133,343,353–4,083,452,597,292)
China	All causes	42,687,819,372,294 (39,829,807,317,213–46,128,577,547,797)	39,580,113,653,104 (36,545,622,638,461–42,906,688,915,644)	37,738,125,793,126 (34,639,226,024,889–41,165,672,248,028)	36,670,890,463,463 (33,269,327,341,568–40,286,824,817,547)	35,118,969,240,805 (31,693,950,345,910–39,006,644,792,386)	34,511,050,582,618 (30,911,743,923,936–38,604,615,131,998)
China	Group I	12,168,411,581,087 (11,299,945,614,304–13,076,636,309,319)	8,449,082,039,426 (7,838,722,766,467–9,156,319,168,806)	5,923,193,466,984 (5,462,447,411,760–6,472,247,723,332)	4,126,087,375,483 (3,730,210,096,602–4,614,454,464,943)	3,233,065,509,422 (2,874,824,685,575–3,681,021,482,130)	2,817,050,322,524 (2,472,244,419,771–3,253,304,046,563)
China	NCD	24,293,396,949,354 (22,059,211,563,499–26,848,358,578,056)	25,271,134,403,506 (22,862,461,650,925–27,819,345,819,951)	26,593,620,657,237 (24,077,257,332,489–29,475,078,394,909)	27,794,451,300,712 (24,984,698,354,672–30,845,117,599,782)	27,659,803,800,720 (24,688,208,445,944–30,925,292,595,563)	28,047,280,088,169 (24,998,725,866,875–31,560,877,702,877)
China	Injuries	6,226,010,841,853 (5,674,603,031,166–6,633,159,037,903)	5,859,897,210,172 (5,400,319,343,516–6,197,984,748,787)	5,221,311,668,905 (4,977,575,086,298–5,457,594,496,727)	4,750,351,787,268 (4,477,803,228,841–4,991,497,731,848)	4,226,099,930,663 (3,918,670,360,288–4,492,084,582,890)	3,646,720,171,925 (3,326,684,136,866–3,936,605,217,973)
North Korea	All causes	669,652,992,544 (512,029,408,466–889,115,499,304)	1,194,883,842,262 (762,997,792,832–1,677,460,797,202)	1,210,993,814,647 (783,810,279,314–1,659,645,932,939)	754,950,005,908 (636,397,909,569–886,706,633,946)	760,339,820,764 (657,189,608,567–881,728,698,394)	779,264,813,167 (657,580,472,446–917,472,446,236)
North Korea	Group I	183,922,426,977 (106,034,930,955–307,912,850,083)	653,473,729,171 (238,279,208,900–1,108,381,175,129)	611,958,277,017 (206,212,158,538–1,046,124,557,530)	117,209,136,900 (78,496,794,366–166,831,024,805)	96,829,094,663 (67,611,377,015–134,759,617,715)	83,802,951,853 (57,492,839,011–120,070,724,624)
North Korea	NCD	387,884,510,858 (313,109,719,970–467,623,975,743)	443,353,067,480 (363,593,532,182–527,272,992,246)	497,932,354,904 (409,423,201,129–594,065,486,497)	538,559,742,782 (456,961,202,025–623,596,698,249)	569,948,854,981 (494,239,620,965–651,904,392,544)	604,200,397,155 (510,138,584,386–709,290,654,474)
North Korea	Injuries	97,846,054,710 (70,200,927,866–137,889,869,172)	98,057,045,611 (73,452,621,081–129,891,647,070)	101,103,182,726 (77,637,411,930–131,522,398,575)	99,181,126,226 (79,768,487,000–123,927,992,062)	93,561,871,119 (76,612,327,127–113,188,133,516)	91,261,464,159 (71,698,824,677–116,164,398,249)
Taiwan	All causes	472,967,611,780 (424,402,577,206–530,123,109,878)	494,869,734,444 (445,317,924,329–550,499,497,058)	507,170,892,115 (453,195,942,701–567,229,075,843)	525,399,766,283 (467,516,458,034–592,174,228,473)	530,195,375,664 (468,359,110,113–599,867,234,100)	572,246,076,188 (492,540,760,073–657,597,295,157)
Taiwan	Group I	51,922,838,287 (41,062,849,954–68,530,628,271)	41,840,619,944 (34,923,337,145–51,365,238,398)	39,065,382,937 (32,859,556,324–46,525,884,851)	35,788,990,467 (28,982,288,125–43,613,382,022)	35,514,629,868 (26,751,969,139–44,124,973,174)	38,716,030,607 (28,609,136,952–48,328,339,542)
Taiwan	NCD	350,245,512,704 (312,137,086,624–393,560,873,905)	383,325,690,321 (342,086,669,867–431,300,652,012)	406,389,276,452 (361,351,333,845–458,332,843,427)	432,194,394,063 (381,490,452,002–490,099,566,100)	443,964,430,326 (387,300,048,378–505,254,626,087)	482,034,832,846 (412,695,932,933–555,896,149,788)
Taiwan	Injuries	70,799,260,788 (64,939,656,177–76,336,894,055)	69,703,424,179 (64,222,913,797–74,593,463,234)	61,716,232,727 (57,831,862,992–66,375,873,276)	57,416,381,754 (54,034,884,372–61,463,793,506)	50,716,315,471 (47,565,922,356–54,395,850,910)	51,495,212,736 (44,538,967,331–59,013,614,040)
Southeast Asia	All causes	19,540,692,558,518 (18,285,966,639,113–20,968,439,383,982)	18,764,158,097,780 (17,440,797,382,503–20,327,657,495,377)	18,680,539,827,000 (17,185,066,197,341–20,293,354,157,511)	18,498,479,422,906 (16,891,052,341,841–20,273,646,837,476)	18,480,536,588,696 (16,762,276,151,222–20,270,175,534,681)	18,669,848,099,400 (16,747,543,169,190–20,751,106,407,244)
Southeast Asia	Group I	9,779,803,464,675 (9,149,839,006,304–10,498,967,039,045)	8,042,483,904,346 (7,526,243,728,610–8,613,445,403,454)	6,884,525,587,829 (6,445,342,739,396–7,435,716,954,851)	5,969,555,722,529 (5,567,336,464,210–6,421,373,162,048)	5,093,599,802,221 (4,720,708,744,632–5,522,823,173,477)	4,346,223,318,533 (3,972,791,444,873–4,779,728,964,820)
Southeast Asia	NCD	7,903,038,052,443 (6,973,937,454,514–8,913,065,728,451)	8,799,145,488,325 (7,742,084,840,651–9,874,873,492,322)	9,820,518,565,872 (8,718,618,052,263–10,956,122,608,173)	10,595,937,036,598 (9,371,390,934,582–11,866,740,517,708)	11,490,854,423,002 (10,140,716,485,620–12,933,949,989,920)	12,519,083,473,382 (10,994,859,319,130–14,136,366,641,875)
Southeast Asia	Injuries	1,857,851,041,400 (1,590,960,677,555–2,076,207,365,853)	1,922,528,705,108 (1,720,096,604,907–2,099,766,293,932)	1,975,495,673,299 (1,815,102,008,930–2,123,549,559,548)	1,932,986,663,779 (1,797,203,364,622–2,072,373,896,280)	1,896,082,363,474 (1,753,176,419,394–2,049,744,726,497)	1,804,541,307,485 (1,637,217,258,346–1,982,757,814,242)
Cambodia	All causes	674,863,049,911 (643,075,675,778–711,860,661,304)	693,686,376,594 (659,590,989,210–731,163,206,934)	651,625,251,059 (616,077,173,926–689,801,336,758)	558,698,719,031 (519,910,659,065–599,777,296,537)	509,008,980,476 (466,920,478,074–556,186,063,295)	479,092,707,506 (424,069,460,993–533,637,176,574)
Cambodia	Group I	426,622,193,183 (392,870,568,551–459,173,145,481)	407,533,450,097 (375,012,856,732–442,237,535,440)	343,450,798,754 (314,404,717,602–372,314,774,293)	245,306,483,414 (223,161,910,951–266,952,359,952)	188,762,817,906 (172,206,071,892–206,075,086,107)	150,858,193,000 (135,452,758,436–168,569,008,585)
Cambodia	NCD	186,914,751,593 (161,838,764,196–217,374,631,081)	215,817,541,955 (188,108,188,686–247,901,275,277)	237,115,454,270 (208,021,036,020–269,405,110,125)	249,918,015,064 (220,440,202,129–281,845,823,143)	259,970,210,313 (227,888,665,022–294,197,386,413)	273,464,698,110 (234,884,222,304–311,073,047,375)
Cambodia	Injuries	61,326,105,135 (45,333,450,862–75,591,540,116)	70,335,384,542 (53,389,260,449–85,462,366,142)	71,058,998,036 (54,939,365,534–84,354,218,355)	63,474,220,554 (52,390,521,417–73,662,320,712)	60,275,952,257 (51,931,421,716–68,526,756,673)	54,769,816,396 (45,716,640,670–63,142,559,792)
Indonesia	All causes	8,181,893,096,254 (7,694,409,495,024–8,729,656,515,851)	7,480,588,082,045 (6,955,539,821,638–8,047,710,567,024)	7,349,849,198,398 (6,826,183,827,065–7,952,212,410,451)	7,363,448,426,486 (6,752,217,740,395–8,041,619,428,508)	7,453,740,921,617 (6,727,002,732,062–8,287,155,711,198)	7,526,924,045,838 (6,513,571,515,763–8,516,122,675,554)
Indonesia	Group I	4,485,221,722,836 (4,150,429,001,236–4,923,053,859,338)	3,525,539,455,018 (3,289,748,606,482–3,844,925,548,447)	2,955,552,602,906 (2,758,223,012,241–3,209,155,638,545)	2,623,146,225,730 (2,441,107,261,517–2,851,224,235,679)	2,321,114,549,850 (2,127,503,314,908–2,552,815,333,775)	2,000,467,828,082 (1,777,753,947,543–2,288,145,679,357)
Indonesia	NCD	3,063,603,913,994 (2,682,519,457,970–3,497,744,023,833)	3,370,837,236,352 (2,961,290,790,296–3,788,655,968,333)	3,801,274,758,276 (3,367,064,400,491–4,249,608,618,982)	4,151,821,981,430 (3,680,160,378,386–4,642,520,136,310)	4,544,261,993,042 (3,985,817,119,984–5,133,821,920,632)	4,949,720,405,366 (4,201,927,599,766–5,699,926,903,108)
Indonesia	Injuries	633,067,459,424 (428,769,258,150–790,439,832,734)	584,211,390,675 (437,930,176,846–685,401,609,380)	593,021,837,216 (496,255,410,236–665,090,737,882)	588,480,219,327 (519,989,800,512–650,018,322,502)	588,364,378,725 (516,861,851,608–676,339,935,366)	576,735,812,390 (475,801,447,564–694,691,226,325)
Laos	All causes	437,137,795,217 (407,698,870,867–466,990,658,644)	425,031,860,788 (396,770,788,794–452,220,346,351)	381,890,683,437 (359,363,397,776–405,817,135,969)	327,077,890,194 (305,749,568,635–350,121,435,281)	282,156,508,236 (259,251,892,381–305,809,009,521)	254,336,030,518 (222,612,269,274–290,640,009,612)
Laos	Group I	290,284,198,254 (260,043,461,723–321,474,540,018)	266,447,729,950 (236,564,488,953–298,038,158,437)	218,761,608,400 (193,408,793,176–248,277,801,631)	167,315,437,271 (147,339,356,756–188,373,031,249)	125,068,218,423 (108,850,104,920–141,965,798,505)	96,842,446,423 (78,997,076,078–119,725,264,414)
Laos	NCD	108,685,862,959 (90,241,076,682–133,699,729,957)	118,929,485,144 (99,898,673,158–143,215,608,073)	123,018,294,323 (104,807,504,458–144,937,387,536)	122,493,382,108 (107,586,923,986–140,789,305,196)	123,153,976,205 (107,544,570,132–140,768,473,238)	126,544,292,591 (108,448,710,847–146,355,067,695)
Laos	Injuries	38,167,734,004 (23,517,941,597–50,871,409,452)	39,654,645,694 (24,841,585,025–53,421,566,099)	40,110,780,715 (25,661,032,394–52,484,742,810)	37,269,070,814 (25,947,510,275–47,499,975,380)	33,934,313,608 (25,756,017,615–41,605,026,340)	30,949,291,503 (24,671,603,250–38,043,569,714)

Appendix Table 3. DALYs for all causes and level 1 cause groups by location for 1990 - 2015, both sexes combined.

	Cause	1990	1995	2000	2005	2010	2015
Malaysia	All causes	433,688,645,744<br>(392,416,298,603–481,781,235,941)	473,951,111,866<br>(425,783,831,054–526,612,974,227)	512,369,694,331<br>(455,985,281,618–572,028,507,136)	551,493,415,011<br>(489,785,433,342–620,407,586,749)	621,906,600,120<br>(552,191,183,639–698,744,574,013)	683,202,116,007<br>(594,392,243,663–778,578,685,709)
Malaysia	Group I	120,314,311,014<br>(106,968,407,812–136,679,994,998)	111,862,960,796<br>(99,815,056,483–126,634,462,204)	107,475,712,808<br>(94,885,860,790–122,078,707,119)	105,157,214,060<br>(89,262,758,753–122,674,506,881)	112,525,394,971<br>(95,648,200,987–131,380,203,706)	111,524,002,820<br>(92,924,860,178–132,464,737,845)
Malaysia	NCD	262,135,580,555<br>(229,821,698,004–296,227,728,441)	302,767,813,588<br>(265,421,301,201–342,618,925,290)	341,239,262,045<br>(298,136,956,938–387,466,897,971)	381,062,269,600<br>(331,049,880,676–434,711,060,037)	436,505,091,380<br>(379,674,645,545–497,412,274,630)	495,390,567,026<br>(424,647,889,750–571,358,128,112)
Malaysia	Injuries	51,238,754,174<br>(46,487,450,825–55,486,399,630)	59,320,337,481<br>(55,073,768,301–63,119,989,516)	63,654,719,478<br>(59,124,026,612–67,555,424,877)	65,273,931,351<br>(60,940,068,924–69,403,652,344)	72,876,113,770<br>(67,226,416,642–78,048,836,448)	76,287,546,161<br>(66,782,915,940–86,072,518,387)
Maldives	All causes	10,460,166,770<br>(9,755,455,833–11,223,613,618)	8,649,819,452<br>(7,948,064,974–9,488,513,779)	7,223,434,003<br>(6,483,009,636–8,083,792,820)	6,326,207,036<br>(5,548,280,155–7,223,244,650)	6,149,053,080<br>(5,298,099,478–7,093,251,741)	6,342,626,439<br>(5,373,619,416–7,383,963,620)
Maldives	Group I	4,562,410,746<br>(3,665,568,668–5,869,470,687)	3,028,473,263<br>(2,560,967,017–3,720,823,543)	1,916,614,257<br>(1,676,131,707–2,190,222,993)	1,487,375,710<br>(1,273,103,380–1,746,643,290)	1,228,451,910<br>(1,042,978,498–1,440,702,763)	1,074,771,597<br>(897,109,463–1,294,409,391)
Maldives	NCD	4,323,347,476<br>(3,647,773,138–5,054,046,618)	4,283,281,911<br>(3,696,901,172–4,896,131,815)	4,168,524,922<br>(3,618,950,513–4,761,879,006)	3,941,675,427<br>(3,356,124,646–4,592,719,566)	4,110,185,504<br>(3,460,655,850–4,819,923,660)	4,508,560,922<br>(3,734,885,027–5,338,444,229)
Maldives	Injuries	1,574,408,548<br>(935,103,585–2,063,350,947)	1,338,064,277<br>(1,005,213,751–1,589,530,584)	1,138,294,825<br>(1,040,835,003–1,236,479,742)	897,155,899<br>(798,382,135–967,740,974)	810,415,666<br>(750,761,315–876,598,431)	759,293,920<br>(657,568,147–865,810,284)
Mauritius	All causes	29,740,924,584<br>(27,292,557,613–32,410,421,749)	31,327,424,146<br>(28,722,581,905–34,157,979,259)	32,463,883,090<br>(29,641,720,804–35,664,871,710)	33,531,646,595<br>(30,536,702,263–36,937,436,910)	35,464,834,092<br>(32,199,672,647–39,242,189,020)	36,405,492,298<br>(32,699,850,656–40,529,280,326)
Mauritius	Group I	5,541,673,600<br>(5,063,965,937–6,288,172,736)	4,748,852,592<br>(4,324,966,598–5,430,130,679)	3,860,388,669<br>(3,516,799,287–4,391,309,111)	3,223,044,225<br>(2,952,101,254–3,602,840,653)	3,124,469,867<br>(2,855,569,269–3,485,067,334)	2,876,378,464<br>(2,624,866,954–3,179,838,108)
Mauritius	NCD	21,539,980,418<br>(19,388,640,972–23,944,069,526)	23,617,600,355<br>(21,231,799,044–26,195,460,952)	25,739,988,809<br>(23,180,801,377–28,561,489,120)	27,563,413,497<br>(24,851,972,808–30,669,159,052)	29,559,676,980<br>(26,630,788,474–33,111,348,061)	30,991,106,507<br>(27,563,574,353–34,773,418,670)
Mauritius	Injuries	2,659,270,566<br>(2,431,015,959–2,822,714,196)	2,960,971,199<br>(2,763,897,140–3,129,500,882)	2,863,505,613<br>(2,663,367,009–3,032,095,784)	2,745,188,873<br>(2,602,161,636–2,889,300,943)	2,780,687,245<br>(2,634,215,582–2,942,475,356)	2,538,007,327<br>(2,353,930,754–2,732,272,335)
Myanmar	All causes	2,569,908,153,944<br>(2,173,303,179,709–3,051,413,310,378)	2,467,045,837,076<br>(2,057,593,043,072–2,942,974,603,575)	2,413,536,367,787<br>(1,991,007,343,765–2,920,928,504,936)	2,232,122,413,055<br>(1,804,290,516,964–2,721,499,926,750)	1,987,785,463,104<br>(1,569,143,469,331–2,539,210,810,475)	1,883,768,605,833<br>(1,477,835,380,137–2,402,190,170,122)
Myanmar	Group I	1,483,663,100,001<br>(1,266,788,487,250–1,708,980,722,076)	1,300,340,266,782<br>(1,109,927,788,577–1,548,882,645,943)	1,176,250,208,383<br>(991,628,256,922–1,442,024,261,865)	959,632,071,415<br>(823,852,111,340–1,171,951,202,987)	688,564,195,848<br>(561,465,133,919–870,763,161,015)	518,150,301,655<br>(413,579,003,372–655,523,833,458)
Myanmar	NCD	911,558,229,154<br>(694,350,211,791–1,163,868,722,102)	984,305,494,192<br>(744,012,679,336–1,246,458,101,174)	1,060,366,943,034<br>(788,140,050,581–1,366,126,224,336)	1,100,585,140,851<br>(818,523,834,590–1,416,478,209,790)	1,132,401,747,752<br>(852,328,381,881–1,501,530,640,122)	1,202,724,568,572<br>(901,470,562,060–1,564,496,319,808)
Myanmar	Injuries	174,686,824,788<br>(131,727,851,569–223,926,578,985)	182,400,076,101<br>(134,144,612,248–236,434,287,335)	176,919,216,370<br>(122,891,377,505–237,786,217,469)	171,905,200,789<br>(117,553,747,227–228,217,455,185)	166,819,519,504<br>(119,350,524,770–226,792,326,008)	162,893,735,607<br>(115,239,090,073–220,318,775,343)
Philippines	All causes	2,449,701,133,175<br>(2,292,953,490,351–2,630,994,691,556)	2,386,585,838,891<br>(2,209,052,662,545–2,588,098,432,628)	2,503,939,025,344<br>(2,306,042,763,958–2,731,918,655,658)	2,681,494,013,784<br>(2,460,217,092,756–2,933,088,405,479)	2,814,676,975,377<br>(2,574,217,265,025–3,082,918,837,729)	2,890,599,189,741<br>(2,613,463,774,567–3,196,749,798,734)
Philippines	Group I	1,279,083,700,956<br>(1,212,450,658,674–1,364,446,196,366)	1,098,006,613,341<br>(1,028,883,202,722–1,184,292,023,220)	995,127,478,526<br>(931,355,125,596–1,068,642,417,801)	951,717,168,649<br>(886,086,051,830–1,027,702,965,994)	863,175,966,507<br>(799,078,733,279–936,014,262,049)	759,276,438,448<br>(693,375,624,277–837,882,759,097)
Philippines	NCD	958,940,041,108<br>(840,943,299,516–1,082,752,924,836)	1,078,990,971,747<br>(946,859,065,173–1,218,967,187,777)	1,256,368,492,665<br>(1,113,467,998,348–1,412,055,387,706)	1,463,874,048,739<br>(1,304,631,605,387–1,640,983,061,729)	1,688,251,346,786<br>(1,502,391,627,670–1,890,797,526,057)	1,867,277,516,671<br>(1,651,285,317,188–2,113,430,021,755)
Philippines	Injuries	211,677,391,111<br>(188,911,843,929–232,646,368,795)	209,588,253,803<br>(188,826,963,029–226,248,831,852)	252,443,054,154<br>(240,006,195,974–265,644,683,603)	265,902,796,396<br>(252,927,759,377–279,192,403,177)	263,249,662,084<br>(248,941,183,044–278,208,084,297)	264,045,234,623<br>(244,133,477,561–287,777,554,584)
Sri Lanka	All causes	572,072,469,156<br>(526,632,531,297–623,214,293,065)	543,048,674,988<br>(496,947,893,427–595,827,826,333)	538,768,600,866<br>(491,225,631,063–593,070,548,151)	521,817,744,243<br>(472,002,265,604–578,705,045,076)	527,601,317,496<br>(468,596,945,007–596,427,578,076)	485,887,760,783<br>(409,056,907,693–578,442,014,081)
Sri Lanka	Group I	174,525,971,759<br>(162,465,110,425–188,781,742,603)	111,927,200,764<br>(102,091,542,945–124,136,149,340)	90,965,717,112<br>(82,661,688,166–101,708,504,320)	86,804,643,696<br>(79,165,434,248–96,258,782,080)	66,980,437,235<br>(59,480,023,814–76,771,207,076)	55,065,526,701<br>(46,522,249,929–64,755,932,955)
Sri Lanka	NCD	273,124,062,528<br>(239,845,523,600–309,644,070,227)	299,098,023,617<br>(262,780,101,022–338,036,364,889)	331,138,365,394<br>(293,952,646,183–372,970,549,913)	349,575,368,714<br>(308,999,264,883–396,134,129,190)	352,227,989,223<br>(308,007,420,448–401,040,136,232)	363,421,054,422<br>(302,960,304,129–434,301,990,227)
Sri Lanka	Injuries	124,422,434,869<br>(116,329,047,841–132,913,602,995)	132,023,450,606<br>(127,655,892,600–136,369,979,676)	116,664,518,360<br>(106,733,108,782–126,123,142,545)	85,437,731,833<br>(81,867,333,004–89,302,994,805)	108,392,891,038<br>(80,354,304,368–136,979,417,129)	67,401,179,660<br>(54,637,376,714–82,056,535,498)
Seychelles	All causes	2,134,108,246<br>(1,969,706,075–2,322,727,869)	2,268,694,561<br>(2,071,493,663–2,469,808,070)	2,379,327,863<br>(2,169,042,748–2,616,407,362)	2,511,725,245<br>(2,282,299,192–2,764,414,527)	2,600,505,418<br>(2,353,743,999–2,887,889,752)	2,623,083,961<br>(2,324,957,025–2,966,220,386)
Seychelles	Group I	423,620,875<br>(380,811,702–468,898,301)	363,746,892<br>(329,590,415–406,510,624)	350,177,577<br>(317,998,886–394,689,477)	372,484,488<br>(339,617,671–414,863,794)	370,453,894<br>(331,507,005–417,137,793)	353,472,757<br>(307,688,876–404,388,506)
Seychelles	NCD	1,467,789,485<br>(1,328,073,973–1,614,866,999)	1,616,758,849<br>(1,456,202,350–1,787,804,354)	1,728,523,094<br>(1,553,626,222–1,922,098,177)	1,831,420,152<br>(1,628,948,694–2,043,901,562)	1,918,859,453<br>(1,707,136,112–2,154,116,541)	2,002,268,741<br>(1,742,595,086–2,276,409,372)
Seychelles	Injuries	242,697,886<br>(225,419,151–261,373,149)	288,188,820<br>(267,368,533–309,064,258)	300,627,192<br>(279,288,860–320,668,960)	307,820,606<br>(280,510,598–331,623,135)	311,192,071<br>(283,958,750–343,551,359)	267,342,463<br>(235,629,025–302,702,461)
Thailand	All causes	1,667,723,552,377<br>(1,509,791,514,273–1,834,524,532,741)	1,854,299,001,594<br>(1,688,486,675,405–2,046,145,076,881)	2,015,169,425,446<br>(1,834,831,732,051–2,226,590,214,700)	1,954,146,708,509<br>(1,771,894,895,956–2,160,253,421,300)	1,957,865,996,113<br>(1,756,342,654,367–2,167,008,113,673)	2,062,070,814,713<br>(1,795,926,535,050–2,329,919,930,956)
Thailand	Group I	426,270,511,862<br>(382,251,748,216–482,017,819,188)	356,017,702,832<br>(311,923,507,904–409,420,950,056)	366,050,973,499<br>(333,951,882,445–401,844,275,397)	311,193,583,784<br>(276,854,918,732–354,971,542,695)	294,395,398,438<br>(251,669,300,683–332,304,437,620)	285,774,984,668<br>(242,931,630,691–327,048,116,272)
Thailand	NCD	973,837,065,263<br>(857,824,404,035–1,100,182,090,607)	1,151,183,406,208<br>(1,020,974,790,815–1,302,584,899,167)	1,291,893,806,908<br>(1,149,400,388,518–1,457,698,186,647)	1,291,733,716,243<br>(1,130,798,076,034–1,464,200,229,032)	1,364,419,586,291<br>(1,186,461,867,000–1,541,931,837,708)	1,501,999,067,027<br>(1,284,148,912,254–1,718,849,777,053)
Thailand	Injuries	267,615,975,252<br>(249,085,133,829–285,528,424,677)	347,097,892,553<br>(322,598,960,720–374,495,561,930)	357,224,645,039<br>(325,234,779,751–389,417,830,644)	351,219,408,482<br>(323,729,424,266–381,783,172,299)	299,051,011,384<br>(276,261,238,648–323,511,470,728)	274,296,763,018<br>(236,106,562,144–317,896,095,861)
Timor-Leste	All causes	59,807,820,353<br>(55,451,813,114–64,337,072,702)	58,111,111,097<br>(53,412,365,029–63,018,411,932)	58,654,156,064<br>(51,838,047,357–65,748,962,613)	42,541,326,865<br>(38,812,435,277–46,677,073,104)	40,119,108,180<br>(34,574,564,761–46,410,171,003)	36,721,219,467<br>(29,918,960,508–45,657,466,138)
Timor-Leste	Group I	42,551,793,695<br>(37,378,014,276–48,543,622,395)	39,409,469,387<br>(34,020,010,232–45,630,756,632)	34,979,491,903<br>(29,726,056,765–41,058,824,329)	24,202,246,914<br>(20,593,160,101–28,488,850,904)	19,798,197,463<br>(15,834,280,612–24,687,107,030)	15,966,784,652<br>(11,461,144,920–22,145,897,384)
Timor-Leste	NCD	12,966,161,497<br>(10,004,496,338–16,469,726,749)	14,326,847,671<br>(11,363,924,057–17,754,555,612)	13,215,894,217<br>(9,990,312,139–16,861,751,797)	14,427,898,123<br>(11,727,956,408–17,273,814,650)	16,490,770,828<br>(13,684,097,254–19,462,428,261)	17,252,350,876<br>(14,257,874,332–20,453,470,616)
Timor-Leste	Injuries	4,289,865,160<br>(2,183,867,901–6,381,724,669)	4,374,794,038<br>(2,345,567,554–6,353,473,732)	10,458,769,944<br>(5,472,616,733–15,768,122,904)	3,911,181,828<br>(2,519,269,668–5,133,342,861)	3,830,139,890<br>(2,683,328,121–4,990,847,184)	3,502,083,939<br>(2,582,019,507–4,610,884,795)
Vietnam	All causes	2,425,300,764,805<br>(2,203,164,940,615–2,679,336,093,949)	2,313,736,029,117<br>(2,044,386,216,819–2,600,936,766,181)	2,185,840,160,771<br>(1,915,482,094,928–2,466,605,982,233)	2,195,690,039,847<br>(1,858,980,584,183–2,528,135,469,488)	2,213,776,727,723<br>(1,856,877,330,114–2,612,875,121,079)	2,293,215,249,383<br>(1,906,396,519,929–2,696,726,481,298)
Vietnam	Group I	1,028,485,238,320<br>(887,963,929,081–1,223,167,149,339)	807,159,880,815<br>(687,970,177,777–959,871,474,918)	580,619,022,043<br>(494,783,439,823–690,086,927,913)	481,725,996,623<br>(407,501,733,067–600,505,481,875)	401,824,109,927<br>(337,196,750,136–511,097,721,814)	342,320,348,966<br>(282,393,140,306–451,230,593,944)
Vietnam	NCD	1,112,397,702,132<br>(950,157,333,766–1,285,462,390,149)	1,220,218,327,602<br>(1,034,585,961,198–1,415,542,709,828)	1,318,253,896,624<br>(1,135,033,810,808–1,506,857,279,778)	1,420,437,272,290<br>(1,180,079,038,068–1,652,632,073,939)	1,519,087,110,466<br>(1,259,026,760,535–1,803,334,294,247)	1,663,184,869,559<br>(1,367,925,365,054–1,970,474,399,454)
Vietnam	Injuries	284,417,824,353<br>(200,138,711,506–357,728,125,799)	286,357,820,701<br>(216,458,104,501–351,906,835,325)	286,967,242,104<br>(234,489,944,146–339,786,865,458)	293,526,770,933<br>(229,343,235,168–360,805,332,046)	292,865,507,331<br>(224,437,436,141–384,796,203,521)	287,710,030,858<br>(218,855,879,706–371,090,499,912)
Oceania	All causes	365,219,664,141<br>(318,200,933,126–420,868,432,717)	394,814,829,692<br>(339,626,663,856–459,475,150,055)	428,819,687,972<br>(360,046,920,995–508,315,696,635)	463,384,661,546<br>(380,128,720,815–563,973,218,641)	478,350,079,239<br>(388,327,524,486–598,521,569,620)	485,043,025,496<br>(392,451,524,597–608,381,969,879)
Oceania	Group I	172,279,484,183<br>(151,971,278,129–195,511,631,661)	175,765,174,066<br>(152,370,017,047–204,331,964,891)	176,782,869,147<br>(145,619,712,625–212,566,810,702)	178,362,263,697<br>(143,516,014,052–220,404,763,157)	164,867,905,175<br>(131,183,917,314–212,544,692,399)	148,710,390,265<br>(117,009,313,509–194,379,783,454)
Oceania	NCD	154,681,768,260<br>(127,934,426,864–187,107,260,401)	177,027,966,544<br>(145,281,599,698–214,196,136,887)	204,977,672,801<br>(166,350,260,204–248,472,275,191)	234,038,872,238<br>(186,170,769,966–289,467,422,432)	260,958,198,082<br>(208,839,411,797–332,604,878,268)	283,276,200,065<br>(227,556,138,714–358,183,178,327)

Appendix Table 3. DALYs for all causes and level 1 cause groups by location for 1990 - 2015, both sexes combined.

	Cause	1990	1995	2000	2005	2010	2015
Oceania	Injuries	38,258,411,697 (29,563,961,013–49,190,965,925)	42,021,689,082 (32,131,460,800–54,667,205,821)	47,059,146,025 (35,915,504,384–62,145,276,619)	50,983,525,612 (37,784,291,319–67,200,926,355)	52,523,975,982 (38,948,723,265–71,514,780,432)	53,056,435,166 (40,188,934,251–71,478,199,264)
American Samoa	All causes	1,304,686,515 (1,169,239,134–1,455,641,925)	1,394,089,405 (1,238,553,818–1,565,810,652)	1,419,193,706 (1,253,662,955–1,592,734,902)	1,548,221,572 (1,372,235,690–1,746,566,978)	1,660,909,745 (1,448,316,588–1,893,765,003)	1,858,613,593 (1,577,649,906–2,135,428,107)
American Samoa	Group I	317,066,728 (281,240,008–361,809,140)	298,279,853 (265,730,959–337,886,671)	255,789,164 (227,226,326–288,020,362)	248,336,809 (221,073,452–282,041,531)	238,032,309 (207,491,799–274,778,663)	245,241,706 (205,977,129–288,520,496)
American Samoa	NCD	822,166,608 (716,379,033–935,459,658)	923,619,431 (801,412,500–1,051,891,413)	996,462,495 (866,599,326–1,132,869,221)	1,121,182,769 (982,342,013–1,279,762,403)	1,243,590,941 (1,076,428,987–1,432,036,654)	1,425,860,932 (1,198,569,503–1,649,802,799)
American Samoa	Injuries	165,453,179 (145,071,147–187,048,506)	172,190,120 (150,616,903–195,223,825)	166,942,047 (151,252,145–184,654,806)	178,701,994 (161,874,129–197,210,052)	179,286,495 (161,204,813–200,531,201)	187,510,955 (160,075,394–216,971,598)
Federated States of Micronesia	All causes	3,651,819,500 (2,957,992,158–4,474,763,837)	3,715,212,459 (2,966,844,308–4,615,742,924)	3,409,318,688 (2,686,786,340–4,361,213,184)	3,159,786,583 (2,463,005,185–4,199,518,362)	2,971,879,355 (2,320,474,363–4,023,601,086)	2,950,121,739 (2,288,270,807–4,082,100,825)
Federated States of Micronesia	Group I	1,275,610,957 (1,039,817,041–1,544,924,476)	1,134,977,698 (902,632,449–1,421,943,906)	872,580,038 (684,723,224–1,141,271,406)	633,176,730 (489,129,586–842,986,575)	501,585,045 (386,041,406–651,554,564)	437,606,331 (343,262,972–569,174,815)
Federated States of Micronesia	NCD	1,960,445,872 (1,486,778,048–2,511,364,025)	2,163,722,959 (1,647,928,382–2,810,863,286)	2,146,998,368 (1,660,675,910–2,881,413,418)	2,165,128,810 (1,650,104,643–2,963,910,547)	2,137,698,551 (1,646,904,645–2,962,221,588)	2,184,595,168 (1,668,865,232–3,058,008,117)
Federated States of Micronesia	Injuries	415,762,671 (297,246,881–554,645,563)	416,511,803 (293,548,376–567,423,478)	389,740,283 (277,669,483–531,274,948)	361,481,043 (257,164,570–514,309,182)	332,595,758 (242,277,534–489,588,622)	327,920,239 (236,328,876–492,187,085)
Fiji	All causes	25,729,096,930 (22,413,161,087–29,360,089,660)	28,472,148,358 (25,012,914,155–32,109,956,755)	31,298,581,741 (27,933,509,768–34,818,339,398)	33,034,805,065 (29,621,533,311–36,582,303,140)	34,387,043,039 (30,492,365,690–38,521,604,243)	35,887,960,687 (31,221,005,316–41,025,020,962)
Fiji	Group I	7,076,752,180 (5,752,235,764–8,748,027,726)	7,032,653,771 (5,781,374,783–8,645,189,789)	7,345,108,977 (6,168,546,728–8,693,760,777)	7,083,971,966 (5,933,549,264–8,424,762,892)	6,732,591,996 (5,501,579,677–8,276,368,314)	6,353,837,998 (4,993,229,060–8,268,619,701)
Fiji	NCD	16,242,857,200 (14,254,121,752–18,679,217,863)	18,787,156,146 (16,494,570,498–21,317,097,854)	20,938,073,423 (18,627,581,664–23,310,914,702)	22,741,628,727 (20,349,054,637–25,217,581,468)	24,676,004,560 (22,041,036,976–27,646,899,768)	26,447,869,922 (22,981,389,974–30,303,186,615)
Fiji	Injuries	2,409,487,550 (2,025,324,682–2,814,458,881)	2,652,338,440 (2,324,993,152–3,002,768,190)	3,015,399,341 (2,706,567,448–3,308,659,818)	3,209,204,378 (2,867,021,450–3,578,876,068)	2,978,446,483 (2,619,128,911–3,397,414,204)	3,086,252,766 (2,605,677,979–3,628,326,884)
Guam	All causes	3,170,339,791 (2,829,425,832–3,541,980,149)	3,523,054,436 (3,138,336,579–3,958,242,893)	3,796,597,310 (3,357,419,709–4,292,732,716)	4,248,984,907 (3,748,008,885–4,771,794,454)	4,952,390,219 (4,372,952,448–5,546,982,611)	5,502,983,348 (4,818,006,536–6,243,739,163)
Guam	Group I	515,131,209 (450,418,351–591,763,434)	496,510,077 (438,447,133–567,268,322)	510,629,789 (450,169,220–583,074,470)	580,393,465 (488,447,348–713,672,062)	643,089,773 (533,590,318–818,725,764)	645,744,210 (523,147,114–801,203,499)
Guam	NCD	2,224,549,713 (1,957,249,504–2,522,673,488)	2,554,126,837 (2,228,339,033–2,907,900,872)	2,855,968,508 (2,500,054,070–3,245,081,881)	3,215,107,479 (2,819,366,283–3,612,068,490)	3,798,620,568 (3,352,339,402–4,261,985,589)	4,307,138,713 (3,757,928,988–4,905,076,829)
Guam	Injuries	430,658,869 (381,537,100–475,982,380)	472,417,522 (420,343,200–524,688,011)	429,999,013 (380,177,655–473,597,041)	453,483,963 (405,536,519–501,871,890)	510,679,878 (463,286,599–565,606,875)	550,100,425 (468,612,508–632,847,205)
Kiribati	All causes	4,455,520,990 (3,968,198,541–4,954,208,722)	4,370,783,606 (3,955,982,005–4,796,438,683)	4,393,578,613 (4,007,939,779–4,822,443,020)	4,592,182,612 (4,092,352,440–5,135,764,583)	4,735,011,385 (4,060,049,282–5,530,293,904)	4,895,602,194 (4,156,170,106–5,737,922,641)
Kiribati	Group I	2,325,211,169 (1,972,303,244–2,710,334,279)	2,102,288,632 (1,846,702,550–2,383,780,583)	1,856,557,117 (1,641,125,019–2,096,681,986)	1,823,717,782 (1,537,352,908–2,163,550,337)	1,703,211,154 (1,315,671,308–2,180,351,563)	1,571,476,333 (1,152,888,210–2,161,725,230)
Kiribati	NCD	1,795,281,191 (1,584,248,150–2,010,753,113)	1,921,317,245 (1,713,152,082–2,138,889,922)	2,156,146,035 (1,928,333,067–2,398,862,153)	2,342,337,323 (2,069,269,868–2,614,883,993)	2,564,534,609 (2,237,337,284–2,911,655,512)	2,838,392,379 (2,440,885,131–3,235,693,396)
Kiribati	Injuries	335,028,630 (282,925,550–387,097,896)	347,177,729 (300,577,437–393,473,362)	380,875,461 (326,361,524–430,001,362)	426,127,507 (347,023,549–492,282,369)	467,265,622 (364,676,847–562,153,603)	485,733,482 (373,014,110–594,441,142)
Marshall Islands	All causes	1,920,146,438 (1,663,762,051–2,196,189,429)	1,862,258,715 (1,644,351,419–2,084,685,526)	2,132,649,662 (1,834,466,078–2,479,091,953)	2,309,999,362 (2,016,338,247–2,594,939,389)	2,374,191,660 (2,055,369,813–2,742,803,748)	2,426,300,035 (2,103,464,739–2,783,748,746)
Marshall Islands	Group I	767,656,630 (610,977,868–940,164,671)	640,043,077 (513,446,735–817,921,359)	723,851,566 (548,774,384–968,642,277)	674,396,142 (530,516,418–870,666,889)	574,970,823 (428,679,078–772,087,074)	491,466,933 (364,802,976–661,021,446)
Marshall Islands	NCD	938,313,533 (815,132,109–1,072,089,015)	1,016,291,366 (880,762,809–1,149,971,349)	1,170,212,727 (998,559,141–1,337,913,138)	1,377,513,511 (1,187,594,419–1,553,278,142)	1,542,850,841 (1,334,111,967–1,754,441,699)	1,682,898,160 (1,446,116,974–1,917,663,749)
Marshall Islands	Injuries	214,176,275 (174,818,899–256,396,080)	205,924,271 (163,780,284–238,194,612)	238,585,369 (185,976,921–289,421,394)	258,089,709 (207,548,917–307,164,717)	256,369,996 (210,284,367–303,625,358)	251,934,941 (208,732,260–296,798,828)
Northern Mariana Islands	All causes	962,591,787 (829,465,701–1,121,646,751)	1,163,726,845 (993,459,565–1,339,108,821)	1,254,539,558 (1,075,325,525–1,443,153,952)	1,461,039,420 (1,259,405,160–1,681,939,242)	1,708,511,872 (1,473,499,675–1,979,954,890)	2,060,375,634 (1,768,879,307–2,400,916,988)
Northern Mariana Islands	Group I	177,248,114 (128,207,815–251,669,757)	190,184,502 (145,892,525–251,341,017)	173,416,712 (144,853,474–207,509,420)	179,244,824 (151,723,373–213,435,589)	184,724,529 (155,255,930–221,306,109)	221,949,255 (180,817,540–273,899,942)
Northern Mariana Islands	NCD	636,340,045 (543,173,699–744,047,495)	810,467,341 (685,570,142–947,219,541)	910,363,832 (764,309,816–1,058,635,770)	1,072,076,019 (902,914,942–1,258,155,003)	1,286,703,423 (1,086,465,969–1,520,858,172)	1,555,893,897 (1,312,189,502–1,834,298,146)
Northern Mariana Islands	Injuries	149,003,628 (126,482,164–173,376,116)	163,075,002 (143,782,519–185,213,391)	170,759,014 (153,836,827–191,101,425)	209,718,578 (189,642,589–230,768,143)	237,083,920 (210,067,777–265,482,734)	282,532,482 (242,883,605–330,870,197)
Papua New Guinea	All causes	265,470,823,861 (226,089,626,190–315,342,652,295)	288,812,782,457 (240,660,263,571–346,295,509,990)	315,415,997,902 (254,205,499,678–386,578,269,035)	343,113,029,969 (268,085,698,799–434,335,688,305)	354,115,008,919 (273,682,623,041–461,678,200,634)	355,400,726,868 (273,392,230,043–469,142,560,157)
Papua New Guinea	Group I	136,742,222,459 (118,401,985,618–157,090,304,724)	141,921,548,205 (120,444,770,485–168,470,317,328)	144,035,926,294 (115,406,983,817–176,795,063,871)	146,811,149,902 (114,720,858,489–185,154,029,957)	137,043,026,931 (104,883,277,825–182,265,472,180)	122,796,421,147 (93,931,551,914–165,962,637,392)
Papua New Guinea	NCD	100,860,545,262 (78,127,568,558–127,541,661,039)	115,987,638,938 (88,839,685,672–147,539,687,810)	136,421,713,790 (103,673,455,487–174,081,903,747)	157,967,162,303 (118,125,805,597–206,965,522,403)	177,003,895,554 (131,767,934,995–240,327,505,199)	192,268,433,766 (145,058,215,611–259,827,231,035)
Papua New Guinea	Injuries	27,868,056,140 (20,019,249,942–37,581,936,255)	30,903,595,314 (21,818,761,047–42,417,401,004)	34,958,357,818 (24,583,471,960–48,768,408,712)	38,334,717,764 (26,236,023,306–53,118,149,144)	40,068,086,434 (27,870,261,375–57,979,607,043)	40,335,871,955 (28,648,587,084–58,029,543,705)
Samoa	All causes	4,644,319,385 (3,919,925,222–5,534,482,365)	4,571,427,495 (3,839,526,336–5,427,560,072)	4,510,887,044 (3,801,026,204–5,312,298,610)	4,520,625,404 (3,836,766,426–5,273,190,721)	4,520,013,421 (3,877,919,841–5,236,089,102)	4,615,603,754 (3,884,290,009–5,400,166,647)
Samoa	Group I	1,223,388,219 (865,822,582–1,794,901,692)	1,081,603,072 (773,474,164–1,615,848,732)	969,104,511 (690,803,457–1,484,934,042)	874,192,743 (630,064,223–1,289,251,532)	768,358,809 (577,248,040–1,096,802,035)	692,872,720 (525,368,685–971,573,517)
Samoa	NCD	2,868,124,213 (2,410,171,291–3,445,988,729)	2,970,215,759 (2,492,551,132–3,475,478,071)	3,046,277,437 (2,559,011,185–3,560,131,232)	3,175,933,765 (2,688,126,460–3,655,418,513)	3,289,910,637 (2,809,075,253–3,806,391,354)	3,473,633,149 (2,908,377,586–4,034,185,592)
Samoa	Injuries	552,806,954 (435,072,503–686,742,043)	519,608,664 (395,317,613–629,341,036)	495,505,096 (383,073,263–622,984,265)	470,498,896 (375,766,668–570,650,670)	461,743,975 (381,253,166–558,829,468)	449,097,885 (363,611,905–548,203,082)
Solomon Islands	All causes	14,612,237,876 (11,815,518,879–17,867,766,617)	15,384,913,028 (12,292,080,111–19,136,779,340)	16,941,057,713 (13,483,238,047–21,159,952,031)	19,210,563,998 (14,882,727,075–24,135,814,300)	20,191,345,311 (15,430,967,953–26,117,655,738)	20,765,725,302 (15,854,729,400–27,488,491,140)
Solomon Islands	Group I	6,042,998,704 (4,861,261,139–7,345,687,145)	5,692,407,219 (4,551,027,733–7,129,909,414)	5,539,916,061 (4,464,348,852–6,948,634,992)	5,890,920,048 (4,649,385,051–7,428,298,276)	5,325,250,586 (4,122,344,633–6,714,672,100)	4,581,947,179 (3,525,577,396–5,850,790,211)
Solomon Islands	NCD	6,989,553,769 (5,311,730,756–9,069,988,254)	7,956,014,838 (5,999,354,145–10,415,376,900)	9,357,016,343 (7,005,737,533–12,271,467,877)	11,089,972,261 (8,128,876,922–14,540,631,192)	12,501,900,967 (9,180,859,496–16,963,125,187)	13,757,667,413 (10,092,666,888–18,915,443,667)
Solomon Islands	Injuries	1,579,685,403 (1,121,877,796–2,120,757,377)	1,736,490,970 (1,235,215,432–2,339,196,126)	2,044,125,309 (1,437,186,523–2,765,192,019)	2,229,671,689 (1,524,269,809–3,097,545,940)	2,364,193,758 (1,668,807,722–3,309,592,066)	2,426,110,710 (1,749,001,339–3,498,571,357)
Tonga	All causes	2,945,258,496 (2,528,826,537–3,458,679,963)	2,931,621,553 (2,558,346,146–3,357,179,619)	3,016,069,762 (2,638,963,332–3,430,830,892)	3,065,333,128 (2,683,854,842–3,489,445,110)	3,033,525,134 (2,616,640,538–3,481,978,661)	2,947,796,325 (2,523,429,504–3,398,155,345)
Tonga	Group I	878,470,499 (667,071,592–1,189,888,264)	772,071,020 (596,525,812–998,344,219)	757,178,234 (582,711,911–964,202,366)	714,616,972 (556,026,962–911,747,269)	668,185,888 (520,510,528–861,085,783)	575,459,528 (447,658,000–752,880,525)

Appendix Table 3. DALYs for all causes and level 1 cause groups by location for 1990 - 2015, both sexes combined.

	Cause	1990	1995	2000	2005	2010	2015
Tonga	NCD	1,683,167,696<br>(1,456,637,900–1,934,026,707)	1,784,917,466<br>(1,575,403,028–2,024,518,641)	1,883,265,074<br>(1,667,213,506–2,121,096,411)	1,973,778,890<br>(1,737,585,494–2,227,548,648)	2,001,564,170<br>(1,729,861,824–2,287,047,337)	2,030,982,995<br>(1,736,757,085–2,358,560,450)
Tonga	Injuries	383,620,300<br>(317,806,685–455,765,768)	374,633,067<br>(321,789,525–439,613,242)	375,626,454<br>(330,234,099–429,496,327)	376,937,266<br>(330,701,767–432,786,008)	363,775,076<br>(312,074,392–430,320,396)	341,353,803<br>(286,430,857–408,144,979)
Vanuatu	All causes	6,221,038,524<br>(5,118,601,247–7,719,835,389)	6,894,749,716<br>(5,458,141,973–8,694,223,816)	7,540,340,971<br>(5,965,271,221–9,454,135,475)	8,445,765,126<br>(6,657,693,344–10,708,279,013)	9,000,795,836<br>(6,990,598,912–11,917,546,833)	9,704,625,498<br>(7,630,433,081–12,623,062,473)
Vanuatu	Group I	2,290,218,103<br>(1,867,385,425–2,781,589,516)	2,340,327,341<br>(1,915,805,952–2,906,374,435)	2,439,529,465<br>(2,012,812,949–3,108,808,260)	2,619,896,707<br>(2,118,274,643–3,277,680,997)	2,366,570,220<br>(1,883,920,418–3,034,486,869)	2,193,448,181<br>(1,722,211,339–2,827,721,393)
Vanuatu	NCD	3,283,345,478<br>(2,541,689,751–4,264,380,373)	3,808,718,124<br>(2,831,390,882–5,138,701,799)	4,222,874,608<br>(3,057,194,906–5,514,285,460)	4,857,112,304<br>(3,558,363,553–6,502,929,325)	5,600,602,391<br>(4,122,346,996–7,698,193,977)	6,372,532,091<br>(4,784,041,978–8,611,230,392)
Vanuatu	Injuries	647,474,943<br>(454,387,561–901,565,569)	745,704,251<br>(488,185,188–1,043,344,620)	877,936,898<br>(600,373,685–1,207,085,129)	968,756,114<br>(657,713,781–1,338,112,892)	1,033,623,225<br>(732,320,538–1,462,734,168)	1,138,645,227<br>(861,407,929–1,519,556,385)
North Africa and Middle East	All causes	15,771,061,378,803<br>(14,840,435,647,930–16,780,281,117,606)	15,050,922,966,231<br>(14,004,600,741,830–16,144,356,728,674)	14,558,423,150,877<br>(13,379,809,557,090–15,793,116,352,957)	14,811,490,814,082<br>(13,526,444,843,446–16,197,008,735,001)	15,194,750,062,863<br>(13,729,200,985,037–16,690,884,377,501)	16,565,717,102,355<br>(14,745,642,512,766–18,374,047,034,872)
North Africa and Middle East	Group I	6,764,757,118,732<br>(6,054,381,406,375–7,283,013,090,860)	5,759,624,107,961<br>(5,202,243,302,912–6,210,784,098,006)	4,897,069,316,838<br>(4,474,063,389,611–5,281,168,975,204)	4,267,857,532,644<br>(3,890,182,960,972–4,636,670,541,825)	3,843,332,696,614<br>(3,485,307,791,117–4,189,508,445,838)	3,399,729,449,472<br>(3,073,332,722,848–3,775,367,232,554)
North Africa and Middle East	NCD	6,823,506,454,793<br>(6,044,700,987,570–7,719,840,493,630)	7,352,832,076,499<br>(6,493,053,859,644–8,230,924,695,020)	7,762,528,260,324<br>(6,833,466,975,193–8,713,878,536,592)	8,513,154,362,368<br>(7,463,492,491,869–9,556,665,241,447)	9,340,073,083,139<br>(8,137,284,337,546–10,513,765,739,203)	10,282,792,771,222<br>(8,975,072,325,813–11,610,660,609,762)
North Africa and Middle East	Injuries	2,182,797,805,279<br>(1,882,540,576,004–2,443,954,406,927)	1,938,466,781,771<br>(1,726,331,423,307–2,125,682,288,132)	1,898,825,573,715<br>(1,725,500,860,727–2,061,293,667,858)	2,030,478,919,069<br>(1,850,632,835,185–2,205,348,815,028)	2,011,344,283,110<br>(1,846,689,208,209–2,182,432,622,578)	2,883,194,881,661<br>(2,393,618,696,603–3,364,925,267,812)
Afghanistan	All causes	1,344,592,896,937<br>(1,237,205,007,525–1,451,943,684,936)	1,699,334,849,408<br>(1,562,264,543,638–1,835,857,973,695)	1,923,529,242,780<br>(1,775,743,134,374–2,091,625,497,900)	2,017,921,927,410<br>(1,825,370,501,110–2,220,263,611,620)	1,994,814,045,801<br>(1,782,084,584,793–2,222,757,988,468)	2,169,145,897,101<br>(1,890,789,566,113–2,472,361,927,739)
Afghanistan	Group I	808,333,125,464<br>(666,738,780,430–947,417,227,220)	950,053,119,514<br>(796,501,647,010–1,109,058,725,260)	1,056,748,954,499<br>(879,995,833,004–1,221,952,905,568)	982,369,705,695<br>(818,217,252,250–1,103,575,711,786)	871,054,616,015<br>(722,127,345,091–1,008,775,573,123)	737,030,347,343<br>(607,736,695,193–875,131,904,629)
Afghanistan	NCD	328,182,545,897<br>(244,590,362,505–447,258,252,188)	458,594,626,911<br>(350,444,088,664–610,245,895,261)	541,984,272,476<br>(421,471,262,030–711,452,867,741)	662,379,887,028<br>(533,947,413,531–841,594,555,926)	723,115,494,086<br>(580,406,974,810–898,489,116,811)	819,773,171,392<br>(663,768,166,598–994,484,715,334)
Afghanistan	Injuries	208,077,225,576<br>(140,685,950,277–266,570,501,855)	290,687,102,984<br>(209,340,325,699–365,440,488,631)	324,796,015,805<br>(238,960,345,621–404,863,221,246)	373,172,334,687<br>(308,409,039,730–444,287,555,598)	400,643,935,700<br>(331,610,433,929–481,784,366,962)	612,342,378,366<br>(450,463,176,667–783,979,517,688)
Algeria	All causes	890,735,554,030<br>(816,120,831,997–970,031,961,698)	840,508,808,470<br>(762,077,507,345–929,465,085,797)	789,321,188,576<br>(706,015,647,806–882,880,389,116)	811,531,977,112<br>(717,715,908,647–915,844,117,946)	880,579,016,501<br>(774,482,581,591–991,244,502,634)	952,793,617,829<br>(827,277,888,042–1,081,222,187,916)
Algeria	Group I	353,855,732,512<br>(312,593,804,151–394,351,738,591)	268,121,916,561<br>(238,477,413,758–300,019,369,120)	203,526,750,588<br>(182,172,590,824–226,018,100,728)	190,764,692,493<br>(167,533,576,450–216,281,671,335)	202,847,053,024<br>(174,052,740,539–233,642,284,312)	194,171,186,955<br>(163,553,270,954–231,868,707,700)
Algeria	NCD	424,180,938,444<br>(371,074,835,360–484,293,760,730)	447,611,864,294<br>(389,748,759,001–514,180,507,710)	480,646,067,245<br>(415,804,209,705–553,388,072,490)	527,451,040,109<br>(453,663,034,313–609,687,085,974)	585,421,264,132<br>(502,909,530,781–674,591,185,779)	661,537,162,551<br>(565,400,529,811–762,128,687,422)
Algeria	Injuries	112,698,883,074<br>(91,745,868,687–131,383,517,401)	124,775,027,615<br>(105,069,927,737–146,014,853,109)	105,148,370,742<br>(94,621,508,606–115,769,744,528)	93,316,244,510<br>(83,601,281,912–103,272,193,457)	92,310,699,345<br>(83,103,225,300–101,689,086,791)	97,085,268,323<br>(85,829,226,018–108,614,908,021)
Bahrain	All causes	12,541,514,967<br>(11,113,606,628–14,072,260,762)	13,332,307,125<br>(11,657,087,226–15,079,380,967)	14,821,550,676<br>(12,816,552,650–16,859,759,503)	16,541,801,246<br>(14,171,136,299–19,175,542,393)	22,024,397,590<br>(18,550,769,393–25,946,135,701)	23,742,699,116<br>(19,720,277,072–28,067,934,117)
Bahrain	Group I	2,523,295,295<br>(2,269,521,938–2,860,975,299)	2,135,586,949<br>(1,899,338,062–2,454,663,291)	1,904,776,574<br>(1,657,704,469–2,206,194,295)	2,030,283,718<br>(1,740,346,987–2,390,022,523)	2,312,136,445<br>(1,918,002,581–2,772,159,179)	2,331,692,943<br>(1,914,474,838–2,833,276,022)
Bahrain	NCD	8,294,230,692<br>(7,201,558,161–9,491,095,799)	9,206,660,667<br>(7,933,615,915–10,542,709,832)	10,247,548,060<br>(8,754,157,731–11,800,633,557)	12,367,438,252<br>(10,514,872,352–14,491,923,210)	17,282,164,960<br>(14,280,830,007–20,521,691,033)	18,938,341,067<br>(15,521,239,771–22,605,465,138)
Bahrain	Injuries	1,723,988,981<br>(1,533,335,124–1,938,776,379)	1,990,059,510<br>(1,749,911,845–2,253,458,128)	2,669,226,042<br>(2,131,628,585–3,206,407,351)	2,144,079,276<br>(1,861,234,089–2,469,322,643)	2,430,096,185<br>(2,134,545,813–2,781,360,583)	2,472,665,105<br>(2,058,559,814–2,960,309,694)
Egypt	All causes	2,788,394,831,203<br>(2,611,105,761,772–2,973,030,583,210)	2,431,816,409,797<br>(2,251,038,506,445–2,624,782,714,202)	2,206,008,368,982<br>(2,018,215,102,445–2,410,328,866,813)	2,302,154,843,577<br>(2,091,655,526,084–2,535,521,972,242)	2,547,607,117,575<br>(2,316,369,954,100–2,805,911,236,751)	2,614,594,267,494<br>(2,307,969,746,661–2,926,793,442,284)
Egypt	Group I	1,254,892,997,819<br>(1,089,282,234,101–1,353,552,041,186)	877,140,899,520<br>(785,796,777,463–955,311,176,691)	613,446,934,307<br>(561,036,180,748–671,696,087,170)	530,092,390,170<br>(473,316,555,097–595,931,521,260)	516,560,142,485<br>(457,073,962,450–583,640,859,605)	459,468,139,929<br>(374,779,912,966–566,592,643,983)
Egypt	NCD	1,379,314,189,106<br>(1,242,874,600,456–1,546,780,526,515)	1,393,685,585,509<br>(1,264,801,945,041–1,536,960,066,741)	1,428,335,498,043<br>(1,282,982,840,211–1,580,523,390,182)	1,608,243,962,487<br>(1,447,397,691,680–1,783,280,053,009)	1,854,105,060,666<br>(1,675,550,314,888–2,054,141,666,883)	1,960,676,081,914<br>(1,742,560,496,578–2,181,379,202,947)
Egypt	Injuries	154,187,644,278<br>(134,657,679,113–181,255,664,901)	160,989,924,768<br>(145,759,032,423–177,927,485,423)	164,225,936,632<br>(151,822,008,342–176,846,889,258)	163,818,490,920<br>(150,888,847,158–176,673,406,295)	176,941,914,425<br>(164,284,322,124–190,865,896,425)	194,450,045,651<br>(174,563,534,067–214,102,845,386)
Iran	All causes	2,591,769,861,658<br>(2,325,041,175,290–2,854,638,475,626)	1,912,397,584,871<br>(1,689,613,491,532–2,135,410,883,298)	1,802,559,605,016<br>(1,596,699,080,626–2,028,204,940,972)	1,894,999,981,043<br>(1,643,832,991,665–2,173,884,949,723)	1,868,430,619,240<br>(1,590,228,251,613–2,164,475,137,303)	1,918,230,400,086<br>(1,621,939,742,422–2,232,680,862,759)
Iran	Group I	781,687,583,440<br>(633,356,441,037–970,146,340,206)	451,396,090,526<br>(371,378,043,928–569,616,750,102)	306,264,977,247<br>(250,988,783,344–377,136,782,782)	255,250,350,760<br>(207,653,348,326–310,596,455,536)	211,543,313,481<br>(172,770,071,101–252,287,410,697)	183,054,503,996<br>(147,980,217,868–223,824,474,809)
Iran	NCD	1,088,539,451,237<br>(904,242,511,499–1,254,429,307,675)	1,084,363,326,866<br>(910,243,402,248–1,246,440,927,073)	1,137,307,380,134<br>(976,019,785,029–1,311,417,336,726)	1,240,557,265,082<br>(1,052,602,179,900–1,443,741,866,176)	1,304,618,324,431<br>(1,095,218,936,731–1,528,121,529,101)	1,420,789,865,696<br>(1,182,911,758,736–1,675,361,961,620)
Iran	Injuries	721,542,826,981<br>(548,751,481,747–894,043,406,379)	376,638,167,480<br>(331,298,686,184–426,766,902,292)	358,987,247,634<br>(325,846,870,314–397,340,896,397)	399,192,365,200<br>(345,000,596,065–464,172,043,146)	352,268,981,327<br>(290,932,394,620–426,893,219,040)	314,386,030,393<br>(258,177,575,906–382,390,820,069)
Iraq	All causes	739,129,097,551<br>(674,591,094,661–807,320,524,615)	833,588,220,004<br>(758,198,107,254–913,516,196,477)	900,422,500,829<br>(813,764,086,494–992,344,867,722)	1,030,530,241,536<br>(913,764,121,046–1,155,899,822,254)	1,049,445,992,919<br>(918,913,590,726–1,193,665,041,932)	1,242,699,448,865<br>(1,066,001,467,744–1,428,634,601,770)
Iraq	Group I	274,148,312,682<br>(236,457,719,165–308,382,231,218)	292,977,345,892<br>(257,502,871,756–331,271,306,542)	286,494,590,689<br>(251,043,568,356–321,217,781,551)	279,065,918,916<br>(243,779,196,323–308,780,720,491)	274,016,043,093<br>(234,982,139,002–309,147,797,942)	252,000,462,613<br>(208,506,883,277–296,971,673,980)
Iraq	NCD	347,376,002,429<br>(292,742,408,800–402,944,452,406)	406,078,166,851<br>(346,107,126,778–469,901,053,153)	471,819,804,613<br>(408,347,245,045–542,201,510,876)	533,040,692,328<br>(459,160,048,192–614,651,772,647)	589,025,817,814<br>(500,465,123,246–686,671,828,219)	667,698,851,731<br>(564,451,806,462–784,231,418,071)
Iraq	Injuries	117,604,782,439<br>(100,506,466,999–137,490,649,028)	134,532,707,261<br>(113,963,738,726–155,005,799,065)	142,108,105,526<br>(119,020,472,984–163,349,948,566)	218,423,630,292<br>(173,467,882,594–264,129,772,459)	186,404,132,011<br>(152,096,273,346–219,293,486,868)	323,000,134,521<br>(226,006,128,622–416,372,100,391)
Jordan	All causes	93,449,107,267<br>(84,617,108,125–103,182,438,317)	109,641,592,987<br>(98,160,936,490–122,595,297,166)	113,742,808,410<br>(101,373,832,643–127,445,142,052)	118,007,497,540<br>(104,590,854,297–133,076,020,186)	123,546,836,727<br>(107,413,029,073–141,722,391,560)	141,150,757,729<br>(120,936,692,894–162,736,661,957)
Jordan	Group I	26,777,715,771<br>(24,067,173,277–29,850,452,348)	26,967,966,687<br>(24,641,049,410–29,619,109,758)	25,756,379,546<br>(23,505,762,674–28,313,819,798)	24,203,841,546<br>(21,670,594,979–27,140,046,827)	23,667,530,171<br>(20,775,345,487–27,000,933,767)	23,654,927,422<br>(20,106,471,089–27,631,303,619)
Jordan	NCD	52,823,010,636<br>(45,693,767,410–60,283,673,431)	66,978,978,654<br>(57,586,738,500–77,215,494,865)	72,451,996,461<br>(62,479,980,378–83,138,966,355)	77,760,369,983<br>(67,146,873,748–89,611,064,561)	86,302,668,669<br>(73,190,258,486–101,118,432,759)	101,462,726,289<br>(85,479,619,882–118,770,509,118)
Jordan	Injuries	13,848,380,861<br>(11,731,161,258–15,930,015,294)	15,694,647,646<br>(13,639,543,578–18,049,769,630)	15,534,432,403<br>(13,769,569,172–17,491,542,048)	16,043,286,011<br>(14,753,243,988–17,449,847,330)	13,576,637,887<br>(12,412,682,557–14,844,888,373)	16,033,104,018<br>(14,270,475,554–17,788,805,961)
Kuwait	All causes	38,519,889,248<br>(32,838,072,707–45,120,508,842)	30,655,050,316<br>(26,796,692,323–35,021,761,877)	33,623,745,066<br>(28,987,552,996–38,727,288,285)	39,294,468,561<br>(33,889,337,400–45,443,540,467)	49,566,876,461<br>(42,212,412,991–57,861,849,395)	59,116,626,206<br>(49,053,653,712–70,145,060,725)
Kuwait	Group I	5,668,863,908<br>(4,906,763,756–6,613,862,856)	4,595,629,802<br>(4,060,932,141–5,247,360,044)	4,283,969,362<br>(3,753,824,856–4,981,496,578)	4,669,514,820<br>(4,054,747,454–5,454,945,226)	5,945,076,026<br>(5,108,310,665–7,009,994,639)	6,307,572,986<br>(5,233,772,543–7,690,345,577)
Kuwait	NCD	23,526,646,389<br>(19,734,268,316–27,543,565,129)	21,183,454,653<br>(18,010,241,378–24,558,093,774)	24,469,952,826<br>(20,714,015,022–28,580,952,954)	29,004,459,477<br>(24,427,268,422–33,929,483,965)	37,424,962,979<br>(31,281,230,637–44,405,416,879)	46,174,944,816<br>(37,537,694,978–55,236,640,865)
Kuwait	Injuries	9,324,378,950<br>(5,880,955,707–13,033,443,791)	4,875,965,861<br>(4,475,652,368–5,353,020,322)	4,869,822,877<br>(4,421,049,668–5,402,523,095)	5,620,494,264<br>(5,192,970,736–6,133,835,926)	6,196,837,456<br>(5,591,892,569–6,877,185,136)	6,634,108,404<br>(5,710,461,003–7,754,394,560)
Lebanon	All causes	104,281,991,001<br>(92,556,649,327–117,613,845,637)	90,029,450,273<br>(78,385,281,013–102,909,791,329)	85,060,152,482<br>(72,996,169,378–98,259,209,454)	92,308,215,932<br>(77,904,480,414–107,167,797,367)	99,441,173,808<br>(82,691,737,556–117,648,012,319)	126,232,200,401<br>(104,286,222,504–150,554,281,189)

Appendix Table 3. DALYs for all causes and level 1 cause groups by location for 1990 - 2015, both sexes combined.

	Cause	1990	1995	2000	2005	2010	2015
Lebanon	Group I	17,665,902,916<br>(15,151,457,364–21,038,906,548)	15,177,948,122<br>(12,674,009,805–18,978,029,852)	12,418,647,475<br>(10,140,381,301–16,024,490,623)	9,772,540,737<br>(7,858,436,690–12,611,541,943)	8,784,046,164<br>(7,013,525,721–11,245,845,627)	10,378,314,850<br>(8,214,231,418–13,330,755,214)
Lebanon	NCD	56,284,032,824<br>(48,865,681,560–64,900,672,190)	57,029,404,405<br>(49,782,482,979–65,297,455,049)	56,679,723,966<br>(48,283,273,955–65,522,050,798)	66,578,909,351<br>(55,856,997,619–77,672,881,605)	75,718,552,395<br>(62,416,821,807–89,633,195,437)	98,392,222,143<br>(80,359,453,507–116,881,406,013)
Lebanon	Injuries	30,332,055,261<br>(24,778,341,469–37,543,475,353)	17,822,097,746<br>(13,111,756,588–24,096,069,728)	15,961,781,041<br>(11,421,358,398–21,777,551,704)	15,956,765,844<br>(11,269,251,587–22,103,780,253)	14,938,575,250<br>(10,821,559,564–20,726,237,152)	17,461,663,409<br>(12,632,077,820–23,825,679,547)
Libya	All causes	118,497,848,271<br>(106,628,255,412–131,107,719,307)	114,831,252,734<br>(102,118,988,261–128,642,423,177)	121,587,943,128<br>(107,491,793,869–136,652,036,992)	129,267,099,828<br>(113,577,127,808–146,275,575,001)	135,878,528,001<br>(118,370,306,204–154,867,143,220)	160,356,689,264<br>(136,552,830,978–184,288,655,082)
Libya	Group I	32,253,481,251<br>(28,334,439,217–37,123,848,770)	25,243,634,865<br>(22,055,399,561–29,358,091,891)	22,888,820,482<br>(19,457,278,674–26,927,640,998)	22,519,234,296<br>(18,627,536,400–27,404,594,949)	20,605,613,354<br>(16,678,114,901–25,613,618,344)	18,078,191,761<br>(14,473,029,626–22,586,758,527)
Libya	NCD	69,908,838,398<br>(60,742,231,531–79,667,323,601)	73,507,023,567<br>(63,673,959,001–84,525,385,180)	81,272,346,849<br>(69,955,504,052–93,290,914,399)	89,528,083,497<br>(77,243,411,647–102,622,862,907)	97,459,051,528<br>(83,435,492,092–112,327,606,078)	104,111,985,078<br>(88,444,715,379–120,150,343,022)
Libya	Injuries	16,335,528,621<br>(13,967,761,582–18,581,053,230)	16,080,594,303<br>(13,956,102,316–18,124,623,824)	17,426,775,798<br>(15,340,262,928–19,392,733,774)	17,219,782,034<br>(14,977,967,218–19,181,775,974)	17,813,863,119<br>(15,585,880,336–19,937,604,465)	38,166,512,424<br>(25,337,938,478–50,891,289,167)
Morocco	All causes	1,014,940,961,277<br>(933,869,074,219–1,101,534,523,683)	941,971,346,692<br>(855,525,124,178–1,036,235,313,104)	874,618,868,831<br>(774,350,952,801–978,838,001,820)	867,322,371,734<br>(746,682,902,859–984,578,216,415)	891,496,929,585<br>(764,648,135,660–1,034,343,852,803)	926,666,052,615<br>(784,470,995,485–1,084,978,781,670)
Morocco	Group I	436,358,259,377<br>(386,826,082,361–472,047,804,804)	329,239,931,609<br>(293,416,405,023–359,810,537,416)	249,859,025,601<br>(222,770,979,627–277,492,455,281)	211,305,062,698<br>(183,767,731,435–238,813,131,281)	189,331,435,855<br>(161,081,479,393–218,304,934,261)	166,990,768,865<br>(139,619,619,603–196,936,310,400)
Morocco	NCD	461,002,331,219<br>(399,201,442,157–528,103,803,995)	498,667,546,546<br>(429,294,700,803–571,488,227,869)	523,841,605,859<br>(448,720,739,473–600,146,728,486)	562,767,435,778<br>(468,385,422,651–652,653,181,633)	606,761,866,409<br>(504,328,364,147–717,025,987,958)	660,959,016,415<br>(545,573,702,249–784,200,851,819)
Morocco	Injuries	117,580,370,682<br>(95,713,474,012–136,171,169,978)	114,063,868,537<br>(98,094,862,873–129,573,938,671)	100,918,237,371<br>(87,918,421,726–115,185,555,780)	93,249,873,259<br>(79,405,557,232–109,955,416,592)	95,403,627,322<br>(79,366,410,355–115,708,250,775)	98,716,267,335<br>(79,878,755,368–122,826,201,050)
Palestine	All causes	68,200,513,737<br>(61,470,918,687–75,497,102,231)	68,728,299,025<br>(61,347,205,465–76,396,368,179)	78,220,557,051<br>(69,439,023,169–87,170,713,135)	81,378,393,694<br>(72,727,187,621–90,686,337,400)	89,683,213,773<br>(78,823,273,609–101,439,386,237)	99,559,130,549<br>(85,116,995,571–115,126,881,106)
Palestine	Group I	25,382,248,246<br>(21,835,259,761–28,549,073,845)	22,681,331,920<br>(19,719,970,153–25,529,761,993)	21,747,433,260<br>(19,070,935,110–24,345,004,528)	21,653,458,303<br>(18,805,849,323–24,589,678,349)	21,571,194,027<br>(18,409,592,461–24,593,426,354)	21,256,641,923<br>(17,149,505,706–25,986,638,879)
Palestine	NCD	33,007,376,782<br>(27,959,761,989–38,447,963,836)	37,373,815,725<br>(31,780,286,688–42,914,543,856)	43,816,341,474<br>(37,456,668,305–49,728,095,110)	49,710,036,592<br>(42,763,432,140–56,457,589,556)	57,821,634,219<br>(49,223,030,977–66,474,506,901)	67,489,155,248<br>(56,760,451,765–79,381,607,394)
Palestine	Injuries	9,810,888,709<br>(7,731,399,825–12,065,780,218)	8,673,151,380<br>(6,924,543,057–10,453,113,542)	12,656,782,317<br>(9,483,824,686–15,807,169,630)	10,014,898,800<br>(8,388,766,309–11,770,873,864)	10,290,385,527<br>(8,547,052,049–12,130,800,512)	10,813,333,379<br>(8,828,495,421–13,174,126,648)
Oman	All causes	57,962,512,052<br>(50,095,469,708–66,157,408,926)	52,652,812,967<br>(45,363,811,402–60,575,736,612)	44,882,038,304<br>(38,345,381,117–51,703,692,893)	46,907,703,065<br>(40,382,239,374–54,097,755,438)	57,621,239,514<br>(49,847,989,546–66,259,235,958)	83,802,798,342<br>(70,159,331,608–97,779,155,898)
Oman	Group I	19,580,037,153<br>(16,278,223,506–23,209,200,605)	13,031,194,763<br>(11,174,956,901–15,204,464,485)	8,416,609,635<br>(7,262,593,447–9,785,205,057)	7,089,163,021<br>(6,107,773,806–8,277,915,010)	8,233,732,282<br>(7,193,219,306–9,475,297,904)	9,884,844,418<br>(8,370,465,340–11,664,614,513)
Oman	NCD	28,016,455,015<br>(23,280,388,243–33,167,734,130)	29,917,346,230<br>(24,929,484,572–35,632,848,717)	28,701,732,526<br>(23,816,703,896–34,099,856,453)	32,110,450,584<br>(26,817,372,269–37,952,886,255)	39,898,706,828<br>(33,318,215,097–47,057,395,026)	59,937,527,908<br>(48,667,815,499–71,666,479,995)
Oman	Injuries	10,366,019,883<br>(7,706,800,974–12,945,346,481)	9,704,271,973<br>(7,762,836,370–11,649,369,803)	7,763,696,143<br>(6,743,579,174–9,088,081,652)	7,708,089,460<br>(6,984,772,798–8,499,668,978)	9,488,800,403<br>(8,688,077,404–10,311,217,290)	13,980,426,016<br>(11,796,046,303–16,741,659,920)
Qatar	All causes	10,248,859,558<br>(8,956,738,482–11,646,158,792)	10,836,240,878<br>(9,365,054,992–12,493,158,598)	12,602,347,642<br>(10,879,433,567–14,356,403,568)	16,025,061,275<br>(13,625,872,138–18,605,759,464)	28,791,187,517<br>(24,082,094,282–34,119,257,886)	34,646,818,861<br>(28,597,290,723–41,696,382,388)
Qatar	Group I	1,677,130,358<br>(1,308,510,955–2,185,539,953)	1,544,999,999<br>(1,232,659,702–1,923,270,399)	1,513,027,854<br>(1,209,260,348–1,880,426,371)	1,661,461,653<br>(1,334,597,311–2,010,809,172)	2,475,624,323<br>(2,009,771,936–3,050,150,760)	2,845,536,448<br>(2,252,595,158–3,596,943,151)
Qatar	NCD	6,165,891,264<br>(5,182,182,551–7,191,234,177)	6,820,504,076<br>(5,730,651,734–7,997,010,443)	8,318,933,762<br>(7,072,995,694–9,713,575,342)	10,888,038,973<br>(9,008,407,374–12,887,307,620)	19,951,441,806<br>(16,148,159,558–24,337,417,965)	24,669,433,539<br>(19,816,399,874–30,295,411,155)
Qatar	Injuries	2,405,837,936<br>(2,092,800,883–2,729,705,515)	2,470,736,803<br>(2,171,575,166–2,810,126,472)	2,770,386,027<br>(2,411,584,569–3,143,720,213)	3,475,560,649<br>(3,056,389,535–3,928,617,618)	6,364,121,387<br>(5,522,156,586–7,251,207,621)	7,131,848,873<br>(5,782,150,955–8,649,171,087)
Saudi Arabia	All causes	485,907,372,853<br>(435,986,356,992–543,896,405,891)	457,814,831,826<br>(409,087,650,256–507,218,373,729)	436,980,683,144<br>(386,721,134,662–494,598,051,558)	455,888,359,075<br>(397,943,425,635–522,041,564,427)	488,641,616,264<br>(423,024,886,286–562,542,202,180)	536,147,643,684<br>(456,788,907,718–623,833,671,656)
Saudi Arabia	Group I	178,063,915,825<br>(148,748,718,557–200,187,545,089)	139,812,154,301<br>(120,081,812,919–156,337,012,380)	100,686,246,277<br>(88,900,601,985–112,932,974,358)	82,993,004,222<br>(72,137,827,495–93,737,339,665)	74,287,853,023<br>(63,878,844,960–84,867,608,280)	67,720,551,640<br>(57,617,399,591–78,160,343,880)
Saudi Arabia	NCD	221,936,573,055<br>(186,643,676,297–263,002,140,870)	243,629,946,209<br>(204,937,715,327–282,516,308,386)	264,546,594,876<br>(221,970,712,516–308,484,568,197)	298,879,905,265<br>(249,192,860,250–350,981,509,533)	339,229,143,829<br>(281,826,946,893–400,099,225,522)	391,954,572,793<br>(323,309,480,219–465,300,950,650)
Saudi Arabia	Injuries	85,906,883,973<br>(67,530,426,027–133,158,323,702)	74,372,731,316<br>(66,460,560,423–81,600,819,446)	71,747,841,991<br>(65,438,091,819–77,616,809,853)	74,015,449,588<br>(67,738,420,192–79,658,548,812)	75,124,619,412<br>(68,423,871,887–81,191,786,093)	76,472,519,252<br>(68,176,680,892–84,843,954,867)
Sudan	All causes	1,522,243,278,211<br>(1,422,786,852,190–1,633,417,496,268)	1,662,482,095,388<br>(1,532,367,969,976–1,800,980,483,741)	1,678,140,772,315<br>(1,536,674,658,858–1,839,673,586,216)	1,619,712,227,110<br>(1,449,276,468,967–1,791,390,606,753)	1,606,623,224,646<br>(1,406,589,399,150–1,808,704,432,427)	1,582,025,649,282<br>(1,344,772,766,893–1,843,632,748,325)
Sudan	Group I	889,758,857,032<br>(752,041,269,725–1,008,406,738,533)	938,027,034,954<br>(795,307,125,689–1,075,070,822,333)	905,199,959,920<br>(768,464,283,763–1,034,689,423,275)	797,130,771,970<br>(676,458,285,549–909,197,845,697)	708,934,036,817<br>(599,450,401,419–834,086,440,614)	625,982,689,428<br>(502,474,944,941–780,815,753,845)
Sudan	NCD	454,462,839,239<br>(371,704,983,776–579,604,345,355)	540,969,552,965<br>(437,356,736,206–680,762,966,792)	571,575,650,169<br>(462,410,750,225–703,640,849,668)	623,264,820,070<br>(505,428,074,524–756,361,487,619)	686,137,936,868<br>(554,373,903,221–827,834,317,775)	746,616,929,376<br>(613,928,132,469–900,708,038,658)
Sudan	Injuries	178,021,581,940<br>(117,988,985,478–233,965,213,983)	183,485,507,469<br>(116,972,326,773–244,320,601,886)	201,365,162,225<br>(137,466,152,538–261,568,268,928)	199,316,635,070<br>(142,657,524,443–255,293,853,609)	211,551,250,962<br>(158,206,461,141–270,673,162,279)	209,426,030,478<br>(161,218,515,259–266,477,984,407)
Syria	All causes	385,587,676,356<br>(349,977,223,551–423,592,073,492)	361,967,047,500<br>(322,001,331,732–401,498,583,877)	352,044,899,339<br>(312,389,821,961–394,114,828,096)	355,299,777,964<br>(315,252,414,540–401,521,554,353)	387,611,264,329<br>(341,429,899,244–440,840,174,922)	709,874,191,439<br>(490,951,032,483–919,110,927,341)
Syria	Group I	154,667,720,430<br>(136,587,361,495–181,592,856,256)	119,902,366,844<br>(105,967,346,857–138,599,636,581)	88,898,234,911<br>(79,088,114,430–100,414,527,885)	78,635,627,362<br>(68,744,439,436–92,238,613,891)	73,768,988,785<br>(65,267,929,192–83,736,110,164)	60,766,060,654<br>(52,221,638,321–70,533,079,253)
Syria	NCD	199,045,549,900<br>(170,074,548,745–227,698,586,203)	210,718,272,550<br>(179,453,701,472–242,766,008,981)	230,812,646,277<br>(199,284,756,151–264,858,338,102)	246,155,111,886<br>(212,763,234,674–283,777,423,743)	280,334,127,624<br>(242,245,812,577–324,000,782,900)	260,299,403,915<br>(222,765,561,112–303,550,950,840)
Syria	Injuries	31,874,406,025<br>(25,940,255,444–37,273,979,262)	31,346,408,106<br>(26,225,140,719–36,520,949,907)	32,334,018,151<br>(28,448,851,123–36,727,988,464)	30,509,038,716<br>(26,916,560,321–33,898,293,836)	33,508,147,921<br>(30,519,263,661–36,684,276,248)	388,808,726,870<br>(176,422,057,994–592,952,974,030)
Tunisia	All causes	256,356,576,060<br>(234,636,681,552–280,845,707,001)	244,593,368,617<br>(220,030,070,459–272,402,072,187)	236,657,718,423<br>(209,164,518,080–267,999,257,534)	237,713,488,114<br>(206,658,285,225–273,438,487,771)	248,063,350,544<br>(213,457,214,283–287,269,912,822)	260,930,983,956<br>(221,622,756,348–304,043,137,067)
Tunisia	Group I	83,466,165,050<br>(72,250,828,466–93,681,761,885)	58,405,927,753<br>(52,029,520,472–64,985,451,090)	42,511,690,782<br>(37,899,564,426–47,052,826,325)	35,475,150,719<br>(31,221,855,154–40,108,568,049)	33,346,384,322<br>(28,996,512,308–38,290,434,625)	30,391,817,361<br>(25,775,320,058–35,346,059,633)
Tunisia	NCD	145,098,258,215<br>(126,348,717,341–164,720,756,192)	160,556,449,988<br>(139,797,694,706–183,491,226,134)	169,669,105,575<br>(146,365,582,831–195,631,401,811)	179,363,265,385<br>(153,518,813,338–209,364,584,938)	191,987,767,221<br>(162,326,735,978–225,405,942,770)	207,890,128,865<br>(174,101,635,204–244,469,332,956)
Tunisia	Injuries	27,792,152,795<br>(23,299,679,371–32,515,502,438)	25,630,990,875<br>(22,512,058,698–29,189,024,767)	24,476,922,066<br>(21,605,786,020–27,666,976,755)	22,875,072,009<br>(19,688,793,693–26,169,423,210)	22,729,199,001<br>(19,336,133,775–26,703,804,883)	22,649,037,730<br>(19,051,370,463–27,176,051,368)
Turkey	All causes	2,202,303,897,080<br>(2,031,203,429,355–2,384,400,363,944)	2,084,319,246,026<br>(1,900,067,468,544–2,288,791,979,206)	1,829,202,411,676<br>(1,651,181,933,684–2,030,260,760,411)	1,675,634,604,460<br>(1,484,873,810,508–1,885,088,264,286)	1,571,283,619,337<br>(1,364,854,026,826–1,791,264,133,273)	1,667,980,251,970<br>(1,433,873,317,641–1,921,953,692,009)
Turkey	Group I	791,665,969,982<br>(713,948,920,756–866,002,779,149)	632,273,231,032<br>(570,953,554,028–700,819,565,195)	439,889,457,580<br>(393,235,255,430–493,815,239,381)	307,327,252,288<br>(264,957,965,422–357,901,886,284)	237,975,533,915<br>(194,535,008,778–289,449,379,725)	203,238,598,447<br>(159,506,548,730–254,314,811,009)
Turkey	NCD	1,186,893,291,757<br>(1,047,376,056,375–1,327,842,379,982)	1,227,679,804,507<br>(1,077,188,363,714–1,382,700,606,015)	1,215,518,732,594<br>(1,063,501,628,964–1,384,609,936,517)	1,216,215,656,350<br>(1,046,953,993,928–1,394,052,974,248)	1,200,730,546,248<br>(1,025,682,373,569–1,393,005,159,498)	1,328,098,275,690<br>(1,130,484,917,760–1,551,322,248,424)
Turkey	Injuries	223,744,635,341<br>(187,127,342,107–255,936,040,101)	224,366,210,487<br>(199,206,542,355–247,538,314,816)	173,794,221,502<br>(157,880,733,792–190,676,816,306)	152,091,695,823<br>(139,019,879,389–165,509,232,616)	132,577,539,174<br>(121,573,981,109–144,015,118,871)	136,643,377,832<br>(123,627,752,318–151,737,048,230)

Appendix Table 3. DALYs for all causes and level 1 cause groups by location for 1990 - 2015, both sexes combined.

	Cause	1990	1995	2000	2005	2010	2015
United Arab Emirates	All causes	47,256,241,792<br>(39,084,348,152–55,813,879,426)	54,808,273,177<br>(46,663,950,532–63,225,812,925)	63,563,514,109<br>(54,291,550,820–73,575,106,451)	87,540,031,619<br>(74,555,310,924–101,334,552,404)	159,987,947,208<br>(133,027,284,176–189,907,699,566)	191,053,307,498<br>(155,529,487,985–231,174,544,206)
United Arab Emirates	Group I	9,692,938,036<br>(6,765,657,687–13,819,794,768)	8,253,719,715<br>(5,927,680,902–11,779,239,004)	7,575,018,355<br>(5,691,614,582–10,726,040,385)	8,650,102,910<br>(6,855,343,208–12,373,430,705)	13,067,036,234<br>(9,837,148,524–19,651,236,007)	13,887,250,806<br>(10,574,810,634–19,685,981,889)
United Arab Emirates	NCD	27,302,257,368<br>(22,532,931,521–32,570,178,105)	34,517,409,554<br>(28,729,858,718–40,454,920,204)	42,232,497,263<br>(35,077,410,220–49,620,320,900)	60,374,359,966<br>(50,061,255,898–71,825,018,565)	114,658,499,191<br>(94,013,795,685–137,408,660,536)	143,119,939,448<br>(116,229,669,906–173,196,246,539)
United Arab Emirates	Injuries	10,261,046,387<br>(8,023,041,240–12,604,458,765)	12,037,143,908<br>(9,914,772,136–14,132,233,867)	13,755,998,490<br>(11,675,740,723–15,961,808,922)	18,515,568,744<br>(15,603,377,974–21,067,879,499)	32,262,411,783<br>(25,878,625,940–39,317,436,480)	34,046,117,245<br>(25,467,415,111–42,782,049,197)
Yemen	All causes	988,599,862,533<br>(906,482,077,064–1,075,772,534,102)	1,024,953,986,688<br>(922,395,509,058–1,145,314,071,603)	951,113,315,504<br>(835,110,348,104–1,095,787,944,915)	902,714,368,502<br>(771,325,607,767–1,058,364,898,281)	879,552,393,732<br>(733,204,119,232–1,084,102,480,048)	1,048,950,926,572<br>(884,637,773,149–1,287,672,257,829)
Yemen	Group I	612,632,500,554<br>(501,432,169,812–715,592,038,234)	578,821,759,452<br>(474,186,502,376–683,169,592,413)	493,855,885,234<br>(405,528,538,482–587,188,242,430)	411,491,832,613<br>(341,272,432,986–489,639,880,252)	339,742,249,387<br>(287,627,626,848–390,398,360,406)	307,432,108,887<br>(261,546,320,700–363,535,210,877)
Yemen	NCD	278,040,189,485<br>(195,843,387,485–392,687,374,427)	339,238,317,325<br>(243,017,210,609–450,145,057,123)	353,170,127,194<br>(259,992,426,455–472,712,389,999)	379,358,036,878<br>(281,821,004,040–501,047,726,893)	423,344,159,014<br>(326,370,249,351–555,563,693,509)	481,960,008,012<br>(369,305,485,511–637,705,075,219)
Yemen	Injuries	97,927,172,495<br>(53,926,201,570–141,187,497,762)	106,893,909,911<br>(60,079,746,053–150,467,205,414)	104,087,303,075<br>(60,485,362,996–150,123,068,749)	111,864,499,011<br>(72,418,522,818–155,118,582,097)	116,465,985,332<br>(83,294,368,343–164,127,867,657)	259,558,809,673<br>(201,864,280,504–331,118,628,537)
South Asia	All causes	73,684,039,924,211<br>(70,251,198,572,328–77,529,688,018,805)	71,727,286,223,000<br>(68,004,455,532,691–75,965,834,157,628)	70,232,523,666,658<br>(66,026,935,965,935–74,957,164,217,013)	68,684,127,236,730<br>(64,342,009,247,399–73,690,199,232,995)	66,042,491,054,054<br>(61,340,688,172,545–71,194,734,440,970)	64,649,008,840,370<br>(59,556,220,977,430–70,120,999,872,764)
South Asia	Group I	45,427,175,249,697<br>(43,982,352,942,682–46,997,757,208,295)	40,774,672,897,781<br>(39,360,627,876,899–42,344,963,626,228)	36,706,968,100,963<br>(35,272,629,091,389–38,298,854,136,928)	33,026,950,670,889<br>(31,654,838,480,621–34,615,474,897,169)	28,361,652,342,093<br>(26,954,213,842,083–29,957,358,627,443)	24,312,716,829,477<br>(22,910,523,904,761–25,856,690,122,040)
South Asia	NCD	22,061,412,115,477<br>(19,780,976,622,437–24,390,765,590,108)	24,405,669,118,050<br>(21,869,013,563,256–27,021,510,881,730)	26,936,992,396,221<br>(24,089,114,864,408–29,844,791,683,753)	28,717,048,463,252<br>(25,589,623,601,445–31,970,281,635,925)	31,329,842,121,705<br>(27,999,166,759,669–34,896,128,205,711)	33,975,121,453,669<br>(30,281,436,788,685–37,879,304,813,905)
South Asia	Injuries	6,195,452,559,036<br>(5,222,932,735,722–6,796,977,319,905)	6,546,944,207,170<br>(5,384,897,595,207–7,130,547,682,656)	6,588,563,169,474<br>(5,463,071,482,509–7,130,750,257,545)	6,940,128,102,589<br>(5,930,434,374,197–7,501,667,510,510)	6,350,996,590,256<br>(5,343,445,877,118–6,856,073,865,975)	6,361,170,557,223<br>(5,399,730,968,075–6,963,278,380,136)
Bangladesh	All causes	7,683,884,738,500<br>(7,303,423,391,195–8,084,463,780,752)	6,753,940,005,500<br>(6,367,967,959,686–7,174,592,201,849)	6,032,779,645,544<br>(5,617,715,860,658–6,507,377,768,392)	5,556,677,789,508<br>(5,129,104,191,826–6,047,725,770,799)	5,166,880,818,818<br>(4,707,196,250,660–5,689,082,560,299)	5,023,799,910,305<br>(4,487,887,705,234–5,644,097,462,022)
Bangladesh	Group I	4,930,004,884,361<br>(4,696,075,147,404–5,173,760,170,060)	3,873,976,664,156<br>(3,666,802,469,084–4,085,173,436,600)	2,982,712,188,683<br>(2,799,361,915,206–3,152,105,417,274)	2,322,451,939,724<br>(2,176,861,847,025–2,473,478,694,906)	1,778,198,594,626<br>(1,653,211,065,702–1,913,637,895,030)	1,444,117,933,332<br>(1,315,686,685,396–1,582,001,101,753)
Bangladesh	NCD	1,983,058,711,485<br>(1,717,403,290,465–2,305,693,524,990)	2,133,328,479,194<br>(1,838,799,442,079–2,456,088,583,317)	2,348,798,194,123<br>(2,044,759,873,822–2,684,259,436,434)	2,605,877,770,828<br>(2,268,199,060,972–2,956,647,728,863)	2,831,646,608,196<br>(2,468,648,395,233–3,203,062,620,282)	3,044,645,462,015<br>(2,617,228,118,303–3,511,044,383,587)
Bangladesh	Injuries	770,821,142,654<br>(560,008,340,043–921,239,019,845)	746,634,862,150<br>(530,576,021,775–883,634,390,838)	701,269,262,739<br>(495,806,614,340–830,504,070,959)	628,348,078,957<br>(467,081,423,064–722,563,833,210)	557,035,615,996<br>(435,290,661,143–630,808,236,371)	535,036,514,958<br>(420,496,688,103–620,332,195,306)
Bhutan	All causes	34,690,929,818<br>(32,165,857,275–37,284,997,974)	27,674,848,130<br>(25,625,705,704–29,849,160,551)	25,566,943,834<br>(23,424,363,769–27,961,436,229)	23,305,359,121<br>(21,018,027,685–25,696,497,428)	22,558,726,185<br>(19,976,832,821–25,334,068,621)	22,439,730,430<br>(19,582,671,822–25,581,788,707)
Bhutan	Group I	22,782,786,826<br>(20,816,303,283–24,829,260,718)	16,955,408,169<br>(15,523,964,961–18,546,396,062)	13,228,325,386<br>(12,045,851,111–14,472,215,584)	11,310,454,144<br>(10,255,857,110–12,533,056,905)	9,530,148,249<br>(8,495,048,101–10,660,358,934)	8,075,963,089<br>(7,085,520,019–9,219,026,386)
Bhutan	NCD	9,569,777,031<br>(8,108,815,932–11,145,156,422)	8,730,290,544<br>(7,459,843,121–10,144,712,217)	9,149,185,549<br>(7,864,077,957–10,532,292,063)	10,183,142,534<br>(8,666,595,300–11,776,462,838)	11,268,150,211<br>(9,477,492,813–13,156,596,278)	12,631,875,747<br>(10,549,789,197–14,816,911,373)
Bhutan	Injuries	2,338,365,961<br>(1,664,262,775–3,043,681,871)	1,989,149,418<br>(1,491,148,914–2,519,319,145)	3,189,432,898<br>(2,542,166,230–3,902,623,100)	1,811,762,443<br>(1,522,587,492–2,096,042,049)	1,760,427,724<br>(1,481,581,154–2,061,330,504)	1,731,891,594<br>(1,432,515,361–2,068,184,182)
India	All causes	57,617,147,581,363<br>(54,838,957,756,606–60,780,900,910,963)	56,433,435,996,423<br>(53,382,850,542,896–59,894,652,956,055)	55,676,683,032,429<br>(52,388,197,967,661–59,463,122,214,825)	54,026,546,254,793<br>(50,557,736,176,279–58,048,354,808,681)	52,003,910,033,314<br>(48,257,641,111,668–56,161,650,967,772)	50,578,804,037,895<br>(46,527,548,008,534–54,932,406,648,531)
India	Group I	34,809,123,387,749<br>(33,644,671,970,976–36,109,650,763,821)	31,553,059,406,314<br>(30,403,831,796,718–32,884,568,616,706)	28,812,719,982,489<br>(27,583,647,184,832–30,174,827,299,833)	26,102,900,721,657<br>(24,904,953,266,332–27,452,653,498,138)	22,215,241,667,957<br>(21,049,082,820,226–23,532,802,643,823)	18,824,362,777,368<br>(17,593,447,140,566–20,108,427,876,334)
India	NCD	17,858,941,056,088<br>(15,987,929,427,111–19,733,927,061,012)	19,608,136,636,345<br>(17,509,932,531,510–21,725,504,114,055)	21,557,618,263,227<br>(19,267,492,935,611–23,890,207,911,927)	22,734,176,620,714<br>(20,311,213,690,133–25,302,645,497,697)	24,742,860,426,647<br>(22,167,393,123,168–27,593,718,187,061)	26,744,628,757,387<br>(23,769,503,353,442–29,864,862,695,721)
India	Injuries	4,949,083,137,526<br>(4,256,749,933,505–5,435,251,500,584)	5,272,239,953,764<br>(4,382,149,175,136–5,752,890,424,595)	5,306,344,786,714<br>(4,446,040,341,021–5,750,189,351,579)	5,189,468,912,422<br>(4,388,419,459,758–5,595,370,519,195)	5,045,807,938,710<br>(4,249,942,011,914–5,458,317,792,680)	5,009,812,503,139<br>(4,235,425,502,192–5,508,790,428,235)
Nepal	All causes	1,393,162,006,831<br>(1,320,247,242,030–1,475,167,731,716)	1,273,782,884,553<br>(1,204,510,005,562–1,353,491,657,288)	1,115,905,341,176<br>(1,044,295,509,109–1,193,791,045,462)	1,004,809,033,274<br>(926,553,041,716–1,088,482,396,015)	903,790,327,374<br>(811,369,471,573–1,000,120,273,875)	929,689,265,621<br>(814,979,286,812–1,048,284,555,339)
Nepal	Group I	996,531,764,949<br>(952,863,281,423–1,040,236,639,568)	849,547,253,163<br>(813,738,554,707–886,290,746,515)	678,755,198,472<br>(647,006,694,814–710,087,521,699)	537,936,577,052<br>(504,209,044,881–573,615,184,659)	413,536,450,039<br>(377,509,613,318–451,061,334,162)	329,070,657,115<br>(293,001,279,726–366,496,282,812)
Nepal	NCD	319,815,524,952<br>(275,763,493,338–369,903,258,383)	344,754,984,793<br>(298,760,727,335–397,267,345,209)	360,584,085,156<br>(313,177,208,475–414,280,513,374)	386,433,958,529<br>(329,891,327,401–446,489,618,206)	420,766,606,391<br>(353,528,771,161–489,846,346,409)	469,373,926,024<br>(390,277,367,637–551,205,093,093)
Nepal	Injuries	76,814,716,930<br>(62,287,372,056–92,023,019,613)	79,480,646,596<br>(64,739,462,993–94,250,661,420)	76,566,057,549<br>(63,618,541,791–89,315,730,241)	80,438,497,693<br>(67,827,269,982–94,592,487,538)	69,487,270,943<br>(56,817,543,552–84,820,616,219)	131,244,682,483<br>(101,536,326,021–161,838,130,246)
Pakistan	All causes	6,955,154,667,699<br>(6,588,986,864,224–7,335,376,201,168)	7,238,452,488,394<br>(6,821,186,328,412–7,731,007,897,321)	7,381,588,703,674<br>(6,900,334,877,430–7,902,396,932,163)	8,072,788,800,034<br>(7,521,662,411,764–8,682,675,901,947)	7,945,351,148,363<br>(7,266,619,623,803–8,661,403,908,065)	8,094,275,896,118<br>(7,345,110,189,198–8,920,765,234,082)
Pakistan	Group I	4,668,732,425,813<br>(4,480,301,400,478–4,868,000,398,254)	4,481,134,165,980<br>(4,283,695,575,006–4,682,476,031,673)	4,219,552,405,933<br>(4,046,752,585,553–4,406,922,231,267)	4,052,350,978,312<br>(3,854,108,247,372–4,259,646,168,393)	3,945,145,481,220<br>(3,704,312,701,508–4,192,615,560,096)	3,707,089,498,573<br>(3,424,579,795,123–4,024,073,129,088)
Pakistan	NCD	1,890,027,045,922<br>(1,651,821,265,532–2,128,069,759,211)	2,310,718,727,174<br>(2,029,667,462,283–2,627,666,863,342)	2,660,842,668,166<br>(2,330,240,446,429–2,987,713,824,309)	2,980,376,970,648<br>(2,602,761,982,421–3,340,557,902,869)	3,323,300,330,260<br>(2,873,805,202,000–3,787,054,184,810)	3,703,841,432,496<br>(3,213,287,533,576–4,269,137,541,182)
Pakistan	Injuries	396,395,195,964<br>(322,395,958,391–488,742,033,379)	446,599,595,241<br>(370,664,436,184–526,530,381,258)	501,193,629,575<br>(420,388,930,745–584,608,557,154)	1,040,060,851,074<br>(812,162,220,185–1,273,453,322,725)	676,905,336,883<br>(582,911,443,497–777,527,838,604)	683,344,965,048<br>(585,628,682,157–798,835,574,290)
Sub-Saharan Africa	All causes	48,380,267,790,927<br>(46,651,216,383,962–50,295,706,659,859)	52,781,697,970,130<br>(50,793,363,722,760–54,951,494,446,663)	57,624,536,397,088<br>(55,458,794,716,860–60,030,022,931,031)	58,195,991,678,457<br>(55,867,689,634,717–60,870,646,126,119)	55,563,233,889,627<br>(52,818,714,396,663–58,953,261,249,531)	52,929,561,173,093<br>(49,696,169,702,407–56,483,238,499,816)
Sub-Saharan Africa	Group I	35,840,385,274,228<br>(34,388,653,556,368–37,338,196,701,424)	39,422,156,127,179<br>(37,925,818,983,344–40,961,636,651,634)	42,551,697,541,940<br>(41,076,359,906,737–44,171,856,601,541)	42,680,791,535,811<br>(41,286,945,979,226–44,265,397,043,971)	38,623,399,004,714<br>(37,151,332,406,468–40,430,743,000,558)	33,737,789,801,363<br>(32,092,308,423,193–35,631,677,717,159)
Sub-Saharan Africa	NCD	9,191,637,654,218<br>(8,069,751,954,859–10,447,313,307,193)	10,154,750,328,394<br>(8,948,191,825,978–11,481,593,402,433)	11,350,954,027,214<br>(10,046,962,119,664–12,752,389,073,544)	12,215,120,600,077<br>(10,774,154,806,326–13,768,370,148,115)	13,470,205,719,776<br>(11,904,527,700,720–15,187,267,864,281)	15,331,061,989,086<br>(13,541,678,584,064–17,410,583,258,429)
Sub-Saharan Africa	Injuries	3,348,244,862,482<br>(2,786,118,068,322–3,841,504,484,046)	3,204,791,514,558<br>(2,696,268,562,222–3,654,920,996,684)	3,721,884,827,934<br>(3,199,978,045,725–4,185,532,691,258)	3,300,079,542,570<br>(2,914,736,318,094–3,646,401,303,515)	3,469,629,165,137<br>(3,136,956,693,686–3,776,912,415,541)	3,860,709,382,644<br>(3,478,538,856,167–4,226,009,560,808)
Southern Sub-Saharan Africa	All causes	2,649,151,774,750<br>(2,490,120,880,148–2,825,927,828,271)	3,041,165,859,677<br>(2,849,872,384,282–3,257,559,030,858)	4,371,419,499,290<br>(4,092,998,193,352–4,693,165,662,448)	5,509,213,452,670<br>(5,163,538,759,161–5,857,549,325,718)	5,263,341,960,859<br>(4,946,944,771,598–5,617,840,503,473)	4,189,838,191,337<br>(3,887,503,590,762–4,527,647,754,885)
Southern Sub-Saharan Africa	Group I	1,324,907,458,278<br>(1,258,122,631,092–1,400,066,577,556)	1,605,912,670,003<br>(1,506,027,146,372–1,736,971,378,091)	2,622,650,868,356<br>(2,447,275,367,045–2,818,811,697,292)	3,701,414,725,713<br>(3,473,552,684,275–3,948,022,800,018)	3,517,103,843,657<br>(3,317,480,228,175–3,740,681,873,615)	2,336,115,472,887<br>(2,208,015,302,331–2,478,768,362,535)
Southern Sub-Saharan Africa	NCD	930,071,349,460<br>(823,001,375,198–1,043,064,682,836)	1,034,150,793,062<br>(908,501,505,403–1,164,718,412,267)	1,279,982,312,349<br>(1,132,495,290,948–1,442,061,959,895)	1,358,516,071,099<br>(1,184,631,365,607–1,550,121,315,726)	1,343,360,771,693<br>(1,167,132,037,527–1,545,735,668,345)	1,439,361,794,825<br>(1,250,840,442,090–1,633,786,420,423)
Southern Sub-Saharan Africa	Injuries	394,172,967,012<br>(356,159,818,622–424,425,004,499)	401,102,396,612<br>(362,093,516,834–437,616,411,438)	468,786,318,585<br>(421,594,716,643–515,239,624,960)	449,282,655,857<br>(393,955,811,039–507,229,880,379)	402,877,345,509<br>(356,137,895,473–459,104,151,516)	414,360,923,626<br>(370,770,854,819–466,984,761,959)
Botswana	All causes	61,750,131,716<br>(44,492,604,835–97,639,645,745)	89,923,945,835<br>(58,610,620,068–150,434,121,947)	149,406,724,895<br>(89,163,500,843–250,360,878,057)	151,888,796,092<br>(93,285,189,720–258,020,942,873)	130,021,321,666<br>(79,563,423,818–237,777,315,094)	126,974,748,237<br>(79,228,908,454–239,582,644,967)
Botswana	Group I	35,745,697,986<br>(27,862,728,198–50,229,775,239)	57,121,432,003<br>(38,648,976,589–82,949,068,208)	105,533,515,940<br>(66,093,128,902–152,059,885,439)	102,918,046,261<br>(68,206,942,040–145,283,946,136)	79,805,222,074<br>(51,577,389,211–116,319,271,301)	72,414,717,662<br>(47,336,606,584–103,900,816,593)
Botswana	NCD	20,463,981,458<br>(12,743,450,218–36,287,477,007)	25,760,953,218<br>(14,502,158,477–54,090,011,951)	33,607,401,398<br>(16,827,857,246–77,785,806,024)	37,290,680,693<br>(18,603,195,193–84,687,570,879)	38,889,920,771<br>(21,106,037,065–89,128,889,141)	43,101,960,138<br>(24,504,035,005–101,182,833,317)

Appendix Table 3. DALYs for all causes and level 1 cause groups by location for 1990 - 2015, both sexes combined.

	Cause	1990	1995	2000	2005	2010	2015
Botswana	Injuries	5,540,452,271<br>(2,974,808,195–11,018,438,294)	7,041,560,615<br>(3,351,812,008–17,199,848,964)	10,265,807,557<br>(3,924,374,878–30,037,520,092)	11,680,069,138<br>(4,240,982,540–33,822,156,250)	11,326,178,820<br>(4,486,751,298–35,187,154,180)	11,458,070,437<br>(4,783,415,387–36,667,549,935)
Lesotho	All causes	92,611,220,451<br>(84,361,837,576–101,825,542,742)	106,506,476,164<br>(96,532,554,131–116,544,988,250)	150,035,560,254<br>(133,720,987,226–169,852,999,313)	204,840,779,205<br>(180,611,263,302–231,731,922,179)	188,554,894,993<br>(162,438,114,719–217,561,496,811)	194,868,788,529<br>(156,994,882,209–241,646,567,062)
Lesotho	Group I	58,679,115,169<br>(54,558,138,661–64,175,057,877)	69,048,503,876<br>(63,912,842,423–75,005,910,055)	106,555,243,489<br>(94,600,402,526–122,747,406,990)	147,262,692,461<br>(129,701,127,721–168,551,407,821)	128,193,900,640<br>(113,612,092,554–145,710,052,416)	129,871,084,813<br>(108,444,436,867–154,284,451,444)
Lesotho	NCD	25,685,085,183<br>(20,951,936,000–30,629,969,911)	27,948,496,589<br>(22,145,154,347–33,247,057,193)	32,267,588,471<br>(26,228,180,549–39,607,186,123)	42,444,780,836<br>(35,240,550,516–51,487,355,407)	43,762,212,109<br>(33,534,814,341–56,030,366,877)	46,954,308,250<br>(32,674,647,156–65,851,906,580)
Lesotho	Injuries	8,247,020,098<br>(6,547,195,762–10,269,847,172)	9,509,475,699<br>(7,198,334,877–11,729,770,831)	11,212,728,294<br>(8,501,231,688–14,452,399,988)	15,133,305,908<br>(11,384,335,582–19,223,622,338)	16,598,782,245<br>(10,835,218,683–23,920,845,206)	18,043,395,466<br>(9,564,122,499–29,843,156,483)
Namibia	All causes	72,789,135,753<br>(67,414,238,183–78,516,112,339)	83,636,535,406<br>(76,424,687,164–91,729,395,467)	123,816,134,119<br>(110,758,260,263–138,925,653,449)	139,685,195,084<br>(123,839,026,968–158,655,821,203)	110,017,153,478<br>(96,000,514,453–127,211,942,040)	107,165,065,835<br>(88,835,887,229–129,101,908,458)
Namibia	Group I	44,466,372,061<br>(41,460,594,753–47,802,279,355)	49,911,293,455<br>(45,748,837,963–54,657,746,432)	78,458,772,673<br>(68,905,041,548–90,604,672,864)	96,198,259,670<br>(84,413,744,837–111,605,812,949)	70,316,017,145<br>(61,720,875,244–82,606,793,386)	64,402,777,662<br>(54,382,243,667–75,858,451,258)
Namibia	NCD	21,148,189,363<br>(17,979,528,647–24,311,433,321)	25,540,602,598<br>(21,744,492,250–29,388,212,649)	33,754,503,276<br>(27,992,862,025–40,147,950,262)	32,935,245,074<br>(26,593,244,525–40,439,855,460)	30,430,399,972<br>(23,864,908,902–37,408,406,637)	33,386,549,722<br>(25,555,312,476–43,283,144,436)
Namibia	Injuries	7,174,574,329<br>(5,969,129,727–8,366,836,819)	8,184,639,354<br>(6,749,126,558–9,744,181,360)	11,602,858,169<br>(9,243,547,783–14,423,027,328)	10,551,690,341<br>(8,148,464,744–13,859,107,457)	9,270,736,362<br>(6,571,797,118–12,000,304,242)	9,375,738,450<br>(5,932,255,493–14,254,700,803)
South Africa	All causes	1,876,379,600,455<br>(1,761,083,666,867–2,007,086,723,220)	1,986,174,240,231<br>(1,862,681,989,022–2,128,702,903,290)	2,825,808,299,896<br>(2,639,362,154,212–3,025,867,162,546)	3,680,054,268,786<br>(3,422,268,155,211–3,949,515,487,438)	3,585,149,143,069<br>(3,334,927,163,239–3,857,610,908,656)	2,827,086,178,664<br>(2,628,249,992,648–3,040,959,452,968)
South Africa	Group I	838,106,863,955<br>(792,754,357,913–895,250,022,897)	865,920,311,248<br>(815,227,199,387–935,494,507,751)	1,472,379,925,702<br>(1,364,787,537,650–1,599,052,888,797)	2,318,450,653,445<br>(2,143,548,959,199–2,528,015,187,408)	2,305,314,089,886<br>(2,136,048,422,023–2,508,993,988,201)	1,447,643,715,415<br>(1,354,395,906,236–1,555,954,901,997)
South Africa	NCD	704,861,508,090<br>(623,589,643,320–786,587,277,356)	785,127,664,592<br>(696,632,617,073–880,956,694,417)	970,107,677,778<br>(859,105,625,699–1,095,335,160,217)	1,008,455,266,365<br>(871,832,963,944–1,159,316,738,314)	978,180,734,211<br>(840,836,653,527–1,126,083,560,446)	1,066,144,826,676<br>(934,946,339,625–1,204,258,667,400)
South Africa	Injuries	333,411,228,410<br>(298,402,531,486–359,658,086,883)	335,126,264,391<br>(298,119,517,062–360,422,554,755)	383,320,696,416<br>(341,398,087,720–419,033,305,468)	353,148,348,977<br>(306,234,682,877–403,563,291,487)	301,654,318,972<br>(260,952,944,375–347,398,305,372)	313,297,636,574<br>(278,597,407,702–350,695,402,090)
Swaziland	All causes	46,562,907,221<br>(41,656,225,359–51,910,382,595)	54,236,056,984<br>(48,161,452,977–60,641,299,677)	86,961,226,784<br>(76,212,017,240–99,897,259,087)	118,585,098,539<br>(103,822,916,490–136,338,538,379)	110,207,990,161<br>(92,859,825,371–129,493,778,480)	93,755,018,660<br>(73,543,379,444–116,951,759,448)
Swaziland	Group I	28,181,234,190<br>(25,887,169,787–30,891,983,529)	35,647,699,238<br>(32,134,477,287–40,251,683,784)	61,481,638,686<br>(53,201,550,659–72,700,982,658)	85,916,290,965<br>(74,680,561,651–100,075,484,946)	75,782,693,754<br>(66,085,837,178–88,869,650,093)	58,588,273,313<br>(49,552,725,914–69,632,798,400)
Swaziland	NCD	13,335,518,754<br>(10,797,106,925–16,030,005,678)	13,791,699,510<br>(10,601,791,179–17,069,427,912)	18,718,677,621<br>(14,493,425,159–23,908,458,731)	23,955,551,988<br>(18,566,936,855–30,970,123,571)	25,290,764,743<br>(17,856,933,114–34,710,880,443)	26,013,060,752<br>(16,907,953,905–37,798,327,655)
Swaziland	Injuries	5,046,154,278<br>(3,863,137,203–6,475,014,341)	4,796,658,236<br>(3,318,290,855–6,200,204,748)	6,760,910,477<br>(4,769,343,784–9,140,263,333)	8,713,255,587<br>(6,050,711,991–12,201,495,196)	9,134,531,664<br>(5,312,752,383–14,283,122,749)	9,153,684,595<br>(4,504,872,760–15,923,725,809)
Zimbabwe	All causes	499,058,779,155<br>(458,905,059,771–545,360,928,756)	720,688,605,056<br>(632,260,812,602–836,529,962,461)	1,035,391,553,342<br>(890,278,730,079–1,201,364,607,141)	1,214,159,314,963<br>(1,074,523,931,662–1,365,214,672,151)	1,139,391,457,490<br>(1,030,338,774,716–1,253,113,837,025)	839,988,391,411<br>(720,270,075,385–963,723,389,857)
Zimbabwe	Group I	319,728,174,919<br>(295,066,013,705–355,741,214,560)	528,263,430,183<br>(455,472,524,783–630,221,984,496)	798,241,771,867<br>(678,317,925,794–950,809,140,228)	950,668,782,912<br>(831,405,555,404–1,085,771,324,938)	857,691,920,158<br>(776,154,646,018–946,469,040,392)	563,194,904,022<br>(495,384,082,610–641,250,696,697)
Zimbabwe	NCD	144,577,066,611<br>(122,143,430,032–168,753,926,567)	155,981,376,554<br>(120,652,267,980–198,640,449,966)	191,526,463,804<br>(150,155,388,044–247,457,108,407)	213,434,546,143<br>(168,987,345,977–265,416,067,265)	226,806,739,887<br>(183,727,794,089–273,844,083,023)	223,761,089,286<br>(170,547,018,051–285,855,903,930)
Zimbabwe	Injuries	34,753,537,626<br>(27,352,442,663–44,637,304,158)	36,443,798,318<br>(24,847,905,209–54,735,866,191)	45,623,317,671<br>(30,225,446,201–70,337,205,663)	50,055,985,907<br>(33,834,816,404–71,018,900,681)	54,892,797,445<br>(39,945,874,048–73,178,333,356)	53,032,398,103<br>(33,860,381,000–76,054,360,906)
Western Sub-Saharan Africa	All causes	20,627,757,614,096<br>(19,764,882,759,199–21,598,701,693,929)	22,377,814,392,290<br>(21,535,773,409,842–23,324,469,413,898)	24,110,729,930,113<br>(23,237,371,110,687–25,149,566,975,761)	24,753,014,868,018<br>(23,711,370,726,047–25,918,269,639,834)	23,851,492,265,111<br>(22,691,251,834,277–25,203,959,031,209)	22,955,239,091,202<br>(21,426,978,733,742–24,747,555,479,809)
Western Sub-Saharan Africa	Group I	15,903,554,571,608<br>(15,102,861,784,810–16,663,910,754,194)	17,123,675,148,665<br>(16,337,819,911,303–17,844,218,631,278)	18,298,088,562,459<br>(17,463,536,557,067–19,061,558,826,005)	18,720,176,829,918<br>(17,845,172,194,429–19,481,730,993,490)	17,268,794,551,875<br>(16,422,493,891,876–18,085,859,294,978)	15,539,418,351,394<br>(14,569,499,405,329–16,591,131,088,088)
Western Sub-Saharan Africa	NCD	3,676,314,985,011<br>(3,089,531,210,839–4,368,341,136,108)	4,097,879,828,064<br>(3,525,285,996,917–4,814,009,095,274)	4,583,882,397,181<br>(3,946,419,931,042–5,342,950,582,963)	4,863,819,659,911<br>(4,182,814,675,073–5,665,479,541,132)	5,346,463,290,838<br>(4,593,626,162,082–6,182,327,833,044)	6,002,789,800,719<br>(5,171,116,532,735–6,941,796,338,554)
Western Sub-Saharan Africa	Injuries	1,047,888,057,476<br>(817,377,432,862–1,253,442,678,200)	1,156,259,415,561<br>(939,581,913,820–1,377,357,290,486)	1,228,758,970,473<br>(1,010,042,422,496–1,434,721,432,403)	1,169,018,378,188<br>(988,649,977,272–1,361,295,320,386)	1,236,234,422,398<br>(1,066,274,005,823–1,410,929,633,210)	1,413,030,939,089<br>(1,232,015,127,753–1,607,723,867,478)
Benin	All causes	492,230,802,427<br>(468,486,737,052–517,074,638,317)	532,020,231,176<br>(504,906,642,894–560,430,185,130)	560,942,994,924<br>(526,572,016,031–598,339,559,614)	570,794,966,762<br>(522,086,078,997–625,087,414,492)	559,832,324,512<br>(485,372,225,311–649,156,739,038)	555,035,393,436<br>(456,806,899,399–681,786,952,731)
Benin	Group I	372,150,698,450<br>(346,107,220,464–401,298,100,425)	391,857,809,069<br>(363,958,015,873–420,366,875,159)	402,463,002,469<br>(373,944,587,616–432,659,946,021)	398,420,408,954<br>(371,414,300,484–427,551,373,171)	365,214,990,717<br>(327,728,524,750–407,341,478,356)	330,633,250,844<br>(284,862,057,244–384,306,645,785)
Benin	NCD	90,727,558,010<br>(73,628,550,017–110,730,973,203)	105,746,082,384<br>(87,738,682,359–125,558,749,856)	121,779,532,919<br>(99,995,382,090–147,282,363,523)	137,722,332,268<br>(111,710,469,575–168,742,990,506)	158,029,275,655<br>(122,885,470,980–204,106,634,114)	184,078,724,178<br>(136,430,489,053–250,595,590,935)
Benin	Injuries	29,352,545,968<br>(19,003,801,570–39,663,173,325)	34,416,339,723<br>(23,996,168,006–44,513,335,718)	36,700,459,537<br>(26,176,222,461–45,786,575,406)	34,652,225,540<br>(26,948,462,867–43,120,155,845)	36,588,058,140<br>(26,542,996,650–48,799,709,549)	40,323,418,414<br>(28,021,322,414–58,577,837,925)
Burkina Faso	All causes	1,023,979,741,046<br>(979,756,313,168–1,069,180,876,595)	1,127,794,678,998<br>(1,075,518,767,492–1,177,706,608,110)	1,195,336,604,559<br>(1,136,436,223,630–1,254,704,652,907)	1,187,840,154,305<br>(1,117,829,451,232–1,268,650,521,931)	1,144,199,743,158<br>(1,049,358,598,336–1,250,654,146,355)	1,123,577,720,523<br>(990,829,601,690–1,286,375,563,210)
Burkina Faso	Group I	820,480,251,890<br>(778,528,602,817–857,839,099,989)	906,965,537,825<br>(862,820,152,583–948,238,230,832)	952,301,615,850<br>(903,456,814,229–998,252,795,861)	935,847,325,140<br>(882,516,421,105–988,678,747,186)	863,345,330,849<br>(799,324,411,211–936,124,497,065)	786,291,093,618<br>(705,151,700,685–883,919,686,292)
Burkina Faso	NCD	152,453,592,238<br>(126,191,860,667–186,477,294,774)	166,762,874,358<br>(138,765,884,875–199,214,478,213)	186,806,333,356<br>(156,066,633,390–227,234,867,034)	195,783,554,548<br>(161,358,012,382–239,338,820,307)	221,421,637,398<br>(175,683,994,877–273,504,019,994)	265,224,423,083<br>(202,756,129,322–342,149,801,434)
Burkina Faso	Injuries	51,045,896,917<br>(39,184,532,672–64,845,657,583)	54,066,266,814<br>(43,608,374,234–65,949,949,845)	56,228,655,353<br>(47,134,828,868–66,972,455,525)	56,209,274,617<br>(47,268,175,235–68,277,907,081)	59,432,774,910<br>(47,907,385,741–74,491,346,550)	72,062,203,822<br>(55,410,359,592–93,779,678,149)
Cameroon	All causes	976,998,297,707<br>(925,790,201,671–1,034,128,319,137)	1,151,189,793,995<br>(1,086,383,111,129–1,220,440,281,069)	1,344,397,399,495<br>(1,257,831,700,575–1,436,718,206,707)	1,442,539,866,681<br>(1,338,203,113,338–1,550,926,352,585)	1,412,615,906,315<br>(1,267,923,774,589–1,564,261,408,369)	1,407,233,045,884<br>(1,193,751,022,407–1,653,526,696,027)
Cameroon	Group I	701,917,149,718<br>(654,954,231,421–750,203,848,423)	836,112,849,652<br>(786,845,600,498–891,149,971,828)	980,191,988,375<br>(918,138,677,802–1,044,787,094,954)	1,046,723,656,318<br>(981,101,077,983–1,121,193,138,275)	968,852,585,782<br>(881,881,524,898–1,056,977,569,158)	910,012,445,877<br>(787,805,994,445–1,032,105,154,631)
Cameroon	NCD	217,311,137,104<br>(185,228,433,273–254,132,001,532)	247,451,115,047<br>(207,289,639,703–290,430,328,559)	290,380,129,213<br>(240,580,873,698–343,336,330,222)	319,537,849,502<br>(265,819,873,002–377,600,042,011)	357,945,464,590<br>(284,366,594,966–437,320,938,485)	399,284,476,665<br>(296,595,736,137–528,555,894,409)
Cameroon	Injuries	57,770,010,885<br>(41,426,698,377–72,485,756,566)	67,625,829,295<br>(51,442,704,733–84,961,963,653)	73,825,281,908<br>(55,486,810,422–92,738,394,960)	76,278,360,862<br>(60,086,295,546–94,193,085,168)	85,817,855,944<br>(66,142,176,296–107,412,092,576)	97,936,123,341<br>(71,604,132,474–131,343,842,652)
Cape Verde	All causes	14,169,813,517<br>(13,204,186,450–15,174,923,067)	14,682,394,477<br>(13,275,192,485–16,195,963,973)	15,016,320,065<br>(12,943,615,066–17,217,367,481)	14,984,507,459<br>(12,874,768,616–17,069,701,995)	14,399,179,526<br>(12,983,159,989–15,961,671,410)	13,919,585,908<br>(12,242,077,520–15,867,579,597)
Cape Verde	Group I	7,464,758,926<br>(6,983,862,456–8,055,851,004)	6,874,374,798<br>(6,248,644,820–7,640,443,035)	6,020,956,793<br>(5,383,254,110–6,766,629,198)	5,074,692,873<br>(4,558,069,952–5,624,040,214)	4,297,524,654<br>(3,935,513,836–4,714,066,098)	3,955,991,436<br>(3,567,268,278–4,393,066,188)
Cape Verde	NCD	5,673,613,016<br>(5,025,262,563–6,355,672,994)	6,375,787,651<br>(5,458,388,214–7,341,193,079)	7,019,336,040<br>(5,703,336,575–8,403,661,724)	7,800,322,028<br>(6,457,186,687–9,197,883,920)	8,063,167,537<br>(7,065,353,805–9,157,680,842)	8,112,396,696<br>(6,884,805,687–9,442,020,805)
Cape Verde	Injuries	1,031,441,575<br>(853,235,262–1,207,588,800)	1,432,232,028<br>(1,172,657,579–1,692,552,059)	1,976,027,232<br>(1,462,311,100–2,537,658,994)	2,109,492,558<br>(1,612,580,100–2,663,956,588)	2,038,487,335<br>(1,718,603,543–2,293,132,887)	1,851,197,776<br>(1,504,972,459–2,268,889,436)
Chad	All causes	691,774,236,964<br>(661,392,253,039–725,076,083,445)	788,395,622,621<br>(748,548,736,736–828,508,328,660)	946,767,596,741<br>(893,561,663,748–1,001,072,957,436)	1,038,601,764,422<br>(957,454,053,520–1,131,881,627,127)	1,068,068,235,077<br>(957,835,136,050–1,203,292,251,036)	1,109,638,604,478<br>(961,219,779,810–1,290,960,507,069)
Chad	Group I	553,832,631,263<br>(520,901,242,641–585,172,368,854)	639,316,191,796<br>(600,405,571,803–673,503,432,602)	750,398,905,766<br>(706,628,143,677–796,075,288,420)	818,707,127,067<br>(755,411,685,089–883,465,293,224)	797,605,857,445<br>(713,320,416,179–888,313,619,637)	800,398,425,876<br>(703,626,515,248–914,277,786,046)

Appendix Table 3. DALYs for all causes and level 1 cause groups by location for 1990 - 2015, both sexes combined.

	Cause	1990	1995	2000	2005	2010	2015
Chad	NCD	95,154,676,959 (77,723,101,207–116,941,951,267)	109,019,831,602 (88,424,510,675–132,422,881,507)	138,778,273,969 (112,687,539,794–171,444,520,950)	161,185,492,952 (126,189,517,371–206,311,231,275)	197,551,166,429 (146,465,101,292–260,604,559,193)	228,515,804,319 (163,839,354,492–307,438,526,456)
Chad	Injuries	42,786,928,743 (30,331,284,707–60,596,476,134)	40,059,599,223 (29,220,310,843–54,669,987,909)	57,590,417,007 (44,484,883,282–74,113,800,764)	58,709,144,403 (43,434,748,211–76,123,251,692)	72,911,211,203 (53,344,233,225–97,088,560,748)	80,724,374,283 (58,897,063,059–109,803,959,327)
Cote d'Ivoire	All causes	1,067,365,872,527 (1,003,581,375,023–1,129,995,091,657)	1,322,480,358,969 (1,237,935,956,494–1,402,285,697,950)	1,487,062,470,820 (1,391,257,080,782–1,582,269,699,300)	1,506,151,931,958 (1,406,124,517,464–1,606,741,026,277)	1,433,843,576,987 (1,297,194,127,583–1,581,758,982,296)	1,397,914,253,294 (1,208,518,338,986–1,639,316,873,751)
Cote d'Ivoire	Group I	784,610,642,483 (737,056,244,508–830,505,099,354)	964,009,445,687 (905,871,363,316–1,021,681,315,819)	1,085,455,442,861 (1,019,256,830,274–1,154,275,681,112)	1,105,327,156,398 (1,036,250,262,855–1,173,253,097,317)	1,013,387,429,755 (935,374,740,157–1,091,919,247,451)	923,630,139,288 (823,455,657,175–1,036,811,102,833)
Cote d'Ivoire	NCD	223,075,749,542 (187,308,422,553–260,368,496,314)	282,012,726,374 (232,128,409,171–331,136,365,221)	316,794,639,768 (264,300,059,970–379,562,662,435)	322,551,516,628 (268,844,940,810–383,525,380,401)	341,087,678,255 (271,398,710,699–420,209,655,855)	387,630,387,768 (295,067,162,982–510,280,882,035)
Cote d'Ivoire	Injuries	59,679,480,502 (45,918,231,477–74,264,160,451)	76,458,186,907 (60,449,918,219–92,732,641,432)	84,812,388,190 (67,332,173,449–104,217,494,852)	78,273,258,931 (62,617,549,556–95,294,088,330)	79,368,468,978 (61,080,335,329–98,789,261,785)	86,653,726,239 (62,382,451,435–120,319,927,145)
The Gambia	All causes	62,636,396,366 (56,077,630,265–70,749,122,370)	66,919,294,220 (60,971,920,068–73,303,528,878)	68,718,111,678 (63,765,638,787–73,729,993,160)	70,765,549,284 (65,058,974,234–76,250,992,898)	72,279,808,330 (65,714,309,463–78,969,449,769)	73,316,423,854 (64,407,739,724–83,703,940,878)
The Gambia	Group I	44,843,833,906 (41,067,414,998–48,899,377,948)	46,953,615,282 (43,528,831,015–50,506,170,423)	46,873,159,605 (44,034,052,746–50,050,717,372)	45,737,206,671 (42,617,805,072–48,889,116,889)	44,552,489,946 (40,886,257,849–48,148,036,204)	42,160,609,636 (37,957,587,847–46,709,987,483)
The Gambia	NCD	14,285,031,026 (11,069,895,788–18,307,446,637)	16,108,624,601 (13,089,292,001–19,572,655,073)	17,737,157,132 (14,878,408,641–20,564,697,857)	20,469,861,667 (17,356,660,685–23,676,871,095)	22,991,927,438 (19,306,697,080–26,883,646,748)	26,159,001,514 (21,260,515,661–31,981,321,306)
The Gambia	Injuries	3,507,531,434 (2,634,746,605–4,605,364,017)	3,857,054,338 (3,059,573,682–4,864,280,631)	4,107,794,942 (3,460,812,801–4,832,674,195)	4,558,480,946 (3,863,250,652–5,296,555,260)	4,735,390,946 (3,958,853,278–5,723,954,196)	4,996,812,704 (3,987,543,832–6,336,830,165)
Ghana	All causes	1,031,599,820,544 (942,884,573,432–1,139,136,293,414)	1,047,632,864,230 (971,292,712,784–1,129,441,032,882)	1,112,515,893,370 (1,020,504,575,071–1,215,308,511,504)	1,182,104,918,517 (1,056,962,666,683–1,308,367,397,938)	1,194,020,056,360 (1,017,590,868,977–1,402,180,315,979)	1,137,705,390,006 (924,446,592,398–1,417,644,995,114)
Ghana	Group I	719,534,030,525 (668,909,948,049–770,068,453,106)	713,468,255,425 (673,239,843,314–755,552,988,356)	737,000,548,288 (693,032,399,854–785,535,301,072)	758,342,813,573 (704,242,180,125–815,948,822,415)	718,852,561,616 (649,522,522,896–799,248,403,731)	599,620,076,968 (519,666,631,369–698,696,756,114)
Ghana	NCD	251,879,163,610 (207,019,739,087–306,664,257,329)	270,928,023,391 (231,296,306,362–316,442,220,200)	311,263,396,072 (259,031,234,044–367,251,954,850)	353,270,640,125 (284,693,073,996–423,529,533,398)	400,547,559,595 (305,155,521,967–516,145,898,296)	454,529,728,201 (334,328,848,605–623,023,473,855)
Ghana	Injuries	60,186,626,409 (47,846,074,941–76,504,670,682)	63,236,585,414 (52,755,434,103–76,072,161,330)	64,251,949,011 (53,178,245,650–77,455,208,154)	70,491,464,819 (56,689,666,155–86,515,194,053)	74,619,935,149 (54,932,626,213–99,516,723,390)	83,555,584,838 (59,250,016,262–119,319,764,952)
Guinea	All causes	733,730,438,605 (701,577,491,556–766,568,648,344)	825,968,829,795 (790,707,546,405–864,462,069,638)	847,284,766,086 (805,319,386,372–891,392,726,919)	818,444,619,463 (770,018,978,636–869,199,586,305)	804,025,267,277 (736,321,225,197–871,845,237,341)	808,983,724,000 (701,393,015,444–934,317,039,558)
Guinea	Group I	566,716,701,653 (531,812,659,346–602,536,052,231)	621,611,442,889 (585,286,260,394–659,360,790,753)	626,362,854,626 (587,033,624,316–666,097,403,016)	597,274,868,254 (556,529,142,861–633,918,678,163)	556,834,722,031 (511,123,414,286–601,659,186,034)	530,639,354,346 (468,896,223,804–595,963,669,418)
Guinea	NCD	132,380,230,551 (108,601,693,469–164,567,277,290)	162,072,072,023 (133,868,422,758–195,113,101,968)	175,870,417,985 (144,829,131,790–208,447,142,491)	179,877,782,961 (149,477,133,522–214,787,472,644)	202,911,844,916 (168,414,321,866–240,995,066,714)	231,293,525,443 (177,695,816,452–293,423,863,255)
Guinea	Injuries	34,633,506,401 (22,751,101,965–46,985,892,963)	42,285,314,882 (29,579,768,274–55,323,927,171)	45,051,493,476 (32,821,539,348–58,929,689,779)	41,291,968,248 (32,462,850,453–51,505,916,963)	44,278,700,330 (34,620,451,553–54,667,947,047)	47,050,844,211 (34,703,569,400–62,490,858,654)
Guinea-Bissau	All causes	120,611,920,536 (103,860,766,153–153,059,130,933)	123,555,796,036 (104,716,467,105–163,989,136,678)	127,352,425,075 (104,991,680,737–171,777,844,807)	133,518,095,618 (105,726,361,968–186,404,515,539)	135,904,833,485 (106,071,343,764–193,868,976,236)	138,117,864,663 (105,541,095,471–221,591,812,892)
Guinea-Bissau	Group I	92,422,306,607 (82,411,828,867–107,441,369,228)	92,208,804,816 (81,485,640,935–109,962,589,200)	92,761,737,981 (79,922,544,014–111,769,786,546)	93,616,487,916 (78,240,100,216–115,989,550,322)	90,576,122,757 (75,191,140,701–113,857,715,706)	88,771,705,412 (71,641,737,476–118,559,408,660)
Guinea-Bissau	NCD	21,837,591,782 (13,717,118,006–37,138,038,075)	23,840,676,785 (14,883,015,602–41,762,031,136)	26,405,591,652 (16,202,876,957–46,777,558,350)	30,560,175,965 (18,155,745,168–55,503,570,806)	34,845,949,124 (20,820,948,255–64,692,466,970)	38,694,007,917 (22,751,542,409–79,433,058,702)
Guinea-Bissau	Injuries	6,352,022,148 (3,854,017,192–10,273,652,322)	7,506,314,435 (4,662,501,829–12,662,516,948)	8,185,095,443 (4,993,762,647–13,881,009,983)	9,341,431,738 (5,676,379,462–16,185,394,432)	10,482,761,604 (6,452,964,757–18,311,708,578)	10,652,151,335 (6,485,793,513–20,998,970,589)
Liberia	All causes	278,066,160,436 (256,648,979,890–301,248,971,963)	255,662,358,478 (240,362,982,452–271,436,749,040)	257,296,888,921 (242,655,948,637–273,057,990,225)	242,741,074,201 (227,294,233,412–259,783,661,931)	234,822,017,057 (213,849,181,692–256,765,423,244)	225,598,205,071 (196,430,085,822–259,053,547,010)
Liberia	Group I	201,784,721,535 (187,128,455,135–214,486,845,154)	195,108,212,336 (183,305,067,634–207,584,546,699)	201,110,786,526 (188,645,760,244–214,349,710,970)	182,842,580,584 (171,597,292,021–194,734,760,558)	162,916,525,348 (150,372,638,723–176,606,002,103)	146,389,803,074 (130,944,519,928–163,899,875,946)
Liberia	NCD	40,268,665,702 (31,727,905,487–51,636,953,550)	35,262,655,069 (28,086,572,610–44,783,902,993)	43,573,320,428 (35,062,683,898–53,659,732,259)	48,141,711,810 (39,981,371,888–58,062,467,474)	58,684,069,342 (47,635,892,517–70,367,866,686)	65,650,608,649 (51,644,102,444–82,549,694,098)
Liberia	Injuries	36,012,773,200 (20,313,771,558–53,695,504,941)	25,291,491,073 (15,210,469,135–36,355,567,409)	12,612,781,967 (9,815,292,502–15,877,802,977)	11,756,781,807 (9,602,508,952–14,573,349,830)	13,221,422,367 (10,578,772,608–16,554,129,151)	13,557,793,349 (10,209,629,085–17,731,527,121)
Mali	All causes	1,098,314,432,064 (1,056,818,704,637–1,137,580,305,634)	1,156,668,183,349 (1,110,572,692,664–1,201,101,866,394)	1,213,167,530,995 (1,160,850,090,139–1,268,693,366,017)	1,226,647,552,343 (1,160,472,360,659–1,293,392,777,152)	1,249,400,846,031 (1,163,035,786,993–1,341,606,736,054)	1,276,219,589,438 (1,139,871,375,393–1,452,680,324,714)
Mali	Group I	870,122,697,415 (812,782,814,052–918,335,349,513)	913,200,485,217 (857,571,980,079–963,341,391,818)	942,667,492,930 (884,764,560,136–998,909,522,814)	960,617,535,043 (900,002,209,598–1,022,373,409,008)	944,400,998,600 (868,162,411,071–1,023,111,648,011)	938,015,499,067 (830,572,091,493–1,051,118,196,822)
Mali	NCD	172,525,454,026 (141,494,856,875–213,733,646,649)	185,229,744,136 (152,799,070,868–230,189,717,767)	194,460,911,836 (156,735,276,536–242,831,114,305)	201,840,654,401 (161,951,839,619–251,596,026,101)	232,835,659,186 (188,193,058,855–287,858,656,126)	261,690,297,593 (206,282,475,469–332,160,607,900)
Mali	Injuries	55,666,280,623 (39,625,396,646–73,396,090,209)	58,237,953,997 (41,968,704,517–76,673,852,206)	76,039,126,229 (56,677,294,726–101,937,470,186)	64,189,362,900 (48,875,571,598–82,952,722,674)	72,164,188,245 (54,933,521,201–93,075,733,686)	76,513,792,778 (59,422,555,469–98,158,727,262)
Mauritania	All causes	136,560,862,598 (127,908,811,248–146,196,465,405)	143,159,236,411 (134,234,917,733–153,419,315,769)	149,313,615,770 (138,480,867,402–160,297,781,616)	154,197,898,066 (141,322,863,551–168,158,828,309)	149,703,991,193 (134,212,285,655–166,659,609,019)	149,190,701,627 (127,290,924,719–173,430,331,591)
Mauritania	Group I	92,614,461,351 (86,539,188,896–99,967,054,004)	96,463,234,956 (89,672,763,382–104,665,348,806)	97,463,223,704 (90,362,987,315–105,368,954,770)	97,991,911,336 (90,278,756,404–106,767,527,149)	88,442,038,039 (79,508,220,924–97,406,965,857)	79,765,540,235 (70,342,666,498–90,632,539,454)
Mauritania	NCD	33,928,326,530 (28,773,707,259–39,561,866,185)	37,608,823,800 (31,793,861,501–43,900,878,863)	41,498,321,727 (34,603,847,875–48,747,484,551)	45,214,043,377 (36,948,741,926–53,615,817,665)	50,272,897,715 (40,352,098,664–60,030,984,092)	57,741,044,917 (45,299,169,121–72,746,938,277)
Mauritania	Injuries	10,018,074,717 (6,842,004,411–13,092,893,355)	9,087,177,654 (6,402,483,331–11,338,847,608)	10,352,070,339 (8,416,645,139–12,429,675,191)	10,991,943,353 (8,684,922,841–13,593,160,956)	10,989,055,439 (8,436,073,021–14,329,448,131)	11,684,116,475 (8,730,044,804–15,933,392,501)
Niger	All causes	1,302,568,231,848 (1,262,365,351,522–1,348,100,891,565)	1,391,789,778,832 (1,341,813,176,128–1,448,827,984,549)	1,420,545,231,600 (1,363,776,857,164–1,479,889,034,726)	1,376,336,979,462 (1,307,370,216,984–1,446,586,198,802)	1,340,044,613,868 (1,243,472,371,360–1,438,261,064,664)	1,395,604,281,920 (1,247,170,301,085–1,569,953,255,226)
Niger	Group I	1,097,990,094,000 (1,035,002,102,058–1,155,716,020,572)	1,166,266,961,291 (1,102,013,487,609–1,227,418,649,786)	1,172,510,568,506 (1,106,107,849,120–1,237,840,228,884)	1,103,083,404,002 (1,038,936,245,677–1,164,546,240,796)	1,037,509,888,341 (963,729,261,297–1,114,465,234,440)	1,017,840,405,908 (914,757,445,910–1,136,348,462,690)
Niger	NCD	144,278,043,813 (113,685,175,594–188,991,408,487)	160,915,623,181 (127,112,002,579–209,340,136,156)	180,116,672,200 (142,858,692,876–229,139,800,890)	204,393,644,346 (165,278,100,669–253,648,267,261)	232,780,173,381 (187,026,948,462–287,755,985,243)	287,242,809,757 (224,174,819,156–366,481,041,187)
Niger	Injuries	60,300,094,036 (38,031,844,781–84,309,123,922)	64,607,194,360 (43,239,671,987–87,709,063,762)	67,917,990,893 (48,681,586,171–90,689,688,756)	68,859,931,113 (52,531,474,250–89,368,271,197)	69,754,552,146 (53,431,777,421–88,176,132,131)	90,521,066,256 (68,227,799,615–115,983,363,346)
Nigeria	All causes	10,191,076,676,033 (9,512,556,537,653–11,001,577,526,005)	10,966,068,142,275 (10,405,110,771,190–11,606,633,125,374)	11,862,801,902,565 (11,281,365,803,021–12,509,583,210,355)	12,247,031,953,193 (11,557,310,483,571–12,984,264,173,078)	11,536,172,152,246 (10,775,259,264,910–12,403,062,235,625)	10,685,704,791,634 (9,570,581,980,831–12,007,203,626,486)
Nigeria	Group I	7,941,990,607,021 (7,384,510,901,112–8,490,236,183,174)	8,481,270,277,517 (7,935,604,648,066–8,991,505,251,297)	9,128,732,333,500 (8,575,867,453,634–9,660,200,860,575)	9,505,290,535,928 (8,902,646,649,846–10,000,997,771,589)	8,631,734,717,140 (8,054,190,487,070–9,219,087,231,691)	7,469,301,404,104 (6,699,693,495,456–8,305,672,349,393)
Nigeria	NCD	1,784,840,734,128 (1,396,749,956,092–2,270,618,399,787)	1,978,435,250,366 (1,614,594,492,803–2,419,784,992,040)	2,194,752,632,048 (1,817,104,726,824–2,673,510,109,757)	2,252,838,474,217 (1,871,878,609,458–2,749,770,505,361)	2,398,273,229,683 (1,948,170,981,043–2,875,478,616,821)	2,621,772,782,522 (2,125,596,619,921–3,213,253,227,141)
Nigeria	Injuries	464,245,334,884 (328,792,932,599–624,909,481,910)	506,362,614,391 (381,441,860,563–659,413,321,527)	539,316,937,017 (411,487,430,999–685,983,590,869)	488,902,943,048 (377,006,183,759–624,029,550,242)	506,164,205,422 (404,858,932,818–623,391,347,016)	594,630,605,008 (475,417,220,470–743,666,527,364)
Sao Tome and Principe	All causes	6,849,916,916 (6,426,209,932–7,326,945,548)	6,936,701,261 (6,418,253,915–7,487,394,633)	6,885,724,512 (6,363,230,410–7,462,787,919)	6,682,593,111 (6,003,437,836–7,424,380,703)	6,438,366,699 (5,504,231,316–7,493,138,106)	6,402,274,215 (5,136,371,752–7,905,928,238)

Appendix Table 3. DALYs for all causes and level 1 cause groups by location for 1990 - 2015, both sexes combined.

	Cause	1990	1995	2000	2005	2010	2015
Sao Tome and Principe	Group I	4,164,836,958<br>(3,860,339,650–4,571,655,571)	4,033,483,547<br>(3,720,302,514–4,396,943,362)	3,709,102,693<br>(3,402,150,332–4,029,122,940)	3,308,698,338<br>(2,967,572,532–3,688,872,131)	2,882,823,888<br>(2,518,559,216–3,308,502,644)	2,619,890,888<br>(2,196,613,423–3,098,902,258)
Sao Tome and Principe	NCD	2,251,112,928<br>(1,949,051,675–2,575,535,546)	2,442,995,741<br>(2,088,329,535–2,787,117,741)	2,692,871,675<br>(2,348,251,317–3,035,300,910)	2,886,944,873<br>(2,489,770,172–3,338,637,671)	3,072,677,584<br>(2,480,107,717–3,749,574,463)	3,296,302,785<br>(2,480,400,868–4,323,277,580)
Sao Tome and Principe	Injuries	433,967,030<br>(320,663,440–529,040,343)	460,221,972<br>(364,211,672–557,055,141)	483,750,143<br>(400,871,477–589,016,571)	486,949,900<br>(393,632,609–597,882,224)	482,865,227<br>(367,662,501–640,338,561)	486,080,541<br>(337,013,684–690,636,611)
Senegal	All causes	607,613,560,064<br>(578,019,633,009–638,706,916,341)	650,478,125,234<br>(613,227,777,099–686,276,949,233)	663,474,029,018<br>(622,359,223,476–706,191,975,847)	635,415,037,595<br>(579,709,003,720–702,459,966,061)	614,960,988,543<br>(531,932,425,313–724,004,404,098)	618,616,733,805<br>(508,791,947,911–773,785,557,577)
Senegal	Group I	451,995,525,346<br>(426,962,604,609–473,340,316,554)	474,939,275,321<br>(448,874,411,372–496,691,556,051)	472,645,877,339<br>(445,439,723,367–498,511,479,942)	422,148,654,838<br>(390,219,549,650–454,515,907,875)	377,549,651,075<br>(340,062,428,110–426,925,611,394)	343,494,217,295<br>(296,597,545,009–409,586,244,248)
Senegal	NCD	121,090,178,925<br>(102,015,921,460–144,081,715,912)	136,248,440,903<br>(114,941,965,729–159,062,667,287)	151,387,102,646<br>(128,275,747,783–177,096,405,804)	170,996,762,596<br>(140,088,858,369–206,068,241,093)	193,905,310,385<br>(148,484,298,410–244,827,204,462)	227,180,834,682<br>(168,197,356,957–302,789,200,360)
Senegal	Injuries	34,527,855,793<br>(27,781,670,487–43,057,790,561)	39,290,409,010<br>(32,399,156,050–47,534,696,825)	39,441,049,033<br>(32,570,383,983–47,459,606,890)	42,269,620,162<br>(34,082,356,647–52,645,809,446)	43,506,027,084<br>(32,532,807,764–58,895,776,315)	47,941,681,828<br>(34,377,058,861–68,955,610,741)
Sierra Leone	All causes	488,374,924,247<br>(460,807,026,699–520,634,418,507)	481,191,685,250<br>(456,806,343,616–509,944,741,155)	471,790,361,586<br>(450,398,724,885–494,979,227,109)	501,567,235,608<br>(477,992,565,259–526,506,597,148)	478,753,722,152<br>(449,769,727,537–512,806,558,718)	447,888,500,443<br>(400,234,009,805–505,008,916,232)
Sierra Leone	Group I	362,552,174,483<br>(325,712,622,110–391,892,764,646)	343,998,226,045<br>(312,433,667,728–369,036,465,877)	347,678,417,884<br>(315,588,867,619–372,159,484,179)	363,954,321,046<br>(332,482,978,289–388,305,882,660)	328,975,938,522<br>(297,485,129,478–354,481,729,850)	288,767,158,864<br>(255,569,887,820–319,580,275,273)
Sierra Leone	NCD	102,067,349,966<br>(75,799,123,785–138,391,924,128)	93,949,661,128<br>(70,971,200,755–128,504,870,012)	95,063,250,040<br>(72,899,258,586–130,181,768,282)	112,795,523,483<br>(89,967,151,650–146,662,530,200)	123,915,393,975<br>(98,532,853,316–157,002,645,043)	132,568,027,224<br>(101,397,376,959–172,617,554,589)
Sierra Leone	Injuries	23,755,399,798<br>(14,578,898,434–32,857,750,708)	43,243,798,077<br>(27,934,558,585–61,543,010,705)	29,048,693,661<br>(20,533,184,136–38,954,308,219)	24,817,391,080<br>(17,972,315,051–32,029,838,810)	25,862,389,654<br>(18,923,551,579–33,545,788,362)	26,553,314,355<br>(19,118,719,973–35,764,436,250)
Togo	All causes	302,846,226,384<br>(286,993,193,458–320,604,898,827)	324,816,613,928<br>(305,745,525,897–345,895,380,514)	359,691,864,010<br>(335,058,196,739–386,239,965,490)	396,381,452,817<br>(365,190,985,779–427,380,015,048)	401,771,681,300<br>(364,107,670,019–439,323,772,350)	384,359,428,363<br>(331,630,976,135–444,810,462,621)
Togo	Group I	216,108,705,695<br>(203,878,877,367–227,601,345,854)	228,744,551,058<br>(215,512,573,983–241,520,324,153)	251,501,714,357<br>(236,673,812,933–269,264,552,373)	275,714,530,479<br>(257,376,060,256–293,922,299,169)	270,743,163,159<br>(249,669,749,885–292,507,359,881)	237,018,912,890<br>(212,140,822,774–264,209,608,488)
Togo	NCD	70,179,977,438<br>(60,095,635,795–82,015,851,986)	77,361,190,817<br>(65,430,998,992–90,807,889,793)	87,395,152,620<br>(72,500,340,924–103,030,231,211)	95,854,441,772<br>(79,410,169,218–113,582,765,703)	107,226,957,558<br>(87,526,380,871–129,117,560,362)	122,019,248,291<br>(95,664,861,739–156,261,780,138)
Togo	Injuries	16,557,543,251<br>(13,234,607,310–20,062,251,547)	18,710,872,053<br>(15,371,552,063–22,766,863,602)	20,794,997,033<br>(16,876,455,637–25,048,014,994)	24,812,480,566<br>(20,068,282,899–30,044,981,397)	23,801,560,583<br>(18,526,818,222–29,298,864,311)	25,321,267,182<br>(18,532,356,478–34,277,060,797)
Eastern Sub-Saharan Africa	All causes	19,564,992,650,389<br>(18,863,863,264,747–20,323,987,720,878)	20,987,359,389,119<br>(20,170,967,700,785–21,807,151,690,345)	22,111,513,129,037<br>(21,199,201,856,553–22,991,965,508,127)	20,834,554,468,750<br>(19,928,154,510,170–21,845,581,085,497)	19,304,894,242,593<br>(18,150,410,961,361–20,565,031,385,339)	18,658,142,404,861<br>(17,221,026,254,795–20,321,419,553,877)
Eastern Sub-Saharan Africa	Group I	14,553,761,133,390<br>(14,085,366,355,358–15,070,762,369,065)	15,946,908,799,616<br>(15,430,989,150,975–16,472,557,848,215)	16,434,384,881,725<br>(15,889,628,876,413–17,016,584,415,993)	15,105,025,288,404<br>(14,570,020,689,993–15,710,301,782,295)	12,936,640,398,574<br>(12,378,574,118,997–13,539,731,061,865)	11,340,501,743,852<br>(10,693,374,006,466–12,084,931,066,881)
Eastern Sub-Saharan Africa	NCD	3,524,548,390,887<br>(3,135,749,295,990–3,923,358,043,415)	3,824,845,955,610<br>(3,392,977,960,717–4,279,157,042,331)	4,147,775,056,005<br>(3,685,888,527,767–4,614,821,761,191)	4,520,275,318,044<br>(4,021,982,065,900–5,066,196,904,344)	5,064,623,343,126<br>(4,416,041,510,992–5,759,576,097,718)	5,868,680,667,429<br>(5,046,347,412,542–6,746,785,338,745)
Eastern Sub-Saharan Africa	Injuries	1,486,683,126,111<br>(1,214,026,182,951–1,753,010,344,275)	1,215,604,633,893<br>(1,007,699,697,339–1,393,325,818,666)	1,529,353,191,308<br>(1,271,970,249,149–1,802,479,229,488)	1,209,253,862,301<br>(1,080,464,249,114–1,338,886,688,910)	1,303,630,500,894<br>(1,164,166,713,486–1,463,119,604,832)	1,448,959,993,581<br>(1,269,746,770,694–1,681,836,068,490)
Burundi	All causes	686,521,100,217<br>(608,309,155,585–776,302,508,898)	750,997,477,593<br>(689,113,119,165–817,989,414,263)	728,927,547,954<br>(675,654,591,349–788,955,864,650)	613,425,902,168<br>(568,979,358,637–661,934,261,041)	562,192,399,564<br>(501,506,985,588–631,568,290,133)	612,226,976,647<br>(494,684,188,875–751,945,092,185)
Burundi	Group I	503,783,668,984<br>(453,189,296,433–559,474,298,863)	549,303,515,552<br>(505,314,387,395–595,904,558,253)	540,532,040,943<br>(501,343,697,442–583,802,400,041)	438,900,929,904<br>(406,837,931,189–472,393,632,753)	377,210,824,788<br>(338,337,980,966–422,147,878,604)	380,964,208,049<br>(305,313,541,821–472,897,951,211)
Burundi	NCD	138,810,192,811<br>(105,509,228,696–172,702,605,851)	146,256,908,678<br>(120,515,133,581–172,297,790,133)	133,817,432,354<br>(112,596,993,793–156,964,119,487)	132,334,629,154<br>(112,858,832,957–152,833,432,747)	144,967,463,959<br>(117,373,384,910–174,471,171,711)	180,107,403,482<br>(139,153,195,986–230,537,961,257)
Burundi	Injuries	43,927,238,422<br>(29,401,887,909–58,406,242,580)	55,437,053,363<br>(40,182,875,461–70,865,590,036)	54,578,074,657<br>(41,376,556,966–70,240,775,447)	42,190,343,111<br>(33,934,538,069–52,982,472,062)	40,014,110,816<br>(30,863,561,352–53,271,497,910)	51,155,365,116<br>(36,736,163,518–70,094,399,673)
Comoros	All causes	30,982,894,538<br>(27,372,524,757–35,888,301,768)	30,697,454,117<br>(27,490,944,914–34,408,196,633)	29,385,296,416<br>(26,204,287,634–32,900,754,640)	27,309,428,396<br>(23,442,011,496–31,675,103,945)	26,704,873,669<br>(22,352,772,884–31,696,511,939)	27,502,633,927<br>(22,100,258,831–34,029,420,177)
Comoros	Group I	20,806,022,992<br>(18,799,235,066–23,128,762,666)	19,792,130,278<br>(17,826,731,605–22,057,289,424)	18,467,886,426<br>(16,094,167,750–21,142,812,616)	16,347,611,138<br>(13,506,798,847–19,650,368,730)	14,668,362,705<br>(11,796,058,285–18,189,709,337)	13,830,004,516<br>(10,780,001,061–17,654,446,345)
Comoros	NCD	7,952,504,889<br>(6,135,292,745–10,175,604,679)	8,616,762,699<br>(7,141,274,135–10,344,351,942)	8,769,995,164<br>(7,500,855,460–10,176,125,186)	8,847,648,805<br>(7,377,134,799–10,392,850,409)	9,913,467,525<br>(8,257,693,097–11,633,407,721)	11,382,853,102<br>(8,889,496,513–14,140,119,548)
Comoros	Injuries	2,224,366,658<br>(1,497,910,123–3,079,126,942)	2,288,561,140<br>(1,673,524,886–2,984,218,627)	2,147,414,826<br>(1,612,826,488–2,728,492,307)	2,114,168,453<br>(1,580,618,588–2,692,493,205)	2,123,043,439<br>(1,647,751,755–2,856,230,305)	2,289,776,308<br>(1,618,202,435–3,316,587,092)
Djibouti	All causes	36,120,310,283<br>(32,147,349,257–41,373,607,608)	40,859,315,605<br>(35,471,927,642–48,321,859,069)	41,944,693,085<br>(34,530,760,106–53,207,129,416)	41,878,558,344<br>(33,603,903,005–53,796,735,344)	40,707,227,625<br>(31,761,252,910–53,836,095,949)	40,179,587,780<br>(30,798,615,906–53,637,945,513)
Djibouti	Group I	22,994,202,355<br>(20,889,695,839–25,518,150,196)	25,669,542,219<br>(22,977,677,552–29,251,660,655)	25,709,212,769<br>(22,248,138,702–29,909,023,242)	24,116,104,549<br>(20,542,343,089–28,851,396,911)	21,426,607,040<br>(17,786,595,614–26,080,552,110)	19,326,591,075<br>(15,660,395,608–23,806,489,694)
Djibouti	NCD	10,021,914,708<br>(7,948,630,556–12,540,608,439)	11,583,400,709<br>(8,776,846,346–15,166,347,774)	12,648,475,818<br>(9,041,869,119–17,799,729,470)	14,047,829,112<br>(9,959,139,398–20,160,281,776)	15,449,441,778<br>(10,889,087,054–22,466,040,414)	16,903,552,835<br>(11,842,269,543–24,636,766,413)
Djibouti	Injuries	3,104,193,219<br>(2,222,422,252–4,076,866,215)	3,606,372,677<br>(2,505,192,053–4,978,956,171)	3,587,004,498<br>(2,192,406,035–5,528,885,747)	3,714,624,682<br>(2,447,797,935–5,880,671,478)	3,831,178,807<br>(2,506,280,575–6,112,889,561)	3,949,443,870<br>(2,503,212,881–6,474,666,287)
Eritrea	All causes	276,155,627,213<br>(258,344,693,588–295,196,848,896)	213,679,844,312<br>(195,735,542,174–232,999,413,700)	524,160,843,398<br>(317,570,544,964–765,186,219,348)	230,937,121,606<br>(190,122,841,346–286,683,750,503)	251,158,789,042<br>(198,496,073,164–328,503,080,122)	258,070,451,090<br>(199,600,446,273–348,142,066,654)
Eritrea	Group I	204,538,963,711<br>(191,200,310,492–221,694,129,910)	152,791,487,071<br>(141,176,950,919–166,208,005,222)	133,875,906,564<br>(118,173,197,689–151,639,418,353)	143,869,148,388<br>(121,656,451,062–172,963,471,011)	151,362,043,818<br>(122,227,203,881–189,555,279,633)	146,736,549,955<br>(116,246,703,595–187,617,996,557)
Eritrea	NCD	54,099,049,343<br>(44,326,977,381–62,896,184,644)	46,641,115,802<br>(38,236,344,667–55,650,012,183)	51,877,402,996<br>(39,872,698,060–65,955,181,691)	65,521,740,507<br>(47,973,646,321–87,485,730,808)	76,122,525,471<br>(54,339,392,594–105,099,128,874)	86,321,670,603<br>(61,360,484,727–126,614,643,295)
Eritrea	Injuries	17,517,614,160<br>(11,505,188,791–23,077,326,311)	14,247,241,439<br>(9,748,005,406–18,740,702,527)	338,407,533,838<br>(135,124,349,955–580,555,457,666)	21,546,232,710<br>(16,050,812,083–29,935,545,091)	23,674,219,753<br>(16,922,765,845–35,156,590,991)	25,012,230,531<br>(16,897,380,883–37,924,483,757)
Ethiopia	All causes	6,257,295,750,203<br>(5,919,281,097,568–6,600,124,258,392)	6,251,083,887,968<br>(5,948,272,551,284–6,549,640,595,890)	6,082,754,308,646<br>(5,755,569,255,867–6,413,767,888,982)	5,433,467,247,300<br>(5,098,001,939,390–5,779,357,217,041)	4,510,415,902,521<br>(4,040,900,663,575–5,031,375,512,819)	4,114,925,588,940<br>(3,464,260,349,806–4,921,110,576,804)
Ethiopia	Group I	4,483,529,612,097<br>(4,227,375,506,204–4,802,552,887,730)	4,736,740,065,148<br>(4,464,350,818,073–5,079,599,377,617)	4,541,166,968,082<br>(4,285,025,571,185–4,836,381,148,053)	3,835,575,141,754<br>(3,611,789,436,525–4,075,232,611,773)	2,856,684,877,762<br>(2,602,915,658,656–3,135,348,201,363)	2,295,620,821,592<br>(1,990,258,013,964–2,646,661,423,685)
Ethiopia	NCD	1,092,952,775,201<br>(914,197,516,422–1,257,679,582,238)	1,145,546,410,115<br>(963,280,585,731–1,321,157,917,188)	1,190,295,468,869<br>(1,008,712,437,750–1,366,282,716,759)	1,259,677,254,782<br>(1,084,126,730,799–1,439,700,175,644)	1,329,094,158,282<br>(1,093,474,685,375–1,605,995,180,236)	1,488,493,437,637<br>(1,143,834,652,297–1,900,257,855,906)
Ethiopia	Injuries	680,813,362,905<br>(449,886,313,660–927,346,137,490)	368,797,412,705<br>(232,341,458,729–479,640,115,469)	351,291,871,694<br>(242,570,004,603–450,270,503,949)	338,214,850,764<br>(265,444,967,593–408,308,842,784)	324,636,866,477<br>(254,681,569,688–405,324,219,795)	330,811,329,711<br>(237,915,935,609–463,920,438,931)
Kenya	All causes	1,383,069,297,653<br>(1,304,450,357,504–1,472,510,489,487)	1,744,229,556,606<br>(1,644,097,333,194–1,855,673,926,527)	2,126,224,905,397<br>(2,006,982,132,192–2,265,597,608,407)	2,210,748,061,413<br>(2,079,305,088,326–2,368,527,440,315)	1,966,171,483,270<br>(1,817,914,270,512–2,137,663,043,592)	1,966,676,536,944<br>(1,790,107,871,358–2,156,855,674,799)
Kenya	Group I	982,959,293,976<br>(939,644,054,742–1,035,936,694,054)	1,289,134,934,955<br>(1,232,657,475,303–1,360,022,397,271)	1,620,594,349,171<br>(1,546,207,889,386–1,707,525,101,030)	1,646,340,856,309<br>(1,570,043,692,536–1,744,948,463,690)	1,326,632,575,459<br>(1,246,760,998,557–1,448,457,529,350)	1,229,210,774,945<br>(1,142,587,950,232–1,339,630,682,941)
Kenya	NCD	308,981,184,344<br>(268,817,972,431–352,716,607,244)	357,276,385,227<br>(309,719,786,660–408,102,467,289)	401,587,172,299<br>(345,497,684,155–461,499,907,513)	452,459,829,334<br>(390,240,822,239–523,719,237,305)	511,714,610,354<br>(439,771,411,924–585,340,133,282)	594,995,236,664<br>(507,166,529,146–684,358,699,546)
Kenya	Injuries	91,128,819,332<br>(79,079,893,400–105,416,315,637)	97,818,236,424<br>(86,293,077,704–111,204,558,674)	104,043,383,927<br>(92,246,934,112–118,397,467,508)	111,947,375,770<br>(99,443,413,644–126,740,657,008)	127,824,297,458<br>(113,444,456,914–145,697,666,192)	142,470,525,335<br>(123,425,694,299–163,895,242,946)

Appendix Table 3. DALYs for all causes and level 1 cause groups by location for 1990 - 2015, both sexes combined.

	Cause	1990	1995	2000	2005	2010	2015
Madagascar	All causes	995,184,048,606 (936,076,438,255–1,052,939,089,605)	1,075,678,743,233 (1,009,814,463,008–1,143,510,851,433)	1,058,288,920,086 (986,200,053,066–1,136,343,812,998)	1,011,691,123,208 (927,692,819,980–1,108,790,521,176)	1,060,532,928,117 (913,543,169,004–1,221,022,672,173)	1,106,864,138,438 (895,239,113,461–1,361,526,242,993)
Madagascar	Group I	712,324,647,673 (666,934,813,986–767,496,920,393)	766,927,006,516 (710,307,480,762–835,150,463,254)	722,211,093,480 (665,348,139,880–810,026,606,366)	645,974,642,723 (589,263,348,752–733,576,152,049)	637,864,738,384 (553,356,165,127–741,831,760,971)	614,265,043,258 (509,203,676,469–752,297,395,317)
Madagascar	NCD	231,664,603,962 (198,717,265,656–259,352,087,912)	255,453,962,098 (210,631,762,271–291,234,513,912)	280,133,605,084 (221,013,227,630–324,315,882,742)	310,153,831,174 (244,953,648,570–365,150,760,086)	360,290,033,143 (278,375,313,739–441,875,522,462)	422,306,460,735 (313,454,713,248–556,373,212,049)
Madagascar	Injuries	51,194,796,971 (36,425,425,683–65,454,722,351)	53,297,774,619 (37,409,857,572–68,247,089,769)	55,944,221,522 (38,561,368,307–72,116,693,602)	55,562,649,310 (40,427,991,678–70,371,467,863)	62,378,156,591 (44,988,824,247–81,894,910,612)	70,292,634,445 (48,016,295,184–101,135,961,298)
Malawi	All causes	1,185,053,004,023 (1,122,004,507,533–1,248,935,012,947)	1,218,675,049,524 (1,140,767,094,936–1,313,331,632,753)	1,298,401,379,293 (1,205,217,478,802–1,395,112,981,348)	1,214,559,248,472 (1,110,408,253,902–1,321,030,500,520)	1,088,988,893,579 (1,000,954,890,214–1,185,446,426,221)	998,443,364,089 (870,644,464,558–1,154,392,798,274)
Malawi	Group I	970,675,426,738 (916,484,726,717–1,027,304,710,094)	1,001,084,902,246 (935,855,521,180–1,073,276,897,713)	1,055,317,438,777 (977,498,666,961–1,137,090,853,840)	964,560,778,481 (888,629,804,825–1,047,589,560,948)	819,145,761,853 (762,880,666,382–884,159,947,386)	685,498,167,587 (602,982,140,928–784,519,970,286)
Malawi	NCD	160,786,970,040 (133,490,408,927–192,635,464,597)	167,120,051,035 (136,043,952,244–203,653,054,682)	189,577,090,984 (151,977,179,824–234,062,261,357)	199,632,589,213 (163,423,866,411–242,764,750,999)	217,929,646,124 (179,475,196,581–258,495,125,082)	253,384,419,325 (197,574,817,042–318,813,058,828)
Malawi	Injuries	53,590,607,246 (37,211,581,280–71,096,792,485)	50,470,096,244 (36,018,729,218–67,107,600,843)	53,506,849,532 (39,572,649,493–69,652,525,985)	50,365,880,777 (39,326,750,498–64,413,899,278)	51,913,485,603 (40,764,564,922–65,760,061,962)	59,560,777,177 (44,302,336,360–78,883,660,658)
Mozambique	All causes	1,590,215,556,708 (1,524,280,323,817–1,661,423,379,962)	1,693,719,402,222 (1,619,054,356,259–1,773,611,209,084)	1,800,412,744,865 (1,703,122,890,423–1,912,252,861,822)	1,861,117,688,680 (1,715,724,422,471–2,016,105,831,767)	1,865,452,476,050 (1,672,564,346,990–2,076,266,409,442)	1,794,290,597,625 (1,541,020,074,124–2,103,986,744,175)
Mozambique	Group I	1,266,470,308,840 (1,202,668,459,085–1,325,960,032,342)	1,339,992,788,105 (1,276,369,042,362–1,408,632,057,599)	1,392,996,931,009 (1,324,873,290,819–1,478,394,098,342)	1,426,777,936,900 (1,330,019,735,073–1,538,586,806,423)	1,359,167,069,228 (1,244,566,326,518–1,493,372,436,657)	1,216,491,374,516 (1,075,120,331,158–1,366,835,328,316)
Mozambique	NCD	233,081,313,040 (198,741,497,909–271,761,373,675)	269,054,233,604 (228,858,141,448–309,073,071,898)	314,083,100,679 (266,636,874,404–362,398,357,959)	345,706,279,914 (287,662,534,420–413,356,056,073)	405,742,485,709 (320,315,484,012–503,062,794,062)	466,814,783,310 (347,640,573,073–616,079,627,505)
Mozambique	Injuries	90,663,934,828 (71,735,148,517–118,689,137,010)	84,672,380,512 (68,545,406,962–107,344,172,834)	93,332,713,177 (77,663,820,985–109,529,110,947)	88,633,471,867 (70,790,916,698–108,039,220,904)	100,542,921,114 (75,326,929,839–129,704,353,941)	110,984,439,799 (76,738,221,523–158,123,239,119)
Rwanda	All causes	711,162,842,296 (671,067,628,493–757,071,166,830)	810,615,110,440 (753,257,482,592–856,391,480,973)	769,431,052,366 (724,100,195,492–816,095,703,037)	581,914,937,235 (541,148,948,966–626,134,800,690)	481,711,851,519 (427,853,507,226–539,614,246,804)	483,540,862,813 (407,992,935,348–581,952,911,388)
Rwanda	Group I	498,329,367,389 (464,257,814,742–533,996,179,286)	555,837,861,273 (516,745,009,590–597,482,686,770)	546,098,267,133 (510,625,236,927–583,159,787,483)	394,817,689,475 (368,290,242,618–423,824,264,188)	286,490,005,955 (257,263,808,869–317,809,927,746)	259,665,594,034 (220,838,805,684–307,163,073,849)
Rwanda	NCD	140,591,058,879 (118,898,683,474–163,302,766,067)	147,508,222,639 (127,492,210,716–170,636,781,593)	153,901,918,382 (131,877,460,400–178,629,415,323)	132,399,652,109 (111,892,327,696–153,766,410,138)	143,370,607,120 (118,489,875,170–170,257,813,825)	169,350,336,978 (133,914,508,905–214,589,248,540)
Rwanda	Injuries	72,242,416,029 (53,475,735,962–93,937,313,002)	107,269,026,528 (71,313,528,324–136,598,587,395)	69,430,866,851 (53,417,595,457–87,159,163,973)	54,697,595,651 (43,443,485,162–69,606,795,215)	51,851,238,444 (40,059,760,521–67,581,315,642)	54,524,931,801 (40,238,003,324–74,606,623,534)
Somalia	All causes	752,680,558,333 (617,621,962,967–979,059,661,544)	740,473,288,792 (603,505,250,822–956,539,542,174)	774,838,513,174 (621,730,823,474–1,020,888,535,174)	796,414,740,079 (627,783,665,620–1,109,671,703,072)	909,075,914,126 (686,110,152,768–1,276,994,038,612)	855,465,488,597 (656,474,129,937–1,255,984,650,857)
Somalia	Group I	550,517,699,232 (473,205,883,383–665,403,930,261)	555,770,683,248 (479,042,029,096–669,672,805,049)	577,982,678,574 (490,165,728,718–715,171,166,702)	586,970,460,673 (488,464,275,014–762,816,827,112)	651,133,111,270 (501,238,828,528–871,168,173,155)	576,251,470,510 (460,106,174,542–787,215,496,882)
Somalia	NCD	135,076,453,342 (82,634,445,594–218,887,577,369)	131,307,894,731 (80,258,320,605–208,753,034,309)	141,496,398,751 (86,861,589,542–225,300,213,146)	152,185,492,793 (93,547,119,875–254,159,463,739)	176,557,443,700 (110,244,612,154–309,684,785,040)	192,655,934,856 (120,396,580,203–332,705,702,852)
Somalia	Injuries	67,086,405,759 (45,123,301,396–97,225,551,368)	53,394,710,814 (34,558,294,850–85,342,405,765)	55,359,435,849 (33,437,385,185–90,712,498,016)	57,258,786,614 (34,572,308,632–95,567,000,320)	81,385,359,155 (53,768,414,471–136,539,950,667)	86,558,083,230 (56,944,929,616–135,888,808,984)
South Sudan	All causes	668,584,085,381 (569,079,587,156–839,857,262,015)	582,119,685,974 (486,354,228,698–753,985,946,794)	582,560,206,588 (469,749,958,517–807,025,932,546)	637,611,477,310 (501,638,432,643–936,002,375,597)	735,931,315,641 (565,209,762,172–1,140,903,691,414)	867,657,974,766 (646,354,522,562–1,326,145,072,015)
South Sudan	Group I	514,611,534,330 (451,760,083,060–607,662,917,582)	446,492,329,775 (390,624,981,175–540,385,487,255)	437,620,144,726 (371,533,315,977–558,290,056,905)	469,923,699,737 (388,699,708,996–629,585,351,827)	520,433,304,701 (420,707,560,611–729,905,077,795)	571,099,499,945 (443,254,137,483–813,947,851,476)
South Sudan	NCD	111,079,774,216 (71,826,189,750–178,669,137,378)	100,796,248,131 (63,696,365,557–165,405,202,825)	110,659,882,236 (69,657,127,419–191,461,441,554)	129,730,352,508 (81,512,697,349–230,433,537,009)	166,035,809,904 (104,891,379,930–315,451,620,331)	211,540,008,455 (134,057,198,144–391,353,467,929)
South Sudan	Injuries	42,892,776,835 (25,838,435,051–67,486,653,960)	34,831,108,068 (18,439,439,474–58,725,350,318)	34,280,179,627 (19,578,393,444–60,724,811,003)	37,957,425,065 (19,776,627,115–74,669,883,643)	49,462,201,036 (26,074,889,454–101,649,462,227)	85,018,466,367 (54,001,371,478–144,967,962,491)
Tanzania	All causes	2,283,564,865,456 (2,170,330,051,325–2,395,251,682,507)	2,608,254,084,795 (2,465,760,266,804–2,760,154,241,529)	2,768,942,184,865 (2,582,512,213,308–2,964,778,168,382)	2,746,291,369,121 (2,524,997,337,797–2,972,588,469,530)	2,601,800,216,440 (2,291,847,615,789–2,951,144,819,593)	2,487,334,286,836 (2,094,902,513,035–3,022,121,573,955)
Tanzania	Group I	1,722,876,320,358 (1,642,382,584,811–1,812,571,520,219)	1,992,958,917,264 (1,892,464,558,299–2,109,997,573,531)	2,117,774,770,471 (1,993,668,447,495–2,264,861,190,593)	2,009,185,254,260 (1,864,190,506,615–2,159,508,148,071)	1,734,569,991,746 (1,569,673,704,411–1,909,892,644,600)	1,469,417,918,268 (1,271,800,971,030–1,691,267,065,097)
Tanzania	NCD	434,107,472,475 (371,209,738,632–500,384,688,735)	487,849,700,416 (416,611,549,282–563,377,618,605)	527,120,593,042 (446,841,683,896–614,228,867,829)	598,417,676,981 (500,518,635,532–709,059,766,254)	706,070,133,108 (564,373,626,309–885,044,397,365)	836,478,035,354 (643,691,989,652–1,105,373,542,539)
Tanzania	Injuries	126,581,072,623 (97,639,121,964–160,510,040,936)	127,445,467,115 (100,921,557,662–156,608,817,320)	124,046,821,351 (99,000,836,743–148,675,726,141)	138,688,437,881 (112,643,309,058–169,642,399,537)	161,160,091,586 (124,611,312,637–216,455,490,554)	181,438,333,214 (134,549,822,753–255,698,051,116)
Uganda	All causes	1,874,448,376,415 (1,772,328,968,814–1,986,911,680,077)	2,203,326,649,428 (2,066,706,658,539–2,345,694,962,015)	2,336,613,510,450 (2,204,591,774,458–2,464,467,222,020)	2,265,477,722,433 (2,130,708,063,492–2,411,035,209,255)	2,171,503,357,132 (1,976,319,400,731–2,371,486,102,930)	2,057,556,494,602 (1,769,909,899,082–2,395,903,745,784)
Uganda	Group I	1,458,839,420,446 (1,379,690,197,229–1,550,651,998,514)	1,721,406,682,954 (1,615,201,036,648–1,830,599,038,984)	1,778,269,937,029 (1,683,337,440,670–1,870,367,050,312)	1,639,916,308,211 (1,548,131,822,394–1,739,479,661,187)	1,480,737,250,683 (1,365,535,198,287–1,598,063,145,601)	1,242,546,635,116 (1,099,247,604,350–1,396,719,611,261)
Uganda	NCD	318,090,616,776 (264,392,370,235–371,978,858,306)	371,688,492,382 (305,675,606,218–445,523,179,086)	426,361,514,438 (355,798,066,798–500,097,258,041)	483,396,922,577 (410,670,700,822–562,779,145,552)	537,780,102,709 (438,718,974,770–641,110,781,228)	643,564,001,268 (494,988,957,021–813,673,594,178)
Uganda	Injuries	97,518,339,192 (75,070,129,834–126,016,490,396)	110,231,474,092 (85,948,828,410–139,549,008,220)	131,982,058,983 (106,925,006,235–161,556,347,519)	142,164,491,646 (115,766,708,410–174,122,129,408)	152,986,003,740 (118,115,785,805–191,627,427,560)	171,445,858,218 (122,792,989,342–233,835,635,615)
Zambia	All causes	824,062,579,847 (776,273,970,445–873,029,478,882)	1,012,059,371,842 (947,671,614,629–1,084,634,782,079)	1,175,389,197,405 (1,088,921,902,837–1,265,338,614,726)	1,148,781,647,173 (1,061,285,898,219–1,242,658,463,804)	1,020,060,245,120 (922,097,267,473–1,117,268,213,233)	975,817,354,301 (835,922,088,983–1,140,332,568,921)
Zambia	Group I	633,132,885,757 (591,706,116,549–676,158,048,672)	784,922,566,030 (727,677,036,667–846,753,738,437)	916,023,988,219 (842,551,754,858–990,513,345,540)	852,482,113,202 (784,835,250,165–926,529,186,713)	690,840,946,351 (630,595,537,910–752,922,548,639)	612,845,741,547 (538,118,211,441–694,583,633,685)
Zambia	NCD	145,495,227,665 (122,435,932,231–169,298,028,819)	176,020,521,421 (148,812,741,537–204,183,724,311)	202,890,047,990 (175,203,091,305–234,210,494,104)	232,858,688,078 (201,396,250,060–266,107,748,699)	260,205,215,608 (222,076,831,393–303,525,230,780)	290,437,528,075 (228,821,994,264–367,076,381,104)
Zambia	Injuries	45,434,466,425 (34,193,677,991–57,961,813,761)	51,116,284,390 (38,362,846,039–63,818,418,970)	56,475,161,196 (44,311,933,610–70,565,592,557)	63,440,845,893 (52,508,575,850–76,644,615,170)	69,014,083,161 (56,496,424,126–84,305,957,273)	72,534,084,679 (51,400,219,334–98,893,771,380)
Central Sub-Saharan Africa	All causes	5,538,365,751,693 (5,099,804,610,860–6,024,309,325,776)	6,375,358,329,044 (5,894,552,469,308–6,986,936,589,973)	7,030,873,838,648 (6,535,526,714,546–7,679,355,184,634)	7,099,208,889,020 (6,528,477,436,962–7,810,080,283,914)	7,143,505,421,065 (6,451,554,690,000–8,056,398,465,172)	7,126,341,485,692 (6,194,128,559,123–8,297,691,253,288)
Central Sub-Saharan Africa	Group I	4,058,162,110,950 (3,688,273,528,624–4,498,181,592,066)	4,745,659,508,895 (4,319,458,142,985–5,224,282,623,964)	5,196,573,229,400 (4,781,383,908,300–5,749,200,614,388)	5,154,174,691,775 (4,723,274,758,175–5,723,002,596,480)	4,900,860,210,609 (4,411,219,031,618–5,561,268,102,600)	4,521,754,233,231 (3,888,126,657,699–5,335,646,697,234)
Central Sub-Saharan Africa	NCD	1,060,702,928,860 (856,375,686,691–1,327,484,402,477)	1,197,873,751,658 (965,233,770,861–1,466,047,852,758)	1,339,314,261,680 (1,095,079,795,184–1,653,139,970,016)	1,472,509,551,022 (1,214,335,675,197–1,810,023,269,788)	1,715,758,314,120 (1,419,734,693,533–2,099,771,699,780)	2,020,229,726,114 (1,651,897,324,718–2,494,631,167,574)
Central Sub-Saharan Africa	Injuries	419,500,711,882 (257,648,325,676–566,795,612,803)	431,825,068,491 (264,099,889,136–580,355,939,899)	494,986,347,569 (320,914,865,100–636,844,107,027)	472,524,646,223 (315,677,501,905–604,221,918,244)	526,886,896,336 (371,150,519,247–687,409,568,928)	584,357,526,347 (421,544,907,879–762,807,208,755)
Angola	All causes	1,389,486,576,249 (1,154,202,629,462–1,732,032,467,217)	1,432,259,677,496 (1,172,226,402,640–1,877,495,645,218)	1,553,220,837,672 (1,284,645,988,584–1,985,212,043,053)	1,518,525,368,587 (1,230,472,357,439–2,055,537,379,444)	1,528,695,780,211 (1,197,954,474,801–2,165,689,378,265)	1,508,996,787,655 (1,123,913,181,698–2,352,992,222,056)
Angola	Group I	966,302,224,303 (799,904,643,317–1,165,141,383,335)	1,001,764,511,918 (830,258,982,722–1,247,864,461,704)	1,084,495,819,773 (905,673,442,630–1,316,682,407,017)	1,029,206,594,341 (858,490,697,014–1,292,430,890,237)	942,357,307,843 (759,754,608,368–1,191,119,372,439)	867,974,049,185 (670,827,902,920–1,179,643,458,838)
Angola	NCD	268,600,139,460 (158,849,753,671–403,877,850,416)	291,140,642,265 (178,406,000,148–463,399,184,781)	318,247,046,471 (198,922,950,625–496,441,514,766)	351,226,024,705 (228,465,625,178–574,758,936,084)	421,625,850,112 (281,845,940,829–709,237,538,056)	475,822,008,410 (312,453,804,291–867,615,221,312)

Appendix Table 3. DALYs for all causes and level 1 cause groups by location for 1990 - 2015, both sexes combined.

	Cause	1990	1995	2000	2005	2010	2015
Angola	Injuries	154,584,212,486 (76,650,670,385–237,435,219,352)	139,354,523,313 (64,890,872,253–226,525,481,230)	150,477,971,427 (74,323,036,663–233,027,222,982)	138,092,749,541 (73,978,916,328–231,497,648,107)	164,712,622,257 (93,539,295,427–285,878,992,881)	165,200,730,060 (96,135,450,251–315,866,338,172)
Central African Republic	All causes	325,412,617,372 (302,134,966,748–349,510,537,813)	372,968,810,645 (329,164,320,246–418,196,835,478)	427,697,967,860 (361,436,381,881–502,643,898,855)	446,802,671,870 (366,669,819,615–541,133,069,722)	438,109,669,836 (350,721,875,572–540,155,640,370)	439,690,732,799 (339,844,812,728–560,286,361,141)
Central African Republic	Group I	227,944,555,531 (207,317,263,468–249,847,455,557)	266,184,687,971 (235,154,434,600–298,989,978,703)	309,811,582,983 (267,166,583,394–357,255,032,874)	321,256,699,170 (276,304,963,952–372,060,636,746)	296,640,105,154 (248,351,687,700–353,993,259,604)	281,315,908,691 (224,189,407,487–348,781,955,268)
Central African Republic	NCD	72,114,390,795 (60,002,054,280–86,990,642,338)	80,309,483,747 (61,133,919,674–99,707,684,828)	88,835,233,256 (62,295,733,397–116,484,999,593)	95,270,136,232 (65,725,668,887–129,944,802,955)	106,443,016,305 (72,325,755,170–144,924,774,895)	118,372,399,077 (79,679,211,783–164,713,451,341)
Central African Republic	Injuries	25,353,671,046 (16,503,087,167–33,958,866,163)	26,474,638,927 (16,335,853,502–37,058,269,545)	29,051,151,621 (15,879,395,496–44,157,775,891)	30,275,836,468 (16,475,937,720–47,344,339,863)	35,026,548,378 (19,723,599,088–54,203,333,520)	40,002,425,030 (23,245,493,348–63,412,492,595)
Congo	All causes	174,111,993,314 (156,535,698,902–194,501,716,715)	213,619,586,703 (194,999,597,598–232,192,059,684)	254,417,414,297 (233,400,563,055–276,181,147,354)	241,109,204,766 (219,974,324,277–264,784,602,158)	227,967,776,833 (201,218,397,190–260,959,305,359)	235,288,055,086 (190,784,394,420–289,081,546,697)
Congo	Group I	105,514,103,583 (95,008,557,567–117,745,196,221)	134,477,479,276 (122,434,873,131–148,520,531,282)	164,448,453,228 (150,067,483,891–179,533,975,860)	156,176,314,243 (141,496,669,112–172,090,791,993)	132,858,880,157 (117,642,038,225–151,371,313,207)	130,292,173,526 (107,368,594,863–157,125,753,600)
Congo	NCD	53,789,560,084 (44,657,463,495–63,975,372,323)	62,352,522,393 (52,882,844,827–72,198,021,757)	67,941,242,456 (57,017,441,889–79,986,833,138)	67,712,275,469 (56,496,224,443–79,459,624,522)	76,859,909,113 (63,132,589,225–92,489,677,838)	86,516,907,859 (65,066,470,812–113,411,123,759)
Congo	Injuries	14,808,329,646 (10,181,105,434–19,176,960,768)	16,789,585,033 (11,475,518,281–21,475,751,971)	22,027,718,612 (15,202,930,799–28,177,192,799)	17,220,615,054 (12,357,665,924–22,096,824,891)	18,248,987,563 (13,540,814,054–23,629,322,973)	18,478,973,701 (12,956,546,044–26,154,262,689)
Democratic Republic of the Congo	All causes	3,544,914,536,202 (3,221,774,520,348–3,884,652,091,996)	4,239,958,660,285 (3,898,200,017,324–4,601,879,860,607)	4,673,443,135,227 (4,319,144,356,455–5,085,659,942,289)	4,765,696,909,892 (4,391,619,378,461–5,202,673,077,969)	4,821,460,028,508 (4,370,158,464,351–5,390,134,181,412)	4,819,290,590,691 (4,102,712,881,663–5,721,778,281,673)
Democratic Republic of the Congo	Group I	2,696,824,194,013 (2,433,876,547,203–3,008,164,068,805)	3,273,331,279,746 (2,971,352,198,022–3,627,491,360,862)	3,564,189,209,238 (3,247,875,363,522–4,012,045,727,367)	3,572,919,540,413 (3,248,344,719,021–4,019,996,882,905)	3,457,656,742,091 (3,095,986,910,709–3,975,721,588,108)	3,179,277,441,205 (2,649,570,203,061–3,878,228,540,418)
Democratic Republic of the Congo	NCD	633,050,452,465 (494,708,963,759–802,670,982,798)	727,691,215,622 (589,742,090,701–890,929,316,949)	825,818,994,134 (680,160,572,480–1,020,166,652,675)	916,843,144,759 (765,669,582,276–1,115,309,780,455)	1,066,324,164,479 (887,846,363,701–1,288,631,896,544)	1,290,747,405,313 (1,042,272,949,597–1,596,417,698,800)
Democratic Republic of the Congo	Injuries	215,039,889,724 (125,302,189,954–301,514,563,212)	238,936,164,917 (149,495,727,379–329,523,583,966)	283,434,931,855 (189,774,714,665–373,581,100,593)	275,934,224,720 (190,977,076,681–358,788,570,599)	297,479,121,938 (211,566,939,874–391,685,040,767)	349,265,744,173 (243,381,297,129–469,486,336,646)
Equatorial Guinea	All causes	42,968,969,632 (32,934,761,056–57,747,390,538)	50,635,399,720 (39,127,389,508–68,411,833,120)	48,770,908,156 (37,966,240,697–66,884,067,354)	49,003,128,443 (38,018,743,418–70,799,061,756)	49,807,177,361 (37,996,742,078–73,886,483,273)	49,156,147,493 (36,360,456,485–79,315,931,550)
Equatorial Guinea	Group I	28,538,239,784 (23,307,701,988–35,213,918,742)	34,678,490,830 (28,530,397,150–43,238,427,312)	32,392,614,489 (26,408,184,980–41,064,206,431)	31,467,333,605 (24,861,802,704–41,054,613,604)	29,749,885,105 (22,985,846,463–39,463,542,129)	27,790,780,105 (20,890,306,112–38,951,820,984)
Equatorial Guinea	NCD	10,617,130,713 (6,278,780,663–16,653,521,974)	11,803,609,603 (6,878,078,714–18,968,444,224)	12,216,593,869 (7,504,092,649–20,597,886,869)	12,852,955,761 (8,536,891,583–22,533,898,046)	15,082,438,340 (10,304,238,781–26,557,585,031)	16,719,961,984 (11,441,918,479–30,944,021,180)
Equatorial Guinea	Injuries	3,813,599,135 (1,848,439,117–6,702,494,903)	4,153,299,287 (1,973,807,020–7,302,555,688)	4,161,699,797 (1,851,707,078–7,590,157,292)	4,682,839,078 (2,508,105,985–8,479,211,833)	4,974,853,916 (2,529,267,974–9,424,932,795)	4,645,405,404 (2,554,417,875–9,305,579,975)
Gabon	All causes	61,471,058,923 (56,341,211,089–66,816,954,161)	65,916,194,195 (60,284,899,069–72,601,801,694)	73,323,575,437 (66,931,732,692–80,755,900,067)	78,071,605,462 (70,142,905,231–86,909,433,016)	77,464,988,316 (67,949,579,757–87,941,183,703)	73,919,171,969 (61,213,378,485–91,715,358,480)
Gabon	Group I	33,038,793,737 (30,244,335,862–36,056,847,755)	35,223,059,154 (32,241,086,148–38,809,052,937)	41,235,549,687 (37,228,688,618–45,341,825,047)	43,148,210,004 (38,865,311,275–48,507,141,581)	41,597,290,260 (36,612,894,815–47,768,204,715)	35,103,880,519 (29,583,796,326–43,703,991,816)
Gabon	NCD	22,531,255,342 (19,265,250,939–25,903,011,603)	24,576,278,027 (21,078,865,151–28,359,203,143)	26,255,151,493 (22,470,207,185–30,754,979,607)	28,605,014,097 (24,365,190,902–33,675,615,263)	29,422,935,771 (24,272,796,080–35,207,180,906)	32,051,043,471 (25,270,857,971–41,752,484,652)
Gabon	Injuries	5,901,009,844 (4,365,754,292–7,397,203,208)	6,116,857,014 (4,622,571,407–7,592,824,872)	5,832,874,257 (4,348,552,562–7,349,677,870)	6,318,381,361 (4,663,092,373–8,180,344,324)	6,444,762,285 (4,777,536,759–8,454,151,699)	6,764,247,979 (4,764,579,666–9,947,251,627)

Appendix Table 4. Rate of DALYs per 100,000 individuals for all causes and level 1 cause groups by location for 1990 - 2015, both sexes combined.

	Cause	1990	1995	2000	2005	2010	2015
Global	All causes	48,958.47 (45,948.54–52,395.93)	47,212.22 (44,247.86–50,575.37)	44,856.96 (41,966.63–48,185.67)	41,561.74 (38,640.76–44,850.35)	37,612.12 (34,692.75–40,764.31)	34,445.68 (31,602.96–37,654.29)
Global	Group I	18,397.88 (17,715.28–19,226.38)	17,219.71 (16,601.34–17,948.21)	16,082.74 (15,484.14–16,783.65)	14,297.57 (13,765.97–14,955.05)	11,987.74 (11,466.12–12,650.55)	10,007.15 (9,498.53–10,629.30)
Global	NCD	25,571.25 (23,182.26–28,087.07)	25,212.88 (22,831.02–27,714.72)	24,352.48 (22,058.76–26,822.08)	23,220.17 (20,898.51–25,688.98)	21,882.64 (19,551.17–24,368.09)	21,062.39 (18,790.57–23,517.95)
Global	Injuries	4,989.34 (4,625.08–5,315.26)	4,779.63 (4,412.45–5,087.28)	4,421.74 (4,122.20–4,701.10)	4,044.00 (3,764.84–4,300.14)	3,741.74 (3,445.21–4,019.38)	3,376.14 (3,120.78–3,607.03)
High SDI	All causes	27,957.58 (25,183.44–31,083.15)	28,101.42 (25,348.34–31,237.54)	26,324.02 (23,599.59–29,476.90)	24,959.87 (22,270.01–28,063.31)	22,730.38 (20,044.00–25,789.82)	21,705.38 (19,004.98–24,704.80)
High SDI	Group I	2,635.56 (2,386.19–2,951.33)	2,503.06 (2,256.12–2,801.00)	2,224.42 (1,978.21–2,526.64)	2,053.52 (1,807.18–2,362.38)	1,844.89 (1,605.10–2,162.67)	1,704.93 (1,468.45–2,017.59)
High SDI	NCD	21,651.59 (19,414.49–24,206.97)	21,684.03 (19,441.01–24,204.06)	20,571.65 (18,321.91–23,089.78)	19,620.95 (17,381.03–22,141.26)	18,221.77 (15,963.30–20,745.10)	17,596.63 (15,309.61–20,123.63)
High SDI	Injuries	3,670.44 (3,425.99–3,957.98)	3,914.34 (3,664.62–4,205.28)	3,527.95 (3,305.29–3,790.33)	3,285.40 (3,067.40–3,544.03)	2,663.73 (2,474.13–2,905.55)	2,403.82 (2,214.41–2,637.65)
High-middle SDI	All causes	39,622.37 (36,694.42–42,916.65)	37,830.41 (34,885.30–40,977.48)	35,501.18 (32,594.34–38,689.56)	33,099.32 (30,241.31–36,301.92)	29,841.62 (27,067.38–32,958.69)	27,124.81 (24,387.39–30,263.03)
High-middle SDI	Group I	8,540.64 (8,077.22–9,113.87)	7,468.67 (7,027.32–7,961.44)	6,771.30 (6,364.06–7,242.28)	6,190.89 (5,823.09–6,661.13)	5,277.70 (4,884.38–5,750.04)	4,186.79 (3,818.07–4,628.76)
High-middle SDI	NCD	26,071.11 (23,646.93–28,710.26)	25,662.92 (23,288.81–28,228.72)	24,559.99 (22,220.90–27,186.97)	23,193.41 (20,857.57–25,769.17)	21,332.06 (18,971.11–23,884.84)	20,083.63 (17,790.25–22,695.09)
High-middle SDI	Injuries	5,010.62 (4,689.06–5,332.62)	4,698.81 (4,435.10–4,948.43)	4,169.89 (3,962.53–4,390.44)	3,715.02 (3,518.74–3,925.23)	3,231.86 (3,048.50–3,428.78)	2,854.39 (2,659.29–3,059.78)
Middle SDI	All causes	46,827.82 (43,979.65–50,256.44)	43,258.81 (40,456.16–46,406.49)	40,272.80 (37,567.76–43,341.26)	36,957.85 (34,144.31–40,064.04)	33,511.56 (30,806.37–36,575.05)	30,874.37 (28,200.51–33,866.72)
Middle SDI	Group I	14,974.99 (14,284.44–15,834.48)	12,818.22 (12,193.23–13,546.91)	11,107.16 (10,528.41–11,773.94)	9,257.15 (8,758.60–9,836.22)	7,631.97 (7,188.85–8,158.90)	6,233.12 (5,797.57–6,737.71)
Middle SDI	NCD	26,908.22 (24,627.55–29,400.29)	25,870.16 (23,611.13–28,261.28)	25,008.27 (22,870.20–27,420.77)	23,901.18 (21,683.84–26,278.63)	22,467.87 (20,248.25–24,888.65)	21,338.09 (19,148.43–23,708.30)
Middle SDI	Injuries	4,944.61 (4,453.44–5,256.03)	4,570.43 (4,103.18–4,856.13)	4,157.37 (3,843.50–4,385.84)	3,799.52 (3,488.14–4,008.88)	3,411.73 (3,106.11–3,604.51)	3,303.16 (2,994.54–3,554.12)
Low-middle SDI	All causes	69,139.20 (65,350.73–73,307.87)	65,402.84 (61,777.34–69,538.70)	61,945.99 (58,272.50–65,854.76)	57,484.98 (53,863.94–61,324.50)	52,412.99 (48,867.65–56,230.56)	47,133.14 (43,570.42–50,921.64)
Low-middle SDI	Group I	34,925.53 (33,529.17–36,358.85)	31,530.48 (30,315.23–32,821.49)	28,737.16 (27,622.99–29,939.67)	25,574.35 (24,590.89–26,759.02)	21,044.46 (20,128.66–22,108.22)	17,326.54 (16,416.80–18,305.07)
Low-middle SDI	NCD	28,787.47 (26,151.35–31,500.56)	28,634.93 (26,008.74–31,342.03)	28,290.46 (25,538.02–31,005.19)	27,137.78 (24,468.85–29,793.10)	26,400.95 (23,752.53–29,111.84)	25,768.22 (23,188.43–28,376.76)
Low-middle SDI	Injuries	5,426.20 (4,883.59–5,924.46)	5,237.43 (4,632.35–5,697.73)	4,918.36 (4,358.59–5,326.55)	4,772.86 (4,267.50–5,149.77)	4,967.58 (4,211.72–5,736.49)	4,038.38 (3,602.49–4,361.62)
Low SDI	All causes	89,500.11 (84,568.99–94,716.50)	87,413.32 (82,649.56–92,180.91)	84,276.53 (79,469.38–88,829.28)	74,510.31 (70,113.54–79,271.48)	65,000.52 (60,450.43–70,262.28)	59,078.97 (54,082.79–65,004.61)
Low SDI	Group I	52,324.48 (49,847.36–54,949.36)	52,147.68 (49,838.32–54,779.05)	49,245.41 (47,052.31–51,765.61)	42,311.49 (40,290.30–44,686.10)	33,903.09 (32,069.53–36,199.13)	27,634.39 (25,747.13–30,036.33)
Low SDI	NCD	29,374.54 (26,432.05–32,317.42)	28,795.98 (26,009.31–31,797.04)	28,099.88 (25,196.98–30,863.02)	26,881.48 (24,111.24–29,816.73)	26,075.30 (23,226.33–29,137.95)	26,185.24 (23,224.28–29,634.97)
Low SDI	Injuries	7,801.08 (6,709.55–8,812.98)	6,469.66 (5,719.49–7,113.01)	6,931.23 (6,070.53–7,782.92)	5,317.34 (4,789.67–5,806.18)	5,022.13 (4,509.75–5,526.27)	5,259.34 (4,655.06–5,947.30)
High-income	All causes	25,643.18 (22,934.38–28,723.60)	24,392.50 (21,702.83–27,482.82)	22,864.57 (20,206.24–25,945.11)	21,572.56 (18,921.61–24,626.73)	20,426.78 (17,794.13–23,459.95)	19,926.25 (17,286.66–22,927.83)
High-income	Group I	2,317.14 (2,095.78–2,600.93)	2,222.69 (2,007.41–2,500.61)	1,894.68 (1,677.66–2,175.75)	1,750.61 (1,532.68–2,038.08)	1,608.61 (1,389.20–1,899.49)	1,491.16 (1,274.63–1,778.40)
High-income	NCD	20,201.74 (17,975.69–22,739.06)	19,249.90 (17,014.55–21,786.41)	18,334.82 (16,098.90–20,872.27)	17,396.93 (15,153.30–19,927.30)	16,676.50 (14,425.65–19,204.89)	16,424.74 (14,163.87–18,961.97)

Appendix Table 4. Rate of DALYs per 100,000 individuals for all causes and level 1 cause groups by location for 1990 - 2015, both sexes combined.							
	**Cause**	**1990**	**1995**	**2000**	**2005**	**2010**	**2015**
High-income	Injuries	3,124.30 (2,880.23–3,415.63)	2,919.90 (2,683.12–3,206.55)	2,635.07 (2,416.81–2,903.05)	2,425.01 (2,216.35–2,682.19)	2,141.67 (1,953.86–2,385.47)	2,010.35 (1,829.97–2,248.58)
High-income North America	All causes	27,265.84 (24,496.72–30,434.94)	26,478.67 (23,718.97–29,619.04)	24,973.88 (22,194.31–28,119.09)	24,258.79 (21,492.51–27,406.91)	23,019.49 (20,198.69–26,155.24)	22,687.32 (19,818.20–25,843.09)
High-income North America	Group I	2,466.49 (2,258.80–2,716.38)	2,509.32 (2,305.42–2,772.38)	2,010.13 (1,795.15–2,291.44)	1,905.61 (1,683.29–2,195.31)	1,714.70 (1,492.57–2,006.67)	1,595.90 (1,375.99–1,885.19)
High-income North America	NCD	21,474.84 (19,083.61–24,205.29)	20,890.11 (18,491.11–23,581.68)	20,173.39 (17,770.38–22,889.25)	19,542.12 (17,119.62–22,260.32)	18,854.30 (16,406.88–21,604.91)	18,744.08 (16,253.09–21,475.23)
High-income North America	Injuries	3,324.51 (3,124.62–3,566.56)	3,079.24 (2,883.02–3,314.77)	2,790.36 (2,604.27–3,019.45)	2,811.05 (2,613.34–3,047.75)	2,450.49 (2,275.75–2,673.99)	2,347.34 (2,167.59–2,573.02)
Canada	All causes	23,881.69 (21,099.59–26,997.16)	23,026.31 (20,289.04–26,144.40)	21,624.66 (18,896.38–24,727.05)	20,544.51 (17,826.66–23,657.80)	19,478.20 (16,768.79–22,529.29)	19,119.17 (16,377.64–22,104.36)
Canada	Group I	1,872.45 (1,623.46–2,205.26)	1,895.58 (1,652.22–2,227.12)	1,627.91 (1,388.18–1,963.45)	1,557.14 (1,320.69–1,877.51)	1,536.19 (1,288.85–1,879.83)	1,462.12 (1,200.97–1,796.21)
Canada	NCD	19,277.49 (16,949.07–21,876.17)	18,671.88 (16,346.16–21,258.69)	17,814.83 (15,502.24–20,437.74)	16,961.00 (14,627.90–19,592.25)	16,125.15 (13,809.15–18,691.13)	15,946.01 (13,590.63–18,482.90)
Canada	Injuries	2,731.76 (2,535.64–2,975.11)	2,458.85 (2,270.23–2,689.77)	2,181.91 (2,005.83–2,396.77)	2,026.37 (1,852.96–2,234.30)	1,816.87 (1,655.24–2,018.38)	1,711.03 (1,551.78–1,909.47)
Greenland	All causes	48,245.76 (44,978.35–51,818.04)	43,669.58 (40,535.60–46,958.89)	40,896.13 (37,692.79–44,377.68)	38,141.69 (34,910.32–41,465.87)	34,702.55 (31,736.05–37,890.51)	32,339.98 (29,274.11–35,707.68)
Greenland	Group I	6,040.67 (5,435.75–6,641.11)	5,193.01 (4,630.11–5,760.19)	4,252.00 (3,761.53–4,749.60)	3,851.91 (3,395.59–4,308.28)	3,484.14 (3,056.90–3,944.66)	3,120.68 (2,695.40–3,537.24)
Greenland	NCD	31,952.52 (29,235.57–34,818.96)	29,601.30 (26,995.29–32,245.71)	28,504.67 (25,932.83–31,348.98)	26,871.85 (24,237.47–29,571.13)	24,703.70 (22,149.95–27,337.68)	23,386.09 (20,778.32–26,051.15)
Greenland	Injuries	10,252.57 (9,576.87–10,882.02)	8,875.26 (8,374.57–9,401.57)	8,139.47 (7,627.20–8,667.91)	7,417.94 (6,979.56–7,857.04)	6,514.71 (6,088.62–6,946.04)	5,833.20 (5,287.52–6,386.43)
United States	All causes	27,635.59 (24,868.46–30,802.34)	26,861.36 (24,097.76–30,002.67)	25,341.50 (22,543.46–28,484.06)	24,671.76 (21,896.62–27,819.61)	23,425.25 (20,584.07–26,566.63)	23,103.97 (20,223.79–26,282.15)
United States	Group I	2,527.96 (2,322.32–2,768.54)	2,574.90 (2,374.86–2,828.71)	2,047.72 (1,835.29–2,322.90)	1,940.37 (1,718.75–2,223.36)	1,733.48 (1,514.00–2,018.24)	1,611.03 (1,395.20–1,896.64)
United States	NCD	21,717.89 (19,314.60–24,451.85)	21,139.96 (18,735.74–23,842.78)	20,439.21 (18,029.40–23,162.71)	19,836.80 (17,396.41–22,566.91)	19,171.70 (16,700.97–21,937.60)	19,073.99 (16,561.73–21,827.43)
United States	Injuries	3,389.73 (3,189.84–3,629.31)	3,146.50 (2,949.09–3,381.88)	2,854.57 (2,666.93–3,084.95)	2,894.59 (2,692.34–3,138.56)	2,520.08 (2,344.09–2,745.11)	2,418.95 (2,237.12–2,648.47)
Australasia	All causes	25,054.95 (22,236.05–28,193.02)	23,270.97 (20,484.68–26,413.11)	21,820.42 (19,002.03–25,011.12)	20,277.31 (17,502.01–23,392.31)	19,328.86 (16,556.77–22,414.21)	18,875.86 (16,062.93–21,914.56)
Australasia	Group I	1,666.44 (1,457.45–1,954.05)	1,510.45 (1,300.94–1,786.53)	1,414.90 (1,200.22–1,688.14)	1,355.78 (1,141.45–1,634.61)	1,273.30 (1,058.91–1,561.68)	1,167.23 (946.41–1,461.72)
Australasia	NCD	20,646.06 (18,218.44–23,419.87)	19,379.00 (16,949.12–22,171.65)	18,210.65 (15,790.42–21,013.54)	17,028.28 (14,615.52–19,785.80)	16,370.77 (13,947.24–19,088.52)	16,131.90 (13,704.33–18,825.55)
Australasia	Injuries	2,742.45 (2,551.67–2,962.50)	2,381.51 (2,202.21–2,592.90)	2,194.87 (2,027.84–2,395.19)	1,893.26 (1,733.71–2,087.15)	1,684.79 (1,539.78–1,871.20)	1,576.74 (1,433.45–1,759.22)
Australia	All causes	24,760.74 (21,935.71–27,910.26)	23,016.27 (20,224.48–26,173.98)	21,611.64 (18,797.54–24,822.86)	20,069.63 (17,299.95–23,199.61)	19,148.17 (16,382.02–22,229.71)	18,757.79 (15,970.63–21,786.45)
Australia	Group I	1,660.79 (1,439.49–1,949.76)	1,516.51 (1,299.69–1,802.81)	1,434.44 (1,209.03–1,711.69)	1,379.29 (1,156.75–1,667.85)	1,286.20 (1,065.41–1,585.90)	1,176.68 (949.70–1,470.10)
Australia	NCD	20,453.63 (18,007.86–23,238.99)	19,201.13 (16,772.30–21,991.71)	18,033.40 (15,615.55–20,840.18)	16,858.98 (14,450.05–19,622.63)	16,236.24 (13,814.22–18,962.20)	16,045.03 (13,623.95–18,737.56)
Australia	Injuries	2,646.31 (2,457.20–2,865.62)	2,298.63 (2,121.35–2,510.77)	2,143.80 (1,971.96–2,345.86)	1,831.36 (1,671.29–2,022.77)	1,625.73 (1,482.58–1,808.66)	1,536.07 (1,394.44–1,717.98)
New Zealand	All causes	26,525.46 (23,804.12–29,623.48)	24,527.52 (21,785.31–27,656.95)	22,878.58 (20,126.42–25,970.46)	21,326.73 (18,532.20–24,405.28)	20,248.64 (17,507.88–23,315.47)	19,473.55 (16,655.11–22,513.29)
New Zealand	Group I	1,698.85 (1,504.59–1,943.39)	1,481.60 (1,288.04–1,727.87)	1,318.81 (1,118.88–1,577.31)	1,241.74 (1,038.56–1,510.56)	1,208.83 (1,004.74–1,469.46)	1,119.56 (916.21–1,378.72)

Appendix Table 4. Rate of DALYs per 100,000 individuals for all causes and level 1 cause groups by location for 1990 - 2015, both sexes combined.

	Cause	1990	1995	2000	2005	2010	2015
New Zealand	NCD	21,612.51 (19,208.49–24,318.89)	20,265.81 (17,826.35–23,029.32)	19,117.77 (16,693.05–21,906.54)	17,899.55 (15,461.69–20,593.35)	17,058.67 (14,639.01–19,737.58)	16,565.55 (14,046.82–19,269.67)
New Zealand	Injuries	3,214.10 (3,010.54–3,445.01)	2,780.10 (2,594.57–3,000.60)	2,442.01 (2,270.37–2,649.07)	2,185.44 (2,024.34–2,384.15)	1,981.14 (1,827.60–2,172.47)	1,788.43 (1,631.05–1,976.70)
High-income Asia Pacific	All causes	22,303.30 (19,924.56–25,032.79)	21,117.69 (18,772.83–23,858.08)	19,366.55 (17,023.98–22,053.15)	17,978.70 (15,662.51–20,601.21)	16,970.22 (14,680.43–19,557.91)	16,493.98 (14,226.18–19,038.78)
High-income Asia Pacific	Group I	2,076.23 (1,819.86–2,383.31)	1,873.10 (1,638.69–2,173.23)	1,710.58 (1,477.34–2,002.61)	1,513.69 (1,285.71–1,813.16)	1,408.02 (1,180.52–1,720.16)	1,336.06 (1,110.58–1,643.68)
High-income Asia Pacific	NCD	17,373.91 (15,408.34–19,618.54)	16,241.43 (14,279.68–18,453.68)	15,095.26 (13,144.75–17,293.49)	14,169.66 (12,230.09–16,334.03)	13,471.58 (11,541.71–15,604.46)	13,229.78 (11,306.99–15,378.18)
High-income Asia Pacific	Injuries	2,853.16 (2,639.36–3,096.09)	3,003.16 (2,670.92–3,394.50)	2,560.70 (2,370.70–2,791.69)	2,295.35 (2,121.77–2,516.45)	2,090.62 (1,934.99–2,296.27)	1,928.14 (1,774.51–2,126.80)
Brunei	All causes	27,903.53 (25,344.58–30,890.70)	26,659.04 (24,142.29–29,577.13)	24,762.55 (22,199.87–27,595.87)	23,425.78 (20,898.27–26,245.60)	22,884.66 (20,383.50–25,703.48)	22,738.97 (20,142.98–25,575.74)
Brunei	Group I	2,510.35 (2,225.38–2,879.37)	2,321.25 (2,045.84–2,668.02)	2,169.68 (1,906.53–2,501.89)	2,100.78 (1,852.51–2,437.21)	2,046.74 (1,797.26–2,391.17)	2,054.05 (1,784.60–2,399.16)
Brunei	NCD	21,905.90 (19,807.40–24,340.04)	21,165.83 (19,082.52–23,545.60)	19,870.10 (17,782.24–22,219.90)	18,923.76 (16,773.57–21,241.27)	18,573.54 (16,484.04–20,848.80)	18,496.87 (16,351.54–20,850.85)
Brunei	Injuries	3,487.28 (3,111.92–3,850.71)	3,171.96 (2,883.76–3,510.24)	2,722.77 (2,462.39–3,005.28)	2,401.25 (2,145.01–2,691.90)	2,264.38 (2,019.06–2,545.84)	2,188.04 (1,929.18–2,493.21)
Japan	All causes	19,964.98 (17,616.43–22,605.75)	19,434.73 (17,099.60–22,153.89)	18,111.62 (15,785.97–20,712.48)	17,202.01 (14,929.50–19,769.87)	16,416.20 (14,142.53–18,936.98)	16,011.68 (13,740.44–18,526.17)
Japan	Group I	1,734.92 (1,523.63–2,003.96)	1,701.04 (1,486.39–1,973.20)	1,562.76 (1,344.29–1,841.52)	1,416.27 (1,201.87–1,702.51)	1,329.10 (1,116.79–1,620.14)	1,277.90 (1,067.63–1,568.87)
Japan	NCD	15,999.66 (14,017.96–18,212.15)	15,234.63 (13,288.15–17,421.88)	14,364.27 (12,420.55–16,540.42)	13,721.37 (11,786.99–15,879.06)	13,192.14 (11,264.56–15,333.21)	12,970.73 (11,040.05–15,111.70)
Japan	Injuries	2,230.40 (2,057.84–2,435.38)	2,499.06 (2,127.27–3,017.75)	2,184.59 (2,012.77–2,397.10)	2,064.37 (1,906.97–2,263.89)	1,894.96 (1,751.27–2,080.79)	1,763.05 (1,622.36–1,950.43)
Singapore	All causes	24,659.14 (22,367.34–27,219.73)	22,314.16 (20,038.58–24,899.26)	19,720.43 (17,531.43–22,281.77)	17,568.43 (15,413.95–20,036.45)	16,197.92 (14,066.91–18,566.60)	15,586.12 (13,463.44–18,040.14)
Singapore	Group I	2,865.31 (2,601.15–3,187.77)	2,491.94 (2,245.74–2,821.06)	2,239.94 (1,994.68–2,559.67)	1,985.46 (1,757.23–2,308.41)	1,871.44 (1,645.62–2,179.21)	1,814.70 (1,578.08–2,120.91)
Singapore	NCD	19,529.60 (17,667.65–21,630.13)	17,897.31 (16,023.51–19,994.20)	15,831.75 (14,014.21–17,921.32)	14,156.29 (12,355.97–16,195.67)	13,147.28 (11,296.73–15,174.51)	12,682.24 (10,809.03–14,745.91)
Singapore	Injuries	2,264.23 (2,049.17–2,523.05)	1,924.92 (1,714.99–2,176.63)	1,648.74 (1,465.18–1,867.41)	1,426.68 (1,262.13–1,632.07)	1,179.20 (1,032.53–1,370.49)	1,089.18 (946.26–1,281.35)
South Korea	All causes	31,341.09 (28,689.19–34,209.57)	27,546.27 (25,075.08–30,390.37)	24,022.68 (21,504.20–26,889.08)	20,720.09 (18,290.59–23,532.15)	18,779.99 (16,366.13–21,503.27)	18,035.86 (15,673.09–20,742.95)
South Korea	Group I	2,859.01 (2,481.98–3,344.16)	2,150.67 (1,847.64–2,532.95)	1,938.11 (1,681.45–2,267.25)	1,638.04 (1,385.30–1,970.76)	1,498.83 (1,242.99–1,846.89)	1,384.19 (1,128.96–1,727.71)
South Korea	NCD	23,851.04 (21,796.45–26,222.48)	20,962.78 (18,913.13–23,244.54)	18,466.19 (16,434.51–20,765.27)	16,072.81 (14,058.28–18,268.92)	14,595.25 (12,594.30–16,834.89)	14,220.54 (12,258.00–16,421.79)
South Korea	Injuries	4,631.05 (4,289.40–5,021.56)	4,432.82 (4,109.06–4,809.94)	3,618.38 (3,356.55–3,956.53)	3,009.24 (2,770.51–3,290.21)	2,685.92 (2,495.63–2,927.69)	2,431.14 (2,226.89–2,679.88)
Western Europe	All causes	25,113.38 (22,348.49–28,276.36)	23,649.80 (20,879.59–26,794.87)	22,205.02 (19,463.56–25,347.70)	20,526.53 (17,804.11–23,655.48)	19,296.29 (16,630.72–22,379.01)	18,658.36 (16,022.00–21,724.06)
Western Europe	Group I	1,902.97 (1,689.97–2,176.90)	1,817.01 (1,609.04–2,093.75)	1,553.50 (1,343.51–1,829.20)	1,415.70 (1,208.10–1,695.08)	1,317.98 (1,114.31–1,598.22)	1,216.69 (1,008.10–1,501.41)
Western Europe	NCD	20,182.45 (17,916.64–22,730.33)	19,141.80 (16,850.35–21,695.66)	18,207.98 (15,938.67–20,748.97)	17,028.60 (14,758.73–19,569.56)	16,192.73 (13,937.19–18,729.06)	15,800.70 (13,501.69–18,310.31)
Western Europe	Injuries	3,027.95 (2,734.71–3,381.84)	2,690.99 (2,410.52–3,025.66)	2,443.53 (2,181.28–2,759.22)	2,082.24 (1,841.57–2,383.84)	1,785.59 (1,565.68–2,068.46)	1,640.96 (1,438.30–1,907.56)
Andorra	All causes	18,874.05 (16,126.30–22,039.54)	17,926.76 (15,218.99–20,946.52)	16,992.29 (14,305.17–19,998.76)	16,425.69 (13,734.88–19,475.41)	15,944.57 (13,329.97–18,996.00)	15,909.17 (13,238.86–18,983.72)

Appendix Table 4. Rate of DALYs per 100,000 individuals for all causes and level 1 cause groups by location for 1990 - 2015, both sexes combined.

	Cause	1990	1995	2000	2005	2010	2015
Andorra	Group I	1,539.34 (1,134.45–3,143.06)	1,483.05 (1,005.85–3,437.24)	1,227.63 (880.99–2,412.06)	1,145.78 (838.15–1,899.82)	1,064.86 (778.26–1,668.58)	1,021.34 (750.95–1,543.61)
Andorra	NCD	15,210.96 (12,851.65–17,837.24)	14,551.21 (12,180.14–17,167.05)	14,012.38 (11,727.77–16,576.08)	13,656.23 (11,372.61–16,145.55)	13,419.07 (11,171.60–15,975.03)	13,480.48 (11,201.47–16,090.86)
Andorra	Injuries	2,123.75 (1,683.51–2,512.03)	1,892.49 (1,420.40–2,269.92)	1,752.27 (1,401.83–2,101.33)	1,623.69 (1,341.45–1,955.32)	1,460.64 (1,191.70–1,777.20)	1,407.35 (1,156.60–1,717.42)
Austria	All causes	25,940.12 (23,159.52–29,113.47)	24,336.23 (21,597.05–27,502.05)	22,762.17 (20,036.91–25,936.14)	20,993.72 (18,234.71–24,143.99)	19,850.64 (17,126.73–22,941.32)	18,960.64 (16,235.07–22,035.63)
Austria	Group I	1,670.35 (1,455.17–1,956.20)	1,444.11 (1,227.86–1,737.67)	1,268.24 (1,053.01–1,532.88)	1,227.60 (1,020.42–1,483.20)	1,146.82 (933.68–1,419.56)	1,025.23 (814.92–1,300.77)
Austria	NCD	20,719.11 (18,412.07–23,297.19)	19,788.96 (17,459.24–22,394.84)	18,749.20 (16,439.07–21,319.62)	17,398.66 (15,089.64–19,918.42)	16,651.93 (14,347.74–19,247.25)	16,108.95 (13,792.78–18,651.14)
Austria	Injuries	3,550.66 (3,214.00–3,926.32)	3,103.17 (2,800.66–3,457.85)	2,744.73 (2,456.77–3,084.57)	2,367.47 (2,101.89–2,701.21)	2,051.90 (1,803.39–2,358.41)	1,826.45 (1,601.10–2,116.53)
Belgium	All causes	25,499.75 (22,742.84–28,637.22)	24,354.24 (21,624.95–27,494.15)	23,311.51 (20,557.55–26,435.99)	21,423.15 (18,747.44–24,504.72)	20,316.42 (17,601.27–23,364.14)	19,746.88 (16,941.27–22,775.51)
Belgium	Group I	1,671.68 (1,490.25–1,911.40)	1,567.21 (1,391.77–1,800.45)	1,489.19 (1,315.93–1,717.34)	1,378.72 (1,208.88–1,603.68)	1,293.70 (1,123.59–1,533.35)	1,184.33 (1,005.35–1,409.20)
Belgium	NCD	20,404.81 (18,065.43–23,023.77)	19,539.00 (17,215.99–22,147.01)	18,679.41 (16,405.48–21,261.64)	17,355.89 (15,039.76–19,946.51)	16,607.14 (14,320.96–19,190.55)	16,303.83 (13,921.77–18,914.67)
Belgium	Injuries	3,423.26 (3,123.58–3,785.13)	3,248.03 (2,967.17–3,592.75)	3,142.91 (2,852.57–3,487.69)	2,688.54 (2,416.35–3,026.60)	2,415.59 (2,162.96–2,730.54)	2,258.73 (2,004.47–2,560.96)
Cyprus	All causes	25,074.89 (22,314.57–28,220.89)	24,477.10 (21,714.22–27,629.90)	22,914.45 (20,177.52–26,056.20)	21,434.71 (18,743.04–24,521.75)	19,602.23 (16,925.14–22,741.50)	18,323.38 (15,641.80–21,384.38)
Cyprus	Group I	2,421.90 (2,163.46–2,714.41)	2,039.91 (1,802.80–2,336.36)	1,708.21 (1,489.16–1,981.09)	1,502.93 (1,295.60–1,776.44)	1,386.56 (1,174.92–1,664.22)	1,294.05 (1,080.83–1,587.01)
Cyprus	NCD	19,672.84 (17,367.19–22,265.50)	19,500.61 (17,163.12–22,201.00)	18,429.66 (16,146.04–21,012.00)	17,263.65 (14,957.56–19,909.26)	16,206.86 (13,902.66–18,889.67)	15,299.91 (12,974.63–17,954.91)
Cyprus	Injuries	2,980.15 (2,679.29–3,349.05)	2,936.57 (2,639.17–3,309.17)	2,776.58 (2,500.66–3,129.79)	2,668.13 (2,381.86–3,015.70)	2,008.80 (1,764.19–2,322.52)	1,729.42 (1,497.16–2,034.04)
Denmark	All causes	26,780.57 (24,065.40–29,873.61)	25,961.17 (23,206.85–29,063.98)	23,857.59 (21,116.37–26,969.66)	22,299.81 (19,570.83–25,457.12)	20,937.85 (18,233.21–23,968.84)	19,816.08 (17,180.42–22,787.10)
Denmark	Group I	1,700.46 (1,505.93–1,964.69)	1,673.11 (1,472.62–1,934.10)	1,398.60 (1,187.70–1,678.62)	1,398.63 (1,178.51–1,683.85)	1,338.09 (1,115.90–1,624.61)	1,242.63 (1,020.95–1,531.42)
Denmark	NCD	21,778.22 (19,480.96–24,330.16)	21,520.76 (19,179.04–24,128.30)	20,162.11 (17,816.52–22,761.48)	18,997.49 (16,626.77–21,625.56)	17,993.44 (15,637.47–20,577.18)	17,123.35 (14,725.05–19,656.05)
Denmark	Injuries	3,301.89 (2,999.14–3,666.79)	2,767.30 (2,502.24–3,071.12)	2,296.88 (2,068.41–2,576.78)	1,903.68 (1,704.60–2,162.29)	1,606.32 (1,422.63–1,843.89)	1,450.10 (1,268.79–1,672.19)
Finland	All causes	26,831.21 (23,990.50–30,110.76)	24,516.59 (21,634.87–27,721.78)	23,216.89 (20,355.50–26,430.47)	22,028.77 (19,173.09–25,294.19)	20,757.18 (17,992.50–23,968.03)	19,645.32 (16,933.79–22,790.10)
Finland	Group I	1,907.29 (1,645.09–2,250.01)	1,680.80 (1,429.93–2,018.26)	1,455.75 (1,219.94–1,767.27)	1,236.12 (1,010.16–1,542.20)	1,081.67 (851.46–1,379.59)	991.52 (764.26–1,284.19)
Finland	NCD	20,785.14 (18,456.57–23,410.38)	19,380.01 (17,041.52–21,970.23)	18,654.89 (16,290.42–21,274.05)	17,870.10 (15,499.99–20,456.33)	17,124.67 (14,786.13–19,685.06)	16,414.50 (14,064.93–18,965.58)
Finland	Injuries	4,138.77 (3,818.18–4,512.96)	3,455.79 (3,176.57–3,784.22)	3,106.25 (2,845.53–3,426.36)	2,922.56 (2,636.93–3,280.07)	2,550.84 (2,275.80–2,888.59)	2,239.30 (1,977.46–2,569.34)
France	All causes	24,328.58 (21,584.32–27,528.53)	23,043.10 (20,311.21–26,219.41)	21,966.62 (19,256.66–25,102.49)	20,253.33 (17,572.10–23,398.53)	19,279.44 (16,631.67–22,343.33)	18,745.93 (16,183.78–21,761.56)
France	Group I	1,939.76 (1,734.18–2,215.01)	1,857.43 (1,657.43–2,151.99)	1,506.70 (1,302.48–1,794.75)	1,346.09 (1,139.95–1,639.74)	1,281.18 (1,072.42–1,566.79)	1,170.86 (949.35–1,473.87)
France	NCD	18,345.18 (16,145.60–20,821.41)	17,562.50 (15,336.25–20,050.12)	17,183.25 (14,995.48–19,624.50)	16,175.92 (13,967.52–18,644.33)	15,582.45 (13,360.00–18,078.25)	15,314.23 (13,111.15–17,787.47)
France	Injuries	4,043.64 (3,684.80–4,487.02)	3,623.17 (3,277.78–4,036.69)	3,276.67 (2,959.45–3,652.38)	2,731.32 (2,443.35–3,086.63)	2,415.81 (2,145.39–2,751.17)	2,260.85 (2,006.06–2,573.40)

Appendix Table 4. Rate of DALYs per 100,000 individuals for all causes and level 1 cause groups by location for 1990 - 2015, both sexes combined.

	Cause	1990	1995	2000	2005	2010	2015
Germany	All causes	26,395.21 (23,589.54–29,592.07)	24,664.75 (21,843.66–27,861.64)	22,871.62 (20,092.38–26,009.56)	21,123.08 (18,383.35–24,266.00)	20,014.32 (17,304.45–23,073.22)	19,399.06 (16,689.53–22,469.01)
Germany	Group I	1,799.18 (1,561.52–2,109.81)	1,548.33 (1,312.36–1,854.68)	1,410.18 (1,181.55–1,717.86)	1,347.34 (1,117.46–1,641.68)	1,294.60 (1,071.05–1,602.37)	1,234.33 (1,001.74–1,562.03)
Germany	NCD	21,729.12 (19,425.04–24,343.80)	20,530.53 (18,173.50–23,138.70)	19,195.89 (16,871.68–21,848.05)	17,884.43 (15,556.98–20,497.04)	17,060.13 (14,732.25–19,648.77)	16,603.31 (14,252.72–19,157.23)
Germany	Injuries	2,866.91 (2,578.14–3,207.33)	2,585.89 (2,313.50–2,902.61)	2,265.55 (2,013.83–2,564.88)	1,891.30 (1,659.90–2,178.02)	1,659.59 (1,443.17–1,920.08)	1,561.43 (1,354.35–1,824.95)
Greece	All causes	23,667.12 (21,007.92–26,738.49)	22,700.48 (20,055.84–25,816.20)	21,706.85 (19,097.41–24,743.97)	20,514.62 (17,880.62–23,512.96)	19,462.32 (16,851.77–22,510.59)	18,804.29 (16,201.40–21,726.51)
Greece	Group I	1,718.14 (1,498.49–2,057.65)	1,626.42 (1,412.94–1,942.86)	1,430.61 (1,216.86–1,710.77)	1,273.45 (1,060.23–1,550.40)	1,208.39 (1,005.29–1,485.79)	1,059.98 (850.97–1,335.35)
Greece	NCD	19,048.12 (16,865.78–21,504.16)	18,391.46 (16,201.11–20,832.65)	17,818.97 (15,629.61–20,283.93)	17,062.92 (14,872.53–19,489.67)	16,395.27 (14,199.35–18,850.63)	16,095.73 (13,850.74–18,571.36)
Greece	Injuries	2,900.86 (2,572.91–3,281.85)	2,682.60 (2,380.08–3,036.75)	2,457.27 (2,176.06–2,791.67)	2,178.26 (1,926.25–2,491.58)	1,858.66 (1,629.42–2,148.46)	1,648.58 (1,438.14–1,918.40)
Iceland	All causes	22,238.96 (19,619.82–25,289.13)	22,762.28 (19,676.12–26,241.03)	19,562.61 (16,926.63–22,557.81)	18,307.07 (15,641.98–21,329.57)	17,104.50 (14,535.13–19,973.02)	16,587.07 (13,941.27–19,523.43)
Iceland	Group I	1,615.58 (1,429.25–1,860.13)	1,516.37 (1,325.03–1,763.95)	1,269.16 (1,075.18–1,523.40)	1,138.68 (943.87–1,399.58)	1,059.53 (860.38–1,318.48)	997.63 (804.23–1,259.30)
Iceland	NCD	17,922.84 (15,683.70–20,474.72)	17,105.90 (14,831.07–19,626.67)	16,139.16 (13,896.36–18,656.95)	15,211.98 (12,974.97–17,674.88)	14,434.41 (12,177.58–16,871.64)	14,120.24 (11,801.43–16,617.18)
Iceland	Injuries	2,700.53 (2,414.07–3,039.96)	4,140.01 (3,292.63–5,196.78)	2,154.29 (1,902.28–2,467.88)	1,956.41 (1,702.68–2,265.94)	1,610.56 (1,410.23–1,850.72)	1,469.19 (1,275.30–1,703.62)
Ireland	All causes	25,285.66 (22,422.02–28,536.90)	23,997.21 (21,237.82–27,219.27)	23,182.04 (20,377.88–26,386.93)	20,799.29 (18,001.10–24,054.03)	19,432.66 (16,608.76–22,539.47)	18,751.17 (16,075.51–21,869.18)
Ireland	Group I	1,916.54 (1,681.80–2,225.75)	1,808.54 (1,577.17–2,113.99)	1,704.90 (1,478.08–2,003.70)	1,432.50 (1,207.46–1,737.81)	1,319.50 (1,091.09–1,647.03)	1,209.06 (980.98–1,523.36)
Ireland	NCD	20,630.05 (18,176.62–23,347.50)	19,753.95 (17,401.65–22,469.99)	19,121.15 (16,754.14–21,793.54)	17,495.97 (15,096.81–20,198.92)	16,487.16 (14,072.14–19,145.90)	16,051.99 (13,633.35–18,677.03)
Ireland	Injuries	2,739.07 (2,444.80–3,077.10)	2,434.72 (2,184.04–2,735.48)	2,356.00 (2,120.90–2,645.93)	1,870.81 (1,649.51–2,138.21)	1,626.00 (1,427.51–1,871.46)	1,490.12 (1,303.64–1,725.07)
Israel	All causes	24,288.52 (21,622.21–27,430.76)	23,238.68 (20,557.42–26,298.62)	21,885.25 (19,212.66–24,931.20)	20,509.31 (17,869.25–23,468.47)	18,520.97 (15,893.74–21,519.88)	18,017.99 (15,392.53–20,990.82)
Israel	Group I	2,044.49 (1,790.33–2,367.73)	1,793.21 (1,543.31–2,101.85)	1,682.77 (1,451.40–2,009.45)	1,529.61 (1,305.01–1,852.28)	1,419.45 (1,180.99–1,752.37)	1,314.10 (1,078.32–1,634.94)
Israel	NCD	19,405.78 (17,214.71–21,951.43)	18,726.61 (16,515.35–21,235.91)	17,619.03 (15,425.54–20,112.42)	16,552.03 (14,369.53–19,005.89)	15,148.29 (12,935.87–17,622.68)	14,878.90 (12,652.57–17,441.42)
Israel	Injuries	2,838.25 (2,522.92–3,205.82)	2,718.86 (2,428.09–3,071.01)	2,583.45 (2,293.86–2,917.80)	2,427.67 (2,156.59–2,754.63)	1,953.23 (1,723.11–2,254.32)	1,824.98 (1,597.03–2,119.64)
Italy	All causes	24,025.64 (21,239.67–27,154.51)	22,610.54 (19,865.57–25,665.38)	20,911.84 (18,163.57–23,979.49)	18,990.13 (16,338.68–22,010.35)	17,998.50 (15,374.67–21,012.63)	17,734.12 (15,117.64–20,803.46)
Italy	Group I	1,821.34 (1,645.35–2,054.03)	1,756.95 (1,584.21–1,980.16)	1,275.96 (1,109.58–1,487.81)	1,113.33 (960.51–1,320.56)	1,056.22 (891.72–1,271.00)	943.30 (778.87–1,163.24)
Italy	NCD	19,486.89 (17,190.06–22,094.60)	18,362.41 (16,070.30–20,941.67)	17,359.12 (15,064.12–19,960.39)	15,963.80 (13,681.35–18,549.37)	15,353.21 (13,066.97–17,915.48)	15,306.37 (12,974.68–17,923.54)
Italy	Injuries	2,717.41 (2,421.45–3,078.92)	2,491.17 (2,209.59–2,833.25)	2,276.75 (2,018.34–2,600.97)	1,913.00 (1,678.93–2,206.70)	1,589.08 (1,375.67–1,860.86)	1,484.44 (1,283.73–1,745.86)
Luxembourg	All causes	27,240.40 (24,368.83–30,576.11)	24,779.52 (21,850.73–28,075.16)	22,684.66 (19,820.49–25,947.51)	20,762.16 (17,952.47–23,955.89)	19,116.64 (16,348.18–22,335.71)	18,274.45 (15,504.38–21,440.86)
Luxembourg	Group I	1,671.10 (1,451.38–1,952.19)	1,461.02 (1,244.95–1,735.92)	1,302.84 (1,098.82–1,565.82)	1,196.44 (984.38–1,462.10)	1,132.29 (920.27–1,398.72)	1,065.71 (850.86–1,340.14)
Luxembourg	NCD	21,964.92 (19,582.94–24,731.83)	20,251.98 (17,793.17–23,024.52)	18,782.99 (16,320.19–21,489.15)	17,432.52 (15,007.59–20,128.66)	16,251.82 (13,851.52–18,989.45)	15,620.50 (13,222.13–18,342.06)

Appendix Table 4. Rate of DALYs per 100,000 individuals for all causes and level 1 cause groups by location for 1990 - 2015, both sexes combined.

	Cause	1990	1995	2000	2005	2010	2015
Luxembourg	Injuries	3,604.39 (3,245.09–4,047.56)	3,066.51 (2,730.98–3,464.53)	2,598.83 (2,294.39–2,961.58)	2,133.20 (1,873.54–2,458.85)	1,732.53 (1,496.12–2,023.00)	1,588.25 (1,366.82–1,867.69)
Malta	All causes	23,903.59 (21,137.81–27,117.48)	22,368.51 (19,600.31–25,626.99)	21,483.86 (18,614.57–24,687.04)	19,792.87 (17,056.13–22,920.65)	19,100.19 (16,355.19–22,206.51)	18,472.25 (15,799.36–21,592.88)
Malta	Group I	1,891.87 (1,662.15–2,179.65)	1,902.23 (1,666.48–2,201.31)	1,683.90 (1,461.43–1,966.70)	1,544.40 (1,324.73–1,826.03)	1,481.24 (1,254.53–1,768.91)	1,405.71 (1,182.30–1,687.77)
Malta	NCD	19,883.03 (17,573.61–22,544.07)	18,549.23 (16,236.78–21,303.45)	17,981.81 (15,571.01–20,763.70)	16,661.96 (14,333.15–19,401.90)	16,191.17 (13,816.12–18,841.93)	15,760.56 (13,455.89–18,476.68)
Malta	Injuries	2,128.69 (1,846.36–2,463.42)	1,917.05 (1,648.05–2,234.39)	1,818.15 (1,557.03–2,125.23)	1,586.52 (1,346.05–1,878.43)	1,427.78 (1,208.03–1,715.95)	1,305.98 (1,088.43–1,580.10)
Netherlands	All causes	24,000.70 (21,241.61–27,095.83)	23,216.23 (20,481.22–26,361.51)	22,584.47 (19,802.62–25,729.81)	20,735.99 (17,966.66–23,873.18)	19,339.83 (16,636.75–22,478.90)	18,794.83 (16,058.13–21,786.90)
Netherlands	Group I	1,586.65 (1,396.47–1,844.80)	1,569.54 (1,382.05–1,816.56)	1,539.71 (1,353.44–1,783.03)	1,383.25 (1,206.22–1,630.02)	1,236.52 (1,062.32–1,482.75)	1,164.80 (985.17–1,415.41)
Netherlands	NCD	20,278.38 (17,936.15–22,897.46)	19,670.41 (17,266.35–22,342.26)	19,170.25 (16,724.88–21,826.17)	17,732.28 (15,298.72–20,416.71)	16,645.38 (14,270.10–19,297.49)	16,221.63 (13,818.04–18,824.56)
Netherlands	Injuries	2,135.67 (1,920.37–2,391.64)	1,976.28 (1,765.46–2,229.63)	1,874.51 (1,676.64–2,112.50)	1,620.46 (1,423.67–1,851.09)	1,457.93 (1,276.64–1,693.53)	1,408.40 (1,226.32–1,643.05)
Norway	All causes	24,783.18 (22,029.72–27,934.74)	22,914.48 (20,170.77–26,069.81)	22,281.69 (19,442.52–25,404.95)	20,475.90 (17,710.14–23,627.87)	19,486.02 (16,751.71–22,627.52)	18,323.32 (15,619.69–21,379.61)
Norway	Group I	1,759.41 (1,581.72–1,993.78)	1,435.45 (1,245.48–1,688.57)	1,305.57 (1,115.68–1,567.20)	1,193.21 (1,002.13–1,444.52)	1,122.43 (923.10–1,392.19)	1,054.06 (852.41–1,318.90)
Norway	NCD	19,990.12 (17,635.49–22,606.14)	19,087.11 (16,675.07–21,750.22)	18,647.90 (16,188.76–21,416.77)	17,285.17 (14,822.02–19,995.99)	16,573.24 (14,185.64–19,273.28)	15,756.15 (13,300.91–18,353.21)
Norway	Injuries	3,033.65 (2,750.15–3,369.09)	2,391.91 (2,159.68–2,690.38)	2,328.22 (2,090.26–2,609.14)	1,997.51 (1,776.80–2,278.24)	1,790.35 (1,589.23–2,047.69)	1,513.10 (1,332.70–1,751.22)
Portugal	All causes	28,882.18 (26,040.69–32,169.41)	26,878.99 (24,096.68–30,184.01)	24,905.51 (22,153.91–28,154.89)	22,469.70 (19,748.69–25,687.94)	20,531.50 (17,873.26–23,681.51)	19,462.46 (16,800.49–22,635.91)
Portugal	Group I	2,591.37 (2,369.40–2,922.84)	2,618.50 (2,396.96–2,923.29)	2,456.53 (2,221.36–2,768.65)	2,058.86 (1,829.91–2,356.38)	1,735.93 (1,510.86–2,037.49)	1,561.49 (1,331.92–1,866.20)
Portugal	NCD	21,762.75 (19,439.11–24,458.98)	20,447.62 (18,196.78–23,157.91)	19,295.12 (17,016.01–21,989.07)	17,866.62 (15,558.51–20,555.07)	16,745.98 (14,448.41–19,416.55)	16,097.94 (13,770.25–18,764.62)
Portugal	Injuries	4,528.05 (4,133.49–4,997.36)	3,812.86 (3,463.98–4,237.08)	3,153.86 (2,838.53–3,537.52)	2,544.21 (2,256.15–2,877.73)	2,049.60 (1,810.35–2,349.63)	1,803.04 (1,588.87–2,082.62)
Spain	All causes	24,260.40 (21,522.49–27,399.46)	22,867.61 (20,161.89–25,977.59)	21,181.36 (18,484.04–24,267.09)	19,708.89 (17,060.83–22,769.43)	17,982.22 (15,379.77–20,964.28)	17,118.08 (14,544.87–20,046.92)
Spain	Group I	2,220.31 (1,942.61–2,565.33)	2,381.24 (2,103.96–2,735.84)	1,762.22 (1,496.77–2,122.36)	1,627.97 (1,366.23–1,979.50)	1,460.01 (1,188.79–1,806.75)	1,336.59 (1,061.74–1,703.07)
Spain	NCD	18,890.33 (16,772.57–21,366.50)	17,914.29 (15,771.85–20,364.78)	17,035.81 (14,894.38–19,458.72)	16,069.32 (13,944.52–18,468.41)	14,985.38 (12,864.11–17,410.09)	14,412.28 (12,239.59–16,798.13)
Spain	Injuries	3,149.75 (2,835.53–3,529.07)	2,572.08 (2,285.54–2,922.54)	2,383.34 (2,114.74–2,704.90)	2,011.60 (1,772.24–2,327.60)	1,536.84 (1,320.91–1,810.48)	1,369.21 (1,179.54–1,618.77)
Sweden	All causes	22,591.52 (20,002.07–25,539.54)	20,974.21 (18,381.71–23,927.13)	20,109.52 (17,470.33–23,064.55)	19,199.82 (16,583.72–22,135.90)	18,184.84 (15,590.59–21,027.58)	17,748.89 (15,165.63–20,565.24)
Sweden	Group I	1,480.16 (1,286.39–1,746.12)	1,305.84 (1,106.35–1,592.93)	1,209.54 (995.04–1,498.33)	1,160.43 (936.64–1,458.14)	1,129.70 (897.71–1,426.79)	1,064.30 (843.19–1,359.56)
Sweden	NCD	18,646.57 (16,417.93–21,099.70)	17,516.51 (15,277.78–19,987.22)	16,946.67 (14,671.70–19,415.52)	16,219.57 (14,046.08–18,671.21)	15,418.79 (13,205.85–17,857.29)	15,144.10 (12,882.21–17,534.03)
Sweden	Injuries	2,464.79 (2,250.30–2,729.98)	2,151.85 (1,945.37–2,412.56)	1,953.31 (1,753.64–2,207.54)	1,819.82 (1,631.70–2,062.00)	1,636.35 (1,455.48–1,873.19)	1,540.49 (1,363.28–1,767.81)
Switzerland	All causes	24,098.14 (21,296.47–27,350.15)	22,675.53 (19,911.39–25,906.68)	21,124.98 (18,311.48–24,348.19)	19,406.41 (16,651.27–22,554.16)	18,183.89 (15,518.88–21,289.83)	17,467.76 (14,766.83–20,500.83)
Switzerland	Group I	1,695.38 (1,485.27–1,971.21)	1,743.98 (1,531.44–2,017.51)	1,411.30 (1,202.39–1,686.23)	1,253.50 (1,049.52–1,538.32)	1,179.62 (981.39–1,461.00)	1,121.75 (899.99–1,393.78)

Appendix Table 4. Rate of DALYs per 100,000 individuals for all causes and level 1 cause groups by location for 1990 - 2015, both sexes combined.							
	Cause	1990	1995	2000	2005	2010	2015
Switzerland	NCD	18,526.28 (16,261.97–21,090.58)	17,878.79 (15,603.82–20,460.01)	17,075.29 (14,731.84–19,665.10)	15,986.65 (13,629.31–18,613.36)	15,185.30 (12,870.68–17,768.81)	14,668.28 (12,373.04–17,208.87)
Switzerland	Injuries	3,876.49 (3,480.55–4,354.10)	3,052.77 (2,720.67–3,455.85)	2,638.40 (2,336.22–2,990.76)	2,166.26 (1,905.18–2,483.10)	1,818.98 (1,580.20–2,124.57)	1,677.73 (1,460.65–1,959.47)
United Kingdom	All causes	25,558.61 (22,855.56–28,645.61)	24,161.61 (21,439.89–27,283.75)	22,900.67 (20,191.09–26,010.95)	21,499.93 (18,778.85–24,619.61)	20,134.38 (17,470.97–23,179.24)	19,321.03 (16,682.76–22,315.21)
United Kingdom	Group I	2,073.21 (1,859.84–2,351.88)	2,049.10 (1,827.58–2,336.40)	1,922.82 (1,702.38–2,216.67)	1,756.17 (1,529.36–2,051.83)	1,598.66 (1,378.95–1,894.50)	1,494.25 (1,274.16–1,788.04)
United Kingdom	NCD	21,266.18 (18,947.68–23,855.84)	20,091.80 (17,770.93–22,670.64)	19,068.38 (16,750.27–21,660.06)	18,027.00 (15,698.22–20,616.71)	17,002.53 (14,680.40–19,566.09)	16,465.77 (14,160.43–19,006.01)
United Kingdom	Injuries	2,219.22 (1,976.55–2,512.05)	2,020.71 (1,793.32–2,299.88)	1,909.47 (1,683.49–2,181.64)	1,716.76 (1,499.92–1,984.12)	1,533.19 (1,332.83–1,785.35)	1,361.01 (1,176.29–1,593.94)
England	All causes	25,165.31 (22,481.64–28,226.28)	23,763.00 (21,069.93–26,852.96)	22,498.06 (19,810.23–25,590.93)	21,082.37 (18,392.31–24,159.56)	19,709.80 (17,082.99–22,727.32)	18,942.19 (16,317.93–21,918.89)
England	Group I	2,065.05 (1,847.47–2,348.01)	2,049.65 (1,825.08–2,345.45)	1,929.10 (1,707.35–2,232.69)	1,754.41 (1,525.91–2,052.26)	1,600.29 (1,376.07–1,901.14)	1,492.16 (1,267.17–1,788.47)
England	NCD	20,956.70 (18,651.53–23,517.13)	19,771.77 (17,464.94–22,331.20)	18,735.02 (16,427.50–21,312.29)	17,686.62 (15,370.71–20,259.02)	16,663.41 (14,367.24–19,207.63)	16,153.56 (13,871.27–18,665.31)
England	Injuries	2,143.56 (1,905.57–2,430.60)	1,941.58 (1,716.35–2,220.69)	1,833.93 (1,611.13–2,100.03)	1,641.35 (1,430.99–1,905.28)	1,446.10 (1,251.40–1,697.83)	1,296.47 (1,115.10–1,522.99)
Northern Ireland	All causes	26,436.57 (23,760.45–29,515.62)	24,630.14 (21,973.64–27,674.58)	23,054.75 (20,320.29–26,208.74)	22,231.44 (19,519.25–25,356.73)	21,586.26 (18,866.85–24,637.14)	20,117.34 (17,495.75–23,143.61)
Northern Ireland	Group I	2,359.24 (2,157.92–2,647.54)	2,248.13 (2,027.34–2,540.83)	1,991.47 (1,760.25–2,293.07)	1,837.46 (1,615.47–2,134.81)	1,696.06 (1,475.01–1,988.92)	1,563.16 (1,326.17–1,872.28)
Northern Ireland	NCD	21,391.91 (19,131.43–23,922.50)	19,939.27 (17,651.53–22,496.59)	18,785.32 (16,451.64–21,391.30)	18,105.31 (15,821.33–20,676.84)	17,370.13 (15,120.98–19,884.13)	16,676.51 (14,442.32–19,187.44)
Northern Ireland	Injuries	2,685.43 (2,416.40–3,030.76)	2,442.74 (2,180.61–2,750.90)	2,277.96 (2,023.81–2,587.01)	2,288.67 (2,015.03–2,600.98)	2,520.08 (2,149.99–2,940.95)	1,877.67 (1,639.83–2,191.32)
Scotland	All causes	28,743.45 (25,872.30–32,008.58)	27,330.27 (24,370.43–30,638.86)	26,187.83 (23,291.94–29,507.05)	24,727.39 (21,782.33–28,001.94)	23,075.45 (20,178.08–26,302.07)	21,961.28 (19,119.21–25,102.78)
Scotland	Group I	2,082.93 (1,886.34–2,341.46)	1,963.75 (1,767.30–2,221.74)	1,804.19 (1,603.74–2,068.35)	1,722.19 (1,517.62–1,992.95)	1,487.87 (1,287.85–1,750.12)	1,415.16 (1,211.05–1,680.43)
Scotland	NCD	23,878.15 (21,452.44–26,647.35)	22,779.05 (20,295.71–25,547.13)	21,910.98 (19,459.52–24,677.71)	20,786.02 (18,214.77–23,541.74)	19,592.20 (17,072.65–22,357.29)	18,783.39 (16,226.90–21,535.09)
Scotland	Injuries	2,782.37 (2,504.47–3,141.09)	2,587.47 (2,324.79–2,917.19)	2,472.66 (2,221.12–2,791.93)	2,219.18 (1,968.84–2,523.11)	1,995.38 (1,765.41–2,281.41)	1,762.72 (1,551.05–2,019.96)
Wales	All causes	26,048.28 (23,182.75–29,280.36)	24,931.54 (22,063.82–28,188.30)	23,797.48 (20,965.21–27,021.41)	22,493.23 (19,639.08–25,679.84)	21,394.99 (18,543.37–24,568.69)	20,744.39 (17,884.29–23,924.34)
Wales	Group I	2,075.06 (1,826.38–2,374.36)	2,089.16 (1,831.63–2,412.49)	1,990.37 (1,741.58–2,309.46)	1,799.55 (1,532.86–2,120.56)	1,681.84 (1,421.55–1,992.92)	1,613.06 (1,348.87–1,941.04)
Wales	NCD	21,746.35 (19,323.65–24,479.64)	20,746.37 (18,311.96–23,417.50)	19,814.02 (17,394.78–22,485.84)	18,882.79 (16,469.52–21,569.00)	18,050.71 (15,610.25–20,676.74)	17,631.25 (15,180.86–20,344.55)
Wales	Injuries	2,226.87 (1,979.05–2,528.34)	2,096.01 (1,860.52–2,378.10)	1,993.08 (1,761.56–2,279.87)	1,810.89 (1,582.41–2,092.07)	1,662.43 (1,444.98–1,939.45)	1,500.08 (1,306.70–1,756.68)
Southern Latin America	All causes	30,732.87 (28,144.44–33,750.06)	28,724.94 (26,131.11–31,698.83)	27,101.34 (24,490.22–30,113.66)	25,481.52 (22,900.03–28,419.83)	24,687.15 (22,116.16–27,604.01)	23,733.05 (21,107.48–26,721.07)
Southern Latin America	Group I	4,687.37 (4,399.86–5,062.48)	4,120.13 (3,841.48–4,473.28)	3,701.23 (3,436.07–4,024.38)	3,357.95 (3,092.68–3,694.19)	3,107.16 (2,843.55–3,433.94)	2,773.29 (2,517.11–3,087.90)
Southern Latin America	NCD	22,499.98 (20,382.12–24,887.11)	21,149.08 (19,047.23–23,563.34)	20,108.89 (17,991.49–22,517.37)	19,175.17 (17,041.32–21,586.96)	18,677.05 (16,539.96–21,084.67)	18,238.71 (16,053.47–20,693.07)
Southern Latin America	Injuries	3,545.53 (3,291.65–3,851.34)	3,455.73 (3,202.42–3,762.50)	3,291.23 (3,046.40–3,590.95)	2,948.40 (2,719.56–3,240.55)	2,902.94 (2,682.38–3,193.30)	2,721.05 (2,496.90–3,007.74)
Argentina	All causes	31,236.31 (28,616.87–34,269.89)	29,617.30 (27,015.66–32,612.71)	28,417.52 (25,774.65–31,443.35)	26,663.11 (24,048.38–29,617.84)	25,720.57 (23,126.21–28,646.85)	24,854.23 (22,211.54–27,907.17)

Appendix Table 4. Rate of DALYs per 100,000 individuals for all causes and level 1 cause groups by location for 1990 - 2015, both sexes combined.

	Cause	1990	1995	2000	2005	2010	2015
Argentina	Group I	5,042.73 (4,707.29–5,471.90)	4,591.67 (4,269.33–4,983.77)	4,238.73 (3,938.85–4,599.17)	3,894.69 (3,596.04–4,279.59)	3,594.02 (3,300.20–3,950.94)	3,202.38 (2,921.90–3,548.67)
Argentina	NCD	22,793.09 (20,672.59–25,177.66)	21,636.83 (19,552.93–24,038.40)	20,777.07 (18,647.51–23,156.74)	19,730.35 (17,604.82–22,138.25)	19,214.56 (17,095.93–21,631.52)	18,849.57 (16,659.47–21,337.13)
Argentina	Injuries	3,400.50 (3,137.55–3,703.20)	3,388.80 (3,132.65–3,702.61)	3,401.73 (3,148.87–3,714.16)	3,038.07 (2,796.56–3,342.32)	2,912.00 (2,682.75–3,212.14)	2,802.27 (2,569.82–3,107.69)
Chile	All causes	29,663.38 (27,079.42–32,608.96)	26,184.85 (23,635.03–29,113.16)	23,500.76 (20,917.99–26,423.58)	22,265.36 (19,750.97–25,167.88)	22,036.37 (19,448.39–24,930.88)	20,937.83 (18,322.87–23,725.41)
Chile	Group I	4,104.46 (3,874.86–4,403.50)	3,091.75 (2,883.26–3,351.42)	2,411.44 (2,215.14–2,669.58)	2,013.07 (1,817.14–2,276.28)	1,932.68 (1,731.35–2,193.34)	1,739.86 (1,535.36–1,991.39)
Chile	NCD	21,628.58 (19,478.17–24,077.62)	19,538.43 (17,421.15–21,953.99)	18,127.81 (15,939.88–20,571.37)	17,592.93 (15,447.35–20,028.59)	17,298.06 (15,155.23–19,707.52)	16,753.01 (14,502.52–19,131.01)
Chile	Injuries	3,930.34 (3,674.15–4,261.80)	3,554.67 (3,301.11–3,847.88)	2,961.50 (2,743.50–3,235.83)	2,659.36 (2,458.62–2,917.45)	2,805.63 (2,534.59–3,134.93)	2,444.96 (2,208.69–2,727.07)
Uruguay	All causes	29,941.56 (27,440.69–32,971.41)	29,402.27 (26,820.16–32,367.77)	27,401.64 (24,853.15–30,324.80)	25,919.79 (23,358.15–28,856.23)	24,826.34 (22,250.07–27,768.02)	24,174.18 (21,567.49–27,167.97)
Uruguay	Group I	3,920.98 (3,628.29–4,290.37)	3,702.01 (3,414.25–4,044.98)	3,166.39 (2,884.41–3,519.61)	2,903.78 (2,604.34–3,279.24)	2,525.54 (2,223.91–2,894.23)	2,349.67 (2,010.01–2,751.51)
Uruguay	NCD	22,621.03 (20,534.96–25,018.16)	22,043.19 (19,900.68–24,434.39)	20,804.79 (18,684.16–23,171.59)	19,875.55 (17,738.77–22,257.05)	19,134.74 (16,967.14–21,522.34)	18,808.88 (16,627.43–21,237.90)
Uruguay	Injuries	3,399.55 (3,145.76–3,712.22)	3,657.08 (3,408.46–3,954.36)	3,430.45 (3,200.12–3,724.75)	3,140.46 (2,919.89–3,407.24)	3,166.06 (2,949.96–3,444.55)	3,015.64 (2,788.50–3,296.62)
Central Europe, Eastern Europe, and Central Asia	All causes	38,139.52 (35,176.29–41,466.28)	43,885.62 (40,990.06–47,166.22)	41,302.12 (38,394.74–44,586.49)	39,754.39 (36,928.71–42,955.00)	34,118.65 (31,316.88–37,349.13)	31,158.70 (28,311.67–34,337.17)
Central Europe, Eastern Europe, and Central Asia	Group I	5,852.06 (5,510.53–6,280.41)	6,263.12 (5,903.06–6,716.30)	5,786.30 (5,421.66–6,184.04)	5,047.54 (4,694.13–5,468.66)	4,208.87 (3,857.20–4,622.75)	3,627.36 (3,283.14–4,037.34)
Central Europe, Eastern Europe, and Central Asia	NCD	26,847.71 (24,527.62–29,420.03)	30,482.66 (28,195.88–33,025.88)	29,218.51 (26,896.01–31,788.24)	28,852.45 (26,541.13–31,408.72)	25,522.18 (23,194.62–28,097.84)	23,634.06 (21,304.34–26,265.43)
Central Europe, Eastern Europe, and Central Asia	Injuries	5,439.74 (5,141.48–5,775.54)	7,139.84 (6,816.84–7,494.47)	6,297.31 (5,997.31–6,622.74)	5,854.41 (5,593.63–6,166.11)	4,387.59 (4,164.45–4,645.58)	3,897.28 (3,682.85–4,173.35)
Eastern Europe	All causes	37,660.24 (34,614.36–41,102.86)	46,842.98 (43,846.24–50,229.91)	45,112.30 (42,103.05–48,537.73)	44,705.60 (41,755.57–48,060.68)	37,000.39 (34,058.15–40,336.14)	33,761.31 (30,763.09–37,102.90)
Eastern Europe	Group I	3,966.08 (3,607.02–4,419.61)	4,440.65 (4,059.93–4,893.10)	4,635.85 (4,272.50–5,075.21)	4,300.34 (3,931.49–4,772.48)	3,640.45 (3,266.41–4,103.30)	3,279.25 (2,914.31–3,756.32)
Eastern Europe	NCD	27,330.59 (24,975.05–29,932.77)	33,287.44 (31,019.24–35,863.60)	32,036.50 (29,703.29–34,680.73)	32,395.67 (30,066.73–34,980.00)	27,736.71 (25,333.35–30,319.78)	25,505.00 (23,034.23–28,150.12)
Eastern Europe	Injuries	6,363.58 (6,025.63–6,752.32)	9,114.89 (8,742.33–9,529.91)	8,439.94 (8,070.63–8,857.37)	8,009.59 (7,647.15–8,406.89)	5,623.23 (5,364.25–5,930.10)	4,977.06 (4,701.79–5,304.24)
Belarus	All causes	35,330.39 (32,369.12–38,622.69)	39,589.10 (36,679.38–42,790.48)	39,697.86 (36,761.02–43,029.93)	38,209.57 (35,395.58–41,420.20)	34,963.17 (32,090.00–38,212.68)	33,504.72 (30,485.83–36,771.00)
Belarus	Group I	3,379.83 (2,907.70–3,943.47)	3,257.58 (2,799.20–3,800.38)	2,969.99 (2,586.17–3,445.79)	2,605.01 (2,246.59–3,055.31)	2,239.52 (1,883.16–2,655.91)	1,944.10 (1,607.37–2,347.60)
Belarus	NCD	26,094.25 (23,678.83–28,731.82)	29,263.02 (26,945.09–31,866.65)	29,555.82 (27,245.20–32,154.86)	29,022.99 (26,706.00–31,584.15)	27,048.27 (24,689.58–29,607.70)	26,276.98 (23,757.42–28,924.71)
Belarus	Injuries	5,856.32 (5,480.92–6,248.24)	7,068.50 (6,657.53–7,472.40)	7,172.05 (6,735.01–7,588.54)	6,581.58 (6,229.67–6,975.92)	5,675.37 (5,357.53–6,047.77)	5,283.64 (4,831.73–5,842.37)
Estonia	All causes	36,114.95 (33,171.31–39,407.88)	40,840.41 (37,926.99–44,087.94)	35,251.32 (32,422.27–38,499.68)	31,086.77 (28,322.96–34,228.78)	25,005.12 (22,265.20–28,062.82)	22,790.12 (20,030.09–25,758.97)
Estonia	Group I	3,040.08 (2,711.21–3,436.71)	3,422.44 (3,087.64–3,807.72)	2,685.84 (2,395.44–3,065.54)	2,162.49 (1,881.63–2,546.50)	1,724.84 (1,437.98–2,074.15)	1,449.05 (1,174.66–1,795.47)
Estonia	NCD	25,915.40 (23,611.40–28,460.54)	28,699.77 (26,407.66–31,254.23)	26,292.66 (23,940.04–28,872.37)	24,197.40 (21,876.63–26,839.42)	20,361.04 (17,968.76–22,902.91)	19,036.42 (16,613.09–21,566.40)
Estonia	Injuries	7,159.46 (6,754.70–7,614.25)	8,718.20 (8,310.96–9,155.59)	6,272.82 (5,941.44–6,639.98)	4,726.89 (4,469.93–5,017.77)	2,919.25 (2,717.87–3,163.49)	2,304.65 (2,101.34–2,569.86)

Appendix Table 4. Rate of DALYs per 100,000 individuals for all causes and level 1 cause groups by location for 1990 - 2015, both sexes combined.

	Cause	1990	1995	2000	2005	2010	2015
Latvia	All causes	37,128.88 (34,240.49–40,408.22)	43,359.43 (40,514.53–46,712.49)	35,220.95 (32,364.19–38,468.26)	33,621.30 (30,801.35–36,783.91)	28,596.58 (25,860.68–31,649.37)	26,262.14 (23,426.29–29,337.76)
Latvia	Group I	3,133.32 (2,807.96–3,518.54)	3,631.57 (3,300.15–4,045.93)	2,868.68 (2,565.28–3,274.26)	2,470.74 (2,169.27–2,844.25)	2,140.66 (1,838.85–2,491.69)	1,840.10 (1,521.56–2,232.26)
Latvia	NCD	26,524.09 (24,201.91–29,074.18)	30,222.51 (27,925.18–32,760.65)	25,796.96 (23,425.09–28,384.11)	25,621.40 (23,278.64–28,191.79)	22,584.77 (20,241.48–25,073.92)	21,147.71 (18,731.27–23,700.85)
Latvia	Injuries	7,471.46 (7,085.56–7,915.11)	9,505.34 (9,066.55–9,962.47)	6,555.30 (6,239.84–6,938.53)	5,529.16 (5,235.16–5,853.84)	3,871.15 (3,651.42–4,136.17)	3,274.34 (3,052.43–3,550.58)
Lithuania	All causes	33,615.43 (30,708.29–36,857.17)	38,068.22 (35,192.03–41,358.28)	32,578.07 (29,701.97–35,838.87)	32,837.97 (30,060.70–36,016.21)	28,995.85 (26,224.21–32,093.68)	26,876.49 (24,135.93–29,958.76)
Lithuania	Group I	2,427.20 (2,136.40–2,777.80)	2,787.78 (2,491.35–3,153.05)	2,373.36 (2,102.52–2,730.45)	2,252.26 (1,980.51–2,601.46)	1,935.88 (1,657.16–2,274.57)	1,679.77 (1,396.03–2,005.51)
Lithuania	NCD	24,524.34 (22,181.92–27,084.72)	27,357.72 (25,043.79–29,951.65)	23,856.22 (21,516.24–26,414.95)	24,422.94 (22,133.84–26,973.06)	22,343.88 (20,041.08–24,913.37)	21,004.20 (18,570.08–23,563.38)
Lithuania	Injuries	6,663.89 (6,309.92–7,061.69)	7,922.71 (7,582.97–8,315.01)	6,348.50 (6,034.30–6,728.84)	6,162.76 (5,891.32–6,483.37)	4,716.09 (4,483.15–5,009.49)	4,192.52 (3,955.55–4,476.74)
Moldova	All causes	40,008.72 (36,968.11–43,463.10)	46,066.90 (42,986.69–49,335.75)	39,068.95 (36,008.38–42,270.66)	36,248.60 (33,377.85–39,451.68)	34,121.93 (31,322.32–37,247.09)	28,336.23 (25,461.86–31,379.33)
Moldova	Group I	4,888.31 (4,306.10–5,545.80)	5,780.91 (5,119.52–6,566.77)	5,584.09 (4,956.04–6,358.91)	3,837.38 (3,365.64–4,452.00)	3,578.72 (3,137.82–4,162.17)	2,713.09 (2,261.74–3,275.36)
Moldova	NCD	28,883.14 (26,591.80–31,509.01)	33,634.57 (31,274.11–36,245.58)	28,526.36 (26,179.33–31,134.56)	28,242.92 (25,969.81–30,810.62)	26,852.39 (24,590.55–29,413.94)	22,780.76 (20,482.96–25,302.52)
Moldova	Injuries	6,237.28 (5,827.25–6,659.93)	6,651.42 (6,266.94–7,073.35)	4,958.51 (4,648.36–5,327.71)	4,168.30 (3,916.19–4,453.45)	3,690.82 (3,480.50–3,939.71)	2,842.38 (2,632.61–3,091.90)
Russia	All causes	38,399.54 (35,314.35–41,944.70)	48,867.64 (45,816.01–52,417.92)	47,367.50 (44,303.52–50,916.73)	47,001.61 (43,969.06–50,447.05)	38,489.05 (35,465.14–41,912.62)	34,587.17 (31,511.30–38,035.96)
Russia	Group I	4,210.49 (3,810.94–4,744.21)	4,732.21 (4,287.02–5,245.53)	4,938.82 (4,534.86–5,466.33)	4,573.45 (4,151.48–5,096.04)	3,877.10 (3,454.38–4,394.64)	3,567.86 (3,139.38–4,119.39)
Russia	NCD	27,585.88 (25,250.16–30,177.20)	34,240.96 (31,965.89–36,848.45)	33,186.67 (30,831.87–35,842.75)	33,479.10 (31,159.80–36,073.37)	28,352.98 (25,942.44–30,944.16)	25,704.12 (23,226.78–28,418.76)
Russia	Injuries	6,603.17 (6,234.29–7,018.94)	9,894.47 (9,473.43–10,374.02)	9,242.01 (8,821.04–9,738.18)	8,949.06 (8,503.18–9,411.38)	6,258.97 (5,958.11–6,603.30)	5,315.19 (5,014.94–5,670.64)
Ukraine	All causes	36,162.31 (33,142.50–39,390.13)	43,451.74 (40,597.69–46,705.65)	41,684.35 (38,648.40–44,903.96)	41,607.12 (38,774.54–44,759.20)	34,412.12 (31,635.82–37,567.90)	32,828.12 (29,825.64–35,974.82)
Ukraine	Group I	3,457.40 (3,017.11–3,930.47)	3,879.30 (3,464.38–4,337.27)	4,276.59 (3,783.23–4,814.02)	4,166.99 (3,765.87–4,604.02)	3,451.82 (3,120.00–3,813.30)	2,903.32 (2,528.86–3,385.47)
Ukraine	NCD	27,042.18 (24,678.11–29,656.21)	32,071.55 (29,716.05–34,699.09)	30,558.81 (28,157.62–33,184.26)	31,363.98 (28,989.11–33,965.68)	26,939.59 (24,564.42–29,590.46)	25,670.81 (23,202.98–28,432.50)
Ukraine	Injuries	5,662.73 (5,347.74–6,001.20)	7,500.89 (7,144.61–7,879.58)	6,848.94 (6,525.93–7,202.59)	6,076.15 (5,773.91–6,389.63)	4,020.70 (3,783.54–4,293.98)	4,254.00 (3,785.92–4,769.05)
Central Europe	All causes	33,684.25 (30,914.13–36,830.20)	33,233.41 (30,494.28–36,354.08)	30,106.70 (27,403.93–33,186.81)	27,710.39 (25,037.84–30,767.87)	25,517.24 (22,885.54–28,528.36)	23,528.44 (20,884.69–26,501.45)
Central Europe	Group I	3,855.77 (3,579.02–4,175.22)	3,289.29 (3,050.40–3,577.68)	2,747.63 (2,508.22–3,039.45)	2,260.58 (2,035.27–2,545.62)	1,865.77 (1,648.12–2,149.63)	1,602.52 (1,385.98–1,895.17)
Central Europe	NCD	25,920.51 (23,645.65–28,446.74)	25,745.66 (23,441.43–28,304.02)	24,171.71 (21,893.03–26,733.88)	22,702.43 (20,400.08–25,274.88)	21,244.55 (18,938.73–23,775.60)	19,806.84 (17,493.66–22,350.05)
Central Europe	Injuries	3,907.98 (3,656.17–4,204.59)	4,198.46 (3,911.37–4,505.40)	3,187.36 (2,977.00–3,436.89)	2,747.37 (2,569.59–2,969.47)	2,406.92 (2,248.74–2,603.63)	2,119.08 (1,969.63–2,311.48)
Albania	All causes	31,188.70 (28,155.10–34,580.82)	30,587.18 (27,572.74–34,034.03)	28,532.97 (25,645.89–31,795.28)	25,990.35 (23,149.89–29,191.74)	24,053.93 (21,176.01–27,137.51)	22,669.33 (19,813.75–25,895.33)
Albania	Group I	7,413.91 (6,263.92–8,334.07)	6,438.76 (5,667.74–7,256.01)	4,690.55 (4,073.30–5,355.01)	3,498.83 (2,986.17–4,109.05)	2,659.97 (2,128.23–3,229.10)	2,183.08 (1,742.58–2,727.64)
Albania	NCD	20,925.90 (18,604.97–23,430.40)	20,882.90 (18,565.34–23,412.40)	20,893.88 (18,644.10–23,426.10)	20,527.85 (18,243.35–23,237.57)	19,722.97 (17,297.40–22,365.67)	19,003.10 (16,521.27–21,763.63)

Appendix Table 4. Rate of DALYs per 100,000 individuals for all causes and level 1 cause groups by location for 1990 - 2015, both sexes combined.							
	**Cause**	**1990**	**1995**	**2000**	**2005**	**2010**	**2015**
Albania	Injuries	2,848.89 (2,583.11–3,126.25)	3,265.51 (2,948.52–3,559.41)	2,948.55 (2,647.75–3,205.15)	1,963.67 (1,785.90–2,164.65)	1,670.99 (1,506.22–1,867.71)	1,483.15 (1,302.74–1,670.74)
Bosnia and Herzegovina	All causes	30,689.10 (27,964.36–33,763.75)	45,009.34 (40,301.87–50,016.95)	26,094.05 (23,455.20–29,168.50)	23,958.84 (21,222.68–26,956.25)	22,567.10 (19,963.55–25,537.86)	21,369.35 (18,737.08–24,266.50)
Bosnia and Herzegovina	Group I	3,722.28 (3,350.30–4,176.03)	3,323.69 (2,936.48–3,772.80)	2,328.60 (2,038.92–2,669.71)	1,948.78 (1,679.75–2,257.75)	1,698.59 (1,445.42–2,013.96)	1,489.25 (1,217.23–1,801.66)
Bosnia and Herzegovina	NCD	23,236.53 (20,919.71–25,635.25)	23,274.07 (21,020.17–25,739.80)	20,989.51 (18,743.04–23,487.00)	19,689.04 (17,428.40–22,208.01)	18,704.08 (16,485.02–21,193.68)	17,925.54 (15,617.59–20,432.59)
Bosnia and Herzegovina	Injuries	3,730.29 (3,151.70–4,366.48)	18,411.58 (15,295.38–21,507.89)	2,775.93 (2,470.69–3,131.24)	2,321.03 (2,077.23–2,638.54)	2,164.43 (1,933.51–2,458.62)	1,954.55 (1,721.40–2,230.76)
Bulgaria	All causes	32,826.34 (30,110.01–35,870.27)	33,570.57 (30,816.03–36,678.67)	32,487.96 (29,776.18–35,548.16)	30,283.45 (27,578.58–33,310.62)	28,204.51 (25,531.96–31,192.80)	26,500.14 (23,786.92–29,532.65)
Bulgaria	Group I	3,431.14 (3,144.80–3,799.01)	3,197.37 (2,907.15–3,574.05)	3,151.70 (2,860.66–3,514.97)	2,517.02 (2,241.96–2,850.60)	2,271.01 (2,006.77–2,586.11)	1,883.72 (1,568.81–2,265.98)
Bulgaria	NCD	25,830.63 (23,617.64–28,367.34)	26,730.84 (24,530.72–29,306.18)	26,124.42 (23,923.67–28,619.67)	25,067.25 (22,823.65–27,558.25)	23,645.88 (21,391.31–26,129.69)	22,544.15 (20,243.68–25,068.02)
Bulgaria	Injuries	3,564.58 (3,274.64–3,888.58)	3,642.36 (3,368.99–3,959.45)	3,211.84 (2,956.26–3,503.11)	2,699.18 (2,478.04–2,951.25)	2,287.62 (2,096.71–2,526.39)	2,072.27 (1,886.71–2,304.72)
Croatia	All causes	30,550.59 (27,854.47–33,744.67)	30,163.58 (27,260.62–33,250.09)	27,933.50 (25,197.51–30,977.91)	25,335.79 (22,648.99–28,358.30)	23,671.21 (21,042.04–26,752.40)	22,254.10 (19,632.55–25,263.16)
Croatia	Group I	2,493.44 (2,236.52–2,816.14)	2,333.34 (2,068.84–2,657.27)	2,099.12 (1,824.83–2,445.33)	1,724.51 (1,463.85–2,053.14)	1,520.53 (1,269.47–1,836.10)	1,350.42 (1,105.20–1,669.46)
Croatia	NCD	24,133.49 (21,880.90–26,666.88)	23,287.73 (20,966.38–25,842.91)	22,975.30 (20,668.25–25,529.66)	21,194.36 (18,888.73–23,778.96)	20,078.76 (17,809.08–22,671.44)	19,066.91 (16,753.47–21,671.86)
Croatia	Injuries	3,923.66 (3,648.04–4,251.71)	4,542.52 (3,616.61–5,564.76)	2,859.08 (2,665.04–3,088.57)	2,416.91 (2,250.28–2,611.97)	2,071.92 (1,921.51–2,251.88)	1,836.76 (1,694.62–2,012.18)
Czech Republic	All causes	31,761.32 (29,069.20–34,892.76)	28,742.34 (26,063.07–31,851.04)	26,263.47 (23,582.84–29,301.85)	24,270.45 (21,586.97–27,294.90)	22,457.80 (19,818.67–25,459.41)	20,876.32 (18,217.73–23,855.28)
Czech Republic	Group I	2,194.61 (1,956.30–2,502.87)	1,880.07 (1,651.04–2,180.31)	1,672.10 (1,438.79–1,992.31)	1,574.20 (1,329.92–1,890.00)	1,469.28 (1,229.51–1,783.53)	1,313.85 (1,074.89–1,629.43)
Czech Republic	NCD	25,985.89 (23,752.14–28,554.81)	23,621.99 (21,361.87–26,165.98)	21,798.67 (19,559.68–24,307.25)	20,253.06 (17,972.51–22,781.39)	18,899.70 (16,619.03–21,410.35)	17,786.46 (15,483.52–20,321.59)
Czech Republic	Injuries	3,580.81 (3,305.99–3,883.24)	3,240.28 (3,007.09–3,512.24)	2,792.70 (2,586.73–3,029.67)	2,443.18 (2,260.55–2,658.45)	2,088.82 (1,934.40–2,283.31)	1,776.01 (1,626.70–1,957.46)
Hungary	All causes	36,628.28 (33,805.58–39,709.54)	35,294.20 (32,569.52–38,431.57)	32,140.70 (29,388.41–35,206.45)	29,666.96 (26,898.86–32,734.95)	27,016.71 (24,287.46–30,055.94)	24,079.43 (21,410.93–26,991.91)
Hungary	Group I	2,913.06 (2,668.77–3,194.71)	2,374.71 (2,137.77–2,661.20)	2,086.88 (1,834.10–2,367.25)	1,716.23 (1,483.42–1,996.49)	1,476.04 (1,245.67–1,755.04)	1,354.55 (1,090.17–1,664.14)
Hungary	NCD	29,157.05 (26,771.44–31,749.31)	28,965.40 (26,667.67–31,584.34)	26,801.57 (24,449.48–29,407.14)	25,201.01 (22,813.70–27,840.21)	23,322.21 (20,924.82–25,908.71)	20,903.19 (18,547.67–23,448.40)
Hungary	Injuries	4,558.17 (4,294.40–4,845.19)	3,954.10 (3,708.45–4,228.46)	3,252.25 (3,042.74–3,478.18)	2,749.72 (2,572.12–2,949.64)	2,218.46 (2,069.81–2,391.84)	1,821.70 (1,670.39–1,994.15)
Macedonia	All causes	32,492.44 (29,741.98–35,574.56)	31,922.21 (29,323.51–34,892.08)	29,632.74 (27,040.42–32,560.54)	27,606.99 (24,947.62–30,619.98)	25,345.97 (22,753.21–28,253.42)	23,737.81 (20,971.84–26,642.36)
Macedonia	Group I	5,699.92 (5,269.85–6,140.86)	4,245.91 (3,926.87–4,624.36)	2,796.75 (2,534.22–3,078.85)	2,373.03 (2,127.72–2,669.30)	1,897.03 (1,686.64–2,160.58)	1,810.28 (1,409.01–2,333.91)
Macedonia	NCD	24,186.43 (21,863.03–26,741.88)	25,264.23 (22,949.83–27,815.63)	24,699.78 (22,446.80–27,228.23)	23,509.32 (21,175.32–26,114.28)	21,917.05 (19,639.21–24,466.05)	20,349.57 (17,946.24–22,977.55)
Macedonia	Injuries	2,606.09 (2,358.81–2,900.20)	2,412.07 (2,181.84–2,687.84)	2,136.21 (1,953.56–2,351.50)	1,724.64 (1,567.67–1,908.34)	1,531.90 (1,390.09–1,698.81)	1,577.96 (1,420.76–1,742.28)
Montenegro	All causes	27,893.21 (25,078.28–31,159.85)	29,235.99 (26,467.14–32,368.58)	29,862.55 (27,159.26–32,929.33)	26,490.86 (23,842.56–29,460.40)	24,053.40 (21,419.81–27,089.45)	23,058.97 (20,334.49–26,101.43)
Montenegro	Group I	3,438.24 (2,712.46–4,267.24)	3,077.49 (2,721.29–3,499.87)	3,550.32 (3,171.99–3,958.96)	2,234.01 (1,953.60–2,586.56)	1,741.47 (1,478.81–2,061.38)	1,495.57 (1,207.90–1,831.48)

Appendix Table 4. Rate of DALYs per 100,000 individuals for all causes and level 1 cause groups by location for 1990 - 2015, both sexes combined.

	Cause	1990	1995	2000	2005	2010	2015
Montenegro	NCD	21,314.07 (19,069.00–23,862.47)	22,856.68 (20,493.85–25,363.00)	23,219.41 (21,016.36–25,764.79)	21,642.99 (19,380.58–24,139.78)	19,937.95 (17,684.46–22,477.07)	19,427.09 (17,039.41–22,046.62)
Montenegro	Injuries	3,140.90 (2,832.64–3,498.86)	3,301.82 (3,051.71–3,603.13)	3,092.82 (2,859.21–3,371.64)	2,613.86 (2,405.23–2,859.76)	2,373.99 (2,170.35–2,595.29)	2,136.31 (1,910.81–2,386.35)
Poland	All causes	33,642.92 (30,915.73–36,747.84)	31,699.99 (28,974.40–34,762.94)	28,518.73 (25,846.87–31,565.36)	26,386.06 (23,753.78–29,434.55)	24,603.41 (22,001.54–27,576.98)	22,758.31 (20,157.81–25,717.88)
Poland	Group I	3,063.21 (2,833.63–3,364.73)	2,589.58 (2,372.67–2,862.10)	1,924.21 (1,730.64–2,179.78)	1,700.98 (1,513.29–1,953.94)	1,542.28 (1,352.34–1,801.86)	1,321.01 (1,099.33–1,587.78)
Poland	NCD	26,636.00 (24,364.48–29,273.19)	25,416.30 (23,097.64–28,017.70)	23,303.01 (20,999.52–25,912.45)	21,707.99 (19,411.68–24,340.78)	20,400.93 (18,104.41–22,950.76)	19,089.47 (16,813.37–21,668.99)
Poland	Injuries	3,943.72 (3,683.61–4,226.30)	3,694.11 (3,457.26–3,956.90)	3,291.52 (3,078.59–3,534.92)	2,977.09 (2,790.82–3,194.53)	2,660.20 (2,499.73–2,849.37)	2,347.84 (2,182.06–2,543.43)
Romania	All causes	35,986.40 (33,198.03–39,147.95)	36,847.24 (34,067.73–39,960.08)	33,756.72 (31,007.56–36,872.71)	30,912.66 (28,217.54–33,951.20)	28,423.63 (25,740.62–31,440.48)	26,048.34 (23,311.14–29,190.25)
Romania	Group I	6,053.74 (5,351.08–6,476.71)	5,204.28 (4,773.72–5,595.86)	4,904.06 (4,457.56–5,290.82)	3,877.30 (3,530.05–4,252.65)	2,811.68 (2,521.03–3,176.78)	2,311.47 (1,959.73–2,742.48)
Romania	NCD	25,680.57 (23,418.01–28,133.09)	27,447.25 (25,176.33–29,934.50)	25,380.64 (23,123.61–27,846.82)	24,019.06 (21,775.33–26,517.67)	22,876.62 (20,588.28–25,339.00)	21,375.76 (19,034.29–23,902.92)
Romania	Injuries	4,252.09 (3,942.44–4,585.16)	4,195.71 (3,915.34–4,508.79)	3,472.02 (3,226.89–3,750.12)	3,016.30 (2,795.22–3,257.09)	2,735.33 (2,546.58–2,955.16)	2,361.10 (2,167.47–2,592.68)
Serbia	All causes	30,916.91 (28,102.51–34,102.82)	31,648.39 (28,939.48–34,578.33)	31,167.50 (28,409.78–34,264.68)	28,809.51 (26,131.22–31,855.27)	26,181.05 (23,529.58–29,169.51)	24,495.78 (21,838.34–27,500.27)
Serbia	Group I	3,819.52 (3,275.47–4,424.83)	3,182.77 (2,887.97–3,521.25)	2,332.37 (2,072.81–2,641.73)	1,974.14 (1,737.11–2,269.33)	1,802.79 (1,577.50–2,073.50)	1,662.32 (1,432.62–1,954.42)
Serbia	NCD	23,862.81 (21,451.41–26,432.70)	25,537.73 (23,180.56–28,071.09)	25,785.51 (23,428.11–28,367.89)	24,449.29 (22,067.99–27,040.73)	22,303.06 (19,968.95–24,911.82)	20,758.63 (18,423.36–23,325.12)
Serbia	Injuries	3,234.57 (2,913.93–3,537.60)	2,927.89 (2,692.75–3,168.50)	3,049.62 (2,754.31–3,361.13)	2,386.08 (2,215.63–2,576.58)	2,075.20 (1,927.42–2,266.37)	2,074.84 (1,921.05–2,264.15)
Slovakia	All causes	33,389.88 (30,644.20–36,499.99)	30,622.20 (27,822.89–33,756.51)	29,291.61 (26,593.01–32,308.74)	27,431.29 (24,726.59–30,515.86)	25,229.00 (22,563.32–28,259.86)	22,747.31 (20,084.43–25,704.31)
Slovakia	Group I	3,112.81 (2,802.93–3,466.86)	2,696.32 (2,413.38–3,024.25)	2,310.31 (2,053.73–2,613.36)	2,102.25 (1,855.77–2,420.25)	1,867.11 (1,621.77–2,183.42)	1,660.81 (1,398.94–1,976.74)
Slovakia	NCD	26,337.70 (24,048.15–28,832.30)	24,578.93 (22,210.63–27,135.02)	23,996.54 (21,718.76–26,539.79)	22,730.06 (20,418.27–25,317.79)	21,065.08 (18,769.56–23,605.16)	19,103.44 (16,803.72–21,623.96)
Slovakia	Injuries	3,939.38 (3,621.60–4,254.88)	3,346.95 (3,090.10–3,635.79)	2,984.77 (2,765.48–3,234.40)	2,598.97 (2,402.00–2,831.53)	2,296.80 (2,113.60–2,517.30)	1,983.06 (1,801.67–2,209.51)
Slovenia	All causes	28,762.38 (25,995.54–31,857.03)	27,102.54 (24,361.02–30,268.16)	25,160.64 (22,446.49–28,213.10)	22,817.71 (20,129.12–25,849.67)	20,279.00 (17,599.93–23,253.78)	18,733.69 (16,083.17–21,703.63)
Slovenia	Group I	2,106.42 (1,827.84–2,446.05)	1,853.17 (1,585.73–2,196.69)	1,692.39 (1,429.89–2,007.09)	1,528.32 (1,264.30–1,864.93)	1,355.06 (1,099.22–1,691.68)	1,234.97 (990.34–1,564.77)
Slovenia	NCD	22,377.54 (20,095.83–24,857.91)	21,407.18 (19,097.59–23,956.79)	20,216.01 (17,895.92–22,733.25)	18,559.99 (16,301.48–21,092.95)	16,836.76 (14,540.17–19,362.04)	15,770.48 (13,477.56–18,299.15)
Slovenia	Injuries	4,278.42 (3,983.52–4,590.11)	3,842.19 (3,593.66–4,115.99)	3,252.24 (3,034.04–3,492.67)	2,729.40 (2,548.60–2,932.47)	2,087.18 (1,930.98–2,270.60)	1,728.24 (1,577.94–1,914.99)
Central Asia	All causes	42,844.52 (39,968.06–46,196.57)	46,498.66 (43,551.71–49,666.30)	43,628.55 (40,740.75–46,700.11)	41,103.54 (38,297.00–44,213.56)	36,689.94 (33,939.67–39,841.28)	33,419.80 (30,598.60–36,491.89)
Central Asia	Group I	11,027.47 (10,470.20–11,665.47)	11,472.03 (10,896.84–12,154.84)	9,965.06 (9,399.71–10,571.96)	8,260.08 (7,663.08–8,916.62)	6,619.25 (6,102.95–7,190.77)	5,408.42 (4,890.81–5,929.98)
Central Asia	NCD	26,990.97 (24,703.16–29,492.23)	29,518.60 (27,199.78–31,991.20)	29,060.17 (26,801.88–31,568.13)	28,631.30 (26,393.08–31,167.38)	26,395.87 (24,147.64–28,930.07)	24,662.83 (22,444.62–27,178.14)
Central Asia	Injuries	4,826.08 (4,556.82–5,115.89)	5,508.03 (5,114.29–5,891.18)	4,603.32 (4,361.10–4,860.63)	4,212.15 (3,992.15–4,463.85)	3,674.83 (3,461.03–3,922.96)	3,348.54 (3,114.88–3,603.37)
Armenia	All causes	36,882.82 (33,988.20–40,064.88)	38,308.17 (35,418.91–41,506.27)	33,945.48 (31,129.78–37,170.24)	32,574.17 (29,779.51–35,682.35)	30,254.96 (27,445.04–33,459.95)	27,392.44 (24,587.74–30,471.43)

Appendix Table 4. Rate of DALYs per 100,000 individuals for all causes and level 1 cause groups by location for 1990 - 2015, both sexes combined.

	Cause	1990	1995	2000	2005	2010	2015
Armenia	Group I	7,581.15 (6,947.61–8,241.11)	7,251.90 (6,565.37–7,989.99)	5,286.14 (4,711.71–5,920.75)	4,402.95 (3,849.01–4,991.43)	3,557.56 (2,962.21–4,131.73)	2,868.19 (2,342.30–3,439.04)
Armenia	NCD	24,087.62 (21,877.08–26,465.31)	26,167.69 (23,829.42–28,601.04)	25,058.13 (22,727.30–27,602.68)	24,887.64 (22,658.75–27,349.76)	23,620.40 (21,343.34–26,175.87)	21,898.26 (19,700.13–24,457.86)
Armenia	Injuries	5,214.05 (4,655.50–5,761.02)	4,888.58 (4,432.02–5,359.15)	3,601.21 (3,231.69–4,005.39)	3,283.58 (2,941.12–3,627.12)	3,077.00 (2,770.97–3,386.35)	2,625.99 (2,335.50–2,930.00)
Azerbaijan	All causes	46,868.99 (43,783.21–50,399.35)	48,114.72 (44,599.19–51,621.31)	43,753.72 (40,565.45–47,211.00)	39,665.04 (36,399.34–43,099.71)	35,602.63 (32,281.06–39,193.10)	30,806.60 (27,619.15–34,308.00)
Azerbaijan	Group I	14,087.78 (12,815.76–15,501.57)	14,334.93 (13,056.04–15,789.14)	12,102.15 (11,053.36–13,299.78)	9,672.40 (8,702.29–10,704.62)	7,245.95 (6,085.29–8,535.60)	5,607.42 (4,664.26–6,621.76)
Azerbaijan	NCD	28,365.69 (26,051.82–30,884.34)	29,041.48 (26,759.41–31,628.89)	28,369.67 (25,952.82–31,036.76)	27,139.84 (24,633.77–29,848.50)	25,880.06 (23,521.31–28,625.27)	22,839.72 (20,426.29–25,561.53)
Azerbaijan	Injuries	4,415.52 (4,052.87–4,772.99)	4,738.30 (4,323.76–5,125.86)	3,281.91 (2,985.43–3,615.95)	2,852.80 (2,584.00–3,143.39)	2,476.62 (2,210.24–2,767.63)	2,359.46 (2,081.81–2,676.64)
Georgia	All causes	36,172.21 (33,235.11–39,343.45)	34,606.89 (31,716.90–37,807.86)	33,204.05 (30,319.12–36,328.21)	32,181.92 (29,259.29–35,404.93)	31,229.16 (28,319.59–34,389.03)	30,310.51 (27,293.51–33,466.84)
Georgia	Group I	7,716.75 (7,069.18–8,395.84)	7,560.63 (6,938.13–8,230.61)	6,823.33 (6,160.48–7,572.53)	5,382.14 (4,725.59–6,145.44)	4,389.58 (3,781.16–5,091.71)	3,685.75 (3,131.01–4,341.98)
Georgia	NCD	24,490.39 (22,095.97–27,042.09)	23,638.59 (21,270.20–26,329.07)	23,625.78 (21,365.47–26,122.80)	24,044.89 (21,758.33–26,583.34)	23,857.11 (21,535.87–26,431.13)	23,468.41 (21,094.40–26,104.70)
Georgia	Injuries	3,965.08 (3,631.84–4,329.23)	3,407.66 (3,086.19–3,724.54)	2,754.93 (2,472.93–3,107.29)	2,754.90 (2,501.58–3,031.78)	2,982.46 (2,743.97–3,257.75)	3,156.35 (2,874.86–3,464.09)
Kazakhstan	All causes	40,865.50 (37,949.88–44,290.41)	50,500.04 (47,199.82–54,054.66)	47,308.63 (44,290.60–50,761.71)	46,256.38 (43,164.43–49,594.72)	38,923.67 (35,904.21–42,249.10)	35,613.20 (32,122.76–39,255.25)
Kazakhstan	Group I	7,765.11 (7,067.48–8,576.79)	8,821.26 (8,013.43–9,643.70)	7,735.79 (7,005.72–8,559.61)	6,636.18 (5,925.76–7,469.63)	5,165.86 (4,515.70–5,916.64)	4,319.25 (3,668.88–5,036.44)
Kazakhstan	NCD	27,355.50 (25,136.24–29,898.88)	32,846.58 (30,545.70–35,407.38)	32,376.38 (30,131.21–34,950.41)	32,603.14 (30,333.03–35,104.98)	28,410.97 (26,190.19–30,940.88)	26,424.36 (23,736.72–29,272.98)
Kazakhstan	Injuries	5,744.89 (5,467.34–6,045.41)	8,832.19 (7,673.19–9,990.67)	7,196.45 (6,864.37–7,526.62)	7,017.07 (6,724.19–7,335.25)	5,346.85 (5,091.34–5,644.95)	4,869.59 (4,490.29–5,281.53)
Kyrgyzstan	All causes	45,563.53 (42,617.93–48,958.38)	47,080.99 (43,908.48–50,317.87)	44,886.30 (41,796.39–48,080.30)	43,020.07 (39,896.18–46,212.12)	39,980.69 (37,095.60–43,381.41)	36,034.20 (33,007.83–39,402.93)
Kyrgyzstan	Group I	11,916.53 (11,287.28–12,654.30)	10,738.40 (10,137.95–11,389.24)	9,865.01 (9,226.12–10,509.09)	8,449.11 (7,840.63–9,066.36)	7,232.12 (6,671.32–7,842.63)	6,114.46 (5,506.64–6,829.92)
Kyrgyzstan	NCD	27,944.99 (25,612.86–30,412.52)	30,858.74 (28,292.46–33,378.99)	30,327.03 (27,940.00–32,862.07)	30,449.28 (27,921.44–33,083.49)	28,582.54 (26,234.84–31,153.10)	26,567.42 (24,150.84–29,352.26)
Kyrgyzstan	Injuries	5,702.01 (5,253.88–6,097.27)	5,483.84 (5,060.09–5,850.22)	4,694.25 (4,377.98–5,001.03)	4,121.68 (3,854.05–4,435.03)	4,166.03 (3,923.57–4,458.91)	3,352.31 (3,090.47–3,668.70)
Mongolia	All causes	53,277.19 (50,143.27–56,727.19)	54,495.30 (51,386.27–57,906.80)	52,553.00 (49,418.36–55,928.77)	49,529.98 (46,173.73–52,936.73)	45,600.34 (42,481.09–48,945.95)	41,354.14 (38,136.48–44,899.45)
Mongolia	Group I	15,586.55 (14,383.62–16,771.37)	13,361.75 (12,299.71–14,399.97)	10,805.51 (9,966.95–11,694.94)	8,348.29 (7,558.14–9,232.06)	6,582.43 (5,758.53–7,464.46)	5,099.49 (4,393.58–6,045.09)
Mongolia	NCD	32,254.53 (29,573.56–34,920.31)	35,659.19 (33,108.72–38,252.41)	36,224.34 (33,686.17–38,964.16)	35,203.03 (32,566.51–37,900.48)	33,074.23 (30,576.07–35,675.41)	30,787.75 (28,114.25–33,473.42)
Mongolia	Injuries	5,436.11 (4,930.24–5,916.90)	5,474.37 (4,963.23–5,951.60)	5,523.16 (5,020.50–5,970.92)	5,978.66 (5,290.64–6,495.90)	5,943.68 (5,129.18–6,439.96)	5,466.90 (4,768.74–5,983.86)
Tajikistan	All causes	47,413.34 (44,096.79–50,996.47)	49,902.88 (46,427.64–53,672.23)	45,861.12 (42,637.95–49,252.83)	40,332.97 (37,335.01–43,634.00)	35,775.35 (32,879.03–39,112.65)	32,010.22 (28,862.12–35,393.94)
Tajikistan	Group I	16,758.50 (15,497.10–18,099.74)	17,095.80 (15,745.11–18,536.99)	14,232.74 (13,171.21–15,388.02)	10,653.85 (9,768.86–11,633.43)	8,581.00 (7,775.42–9,415.65)	7,101.23 (6,116.62–8,175.42)
Tajikistan	NCD	26,305.98 (23,924.31–28,926.52)	27,713.54 (25,336.87–30,339.19)	27,825.82 (25,464.55–30,361.97)	26,604.60 (24,328.85–29,219.81)	24,379.38 (22,087.44–26,989.99)	22,365.74 (20,014.04–24,842.98)
Tajikistan	Injuries	4,348.86 (4,003.47–4,714.57)	5,093.54 (4,633.40–5,548.85)	3,802.56 (3,504.30–4,137.98)	3,074.52 (2,824.41–3,359.07)	2,814.97 (2,554.81–3,098.42)	2,543.25 (2,284.70–2,850.95)

Appendix Table 4. Rate of DALYs per 100,000 individuals for all causes and level 1 cause groups by location for 1990 - 2015, both sexes combined.

	Cause	1990	1995	2000	2005	2010	2015
Turkmenistan	All causes	49,833.15 (46,314.54–53,750.29)	50,884.35 (47,290.65–54,728.91)	49,725.50 (45,840.51–54,069.95)	44,905.30 (41,188.69–48,948.30)	39,807.79 (36,224.90–43,642.27)	36,053.94 (32,841.62–39,695.55)
Turkmenistan	Group I	16,252.64 (14,385.58–18,225.17)	15,546.91 (13,613.93–17,669.14)	14,341.99 (12,099.81–17,083.41)	11,552.70 (9,349.46–13,991.98)	8,954.65 (7,186.18–10,971.37)	7,137.61 (5,758.60–8,848.14)
Turkmenistan	NCD	28,856.44 (26,596.64–31,287.99)	30,772.23 (28,550.53–33,170.07)	30,888.22 (28,638.88–33,351.47)	29,388.56 (27,107.98–32,024.46)	27,573.52 (25,273.97–30,356.16)	26,022.63 (23,619.82–28,713.97)
Turkmenistan	Injuries	4,724.07 (4,306.62–5,150.00)	4,565.21 (4,186.57–5,015.52)	4,495.28 (4,065.71–4,935.75)	3,964.04 (3,571.52–4,374.60)	3,279.62 (2,978.84–3,670.53)	2,893.70 (2,601.09–3,310.69)
Uzbekistan	All causes	41,201.32 (37,969.75–44,863.30)	43,500.19 (40,272.64–47,055.76)	41,287.31 (38,003.21–44,550.68)	38,986.41 (35,704.79–42,559.56)	35,367.47 (32,191.03–38,800.20)	32,532.35 (29,448.34–36,069.50)
Uzbekistan	Group I	9,857.50 (8,962.92–10,810.46)	10,424.09 (9,575.38–11,412.03)	9,002.56 (8,124.88–10,012.56)	7,954.16 (6,927.38–9,224.22)	6,630.31 (5,689.57–7,773.11)	5,377.79 (4,540.77–6,336.63)
Uzbekistan	NCD	27,130.61 (24,782.26–29,864.78)	29,000.32 (26,570.27–31,571.40)	28,425.24 (26,087.03–30,954.74)	27,647.34 (25,248.68–30,279.87)	25,572.48 (23,187.85–28,225.31)	24,211.47 (21,874.16–26,892.78)
Uzbekistan	Injuries	4,213.21 (3,869.01–4,573.58)	4,075.78 (3,769.01–4,390.09)	3,859.52 (3,547.56–4,166.92)	3,384.90 (3,104.65–3,698.17)	3,164.69 (2,874.55–3,494.38)	2,943.09 (2,635.43–3,296.47)
Latin America and Caribbean	All causes	38,727.53 (35,846.93–42,021.86)	36,239.15 (33,393.81–39,422.11)	33,244.69 (30,381.75–36,419.09)	30,872.15 (28,052.66–34,053.61)	31,780.58 (28,416.85–35,474.01)	27,917.02 (25,100.90–30,978.22)
Latin America and Caribbean	Group I	10,263.07 (9,829.88–10,772.75)	8,550.86 (8,187.31–9,002.11)	6,988.17 (6,632.84–7,419.48)	5,935.52 (5,580.92–6,379.31)	5,093.34 (4,728.29–5,543.17)	4,396.99 (4,034.09–4,850.92)
Latin America and Caribbean	NCD	23,381.48 (20,912.19–25,979.64)	22,905.42 (20,492.05–25,510.15)	21,915.29 (19,525.99–24,566.41)	20,977.32 (18,586.00–23,596.44)	20,333.02 (17,912.34–22,948.17)	19,980.76 (17,595.70–22,593.48)
Latin America and Caribbean	Injuries	5,082.98 (4,897.47–5,280.58)	4,782.86 (4,629.79–4,942.25)	4,341.23 (4,206.25–4,494.23)	3,959.30 (3,830.65–4,101.21)	6,354.21 (4,289.99–8,551.11)	3,539.27 (3,402.68–3,680.86)
Central Latin America	All causes	35,014.04 (32,362.29–38,113.42)	32,729.35 (30,030.86–35,744.21)	30,222.31 (27,535.48–33,271.15)	28,505.42 (25,869.82–31,542.93)	27,505.13 (24,879.89–30,495.69)	26,352.65 (23,689.94–29,361.04)
Central Latin America	Group I	8,525.77 (8,200.64–8,954.61)	6,951.07 (6,616.73–7,367.00)	5,721.61 (5,387.56–6,135.44)	4,889.38 (4,563.15–5,307.33)	4,209.31 (3,882.09–4,629.22)	3,650.33 (3,327.28–4,059.66)
Central Latin America	NCD	21,436.07 (19,102.52–23,980.07)	21,176.98 (18,876.34–23,744.47)	20,333.05 (18,048.54–22,912.72)	19,877.57 (17,601.63–22,463.23)	19,561.91 (17,276.37–22,166.45)	19,339.27 (17,014.01–21,927.92)
Central Latin America	Injuries	5,052.20 (4,928.25–5,194.85)	4,601.30 (4,507.83–4,709.66)	4,167.65 (4,070.24–4,272.09)	3,738.48 (3,648.41–3,838.63)	3,733.91 (3,647.00–3,835.82)	3,363.05 (3,242.90–3,488.95)
Colombia	All causes	34,043.90 (31,432.62–37,149.38)	33,047.27 (30,356.60–36,074.43)	30,742.76 (28,076.51–33,786.85)	27,699.01 (25,073.66–30,794.75)	25,867.81 (23,142.21–28,878.45)	23,743.62 (20,942.63–26,750.64)
Colombia	Group I	5,934.93 (5,549.98–6,381.43)	5,322.84 (4,922.81–5,774.05)	4,562.55 (4,197.09–5,001.52)	4,127.44 (3,761.67–4,596.20)	3,671.47 (3,202.66–4,218.12)	3,060.15 (2,572.47–3,647.59)
Colombia	NCD	21,162.91 (18,844.17–23,781.90)	20,737.20 (18,433.68–23,359.55)	19,777.51 (17,468.14–22,419.28)	18,854.76 (16,554.52–21,562.27)	18,118.60 (15,764.70–20,742.48)	17,199.21 (14,876.02–19,855.79)
Colombia	Injuries	6,946.06 (6,762.21–7,141.84)	6,987.23 (6,822.32–7,165.55)	6,402.70 (6,231.02–6,575.57)	4,716.82 (4,590.69–4,851.27)	4,077.74 (3,956.18–4,207.33)	3,484.25 (3,296.81–3,666.82)
Costa Rica	All causes	24,919.28 (22,286.94–27,900.27)	24,715.23 (22,191.37–27,730.10)	23,543.40 (20,905.45–26,614.74)	22,103.48 (19,460.80–25,119.86)	21,149.17 (18,457.99–24,123.97)	20,491.36 (17,791.69–23,522.02)
Costa Rica	Group I	3,810.50 (3,364.93–4,361.75)	3,534.49 (3,089.72–4,070.44)	3,020.20 (2,612.17–3,567.70)	2,628.26 (2,253.04–3,103.63)	2,332.74 (1,958.34–2,789.86)	2,103.51 (1,733.46–2,588.40)
Costa Rica	NCD	18,600.11 (16,356.93–21,184.77)	18,597.20 (16,399.28–21,200.34)	18,004.70 (15,757.69–20,619.68)	17,156.19 (14,915.32–19,780.05)	16,574.62 (14,293.60–19,113.29)	16,336.06 (13,966.88–19,009.28)
Costa Rica	Injuries	2,508.67 (2,401.60–2,628.14)	2,583.55 (2,482.30–2,705.43)	2,518.49 (2,412.54–2,629.12)	2,319.03 (2,222.28–2,418.66)	2,241.80 (2,148.52–2,338.67)	2,051.79 (1,922.12–2,189.98)
El Salvador	All causes	42,403.01 (39,216.49–45,854.01)	37,625.26 (34,493.21–40,885.76)	34,134.55 (31,098.81–37,259.10)	32,119.76 (29,212.75–35,590.08)	30,311.39 (27,350.10–33,765.35)	28,842.99 (25,621.06–32,280.40)
El Salvador	Group I	10,006.01 (9,189.15–10,914.07)	8,119.65 (7,360.97–8,897.67)	6,711.92 (6,031.36–7,423.02)	5,156.65 (4,552.88–5,902.48)	4,232.97 (3,588.92–5,027.58)	3,799.27 (3,205.58–4,535.51)
El Salvador	NCD	23,661.41 (21,168.46–26,294.64)	22,208.05 (19,724.47–24,820.99)	20,721.80 (18,336.07–23,345.54)	20,323.76 (17,987.36–23,048.44)	19,929.04 (17,542.85–22,684.41)	19,396.12 (16,916.46–22,222.78)

Appendix Table 4. Rate of DALYs per 100,000 individuals for all causes and level 1 cause groups by location for 1990 - 2015, both sexes combined.

	Cause	1990	1995	2000	2005	2010	2015
El Salvador	Injuries	8,735.60 (7,925.23–9,448.29)	7,297.57 (6,721.18–7,699.19)	6,700.83 (6,329.44–7,079.84)	6,639.35 (6,241.59–6,988.14)	6,149.38 (5,691.45–6,546.14)	5,647.60 (5,095.51–6,213.97)
Guatemala	All causes	49,513.35 (46,347.60–52,964.69)	44,304.48 (41,175.43–47,546.67)	40,499.23 (37,355.04–43,891.39)	38,376.41 (35,219.35–41,984.61)	35,458.40 (32,169.40–39,021.98)	32,906.61 (29,167.62–36,993.72)
Guatemala	Group I	21,762.49 (20,722.21–22,905.59)	16,753.78 (15,957.26–17,738.33)	14,083.30 (13,271.21–15,018.35)	11,607.02 (10,701.93–12,535.24)	9,160.53 (8,278.38–10,148.70)	7,402.74 (6,520.75–8,345.62)
Guatemala	NCD	22,230.45 (19,798.45–24,835.15)	22,222.85 (19,796.40–24,794.76)	21,311.41 (18,969.30–23,967.35)	21,159.90 (18,738.80–23,862.05)	21,111.59 (18,732.41–23,787.75)	20,707.50 (17,877.49–23,720.80)
Guatemala	Injuries	5,520.41 (5,234.66–5,812.01)	5,327.84 (5,140.60–5,512.78)	5,104.52 (4,934.41–5,281.79)	5,609.49 (5,421.60–5,813.51)	5,186.29 (5,023.12–5,353.77)	4,796.37 (4,227.37–5,395.57)
Honduras	All causes	38,040.17 (34,844.45–41,493.42)	37,013.56 (33,399.99–41,103.96)	35,885.26 (31,913.51–40,582.45)	34,099.61 (29,811.62–39,029.03)	32,466.35 (28,260.90–37,224.50)	30,728.35 (26,593.67–35,422.04)
Honduras	Group I	10,156.90 (9,415.27–10,946.30)	8,535.94 (7,869.52–9,338.29)	7,540.65 (6,861.51–8,323.58)	6,427.52 (5,784.24–7,168.56)	5,575.64 (4,970.18–6,232.34)	4,777.93 (4,186.45–5,403.17)
Honduras	NCD	24,040.34 (21,518.72–26,804.63)	24,630.48 (21,937.48–27,724.98)	24,141.74 (21,000.31–27,632.90)	23,414.68 (20,112.94–26,994.83)	22,499.39 (19,275.65–25,904.77)	22,066.95 (18,938.22–25,435.16)
Honduras	Injuries	3,842.93 (3,511.46–4,162.62)	3,847.14 (3,255.75–4,405.45)	4,202.87 (3,235.46–5,075.19)	4,257.41 (3,085.30–5,259.95)	4,391.33 (3,087.08–5,552.83)	3,883.46 (2,773.84–5,027.78)
Mexico	All causes	34,349.05 (31,763.19–37,319.15)	31,347.91 (28,782.82–34,259.46)	28,563.07 (25,958.68–31,433.34)	27,328.19 (24,749.32–30,121.95)	26,845.47 (24,230.07–29,751.23)	25,927.14 (23,324.55–28,788.99)
Mexico	Group I	8,380.78 (8,079.42–8,710.76)	6,350.17 (6,076.10–6,663.87)	4,900.10 (4,664.65–5,190.84)	4,077.61 (3,845.97–4,354.65)	3,476.05 (3,251.42–3,747.73)	2,993.72 (2,768.10–3,268.91)
Mexico	NCD	21,453.92 (19,157.90–24,030.77)	21,263.35 (18,968.33–23,851.39)	20,564.12 (18,276.43–23,113.96)	20,425.28 (18,146.09–22,967.65)	20,294.33 (17,981.66–22,886.82)	20,219.36 (17,912.00–22,797.52)
Mexico	Injuries	4,514.35 (4,389.58–4,669.93)	3,734.40 (3,654.63–3,848.35)	3,098.85 (3,015.18–3,205.49)	2,825.30 (2,750.54–2,915.20)	3,075.08 (3,004.34–3,161.55)	2,714.05 (2,645.93–2,793.70)
Nicaragua	All causes	33,814.47 (30,945.46–37,192.00)	31,165.23 (28,223.12–34,335.95)	28,145.31 (25,314.49–31,333.08)	26,510.62 (23,722.22–29,703.81)	25,082.88 (22,456.67–28,104.85)	24,001.80 (21,059.00–27,255.92)
Nicaragua	Group I	11,411.66 (10,680.49–12,142.31)	9,112.77 (8,550.72–9,753.81)	6,827.56 (6,326.27–7,432.27)	5,289.26 (4,799.41–5,825.40)	4,204.22 (3,707.85–4,749.90)	3,479.82 (2,987.54–4,024.56)
Nicaragua	NCD	18,515.82 (16,084.87–21,101.25)	18,562.10 (16,100.57–21,235.61)	18,226.82 (15,880.45–20,887.97)	18,414.66 (15,975.25–21,149.38)	18,369.28 (16,030.06–21,034.41)	18,172.70 (15,540.87–20,893.97)
Nicaragua	Injuries	3,886.99 (3,634.70–4,159.06)	3,490.37 (3,218.49–3,740.84)	3,090.93 (2,881.10–3,304.66)	2,806.70 (2,612.82–2,991.59)	2,509.38 (2,349.59–2,692.91)	2,349.28 (2,112.02–2,611.64)
Panama	All causes	28,230.19 (25,320.75–31,366.06)	27,704.94 (24,768.76–31,016.88)	26,236.29 (23,378.82–29,484.56)	25,442.73 (22,630.34–28,555.20)	25,385.82 (22,539.83–28,589.69)	24,295.40 (21,162.21–27,745.50)
Panama	Group I	5,420.93 (4,791.89–6,204.61)	5,154.93 (4,524.88–5,976.46)	4,906.25 (4,276.61–5,639.93)	4,924.04 (4,329.04–5,608.54)	4,631.23 (4,066.38–5,300.44)	4,126.13 (3,490.84–4,889.98)
Panama	NCD	19,441.22 (17,010.83–21,999.97)	19,114.52 (16,651.78–21,914.22)	18,193.99 (15,759.18–20,948.14)	17,694.16 (15,270.98–20,364.25)	17,575.97 (15,153.04–20,338.60)	17,286.74 (14,707.55–20,199.16)
Panama	Injuries	3,368.04 (3,115.26–3,615.89)	3,435.49 (3,180.07–3,681.00)	3,136.04 (2,905.62–3,370.65)	2,824.53 (2,591.02–3,050.77)	3,178.63 (2,914.58–3,433.97)	2,882.53 (2,501.31–3,285.40)
Venezuela	All causes	31,915.27 (29,115.29–35,052.05)	32,353.15 (29,307.12–35,662.95)	31,146.48 (28,152.94–34,577.24)	30,004.89 (26,986.71–33,436.65)	29,663.38 (26,639.80–33,129.75)	29,704.82 (26,000.73–33,616.19)
Venezuela	Group I	6,452.11 (5,974.54–7,062.71)	6,707.42 (6,084.78–7,453.50)	6,107.59 (5,380.23–7,036.50)	5,540.87 (4,844.67–6,522.64)	5,163.66 (4,477.58–6,114.75)	4,982.85 (4,241.13–5,897.88)
Venezuela	NCD	21,562.81 (19,275.77–24,039.67)	21,466.46 (19,121.35–23,984.22)	20,411.68 (18,163.95–22,927.56)	19,530.40 (17,232.26–22,052.69)	19,255.76 (16,959.62–21,769.98)	19,565.94 (16,883.66–22,371.90)
Venezuela	Injuries	3,900.34 (3,758.85–4,047.53)	4,179.27 (4,020.31–4,331.13)	4,627.21 (4,486.61–4,765.49)	4,933.62 (4,799.95–5,071.91)	5,243.96 (5,105.91–5,382.99)	5,156.03 (4,548.44–5,799.85)
Andean Latin America	All causes	42,469.17 (39,374.14–45,904.99)	37,768.20 (34,801.97–40,991.92)	32,612.88 (29,746.62–35,712.98)	28,967.26 (26,149.09–32,109.43)	26,787.48 (24,001.47–29,892.84)	25,032.27 (22,096.69–28,288.70)
Andean Latin America	Group I	14,903.65 (14,088.17–15,723.36)	11,910.82 (11,337.40–12,552.09)	9,006.97 (8,512.78–9,603.16)	7,197.31 (6,725.23–7,759.48)	5,871.57 (5,398.98–6,427.80)	4,920.53 (4,425.24–5,478.09)

Appendix Table 4. Rate of DALYs per 100,000 individuals for all causes and level 1 cause groups by location for 1990 - 2015, both sexes combined.							
	**Cause**	**1990**	**1995**	**2000**	**2005**	**2010**	**2015**
Andean Latin America	NCD	22,340.65 (19,909.69–24,955.12)	21,288.29 (18,909.05–23,933.20)	19,736.65 (17,452.36–22,276.02)	18,467.68 (16,142.59–21,023.32)	17,817.64 (15,492.21–20,364.48)	17,339.68 (14,870.89–19,976.55)
Andean Latin America	Injuries	5,224.87 (4,728.65–5,655.15)	4,569.10 (4,255.76–4,871.53)	3,869.25 (3,627.79–4,100.42)	3,302.28 (3,083.61–3,510.99)	3,098.27 (2,880.43–3,318.63)	2,772.05 (2,525.17–3,054.54)
Bolivia	All causes	55,789.68 (52,099.23–59,719.16)	49,079.11 (45,595.38–52,734.31)	42,285.93 (38,879.75–45,875.49)	36,983.77 (33,572.20–40,452.45)	33,801.49 (30,012.59–37,863.46)	31,467.35 (27,545.01–35,952.12)
Bolivia	Group I	21,136.10 (19,802.46–22,414.87)	17,133.93 (16,065.13–18,240.15)	13,341.63 (12,515.48–14,114.66)	10,451.95 (9,636.37–11,290.25)	8,487.96 (7,615.26–9,450.46)	6,986.09 (6,077.97–7,948.01)
Bolivia	NCD	26,511.79 (23,656.04–29,642.12)	25,136.35 (22,434.35–27,888.58)	23,298.43 (20,754.78–26,002.11)	21,754.47 (19,147.03–24,364.00)	21,041.01 (18,170.12–23,970.05)	20,646.14 (17,699.81–23,782.51)
Bolivia	Injuries	8,141.79 (6,971.55–9,256.06)	6,808.82 (6,051.66–7,539.30)	5,645.86 (5,233.78–6,117.60)	4,777.35 (4,346.09–5,262.63)	4,272.52 (3,772.94–4,879.37)	3,835.12 (3,288.84–4,575.68)
Ecuador	All causes	35,922.09 (32,963.90–39,189.09)	33,743.01 (30,896.46–36,927.96)	31,858.35 (28,985.73–35,086.13)	30,064.02 (27,108.58–33,401.68)	28,230.82 (25,173.28–31,617.59)	26,681.06 (23,473.89–30,280.15)
Ecuador	Group I	10,308.22 (9,664.23–11,044.03)	8,824.55 (8,267.19–9,525.49)	7,623.48 (7,034.84–8,340.95)	6,388.03 (5,805.84–7,106.73)	5,207.11 (4,614.73–5,944.10)	4,421.10 (3,842.65–5,110.33)
Ecuador	NCD	21,030.65 (18,677.89–23,625.10)	20,327.46 (17,988.53–22,955.74)	20,039.46 (17,745.99–22,661.55)	19,709.53 (17,339.06–22,363.17)	19,227.90 (16,840.37–21,910.54)	18,828.83 (16,318.66–21,733.42)
Ecuador	Injuries	4,583.22 (4,343.29–4,854.48)	4,590.99 (4,327.04–4,865.19)	4,195.41 (3,911.72–4,483.19)	3,966.45 (3,675.47–4,249.93)	3,795.80 (3,483.81–4,084.36)	3,431.13 (3,102.27–3,795.25)
Peru	All causes	41,090.28 (37,897.56–44,553.49)	35,836.68 (32,875.35–39,088.95)	29,568.93 (26,692.94–32,682.76)	25,592.66 (22,761.98–28,755.36)	23,649.29 (20,947.31–26,674.12)	22,018.67 (19,065.46–25,232.86)
Peru	Group I	14,879.34 (14,014.70–15,822.73)	11,484.21 (10,832.28–12,201.63)	8,016.67 (7,441.93–8,693.40)	6,320.79 (5,860.98–6,852.80)	5,200.85 (4,760.57–5,706.45)	4,388.95 (3,881.22–4,939.36)
Peru	NCD	21,634.19 (19,128.92–24,320.10)	20,529.56 (18,149.86–23,150.73)	18,441.16 (16,069.68–21,027.72)	16,814.66 (14,498.83–19,382.55)	16,112.92 (13,789.67–18,619.32)	15,563.65 (13,165.76–18,172.93)
Peru	Injuries	4,576.75 (4,032.42–5,079.65)	3,822.91 (3,400.03–4,220.64)	3,111.10 (2,834.67–3,349.09)	2,457.21 (2,247.99–2,646.71)	2,335.52 (2,135.62–2,528.60)	2,066.07 (1,837.17–2,334.92)
Caribbean	All causes	42,500.68 (39,459.85–45,950.45)	40,550.93 (37,502.84–44,002.82)	37,740.16 (34,562.30–41,151.33)	36,430.39 (33,406.07–39,905.42)	66,234.79 (40,209.59–94,084.54)	32,412.34 (29,115.90–36,029.94)
Caribbean	Group I	13,374.78 (12,519.72–14,389.48)	12,523.27 (11,680.79–13,439.73)	11,382.99 (10,634.67–12,227.90)	10,833.82 (10,141.67–11,617.28)	9,231.96 (8,527.73–10,029.65)	7,616.87 (6,700.90–8,633.98)
Caribbean	NCD	24,551.78 (22,112.73–27,150.23)	23,835.14 (21,426.38–26,509.36)	22,657.98 (20,179.43–25,371.28)	22,056.65 (19,585.20–24,814.13)	21,842.26 (19,311.08–24,563.17)	21,454.99 (18,925.23–24,221.44)
Caribbean	Injuries	4,574.12 (4,020.97–4,973.66)	4,192.52 (3,771.10–4,526.74)	3,699.19 (3,346.33–3,986.97)	3,539.92 (3,202.64–3,819.36)	35,160.57 (9,501.29–63,575.45)	3,340.48 (3,026.74–3,669.46)
Antigua and Barbuda	All causes	29,788.81 (26,683.68–33,106.55)	30,029.72 (26,929.20–33,394.78)	29,738.92 (26,699.42–33,108.99)	27,965.00 (24,996.36–31,418.90)	26,522.22 (23,623.67–29,951.40)	25,627.25 (22,472.90–29,117.91)
Antigua and Barbuda	Group I	4,748.82 (4,087.04–5,525.02)	5,165.27 (4,535.29–5,931.57)	5,438.66 (4,805.04–6,165.64)	4,326.71 (3,749.62–5,011.54)	3,859.55 (3,324.82–4,537.49)	3,658.84 (3,084.97–4,442.30)
Antigua and Barbuda	NCD	22,753.74 (20,189.28–25,514.54)	22,619.13 (20,071.24–25,463.93)	22,210.03 (19,617.17–24,959.22)	21,544.48 (19,038.35–24,366.47)	20,760.54 (18,204.39–23,619.16)	20,266.41 (17,630.67–23,244.49)
Antigua and Barbuda	Injuries	2,286.25 (2,106.07–2,494.66)	2,245.32 (2,068.01–2,463.30)	2,090.23 (1,907.37–2,289.39)	2,093.81 (1,935.73–2,262.25)	1,902.13 (1,729.34–2,076.01)	1,702.00 (1,509.05–1,914.96)
The Bahamas	All causes	39,852.11 (36,142.27–43,965.00)	37,649.90 (34,235.47–41,359.99)	36,458.89 (33,117.08–40,151.74)	33,739.13 (30,483.68–37,356.22)	32,174.35 (28,806.29–35,912.86)	30,253.67 (26,685.41–34,150.05)
The Bahamas	Group I	7,801.38 (6,363.62–9,398.71)	7,416.66 (6,548.39–8,460.32)	7,381.38 (6,527.79–8,295.85)	6,503.75 (5,573.77–7,630.78)	6,114.04 (5,177.55–7,254.25)	5,010.79 (4,002.06–6,640.40)
The Bahamas	NCD	27,645.08 (25,028.75–30,570.44)	26,152.55 (23,559.74–29,020.44)	25,167.77 (22,438.20–28,022.58)	23,526.98 (20,926.62–26,366.95)	22,368.45 (19,706.37–25,188.56)	22,028.65 (19,278.23–24,828.77)
The Bahamas	Injuries	4,405.65 (4,045.34–4,796.38)	4,080.69 (3,708.54–4,500.75)	3,909.74 (3,465.06–4,410.84)	3,708.40 (3,311.46–4,116.25)	3,691.86 (3,230.98–4,153.84)	3,214.23 (2,763.93–3,677.59)
Barbados	All causes	31,146.02 (27,670.55–34,722.93)	30,573.56 (27,179.93–34,368.00)	29,529.04 (26,210.94–33,063.10)	28,073.24 (24,931.24–31,551.29)	27,573.46 (24,463.19–30,804.80)	26,889.10 (23,389.38–30,723.66)

Appendix Table 4. Rate of DALYs per 100,000 individuals for all causes and level 1 cause groups by location for 1990 - 2015, both sexes combined.							
	Cause	1990	1995	2000	2005	2010	2015
Barbados	Group I	5,588.81 (4,477.87–6,894.12)	5,443.17 (4,437.48–6,704.25)	5,795.44 (4,848.81–6,953.27)	4,897.48 (4,141.76–5,820.35)	4,532.74 (3,763.34–5,458.23)	4,159.09 (3,262.48–5,389.27)
Barbados	NCD	23,255.77 (20,743.48–25,965.51)	22,998.47 (20,393.19–25,872.03)	21,748.26 (19,202.50–24,613.41)	21,028.40 (18,389.55–23,908.28)	20,951.58 (18,414.40–23,798.56)	20,736.75 (17,930.46–23,672.97)
Barbados	Injuries	2,301.44 (2,144.94–2,474.55)	2,131.92 (1,981.65–2,296.13)	1,985.35 (1,820.89–2,148.24)	2,147.36 (2,007.19–2,297.15)	2,089.15 (1,923.24–2,259.44)	1,993.27 (1,747.55–2,238.31)
Belize	All causes	34,629.08 (31,438.98–38,219.88)	35,491.94 (32,417.44–38,969.32)	38,360.60 (35,269.96–41,784.81)	36,376.68 (33,205.93–39,938.69)	33,900.27 (30,815.56–37,201.74)	32,947.84 (29,130.75–36,644.99)
Belize	Group I	7,989.58 (7,064.26–8,966.14)	6,848.15 (6,081.57–7,796.88)	7,339.79 (6,599.35–8,215.61)	6,961.11 (6,232.87–7,790.09)	6,218.09 (5,418.11–7,269.48)	5,913.99 (4,861.35–7,309.39)
Belize	NCD	22,799.70 (20,305.37–25,436.14)	24,562.66 (22,117.48–27,217.32)	26,017.48 (23,538.32–28,682.49)	24,771.84 (22,224.32–27,656.69)	23,328.08 (20,731.26–26,103.11)	23,035.56 (20,088.51–25,946.99)
Belize	Injuries	3,839.80 (3,515.13–4,196.50)	4,081.12 (3,767.68–4,412.08)	5,003.33 (4,570.86–5,467.68)	4,643.73 (4,260.06–5,060.74)	4,354.10 (3,853.50–4,770.38)	3,998.29 (3,383.29–4,560.24)
Bermuda	All causes	33,844.41 (30,867.27–37,017.95)	31,306.59 (28,352.75–34,429.51)	28,509.63 (25,577.44–31,823.19)	25,377.99 (22,471.62–28,540.48)	23,572.76 (20,761.06–26,738.32)	22,558.70 (19,748.14–25,656.21)
Bermuda	Group I	3,665.51 (3,234.92–4,210.76)	3,742.09 (3,317.80–4,274.26)	3,895.49 (3,498.50–4,380.98)	2,997.13 (2,606.66–3,488.59)	2,673.15 (2,287.91–3,129.71)	2,478.66 (2,080.33–2,974.07)
Bermuda	NCD	26,988.35 (24,535.80–29,613.49)	24,947.48 (22,401.57–27,486.12)	22,577.28 (20,063.07–25,277.71)	20,316.60 (17,847.68–23,022.64)	19,071.23 (16,651.17–21,673.16)	18,519.32 (16,093.92–21,164.91)
Bermuda	Injuries	3,190.56 (2,979.98–3,425.95)	2,617.02 (2,428.50–2,811.66)	2,036.86 (1,818.89–2,227.11)	2,064.25 (1,885.76–2,258.09)	1,828.38 (1,648.12–2,030.58)	1,560.72 (1,359.34–1,764.24)
Cuba	All causes	27,874.62 (25,148.95–30,979.27)	27,183.01 (24,441.81–30,334.67)	25,377.35 (22,685.80–28,497.17)	23,962.86 (21,243.14–27,095.73)	23,067.89 (20,387.91–26,059.71)	22,713.26 (20,007.09–25,749.06)
Cuba	Group I	3,512.32 (3,118.25–4,030.64)	3,214.33 (2,826.62–3,725.84)	2,847.53 (2,480.79–3,328.30)	2,597.69 (2,245.53–3,064.75)	2,482.29 (2,128.07–2,927.14)	2,424.24 (2,063.65–2,875.83)
Cuba	NCD	20,557.07 (18,269.71–23,070.19)	20,454.65 (18,210.91–22,959.25)	19,729.73 (17,431.85–22,263.92)	19,110.95 (16,815.58–21,671.96)	18,575.42 (16,306.09–21,115.00)	18,318.75 (16,053.41–20,865.75)
Cuba	Injuries	3,805.23 (3,641.20–3,985.99)	3,514.03 (3,355.04–3,688.03)	2,800.09 (2,664.31–2,959.10)	2,254.22 (2,135.72–2,396.52)	2,010.18 (1,896.61–2,148.60)	1,970.26 (1,844.67–2,122.08)
Dominica	All causes	31,853.27 (28,590.45–35,541.35)	31,961.60 (28,662.85–35,615.93)	30,663.99 (27,359.34–34,131.74)	30,191.66 (26,937.30–33,740.31)	31,056.18 (27,600.80–34,798.15)	31,930.50 (27,931.75–36,124.40)
Dominica	Group I	5,695.12 (4,794.85–6,678.10)	5,616.96 (4,803.96–6,516.22)	5,568.33 (4,781.04–6,420.76)	5,412.50 (4,629.95–6,357.32)	5,466.57 (4,569.04–6,653.71)	5,539.52 (4,367.02–7,024.75)
Dominica	NCD	23,816.28 (21,260.22–26,826.97)	23,961.20 (21,336.18–26,931.80)	22,699.80 (20,053.68–25,560.89)	22,237.89 (19,627.17–25,073.33)	22,825.98 (20,076.38–25,822.01)	23,608.17 (20,644.13–26,763.23)
Dominica	Injuries	2,341.88 (2,145.14–2,565.26)	2,383.44 (2,186.18–2,599.02)	2,395.87 (2,186.45–2,610.12)	2,541.27 (2,315.91–2,769.17)	2,763.63 (2,484.54–3,012.56)	2,782.81 (2,441.05–3,153.42)
Dominican Republic	All causes	35,345.41 (32,371.85–38,671.93)	33,362.03 (30,450.17–36,595.86)	31,997.76 (29,040.70–35,321.88)	31,975.84 (29,117.42–35,253.15)	29,824.31 (26,922.95–33,105.26)	28,186.55 (25,249.91–31,494.26)
Dominican Republic	Group I	11,256.74 (10,451.54–12,054.16)	9,678.21 (8,956.40–10,485.19)	9,307.17 (8,418.25–10,299.94)	9,324.77 (8,511.92–10,217.61)	7,334.02 (6,601.62–8,146.03)	5,869.07 (5,054.50–6,659.14)
Dominican Republic	NCD	20,698.89 (18,199.08–23,317.10)	20,501.24 (18,074.83–23,143.18)	19,504.54 (17,098.96–22,173.02)	19,346.59 (16,933.59–21,959.92)	19,276.95 (16,882.22–21,925.14)	19,256.56 (16,937.51–21,865.31)
Dominican Republic	Injuries	3,389.78 (3,150.85–3,685.43)	3,182.58 (2,937.38–3,435.89)	3,186.05 (2,964.09–3,429.39)	3,304.48 (3,072.75–3,523.63)	3,213.35 (2,902.81–3,457.56)	3,060.92 (2,817.36–3,347.19)
Grenada	All causes	37,112.93 (33,267.27–41,116.99)	35,069.69 (31,624.04–38,637.02)	35,383.68 (32,056.27–39,124.29)	36,022.72 (32,833.80–39,857.94)	34,682.86 (31,416.84–38,438.95)	33,914.54 (30,282.21–37,865.29)
Grenada	Group I	7,164.35 (5,676.71–8,963.85)	5,894.29 (4,936.82–6,955.26)	5,912.04 (5,049.10–7,006.17)	5,494.49 (4,633.67–6,435.23)	5,111.87 (4,301.92–6,129.39)	5,000.74 (4,010.27–6,340.73)
Grenada	NCD	26,868.83 (24,212.36–29,621.78)	26,250.45 (23,701.29–29,075.04)	26,224.56 (23,657.35–29,077.49)	26,855.88 (24,232.79–29,817.07)	26,201.10 (23,622.17–29,124.16)	25,865.56 (22,954.69–28,914.16)
Grenada	Injuries	3,079.75 (2,818.61–3,367.87)	2,924.95 (2,713.62–3,155.08)	3,247.08 (3,013.00–3,505.55)	3,672.36 (3,323.56–4,040.49)	3,369.89 (3,036.79–3,691.60)	3,048.24 (2,662.72–3,408.23)

Appendix Table 4. Rate of DALYs per 100,000 individuals for all causes and level 1 cause groups by location for 1990 - 2015, both sexes combined.							
	Cause	1990	1995	2000	2005	2010	2015
Guyana	All causes	49,736.86 (46,248.06–53,524.10)	50,345.81 (46,986.84–54,020.94)	49,417.01 (45,783.23–53,185.42)	48,956.27 (45,309.94–52,950.97)	46,345.01 (42,692.11–50,075.61)	43,471.79 (39,470.09–47,859.70)
Guyana	Group I	12,489.80 (11,629.05–13,421.21)	12,011.31 (11,194.22–12,843.06)	12,547.09 (11,638.99–13,594.53)	11,841.63 (10,806.48–13,391.70)	11,210.30 (9,884.33–12,927.44)	9,924.61 (8,546.85–11,532.59)
Guyana	NCD	31,835.94 (29,205.33–34,692.20)	32,856.39 (30,248.52–35,741.10)	30,913.39 (28,072.13–33,906.62)	30,813.82 (28,095.36–33,978.90)	29,444.47 (26,607.26–32,479.63)	28,245.68 (25,203.93–31,545.50)
Guyana	Injuries	5,411.12 (5,067.68–5,897.31)	5,478.12 (5,125.12–5,943.85)	5,956.53 (5,480.08–6,463.38)	6,300.82 (5,606.21–6,977.48)	5,690.24 (4,994.31–6,396.49)	5,301.50 (4,475.83–6,128.84)
Haiti	All causes	77,399.46 (72,188.75–82,745.61)	71,553.64 (66,595.47–76,950.68)	64,334.20 (58,674.58–70,013.33)	60,660.98 (55,279.69–66,915.40)	184,678.51 (81,528.43–298,980.40)	50,396.58 (43,716.34–57,379.48)
Haiti	Group I	32,012.31 (29,383.22–35,232.93)	30,923.61 (28,104.93–34,049.36)	27,662.29 (25,148.75–30,371.81)	25,292.65 (23,251.84–27,407.72)	20,101.36 (18,185.60–22,207.62)	15,642.26 (13,192.04–18,563.78)
Haiti	NCD	37,506.05 (34,137.22–41,279.17)	34,036.97 (30,181.83–37,863.90)	31,191.22 (27,221.60–35,350.09)	30,313.91 (26,128.75–34,918.34)	30,598.50 (26,175.03–34,991.40)	29,567.75 (25,084.97–34,214.80)
Haiti	Injuries	7,881.10 (5,818.47–9,362.43)	6,593.06 (5,102.41–7,757.15)	5,480.69 (4,373.38–6,489.23)	5,054.42 (3,993.33–6,052.76)	133,978.65 (30,140.09–248,852.87)	5,186.57 (4,026.97–6,423.35)
Jamaica	All causes	28,990.25 (25,852.81–32,606.99)	29,045.97 (25,851.87–32,652.90)	29,757.99 (26,578.87–33,297.51)	28,621.15 (25,351.74–32,308.14)	28,985.38 (25,551.10–32,523.48)	28,671.95 (25,092.94–32,670.40)
Jamaica	Group I	5,877.53 (4,943.88–6,966.93)	5,504.20 (4,617.89–6,652.38)	5,717.04 (4,829.00–6,777.41)	5,347.66 (4,454.64–6,534.86)	5,134.91 (4,135.30–6,233.22)	4,735.75 (3,751.86–5,996.91)
Jamaica	NCD	21,603.88 (19,132.88–24,398.34)	21,589.29 (19,129.82–24,426.39)	21,769.14 (19,273.65–24,535.68)	20,539.47 (17,985.97–23,309.62)	21,220.46 (18,593.54–23,973.96)	21,471.28 (18,592.93–24,558.39)
Jamaica	Injuries	1,508.84 (1,371.69–1,709.31)	1,952.49 (1,743.22–2,137.37)	2,271.81 (1,961.90–2,484.60)	2,734.03 (2,183.03–3,030.84)	2,630.00 (1,961.21–2,908.64)	2,464.93 (1,876.79–2,818.87)
Puerto Rico	All causes	30,287.47 (27,392.31–33,548.61)	31,020.61 (28,160.76–34,278.63)	27,543.33 (24,683.40–30,796.97)	26,073.78 (23,219.31–29,354.90)	25,198.50 (22,426.09–28,331.65)	23,566.73 (20,714.34–26,713.40)
Puerto Rico	Group I	5,147.85 (4,791.56–5,613.44)	5,592.12 (5,240.61–6,049.22)	3,993.53 (3,644.55–4,451.81)	3,465.00 (3,130.80–3,901.43)	3,027.37 (2,697.70–3,430.07)	2,714.72 (2,381.41–3,148.45)
Puerto Rico	NCD	21,406.04 (18,954.21–24,110.26)	21,560.72 (19,191.03–24,309.14)	20,382.03 (17,971.46–23,102.14)	19,683.58 (17,223.04–22,472.04)	19,220.82 (16,833.70–21,930.31)	18,276.07 (15,766.17–21,024.52)
Puerto Rico	Injuries	3,733.59 (3,573.17–3,893.98)	3,867.76 (3,702.39–4,031.42)	3,167.77 (3,031.38–3,313.17)	2,925.20 (2,804.26–3,069.34)	2,950.32 (2,838.62–3,082.25)	2,575.95 (2,389.97–2,767.01)
Saint Lucia	All causes	37,024.69 (33,239.70–41,535.70)	34,722.53 (31,321.94–38,399.85)	32,955.23 (29,709.15–36,595.09)	31,335.94 (28,088.53–35,114.53)	29,400.76 (26,081.62–32,963.36)	28,740.44 (25,197.04–32,739.87)
Saint Lucia	Group I	7,575.75 (5,981.31–9,454.97)	6,007.17 (5,075.05–7,134.00)	5,488.64 (4,647.49–6,417.55)	5,083.95 (4,178.04–6,215.73)	4,710.59 (3,817.87–5,812.99)	4,247.01 (3,238.82–5,716.63)
Saint Lucia	NCD	26,250.57 (23,665.07–29,051.24)	25,646.23 (23,072.92–28,384.44)	24,437.91 (21,868.82–27,314.67)	23,161.57 (20,622.79–26,013.57)	21,685.67 (19,177.69–24,399.94)	21,840.38 (19,186.07–24,795.72)
Saint Lucia	Injuries	3,198.37 (2,915.90–3,502.14)	3,069.13 (2,847.37–3,307.96)	3,028.68 (2,788.44–3,263.32)	3,090.42 (2,881.10–3,328.32)	3,004.50 (2,801.51–3,220.75)	2,653.05 (2,375.02–2,972.57)
Saint Vincent and the Grenadines	All causes	37,614.76 (33,887.59–41,758.60)	37,836.38 (34,282.67–41,602.63)	38,696.53 (35,297.43–42,485.09)	36,115.76 (32,852.56–39,751.45)	34,975.79 (31,724.96–38,548.46)	34,420.65 (30,868.08–38,230.56)
Saint Vincent and the Grenadines	Group I	8,336.92 (6,924.94–10,098.61)	7,887.39 (6,789.19–9,121.08)	8,688.55 (7,707.05–9,834.51)	6,887.03 (6,016.60–8,003.97)	6,252.83 (5,344.73–7,481.14)	5,684.31 (4,629.10–7,086.01)
Saint Vincent and the Grenadines	NCD	25,998.40 (23,178.15–28,792.75)	26,836.78 (24,196.19–29,777.20)	26,981.55 (24,328.74–29,865.28)	25,853.49 (23,224.67–28,768.61)	25,369.08 (22,731.95–28,232.59)	25,444.56 (22,744.16–28,701.28)
Saint Vincent and the Grenadines	Injuries	3,279.44 (2,973.48–3,615.87)	3,112.21 (2,839.24–3,401.94)	3,026.43 (2,795.11–3,273.12)	3,375.24 (3,117.94–3,655.51)	3,353.86 (3,102.68–3,616.75)	3,291.78 (2,935.25–3,641.57)
Suriname	All causes	38,308.40 (35,122.33–41,718.57)	37,616.63 (34,319.27–41,134.46)	38,085.23 (34,802.79–41,694.08)	37,486.23 (34,234.46–41,087.97)	35,727.61 (32,314.60–39,278.15)	33,368.21 (29,683.17–37,020.90)
Suriname	Group I	9,286.51 (8,484.50–10,230.29)	9,182.03 (8,289.79–10,074.19)	9,284.61 (8,387.60–10,298.80)	8,426.48 (7,486.91–9,475.49)	7,713.65 (6,778.23–8,808.85)	6,825.11 (5,911.24–7,936.01)
Suriname	NCD	24,627.98 (22,054.42–27,350.82)	24,161.55 (21,695.16–26,930.90)	24,480.49 (21,920.90–27,323.49)	24,520.69 (21,834.83–27,454.13)	23,619.50 (20,938.59–26,468.10)	22,546.88 (19,631.41–25,571.16)

Appendix Table 4. Rate of DALYs per 100,000 individuals for all causes and level 1 cause groups by location for 1990 - 2015, both sexes combined.

	Cause	1990	1995	2000	2005	2010	2015
Suriname	Injuries	4,393.91 (4,103.02–4,720.22)	4,273.04 (3,972.66–4,600.54)	4,320.13 (3,988.82–4,665.49)	4,539.06 (4,172.28–4,959.80)	4,394.46 (3,980.48–4,832.10)	3,996.22 (3,543.65–4,510.15)
Trinidad and Tobago	All causes	38,198.58 (34,946.16–41,637.51)	37,783.23 (34,643.91–41,118.16)	37,649.01 (34,488.51–40,993.38)	34,833.43 (31,694.63–38,360.66)	33,607.20 (30,231.61–37,010.24)	32,565.77 (28,877.82–36,558.03)
Trinidad and Tobago	Group I	6,286.78 (5,657.16–6,977.33)	6,542.54 (5,896.93–7,220.55)	7,390.63 (6,746.67–8,152.19)	5,657.14 (4,983.22–6,419.49)	4,956.68 (4,167.35–5,864.24)	4,618.73 (3,699.07–5,793.62)
Trinidad and Tobago	NCD	28,282.03 (25,673.45–31,063.95)	27,870.09 (25,367.78–30,668.50)	27,061.91 (24,549.60–29,950.34)	25,298.51 (22,760.71–28,131.34)	24,727.83 (22,011.07–27,644.77)	24,351.80 (21,427.66–27,462.96)
Trinidad and Tobago	Injuries	3,629.78 (3,450.26–3,820.65)	3,370.60 (3,215.38–3,541.74)	3,196.47 (3,036.08–3,348.27)	3,877.77 (3,715.74–4,048.35)	3,922.69 (3,697.68–4,128.88)	3,595.24 (3,217.12–4,010.69)
Virgin Islands, U.S.	All causes	30,765.61 (27,791.88–34,106.14)	30,231.90 (27,285.03–33,446.84)	29,298.31 (26,450.04–32,599.75)	28,649.21 (25,745.49–31,937.95)	28,743.45 (25,692.38–31,974.77)	28,569.86 (25,228.31–31,921.35)
Virgin Islands, U.S.	Group I	3,747.86 (3,327.70–4,232.16)	3,516.06 (3,080.15–4,040.08)	3,359.96 (2,964.74–3,845.30)	3,017.55 (2,646.59–3,501.59)	2,802.86 (2,409.19–3,287.28)	2,701.59 (2,303.33–3,193.61)
Virgin Islands, U.S.	NCD	23,718.18 (21,270.07–26,379.77)	23,221.11 (20,780.86–25,884.92)	22,753.23 (20,283.09–25,542.31)	22,261.98 (19,692.14–24,968.45)	22,488.91 (19,914.56–25,224.75)	22,494.65 (19,717.69–25,411.80)
Virgin Islands, U.S.	Injuries	3,299.57 (3,050.15–3,616.03)	3,494.73 (3,185.88–3,862.79)	3,185.12 (2,940.86–3,447.02)	3,369.68 (3,106.15–3,617.95)	3,451.68 (3,115.56–3,740.21)	3,373.62 (3,005.19–3,763.38)
Tropical Latin America	All causes	41,044.55 (38,058.27–44,437.84)	38,722.82 (35,787.16–41,988.70)	35,696.37 (32,754.61–38,970.44)	32,670.65 (29,731.71–35,924.60)	30,668.04 (27,652.93–33,939.11)	29,368.99 (26,367.29–32,632.82)
Tropical Latin America	Group I	10,446.41 (9,922.74–10,990.61)	8,667.70 (8,239.19–9,162.56)	6,971.68 (6,571.40–7,471.73)	5,783.31 (5,406.10–6,248.59)	5,055.96 (4,661.92–5,527.78)	4,467.23 (4,060.41–4,946.03)
Tropical Latin America	NCD	25,409.88 (22,846.41–28,105.15)	24,923.11 (22,419.54–27,555.95)	23,965.14 (21,415.19–26,756.08)	22,458.69 (19,917.80–25,213.71)	21,368.08 (18,848.30–24,123.95)	20,944.34 (18,425.86–23,647.08)
Tropical Latin America	Injuries	5,188.25 (4,985.59–5,421.72)	5,132.01 (4,924.00–5,355.16)	4,759.55 (4,565.88–4,980.59)	4,428.65 (4,243.93–4,626.96)	4,243.99 (4,053.96–4,427.51)	3,957.43 (3,743.59–4,174.91)
Brazil	All causes	41,381.38 (38,403.74–44,779.86)	38,991.28 (36,054.41–42,254.86)	35,862.32 (32,918.95–39,123.85)	32,746.79 (29,813.36–36,017.14)	30,698.18 (27,700.56–33,968.87)	29,381.06 (26,375.40–32,627.94)
Brazil	Group I	10,577.04 (10,041.70–11,130.47)	8,756.92 (8,317.56–9,249.16)	7,000.40 (6,596.41–7,504.42)	5,790.62 (5,412.38–6,261.10)	5,062.09 (4,667.05–5,533.80)	4,470.56 (4,062.09–4,950.32)
Brazil	NCD	25,550.61 (22,995.51–28,238.90)	25,045.22 (22,540.02–27,677.15)	24,061.40 (21,513.46–26,858.72)	22,494.96 (19,944.97–25,232.46)	21,364.50 (18,850.37–24,120.39)	20,930.98 (18,420.25–23,639.30)
Brazil	Injuries	5,253.74 (5,050.98–5,490.63)	5,189.14 (4,978.80–5,415.09)	4,800.51 (4,604.81–5,021.19)	4,461.21 (4,274.86–4,659.89)	4,271.59 (4,080.08–4,456.96)	3,979.52 (3,762.98–4,200.74)
Paraguay	All causes	29,780.63 (26,688.60–33,010.52)	29,748.08 (26,640.45–33,115.73)	29,780.18 (26,745.02–33,134.31)	29,907.67 (26,729.08–33,284.17)	29,719.71 (26,492.51–33,182.96)	29,067.78 (25,514.68–32,973.87)
Paraguay	Group I	6,880.79 (6,356.16–7,432.18)	6,229.92 (5,728.96–6,780.39)	6,072.94 (5,558.03–6,696.39)	5,516.49 (4,977.13–6,128.96)	4,832.60 (4,230.01–5,507.27)	4,313.70 (3,712.32–5,028.40)
Paraguay	NCD	20,165.01 (17,602.40–22,902.20)	20,467.18 (17,834.71–23,280.12)	20,409.90 (17,808.67–23,129.41)	21,055.86 (18,401.80–23,994.07)	21,487.54 (18,944.25–24,491.06)	21,440.87 (18,700.63–24,582.27)
Paraguay	Injuries	2,734.83 (2,510.55–2,958.47)	3,050.98 (2,805.87–3,314.04)	3,297.33 (3,044.75–3,563.42)	3,335.32 (3,068.06–3,623.02)	3,399.57 (3,084.39–3,736.47)	3,313.21 (2,861.24–3,922.60)
Southeast Asia, East Asia, and Oceania	All causes	43,364.03 (40,613.34–46,557.77)	39,991.57 (37,252.06–43,079.69)	37,117.42 (34,551.50–39,977.96)	33,195.99 (30,547.34–36,105.23)	29,341.23 (26,651.69–32,253.01)	26,536.15 (23,992.06–29,426.90)
Southeast Asia, East Asia, and Oceania	Group I	11,795.13 (11,071.77–12,625.16)	9,979.15 (9,353.84–10,655.08)	8,375.25 (7,849.79–8,970.97)	6,230.58 (5,816.17–6,714.28)	4,897.58 (4,525.74–5,333.94)	4,028.68 (3,690.25–4,435.57)
Southeast Asia, East Asia, and Oceania	NCD	26,831.18 (24,576.13–29,370.68)	25,648.19 (23,426.44–28,064.21)	24,792.40 (22,686.14–27,122.36)	23,444.68 (21,244.86–25,791.62)	21,357.01 (19,119.89–23,714.94)	19,866.14 (17,753.82–22,215.73)
Southeast Asia, East Asia, and Oceania	Injuries	4,737.72 (4,362.95–5,024.44)	4,364.23 (4,049.01–4,604.71)	3,949.76 (3,754.93–4,133.28)	3,520.73 (3,326.20–3,693.62)	3,086.64 (2,881.98–3,254.78)	2,641.33 (2,443.34–2,820.57)
East Asia	All causes	41,882.96 (39,135.08–45,184.34)	38,563.14 (35,919.05–41,546.12)	35,533.91 (32,990.25–38,244.56)	30,951.32 (28,336.57–33,748.99)	26,813.65 (24,344.07–29,653.10)	23,927.63 (21,488.76–26,658.49)
East Asia	Group I	9,256.82 (8,573.12–9,981.95)	7,977.34 (7,357.10–8,624.03)	6,524.20 (5,952.45–7,125.60)	4,098.61 (3,756.78–4,508.42)	3,022.89 (2,737.33–3,390.57)	2,486.45 (2,201.34–2,841.66)

Appendix Table 4. Rate of DALYs per 100,000 individuals for all causes and level 1 cause groups by location for 1990 - 2015, both sexes combined.

	Cause	1990	1995	2000	2005	2010	2015
East Asia	NCD	27,538.98 (25,319.86–30,045.08)	25,966.75 (23,779.53–28,289.26)	24,892.61 (22,773.59–27,260.87)	23,256.19 (21,112.83–25,597.19)	20,688.02 (18,585.40–23,013.38)	18,861.36 (16,841.05–21,180.02)
East Asia	Injuries	5,087.16 (4,623.75–5,424.45)	4,619.06 (4,244.65–4,898.17)	4,117.10 (3,909.92–4,305.23)	3,596.53 (3,380.22–3,781.44)	3,102.73 (2,876.11–3,286.62)	2,579.82 (2,373.76–2,773.48)
China	All causes	42,160.68 (39,415.19–45,447.85)	38,359.90 (35,654.09–41,281.18)	35,213.97 (32,671.04–38,054.65)	31,028.28 (28,422.00–33,825.36)	26,818.58 (24,342.78–29,661.39)	23,867.88 (21,435.71–26,616.64)
China	Group I	9,353.01 (8,673.21–10,080.48)	7,638.00 (7,135.09–8,236.87)	6,095.01 (5,707.21–6,531.68)	4,096.13 (3,757.15–4,502.73)	3,002.42 (2,714.40–3,364.58)	2,465.72 (2,181.96–2,816.07)
China	NCD	27,687.74 (25,429.66–30,191.83)	26,077.47 (23,898.72–28,400.20)	24,984.01 (22,874.33–27,367.14)	23,325.21 (21,184.21–25,647.30)	20,707.15 (18,594.27–23,008.90)	18,830.11 (16,834.02–21,148.94)
China	Injuries	5,119.93 (4,647.42–5,457.70)	4,644.42 (4,262.02–4,925.73)	4,134.96 (3,925.34–4,322.42)	3,606.95 (3,392.35–3,796.10)	3,109.02 (2,876.51–3,297.33)	2,572.05 (2,357.83–2,767.94)
North Korea	All causes	38,085.33 (30,125.26–48,422.46)	59,838.94 (39,135.28–82,190.60)	59,510.71 (38,972.43–81,660.10)	34,659.33 (29,211.90–40,734.19)	32,533.62 (28,181.09–37,804.91)	30,895.75 (26,132.10–36,573.39)
North Korea	Group I	8,622.97 (5,095.59–14,229.25)	30,486.65 (10,845.05–51,828.92)	29,534.99 (9,729.21–50,722.31)	5,578.42 (3,669.36–7,925.73)	4,622.94 (3,152.12–6,573.36)	3,852.06 (2,559.77–5,701.44)
North Korea	NCD	24,836.80 (20,403.32–29,798.90)	25,006.50 (20,609.45–29,437.23)	25,601.36 (21,215.16–30,112.06)	24,948.68 (21,330.94–28,706.49)	24,137.03 (21,037.67–27,559.53)	23,497.04 (19,929.58–27,412.52)
North Korea	Injuries	4,625.55 (3,365.31–6,507.74)	4,345.80 (3,272.08–5,728.83)	4,374.36 (3,368.79–5,682.75)	4,132.23 (3,337.84–5,110.51)	3,773.66 (3,099.28–4,556.50)	3,546.65 (2,804.28–4,500.79)
Taiwan	All causes	27,977.06 (25,373.71–31,021.91)	26,356.55 (23,900.54–29,121.85)	24,440.90 (21,962.15–27,211.29)	22,695.80 (20,234.77–25,508.20)	20,752.89 (18,290.87–23,534.57)	20,378.46 (17,549.41–23,420.86)
Taiwan	Group I	2,828.77 (2,288.15–3,648.39)	2,233.55 (1,889.92–2,675.42)	2,038.00 (1,728.69–2,406.47)	1,869.23 (1,527.70–2,235.87)	1,771.96 (1,375.58–2,182.07)	1,816.72 (1,375.60–2,286.23)
Taiwan	NCD	21,656.86 (19,576.35–24,032.47)	20,877.37 (18,818.55–23,261.82)	19,653.72 (17,555.38–22,029.56)	18,409.72 (16,280.44–20,842.20)	16,950.63 (14,775.25–19,301.15)	16,604.28 (14,191.36–19,180.16)
Taiwan	Injuries	3,491.43 (3,196.12–3,772.93)	3,245.64 (2,987.23–3,480.37)	2,749.17 (2,565.14–2,951.13)	2,416.85 (2,274.54–2,578.85)	2,030.29 (1,908.42–2,167.95)	1,957.46 (1,701.69–2,228.56)
Southeast Asia	All causes	46,591.27 (43,381.55–50,279.26)	43,141.50 (39,972.70–46,825.79)	40,521.95 (37,316.14–43,854.66)	37,351.40 (34,218.33–40,798.94)	34,496.92 (31,388.14–37,723.27)	32,090.92 (28,861.91–35,554.90)
Southeast Asia	Group I	18,186.71 (16,991.22–19,574.97)	14,811.22 (13,833.77–15,945.26)	12,467.63 (11,667.44–13,440.26)	10,393.58 (9,706.26–11,172.95)	8,508.72 (7,909.74–9,182.63)	6,992.38 (6,420.45–7,643.03)
Southeast Asia	NCD	24,456.70 (21,928.83–27,222.51)	24,514.42 (21,901.51–27,259.90)	24,396.05 (21,937.52–26,956.51)	23,598.51 (21,124.05–26,172.07)	22,876.88 (20,416.20–25,495.37)	22,298.05 (19,730.24–25,037.67)
Southeast Asia	Injuries	3,947.86 (3,507.67–4,339.03)	3,815.86 (3,468.16–4,130.19)	3,658.27 (3,385.10–3,929.28)	3,359.31 (3,117.13–3,604.88)	3,111.32 (2,880.85–3,361.64)	2,800.49 (2,534.19–3,069.09)
Cambodia	All causes	67,390.33 (63,310.87–72,215.59)	65,745.76 (61,877.19–70,062.02)	60,363.72 (56,681.42–64,288.38)	49,963.46 (46,403.45–53,708.10)	42,572.34 (38,946.38–46,389.65)	37,331.65 (33,060.84–41,490.72)
Cambodia	Group I	29,792.91 (27,342.15–32,046.46)	28,011.69 (25,899.51–30,130.64)	23,921.71 (22,006.83–25,707.07)	16,651.78 (15,265.28–18,000.60)	12,167.45 (11,183.29–13,237.15)	9,399.22 (8,437.26–10,439.77)
Cambodia	NCD	31,348.28 (28,048.80–34,786.05)	31,337.80 (28,336.06–34,531.73)	30,638.89 (27,594.06–33,711.05)	28,555.81 (25,748.03–31,479.06)	26,251.40 (23,410.70–29,251.52)	24,415.10 (21,248.69–27,478.48)
Cambodia	Injuries	6,249.14 (5,114.04–7,264.49)	6,396.28 (5,223.19–7,391.66)	5,803.12 (4,709.32–6,742.70)	4,755.87 (4,009.79–5,472.51)	4,153.49 (3,612.63–4,697.03)	3,517.33 (2,966.27–4,045.52)
Indonesia	All causes	49,878.45 (46,555.58–53,469.04)	44,696.85 (41,567.98–48,149.84)	41,418.65 (38,479.87–44,697.07)	38,728.89 (35,622.96–42,164.88)	36,299.87 (32,842.17–40,169.88)	33,810.35 (29,413.59–38,112.95)
Indonesia	Group I	21,943.89 (20,317.81–23,992.87)	17,249.12 (16,112.80–18,718.05)	14,022.50 (13,106.17–15,240.59)	11,715.55 (10,892.52–12,791.83)	9,765.32 (8,959.81–10,752.10)	8,112.37 (7,179.92–9,239.04)
Indonesia	NCD	24,637.62 (21,966.22–27,456.44)	24,531.65 (21,961.65–27,130.43)	24,621.56 (22,143.48–27,209.22)	24,411.30 (21,870.39–27,012.51)	24,082.72 (21,346.70–27,007.52)	23,423.20 (20,052.97–26,819.74)
Indonesia	Injuries	3,296.93 (2,465.62–3,968.76)	2,916.09 (2,303.73–3,356.63)	2,774.60 (2,369.00–3,090.75)	2,602.04 (2,310.98–2,871.12)	2,451.83 (2,161.61–2,797.45)	2,274.77 (1,895.96–2,716.09)
Laos	All causes	84,928.18 (76,956.28–92,686.15)	78,184.96 (71,912.07–84,428.05)	70,704.75 (66,085.77–75,540.03)	59,778.19 (55,609.04–64,206.60)	49,521.53 (45,190.81–53,865.24)	42,810.05 (37,739.91–48,560.68)

Appendix Table 4. Rate of DALYs per 100,000 individuals for all causes and level 1 cause groups by location for 1990 - 2015, both sexes combined.							
	**Cause**	**1990**	**1995**	**2000**	**2005**	**2010**	**2015**
Laos	Group I	40,514.49 (36,289.97–44,652.65)	34,671.47 (31,055.92–38,588.32)	29,239.57 (26,177.61–32,882.27)	22,351.01 (19,873.03–25,073.02)	15,909.20 (14,007.88–17,993.33)	11,958.38 (9,935.73–14,496.63)
Laos	NCD	36,534.93 (31,066.48–42,622.19)	36,137.44 (31,598.23–41,118.82)	34,453.10 (30,509.86–38,407.57)	31,296.83 (28,097.43–34,939.94)	28,453.05 (25,238.05–31,766.06)	26,456.48 (22,861.47–30,344.15)
Laos	Injuries	7,878.76 (5,523.00–9,942.21)	7,376.05 (5,209.77–9,365.80)	7,012.08 (4,934.74–8,718.58)	6,130.34 (4,540.05–7,610.64)	5,159.28 (4,096.76–6,243.84)	4,395.18 (3,593.47–5,323.24)
Malaysia	All causes	32,034.75 (29,340.21–35,135.40)	30,914.03 (28,168.63–33,910.36)	29,292.54 (26,524.25–32,243.33)	27,647.29 (24,975.03–30,642.49)	27,344.56 (24,612.69–30,405.01)	26,075.11 (22,908.40–29,516.74)
Malaysia	Group I	6,429.70 (5,755.90–7,239.69)	5,598.73 (5,035.28–6,278.33)	5,139.48 (4,575.64–5,768.06)	4,804.46 (4,034.62–5,504.83)	4,698.35 (3,950.68–5,396.68)	4,167.16 (3,456.96–4,902.35)
Malaysia	NCD	22,550.85 (20,307.59–24,946.17)	22,241.71 (20,017.79–24,639.52)	21,251.12 (19,009.62–23,628.27)	20,200.85 (17,921.06–22,572.13)	20,020.81 (17,761.97–22,410.14)	19,421.40 (16,815.95–22,161.81)
Malaysia	Injuries	3,054.20 (2,808.23–3,280.93)	3,073.59 (2,843.61–3,267.60)	2,901.94 (2,688.87–3,090.35)	2,641.98 (2,450.43–2,810.02)	2,625.40 (2,419.88–2,808.32)	2,486.55 (2,186.22–2,795.55)
Maldives	All causes	46,605.38 (43,172.63–50,442.06)	39,277.00 (35,999.94–43,189.61)	32,367.41 (29,251.42–35,913.71)	26,023.00 (23,087.67–29,375.70)	22,412.77 (19,551.87–25,664.49)	20,914.73 (17,862.90–24,243.77)
Maldives	Group I	12,371.34 (10,302.69–15,493.20)	8,877.73 (7,713.39–10,543.36)	6,255.65 (5,542.66–7,102.62)	4,772.89 (4,132.81–5,545.28)	3,586.78 (3,061.77–4,177.12)	2,934.40 (2,469.00–3,492.19)
Maldives	NCD	27,569.44 (24,787.62–30,625.36)	24,939.26 (22,276.89–27,841.59)	21,698.66 (19,175.93–24,429.89)	18,043.93 (15,735.30–20,672.05)	16,263.72 (13,940.19–18,829.85)	15,767.70 (13,294.25–18,499.83)
Maldives	Injuries	6,664.61 (5,045.73–7,952.55)	5,460.01 (4,560.98–6,125.84)	4,413.10 (4,080.25–4,751.49)	3,206.19 (2,909.53–3,461.03)	2,562.26 (2,373.63–2,776.87)	2,212.63 (1,922.04–2,520.35)
Mauritius	All causes	36,402.80 (33,753.59–39,278.38)	34,428.77 (31,828.95–37,220.20)	32,209.91 (29,646.80–35,097.33)	30,691.11 (28,151.67–33,579.04)	29,353.62 (26,751.46–32,345.13)	27,619.36 (24,928.25–30,711.65)
Mauritius	Group I	5,298.08 (4,890.17–5,919.52)	4,405.83 (4,049.05–4,979.89)	3,580.47 (3,281.10–4,035.65)	3,073.69 (2,829.35–3,410.38)	3,058.73 (2,801.94–3,443.17)	2,809.84 (2,551.72–3,171.03)
Mauritius	NCD	28,595.45 (26,248.47–31,184.27)	27,445.36 (25,089.24–30,000.00)	26,267.94 (23,960.44–28,855.57)	25,410.08 (23,116.46–28,027.90)	24,124.76 (21,773.88–26,895.66)	22,887.73 (20,379.62–25,698.08)
Mauritius	Injuries	2,509.27 (2,315.80–2,661.84)	2,577.59 (2,399.95–2,730.45)	2,361.50 (2,187.14–2,510.09)	2,207.35 (2,088.68–2,327.73)	2,170.13 (2,041.34–2,297.81)	1,921.79 (1,777.91–2,064.95)
Myanmar	All causes	66,985.67 (53,429.37–82,904.52)	62,881.14 (50,296.06–77,265.76)	57,996.27 (46,112.22–72,152.10)	51,452.40 (40,153.38–63,920.47)	44,796.18 (35,025.86–57,466.72)	40,237.53 (31,457.39–50,931.37)
Myanmar	Group I	30,650.75 (25,451.44–36,696.21)	26,862.60 (22,365.34–33,073.48)	23,070.87 (18,912.55–29,473.73)	18,309.56 (15,243.87–22,962.63)	13,448.10 (10,886.47–17,018.43)	10,148.55 (8,116.14–12,751.48)
Myanmar	NCD	32,205.75 (23,859.75–41,865.88)	31,930.86 (23,470.91–40,820.69)	31,215.75 (22,872.65–40,585.52)	29,693.39 (21,842.55–38,278.50)	28,053.19 (21,060.71–37,023.74)	27,009.09 (20,403.81–34,878.31)
Myanmar	Injuries	4,129.17 (3,113.61–5,358.10)	4,087.67 (2,962.73–5,372.36)	3,709.65 (2,581.98–5,032.16)	3,449.45 (2,359.00–4,573.34)	3,294.89 (2,382.09–4,449.36)	3,079.89 (2,197.96–4,113.06)
Philippines	All causes	43,061.88 (40,089.63–46,576.08)	39,776.51 (36,741.02–43,141.07)	38,848.87 (35,846.10–42,248.04)	38,705.55 (35,778.60–42,125.48)	36,471.78 (33,535.60–39,777.05)	34,554.40 (31,380.83–38,105.10)
Philippines	Group I	16,456.69 (15,631.05–17,507.15)	13,359.64 (12,544.09–14,415.82)	11,469.64 (10,735.33–12,374.83)	10,531.91 (9,854.69–11,356.72)	9,057.38 (8,458.15–9,744.28)	7,617.21 (7,005.02–8,321.32)
Philippines	NCD	23,203.57 (20,792.27–25,824.23)	23,428.71 (21,057.08–25,981.75)	24,156.38 (21,813.67–26,711.59)	25,027.91 (22,668.93–27,628.38)	24,568.61 (22,226.41–27,184.09)	24,279.27 (21,740.12–27,184.22)
Philippines	Injuries	3,401.62 (3,083.97–3,697.57)	2,988.16 (2,773.39–3,190.93)	3,222.85 (3,078.76–3,381.16)	3,145.73 (3,005.31–3,304.14)	2,845.79 (2,695.79–3,010.24)	2,657.92 (2,458.53–2,892.07)
Sri Lanka	All causes	37,008.16 (34,107.80–40,276.87)	33,787.47 (31,017.15–36,928.22)	32,003.76 (29,321.21–35,093.69)	29,240.34 (26,561.35–32,293.30)	27,617.65 (24,671.87–31,064.73)	23,617.12 (19,922.99–28,024.76)
Sri Lanka	Group I	9,728.57 (9,081.31–10,495.71)	6,318.72 (5,795.35–6,952.08)	5,022.66 (4,590.74–5,584.84)	4,579.27 (4,189.84–5,059.20)	3,443.17 (3,068.38–3,931.01)	2,802.48 (2,373.68–3,287.99)
Sri Lanka	NCD	20,090.81 (17,877.40–22,511.13)	20,346.07 (18,101.59–22,772.04)	20,898.40 (18,749.32–23,339.84)	20,282.75 (18,101.98–22,807.82)	18,846.50 (16,597.41–21,338.01)	17,569.19 (14,703.51–20,940.33)
Sri Lanka	Injuries	7,188.79 (6,733.35–7,664.91)	7,122.68 (6,868.40–7,372.10)	6,082.70 (5,574.93–6,569.84)	4,378.33 (4,188.42–4,587.97)	5,327.98 (3,970.43–6,713.94)	3,245.46 (2,634.03–3,949.65)

Appendix Table 4. Rate of DALYs per 100,000 individuals for all causes and level 1 cause groups by location for 1990 - 2015, both sexes combined.

	Cause	1990	1995	2000	2005	2010	2015
Seychelles	All causes	37,104.38 (34,281.02–40,139.73)	35,659.39 (32,750.56–38,629.67)	33,720.29 (30,896.11–36,877.42)	31,897.40 (29,115.74–34,898.88)	29,803.42 (27,081.54–33,001.33)	28,249.88 (25,207.37–31,857.79)
Seychelles	Group I	5,984.53 (5,430.12–6,562.86)	5,025.54 (4,585.97–5,588.21)	4,557.36 (4,160.91–5,117.99)	4,442.87 (4,066.46–4,912.31)	4,148.19 (3,709.67–4,648.59)	3,878.67 (3,372.05–4,424.24)
Seychelles	NCD	27,422.85 (25,019.27–29,950.59)	26,720.70 (24,345.25–29,330.62)	25,408.37 (23,068.89–28,065.88)	24,061.04 (21,644.38–26,647.62)	22,438.98 (20,093.77–25,051.71)	21,658.16 (18,950.90–24,522.36)
Seychelles	Injuries	3,697.00 (3,426.46–3,996.42)	3,913.15 (3,613.95–4,196.66)	3,754.57 (3,492.42–4,005.39)	3,393.49 (3,100.91–3,652.64)	3,216.24 (2,936.22–3,538.48)	2,713.04 (2,397.86–3,063.10)
Thailand	All causes	35,087.73 (31,917.85–38,360.17)	35,725.61 (32,664.89–39,290.74)	35,030.80 (32,022.77–38,605.17)	30,850.57 (28,001.05–34,041.82)	28,466.93 (25,611.19–31,453.20)	27,364.77 (23,807.86–30,925.59)
Thailand	Group I	8,093.53 (7,355.78–9,013.09)	6,562.68 (5,820.19–7,455.87)	6,387.46 (5,859.14–6,991.99)	5,206.54 (4,616.69–5,893.10)	4,616.69 (3,970.28–5,181.02)	4,149.75 (3,566.59–4,687.77)
Thailand	NCD	22,443.53 (20,053.53–25,019.08)	23,604.44 (21,148.00–26,435.45)	23,199.11 (20,832.69–25,998.73)	20,552.00 (18,078.74–23,168.52)	19,618.06 (17,076.71–22,143.37)	19,489.43 (16,689.75–22,328.05)
Thailand	Injuries	4,550.67 (4,243.78–4,870.95)	5,558.49 (5,166.68–5,985.05)	5,444.24 (4,966.00–5,920.26)	5,092.02 (4,690.31–5,520.78)	4,232.17 (3,910.49–4,580.33)	3,725.59 (3,220.03–4,285.82)
Timor-Leste	All causes	64,406.66 (58,851.28–70,468.57)	55,749.83 (50,928.99–60,673.73)	58,711.99 (51,377.34–66,202.42)	42,421.38 (38,456.44–46,480.24)	35,805.24 (31,564.29–40,442.87)	32,438.03 (27,534.46–38,309.15)
Timor-Leste	Group I	33,214.05 (29,174.89–37,979.53)	26,322.91 (22,844.41–30,690.04)	23,182.37 (19,816.42–27,500.63)	16,122.40 (13,867.95–19,276.96)	11,886.68 (9,696.01–14,740.54)	9,418.12 (7,264.95–12,448.85)
Timor-Leste	NCD	26,379.41 (22,501.30–30,879.62)	25,047.26 (21,633.64–28,791.01)	23,593.17 (19,867.63–27,216.71)	22,564.19 (19,332.07–25,718.41)	20,627.65 (17,422.18–23,831.46)	20,088.86 (16,741.93–23,443.35)
Timor-Leste	Injuries	4,813.21 (3,054.87–6,508.62)	4,379.66 (2,915.82–5,665.50)	11,936.45 (6,023.18–17,918.60)	3,734.79 (2,749.39–4,535.82)	3,290.90 (2,502.31–4,034.03)	2,931.06 (2,300.09–3,723.51)
Vietnam	All causes	38,542.12 (34,690.99–43,000.64)	35,832.63 (31,324.60–40,701.89)	32,918.83 (28,881.30–36,966.48)	30,504.15 (25,879.82–35,042.32)	27,989.67 (23,504.70–32,918.14)	26,348.41 (22,090.69–30,787.94)
Vietnam	Group I	12,095.33 (10,444.88–14,296.50)	9,815.16 (8,373.27–11,606.15)	7,628.65 (6,529.21–8,978.49)	6,192.80 (5,256.75–7,601.48)	4,814.96 (4,055.85–5,992.02)	3,863.22 (3,197.46–4,939.49)
Vietnam	NCD	22,214.54 (19,457.29–25,416.82)	22,074.13 (18,555.62–25,519.08)	21,603.35 (18,674.58–24,407.95)	20,808.74 (17,302.51–24,248.11)	19,915.46 (16,549.78–23,612.34)	19,465.38 (16,085.34–22,889.83)
Vietnam	Injuries	4,232.25 (3,243.62–5,172.85)	3,943.34 (3,129.91–4,764.26)	3,686.83 (3,030.43–4,337.00)	3,502.60 (2,774.28–4,275.73)	3,259.24 (2,538.96–4,199.54)	3,019.80 (2,327.39–3,825.74)
Oceania	All causes	65,486.86 (55,151.41–77,623.95)	63,720.12 (52,787.13–76,047.72)	62,618.54 (51,384.59–74,978.21)	61,861.89 (50,149.58–75,577.71)	59,022.30 (47,633.99–73,901.40)	54,772.34 (44,610.87–68,658.11)
Oceania	Group I	20,715.45 (18,051.73–23,854.04)	19,160.02 (16,503.36–22,383.18)	17,784.45 (14,765.40–20,986.94)	16,922.31 (13,763.18–20,500.73)	14,941.14 (12,062.12–19,015.50)	12,747.62 (10,229.20–16,236.43)
Oceania	NCD	38,963.58 (31,905.79–47,222.74)	38,905.62 (31,707.72–47,280.66)	39,204.76 (31,483.34–47,359.28)	39,376.60 (31,510.36–48,223.85)	38,795.95 (31,313.20–49,119.43)	37,116.02 (30,109.59–46,583.91)
Oceania	Injuries	5,807.83 (4,525.99–7,504.01)	5,654.48 (4,338.51–7,419.46)	5,629.32 (4,241.43–7,360.38)	5,562.98 (4,113.96–7,362.98)	5,285.20 (3,931.52–7,243.44)	4,908.70 (3,697.49–6,630.60)
American Samoa	All causes	35,900.40 (32,255.74–39,886.81)	35,211.94 (31,253.97–39,474.89)	33,311.78 (29,677.24–37,061.93)	32,673.74 (29,181.28–36,512.63)	31,264.96 (27,691.41–35,336.20)	30,979.19 (26,542.57–35,263.14)
American Samoa	Group I	4,984.38 (4,471.96–5,608.36)	4,527.43 (4,061.26–5,064.99)	3,937.55 (3,513.68–4,410.15)	3,486.38 (3,118.78–3,931.01)	3,047.10 (2,683.13–3,455.40)	2,850.31 (2,437.96–3,302.46)
American Samoa	NCD	27,312.20 (24,379.16–30,540.77)	27,274.98 (24,126.24–30,631.65)	26,296.86 (23,239.72–29,479.06)	26,174.13 (23,220.91–29,477.29)	25,457.04 (22,427.41–28,902.44)	25,514.14 (21,765.62–29,213.33)
American Samoa	Injuries	3,603.82 (3,177.06–4,077.18)	3,409.52 (2,986.18–3,881.96)	3,077.37 (2,784.21–3,419.54)	3,013.23 (2,718.89–3,328.43)	2,760.83 (2,477.88–3,088.46)	2,614.75 (2,233.93–3,009.45)
Federated States of Micronesia	All causes	48,685.85 (36,542.36–62,245.62)	45,989.27 (34,998.21–59,412.93)	43,122.48 (33,288.60–56,876.86)	40,812.30 (31,270.41–55,876.41)	39,423.65 (30,268.13–55,170.29)	37,933.50 (29,075.54–53,827.86)
Federated States of Micronesia	Group I	10,030.50 (8,125.00–12,271.42)	8,629.15 (6,845.37–11,228.75)	7,029.94 (5,431.04–9,819.34)	5,651.56 (4,346.78–7,730.25)	4,884.39 (3,697.75–6,548.30)	4,316.28 (3,332.49–5,790.41)
Federated States of Micronesia	NCD	34,260.00 (24,801.75–44,510.78)	33,273.91 (24,768.08–43,946.93)	32,304.36 (24,633.16–43,627.87)	31,588.60 (23,978.69–43,911.86)	31,185.31 (23,698.10–43,745.43)	30,417.32 (23,041.84–43,483.18)

Appendix Table 4. Rate of DALYs per 100,000 individuals for all causes and level 1 cause groups by location for 1990 - 2015, both sexes combined.

	Cause	1990	1995	2000	2005	2010	2015
Federated States of Micronesia	Injuries	4,395.36 (2,968.70–5,982.96)	4,086.21 (2,796.94–5,685.67)	3,788.18 (2,641.63–5,270.50)	3,572.14 (2,483.43–5,282.68)	3,353.95 (2,378.29–5,068.88)	3,199.91 (2,269.33–4,881.86)
Fiji	All causes	47,505.46 (42,048.75–53,874.62)	49,001.41 (43,665.72–54,692.65)	50,115.16 (45,162.40–55,152.61)	48,408.04 (43,918.55–53,128.71)	47,420.68 (42,553.06–52,568.31)	45,705.33 (40,041.85–51,949.32)
Fiji	Group I	8,658.46 (7,224.77–10,282.99)	8,406.44 (7,137.03–9,964.27)	8,636.87 (7,393.10–10,074.23)	8,154.18 (6,972.39–9,530.14)	7,697.85 (6,381.32–9,360.39)	7,216.04 (5,722.93–9,272.80)
Fiji	NCD	35,587.36 (31,449.23–40,682.71)	37,233.05 (33,183.25–41,692.38)	37,831.46 (34,144.15–41,728.22)	36,491.61 (32,900.60–40,080.89)	36,345.46 (32,701.27–40,429.50)	35,065.45 (30,742.06–40,015.30)
Fiji	Injuries	3,259.64 (2,768.33–3,796.25)	3,361.93 (2,950.97–3,797.72)	3,646.84 (3,278.34–4,015.43)	3,762.26 (3,369.77–4,173.77)	3,377.36 (2,986.80–3,829.64)	3,423.83 (2,906.84–4,009.32)
Guam	All causes	30,579.13 (27,554.47–34,010.76)	30,053.87 (26,952.08–33,572.28)	29,036.46 (25,921.84–32,581.34)	30,484.57 (27,114.97–33,998.86)	33,433.30 (29,683.61–37,291.31)	33,196.09 (29,155.80–37,570.05)
Guam	Group I	3,730.81 (3,258.55–4,262.38)	3,376.04 (2,988.89–3,831.59)	3,250.47 (2,881.13–3,704.06)	3,711.75 (3,138.39–4,546.20)	4,158.07 (3,471.61–5,252.30)	3,973.63 (3,220.16–4,914.35)
Guam	NCD	23,707.85 (21,222.15–26,430.96)	23,488.33 (20,835.45–26,443.91)	22,994.82 (20,390.36–25,859.54)	23,848.40 (21,125.86–26,585.31)	26,030.03 (23,120.95–29,068.78)	25,972.72 (22,728.95–29,478.71)
Guam	Injuries	3,140.47 (2,803.46–3,475.32)	3,189.50 (2,851.54–3,537.53)	2,791.17 (2,483.87–3,071.46)	2,924.42 (2,624.24–3,229.44)	3,245.21 (2,943.31–3,589.91)	3,249.74 (2,770.26–3,736.46)
Kiribati	All causes	66,925.07 (60,700.43–73,162.98)	64,417.08 (58,893.26–69,899.25)	62,034.65 (57,007.89–67,543.24)	59,078.65 (53,306.69–64,918.15)	55,879.78 (49,397.53–63,333.89)	52,844.25 (46,157.12–59,991.73)
Kiribati	Group I	24,598.54 (21,479.48–27,787.28)	21,743.16 (19,517.88–24,197.13)	18,679.77 (16,764.03–21,147.17)	16,987.78 (14,763.44–19,586.57)	14,739.19 (12,026.23–17,998.50)	12,704.60 (9,937.44–16,532.15)
Kiribati	NCD	37,964.10 (33,793.11–42,012.86)	38,294.09 (34,722.44–42,088.34)	38,919.41 (35,179.96–42,946.78)	37,631.99 (33,709.73–41,740.18)	36,738.80 (32,462.61–41,240.78)	35,908.46 (31,313.16–40,476.65)
Kiribati	Injuries	4,362.43 (3,719.96–4,970.86)	4,379.83 (3,838.11–4,940.66)	4,435.48 (3,801.12–4,991.14)	4,458.89 (3,627.97–5,157.18)	4,401.80 (3,454.77–5,265.99)	4,231.19 (3,227.81–5,128.32)
Marshall Islands	All causes	49,287.30 (44,309.89–54,779.14)	47,853.08 (43,107.10–52,660.54)	49,375.05 (44,131.06–54,851.11)	49,316.53 (43,729.84–54,442.85)	46,674.92 (41,054.52–52,796.95)	43,989.74 (38,551.29–49,909.20)
Marshall Islands	Group I	11,419.98 (9,483.03–13,649.19)	10,065.58 (8,328.67–13,593.41)	10,161.20 (8,067.86–14,590.08)	8,997.14 (7,368.81–11,476.86)	7,488.48 (5,824.58–9,864.33)	6,421.16 (4,939.98–8,327.95)
Marshall Islands	NCD	33,458.99 (29,853.70–37,060.27)	33,698.70 (30,032.55–37,375.78)	34,957.14 (30,595.65–39,049.41)	36,080.53 (31,620.26–40,148.86)	35,290.55 (30,794.25–39,597.53)	34,003.42 (29,478.08–38,499.43)
Marshall Islands	Injuries	4,408.32 (3,754.46–5,048.38)	4,088.80 (3,316.13–4,678.44)	4,256.70 (3,385.95–5,030.84)	4,238.86 (3,443.83–5,000.13)	3,895.90 (3,233.99–4,565.37)	3,565.16 (2,978.82–4,192.94)
Northern Mariana Islands	All causes	31,722.41 (28,081.58–35,841.60)	28,431.21 (25,043.82–31,903.54)	26,273.94 (23,191.54–29,503.13)	25,605.46 (22,811.70–28,888.55)	24,908.44 (22,084.81–28,146.17)	25,232.01 (22,262.59–28,737.80)
Northern Mariana Islands	Group I	4,497.64 (3,457.04–6,115.79)	3,504.97 (2,835.14–4,417.56)	2,920.68 (2,513.95–3,402.32)	2,613.34 (2,265.00–3,041.33)	2,291.78 (1,968.54–2,675.18)	2,366.65 (1,959.87–2,860.49)
Northern Mariana Islands	NCD	23,951.31 (21,236.45–27,210.35)	22,198.12 (19,468.72–25,110.92)	20,934.50 (18,335.78–23,599.36)	20,520.82 (18,048.09–23,220.01)	20,313.16 (17,820.17–23,099.00)	20,562.97 (18,064.37–23,457.92)
Northern Mariana Islands	Injuries	3,273.46 (2,807.20–3,776.25)	2,728.12 (2,417.13–3,062.31)	2,418.77 (2,190.32–2,676.50)	2,471.30 (2,246.75–2,711.32)	2,303.50 (2,062.79–2,560.43)	2,302.39 (2,012.35–2,649.65)
Papua New Guinea	All causes	74,212.13 (59,221.88–92,221.35)	71,511.43 (55,686.16–89,189.03)	69,689.71 (53,259.01–86,897.37)	68,885.60 (52,452.92–88,276.39)	65,097.43 (49,112.79–85,961.24)	59,781.65 (45,733.54–80,168.43)
Papua New Guinea	Group I	25,819.56 (21,948.02–30,294.18)	23,634.10 (19,862.05–28,273.52)	21,617.63 (17,367.04–26,182.79)	20,497.84 (16,154.55–25,436.61)	17,952.89 (13,985.96–23,353.81)	15,166.71 (11,851.63–19,931.81)
Papua New Guinea	NCD	41,681.69 (31,404.13–53,309.67)	41,394.67 (30,990.00–53,558.51)	41,697.42 (30,724.35–53,400.89)	42,133.84 (30,704.46–54,932.96)	41,224.86 (30,591.54–55,885.38)	39,198.87 (29,408.17–52,764.17)
Papua New Guinea	Injuries	6,710.89 (4,798.77–9,177.20)	6,482.66 (4,556.75–9,059.45)	6,374.67 (4,435.68–8,865.29)	6,253.92 (4,264.24–8,720.81)	5,919.69 (4,077.07–8,663.25)	5,416.06 (3,819.92–7,868.09)
Samoa	All causes	37,678.10 (32,738.02–43,768.54)	35,909.39 (30,986.97–41,721.32)	34,447.83 (29,824.64–39,785.85)	32,637.26 (28,221.51–37,328.89)	31,208.24 (27,202.98–35,476.55)	30,274.61 (25,633.84–34,806.86)
Samoa	Group I	6,213.01 (4,840.82–8,431.54)	5,554.82 (4,287.83–7,828.06)	4,981.94 (3,727.78–7,244.49)	4,382.12 (3,386.60–6,270.93)	3,843.82 (3,022.19–5,212.39)	3,496.53 (2,738.70–4,632.86)

Appendix Table 4. Rate of DALYs per 100,000 individuals for all causes and level 1 cause groups by location for 1990 - 2015, both sexes combined.							
	Cause	1990	1995	2000	2005	2010	2015
Samoa	NCD	28,002.04 (23,937.09–32,563.46)	27,156.51 (23,089.05–31,638.95)	26,440.98 (22,512.39–30,518.10)	25,447.03 (21,794.77–29,128.87)	24,693.68 (21,277.14–28,204.01)	24,246.51 (20,375.58–27,999.31)
Samoa	Injuries	3,463.06 (2,727.54–4,255.14)	3,198.06 (2,441.73–4,026.19)	3,024.91 (2,354.28–3,753.01)	2,808.11 (2,268.11–3,360.52)	2,670.74 (2,213.45–3,180.70)	2,531.56 (2,056.87–3,066.83)
Solomon Islands	All causes	60,683.70 (46,808.48–78,074.46)	57,542.89 (44,313.76–74,329.57)	55,899.01 (42,955.29–72,262.08)	55,705.89 (41,753.53–72,258.07)	53,130.60 (39,226.61–70,897.91)	49,876.33 (36,894.95–68,259.83)
Solomon Islands	Group I	14,926.55 (11,811.13–18,546.66)	12,832.85 (10,098.62–16,458.72)	11,236.70 (8,796.15–14,755.78)	10,579.95 (8,249.86–13,634.72)	8,952.62 (6,903.25–11,584.01)	7,462.18 (5,729.84–9,745.00)
Solomon Islands	NCD	40,330.26 (30,195.38–53,227.44)	39,600.66 (29,557.94–52,205.10)	39,561.80 (29,312.64–52,116.42)	40,147.87 (29,469.47–53,093.78)	39,448.86 (28,707.92–53,748.99)	38,002.55 (27,575.84–52,655.28)
Solomon Islands	Injuries	5,426.90 (3,813.63–7,455.83)	5,109.37 (3,544.24–7,065.76)	5,100.51 (3,456.66–7,098.79)	4,978.08 (3,351.26–7,068.40)	4,729.11 (3,280.71–6,747.79)	4,411.60 (3,107.29–6,442.57)
Tonga	All causes	38,692.22 (33,847.94–44,024.12)	37,300.84 (33,460.63–41,593.10)	37,596.92 (33,843.52–41,711.61)	36,619.39 (32,631.97–40,772.80)	35,309.31 (30,733.84–39,933.73)	33,716.44 (28,994.24–38,841.09)
Tonga	Group I	7,714.63 (6,159.96–10,160.26)	6,780.40 (5,533.02–8,246.22)	6,729.08 (5,493.99–8,218.91)	6,085.86 (4,959.28–7,420.50)	5,683.35 (4,629.46–7,040.39)	5,076.94 (4,108.25–6,361.50)
Tonga	NCD	26,901.80 (23,550.25–30,595.14)	26,534.52 (23,689.81–29,703.31)	26,957.94 (24,274.57–29,888.27)	26,732.82 (23,791.91–29,842.26)	26,047.60 (22,763.93–29,455.44)	25,301.37 (21,786.49–29,194.51)
Tonga	Injuries	4,075.79 (3,434.99–4,739.74)	3,985.91 (3,475.79–4,533.22)	3,909.90 (3,491.76–4,371.66)	3,800.70 (3,381.70–4,316.98)	3,578.36 (3,097.07–4,151.26)	3,338.13 (2,793.51–3,993.68)
Vanuatu	All causes	54,734.10 (42,691.99–70,428.72)	53,627.82 (40,578.52–70,844.50)	53,187.38 (39,656.61–69,184.15)	52,581.37 (39,701.09–68,482.81)	49,681.50 (37,774.31–67,527.37)	47,158.75 (36,541.04–62,921.33)
Vanuatu	Group I	12,473.19 (10,036.23–15,555.76)	11,491.90 (9,085.92–14,871.49)	10,987.14 (8,793.97–15,083.27)	10,704.18 (8,560.08–13,559.48)	9,081.93 (7,302.23–11,963.09)	7,773.37 (6,092.44–9,981.61)
Vanuatu	NCD	37,559.36 (28,405.00–49,597.75)	37,467.26 (27,668.04–50,947.23)	37,346.81 (26,708.78–49,681.18)	37,164.32 (27,018.09–49,838.10)	36,156.30 (26,462.97–49,954.38)	35,008.11 (26,229.06–47,434.89)
Vanuatu	Injuries	4,701.55 (3,284.93–6,667.27)	4,668.65 (3,034.16–6,674.18)	4,853.43 (3,225.13–6,842.24)	4,712.88 (3,224.87–6,588.43)	4,443.26 (3,086.56–6,337.73)	4,377.27 (3,259.02–5,873.41)
North Africa and Middle East	All causes	47,077.34 (43,748.66–50,743.07)	43,326.26 (39,987.02–46,850.21)	39,453.86 (36,098.58–42,960.92)	36,879.74 (33,634.19–40,412.17)	34,117.58 (30,836.97–37,496.91)	33,421.27 (29,866.62–37,002.48)
North Africa and Middle East	Group I	12,965.67 (11,755.24–13,943.76)	11,039.88 (10,037.15–11,876.45)	9,232.05 (8,428.36–9,965.82)	7,636.86 (6,977.80–8,311.52)	6,308.45 (5,745.52–6,886.55)	5,273.78 (4,772.90–5,837.59)
North Africa and Middle East	NCD	28,128.33 (25,418.19–30,970.79)	27,338.57 (24,630.80–30,134.51)	25,764.75 (23,072.78–28,509.13)	24,962.52 (22,256.70–27,826.27)	23,985.77 (21,223.60–26,759.75)	23,244.04 (20,547.98–25,929.49)
North Africa and Middle East	Injuries	5,983.35 (5,260.20–6,623.62)	4,947.81 (4,452.01–5,389.36)	4,457.06 (4,052.61–4,847.10)	4,280.36 (3,900.47–4,658.65)	3,823.36 (3,498.77–4,157.73)	4,903.45 (4,098.11–5,681.38)
Afghanistan	All causes	96,535.26 (84,778.31–109,340.47)	97,860.13 (85,710.05–111,186.40)	95,441.30 (83,832.19–107,905.59)	88,665.62 (77,346.68–101,012.85)	83,014.54 (72,769.27–95,522.86)	81,984.51 (70,997.88–94,545.19)
Afghanistan	Group I	33,184.13 (27,620.61–39,177.33)	31,435.10 (26,573.88–36,735.97)	28,699.03 (24,186.67–33,537.06)	23,444.42 (19,979.41–26,649.18)	19,859.40 (16,782.53–23,036.37)	16,363.68 (13,721.32–19,406.95)
Afghanistan	NCD	47,428.34 (38,574.57–56,965.45)	49,119.78 (40,314.59–58,688.99)	50,116.71 (41,627.68–59,032.91)	49,896.10 (41,609.13–58,227.57)	48,253.26 (40,395.66–56,544.04)	46,547.42 (38,756.33–54,913.44)
Afghanistan	Injuries	15,922.79 (12,413.73–19,391.28)	17,305.25 (13,535.66–20,888.72)	16,625.57 (13,328.16–20,066.02)	15,325.10 (12,708.85–18,501.61)	14,901.87 (12,396.31–17,882.38)	19,073.42 (14,199.44–24,168.78)
Algeria	All causes	37,590.27 (34,112.84–41,541.11)	34,916.59 (31,603.70–38,653.42)	31,731.60 (28,547.36–35,400.32)	29,217.46 (26,009.40–32,898.54)	27,319.87 (24,129.87–30,755.14)	26,554.64 (23,276.59–30,003.26)
Algeria	Group I	9,448.00 (8,483.49–10,436.17)	7,777.63 (6,991.42–8,640.18)	6,497.11 (5,852.13–7,157.52)	5,561.67 (4,900.46–6,260.10)	4,898.99 (4,261.04–5,592.62)	4,372.19 (3,714.49–5,156.99)
Algeria	NCD	23,937.83 (21,288.99–27,165.84)	22,803.72 (20,108.65–25,877.93)	21,761.50 (19,128.74–24,693.40)	20,778.76 (18,120.70–23,658.56)	19,850.22 (17,231.97–22,614.00)	19,720.16 (17,058.40–22,542.43)
Algeria	Injuries	4,204.45 (3,600.24–4,744.76)	4,335.24 (3,697.53–5,038.16)	3,472.98 (3,124.52–3,820.84)	2,877.03 (2,576.91–3,185.50)	2,570.66 (2,310.39–2,841.99)	2,462.29 (2,181.77–2,767.21)
Bahrain	All causes	33,878.03 (30,088.90–37,983.56)	32,106.82 (28,436.28–36,017.59)	30,776.71 (27,126.81–34,394.04)	26,664.70 (23,512.60–30,289.89)	22,967.32 (19,750.66–26,534.65)	22,396.32 (18,913.75–26,077.69)

Appendix Table 4. Rate of DALYs per 100,000 individuals for all causes and level 1 cause groups by location for 1990 - 2015, both sexes combined.

	Cause	1990	1995	2000	2005	2010	2015
Bahrain	Group I	4,139.35 (3,710.70–4,677.78)	3,505.23 (3,126.17–4,016.84)	2,922.21 (2,576.77–3,359.02)	2,546.13 (2,219.51–2,953.41)	2,123.83 (1,796.78–2,501.41)	1,981.53 (1,653.59–2,369.06)
Bahrain	NCD	26,273.43 (23,180.80–29,657.30)	25,111.65 (22,204.07–28,243.38)	23,820.92 (20,937.12–26,668.20)	21,651.01 (19,043.03–24,593.42)	19,011.17 (16,265.14–21,972.10)	18,663.04 (15,761.94–21,699.51)
Bahrain	Injuries	3,465.24 (3,038.70–3,930.68)	3,489.94 (3,064.06–3,965.51)	4,033.59 (3,251.27–4,790.75)	2,467.56 (2,153.81–2,816.78)	1,832.32 (1,601.72–2,112.72)	1,751.75 (1,461.76–2,074.72)
Egypt	All causes	48,468.89 (45,001.47–52,087.82)	43,133.30 (39,790.57–46,552.74)	37,933.53 (34,809.16–41,338.37)	36,899.68 (33,738.60–40,342.04)	36,778.39 (33,658.37–40,267.33)	34,129.71 (30,588.53–37,883.08)
Egypt	Group I	14,483.44 (12,775.75–15,647.90)	10,720.83 (9,743.48–11,674.50)	7,486.58 (6,829.44–8,227.03)	6,089.39 (5,435.23–6,863.10)	5,227.02 (4,616.09–5,919.18)	4,263.91 (3,529.20–5,123.97)
Egypt	NCD	31,395.11 (28,631.84–34,093.04)	29,904.28 (27,304.87–32,599.63)	28,020.57 (25,482.65–30,757.43)	28,571.41 (26,030.60–31,356.44)	29,349.94 (26,821.15–32,180.10)	27,666.34 (24,882.81–30,590.09)
Egypt	Injuries	2,590.33 (2,278.06–3,055.04)	2,508.19 (2,271.42–2,796.07)	2,426.38 (2,230.89–2,625.43)	2,238.88 (2,056.79–2,429.69)	2,201.43 (2,031.35–2,390.28)	2,199.46 (1,981.10–2,419.07)
Iran	All causes	47,157.92 (42,165.77–52,025.12)	38,376.55 (34,151.55–42,786.73)	35,267.77 (31,599.36–39,321.89)	33,432.45 (29,001.06–38,096.00)	30,148.40 (25,822.07–34,899.06)	28,442.76 (24,284.48–32,881.78)
Iran	Group I	8,827.13 (7,292.20–10,818.73)	6,122.15 (5,088.20–7,608.99)	4,702.71 (3,878.39–5,767.59)	3,893.64 (3,191.72–4,718.14)	3,011.85 (2,478.64–3,573.09)	2,527.87 (2,054.67–3,081.37)
Iran	NCD	26,708.81 (23,293.39–29,980.52)	26,044.10 (22,609.62–29,433.53)	24,983.83 (21,802.18–28,203.28)	24,064.28 (20,702.67–27,647.36)	22,647.65 (19,160.75–26,362.01)	22,053.07 (18,561.34–25,636.29)
Iran	Injuries	11,621.98 (8,872.60–14,278.39)	6,210.30 (5,481.75–7,014.93)	5,581.23 (5,061.74–6,184.15)	5,474.53 (4,741.60–6,326.79)	4,488.90 (3,751.48–5,369.68)	3,861.82 (3,211.05–4,643.20)
Iraq	All causes	47,543.48 (42,074.24–53,221.23)	48,527.48 (43,100.66–54,395.70)	47,180.82 (41,702.53–53,210.94)	47,311.62 (41,479.22–54,560.79)	43,296.05 (37,095.47–50,156.24)	43,363.92 (36,875.55–50,311.17)
Iraq	Group I	9,612.07 (8,479.26–10,765.20)	9,217.23 (8,207.76–10,385.74)	8,014.58 (7,131.65–8,897.10)	6,868.29 (6,088.25–7,651.64)	5,883.38 (5,151.18–6,633.25)	4,830.20 (4,112.58–5,586.78)
Iraq	NCD	30,792.96 (26,407.66–35,165.08)	32,152.65 (28,029.56–36,757.12)	32,582.37 (28,558.01–37,325.58)	32,007.37 (27,896.42–37,400.40)	30,899.16 (26,445.24–35,975.93)	29,422.96 (24,913.23–34,695.20)
Iraq	Injuries	7,138.45 (6,050.00–8,336.54)	7,157.60 (6,104.25–8,299.05)	6,583.86 (5,553.91–7,637.76)	8,435.95 (6,792.58–10,132.01)	6,513.50 (5,295.85–7,717.44)	9,110.75 (6,494.89–11,545.38)
Jordan	All causes	34,725.45 (30,875.53–38,869.54)	32,749.80 (29,005.15–37,137.06)	31,143.70 (27,665.26–35,064.32)	29,103.51 (25,843.82–32,780.96)	24,367.20 (21,308.20–27,823.22)	23,585.85 (20,432.93–27,123.11)
Jordan	Group I	5,335.73 (4,800.60–5,936.83)	4,496.93 (4,099.84–4,976.96)	3,984.83 (3,615.92–4,412.26)	3,502.83 (3,128.78–3,956.95)	2,919.90 (2,570.20–3,337.44)	2,649.41 (2,246.96–3,096.58)
Jordan	NCD	25,390.82 (22,095.35–28,905.55)	24,760.86 (21,520.27–28,455.97)	24,047.05 (21,103.91–27,337.79)	22,730.66 (19,981.09–25,755.52)	19,418.49 (16,822.11–22,348.86)	18,879.04 (16,112.21–21,769.15)
Jordan	Injuries	3,998.89 (3,412.82–4,569.42)	3,492.01 (3,027.02–4,013.29)	3,111.81 (2,772.14–3,524.68)	2,870.02 (2,629.54–3,128.26)	2,028.81 (1,856.99–2,227.76)	2,057.40 (1,828.90–2,300.13)
Kuwait	All causes	25,281.64 (22,149.36–28,894.32)	25,656.60 (22,849.34–28,805.75)	23,781.82 (20,905.86–26,954.45)	23,333.53 (20,520.21–26,484.19)	22,137.55 (19,353.84–25,286.62)	19,951.13 (16,962.20–23,313.18)
Kuwait	Group I	2,810.40 (2,473.86–3,212.83)	2,744.38 (2,459.40–3,108.34)	2,297.35 (2,038.65–2,634.33)	2,177.67 (1,918.03–2,501.47)	2,189.14 (1,923.39–2,523.67)	1,911.08 (1,619.64–2,262.22)
Kuwait	NCD	17,938.28 (15,552.25–20,477.57)	19,914.90 (17,567.93–22,487.01)	18,887.54 (16,477.63–21,500.67)	18,679.45 (16,225.50–21,258.76)	17,932.56 (15,536.20–20,608.33)	16,336.11 (13,784.37–19,113.70)
Kuwait	Injuries	4,532.96 (3,020.99–6,190.08)	2,997.32 (2,729.73–3,328.23)	2,596.94 (2,342.56–2,896.58)	2,476.41 (2,264.94–2,731.78)	2,015.85 (1,806.62–2,254.90)	1,703.94 (1,466.84–2,000.06)
Lebanon	All causes	42,232.80 (37,326.13–47,864.22)	33,740.04 (29,461.06–38,374.12)	29,500.11 (25,412.77–33,986.82)	26,476.51 (22,386.10–30,580.64)	24,657.50 (20,561.39–28,932.12)	23,400.33 (19,402.95–27,729.78)
Lebanon	Group I	5,312.23 (4,559.66–6,365.69)	4,547.47 (3,797.15–5,733.71)	3,761.33 (3,076.67–4,915.77)	2,816.26 (2,265.91–3,584.65)	2,240.62 (1,798.42–2,833.53)	1,957.99 (1,553.91–2,502.79)
Lebanon	NCD	25,756.29 (22,399.64–29,725.61)	23,120.10 (20,257.70–26,460.01)	20,664.30 (17,729.43–23,695.23)	19,480.55 (16,452.61–22,611.40)	18,938.75 (15,699.64–22,298.13)	18,378.53 (15,063.25–21,756.85)
Lebanon	Injuries	11,164.28 (9,091.10–13,894.70)	6,072.48 (4,412.97–8,135.87)	5,074.48 (3,613.00–6,894.52)	4,179.71 (2,930.81–5,749.27)	3,478.13 (2,515.08–4,805.56)	3,063.81 (2,211.16–4,197.35)

Appendix Table 4. Rate of DALYs per 100,000 individuals for all causes and level 1 cause groups by location for 1990 - 2015, both sexes combined.

	Cause	1990	1995	2000	2005	2010	2015
Libya	All causes	31,565.50 (28,069.52–35,367.80)	29,394.88 (26,253.90–32,877.14)	28,777.08 (25,565.13–32,297.32)	27,755.17 (24,637.21–31,192.84)	26,735.35 (23,544.34–30,166.85)	30,041.90 (26,082.28–34,188.96)
Libya	Group I	5,536.71 (4,917.44–6,343.51)	4,587.99 (4,019.51–5,321.23)	4,067.30 (3,478.10–4,779.37)	3,668.27 (3,069.75–4,464.69)	3,157.12 (2,593.36–3,927.62)	2,866.09 (2,331.67–3,531.60)
Libya	NCD	22,339.06 (19,556.94–25,360.69)	21,471.90 (18,780.80–24,458.51)	21,434.35 (18,773.53–24,262.61)	21,155.07 (18,610.12–23,795.85)	20,783.26 (18,130.49–23,597.71)	21,253.41 (18,265.44–24,285.81)
Libya	Injuries	3,689.72 (3,187.69–4,178.77)	3,334.99 (2,906.44–3,774.71)	3,275.43 (2,866.98–3,660.99)	2,931.82 (2,544.79–3,270.33)	2,794.97 (2,463.24–3,140.09)	5,922.40 (3,971.65–7,847.69)
Morocco	All causes	43,427.26 (39,347.13–47,790.45)	39,573.12 (35,575.81–43,878.49)	35,640.47 (31,475.82–39,979.12)	33,169.70 (28,404.92–37,893.56)	31,280.26 (26,740.89–36,450.89)	29,793.35 (25,165.98–34,821.23)
Morocco	Group I	13,012.84 (11,732.78–14,062.80)	10,142.30 (9,183.13–11,009.26)	8,087.13 (7,249.31–8,918.86)	6,786.33 (5,927.55–7,623.10)	5,664.09 (4,853.01–6,504.89)	4,773.98 (3,995.90–5,590.86)
Morocco	NCD	25,958.42 (22,818.83–29,278.65)	25,319.84 (22,003.44–28,840.73)	24,059.65 (20,759.71–27,493.47)	23,285.04 (19,465.34–26,875.30)	22,643.44 (18,882.39–26,757.38)	22,149.26 (18,257.28–26,276.63)
Morocco	Injuries	4,456.01 (3,761.19–5,079.31)	4,110.98 (3,564.77–4,670.97)	3,493.70 (3,042.31–4,008.72)	3,098.33 (2,638.43–3,661.26)	2,972.73 (2,472.95–3,615.07)	2,870.10 (2,327.36–3,561.62)
Palestine	All causes	35,080.88 (30,554.65–40,065.21)	31,240.49 (27,528.11–35,374.38)	30,259.75 (26,738.70–33,883.15)	29,322.02 (26,054.35–32,783.66)	29,360.61 (25,609.35–33,444.98)	28,613.39 (24,485.05–33,067.37)
Palestine	Group I	6,199.55 (5,494.75–6,893.77)	4,822.52 (4,287.66–5,387.98)	4,256.90 (3,800.25–4,741.11)	4,062.71 (3,620.95–4,580.92)	3,765.27 (3,326.96–4,245.27)	3,382.16 (2,835.55–4,028.13)
Palestine	NCD	24,203.01 (20,470.21–28,509.73)	22,803.83 (19,664.09–26,267.87)	21,728.94 (19,014.09–24,567.40)	22,122.91 (19,357.68–24,965.10)	22,786.39 (19,588.75–26,262.47)	22,683.19 (19,152.54–26,454.43)
Palestine	Injuries	4,678.33 (3,552.65–5,881.18)	3,614.14 (2,700.82–4,524.33)	4,273.91 (3,110.64–5,473.38)	3,136.41 (2,427.96–3,884.78)	2,808.95 (2,200.73–3,467.27)	2,548.04 (1,996.08–3,163.55)
Oman	All causes	34,641.70 (29,355.43–40,155.71)	30,933.05 (26,296.32–35,810.05)	28,279.65 (24,421.20–32,244.84)	27,053.26 (23,679.75–30,607.69)	26,972.45 (23,700.45–30,532.01)	25,338.45 (21,516.13–29,120.71)
Oman	Group I	7,121.67 (6,017.62–8,332.47)	5,113.18 (4,401.63–5,939.35)	3,769.24 (3,287.71–4,343.39)	3,162.93 (2,734.67–3,645.55)	3,088.01 (2,700.47–3,507.39)	2,714.40 (2,302.69–3,146.06)
Oman	NCD	22,365.08 (18,680.02–26,451.08)	21,448.32 (17,936.51–25,304.32)	20,906.70 (17,698.54–24,250.18)	20,644.38 (17,854.46–23,613.05)	20,732.50 (17,917.87–23,691.64)	19,724.57 (16,530.89–22,937.34)
Oman	Injuries	5,154.95 (3,932.69–6,379.47)	4,371.55 (3,521.24–5,322.46)	3,603.72 (3,086.39–4,210.92)	3,245.95 (2,937.28–3,567.86)	3,151.94 (2,872.87–3,434.27)	2,899.48 (2,451.09–3,397.34)
Qatar	All causes	29,458.17 (26,125.43–32,963.41)	29,601.68 (26,147.28–33,610.10)	28,808.24 (25,450.58–32,420.73)	26,341.19 (22,824.08–29,947.16)	22,371.71 (19,335.73–25,870.73)	21,533.41 (18,047.40–25,539.42)
Qatar	Group I	3,258.74 (2,604.13–4,171.78)	2,945.94 (2,388.07–3,627.13)	2,583.77 (2,110.94–3,165.20)	2,205.00 (1,785.95–2,634.75)	1,909.98 (1,572.69–2,325.73)	1,676.14 (1,342.23–2,080.62)
Qatar	NCD	21,316.78 (18,560.22–24,109.82)	21,993.46 (19,215.46–25,033.64)	21,706.43 (19,019.92–24,589.41)	20,170.11 (17,328.30–23,295.66)	17,319.91 (14,789.51–20,173.01)	16,956.27 (14,170.34–20,171.52)
Qatar	Injuries	4,882.65 (4,238.83–5,536.88)	4,662.27 (4,101.38–5,309.63)	4,518.05 (3,935.93–5,085.93)	3,966.08 (3,485.79–4,498.05)	3,141.82 (2,735.60–3,587.45)	2,901.00 (2,372.15–3,520.05)
Saudi Arabia	All causes	31,249.00 (27,926.67–35,336.69)	28,220.69 (25,170.45–31,513.86)	25,719.49 (22,789.45–28,967.01)	23,990.00 (21,158.33–27,158.52)	22,566.05 (19,786.51–25,639.10)	21,463.66 (18,543.09–24,729.24)
Saudi Arabia	Group I	6,971.32 (5,916.59–7,785.70)	5,454.77 (4,734.30–6,104.04)	4,037.94 (3,541.73–4,505.43)	3,233.38 (2,810.73–3,635.71)	2,723.57 (2,338.60–3,083.90)	2,351.96 (1,986.78–2,686.94)
Saudi Arabia	NCD	19,148.57 (16,355.40–22,008.61)	18,699.60 (16,084.34–21,377.98)	18,173.36 (15,697.33–20,795.01)	17,651.09 (15,150.28–20,271.37)	17,089.94 (14,641.88–19,712.09)	16,627.83 (14,122.80–19,351.12)
Saudi Arabia	Injuries	5,129.11 (4,094.63–7,688.00)	4,066.31 (3,615.35–4,490.33)	3,508.19 (3,163.85–3,833.70)	3,105.53 (2,811.89–3,378.90)	2,752.54 (2,483.64–2,999.60)	2,483.87 (2,209.08–2,757.24)
Sudan	All causes	65,008.12 (58,587.15–71,736.56)	60,711.47 (53,989.18–67,685.26)	56,048.08 (48,992.79–63,977.83)	50,573.76 (43,597.32–57,902.07)	46,960.73 (39,866.78–53,760.19)	43,547.64 (36,653.12–51,346.62)
Sudan	Group I	25,393.24 (21,759.39–28,993.70)	22,702.93 (19,423.87–26,084.98)	19,684.77 (16,871.83–22,468.74)	16,151.72 (13,840.40–18,530.10)	13,543.17 (11,519.17–15,718.27)	11,341.78 (9,329.81–13,724.89)
Sudan	NCD	31,930.03 (27,241.76–37,216.47)	31,517.96 (26,383.20–37,003.19)	29,904.64 (24,636.99–35,588.65)	28,747.01 (23,558.83–34,180.51)	27,944.91 (22,578.41–32,987.21)	27,190.86 (22,208.86–32,627.64)

Appendix Table 4. Rate of DALYs per 100,000 individuals for all causes and level 1 cause groups by location for 1990 - 2015, both sexes combined.

	Cause	1990	1995	2000	2005	2010	2015
Sudan	Injuries	7,684.85 (5,659.48–9,568.59)	6,490.58 (4,615.39–8,189.50)	6,458.67 (4,822.82–8,195.35)	5,675.03 (4,289.82–7,197.96)	5,472.66 (4,187.23–6,935.30)	5,015.00 (3,887.58–6,359.40)
Syria	All causes	36,020.59 (32,281.13–40,275.05)	31,627.68 (27,845.92–35,693.37)	28,487.58 (25,133.61–32,013.15)	26,482.86 (23,670.56–29,698.06)	25,026.94 (22,274.81–28,224.48)	43,132.03 (31,716.32–53,915.54)
Syria	Group I	7,842.29 (6,945.63–9,098.86)	5,791.36 (5,080.45–6,708.80)	4,154.15 (3,654.64–4,811.21)	3,540.96 (3,063.25–4,183.55)	3,174.52 (2,777.56–3,679.94)	2,931.08 (2,508.38–3,447.77)
Syria	NCD	25,449.16 (22,271.06–28,940.15)	23,481.11 (20,220.68–26,850.25)	22,213.42 (19,348.93–25,296.31)	21,113.29 (18,660.21–23,854.37)	20,145.20 (17,761.61–22,900.64)	20,011.15 (17,365.17–23,154.77)
Syria	Injuries	2,729.14 (2,318.38–3,144.07)	2,355.21 (2,006.19–2,705.95)	2,120.02 (1,853.62–2,413.10)	1,828.60 (1,620.01–2,037.06)	1,707.22 (1,541.75–1,892.14)	20,189.80 (9,258.61–30,722.90)
Tunisia	All causes	34,589.06 (31,418.38–38,016.50)	31,986.03 (28,861.59–35,528.75)	28,613.75 (25,372.00–32,249.02)	26,508.77 (23,172.75–30,296.50)	25,111.23 (21,689.61–29,000.02)	24,231.94 (20,613.80–28,145.74)
Tunisia	Group I	8,051.31 (7,070.58–8,960.06)	6,122.49 (5,540.83–6,736.52)	4,669.19 (4,193.33–5,144.98)	3,830.23 (3,394.49–4,314.10)	3,285.97 (2,872.58–3,759.01)	2,799.66 (2,382.88–3,249.40)
Tunisia	NCD	23,179.32 (20,502.68–25,858.13)	22,933.73 (20,240.55–25,926.61)	21,334.21 (18,561.32–24,360.35)	20,388.71 (17,521.15–23,658.78)	19,706.88 (16,800.31–23,017.13)	19,433.68 (16,330.76–22,761.30)
Tunisia	Injuries	3,358.44 (2,926.40–3,829.68)	2,929.82 (2,590.11–3,310.82)	2,610.34 (2,305.44–2,935.23)	2,289.84 (1,979.63–2,622.75)	2,118.38 (1,811.24–2,466.91)	1,998.61 (1,686.39–2,390.70)
Turkey	All causes	42,594.87 (39,203.56–46,309.57)	38,478.07 (35,008.11–42,310.40)	31,604.51 (28,559.04–35,079.04)	27,216.08 (24,230.30–30,505.82)	23,671.96 (20,659.33–26,874.53)	22,709.06 (19,620.08–26,098.36)
Turkey	Group I	11,207.54 (10,175.98–12,249.82)	8,998.43 (8,119.99–9,967.57)	6,218.33 (5,570.70–6,980.10)	4,382.49 (3,781.42–5,104.42)	3,358.71 (2,748.12–4,097.73)	2,787.13 (2,183.78–3,493.16)
Turkey	NCD	27,507.09 (24,594.84–30,607.32)	25,806.70 (22,920.22–28,889.83)	22,720.49 (20,075.08–25,725.27)	20,660.92 (17,958.07–23,501.00)	18,518.07 (15,946.81–21,342.26)	18,195.79 (15,605.43–21,118.92)
Turkey	Injuries	3,880.24 (3,277.98–4,386.53)	3,672.94 (3,253.79–4,059.02)	2,665.69 (2,413.19–2,935.56)	2,172.67 (1,980.18–2,381.04)	1,795.18 (1,639.72–1,955.19)	1,726.13 (1,556.50–1,924.19)
United Arab Emirates	All causes	36,013.79 (30,725.50–41,611.25)	33,372.08 (29,377.17–37,618.84)	30,948.14 (27,388.73–34,762.46)	27,662.62 (24,315.23–31,252.24)	26,801.00 (23,194.82–30,946.50)	26,804.14 (22,513.50–31,913.04)
United Arab Emirates	Group I	5,094.07 (3,818.80–6,778.68)	3,957.09 (3,009.14–5,345.01)	3,184.36 (2,510.06–4,148.25)	2,591.25 (2,155.53–3,268.82)	2,298.98 (1,817.14–2,940.89)	2,141.49 (1,669.33–2,759.45)
United Arab Emirates	NCD	25,218.55 (21,503.32–29,126.30)	24,403.31 (21,366.64–27,751.29)	23,291.99 (20,475.82–26,363.94)	21,228.47 (18,477.59–24,174.14)	21,071.97 (18,192.82–24,260.33)	21,376.76 (17,929.28–25,368.21)
United Arab Emirates	Injuries	5,701.17 (4,477.33–6,882.36)	5,011.69 (4,162.16–5,833.22)	4,471.80 (3,817.80–5,080.21)	3,842.91 (3,332.36–4,322.03)	3,430.06 (2,851.83–4,050.84)	3,285.89 (2,568.08–4,040.99)
Yemen	All causes	64,784.77 (52,632.30–79,459.63)	59,623.44 (47,859.19–74,706.30)	54,383.39 (43,177.91–68,882.51)	49,133.38 (37,657.87–63,614.64)	44,384.97 (33,727.12–59,606.44)	46,046.86 (36,247.10–60,655.73)
Yemen	Group I	23,794.67 (19,837.68–27,939.16)	20,043.04 (16,356.30–24,038.89)	16,521.02 (13,569.42–19,817.50)	12,906.26 (10,771.78–15,435.58)	9,877.94 (8,264.52–11,751.03)	8,397.91 (7,048.28–10,228.26)
Yemen	NCD	34,239.14 (25,181.41–45,557.39)	33,244.12 (24,432.13–44,473.08)	32,164.52 (23,713.66–42,983.17)	30,755.27 (21,962.67–41,599.06)	29,579.74 (21,714.61–41,416.00)	29,441.63 (21,803.12–40,483.85)
Yemen	Injuries	6,750.96 (4,386.95–9,212.95)	6,336.28 (4,155.44–8,780.50)	5,697.85 (3,758.77–8,114.04)	5,471.84 (3,661.48–7,743.01)	4,927.29 (3,389.94–7,193.56)	8,207.32 (6,338.14–10,724.69)
South Asia	All causes	66,116.09 (62,365.24–70,251.84)	61,269.78 (57,651.69–65,322.14)	56,718.49 (53,107.89–60,681.28)	52,169.88 (48,822.52–55,986.85)	47,505.15 (44,182.14–51,154.39)	43,467.77 (40,148.70–47,005.45)
South Asia	Group I	31,130.19 (29,991.34–32,377.53)	26,760.67 (25,727.36–27,938.47)	23,237.79 (22,257.34–24,311.82)	20,351.82 (19,439.30–21,366.79)	17,045.92 (16,195.20–17,988.55)	14,206.48 (13,407.40–15,087.82)
South Asia	NCD	29,360.69 (26,624.72–32,103.04)	29,040.01 (26,374.50–31,774.34)	28,418.90 (25,795.66–31,098.21)	26,924.60 (24,364.75–29,615.27)	26,272.26 (23,800.61–28,861.98)	25,365.80 (22,863.86–27,968.79)
South Asia	Injuries	5,625.21 (4,836.14–6,144.16)	5,469.10 (4,575.66–5,936.45)	5,061.81 (4,259.82–5,473.44)	4,893.47 (4,216.39–5,291.64)	4,186.98 (3,549.68–4,523.21)	3,895.48 (3,327.87–4,262.46)
Bangladesh	All causes	67,437.29 (62,857.03–71,978.15)	57,537.17 (53,507.42–61,922.59)	49,429.17 (45,619.26–53,665.39)	44,448.82 (40,848.78–48,566.68)	40,414.34 (36,816.90–44,514.83)	37,165.79 (33,258.91–41,556.94)
Bangladesh	Group I	33,499.38 (31,598.56–35,274.41)	25,126.43 (23,688.31–26,624.46)	18,408.79 (17,295.51–19,560.38)	14,226.17 (13,318.20–15,181.16)	11,089.79 (10,322.08–11,898.81)	9,046.85 (8,231.16–9,897.30)

Appendix Table 4. Rate of DALYs per 100,000 individuals for all causes and level 1 cause groups by location for 1990 - 2015, both sexes combined.

	Cause	1990	1995	2000	2005	2010	2015
Bangladesh	NCD	27,905.03 (24,805.27–31,220.59)	26,908.82 (23,840.15–30,241.41)	26,171.70 (23,211.02–29,379.07)	26,032.40 (23,053.23–29,036.27)	25,689.03 (22,766.92–28,743.90)	24,770.63 (21,649.16–28,181.69)
Bangladesh	Injuries	6,032.88 (4,711.09–6,980.92)	5,501.93 (4,223.09–6,337.94)	4,848.68 (3,640.78–5,609.80)	4,190.25 (3,246.30–4,762.85)	3,635.52 (2,915.13–4,082.38)	3,348.31 (2,668.13–3,872.86)
Bhutan	All causes	58,296.63 (52,902.44–64,181.95)	52,370.97 (47,482.84–57,550.47)	47,998.20 (43,508.73–53,006.05)	40,751.26 (36,567.20–45,140.38)	36,812.57 (32,580.94–41,460.95)	34,323.79 (30,013.71–39,069.88)
Bhutan	Group I	28,057.43 (25,129.64–31,093.06)	23,140.05 (20,786.87–25,766.30)	18,792.13 (16,784.96–20,976.75)	15,743.69 (14,040.97–17,619.84)	13,152.72 (11,577.97–14,786.23)	11,138.65 (9,744.79–12,736.40)
Bhutan	NCD	26,148.31 (22,492.42–30,372.55)	25,454.15 (21,841.75–29,296.18)	23,673.67 (20,434.19–27,180.41)	22,144.86 (19,167.59–25,574.33)	21,139.10 (18,029.27–24,481.31)	20,882.19 (17,604.70–24,370.57)
Bhutan	Injuries	4,090.89 (3,173.93–5,122.99)	3,776.76 (3,004.50–4,643.43)	5,532.39 (4,439.84–6,708.86)	2,862.71 (2,413.18–3,301.34)	2,520.75 (2,119.01–2,947.18)	2,302.96 (1,895.66–2,736.61)
India	All causes	67,084.47 (63,273.95–71,336.52)	62,311.97 (58,564.04–66,422.13)	57,884.55 (54,242.17–61,877.94)	52,742.87 (49,333.41–56,639.50)	48,058.31 (44,720.11–51,785.76)	43,720.92 (40,361.76–47,330.32)
India	Group I	31,349.05 (30,143.57–32,719.68)	27,251.14 (26,144.87–28,552.04)	23,964.42 (22,873.20–25,189.50)	21,116.14 (20,148.51–22,229.46)	17,608.24 (16,726.85–18,624.08)	14,565.94 (13,656.02–15,531.68)
India	NCD	29,904.64 (27,178.24–32,735.68)	29,357.33 (26,674.96–32,125.01)	28,648.55 (26,008.75–31,356.83)	26,870.30 (24,333.82–29,582.78)	26,163.39 (23,765.43–28,857.08)	25,199.14 (22,651.45–27,850.22)
India	Injuries	5,830.78 (5,071.23–6,367.88)	5,703.49 (4,796.78–6,207.79)	5,271.58 (4,457.56–5,700.67)	4,756.43 (4,046.69–5,125.15)	4,286.69 (3,626.18–4,649.36)	3,955.84 (3,352.29–4,350.64)
Nepal	All causes	65,128.25 (59,833.14–70,782.02)	56,482.80 (52,313.52–61,140.81)	48,131.42 (44,350.75–52,105.90)	43,192.10 (39,498.81–47,434.09)	39,279.62 (34,963.43–43,828.63)	38,148.21 (33,438.67–43,025.62)
Nepal	Group I	35,746.77 (33,364.69–38,375.77)	28,373.85 (26,568.93–30,182.02)	21,938.93 (20,436.27–23,440.53)	17,945.60 (16,364.64–19,505.16)	14,665.77 (13,046.23–16,242.58)	11,804.96 (10,390.18–13,374.95)
Nepal	NCD	25,274.71 (21,670.79–29,093.53)	24,251.05 (21,104.90–27,589.88)	22,728.45 (19,865.10–25,759.89)	21,805.46 (18,899.71–24,955.23)	21,712.12 (18,445.01–25,076.55)	21,593.34 (18,125.16–25,260.10)
Nepal	Injuries	4,106.77 (3,387.05–4,809.06)	3,857.90 (3,188.21–4,468.78)	3,464.03 (2,921.29–4,034.35)	3,441.04 (2,888.47–4,072.95)	2,901.74 (2,357.52–3,552.70)	4,749.91 (3,717.97–5,863.48)
Pakistan	All causes	56,105.37 (51,868.34–60,448.82)	56,424.22 (51,915.49–61,649.00)	55,016.97 (50,317.87–59,675.50)	55,545.44 (51,066.70–60,351.99)	49,855.31 (44,972.09–54,986.98)	46,675.49 (42,084.56–52,053.36)
Pakistan	Group I	26,005.31 (24,687.72–27,523.31)	23,788.97 (22,565.67–25,167.65)	21,763.27 (20,730.14–22,890.65)	19,741.71 (18,666.64–20,883.61)	17,350.09 (16,204.96–18,530.46)	15,097.76 (13,959.32–16,382.29)
Pakistan	NCD	26,583.23 (23,534.41–29,656.53)	29,025.62 (25,672.19–32,634.83)	29,555.52 (26,077.33–32,861.59)	29,143.27 (25,747.55–32,589.98)	28,496.50 (24,869.31–32,292.05)	27,899.46 (24,316.80–31,861.75)
Pakistan	Injuries	3,516.82 (2,954.18–4,103.49)	3,609.62 (3,071.94–4,182.18)	3,698.18 (3,133.89–4,262.97)	6,660.47 (5,226.31–8,087.35)	4,008.72 (3,434.19–4,609.43)	3,678.27 (3,163.45–4,282.88)
Sub-Saharan Africa	All causes	80,691.44 (76,254.02–85,747.96)	80,216.86 (75,877.59–85,111.77)	81,347.50 (77,093.32–86,022.94)	75,317.01 (71,341.81–79,981.55)	64,733.70 (60,483.77–69,738.78)	56,551.71 (52,272.75–61,475.97)
Sub-Saharan Africa	Group I	46,840.13 (44,584.55–49,423.60)	47,997.27 (45,921.84–50,247.35)	48,899.78 (46,887.80–51,049.55)	45,272.24 (43,451.85–47,348.15)	36,224.44 (34,582.97–38,267.06)	28,137.94 (26,510.02–30,227.04)
Sub-Saharan Africa	NCD	27,448.76 (24,670.93–30,368.13)	26,748.92 (24,143.54–29,667.95)	26,720.35 (24,043.29–29,566.31)	25,454.40 (22,866.04–28,341.77)	24,269.50 (21,694.11–27,223.09)	24,200.21 (21,514.66–27,271.30)
Sub-Saharan Africa	Injuries	6,402.55 (5,646.80–7,111.33)	5,470.67 (4,915.13–6,003.95)	5,727.36 (5,160.99–6,282.16)	4,590.38 (4,171.92–4,985.65)	4,239.77 (3,867.92–4,629.50)	4,213.56 (3,811.46–4,649.68)
Southern Sub-Saharan Africa	All causes	54,780.29 (51,131.03–58,813.58)	57,899.24 (53,960.87–62,357.13)	78,158.06 (73,061.13–83,901.26)	89,843.65 (83,981.40–95,919.10)	80,084.90 (75,188.46–85,623.03)	60,163.61 (55,686.04–65,226.12)
Southern Sub-Saharan Africa	Group I	20,242.23 (19,051.02–21,871.19)	24,502.50 (22,688.08–26,851.20)	40,304.20 (37,435.96–43,556.43)	54,141.72 (50,956.98–57,769.32)	48,337.84 (45,653.38–51,419.59)	29,292.52 (27,707.16–31,096.00)
Southern Sub-Saharan Africa	NCD	27,228.76 (24,496.29–30,087.49)	26,613.54 (23,833.82–29,672.27)	30,397.89 (27,257.79–33,974.55)	29,064.83 (25,547.45–32,854.20)	26,195.61 (23,040.74–29,991.81)	25,590.41 (22,534.26–28,714.70)
Southern Sub-Saharan Africa	Injuries	7,309.29 (6,545.43–7,970.72)	6,783.20 (6,072.95–7,467.76)	7,455.96 (6,655.42–8,268.05)	6,637.10 (5,806.47–7,522.21)	5,551.45 (4,926.94–6,343.52)	5,280.68 (4,721.73–5,955.35)
Botswana	All causes	54,942.21 (33,500.70–99,978.82)	70,850.94 (40,889.85–130,816.08)	105,477.39 (58,188.42–188,350.19)	98,865.34 (56,958.47–176,848.05)	75,290.22 (44,713.96–145,398.57)	65,852.12 (40,519.51–130,297.68)

Appendix Table 4. Rate of DALYs per 100,000 individuals for all causes and level 1 cause groups by location for 1990 - 2015, both sexes combined.							
	**Cause**	**1990**	**1995**	**2000**	**2005**	**2010**	**2015**
Botswana	Group I	23,355.50 (15,210.82–39,424.75)	36,917.39 (21,713.10–59,229.79)	65,912.00 (38,812.62–98,527.40)	58,880.38 (37,400.77–85,173.85)	39,582.99 (25,004.88–60,288.98)	32,094.34 (20,682.18–48,067.75)
Botswana	NCD	27,214.43 (15,590.26–50,555.88)	29,051.95 (15,461.01–62,770.53)	33,298.45 (15,798.62–76,621.69)	33,458.44 (15,842.29–75,886.79)	30,051.96 (15,820.86–68,805.21)	28,615.75 (15,953.81–66,989.94)
Botswana	Injuries	4,372.28 (2,147.75–9,453.71)	4,881.59 (2,174.37–12,340.85)	6,266.94 (2,288.86–18,414.42)	6,526.53 (2,305.89–18,827.48)	5,655.26 (2,223.58–17,267.05)	5,142.03 (2,175.45–16,172.01)
Lesotho	All causes	59,025.17 (51,300.27–67,899.65)	64,040.42 (55,921.75–72,138.07)	89,795.62 (78,004.81–104,431.51)	125,170.53 (109,283.28–142,863.98)	108,696.46 (90,542.36–129,652.25)	106,744.95 (82,943.78–136,690.35)
Lesotho	Group I	28,450.57 (25,027.47–33,285.56)	33,358.29 (29,824.16–38,071.30)	55,804.25 (47,882.39–66,723.90)	81,316.01 (70,249.43–94,649.86)	64,691.24 (56,503.80–74,193.58)	61,523.60 (51,614.06–73,988.23)
Lesotho	NCD	25,299.94 (20,568.97–30,566.72)	25,127.00 (19,595.40–30,057.75)	27,742.54 (22,319.26–34,710.72)	35,645.36 (29,659.47–43,580.04)	35,514.25 (26,806.13–46,579.95)	36,608.46 (24,736.21–52,754.43)
Lesotho	Injuries	5,274.66 (4,131.30–6,659.89)	5,555.13 (4,125.66–6,982.46)	6,248.83 (4,578.39–8,215.14)	8,209.16 (6,025.55–10,649.90)	8,490.97 (5,382.96–12,438.23)	8,612.88 (4,411.40–14,535.92)
Namibia	All causes	56,384.21 (50,689.73–61,848.62)	59,908.79 (53,825.73–66,420.40)	80,141.33 (70,466.15–91,525.72)	82,677.54 (72,051.23–94,983.30)	57,397.00 (48,395.06–67,699.38)	50,815.81 (40,694.89–63,617.97)
Namibia	Group I	25,288.41 (22,877.00–28,107.71)	27,305.85 (24,452.97–30,796.38)	41,823.47 (36,182.95–48,873.92)	48,961.29 (42,512.98–56,754.00)	30,676.27 (26,413.97–36,788.23)	25,716.00 (21,380.58–30,730.02)
Namibia	NCD	25,702.36 (22,001.52–29,222.06)	27,165.55 (23,333.90–30,935.33)	31,621.86 (26,234.94–37,436.26)	28,045.78 (22,455.01–34,758.51)	22,296.14 (17,308.68–27,875.93)	21,118.54 (15,789.81–28,030.89)
Namibia	Injuries	5,393.44 (4,487.29–6,305.64)	5,437.39 (4,533.90–6,517.37)	6,696.00 (5,278.55–8,459.32)	5,670.47 (4,251.77–7,590.32)	4,424.59 (2,995.81–5,871.05)	3,981.27 (2,488.17–6,189.14)
South Africa	All causes	55,047.30 (51,353.14–59,104.54)	53,773.02 (50,387.31–57,739.51)	71,151.06 (66,388.62–76,387.89)	82,805.79 (76,895.82–88,988.44)	75,815.00 (70,558.92–81,504.11)	57,336.56 (53,154.44–61,805.12)
South Africa	Group I	18,434.37 (17,365.85–20,090.19)	18,682.67 (17,536.00–20,479.42)	31,780.53 (29,385.84–34,710.13)	47,182.82 (43,725.55–51,212.81)	44,821.89 (41,636.90–48,465.27)	26,516.25 (24,881.41–28,477.50)
South Africa	NCD	28,079.00 (25,180.65–31,096.23)	27,296.55 (24,614.19–30,210.08)	31,000.83 (27,718.53–34,646.68)	28,539.04 (24,846.42–32,726.56)	25,296.37 (21,982.84–28,998.61)	25,292.42 (22,415.25–28,263.92)
South Africa	Injuries	8,533.93 (7,601.75–9,287.17)	7,793.80 (6,892.47–8,443.06)	8,369.69 (7,439.21–9,172.96)	7,083.92 (6,134.39–8,110.86)	5,696.73 (4,936.07–6,546.15)	5,527.89 (4,924.86–6,169.11)
Swaziland	All causes	59,113.18 (48,891.23–69,809.06)	63,588.20 (52,635.90–74,422.52)	98,682.30 (83,578.93–116,140.69)	130,692.86 (111,349.20–153,455.13)	110,247.72 (88,864.64–134,501.60)	88,875.38 (65,636.28–116,849.08)
Swaziland	Group I	24,215.45 (20,674.10–28,656.63)	32,538.48 (28,015.76–39,006.73)	60,281.06 (50,303.38–73,985.57)	83,565.38 (70,663.08–99,314.55)	65,471.45 (56,003.96–77,356.18)	46,915.63 (39,094.95–56,445.25)
Swaziland	NCD	28,718.91 (22,584.97–34,926.12)	25,776.04 (18,846.20–32,427.78)	31,673.70 (24,134.00–41,023.63)	38,854.88 (29,713.97–50,714.76)	36,878.09 (24,852.56–51,688.15)	34,590.04 (21,325.86–51,500.27)
Swaziland	Injuries	6,178.82 (4,507.46–8,212.02)	5,273.68 (3,402.43–7,057.39)	6,727.54 (4,521.59–9,464.70)	8,272.59 (5,519.58–12,059.51)	7,898.19 (4,347.15–12,773.25)	7,369.71 (3,414.85–13,175.48)
Zimbabwe	All causes	52,705.99 (46,754.07–58,997.90)	72,227.64 (60,435.14–86,619.30)	102,526.10 (85,800.87–121,390.38)	116,617.99 (101,328.65–131,920.16)	97,308.79 (86,376.63–108,757.71)	62,775.02 (50,753.14–76,971.84)
Zimbabwe	Group I	25,467.38 (22,713.87–29,735.40)	45,512.99 (37,713.41–56,708.62)	71,199.33 (58,909.00–86,445.76)	82,312.06 (71,298.47–94,382.23)	63,877.30 (58,194.05–69,813.59)	34,292.95 (29,646.99–39,758.59)
Zimbabwe	NCD	23,257.30 (19,324.13–27,238.92)	22,760.95 (16,875.56–30,090.52)	26,632.12 (20,567.51–35,685.07)	29,212.99 (22,485.90–37,238.30)	28,381.26 (22,312.42–35,341.26)	24,182.51 (17,583.58–32,536.25)
Zimbabwe	Injuries	3,981.31 (2,997.51–5,283.09)	3,953.71 (2,536.61–6,138.93)	4,694.65 (3,021.03–7,363.54)	5,092.94 (3,339.75–7,325.23)	5,050.22 (3,540.67–6,724.66)	4,299.56 (2,672.91–6,335.68)
Western Sub-Saharan Africa	All causes	78,550.36 (72,711.67–85,528.89)	76,875.97 (72,391.83–82,145.85)	75,794.06 (71,616.17–80,706.38)	70,773.75 (66,415.75–75,745.20)	61,516.53 (57,017.00–66,534.30)	54,904.59 (50,083.64–61,151.27)
Western Sub-Saharan Africa	Group I	48,561.64 (45,385.40–52,107.03)	47,529.82 (44,978.59–50,164.61)	46,674.97 (44,223.18–49,150.71)	43,180.73 (40,892.41–45,349.73)	35,262.84 (33,343.95–37,286.60)	28,761.99 (26,706.81–31,207.63)
Western Sub-Saharan Africa	NCD	25,242.54 (21,692.60–29,163.61)	24,666.67 (21,776.76–27,882.86)	24,633.31 (21,741.32–27,711.32)	23,698.88 (20,891.42–26,773.38)	22,655.46 (19,626.87–25,818.89)	22,463.11 (19,444.65–25,927.11)
Western Sub-Saharan Africa	Injuries	4,746.17 (4,023.08–5,529.68)	4,679.48 (4,118.09–5,279.11)	4,485.78 (3,902.33–5,036.53)	3,894.14 (3,422.43–4,390.59)	3,598.23 (3,165.46–4,068.23)	3,679.49 (3,201.54–4,201.05)

Appendix Table 4. Rate of DALYs per 100,000 individuals for all causes and level 1 cause groups by location for 1990 - 2015, both sexes combined.							
	Cause	1990	1995	2000	2005	2010	2015
Benin	All causes	72,984.12 (67,027.15–79,285.90)	70,607.35 (64,525.82–76,545.05)	68,844.82 (61,941.17–76,274.09)	64,520.77 (55,462.94–74,943.97)	58,504.48 (46,546.09–74,192.02)	54,197.91 (40,681.68–72,933.29)
Benin	Group I	43,355.90 (40,125.68–47,254.61)	40,585.02 (37,518.68–44,026.83)	38,148.91 (35,041.50–41,525.16)	34,508.74 (30,832.82–38,584.97)	28,462.93 (23,906.07–33,735.95)	23,895.44 (19,051.47–30,245.93)
Benin	NCD	24,641.10 (21,188.55–28,437.71)	24,871.74 (21,320.93–28,398.93)	25,666.87 (21,603.12–30,112.84)	25,693.09 (20,787.96–31,568.31)	25,944.84 (19,471.04–35,014.89)	26,289.45 (18,414.56–37,374.51)
Benin	Injuries	4,987.12 (3,777.62–6,276.71)	5,150.58 (4,123.96–6,157.52)	5,029.04 (4,007.26–6,018.79)	4,318.94 (3,323.31–5,501.91)	4,096.71 (2,819.35–5,703.12)	4,013.03 (2,635.48–6,143.02)
Burkina Faso	All causes	86,724.25 (79,845.05–93,856.54)	86,775.39 (79,815.24–93,888.60)	82,621.41 (76,231.09–89,812.54)	72,458.79 (65,604.44–80,481.40)	63,120.11 (54,181.74–73,613.49)	57,763.94 (46,384.11–73,423.50)
Burkina Faso	Group I	57,333.98 (53,315.31–61,176.68)	58,001.52 (54,121.32–61,741.98)	54,022.38 (50,598.48–57,707.78)	45,470.95 (41,998.38–49,133.48)	36,473.52 (32,669.45–41,217.30)	30,102.58 (25,388.85–36,778.30)
Burkina Faso	NCD	24,128.22 (20,704.37–27,758.97)	23,709.17 (20,197.06–27,254.89)	23,870.00 (20,502.58–27,731.14)	22,819.23 (19,104.94–27,093.15)	22,738.33 (17,952.96–28,040.23)	23,555.36 (17,279.54–31,777.36)
Burkina Faso	Injuries	5,262.05 (4,356.36–6,213.14)	5,064.69 (4,240.66–5,909.30)	4,729.02 (4,014.93–5,544.19)	4,168.60 (3,492.21–4,963.97)	3,908.26 (3,041.80–4,862.85)	4,106.00 (2,979.58–5,588.08)
Cameroon	All causes	66,400.24 (60,790.15–72,500.15)	71,086.68 (64,620.60–78,023.49)	77,650.67 (69,834.67–86,329.78)	75,741.73 (67,291.16–84,707.08)	66,929.26 (56,999.83–77,635.70)	62,233.01 (49,502.23–79,361.41)
Cameroon	Group I	36,271.87 (33,611.23–39,304.99)	40,492.90 (37,216.85–44,083.79)	45,399.71 (41,612.65–49,356.26)	44,137.97 (40,592.69–48,003.73)	36,389.99 (32,422.34–40,407.96)	31,781.08 (26,688.32–37,459.68)
Cameroon	NCD	25,717.55 (22,482.23–29,225.07)	25,921.03 (22,247.79–29,994.26)	27,584.34 (22,600.80–32,573.94)	27,218.84 (22,295.03–32,697.16)	26,315.29 (20,507.23–32,999.88)	26,122.57 (18,708.46–36,505.40)
Cameroon	Injuries	4,410.82 (3,600.68–5,207.63)	4,672.75 (3,812.73–5,546.44)	4,666.62 (3,657.88–5,702.05)	4,384.92 (3,451.92–5,409.47)	4,223.98 (3,138.42–5,390.59)	4,329.36 (3,030.45–6,086.81)
Cape Verde	All causes	40,465.09 (37,175.12–43,817.59)	41,045.91 (36,104.04–46,440.41)	40,512.95 (33,634.02–47,867.84)	38,541.52 (32,457.67–44,610.89)	34,900.40 (31,648.43–38,575.24)	31,771.67 (27,849.41–36,319.34)
Cape Verde	Group I	13,959.46 (13,040.20–15,033.11)	13,096.29 (11,930.28–14,487.33)	11,906.78 (10,445.74–13,483.56)	10,342.54 (9,241.78–11,525.13)	8,607.94 (7,908.68–9,417.10)	7,610.23 (6,890.14–8,440.11)
Cape Verde	NCD	23,530.15 (21,110.76–26,247.50)	24,132.09 (20,611.75–28,055.11)	23,807.94 (19,027.29–28,964.02)	23,497.34 (19,355.31–27,798.32)	22,096.47 (19,594.52–24,865.90)	20,577.18 (17,514.26–23,899.26)
Cape Verde	Injuries	2,975.48 (2,589.25–3,455.63)	3,817.54 (3,129.45–4,508.17)	4,798.23 (3,412.93–6,415.71)	4,701.64 (3,534.65–6,062.02)	4,195.99 (3,566.94–4,697.93)	3,584.26 (2,923.78–4,382.87)
Chad	All causes	80,290.04 (74,252.19–86,736.02)	80,211.03 (73,280.70–87,097.22)	86,366.72 (77,717.85–95,133.28)	81,696.08 (70,257.81–96,319.68)	73,355.80 (57,941.30–91,114.51)	68,545.99 (52,784.62–89,004.97)
Chad	Group I	51,233.29 (47,707.81–54,953.70)	52,306.68 (48,300.25–56,291.30)	54,396.38 (50,103.44–59,265.01)	50,943.87 (45,207.78–57,580.78)	42,111.98 (35,416.43–50,083.78)	37,443.59 (30,896.60–46,019.41)
Chad	NCD	22,618.72 (19,243.05–26,234.83)	22,801.67 (19,208.64–26,560.75)	25,479.32 (21,111.35–30,015.64)	25,157.30 (19,581.87–32,276.89)	25,649.37 (18,136.02–34,880.50)	25,645.95 (17,434.47–36,560.37)
Chad	Injuries	6,438.03 (4,831.31–9,020.57)	5,102.68 (4,076.77–6,912.17)	6,491.02 (5,242.31–7,933.76)	5,594.91 (4,159.45–7,442.83)	5,594.45 (3,910.00–7,742.07)	5,456.44 (3,830.42–7,794.10)
Cote d'Ivoire	All causes	75,439.18 (68,499.43–82,628.05)	83,710.54 (74,938.05–92,162.64)	84,953.21 (76,849.91–93,984.99)	80,183.79 (72,345.60–88,615.98)	70,143.88 (60,102.61–82,163.40)	62,891.27 (50,290.04–79,438.95)
Cote d'Ivoire	Group I	43,044.67 (39,931.60–46,363.44)	48,222.75 (44,569.74–52,438.27)	49,862.32 (45,981.24–53,954.74)	47,571.22 (43,896.96–51,292.63)	39,476.72 (35,697.74–43,860.80)	32,225.52 (27,560.31–38,078.24)
Cote d'Ivoire	NCD	27,389.33 (23,434.74–31,593.56)	29,832.66 (24,409.42–35,030.89)	29,557.10 (24,569.12–35,674.29)	27,830.51 (23,097.79–33,303.72)	26,283.14 (20,404.88–33,182.03)	26,408.44 (19,255.96–35,913.56)
Cote d'Ivoire	Injuries	5,005.18 (4,152.39–5,935.87)	5,655.13 (4,559.63–6,750.76)	5,533.79 (4,459.92–6,724.67)	4,782.06 (3,841.30–5,837.81)	4,384.03 (3,188.97–5,650.24)	4,257.31 (2,859.19–6,247.79)
The Gambia	All causes	57,372.21 (45,692.43–72,937.24)	54,114.63 (45,282.49–64,574.40)	51,234.87 (45,234.70–57,315.77)	48,091.55 (42,393.79–53,840.28)	44,391.47 (38,545.84–50,400.65)	40,849.12 (33,671.75–49,875.60)
The Gambia	Group I	29,259.94 (24,722.28–35,209.06)	26,824.62 (23,509.98–30,811.27)	24,424.95 (22,240.55–26,842.39)	21,728.79 (19,721.28–23,789.33)	19,013.27 (17,054.52–21,055.79)	16,097.21 (13,867.25–18,854.38)
The Gambia	NCD	24,384.17 (17,810.61–32,968.81)	23,675.94 (18,438.33–29,935.33)	23,252.13 (19,368.57–26,992.93)	22,953.82 (19,359.16–26,576.30)	22,267.13 (18,453.83–26,149.60)	21,848.74 (17,318.86–27,602.09)

Appendix Table 4. Rate of DALYs per 100,000 individuals for all causes and level 1 cause groups by location for 1990 - 2015, both sexes combined.

	Cause	1990	1995	2000	2005	2010	2015
The Gambia	Injuries	3,728.09 (2,779.42–4,979.18)	3,614.07 (2,854.49–4,560.70)	3,557.79 (2,982.94–4,184.37)	3,408.94 (2,867.63–4,020.22)	3,111.07 (2,558.19–3,752.41)	2,903.17 (2,249.53–3,829.36)
Ghana	All causes	66,193.13 (56,326.46–78,540.02)	60,760.18 (53,785.88–68,365.20)	60,216.57 (52,714.59–68,419.68)	57,930.68 (49,218.17–67,142.80)	52,515.07 (41,361.01–65,731.50)	46,529.82 (35,166.41–62,268.70)
Ghana	Group I	35,267.03 (31,217.78–39,849.64)	32,311.71 (29,747.14–35,176.35)	31,437.82 (28,846.13–34,408.45)	29,443.81 (26,504.82–32,683.72)	24,590.22 (20,966.72–28,960.47)	18,626.92 (15,181.02–22,983.52)
Ghana	NCD	26,636.62 (21,058.21–33,540.99)	24,508.30 (20,484.09–29,060.94)	25,145.05 (20,617.08–30,281.28)	24,845.41 (19,613.17–30,256.34)	24,481.23 (17,702.96–32,563.46)	24,492.27 (17,352.79–34,276.27)
Ghana	Injuries	4,289.48 (3,349.81–5,519.21)	3,940.16 (3,285.67–4,764.96)	3,633.70 (2,933.17–4,452.03)	3,641.46 (2,848.93–4,566.93)	3,443.62 (2,422.85–4,758.13)	3,410.63 (2,342.19–4,983.30)
Guinea	All causes	85,274.99 (79,061.66–91,404.52)	80,397.58 (75,113.16–86,694.41)	77,377.34 (71,250.14–83,868.25)	71,857.46 (65,259.68–78,824.55)	66,422.33 (58,326.80–74,310.90)	62,825.88 (51,095.27–77,402.10)
Guinea	Group I	54,055.52 (50,343.88–58,055.88)	49,497.19 (46,501.28–52,873.46)	46,090.20 (42,800.80–49,331.80)	41,915.89 (38,611.48–45,329.08)	36,065.85 (32,644.66–39,574.51)	32,128.97 (27,354.29–37,643.54)
Guinea	NCD	26,517.09 (22,793.64–30,513.18)	26,130.61 (22,672.47–29,802.05)	26,516.40 (22,871.29–30,561.15)	25,776.02 (21,885.58–29,923.73)	26,268.21 (21,703.06–31,174.22)	26,747.73 (19,966.97–35,139.19)
Guinea	Injuries	4,702.38 (3,592.24–5,809.46)	4,769.78 (3,781.26–5,742.49)	4,770.73 (3,867.57–5,738.31)	4,165.56 (3,429.94–4,970.81)	4,088.28 (3,210.47–4,988.42)	3,949.18 (2,798.01–5,417.61)
Guinea-Bissau	All causes	85,720.30 (63,176.44–133,572.14)	82,774.19 (59,474.53–136,327.23)	81,363.65 (56,988.69–132,127.40)	80,834.44 (54,445.54–134,465.75)	76,599.85 (51,390.44–128,935.81)	72,481.72 (47,802.19–137,827.58)
Guinea-Bissau	Group I	51,656.04 (42,004.85–70,504.01)	48,508.63 (38,754.27–69,420.03)	46,585.99 (35,885.71–67,208.43)	44,538.10 (33,095.77–64,818.04)	39,896.27 (29,503.09–57,716.80)	36,451.13 (26,726.33–57,729.58)
Guinea-Bissau	NCD	28,702.84 (16,651.01–52,939.31)	28,523.92 (16,162.63–54,841.92)	28,963.62 (16,224.94–54,702.00)	30,295.40 (16,501.07–59,069.16)	30,701.59 (16,866.58–60,138.24)	30,458.40 (16,750.35–67,569.92)
Guinea-Bissau	Injuries	5,361.42 (3,140.44–9,544.39)	5,741.64 (3,409.99–10,790.17)	5,814.04 (3,345.68–11,009.00)	6,000.94 (3,345.49–11,868.85)	6,001.99 (3,368.22–12,067.40)	5,572.20 (3,111.67–12,491.58)
Liberia	All causes	97,142.45 (85,300.09–109,745.54)	89,890.82 (81,559.51–98,919.75)	74,970.80 (68,605.68–81,892.84)	67,356.49 (60,521.50–74,337.61)	60,241.51 (52,196.80–68,470.93)	53,750.39 (44,566.85–65,676.69)
Liberia	Group I	56,343.28 (50,979.78–62,038.58)	56,374.86 (52,103.56–61,557.87)	49,409.26 (45,449.77–53,750.50)	41,780.60 (38,108.23–45,532.82)	33,936.02 (30,329.71–37,955.73)	28,090.27 (24,078.98–33,377.41)
Liberia	NCD	25,130.53 (20,262.75–30,584.90)	22,481.70 (18,644.60–26,405.72)	21,329.81 (17,889.00–25,409.89)	21,901.59 (18,286.32–25,878.48)	22,734.95 (18,534.41–27,007.55)	22,339.63 (17,265.52–28,462.35)
Liberia	Injuries	15,668.64 (8,762.94–24,038.50)	11,034.26 (6,694.82–16,107.80)	4,231.73 (3,429.01–5,159.97)	3,674.31 (3,033.50–4,420.50)	3,570.54 (2,862.56–4,423.68)	3,320.49 (2,482.75–4,409.53)
Mali	All causes	91,934.59 (85,991.28–97,916.61)	87,308.08 (81,365.16–93,193.10)	80,798.15 (75,287.60–86,772.80)	71,004.07 (65,162.44–77,923.85)	64,014.28 (57,602.29–71,398.96)	60,760.77 (51,253.42–72,828.83)
Mali	Group I	58,541.77 (54,574.97–62,442.93)	55,029.25 (51,417.12–58,632.76)	49,542.00 (46,246.10–53,037.92)	43,392.56 (40,492.54–46,713.94)	36,719.10 (33,535.43–40,241.32)	33,130.35 (28,988.01–38,282.84)
Mali	NCD	27,897.35 (24,134.85–31,696.47)	27,199.03 (23,512.35–30,903.94)	25,330.21 (21,492.25–29,218.49)	23,241.77 (19,591.68–27,039.64)	23,109.19 (19,221.96–27,512.90)	23,534.12 (18,248.15–30,916.68)
Mali	Injuries	5,495.47 (4,443.76–6,574.35)	5,079.80 (4,165.79–6,111.90)	5,925.94 (4,747.92–7,450.48)	4,369.74 (3,592.95–5,291.47)	4,185.99 (3,344.35–5,131.13)	4,096.30 (3,195.66–5,321.07)
Mauritania	All causes	63,335.63 (57,739.81–69,735.87)	58,628.00 (53,109.07–64,699.49)	52,381.74 (47,371.38–58,143.97)	46,857.87 (41,528.08–52,758.26)	41,773.53 (35,832.86–48,686.76)	39,080.40 (31,282.31–48,219.39)
Mauritania	Group I	31,866.90 (29,389.91–34,851.26)	29,449.27 (27,006.52–32,621.35)	25,674.15 (23,374.80–28,841.10)	22,359.99 (20,205.88–25,313.78)	18,255.50 (16,058.07–20,797.82)	15,423.02 (13,001.06–18,389.52)
Mauritania	NCD	26,389.80 (22,808.96–30,283.94)	25,119.25 (21,671.36–28,855.89)	22,916.32 (19,303.53–26,641.05)	21,014.54 (17,210.40–24,935.67)	20,381.10 (16,513.32–24,803.82)	20,603.50 (15,643.57–26,575.94)
Mauritania	Injuries	5,078.93 (3,967.10–6,364.53)	4,059.49 (3,241.14–4,862.87)	3,791.27 (3,075.28–4,484.23)	3,483.34 (2,738.26–4,250.74)	3,136.93 (2,443.95–4,027.58)	3,053.88 (2,239.81–4,226.84)
Niger	All causes	101,452.34 (95,556.87–108,161.07)	93,329.13 (87,375.39–100,199.06)	83,499.21 (78,103.97–89,423.29)	72,492.80 (67,136.57–78,807.86)	63,025.62 (55,958.17–71,220.22)	57,942.09 (48,031.23–70,182.17)
Niger	Group I	71,009.22 (66,630.70–75,461.87)	64,747.05 (60,819.98–69,064.62)	56,212.37 (52,505.90–59,810.70)	46,211.98 (43,140.45–49,674.19)	37,351.15 (34,011.44–41,004.87)	31,058.91 (26,728.11–36,739.55)

Appendix Table 4. Rate of DALYs per 100,000 individuals for all causes and level 1 cause groups by location for 1990 - 2015, both sexes combined.

	Cause	1990	1995	2000	2005	2010	2015
Niger	NCD	24,392.66 (20,766.31–28,543.59)	23,066.33 (19,569.05–27,221.95)	22,186.79 (18,835.00–25,654.07)	21,746.59 (18,385.40–25,483.91)	21,545.97 (17,718.66–25,696.41)	22,349.26 (16,998.75–28,811.76)
Niger	Injuries	6,050.45 (4,634.28–7,653.77)	5,515.76 (4,362.68–6,860.42)	5,100.05 (4,170.25–6,224.46)	4,534.24 (3,642.98–5,536.91)	4,128.51 (3,224.12–5,169.60)	4,533.92 (3,385.49–6,020.04)
Nigeria	All causes	79,233.19 (69,652.23–91,350.87)	76,659.61 (69,943.99–84,765.17)	75,774.57 (69,761.72–82,522.33)	71,170.41 (64,923.82–77,923.28)	59,890.28 (53,588.24–66,792.99)	51,571.14 (44,656.12–61,048.84)
Nigeria	Group I	50,362.70 (45,328.14–56,325.45)	48,819.83 (44,981.87–53,242.52)	48,427.08 (44,865.18–52,172.18)	45,758.49 (42,197.12–49,197.63)	36,769.90 (33,731.43–40,155.85)	28,892.80 (25,377.41–33,069.54)
Nigeria	NCD	24,580.16 (19,320.58–31,283.05)	23,659.57 (19,797.36–28,256.24)	23,317.55 (19,778.44–27,143.50)	22,040.44 (18,390.87–25,976.37)	20,115.16 (16,267.79–24,107.93)	19,482.31 (15,710.24–24,699.04)
Nigeria	Injuries	4,290.33 (3,265.86–5,630.39)	4,180.22 (3,405.83–5,116.34)	4,029.94 (3,312.44–4,817.85)	3,371.48 (2,793.63–4,063.57)	3,005.21 (2,501.07–3,646.16)	3,196.02 (2,589.40–4,052.84)
Sao Tome and Principe	All causes	52,740.65 (47,872.65–57,969.41)	51,002.05 (45,521.04–56,942.28)	49,284.83 (44,244.30–54,939.22)	47,030.02 (41,179.35–53,617.07)	43,685.47 (35,309.32–54,021.21)	41,299.95 (30,748.96–54,888.36)
Sao Tome and Principe	Group I	23,428.95 (21,523.71–25,693.91)	21,255.28 (19,379.79–23,540.75)	18,529.66 (16,828.00–20,499.95)	15,857.59 (14,028.51–18,170.88)	13,320.05 (11,296.90–15,987.10)	11,648.70 (9,163.42–14,659.97)
Sao Tome and Principe	NCD	25,933.55 (22,619.76–29,531.13)	26,438.46 (22,708.49–30,397.57)	27,508.29 (23,840.32–31,391.69)	28,036.93 (23,983.43–32,667.55)	27,492.38 (21,257.27–34,799.36)	26,951.46 (19,135.57–37,060.67)
Sao Tome and Principe	Injuries	3,378.15 (2,774.57–3,935.70)	3,308.31 (2,780.36–3,878.24)	3,246.87 (2,768.67–3,833.04)	3,135.50 (2,580.77–3,817.00)	2,873.03 (2,159.66–3,803.74)	2,699.79 (1,805.27–3,972.57)
Senegal	All causes	66,733.55 (60,913.81–72,946.32)	64,870.80 (58,709.15–70,898.12)	61,402.24 (55,566.31–68,146.49)	54,988.00 (47,336.66–64,263.34)	49,827.89 (38,896.25–64,395.42)	46,699.50 (34,369.64–63,974.98)
Senegal	Group I	38,273.85 (35,628.14–41,088.38)	36,564.97 (33,900.03–39,192.20)	33,543.98 (30,921.52–36,369.61)	27,544.41 (24,465.84–31,187.36)	22,372.09 (18,451.44–27,851.18)	18,750.35 (14,535.04–25,291.52)
Senegal	NCD	24,137.94 (20,746.90–27,845.86)	23,884.44 (20,386.06–27,265.48)	23,810.70 (20,338.51–27,649.45)	23,618.98 (19,301.46–28,830.47)	23,867.56 (17,470.09–31,603.98)	24,399.35 (17,061.44–34,078.64)
Senegal	Injuries	4,321.77 (3,619.75–5,123.41)	4,421.39 (3,788.19–5,183.87)	4,047.56 (3,368.74–4,804.18)	3,824.61 (3,000.12–4,856.12)	3,588.24 (2,570.16–5,092.07)	3,549.80 (2,408.95–5,330.23)
Sierra Leone	All causes	86,510.07 (76,747.15–98,558.47)	88,547.66 (80,957.66–97,394.36)	86,425.70 (79,965.79–93,400.33)	83,847.91 (77,710.01–90,597.78)	74,758.80 (66,933.41–83,080.48)	67,927.72 (56,823.21–82,427.50)
Sierra Leone	Group I	53,682.97 (47,875.49–59,231.20)	51,303.43 (47,017.53–55,706.79)	51,810.54 (47,344.89–55,813.22)	49,100.35 (45,330.01–52,959.88)	40,552.55 (36,753.69–44,053.90)	34,946.36 (30,366.55–40,063.30)
Sierra Leone	NCD	27,910.18 (22,329.68–34,576.89)	27,186.52 (22,745.25–32,503.80)	28,100.40 (23,948.04–33,105.32)	29,837.69 (25,760.50–34,428.22)	29,648.77 (24,861.04–34,661.76)	28,726.73 (21,941.70–37,492.30)
Sierra Leone	Injuries	4,916.93 (3,492.97–6,414.13)	10,057.71 (6,573.66–14,340.24)	6,514.76 (5,047.64–8,187.00)	4,909.88 (3,854.28–5,922.66)	4,557.48 (3,583.13–5,643.06)	4,254.64 (3,114.21–5,804.55)
Togo	All causes	66,839.53 (60,987.47–73,180.62)	67,178.10 (60,829.51–74,164.69)	69,629.01 (62,173.27–77,490.25)	69,243.97 (61,166.21–77,858.90)	63,723.61 (55,056.72–73,072.70)	56,003.24 (45,069.61–68,811.36)
Togo	Group I	36,302.95 (33,844.74–38,930.79)	36,578.30 (33,767.92–39,464.71)	38,331.20 (35,172.05–42,098.62)	38,278.53 (34,856.99–41,930.63)	33,642.39 (30,205.54–37,283.13)	26,495.61 (22,732.88–31,049.51)
Togo	NCD	26,322.04 (22,655.06–30,343.70)	26,206.85 (22,424.29–30,446.97)	26,800.66 (22,168.17–31,521.45)	26,219.00 (21,625.44–31,405.27)	25,941.46 (21,064.35–31,408.95)	25,617.67 (19,391.71–33,656.35)
Togo	Injuries	4,214.55 (3,543.63–4,969.99)	4,392.95 (3,707.06–5,171.89)	4,497.15 (3,668.49–5,381.23)	4,746.44 (3,849.28–5,801.17)	4,139.75 (3,185.02–5,195.22)	3,889.96 (2,729.10–5,472.21)
Eastern Sub-Saharan Africa	All causes	88,488.53 (83,260.92–93,823.68)	87,822.41 (82,632.09–93,025.05)	85,870.95 (80,819.89–90,710.43)	74,288.83 (69,817.42–79,123.88)	61,380.59 (56,175.74–67,045.41)	54,266.06 (48,564.98–60,831.77)
Eastern Sub-Saharan Africa	Group I	51,510.82 (49,329.71–53,996.44)	53,809.70 (51,570.77–56,146.09)	52,527.85 (50,280.97–54,864.23)	44,401.93 (42,438.21–46,667.19)	32,825.32 (30,819.80–35,021.40)	25,718.64 (23,651.93–28,009.51)
Eastern Sub-Saharan Africa	NCD	29,094.09 (26,121.44–32,144.84)	28,202.61 (25,291.48–31,377.30)	26,890.84 (24,122.00–29,663.60)	25,284.87 (22,594.70–28,173.94)	24,193.61 (21,185.08–27,415.39)	24,242.01 (20,793.93–28,049.56)
Eastern Sub-Saharan Africa	Injuries	7,883.62 (6,666.83–9,144.53)	5,810.10 (5,112.95–6,420.76)	6,452.25 (5,511.12–7,465.43)	4,602.03 (4,162.61–5,075.99)	4,361.66 (3,883.81–5,006.66)	4,305.41 (3,708.41–5,095.94)
Burundi	All causes	111,055.37 (87,596.99–135,087.41)	115,943.90 (100,364.84–130,903.90)	105,024.59 (93,775.95–116,518.84)	75,755.89 (67,743.43–84,646.04)	58,541.51 (49,552.85–69,450.57)	55,603.12 (42,679.76–72,320.35)

Appendix Table 4. Rate of DALYs per 100,000 individuals for all causes and level 1 cause groups by location for 1990 - 2015, both sexes combined.

	Cause	1990	1995	2000	2005	2010	2015
Burundi	Group I	61,480.16 (50,655.87–73,873.54)	65,444.03 (57,505.80–73,756.40)	61,864.17 (56,097.35–68,220.22)	42,902.99 (39,237.35–47,211.36)	29,883.95 (26,091.39–34,643.12)	25,543.24 (19,946.10–32,436.90)
Burundi	NCD	41,296.33 (29,766.21–52,350.28)	40,836.84 (33,015.42–48,403.48)	34,438.29 (28,820.96–40,181.90)	27,243.80 (22,936.29–31,806.90)	24,174.24 (19,370.43–29,800.65)	25,237.90 (18,487.05–34,063.49)
Burundi	Injuries	8,278.89 (5,439.03–11,279.37)	9,663.02 (7,145.57–11,955.43)	8,722.12 (6,765.92–10,933.02)	5,609.11 (4,506.70–6,926.88)	4,483.32 (3,397.54–6,010.32)	4,821.98 (3,385.14–6,870.53)
Comoros	All causes	68,947.98 (54,346.00–88,238.28)	63,801.52 (53,722.66–75,968.12)	55,227.98 (48,527.01–62,495.04)	46,056.71 (40,036.97–52,100.49)	41,906.96 (35,389.08–48,834.06)	40,822.02 (32,195.14–51,838.30)
Comoros	Group I	33,374.88 (28,122.11–40,531.72)	29,721.65 (25,759.72–34,275.23)	25,304.02 (22,277.06–28,589.73)	20,037.66 (17,042.57–23,068.33)	16,782.59 (13,898.86–20,028.07)	15,115.15 (11,877.42–19,276.36)
Comoros	NCD	30,062.64 (21,702.22–40,189.90)	28,917.60 (23,313.35–35,498.25)	25,594.09 (21,817.59–29,683.39)	22,237.17 (18,800.54–25,854.71)	21,727.32 (18,144.00–25,383.83)	22,303.08 (17,133.11–28,479.02)
Comoros	Injuries	5,510.47 (3,808.16–8,014.06)	5,162.28 (3,908.16–6,748.80)	4,329.87 (3,433.38–5,420.56)	3,781.88 (2,985.53–4,682.72)	3,397.04 (2,647.98–4,481.43)	3,403.79 (2,384.77–4,993.24)
Djibouti	All causes	58,427.36 (48,094.69–72,257.96)	59,743.52 (47,231.99–78,001.64)	60,800.23 (45,411.58–84,111.75)	58,955.16 (43,754.48–81,205.71)	54,813.84 (40,328.63–77,371.93)	51,359.82 (37,632.09–72,268.80)
Djibouti	Group I	27,241.67 (23,571.77–32,105.61)	27,961.60 (23,555.21–34,322.23)	29,036.80 (23,450.85–36,763.54)	27,377.64 (22,079.27–34,630.60)	23,611.71 (18,715.73–30,123.02)	20,553.63 (16,003.72–26,512.63)
Djibouti	NCD	25,897.90 (19,825.51–34,219.77)	26,344.15 (19,123.68–36,654.97)	26,489.90 (17,979.75–39,258.28)	26,486.61 (17,950.11–39,294.65)	26,262.35 (17,754.20–39,761.94)	25,983.36 (17,916.50–38,511.39)
Djibouti	Injuries	5,287.79 (3,883.19–6,965.53)	5,437.77 (3,724.90–7,904.81)	5,273.53 (3,335.60–8,601.46)	5,090.92 (3,254.56–8,277.17)	4,939.78 (3,185.25–8,022.54)	4,822.83 (3,094.19–7,939.23)
Eritrea	All causes	84,199.53 (75,757.28–93,385.54)	67,227.05 (57,148.06–78,216.05)	150,525.86 (93,409.06–214,086.08)	61,492.99 (44,584.12–83,625.31)	60,835.19 (43,282.84–86,852.43)	58,979.72 (40,256.27–87,164.91)
Eritrea	Group I	47,031.92 (42,893.15–51,858.11)	36,053.88 (31,649.88–40,930.40)	31,367.19 (25,177.69–38,276.62)	29,283.38 (22,540.92–38,627.93)	27,630.50 (20,391.87–38,750.92)	25,514.99 (18,342.28–36,657.41)
Eritrea	NCD	31,161.73 (26,556.32–36,026.16)	26,279.83 (21,304.12–31,826.68)	25,688.27 (18,372.14–34,194.86)	26,508.46 (17,798.48–37,746.66)	27,478.38 (18,310.90–40,456.67)	27,849.07 (18,505.93–42,351.58)
Eritrea	Injuries	6,005.87 (4,674.45–7,312.38)	4,893.34 (3,712.78–6,362.50)	93,470.39 (38,829.04–155,985.15)	5,701.15 (4,131.07–8,267.21)	5,726.31 (3,903.45–8,925.37)	5,615.66 (3,622.42–8,822.20)
Ethiopia	All causes	110,167.68 (101,424.57–119,656.81)	94,214.66 (87,212.66–101,434.68)	84,120.70 (77,176.92–91,461.00)	71,172.26 (64,910.22–77,935.24)	54,518.92 (46,139.80–64,059.72)	47,099.80 (36,546.70–60,314.21)
Ethiopia	Group I	61,403.25 (56,945.25–66,533.10)	56,958.60 (52,972.40–61,261.43)	50,579.32 (46,733.82–54,475.98)	40,462.27 (37,616.89–43,703.96)	27,007.87 (23,650.80–30,598.41)	20,200.72 (16,532.23–24,917.86)
Ethiopia	NCD	34,790.28 (30,134.81–39,372.65)	30,793.50 (27,072.84–35,065.75)	28,112.44 (24,342.72–32,034.79)	26,078.37 (22,507.69–29,911.74)	23,481.18 (18,734.93–28,857.89)	23,118.07 (17,124.41–30,579.56)
Ethiopia	Injuries	13,974.15 (9,607.37–18,704.24)	6,462.57 (5,111.84–7,679.62)	5,428.93 (4,404.83–6,427.09)	4,631.62 (3,802.56–5,437.44)	4,029.87 (3,095.31–5,129.40)	3,781.00 (2,642.88–5,500.55)
Kenya	All causes	56,744.38 (52,203.63–61,896.46)	66,810.77 (61,591.80–72,348.78)	74,242.33 (68,973.86–80,403.83)	69,444.63 (64,582.85–75,146.85)	54,424.07 (49,545.51–59,898.56)	48,634.39 (43,912.72–53,902.40)
Kenya	Group I	31,411.43 (29,319.35–33,870.16)	40,993.40 (38,505.38–43,923.85)	49,462.99 (46,739.92–52,726.08)	45,569.40 (43,151.92–48,631.02)	30,829.95 (28,552.49–34,079.20)	24,874.33 (22,878.47–27,460.21)
Kenya	NCD	20,968.78 (18,407.33–23,901.51)	21,563.61 (18,771.00–24,444.83)	20,785.39 (18,041.10–23,727.32)	20,092.62 (17,558.68–22,864.44)	19,844.11 (17,299.46–22,486.56)	20,044.99 (17,363.83–22,742.97)
Kenya	Injuries	4,364.17 (3,749.61–5,180.03)	4,253.77 (3,698.09–4,944.77)	3,993.95 (3,505.72–4,590.49)	3,782.61 (3,343.80–4,339.95)	3,750.01 (3,297.27–4,298.39)	3,715.07 (3,214.93–4,280.00)
Madagascar	All causes	73,436.39 (67,693.73–79,141.62)	69,843.00 (64,534.58–75,836.83)	64,297.17 (58,860.45–70,333.39)	58,515.16 (52,260.04–66,303.71)	54,968.83 (45,097.61–66,547.15)	51,942.13 (39,271.30–67,151.36)
Madagascar	Group I	38,338.53 (35,681.05–41,625.91)	36,205.41 (33,173.56–42,199.72)	31,695.12 (28,518.77–38,542.40)	27,033.78 (24,036.61–33,328.13)	23,904.53 (20,005.44–29,405.73)	20,736.85 (16,485.26–26,514.56)
Madagascar	NCD	30,700.53 (27,185.41–34,171.32)	29,629.23 (25,036.67–33,610.79)	28,814.30 (23,161.32–33,125.61)	28,049.43 (22,540.97–33,232.37)	27,712.02 (21,252.20–34,984.22)	27,860.18 (19,897.89–37,560.96)
Madagascar	Injuries	4,397.32 (3,589.62–5,138.56)	4,008.36 (3,017.18–4,801.30)	3,787.75 (2,660.18–4,614.94)	3,431.94 (2,440.90–4,253.79)	3,352.27 (2,377.15–4,411.95)	3,345.10 (2,241.64–4,774.56)

Appendix Table 4. Rate of DALYs per 100,000 individuals for all causes and level 1 cause groups by location for 1990 - 2015, both sexes combined.

	Cause	1990	1995	2000	2005	2010	2015
Malawi	All causes	94,703.59 (86,726.65–103,534.36)	102,163.82 (90,419.11–115,820.09)	109,337.19 (97,139.89–122,876.08)	97,813.58 (86,519.28–109,609.90)	73,763.49 (65,625.15–83,067.54)	58,936.60 (48,442.36–73,003.17)
Malawi	Group I	64,812.12 (60,233.58–70,099.75)	71,920.27 (65,078.39–80,067.68)	78,063.39 (70,372.47–87,269.15)	68,958.05 (62,184.83–76,185.20)	47,125.29 (43,123.56–51,635.79)	32,904.16 (28,357.56–38,350.90)
Malawi	NCD	24,775.40 (20,717.79–29,273.87)	25,414.28 (19,781.41–32,360.24)	26,529.68 (20,310.90–33,914.18)	24,789.16 (19,537.12–31,474.60)	22,967.93 (18,409.37–28,149.08)	22,426.90 (16,579.89–30,020.19)
Malawi	Injuries	5,116.08 (3,968.01–6,339.79)	4,829.27 (3,602.19–6,385.81)	4,744.12 (3,563.18–6,210.35)	4,066.37 (3,082.66–5,256.29)	3,670.26 (2,818.84–4,629.87)	3,605.55 (2,587.19–5,021.15)
Mozambique	All causes	86,855.16 (80,871.26–93,722.84)	83,085.73 (76,727.92–89,843.64)	84,276.52 (76,764.15–92,370.24)	82,714.19 (72,817.13–93,744.27)	78,201.27 (65,855.33–91,554.56)	69,247.64 (54,693.47–87,939.03)
Mozambique	Group I	55,523.86 (51,959.58–59,769.74)	53,273.49 (49,737.61–57,578.54)	53,064.11 (49,083.95–57,964.87)	53,145.60 (47,607.61–59,271.25)	47,394.46 (41,876.56–53,447.82)	38,391.69 (32,457.25–45,477.94)
Mozambique	NCD	24,857.52 (21,573.98–28,636.21)	24,571.28 (21,006.29–28,136.05)	25,902.09 (21,526.15–30,224.66)	25,005.16 (19,868.94–30,884.30)	26,112.49 (19,597.01–33,536.22)	26,277.51 (18,216.51–36,059.08)
Mozambique	Injuries	6,473.78 (5,408.26–7,769.31)	5,240.96 (4,349.67–6,247.02)	5,310.33 (4,369.37–6,244.98)	4,563.43 (3,448.93–5,729.79)	4,694.32 (3,260.78–6,268.23)	4,578.44 (2,930.01–6,878.89)
Rwanda	All causes	89,183.20 (80,876.73–98,353.86)	120,104.25 (110,028.09–129,896.14)	94,605.55 (86,466.90–102,840.47)	62,265.62 (55,981.27–69,410.01)	48,613.01 (41,084.22–57,142.23)	46,205.20 (36,690.11–59,843.20)
Rwanda	Group I	46,595.04 (42,202.46–50,988.20)	62,742.14 (57,736.08–68,119.01)	54,478.54 (50,347.40–58,722.75)	34,167.67 (31,384.23–37,220.49)	21,962.52 (19,109.82–25,216.77)	18,844.80 (15,546.07–23,235.71)
Rwanda	NCD	32,873.21 (28,108.30–38,172.46)	41,311.20 (35,731.18–47,359.82)	31,054.43 (26,373.06–36,075.06)	21,904.22 (18,379.48–25,878.49)	21,247.41 (17,202.93–26,007.84)	22,120.29 (16,819.45–29,875.15)
Rwanda	Injuries	9,714.95 (7,578.00–12,314.16)	16,050.90 (11,697.87–20,496.20)	9,072.58 (7,315.70–11,203.58)	6,193.73 (4,891.17–7,819.39)	5,403.07 (4,089.19–7,100.07)	5,240.12 (3,789.40–7,449.60)
Somalia	All causes	95,904.20 (63,680.88–149,897.47)	92,389.61 (58,862.10–144,893.74)	90,141.71 (56,283.71–143,429.73)	85,866.73 (51,808.72–144,815.17)	87,384.04 (54,249.16–150,015.19)	79,062.30 (48,427.78–138,904.86)
Somalia	Group I	54,507.20 (39,432.45–79,428.94)	53,696.45 (37,606.06–79,520.12)	52,676.09 (35,883.90–79,279.27)	49,563.43 (32,641.24–79,473.88)	48,870.68 (31,967.98–79,545.25)	41,254.48 (26,681.23–70,696.23)
Somalia	NCD	31,565.31 (16,885.66–54,553.34)	30,968.94 (16,218.36–52,403.35)	30,074.23 (15,707.44–51,140.58)	29,195.79 (15,224.54–51,646.54)	29,867.66 (15,882.24–55,832.87)	29,396.77 (15,729.93–54,072.25)
Somalia	Injuries	9,831.69 (6,089.53–15,904.15)	7,724.21 (4,388.75–13,318.23)	7,391.40 (3,868.42–13,509.21)	7,107.51 (3,765.38–13,521.92)	8,645.70 (5,237.56–15,650.91)	8,411.05 (5,092.27–14,772.10)
South Sudan	All causes	89,514.14 (63,744.07–135,733.81)	83,961.85 (57,463.74–134,779.57)	78,724.02 (52,410.36–132,479.57)	75,884.87 (49,224.72–133,164.81)	73,432.05 (48,009.29–135,731.63)	72,823.16 (47,105.50–130,088.09)
South Sudan	Group I	54,718.36 (42,633.54–75,831.28)	50,631.39 (38,112.12–75,135.34)	47,732.51 (34,377.52–74,016.75)	45,550.04 (31,690.38–73,906.47)	42,268.53 (29,417.29–72,333.96)	39,223.00 (26,570.31–66,186.00)
South Sudan	NCD	28,074.18 (16,185.19–48,953.76)	27,378.20 (15,521.80–49,090.35)	25,626.01 (14,721.33–47,156.45)	25,166.95 (14,335.78–47,837.13)	25,716.60 (14,699.80–52,319.35)	26,306.21 (15,011.02–51,563.23)
South Sudan	Injuries	6,721.59 (3,916.12–11,387.22)	5,952.26 (3,152.69–11,060.08)	5,365.49 (2,826.51–10,659.58)	5,167.87 (2,657.36–11,051.45)	5,446.92 (2,817.11–12,391.27)	7,293.94 (4,393.54–13,875.89)
Tanzania	All causes	74,635.69 (68,453.84–80,883.80)	78,662.01 (71,466.76–85,705.50)	77,312.98 (69,091.79–85,568.61)	68,940.51 (60,856.21–77,944.25)	57,332.06 (47,248.62–69,725.04)	50,165.28 (38,930.40–66,871.37)
Tanzania	Group I	44,808.27 (41,845.01–47,970.01)	49,552.48 (45,891.37–53,825.30)	49,959.40 (45,715.35–54,755.95)	43,167.12 (38,940.96–47,609.88)	31,643.76 (27,383.17–36,467.93)	23,575.09 (19,192.03–29,391.17)
Tanzania	NCD	25,054.45 (21,576.90–28,700.61)	24,821.96 (20,805.54–28,793.89)	23,582.84 (19,417.91–28,053.20)	22,224.89 (18,092.08–27,164.92)	22,129.60 (16,674.07–28,919.63)	22,970.24 (16,770.27–32,669.92)
Tanzania	Injuries	4,772.98 (3,964.93–5,599.04)	4,287.57 (3,488.08–5,165.44)	3,770.74 (3,011.04–4,568.02)	3,548.50 (2,811.07–4,484.37)	3,558.70 (2,636.70–5,017.68)	3,619.96 (2,530.49–5,498.78)
Uganda	All causes	89,484.92 (80,168.07–98,827.73)	98,303.88 (87,653.51–108,977.74)	95,751.70 (85,990.18–104,297.08)	80,269.82 (71,857.60–89,413.57)	66,659.71 (56,289.25–77,660.63)	56,970.88 (43,861.26–72,711.13)
Uganda	Group I	55,992.24 (51,141.73–62,122.31)	63,749.52 (57,337.78–70,472.60)	60,087.34 (55,562.03–64,564.11)	45,895.41 (42,518.85–49,822.94)	34,887.71 (31,006.06–38,985.48)	25,599.83 (21,266.69–30,528.30)
Uganda	NCD	27,877.55 (22,137.64–32,686.86)	28,875.05 (22,395.91–34,731.75)	29,590.53 (23,469.14–34,878.95)	28,719.41 (23,693.52–34,007.77)	26,592.54 (20,461.59–33,087.01)	26,451.90 (18,626.18–35,737.44)

Appendix Table 4. Rate of DALYs per 100,000 individuals for all causes and level 1 cause groups by location for 1990 - 2015, both sexes combined.

	Cause	1990	1995	2000	2005	2010	2015
Uganda	Injuries	5,615.14 (4,436.06–6,821.27)	5,679.31 (4,378.51–7,065.73)	6,073.83 (4,832.99–7,306.13)	5,654.99 (4,673.27–6,851.32)	5,179.45 (3,789.12–6,608.16)	4,919.15 (3,248.47–7,328.64)
Zambia	All causes	84,098.98 (76,204.37–91,970.11)	103,319.92 (93,447.20–114,101.89)	113,576.22 (102,749.26–125,174.02)	105,396.14 (95,985.98–115,732.42)	81,958.84 (71,717.13–93,358.42)	69,974.43 (56,070.46–87,435.56)
Zambia	Group I	52,862.09 (48,515.75–58,403.06)	67,788.77 (61,176.05–76,143.83)	76,089.23 (68,453.68–84,375.91)	66,288.83 (60,614.85–72,700.63)	44,260.38 (39,498.37–49,317.25)	34,153.55 (28,897.80–40,270.00)
Zambia	NCD	26,032.95 (21,739.42–29,941.12)	29,885.55 (24,935.97–35,008.77)	31,662.88 (26,839.69–36,556.71)	33,000.29 (28,476.15–38,069.35)	31,820.88 (26,648.17–37,853.04)	30,374.75 (22,825.72–40,036.92)
Zambia	Injuries	5,203.94 (4,104.53–6,209.63)	5,645.59 (4,449.03–6,793.65)	5,824.12 (4,677.88–7,015.23)	6,107.01 (5,013.59–7,357.57)	5,877.58 (4,721.90–7,344.22)	5,446.13 (3,563.97–7,625.10)
Central Sub-Saharan Africa	All causes	85,050.74 (73,693.53–98,731.55)	84,920.90 (75,000.42–98,256.78)	83,692.17 (74,433.58–95,764.80)	76,663.20 (67,634.81–88,826.36)	69,795.21 (60,448.64–82,789.48)	63,600.40 (52,915.13–77,566.35)
Central Sub-Saharan Africa	Group I	49,419.75 (43,554.80–56,729.95)	50,862.31 (45,206.59–58,232.43)	49,880.55 (44,577.45–57,673.73)	44,652.35 (39,777.76–51,976.87)	37,801.67 (33,007.32–45,243.54)	31,212.05 (26,173.17–38,843.27)
Central Sub-Saharan Africa	NCD	29,106.01 (23,329.87–36,111.73)	28,255.85 (23,184.93–34,464.63)	27,889.53 (22,984.58–34,501.24)	26,989.22 (22,054.60–33,640.01)	27,116.05 (22,174.08–33,516.19)	27,585.71 (21,645.12–35,206.42)
Central Sub-Saharan Africa	Injuries	6,524.98 (4,720.90–8,576.98)	5,802.73 (4,200.64–7,669.60)	5,922.08 (4,464.80–7,534.63)	5,021.63 (3,808.20–6,495.11)	4,877.49 (3,682.30–6,631.56)	4,802.65 (3,554.49–6,546.88)
Angola	All causes	98,226.86 (64,387.60–149,590.50)	90,303.73 (58,875.12–147,432.57)	85,680.81 (56,783.32–135,546.03)	76,468.52 (50,012.15–129,212.31)	69,136.38 (44,707.68–124,916.99)	63,211.73 (40,011.34–119,150.29)
Angola	Group I	51,577.09 (36,857.81–73,167.97)	47,501.43 (33,658.54–72,193.96)	45,049.38 (32,620.41–66,018.22)	38,375.82 (27,229.71–59,116.78)	31,138.88 (21,566.98–49,925.95)	26,157.22 (17,747.38–44,543.86)
Angola	NCD	35,729.73 (18,591.15–59,315.47)	34,271.51 (18,065.50–60,202.83)	32,624.24 (17,773.24–55,893.87)	31,526.38 (17,382.73–57,815.94)	31,437.75 (17,922.98–61,154.62)	31,063.68 (17,817.99–62,950.90)
Angola	Injuries	10,920.05 (6,029.28–17,928.26)	8,530.78 (4,409.49–15,370.75)	8,007.18 (4,195.61–14,133.15)	6,566.32 (3,463.96–12,909.12)	6,559.75 (3,626.41–13,852.23)	5,990.83 (3,277.78–13,500.28)
Central African Republic	All causes	99,861.09 (90,325.21–109,727.53)	106,723.07 (87,445.73–124,384.02)	112,871.23 (87,009.25–142,068.68)	112,253.84 (83,490.59–145,907.34)	102,184.07 (75,351.63–134,456.55)	95,671.88 (68,566.30–128,617.43)
Central African Republic	Group I	56,964.85 (51,351.21–62,907.58)	63,647.16 (54,713.27–73,738.59)	69,391.57 (56,629.20–84,127.13)	68,820.57 (54,984.12–84,790.96)	58,193.43 (46,206.77–73,475.10)	51,087.67 (38,907.19–66,032.17)
Central African Republic	NCD	34,999.54 (29,454.78–40,323.88)	35,506.00 (26,603.45–44,247.95)	35,823.85 (23,914.29–48,447.01)	35,904.70 (23,086.28–50,229.19)	36,194.49 (23,389.07–51,108.79)	36,487.74 (23,282.71–51,574.26)
Central African Republic	Injuries	7,896.70 (5,693.75–9,999.26)	7,569.91 (4,745.79–10,526.52)	7,655.81 (4,281.50–11,994.97)	7,528.58 (4,109.48–12,145.51)	7,796.14 (4,221.23–12,509.54)	8,096.47 (4,519.45–13,123.41)
Congo	All causes	80,293.48 (68,984.31–92,238.66)	85,733.28 (75,446.34–95,826.29)	85,465.91 (76,142.95–95,230.00)	71,575.15 (62,956.79–81,288.53)	60,891.32 (51,562.24–72,935.57)	58,141.07 (44,611.78–75,755.32)
Congo	Group I	38,198.07 (33,141.10–44,107.38)	43,269.46 (38,065.33–48,523.53)	44,769.33 (40,107.26–49,749.47)	37,327.62 (33,503.26–42,104.16)	27,623.81 (24,026.64–32,464.90)	25,163.79 (20,021.37–31,608.47)
Congo	NCD	35,924.20 (29,465.11–42,712.40)	36,417.61 (30,588.69–42,109.57)	34,022.68 (28,303.72–40,098.07)	29,677.60 (24,680.60–35,033.11)	29,011.77 (23,164.85–35,721.72)	28,982.63 (20,741.29–39,341.29)
Congo	Injuries	6,171.20 (4,540.31–7,955.40)	6,046.21 (4,448.75–7,577.21)	6,673.90 (4,966.01–8,275.82)	4,569.93 (3,541.51–5,794.19)	4,255.75 (3,272.95–5,604.09)	3,994.65 (2,771.96–5,887.33)
Democratic Republic of the Congo	All causes	80,048.08 (67,893.46–93,742.21)	81,586.90 (72,959.49–91,593.28)	80,847.99 (73,001.53–90,376.39)	74,667.01 (67,184.50–84,095.00)	68,671.18 (60,261.82–79,868.13)	62,333.05 (50,613.11–76,320.48)
Democratic Republic of the Congo	Group I	49,239.80 (42,565.63–58,088.36)	51,782.79 (46,040.11–59,934.01)	50,570.93 (45,117.71–59,392.46)	45,700.88 (40,713.95–54,289.12)	39,515.44 (34,496.40–47,946.86)	32,232.14 (26,102.05–41,287.62)
Democratic Republic of the Congo	NCD	25,809.69 (20,152.15–32,357.55)	25,056.67 (20,650.64–29,458.96)	25,209.15 (21,007.97–29,315.99)	24,618.14 (20,373.46–29,145.37)	24,994.19 (20,209.43–29,829.72)	25,848.06 (19,603.05–32,539.44)
Democratic Republic of the Congo	Injuries	4,998.59 (3,402.58–6,679.70)	4,747.44 (3,562.47–6,035.23)	5,067.91 (3,956.85–6,285.94)	4,347.99 (3,427.47–5,368.68)	4,161.55 (3,253.85–5,284.96)	4,252.85 (3,026.64–5,748.04)
Equatorial Guinea	All causes	97,040.12 (59,329.72–152,339.63)	97,325.91 (61,539.67–152,597.03)	81,941.65 (54,647.99–130,528.04)	72,563.54 (50,588.84–121,500.56)	65,465.22 (45,788.87–111,795.09)	58,721.35 (40,625.64–107,207.42)
Equatorial Guinea	Group I	49,639.88 (34,596.19–72,939.32)	51,892.92 (36,881.11–75,169.02)	42,445.45 (31,227.47–60,098.01)	36,841.68 (26,825.19–54,546.18)	31,099.19 (22,166.56–46,230.95)	26,218.89 (18,534.54–41,277.60)

Appendix Table 4. Rate of DALYs per 100,000 individuals for all causes and level 1 cause groups by location for 1990 - 2015, both sexes combined.

	Cause	1990	1995	2000	2005	2010	2015
Equatorial Guinea	NCD	38,775.97 (19,879.80–64,739.53)	37,360.99 (19,116.95–63,397.47)	32,535.25 (17,994.62–58,836.51)	28,856.84 (17,738.34–54,730.34)	28,315.30 (18,401.82–54,518.45)	27,352.81 (18,070.05–53,991.25)
Equatorial Guinea	Injuries	8,624.27 (4,014.38–16,144.73)	8,072.00 (3,764.17–15,380.79)	6,960.95 (3,173.33–13,659.95)	6,865.01 (3,818.63–13,675.94)	6,050.73 (3,240.81–12,515.60)	5,149.66 (2,923.59–11,413.87)
Gabon	All causes	63,967.90 (57,041.31–71,243.64)	63,028.80 (56,201.33–71,079.33)	64,367.78 (57,623.24–72,426.43)	62,235.63 (54,943.18–70,732.89)	55,187.89 (47,240.92–64,298.40)	48,461.65 (38,952.54–62,066.84)
Gabon	Group I	27,407.18 (24,564.33–30,564.33)	27,177.71 (24,365.34–30,629.44)	30,413.37 (26,988.73–34,186.94)	29,390.25 (25,908.95–33,400.93)	25,372.30 (21,986.63–29,705.96)	19,137.55 (15,604.41–24,451.15)
Gabon	NCD	30,425.36 (26,062.55–34,894.34)	30,117.84 (25,594.77–34,735.18)	29,114.93 (24,856.17–34,154.77)	28,325.14 (24,056.79–33,741.26)	25,737.43 (20,922.96–31,456.99)	25,374.49 (19,517.44–33,986.07)
Gabon	Injuries	6,135.36 (4,816.34–7,426.93)	5,733.26 (4,563.71–7,075.78)	4,839.48 (3,745.80–6,097.24)	4,520.23 (3,433.47–5,907.55)	4,078.17 (3,045.29–5,399.89)	3,949.61 (2,766.26–5,982.71)

Appendix Table 5. Socio-Demographic Index values for all estimated GBD 2015 locations, 1980–2015

Location	1980	1981	1982	1983	1984	1985	1986	1987	1988	1989	1990	1991	1992	1993	1994	1995	1996	1997	1998	1999	2000	2001	2002	2003	2004	2005	2006	2007	2008	2009	2010	2011	2012	2013	2014	2015
Global	0.4199	0.4256	0.4311	0.4365	0.4424	0.4489	0.4551	0.4615	0.4681	0.4745	0.4811	0.4877	0.4944	0.5011	0.5078	0.5146	0.5214	0.5279	0.5340	0.5398	0.5456	0.5512	0.5568	0.5625	0.5685	0.5748	0.5815	0.5886	0.5953	0.6015	0.6079	0.6143	0.6204	0.6265	0.6324	0.6381
Southeast Asia, East Asia, and Oceania	0.3345	0.3444	0.3544	0.3636	0.3744	0.3865	0.3973	0.4081	0.4187	0.4284	0.4380	0.4483	0.4596	0.4715	0.4836	0.4957	0.5074	0.5185	0.5281	0.5370	0.5454	0.5535	0.5618	0.5698	0.5782	0.5869	0.5958	0.6052	0.6138	0.6219	0.6301	0.6380	0.6458	0.6533	0.6605	0.6672
East Asia	0.3249	0.3346	0.3447	0.3538	0.3650	0.3783	0.3901	0.4019	0.4134	0.4237	0.4338	0.4449	0.4572	0.4703	0.4835	0.4967	0.5092	0.5211	0.5316	0.5413	0.5504	0.5592	0.5681	0.5768	0.5858	0.5951	0.6047	0.6149	0.6240	0.6327	0.6413	0.6495	0.6576	0.6653	0.6725	0.6792
China	0.3160	0.3258	0.3361	0.3452	0.3565	0.3700	0.3819	0.3939	0.4057	0.4161	0.4265	0.4377	0.4502	0.4636	0.4773	0.4909	0.5038	0.5161	0.5268	0.5368	0.5463	0.5553	0.5644	0.5733	0.5826	0.5921	0.6019	0.6124	0.6216	0.6305	0.6393	0.6477	0.6561	0.6639	0.6712	0.6780
Anhui	0.2770	0.2948	0.3045	0.3140	0.3279	0.3402	0.3532	0.3665	0.3721	0.3790	0.3881	0.3984	0.4118	0.4258	0.4382	0.4502	0.4615	0.4709	0.4798	0.4888	0.4985	0.5075	0.5177	0.5250	0.5318	0.5395	0.5472	0.5558	0.5627	0.5700	0.5793	0.5868	0.5916	0.5983	0.6047	0.6110
Beijing	0.5106	0.5177	0.5194	0.5281	0.5328	0.5418	0.5529	0.5649	0.5813	0.5979	0.6083	0.6240	0.6331	0.6429	0.6529	0.6633	0.6733	0.6828	0.6917	0.7003	0.7089	0.7183	0.7280	0.7380	0.7482	0.7587	0.7697	0.7809	0.7915	0.8019	0.8122	0.8225	0.8325	0.8423	0.8516	0.8605
Chongqing	0.2739	0.2896	0.3005	0.2931	0.2987	0.3053	0.3373	0.3653	0.3735	0.3864	0.4016	0.4080	0.4194	0.4338	0.4490	0.4630	0.4773	0.4894	0.5020	0.5151	0.5268	0.5359	0.5440	0.5492	0.5581	0.5688	0.5753	0.5837	0.5914	0.5994	0.6089	0.6116	0.6144	0.6203	0.6257	0.6309
Fujian	0.3017	0.3122	0.3218	0.3318	0.3412	0.3577	0.3780	0.3979	0.4094	0.4139	0.4253	0.4411	0.4563	0.4734	0.4892	0.5050	0.5228	0.5366	0.5497	0.5604	0.5701	0.5781	0.5880	0.5969	0.6063	0.6158	0.6247	0.6361	0.6452	0.6548	0.6663	0.6742	0.6797	0.6807	0.6798	0.6770
Gansu	0.2973	0.3077	0.3142	0.3208	0.3279	0.3406	0.3522	0.3642	0.3728	0.3795	0.3927	0.3988	0.4061	0.4140	0.4231	0.4325	0.4469	0.4572	0.4666	0.4751	0.4832	0.4907	0.4978	0.5055	0.5131	0.5200	0.5264	0.5335	0.5402	0.5464	0.5577	0.5630	0.5697	0.5771	0.5841	0.5908
Guangdong	0.2644	0.2774	0.2982	0.2651	0.2380	0.3139	0.3240	0.3388	0.3700	0.3936	0.4041	0.4200	0.4441	0.4702	0.4917	0.5086	0.5274	0.5458	0.5609	0.5765	0.5877	0.5956	0.6066	0.6133	0.6244	0.6359	0.6454	0.6556	0.6671	0.6772	0.6857	0.6996	0.7046	0.7165	0.7284	0.7402
Guangxi	0.2408	0.2522	0.2642	0.2819	0.2830	0.2995	0.3177	0.3364	0.3511	0.3688	0.3816	0.3823	0.3958	0.4095	0.4244	0.4398	0.4541	0.4663	0.4763	0.4870	0.4960	0.5046	0.5142	0.5211	0.5327	0.5416	0.5503	0.5615	0.5702	0.5798	0.5917	0.6011	0.6098	0.6195	0.6291	0.6385
Guizhou	0.1644	0.1827	0.1954	0.2076	0.2265	0.2523	0.2667	0.2805	0.2909	0.3098	0.3175	0.3234	0.3325	0.3439	0.3561	0.3701	0.3821	0.3915	0.4010	0.4090	0.4191	0.4277	0.4350	0.4468	0.4569	0.4679	0.4773	0.4882	0.4949	0.5012	0.5108	0.5187	0.5262	0.5339	0.5415	0.5489
Hainan	0.3372	0.3483	0.3562	0.3515	0.3557	0.3606	0.3839	0.4058	0.4126	0.4153	0.4243	0.4325	0.4456	0.4585	0.4715	0.4845	0.4970	0.5090	0.5205	0.5320	0.5416	0.5511	0.5594	0.5689	0.5778	0.5865	0.5953	0.6050	0.6137	0.6224	0.6311	0.6389	0.6493	0.6588	0.6683	0.6774
Hebei	0.3012	0.3000	0.3102	0.3194	0.3271	0.3358	0.3373	0.3391	0.3550	0.3712	0.3842	0.4054	0.4212	0.4335	0.4505	0.4678	0.4816	0.4948	0.5050	0.5128	0.5236	0.5346	0.5400	0.5481	0.5540	0.5568	0.5649	0.5715	0.5827	0.5923	0.5971	0.6056	0.6192	0.6289	0.6388	0.6485
Heilongjiang	0.4107	0.4211	0.4243	0.4322	0.4416	0.4501	0.4553	0.4604	0.4713	0.4766	0.4880	0.4964	0.5026	0.5125	0.5239	0.5369	0.5479	0.5567	0.5652	0.5746	0.5823	0.5898	0.5974	0.6046	0.6116	0.6186	0.6261	0.6340	0.6412	0.6486	0.6557	0.6627	0.6693	0.6756	0.6816	0.6870
Henan	0.2599	0.2737	0.2863	0.3021	0.3261	0.3410	0.3521	0.3619	0.3732	0.3806	0.3907	0.4066	0.4202	0.4378	0.4525	0.4675	0.4809	0.4928	0.5030	0.5126	0.5218	0.5308	0.5417	0.5516	0.5630	0.5746	0.5849	0.5976	0.6073	0.6169	0.6265	0.6349	0.6438	0.6433	0.6401	0.6338
Hong Kong Special Administrative Region of China	0.6812	0.6920	0.7014	0.7095	0.7179	0.7255	0.7341	0.7432	0.7521	0.7599	0.7669	0.7746	0.7823	0.7901	0.7980	0.8055	0.8115	0.8161	0.8194	0.8224	0.8257	0.8294	0.8329	0.8364	0.8404	0.8449	0.8495	0.8545	0.8589	0.8622	0.8654	0.8680	0.8702	0.8723	0.8743	0.8762
Hubei	0.3301	0.3433	0.3583	0.3660	0.3777	0.3887	0.3979	0.4074	0.4212	0.4283	0.4388	0.4451	0.4578	0.4668	0.4832	0.4988	0.5107	0.5228	0.5367	0.5471	0.5583	0.5668	0.5752	0.5840	0.5929	0.6019	0.6099	0.6191	0.6275	0.6346	0.6394	0.6459	0.6519	0.6592	0.6662	0.6730
Hunan	0.3095	0.3186	0.3209	0.3331	0.3484	0.3595	0.3692	0.3792	0.3883	0.4020	0.4120	0.4233	0.4406	0.4574	0.4695	0.4825	0.4944	0.5045	0.5142	0.5235	0.5308	0.5375	0.5456	0.5525	0.5611	0.5718	0.5806	0.5903	0.5970	0.6040	0.6108	0.6169	0.6251	0.6340	0.6426	0.6510
Inner Mongolia	0.3320	0.3421	0.3502	0.3683	0.3758	0.3954	0.4090	0.4226	0.4305	0.4430	0.4500	0.4648	0.4738	0.4834	0.4960	0.5122	0.5276	0.5410	0.5537	0.5660	0.5779	0.5894	0.6015	0.6104	0.6177	0.6237	0.6323	0.6407	0.6506	0.6598	0.6685	0.6772	0.6852	0.6945	0.7033	0.7115
Jiangsu	0.3724	0.3806	0.3916	0.4044	0.4194	0.4297	0.4429	0.4563	0.4640	0.4652	0.4762	0.4889	0.5012	0.5168	0.5296	0.5446	0.5569	0.5683	0.5787	0.5881	0.5974	0.6069	0.6157	0.6252	0.6342	0.6447	0.6545	0.6655	0.6756	0.6844	0.6925	0.7013	0.7117	0.7207	0.7293	0.7375
Jiangxi	0.3010	0.3100	0.3222	0.3338	0.3432	0.3554	0.3685	0.3821	0.3922	0.3962	0.4046	0.4160	0.4287	0.4382	0.4528	0.4652	0.4801	0.4900	0.5007	0.5096	0.5184	0.5265	0.5353	0.5443	0.5539	0.5615	0.5698	0.5790	0.5873	0.5954	0.6036	0.6111	0.6202	0.6295	0.6386	0.6476
Jilin	0.3815	0.3897	0.4007	0.4116	0.4225	0.4323	0.4430	0.4538	0.4615	0.4699	0.4803	0.4902	0.5021	0.5143	0.5288	0.5427	0.5550	0.5658	0.5763	0.5871	0.5964	0.6055	0.6147	0.6237	0.6326	0.6417	0.6513	0.6615	0.6709	0.6802	0.6882	0.6982	0.7068	0.7150	0.7228	0.7300
Liaoning	0.4291	0.4406	0.4443	0.4539	0.4686	0.4730	0.4822	0.4919	0.5010	0.5128	0.5194	0.5332	0.5398	0.5498	0.5604	0.5703	0.5804	0.5896	0.5987	0.6083	0.6172	0.6255	0.6340	0.6423	0.6507	0.6591	0.6680	0.6772	0.6860	0.6945	0.7030	0.7113	0.7194	0.7272	0.7346	0.7415
Macao Special Administrative Region of China	0.6366	0.6438	0.6507	0.6569	0.6640	0.6715	0.6798	0.6902	0.7014	0.7122	0.7228	0.7317	0.7412	0.7505	0.7593	0.7678	0.7755	0.7827	0.7888	0.7943	0.7986	0.8027	0.8072	0.8129	0.8211	0.8294	0.8386	0.8483	0.8569	0.8635	0.8709	0.8726	0.8742	0.8757	0.8771	0.8785
Ningxia	0.3169	0.3372	0.3447	0.3577	0.3627	0.3754	0.3860	0.3976	0.4056	0.4217	0.4325	0.4415	0.4545	0.4671	0.4784	0.4900	0.5019	0.5111	0.5214	0.5296	0.5377	0.5449	0.5519	0.5610	0.5689	0.5772	0.5865	0.5979	0.6075	0.6149	0.6229	0.6310	0.6407	0.6482	0.6556	0.6630
Qinghai	0.3005	0.3151	0.3245	0.3308	0.3373	0.3501	0.3575	0.3657	0.3808	0.3903	0.3924	0.3970	0.4055	0.4188	0.4253	0.4341	0.4436	0.4505	0.4583	0.4649	0.4711	0.4764	0.4834	0.4914	0.5000	0.5083	0.5162	0.5246	0.5326	0.5388	0.5431	0.5495	0.5567	0.5594	0.5619	0.5644
Shaanxi	0.3076	0.3161	0.3242	0.3336	0.3413	0.3534	0.3679	0.3827	0.3923	0.4008	0.4093	0.4216	0.4327	0.4473	0.4601	0.4755	0.4897	0.5023	0.5129	0.5240	0.5343	0.5450	0.5541	0.5625	0.5724	0.5834	0.5927	0.6034	0.6131	0.6227	0.6339	0.6423	0.6508	0.6560	0.6604	0.6641
Shandong	0.3279	0.3385	0.3532	0.3664	0.3777	0.3940	0.4014	0.4073	0.4303	0.4439	0.4522	0.4640	0.4828	0.4969	0.5113	0.5235	0.5346	0.5440	0.5533	0.5632	0.5711	0.5789	0.5874	0.5958	0.6027	0.6136	0.6254	0.6382	0.6479	0.6559	0.6651	0.6740	0.6828	0.6926	0.7020	0.7110
Shanghai	0.5528	0.5550	0.5590	0.5615	0.5661	0.5713	0.5809	0.5913	0.6010	0.6109	0.6271	0.6383	0.6463	0.6547	0.6631	0.6717	0.6800	0.6879	0.6952	0.7023	0.7095	0.7178	0.7263	0.7355	0.7449	0.7546	0.7645	0.7744	0.7839	0.7931	0.8023	0.8117	0.8209	0.8299	0.8387	0.8472
Shanxi	0.3492	0.3544	0.3617	0.3735	0.3846	0.3945	0.4062	0.4185	0.4257	0.4374	0.4429	0.4487	0.4620	0.4783	0.4905	0.5036	0.5154	0.5259	0.5356	0.5441	0.5547	0.5652	0.5737	0.5834	0.5922	0.6019	0.6124	0.6230	0.6321	0.6423	0.6503	0.6589	0.6676	0.6765	0.6851	0.6935
Sichuan	0.2353	0.2335	0.2437	0.2598	0.2784	0.2689	0.2859	0.3047	0.3123	0.3280	0.3413	0.3550	0.3577	0.3641	0.3741	0.3819	0.3948	0.4293	0.4408	0.4492	0.4589	0.4677	0.4757	0.4884	0.4970	0.5042	0.5158	0.5247	0.5301	0.5395	0.5488	0.5467	0.5539	0.5565	0.5587	0.5611
Tianjin	0.4934	0.4956	0.4993	0.5020	0.5099	0.5204	0.5346	0.5491	0.5582	0.5692	0.5793	0.5931	0.6000	0.6153	0.6257	0.6381	0.6491	0.6584	0.6672	0.6758	0.6843	0.6940	0.7039	0.7139	0.7240	0.7342	0.7448	0.7558	0.7662	0.7764	0.7865	0.7958	0.8062	0.8159	0.8250	0.8337
Tibet	0.2045	0.2114	0.2176	0.2074	0.2150	0.2190	0.2354	0.2509	0.2546	0.2631	0.2732	0.2768	0.2830	0.2855	0.2955	0.3038	0.3118	0.3184	0.3242	0.3296	0.3374	0.3443	0.3484	0.3561	0.3624	0.3675	0.3742	0.3824	0.3897	0.3952	0.3984	0.4034	0.4084	0.4142	0.4199	0.4253
Xinjiang	0.3267	0.3382	0.3462	0.3477	0.3527	0.3596	0.3721	0.3856	0.4132	0.4071	0.4195	0.4282	0.4417	0.4573	0.4723	0.4883	0.4999	0.5103	0.5209	0.5324	0.5422	0.5508	0.5597	0.5672	0.5748	0.5802	0.5890	0.5946	0.6047	0.6127	0.6204	0.6304	0.6384	0.6460	0.6534	0.6605
Yunnan	0.2386	0.2436	0.2558	0.2544	0.2714	0.2919	0.3050	0.3186	0.3271	0.3417	0.3530	0.3612	0.3719	0.3815	0.3944	0.4080	0.4197	0.4294	0.4401	0.4491	0.4569	0.4632	0.4709	0.4796	0.4911	0.5011	0.5131	0.5226	0.5320	0.5399	0.5456	0.5532	0.5616	0.5690	0.5763	0.5835
Zhejiang	0.3510	0.3602	0.3709	0.3797	0.3988	0.4124	0.4268	0.4412	0.4544	0.4667	0.4789	0.4874	0.4971	0.5122	0.5265	0.5407	0.5548	0.5669	0.5777	0.5876	0.5969	0.6064	0.6161	0.6267	0.6344	0.6428	0.6558	0.6671	0.6783	0.6883	0.6966	0.7087	0.7173	0.7274	0.7372	0.7466
North Korea	0.5315	0.5362	0.5411	0.5463	0.5521	0.5581	0.5636	0.5689	0.5734	0.5776	0.5817	0.5862	0.5892	0.5923	0.5913	0.5880	0.5828	0.5767	0.5704	0.5641	0.5579	0.5524	0.5479	0.5449	0.5442	0.5449	0.5466	0.5486	0.5507	0.5521	0.5538	0.5558	0.5580	0.5603	0.5627	0.5652
Taiwan	0.6114	0.6199	0.6299	0.6413	0.6516	0.6633	0.6772	0.6848	0.6884	0.7015	0.7059	0.7168	0.7246	0.7314	0.7391	0.7456	0.7526	0.7590	0.7749	0.7782	0.7799	0.7939	0.8011	0.8094	0.8159	0.8228	0.8275	0.8328	0.8384	0.8430	0.8485	0.8512	0.8496	0.8590	0.8613	0.8648
Southeast Asia	0.3610	0.3711	0.3807	0.3902	0.3995	0.4084	0.4169	0.4252	0.4331	0.4413	0.4496	0.4580	0.4667	0.4756	0.4849	0.4945	0.5042	0.5133	0.5209	0.5279	0.5349	0.5417	0.5484	0.5552	0.5623	0.5696	0.5773	0.5851	0.5928	0.6000	0.6072	0.6146	0.6220	0.6294	0.6365	0.6436
Cambodia	0.2063	0.2041	0.2026	0.2025	0.2042	0.2076	0.2105	0.2169	0.2253	0.2337	0.2412	0.2502	0.2591	0.2669	0.2744	0.2824	0.2899	0.2978	0.3060	0.3159	0.3265	0.3372	0.3481	0.3590	0.3703	0.3822	0.3946	0.4069	0.4187	0.4287	0.4383	0.4482	0.4582	0.4682	0.4775	0.4861
Indonesia	0.3612	0.3726	0.3834	0.3940	0.4049	0.4154	0.4257	0.4359	0.4459	0.4562	0.4668	0.4777	0.4886	0.4993	0.5101	0.5208	0.5313	0.5409	0.5480	0.5540	0.5594	0.5644	0.5692	0.5737	0.5786	0.5839	0.5900	0.5965	0.6033	0.6099	0.6167	0.6237	0.6309	0.6381	0.6452	0.6523
Laos	0.2058	0.2109	0.2163	0.2217	0.2270	0.2328	0.2385	0.2435	0.2482	0.2539	0.2602	0.2668	0.2745	0.2835	0.2936	0.3044	0.3151	0.3263	0.3377	0.3490	0.3598	0.3702	0.3801	0.3896	0.3989	0.4081	0.4175	0.4273	0.4370	0.4471	0.4572	0.4674	0.4776	0.4880	0.4980	0.5077
Malaysia	0.4959	0.5045	0.5125	0.5196	0.5269	0.5331	0.5391	0.5451	0.5520	0.5590	0.5660	0.5738	0.5815	0.5895	0.5979	0.6071	0.6172	0.6278	0.6371	0.6468	0.6570	0.6664	0.6757	0.6849	0.6940	0.7026	0.7103	0.7179	0.7255	0.7319	0.7383	0.7445	0.7504	0.7560	0.7615	0.7669
Maldives	0.2582	0.2627	0.2678	0.2724	0.2797	0.2899	0.3003	0.3133	0.3273	0.3421	0.3571	0.3728	0.3885	0.4044	0.4198	0.4346	0.4490	0.4631	0.4768	0.4896	0.5016	0.5134	0.5243	0.5357	0.5457	0.5534	0.5621	0.5709	0.5796	0.5862	0.5929	0.5997	0.6060	0.6116	0.6174	0.6229
Mauritius	0.4827	0.4912	0.4994	0.5070	0.5139	0.5205	0.5275	0.5352	0.5428	0.5505	0.5588	0.5676	0.5765	0.5852	0.5938	0.6025	0.6106	0.6183	0.6260	0.6330	0.6400	0.6467	0.6529	0.6590	0.6652	0.6711	0.6772	0.6839	0.6907	0.6974	0.7038	0.7106	0.7172	0.7234	0.7295	0.7353
Myanmar	0.1835	0.1927	0.2022	0.2116	0.2211	0.2301	0.2376	0.2431	0.2451	0.2471	0.2490	0.2500	0.2531	0.2576	0.2639	0.2717	0.2807	0.2903	0.2999	0.3110	0.3240	0.3376	0.3520	0.3672	0.3830	0.3991	0.4152	0.4309	0.4448	0.4574	0.4690	0.4801	0.4905	0.5006	0.5104	0.5200
Philippines	0.4486	0.4568	0.4647	0.4720	0.4776	0.4817	0.4858	0.4901	0.4952	0.5006	0.5062	0.5114	0.5163	0.5210	0.5261	0.5314	0.5370	0.5421	0.5461	0.5500	0.5543	0.5587	0.5635	0.5686	0.5743	0.5802	0.5864	0.5929	0.5992	0.6052	0.6115	0.6178	0.6244	0.6315	0.6385	0.6454
Sri Lanka	0.4658	0.4711	0.4769	0.4831	0.4902	0.4982	0.5066	0.5146	0.5220	0.5288	0.5355	0.5421	0.5481	0.5545	0.5610	0.5675	0.5752	0.5827	0.5901	0.5966	0.6029	0.6083	0.6133	0.6184	0.6235	0.6290	0.6351	0.6418	0.6489	0.6562	0.6641	0.6723	0.6810	0.6893	0.6975	0.7054
Seychelles	0.5098	0.5209	0.5313	0.5417	0.5518	0.5622	0.5703	0.5781	0.5859	0.5944	0.6037	0.6149	0.6263	0.6375	0.6471	0.6553	0.6625	0.6700	0.6769	0.6829	0.6883	0.6949	0.7005	0.7048	0.7082	0.7120	0.7148	0.7186	0.7218	0.7251	0.7291	0.7343	0.7402	0.7465	0.7526	0.7585
Thailand	0.4398	0.4509	0.4616	0.4717	0.4816	0.4910	0.5000	0.5090	0.5184	0.5281	0.5378	0.5475	0.5571	0.5668	0.5764	0.5861	0.5956	0.6032	0.6086	0.6136	0.6183	0.6235	0.6289	0.6347	0.6407	0.6468	0.6530	0.6594	0.6652	0.6704	0.6760	0.6817	0.6878	0.6938	0.6994	0.7050
Timor-Leste	0.2015	0.2077	0.2135	0.2196	0.2264	0.2337	0.2398	0.2463	0.2529	0.2594	0.2652	0.2709	0.2764	0.2811	0.2852	0.2888	0.2851	0.2780	0.2674	0.2519	0.2402	0.2521	0.2656	0.2783	0.3014	0.3270	0.3551	0.3739	0.3893	0.4005	0.4099	0.4209	0.4307	0.4380	0.4436	0.4499
Vietnam	0.2937	0.3056	0.3153	0.3261	0.3368	0.3466	0.3558	0.3642	0.3725	0.3810	0.3898	0.3992	0.4097	0.4209	0.4328	0.4455	0.4586	0.4716	0.4836	0.4946	0.5047	0.5142	0.5232	0.5317	0.5402	0.5490	0.5575	0.5660	0.5744	0.5824	0.5905	0.5986	0.6064	0.6139	0.6212	0.6283
Oceania	0.3305	0.3348	0.3389	0.3440	0.3490	0.3538	0.3589	0.3637	0.3688	0.3733	0.3771	0.3816	0.3865	0.3928	0.3991	0.4044	0.4097	0.4138	0.4176	0.4213	0.4244	0.4270	0.4290	0.4312	0.4335	0.4362	0.4394	0.4434	0.4481	0.4533	0.4591	0.4657	0.4726	0.4795	0.4867	0.4944
American Samoa	0.6156	0.6188	0.6306	0.6395	0.6266	0.6177	0.6314	0.6285	0.6431	0.6426	0.6427	0.6528	0.6432	0.6435	0.6605	0.6753	0.6894	0.7027	0.7026	0.7031	0.7089	0.7214	0.7116	0.7122	0.7128	0.7128	0.7124	0.7115	0.7117	0.7131	0.7131	0.7130	0.7135	0.7137	0.7137	0.7136
Federated States of Micronesia	0.4218	0.4280	0.4338	0.4401	0.4474	0.4555	0.4623	0.4692	0.4760	0.4826	0.4889	0.4956	0.5017	0.5079	0.5131	0.5189	0.5254	0.5304	0.5356	0.5412	0.5480	0.5534	0.5597	0.5664	0.5725	0.5789	0.5852	0.5906	0.5952	0.5994	0.6039	0.6093	0.6143	0.6180	0.6212	0.6242
Fiji	0.5149	0.5218	0.5286	0.5344	0.5405	0.5456	0.5497	0.5525	0.5559	0.5603	0.5657	0.5706	0.5763	0.5815	0.5872	0.5932	0.5989	0.6043	0.6096	0.6153	0.6207	0.6265	0.6318	0.6367	0.6417	0.6464	0.6520	0.6573	0.6625	0.6672	0.6720	0.6756	0.6792	0.6835	0.6883	0.6934
Guam	0.7046	0.7126	0.7197	0.7259	0.7315	0.7368	0.7421	0.7477	0.7539	0.7607	0.7681	0.7749	0.7815	0.7876	0.7933	0.7985	0.8030	0.8076	0.8124	0.8175	0.8228	0.8276	0.8323	0.8367	0.8415	0.8464	0.8510	0.8552	0.8593	0.8634	0.8672	0.8709	0.8746	0.8780	0.8812	0.8841
Kiribati	0.4170	0.4140	0.4110	0.4071	0.4031	0.3991	0.3978	0.3962	0.3970	0.3980	0.4002	0.4010	0.4018	0.4032	0.4055	0.4080	0.4117	0.4161	0.4218	0.4268	0.4327	0.4374	0.4418	0.4466	0.4504	0.4539	0.4573	0.4601	0.4623	0.4642	0.4660	0.4668	0.4687	0.4713	0.4744	0.4778
Marshall Islands	0.3554	0.3625	0.3693	0.3770	0.3841	0.3896	0.3974	0.4059	0.4057	0.4211	0.4312	0.4697	0.4836	0.4860	0.4808	0.4800	0.4828	0.4768	0.4768	0.4830	0.4958	0.5011	0.5057	0.5107	0.5159	0.5214	0.5273	0.5344	0.5416	0.5480	0.5548	0.5616	0.5695	0.5774	0.5847	0.5916
Northern Mariana Islands	0.6463	0.6647	0.6810	0.6957	0.7090	0.7217	0.7332	0.7446	0.7552	0.7652	0.7748	0.7792	0.7835	0.7879	0.7926	0.7974	0.8056	0.8133	0.8202	0.8269	0.8334	0.8385	0.8434	0.8474	0.8506	0.8519	0.8523	0.8521	0.8516	0.8495	0.8473	0.8442	0.8417	0.8401	0.8397	0.8406
Papua New Guinea	0.2716	0.2752	0.2782	0.2829	0.2875	0.2925	0.2976	0.3032	0.3090	0.3138	0.3173	0.3214	0.3264	0.3333	0.3402	0.3455	0.3511	0.3552	0.3593	0.3632	0.3664	0.3689	0.3712	0.3738	0.3764	0.3797	0.3832	0.3877	0.3930	0.3991	0.4059	0.4138	0.4221	0.4303	0.4388	0.4481
Samoa	0.4295	0.4383	0.4462	0.4532	0.4596	0.4659	0.4742	0.4817	0.4883	0.4946	0.4998	0.5065	0.5121	0.5193	0.5243	0.5288	0.5354	0.5414	0.5457	0.5496	0.5541	0.5613	0.5680	0.5742	0.5796	0.5848	0.5905	0.5964	0.6025	0.6078	0.6131	0.6182	0.6234	0.6280	0.6324	0.6367
Solomon Islands	0.2539	0.2615	0.2679	0.2735	0.2786	0.2832	0.2874	0.2920	0.2970	0.3029	0.3098	0.3173	0.3269	0.3368	0.3475	0.3587	0.3684	0.3767	0.3839	0.3900	0.3929	0.3938	0.3933	0.3931	0.3935	0.3954	0.3990	0.4042	0.4109	0.4165	0.4235	0.4318	0.4401	0.4478	0.4548	0.4614
Tonga	0.3910	0.3983	0.4080	0.4183	0.4332	0.4481	0.4635	0.4777	0.4890	0.4981	0.5056	0.5127	0.5176	0.5224	0.5269	0.5315	0.5367	0.5413	0.5455	0.5492	0.5522	0.5592	0.5655	0.5709	0.5757	0.5801	0.5847	0.5890	0.5936	0.5985	0.6038	0.6078	0.6116	0.6149	0.6185	0.6224
Vanuatu	0.3452	0.3506	0.3559	0.3615	0.3676	0.3731	0.3786	0.3834	0.3872	0.3909	0.3957	0.4012	0.4065	0.4117	0.4175	0.4230	0.4281	0.4336	0.4394	0.4452	0.4518	0.4579	0.4632	0.4688	0.4743	0.4799	0.4865	0.4929	0.4997	0.5062	0.5123	0.5176	0.5228	0.5277	0.5323	0.5360
Central Europe, Eastern Europe, and Central Asia	0.6545	0.6590	0.6633	0.6675	0.6716	0.6758	0.6807	0.6860	0.6919	0.6981	0.7040	0.7095	0.7137	0.7165	0.7186	0.7204	0.7218	0.7231	0.7245	0.7262	0.7292	0.7329	0.7374	0.7430	0.7493	0.7559	0.7630	0.7707	0.7784	0.7839	0.7893	0.7950	0.8005	0.8058	0.8109	0.8158
Central Asia	0.5516	0.5571	0.5622	0.5670	0.5716	0.5762	0.5813	0.5866	0.5925	0.5985	0.6043	0.6094	0.6122	0.6140	0.6145	0.6144	0.6145	0.6147	0.6149	0.6159	0.6181	0.6218	0.6269	0.6331	0.6401	0.6477	0.6560	0.6647	0.6733	0.6807	0.6883	0.6959	0.7034	0.7106	0.7176	0.7240
Armenia	0.5513	0.5561	0.5604	0.5643	0.5678	0.5712	0.5758	0.5806	0.5857	0.5910	0.5955	0.5991	0.5969	0.5933	0.5909	0.5898	0.5911	0.5929	0.5961	0.6000	0.6052	0.6126	0.6227	0.6344	0.6464	0.6595	0.6731	0.6873	0.7002	0.7084	0.7163	0.7246	0.7333	0.7415	0.7488	0.7552
Azerbaijan	0.5867	0.5946	0.6021	0.6092	0.6155	0.6208	0.6264	0.6314	0.6361	0.6408	0.6446	0.6505	0.6533	0.6541	0.6530	0.6502	0.6473	0.6443	0.6415	0.6398	0.6403	0.6432	0.6485	0.6556	0.6637	0.6757	0.6918	0.7092	0.7250	0.7377	0.7493	0.7589	0.7673	0.7751	0.7820	0.7883
Georgia	0.6430	0.6483	0.6533	0.6582	0.6627	0.6671	0.6724	0.6778	0.6834	0.6890	0.6938	0.6959	0.6920	0.6850	0.6766	0.6679	0.6599	0.6534	0.6481	0.6451	0.6451	0.6489	0.6558	0.6646	0.6733	0.6824	0.6918	0.7022	0.7117	0.7193	0.7267	0.7344	0.7419	0.7488	0.7552	0.7611
Kazakhstan	0.6312	0.6361	0.6405	0.6446	0.6486	0.6527	0.6576	0.6630	0.6691	0.6755	0.6818	0.6875	0.6936	0.6980	0.7012	0.7035	0.7062	0.7087	0.7106	0.7125	0.7156	0.7202	0.7253	0.7310	0.7369	0.7431	0.7494	0.7559	0.7623	0.7676	0.7738	0.7805	0.7873	0.7941	0.8006	0.8067
Kyrgyzstan	0.5515	0.5549	0.5581	0.5615	0.5651	0.5691	0.5733	0.5778	0.5827	0.5876	0.5925	0.5972	0.5997	0.6003	0.5987	0.5961	0.5938	0.5918	0.5889	0.5865	0.5853	0.5854	0.5861	0.5888	0.5927	0.5955	0.5974	0.6004	0.6045	0.6081	0.6108	0.6143	0.6174	0.6217	0.6261	0.6307
Mongolia	0.3804	0.3917	0.4030	0.4145	0.4265	0.4390	0.4524	0.4668	0.4824	0.4977	0.5133	0.5257	0.5361	0.5442	0.5518	0.5598	0.5672	0.5744	0.5810	0.5878	0.5944	0.6011	0.6077	0.6144	0.6216	0.6289	0.6365	0.6441	0.6511	0.6563	0.6620	0.6693	0.6777	0.6868	0.6960	0.7047
Tajikistan	0.4511	0.4567	0.4614	0.4655	0.4696	0.4739	0.4792	0.4853	0.4918	0.4987	0.5054	0.5106	0.5111	0.5104	0.5071	0.5022	0.4952	0.4878	0.4799	0.4718	0.4662	0.4647	0.4689	0.4768	0.4872	0.4973	0.5074	0.5170	0.5255	0.5331	0.5407	0.5473	0.5538	0.5606	0.5674	0.5741
Turkmenistan	0.5304	0.5378	0.5446	0.5511	0.5573	0.5632	0.5688	0.5744	0.5807	0.5875	0.5947	0.6019	0.6082	0.6161	0.6213	0.6255	0.6292	0.6311	0.6331	0.6364	0.6421	0.6481	0.6547	0.6618	0.6701	0.6808	0.6919	0.7031	0.7139	0.7240	0.7340	0.7446	0.7547	0.7641	0.7727	0.7805
Uzbekistan	0.4818	0.4885	0.4947	0.5007	0.5064	0.5118	0.5178	0.5238	0.5306	0.5376	0.5446	0.5508	0.5551	0.5599	0.5634	0.5669	0.5708	0.5752	0.5797	0.5849	0.5902	0.5956	0.6012	0.6071	0.6134	0.6201	0.6273	0.6346	0.6426	0.6506	0.6589	0.6674	0.6758	0.6840	0.6918	0.6992
Central Europe	0.6428	0.6483	0.6534	0.6584	0.6634	0.6682	0.6733	0.6785	0.6840	0.6896	0.6946	0.6986	0.7021	0.7057	0.7099	0.7148	0.7209	0.7272	0.7337	0.7400	0.7464	0.7529	0.7592	0.7652	0.7710	0.7767	0.7828	0.7892	0.7958	0.8013	0.8067	0.8123	0.8175	0.8226	0.8276	0.8327
Albania	0.5091	0.5167	0.5238	0.5303	0.5363	0.5419	0.5481	0.5541	0.5598	0.5663	0.5719	0.5713	0.5693	0.5688	0.5707	0.5750	0.5830	0.5893	0.5969	0.6068	0.6176	0.6288	0.6395	0.6500	0.6604	0.6703	0.6798	0.6885	0.6965	0.7030	0.7083	0.7146	0.7205	0.7259	0.7312	0.7364
Bosnia and Herzegovina	0.4259	0.4345	0.4424	0.4494	0.4562	0.4629	0.4697	0.4760	0.4814	0.4866	0.4891	0.4903	0.4909	0.4918	0.4953	0.5041	0.5280	0.5555	0.5817	0.6043	0.6226	0.6382	0.6516	0.6628	0.6724	0.6805	0.6881	0.6954	0.7026	0.7086	0.7144	0.7199	0.7248	0.7297	0.7343	0.7387
Bulgaria	0.6353	0.6425	0.6499	0.6570	0.6639	0.6699	0.6759	0.6826	0.6900	0.6968	0.7019	0.7067	0.7101	0.7129	0.7158	0.7186	0.7209	0.7229	0.7254	0.7275	0.7304	0.7340	0.7384	0.7430	0.7479	0.7530	0.7589	0.7657	0.7727	0.7785	0.7840	0.7894	0.7944	0.7992	0.8037	0.8079
Croatia	0.6084	0.6157	0.6225	0.6287	0.6349	0.6410	0.6473	0.6535	0.6596	0.6654	0.6702	0.6725	0.6735	0.6734	0.6736	0.6745	0.6775	0.6817	0.6870	0.6926	0.6995	0.7066	0.7140	0.7214	0.7283	0.7347	0.7411	0.7477	0.7541	0.7591	0.7637	0.7684	0.7726	0.7766	0.7805	0.7842
Czech Republic	0.7264	0.7341	0.7412	0.7481	0.7547	0.7606	0.7656	0.7701	0.7749	0.7801	0.7857	0.7901	0.7949	0.8000	0.8057	0.8121	0.8187	0.8245	0.8297	0.8343	0.8387	0.8429	0.8465	0.8497	0.8529	0.8563	0.8602	0.8648	0.8690	0.8724	0.8758	0.8792	0.8823	0.8854	0.8885	0.8918
Hungary	0.6771	0.6845	0.6915	0.6979	0.7033	0.7076	0.7113	0.7153	0.7193	0.7234	0.7274	0.7304	0.7335	0.7369	0.7411	0.7458	0.7509	0.7562	0.7616	0.7670	0.7729	0.7792	0.7855	0.7914	0.7973	0.8031	0.8091	0.8146	0.8198	0.8238	0.8278	0.8319	0.8357	0.8399	0.8443	0.8491
Macedonia	0.5956	0.6023	0.6085	0.6139	0.6193	0.6244	0.6299	0.6349	0.6391	0.6435	0.6465	0.6495	0.6519	0.6537	0.6558	0.6582	0.6610	0.6645	0.6686	0.6735	0.6792	0.6843	0.6896	0.6950	0.7009	0.7073	0.7141	0.7212	0.7280	0.7338	0.7393	0.7446	0.7491	0.7535	0.7578	0.7622
Montenegro	0.6382	0.6443	0.6500	0.6555	0.6609	0.6660	0.6704	0.6747	0.6788	0.6829	0.6870	0.6907	0.6921	0.6903	0.6885	0.6877	0.6896	0.6927	0.6967	0.7000	0.7056	0.7119	0.7178	0.7239	0.7300	0.7361	0.7431	0.7513	0.7597	0.7664	0.7727	0.7790	0.7844	0.7896	0.7944	0.7989
Poland	0.6632	0.6656	0.6676	0.6705	0.6740	0.6782	0.6831	0.6884	0.6941	0.7005	0.7057	0.7103	0.7153	0.7211	0.7277	0.7353	0.7437	0.7527	0.7618	0.7708	0.7795	0.7871	0.7936	0.7994	0.8047	0.8096	0.8144	0.8198	0.8253	0.8308	0.8368	0.8431	0.8492	0.8553	0.8614	0.8675

Appendix Table 5. Socio-Demographic Index values for all estimated GBD 2015 locations, 1980–2015

Location	1980	1981	1982	1983	1984	1985	1986	1987	1988	1989	1990	1991	1992	1993	1994	1995	1996	1997	1998	1999	2000	2001	2002	2003	2004	2005	2006	2007	2008	2009	2010	2011	2012	2013	2014	2015
Romania	0.6376	0.6439	0.6497	0.6552	0.6604	0.6654	0.6710	0.6767	0.6825	0.6879	0.6931	0.6964	0.6984	0.7001	0.7018	0.7037	0.7065	0.7086	0.7104	0.7128	0.7158	0.7199	0.7247	0.7298	0.7349	0.7407	0.7476	0.7550	0.7631	0.7691	0.7744	0.7800	0.7851	0.7901	0.7947	0.7991
Serbia	0.6039	0.6091	0.6140	0.6187	0.6233	0.6279	0.6326	0.6375	0.6426	0.6478	0.6531	0.6579	0.6601	0.6596	0.6589	0.6585	0.6602	0.6632	0.6670	0.6697	0.6741	0.6807	0.6885	0.6966	0.7051	0.7136	0.7218	0.7300	0.7383	0.7448	0.7503	0.7558	0.7605	0.7649	0.7686	0.7718
Slovakia	0.6493	0.6564	0.6632	0.6697	0.6762	0.6823	0.6881	0.6938	0.6997	0.7060	0.7126	0.7173	0.7218	0.7267	0.7323	0.7387	0.7454	0.7526	0.7598	0.7665	0.7729	0.7793	0.7855	0.7915	0.7974	0.8032	0.8096	0.8168	0.8241	0.8297	0.8355	0.8411	0.8465	0.8516	0.8566	0.8615
Slovenia	0.6788	0.6874	0.6957	0.7037	0.7115	0.7191	0.7266	0.7338	0.7406	0.7472	0.7529	0.7570	0.7604	0.7641	0.7682	0.7725	0.7774	0.7827	0.7882	0.7941	0.8004	0.8064	0.8123	0.8176	0.8227	0.8273	0.8320	0.8367	0.8412	0.8440	0.8465	0.8487	0.8505	0.8521	0.8540	0.8561
Eastern Europe	0.6892	0.6933	0.6972	0.7012	0.7051	0.7092	0.7143	0.7201	0.7264	0.7334	0.7403	0.7469	0.7521	0.7552	0.7570	0.7580	0.7575	0.7568	0.7560	0.7559	0.7575	0.7601	0.7639	0.7696	0.7764	0.7836	0.7916	0.8001	0.8086	0.8140	0.8192	0.8246	0.8297	0.8346	0.8391	0.8431
Belarus	0.6315	0.6366	0.6415	0.6462	0.6509	0.6559	0.6616	0.6674	0.6738	0.6806	0.6868	0.6932	0.6985	0.7026	0.7043	0.7051	0.7065	0.7088	0.7122	0.7163	0.7214	0.7274	0.7341	0.7418	0.7506	0.7600	0.7700	0.7802	0.7910	0.8002	0.8094	0.8185	0.8267	0.8340	0.8408	0.8468
Estonia	0.6590	0.6635	0.6677	0.6717	0.6757	0.6799	0.6849	0.6906	0.6973	0.7048	0.7123	0.7195	0.7240	0.7274	0.7307	0.7348	0.7388	0.7444	0.7506	0.7565	0.7633	0.7702	0.7771	0.7840	0.7907	0.7978	0.8056	0.8140	0.8214	0.8266	0.8320	0.8381	0.8444	0.8504	0.8558	0.8608
Latvia	0.6867	0.6904	0.6938	0.6972	0.7004	0.7040	0.7086	0.7142	0.7206	0.7277	0.7348	0.7401	0.7414	0.7422	0.7434	0.7447	0.7459	0.7481	0.7515	0.7555	0.7603	0.7662	0.7731	0.7803	0.7878	0.7959	0.8050	0.8149	0.8235	0.8292	0.8343	0.8400	0.8458	0.8514	0.8566	0.8614
Lithuania	0.6683	0.6734	0.6783	0.6832	0.6878	0.6922	0.6970	0.7019	0.7071	0.7125	0.7175	0.7220	0.7243	0.7251	0.7253	0.7260	0.7274	0.7301	0.7344	0.7392	0.7449	0.7518	0.7597	0.7682	0.7764	0.7843	0.7922	0.8008	0.8088	0.8137	0.8173	0.8215	0.8256	0.8296	0.8334	0.8369
Moldova	0.5747	0.5781	0.5813	0.5844	0.5875	0.5907	0.5949	0.5995	0.6046	0.6101	0.6156	0.6203	0.6210	0.6214	0.6190	0.6155	0.6112	0.6071	0.6030	0.5990	0.5970	0.5983	0.6032	0.6100	0.6185	0.6278	0.6367	0.6450	0.6539	0.6605	0.6678	0.6759	0.6829	0.6905	0.6974	0.7033
Russia	0.7150	0.7185	0.7217	0.7250	0.7281	0.7315	0.7360	0.7414	0.7475	0.7544	0.7613	0.7683	0.7736	0.7769	0.7792	0.7805	0.7799	0.7792	0.7780	0.7777	0.7793	0.7816	0.7849	0.7899	0.7957	0.8020	0.8091	0.8168	0.8246	0.8296	0.8343	0.8391	0.8438	0.8482	0.8524	0.8564
Ukraine	0.6403	0.6461	0.6518	0.6575	0.6632	0.6691	0.6757	0.6823	0.6894	0.6968	0.7038	0.7099	0.7150	0.7185	0.7195	0.7192	0.7181	0.7166	0.7152	0.7142	0.7143	0.7166	0.7205	0.7270	0.7361	0.7453	0.7551	0.7656	0.7756	0.7814	0.7871	0.7933	0.7987	0.8041	0.8080	0.8111
High-income	0.7386	0.7442	0.7490	0.7537	0.7585	0.7634	0.7684	0.7733	0.7784	0.7835	0.7887	0.7938	0.7988	0.8034	0.8082	0.8128	0.8177	0.8226	0.8272	0.8317	0.8361	0.8402	0.8440	0.8474	0.8509	0.8543	0.8579	0.8618	0.8655	0.8686	0.8720	0.8755	0.8789	0.8822	0.8853	0.8884
High-income Asia Pacific	0.7119	0.7188	0.7251	0.7312	0.7376	0.7442	0.7510	0.7579	0.7651	0.7727	0.7802	0.7877	0.7946	0.8012	0.8075	0.8138	0.8202	0.8264	0.8317	0.8366	0.8413	0.8460	0.8503	0.8543	0.8581	0.8613	0.8645	0.8680	0.8709	0.8732	0.8759	0.8784	0.8810	0.8836	0.8862	0.8887
Brunei	0.7178	0.7254	0.7320	0.7384	0.7433	0.7469	0.7542	0.7613	0.7687	0.7762	0.7839	0.7900	0.7973	0.8050	0.8137	0.8233	0.8325	0.8424	0.8527	0.8625	0.8714	0.8767	0.8815	0.8858	0.8898	0.8934	0.8963	0.8991	0.9021	0.9053	0.9089	0.9121	0.9150	0.9179	0.9206	0.9232
Japan	0.7495	0.7553	0.7603	0.7648	0.7695	0.7746	0.7798	0.7853	0.7916	0.7984	0.8054	0.8124	0.8190	0.8250	0.8305	0.8357	0.8411	0.8462	0.8505	0.8544	0.8580	0.8615	0.8648	0.8678	0.8706	0.8730	0.8755	0.8784	0.8807	0.8824	0.8845	0.8865	0.8886	0.8910	0.8932	0.8955
Aichi	0.7613	0.7674	0.7736	0.7764	0.7808	0.7857	0.7918	0.7960	0.8034	0.8107	0.8177	0.8252	0.8312	0.8374	0.8431	0.8482	0.8534	0.8583	0.8617	0.8656	0.8692	0.8731	0.8766	0.8794	0.8815	0.8841	0.8859	0.8885	0.8901	0.8917	0.8938	0.8954	0.8978	0.9007	0.9030	0.9053
Akita	0.7333	0.7401	0.7444	0.7493	0.7529	0.7604	0.7637	0.7682	0.7747	0.7791	0.7869	0.7936	0.7982	0.8040	0.8082	0.8138	0.8207	0.8248	0.8305	0.8337	0.8380	0.8410	0.8455	0.8491	0.8515	0.8529	0.8560	0.8598	0.8622	0.8651	0.8691	0.8698	0.8715	0.8756	0.8781	0.8807
Aomori	0.7357	0.7421	0.7474	0.7512	0.7562	0.7618	0.7659	0.7719	0.7788	0.7843	0.7923	0.7967	0.8038	0.8085	0.8138	0.8188	0.8245	0.8296	0.8339	0.8377	0.8421	0.8444	0.8482	0.8523	0.8548	0.8589	0.8618	0.8657	0.8673	0.8706	0.8708	0.8720	0.8745	0.8758	0.8778	0.8799
Chiba	0.7415	0.7448	0.7507	0.7560	0.7607	0.7664	0.7733	0.7787	0.7853	0.7923	0.7997	0.8066	0.8142	0.8197	0.8248	0.8305	0.8356	0.8408	0.8452	0.8487	0.8518	0.8550	0.8574	0.8605	0.8625	0.8649	0.8675	0.8700	0.8718	0.8727	0.8748	0.8769	0.8797	0.8821	0.8842	0.8865
Ehime	0.7371	0.7422	0.7478	0.7526	0.7573	0.7627	0.7665	0.7729	0.7786	0.7845	0.7920	0.7991	0.8047	0.8109	0.8172	0.8210	0.8275	0.8319	0.8369	0.8416	0.8449	0.8482	0.8525	0.8538	0.8578	0.8598	0.8623	0.8646	0.8675	0.8680	0.8701	0.8712	0.8732	0.8770	0.8794	0.8819
Fukui	0.7448	0.7532	0.7567	0.7611	0.7643	0.7699	0.7752	0.7788	0.7864	0.7911	0.7993	0.8071	0.8142	0.8191	0.8240	0.8294	0.8353	0.8411	0.8450	0.8493	0.8535	0.8567	0.8607	0.8645	0.8680	0.8688	0.8709	0.8739	0.8763	0.8772	0.8805	0.8823	0.8836	0.8874	0.8897	0.8920
Fukuoka	0.7455	0.7510	0.7557	0.7605	0.7650	0.7704	0.7752	0.7824	0.7888	0.7944	0.8019	0.8081	0.8155	0.8215	0.8268	0.8321	0.8374	0.8425	0.8472	0.8514	0.8552	0.8585	0.8618	0.8652	0.8675	0.8699	0.8718	0.8736	0.8758	0.8772	0.8787	0.8805	0.8826	0.8845	0.8863	0.8882
Fukushima	0.7312	0.7378	0.7419	0.7463	0.7506	0.7567	0.7604	0.7670	0.7720	0.7796	0.7861	0.7920	0.7987	0.8052	0.8112	0.8158	0.8216	0.8279	0.8322	0.8352	0.8395	0.8429	0.8469	0.8501	0.8537	0.8557	0.8592	0.8628	0.8655	0.8677	0.8702	0.8745	0.8804	0.8790	0.8817	0.8843
Gifu	0.7408	0.7469	0.7527	0.7560	0.7608	0.7652	0.7725	0.7763	0.7829	0.7900	0.7970	0.8048	0.8101	0.8162	0.8225	0.8268	0.8321	0.8381	0.8415	0.8449	0.8486	0.8528	0.8552	0.8578	0.8619	0.8637	0.8660	0.8692	0.8717	0.8725	0.8754	0.8762	0.8784	0.8817	0.8840	0.8864
Gunma	0.7454	0.7512	0.7560	0.7604	0.7655	0.7699	0.7766	0.7806	0.7870	0.7932	0.8011	0.8072	0.8139	0.8204	0.8253	0.8309	0.8363	0.8415	0.8465	0.8501	0.8533	0.8570	0.8602	0.8631	0.8668	0.8685	0.8721	0.8753	0.8767	0.8793	0.8817	0.8838	0.8873	0.8896	0.8921	0.8946
Hiroshima	0.7456	0.7525	0.7563	0.7613	0.7661	0.7710	0.7753	0.7819	0.7887	0.7949	0.8012	0.8094	0.8156	0.8217	0.8280	0.8335	0.8388	0.8437	0.8486	0.8529	0.8565	0.8592	0.8630	0.8649	0.8679	0.8698	0.8722	0.8734	0.8761	0.8762	0.8780	0.8788	0.8813	0.8832	0.8849	0.8867
HokkaidÅ	0.7460	0.7521	0.7561	0.7606	0.7656	0.7714	0.7759	0.7817	0.7877	0.7946	0.8005	0.8074	0.8136	0.8203	0.8257	0.8308	0.8364	0.8412	0.8453	0.8497	0.8532	0.8562	0.8588	0.8614	0.8644	0.8672	0.8697	0.8728	0.8753	0.8767	0.8784	0.8801	0.8819	0.8839	0.8859	0.8879
HyÅ go	0.7410	0.7471	0.7518	0.7563	0.7613	0.7665	0.7716	0.7780	0.7844	0.7914	0.7975	0.8051	0.8114	0.8177	0.8225	0.8285	0.8338	0.8384	0.8425	0.8461	0.8499	0.8540	0.8567	0.8598	0.8626	0.8652	0.8672	0.8699	0.8717	0.8734	0.8749	0.8763	0.8786	0.8812	0.8834	0.8857
Ibaraki	0.7456	0.7511	0.7559	0.7611	0.7657	0.7721	0.7770	0.7825	0.7899	0.7962	0.8032	0.8101	0.8164	0.8232	0.8292	0.8348	0.8400	0.8453	0.8496	0.8530	0.8571	0.8603	0.8636	0.8665	0.8692	0.8728	0.8746	0.8777	0.8804	0.8817	0.8840	0.8864	0.8883	0.8912	0.8934	0.8958
Ishikawa	0.7422	0.7495	0.7535	0.7587	0.7639	0.7699	0.7741	0.7812	0.7861	0.7931	0.7998	0.8082	0.8139	0.8212	0.8248	0.8318	0.8364	0.8417	0.8452	0.8495	0.8529	0.8560	0.8604	0.8617	0.8656	0.8679	0.8707	0.8727	0.8752	0.8774	0.8805	0.8816	0.8828	0.8851	0.8873	0.8896
Iwate	0.7280	0.7351	0.7396	0.7436	0.7494	0.7550	0.7595	0.7661	0.7707	0.7768	0.7829	0.7889	0.7955	0.8015	0.8079	0.8127	0.8194	0.8252	0.8282	0.8321	0.8359	0.8389	0.8429	0.8464	0.8491	0.8519	0.8553	0.8587	0.8613	0.8634	0.8657	0.8688	0.8697	0.8714	0.8735	0.8758
Kagawa	0.7423	0.7493	0.7534	0.7578	0.7622	0.7675	0.7717	0.7777	0.7840	0.7912	0.7983	0.8064	0.8122	0.8193	0.8240	0.8284	0.8347	0.8383	0.8424	0.8454	0.8488	0.8531	0.8557	0.8589	0.8615	0.8635	0.8670	0.8686	0.8712	0.8728	0.8741	0.8753	0.8774	0.8797	0.8819	0.8841
Kagoshima	0.7243	0.7293	0.7337	0.7396	0.7448	0.7508	0.7597	0.7581	0.7666	0.7731	0.7807	0.7883	0.7944	0.8001	0.8059	0.8120	0.8174	0.8230	0.8284	0.8328	0.8348	0.8388	0.8423	0.8448	0.8482	0.8495	0.8527	0.8553	0.8566	0.8592	0.8605	0.8607	0.8635	0.8661	0.8679	0.8697
Kanagawa	0.7491	0.7549	0.7605	0.7650	0.7703	0.7748	0.7809	0.7862	0.7917	0.7997	0.8066	0.8133	0.8205	0.8264	0.8319	0.8373	0.8427	0.8475	0.8516	0.8553	0.8590	0.8623	0.8652	0.8677	0.8705	0.8729	0.8750	0.8777	0.8801	0.8814	0.8836	0.8861	0.8882	0.8911	0.8936	0.8961
KÅ chi	0.7268	0.7264	0.7327	0.7367	0.7417	0.7467	0.7515	0.7582	0.7642	0.7706	0.7790	0.7840	0.7910	0.7939	0.8016	0.8067	0.8111	0.8183	0.8233	0.8269	0.8298	0.8329	0.8372	0.8410	0.8445	0.8460	0.8485	0.8531	0.8542	0.8577	0.8581	0.8615	0.8623	0.8639	0.8660	0.8684
Kumamoto	0.7277	0.7324	0.7384	0.7428	0.7464	0.7524	0.7568	0.7625	0.7690	0.7751	0.7825	0.7887	0.7953	0.8007	0.8071	0.8112	0.8168	0.8226	0.8272	0.8306	0.8340	0.8377	0.8408	0.8440	0.8463	0.8485	0.8507	0.8531	0.8551	0.8566	0.8587	0.8599	0.8616	0.8634	0.8651	0.8668
KyÅ to	0.7522	0.7590	0.7633	0.7671	0.7713	0.7773	0.7810	0.7885	0.7942	0.8008	0.8080	0.8168	0.8224	0.8293	0.8347	0.8401	0.8448	0.8503	0.8539	0.8579	0.8618	0.8658	0.8695	0.8722	0.8751	0.8768	0.8792	0.8828	0.8844	0.8866	0.8881	0.8904	0.8932	0.8953	0.8976	0.9002
Mie	0.7469	0.7542	0.7585	0.7616	0.7666	0.7729	0.7770	0.7828	0.7890	0.7956	0.8029	0.8115	0.8172	0.8236	0.8293	0.8345	0.8399	0.8447	0.8490	0.8530	0.8560	0.8605	0.8628	0.8664	0.8693	0.8722	0.8743	0.8770	0.8793	0.8800	0.8827	0.8843	0.8867	0.8893	0.8915	0.8940
Miyagi	0.7379	0.7441	0.7484	0.7537	0.7594	0.7652	0.7701	0.7757	0.7831	0.7888	0.7964	0.8036	0.8101	0.8179	0.8233	0.8275	0.8340	0.8390	0.8432	0.8470	0.8507	0.8544	0.8576	0.8609	0.8645	0.8670	0.8700	0.8725	0.8748	0.8778	0.8797	0.8835	0.8833	0.8846	0.8866	0.8887
Miyazaki	0.7269	0.7317	0.7358	0.7421	0.7466	0.7531	0.7566	0.7628	0.7690	0.7742	0.7832	0.7891	0.7944	0.8002	0.8048	0.8094	0.8149	0.8203	0.8255	0.8298	0.8330	0.8357	0.8399	0.8438	0.8455	0.8481	0.8499	0.8514	0.8539	0.8551	0.8567	0.8575	0.8601	0.8614	0.8627	0.8641
Nagano	0.7380	0.7467	0.7518	0.7558	0.7604	0.7651	0.7684	0.7751	0.7807	0.7861	0.7934	0.8008	0.8061	0.8140	0.8185	0.8238	0.8300	0.8351	0.8395	0.8443	0.8476	0.8510	0.8549	0.8579	0.8612	0.8632	0.8659	0.8686	0.8720	0.8748	0.8764	0.8782	0.8799	0.8827	0.8850	0.8875
Nagasaki	0.7282	0.7327	0.7381	0.7424	0.7474	0.7533	0.7566	0.7626	0.7688	0.7747	0.7823	0.7876	0.7947	0.7997	0.8060	0.8122	0.8163	0.8229	0.8262	0.8304	0.8340	0.8376	0.8419	0.8444	0.8463	0.8492	0.8515	0.8547	0.8567	0.8583	0.8589	0.8605	0.8609	0.8628	0.8642	0.8657
Nara	0.7242	0.7298	0.7334	0.7378	0.7431	0.7501	0.7541	0.7619	0.7665	0.7737	0.7808	0.7890	0.7948	0.8001	0.8077	0.8122	0.8170	0.8221	0.8256	0.8303	0.8338	0.8378	0.8408	0.8431	0.8463	0.8484	0.8505	0.8534	0.8564	0.8575	0.8592	0.8610	0.8612	0.8643	0.8663	0.8685
Niigata	0.7390	0.7450	0.7505	0.7547	0.7586	0.7640	0.7682	0.7744	0.7802	0.7852	0.7934	0.7999	0.8070	0.8118	0.8178	0.8238	0.8295	0.8347	0.8390	0.8437	0.8475	0.8515	0.8567	0.8596	0.8622	0.8648	0.8674	0.8707	0.8740	0.8758	0.8774	0.8794	0.8812	0.8841	0.8864	0.8888
Å"ita	0.7377	0.7443	0.7498	0.7538	0.7596	0.7642	0.7684	0.7741	0.7803	0.7862	0.7941	0.7995	0.8062	0.8117	0.8167	0.8220	0.8270	0.8323	0.8368	0.8413	0.8443	0.8472	0.8521	0.8549	0.8582	0.8595	0.8615	0.8642	0.8659	0.8685	0.8700	0.8713	0.8746	0.8767	0.8787	0.8808
Okayama	0.7406	0.7473	0.7512	0.7549	0.7596	0.7648	0.7698	0.7756	0.7829	0.7892	0.7959	0.8043	0.8103	0.8171	0.8229	0.8273	0.8323	0.8370	0.8419	0.8459	0.8495	0.8530	0.8565	0.8602	0.8629	0.8660	0.8681	0.8712	0.8738	0.8765	0.8774	0.8788	0.8815	0.8836	0.8859	0.8882
Okinawa	0.7094	0.7174	0.7230	0.7291	0.7338	0.7376	0.7414	0.7489	0.7559	0.7625	0.7728	0.7778	0.7846	0.7908	0.7985	0.8035	0.8086	0.8146	0.8183	0.8217	0.8256	0.8273	0.8328	0.8358	0.8383	0.8393	0.8425	0.8450	0.8472	0.8479	0.8487	0.8504	0.8512	0.8528	0.8544	0.8560
ÅŒsaka	0.7637	0.7704	0.7748	0.7791	0.7838	0.7880	0.7931	0.7991	0.8051	0.8120	0.8190	0.8264	0.8327	0.8395	0.8448	0.8494	0.8536	0.8581	0.8620	0.8656	0.8697	0.8731	0.8763	0.8791	0.8817	0.8838	0.8864	0.8893	0.8911	0.8929	0.8948	0.8964	0.8982	0.9006	0.9028	0.9050
Saga	0.7276	0.7344	0.7380	0.7435	0.7483	0.7524	0.7569	0.7641	0.7703	0.7783	0.7826	0.7899	0.7967	0.8025	0.8081	0.8137	0.8183	0.8226	0.8282	0.8318	0.8343	0.8383	0.8434	0.8471	0.8497	0.8520	0.8550	0.8573	0.8588	0.8618	0.8629	0.8639	0.8664	0.8694	0.8714	0.8735
Saitama	0.7362	0.7413	0.7468	0.7520	0.7571	0.7616	0.7686	0.7739	0.7793	0.7861	0.7934	0.7994	0.8068	0.8120	0.8178	0.8233	0.8287	0.8340	0.8389	0.8427	0.8463	0.8493	0.8520	0.8544	0.8572	0.8589	0.8616	0.8645	0.8668	0.8681	0.8702	0.8722	0.8745	0.8759	0.8780	0.8802
Shiga	0.7484	0.7550	0.7603	0.7633	0.7680	0.7742	0.7790	0.7849	0.7913	0.7988	0.8051	0.8146	0.8209	0.8281	0.8330	0.8392	0.8439	0.8500	0.8543	0.8578	0.8625	0.8660	0.8694	0.8727	0.8754	0.8786	0.8804	0.8835	0.8854	0.8875	0.8886	0.8899	0.8919	0.8951	0.8974	0.8997
Shimane	0.7241	0.7294	0.7361	0.7389	0.7449	0.7493	0.7526	0.7576	0.7644	0.7701	0.7771	0.7844	0.7920	0.7974	0.8037	0.8084	0.8143	0.8214	0.8266	0.8302	0.8333	0.8352	0.8406	0.8443	0.8465	0.8500	0.8509	0.8541	0.8589	0.8600	0.8602	0.8624	0.8633	0.8652	0.8669	0.8686
Shizuoka	0.7539	0.7584	0.7637	0.7679	0.7722	0.7774	0.7835	0.7871	0.7940	0.8016	0.8092	0.8156	0.8236	0.8285	0.8342	0.8403	0.8452	0.8502	0.8543	0.8577	0.8611	0.8645	0.8669	0.8701	0.8728	0.8753	0.8772	0.8788	0.8818	0.8833	0.8850	0.8869	0.8885	0.8911	0.8931	0.8952
Tochigi	0.7480	0.7536	0.7587	0.7632	0.7683	0.7723	0.7784	0.7821	0.7902	0.7973	0.8042	0.8114	0.8188	0.8253	0.8309	0.8369	0.8420	0.8478	0.8522	0.8560	0.8588	0.8618	0.8659	0.8683	0.8715	0.8728	0.8757	0.8793	0.8816	0.8827	0.8864	0.8891	0.8902	0.8933	0.8957	0.8981
Tokushima	0.7421	0.7469	0.7517	0.7559	0.7609	0.7658	0.7710	0.7777	0.7816	0.7891	0.7954	0.8034	0.8109	0.8160	0.8230	0.8252	0.8319	0.8378	0.8420	0.8455	0.8489	0.8529	0.8569	0.8605	0.8631	0.8678	0.8685	0.8727	0.8754	0.8758	0.8773	0.8779	0.8805	0.8828	0.8849	0.8871
TÅ kyÅ	0.8040	0.8096	0.8152	0.8201	0.8245	0.8293	0.8346	0.8400	0.8466	0.8539	0.8606	0.8675	0.8742	0.8802	0.8857	0.8910	0.8965	0.9012	0.9057	0.9094	0.9129	0.9170	0.9196	0.9227	0.9254	0.9279	0.9305	0.9334	0.9359	0.9376	0.9403	0.9432	0.9454	0.9473	0.9496	0.9517
Tottori	0.7244	0.7299	0.7347	0.7365	0.7427	0.7495	0.7529	0.7592	0.7639	0.7713	0.7766	0.7848	0.7921	0.7981	0.8033	0.8084	0.8163	0.8207	0.8255	0.8309	0.8323	0.8358	0.8409	0.8419	0.8464	0.8492	0.8509	0.8555	0.8590	0.8597	0.8622	0.8615	0.8643	0.8662	0.8680	0.8701
Toyama	0.7486	0.7540	0.7590	0.7634	0.7671	0.7723	0.7770	0.7843	0.7888	0.7963	0.8033	0.8108	0.8173	0.8237	0.8287	0.8327	0.8379	0.8432	0.8482	0.8511	0.8550	0.8585	0.8615	0.8655	0.8675	0.8697	0.8733	0.8767	0.8789	0.8810	0.8840	0.8872	0.8881	0.8909	0.8935	0.8963
Wakayama	0.7379	0.7440	0.7479	0.7522	0.7577	0.7631	0.7672	0.7740	0.7808	0.7872	0.7943	0.8011	0.8081	0.8143	0.8197	0.8240	0.8285	0.8342	0.8381	0.8421	0.8458	0.8488	0.8529	0.8559	0.8599	0.8613	0.8640	0.8675	0.8682	0.8706	0.8714	0.8727	0.8736	0.8773	0.8795	0.8819
Yamagata	0.7250	0.7317	0.7373	0.7421	0.7464	0.7517	0.7544	0.7604	0.7644	0.7714	0.7786	0.7849	0.7921	0.7973	0.8026	0.8078	0.8131	0.8193	0.8248	0.8282	0.8315	0.8343	0.8390	0.8427	0.8459	0.8487	0.8517	0.8562	0.8584	0.8615	0.8636	0.8649	0.8681	0.8699	0.8723	0.8747
Yamaguchi	0.7465	0.7519	0.7568	0.7612	0.7655	0.7701	0.7750	0.7812	0.7881	0.7938	0.8016	0.8073	0.8146	0.8193	0.8247	0.8299	0.8355	0.8403	0.8447	0.8479	0.8518	0.8547	0.8586	0.8617	0.8642	0.8661	0.8688	0.8711	0.8740	0.8753	0.8757	0.8777	0.8807	0.8825	0.8844	0.8863
Yamanashi	0.7419	0.7479	0.7527	0.7569	0.7621	0.7652	0.7740	0.7738	0.7831	0.7891	0.7969	0.8017	0.8085	0.8147	0.8200	0.8262	0.8315	0.8368	0.8425	0.8467	0.8503	0.8543	0.8579	0.8605	0.8639	0.8663	0.8699	0.8729	0.8753	0.8777	0.8788	0.8814	0.8829	0.8856	0.8879	0.8905
South Korea	0.6008	0.6119	0.6229	0.6344	0.6462	0.6577	0.6696	0.6815	0.6929	0.7031	0.7127	0.7219	0.7302	0.7383	0.7469	0.7562	0.7656	0.7750	0.7828	0.7910	0.7990	0.8064	0.8137	0.8202	0.8262	0.8317	0.8369	0.8419	0.8464	0.8503	0.8545	0.8583	0.8618	0.8652	0.8684	0.8713
Singapore	0.6401	0.6491	0.6577	0.6689	0.6763	0.6827	0.6943	0.6960	0.6916	0.7058	0.7125	0.7237	0.7316	0.7391	0.7482	0.7575	0.7657	0.7754	0.7866	0.7931	0.7951	0.8071	0.8136	0.8232	0.8298	0.8362	0.8423	0.8464	0.8520	0.8588	0.8660	0.8658	0.8708	0.8743	0.8777	0.8811
Australasia	0.7541	0.7600	0.7653	0.7701	0.7750	0.7799	0.7847	0.7899	0.7952	0.8003	0.8051	0.8095	0.8136	0.8178	0.8225	0.8277	0.8332	0.8387	0.8440	0.8493	0.8542	0.8587	0.8629	0.8665	0.8697	0.8726	0.8754	0.8786	0.8820	0.8856	0.8894	0.8935	0.8978	0.9020	0.9062	0.9103
Australia	0.7558	0.7619	0.7670	0.7717	0.7766	0.7817	0.7867	0.7921	0.7977	0.8032	0.8081	0.8125	0.8167	0.8210	0.8258	0.8309	0.8364	0.8419	0.8474	0.8529	0.8580	0.8626	0.8668	0.8704	0.8736	0.8765	0.8793	0.8825	0.8861	0.8898	0.8938	0.8980	0.9023	0.9066	0.9109	0.9151
New Zealand	0.7458	0.7514	0.7568	0.7621	0.7671	0.7711	0.7749	0.7789	0.7827	0.7861	0.7901	0.7945	0.7981	0.8020	0.8067	0.8120	0.8175	0.8227	0.8271	0.8314	0.8354	0.8392	0.8433	0.8473	0.8507	0.8535	0.8562	0.8590	0.8614	0.8640	0.8670	0.8703	0.8739	0.8774	0.8810	0.8842
Western Europe	0.7000	0.7065	0.7124	0.7179	0.7235	0.7290	0.7347	0.7404	0.7464	0.7526	0.7588	0.7649	0.7707	0.7760	0.7811	0.7861	0.7910	0.7960	0.8010	0.8060	0.8111	0.8159	0.8204	0.8246	0.8285	0.8323	0.8363	0.8404	0.8443	0.8475	0.8509	0.8544	0.8577	0.8609	0.8640	0.8671
Andorra	0.7740	0.7800	0.7857	0.7914	0.7969	0.8015	0.8055	0.8097	0.8140	0.8185	0.8240	0.8274	0.8299	0.8360	0.8413	0.8438	0.8473	0.8497	0.8506	0.8510	0.8620	0.8643	0.8702	0.8776	0.8766	0.8804	0.8835	0.8861	0.8894	0.8930	0.8972	0.9017	0.9062	0.9105	0.9148	0.9186
Austria	0.7409	0.7462	0.7512	0.7561	0.7608	0.7655	0.7701	0.7745	0.7787	0.7831	0.7879	0.7929	0.7977	0.8020	0.8062	0.8102	0.8148	0.8194	0.8241	0.8289	0.8339	0.8385	0.8430	0.8472	0.8514	0.8555	0.8596	0.8638	0.8678	0.8709	0.8742	0.8774	0.8805	0.8833	0.8859	0.8883
Belgium	0.7294	0.7354	0.7409	0.7459	0.7508	0.7556	0.7603	0.7649	0.7698	0.7747	0.7797	0.7847	0.7896	0.7941	0.7986	0.8030	0.8079	0.8130	0.8178	0.8224	0.8271	0.8313	0.8354	0.8389	0.8423	0.8457	0.8492	0.8530	0.8568	0.8604	0.8641	0.8679	0.8715	0.8750	0.8783	0.8815
Cyprus	0.6425	0.6509	0.6594	0.6677	0.6763	0.6846	0.6924	0.7002	0.7082	0.7164	0.7246	0.7318	0.7393	0.7459	0.7526	0.7596	0.7657	0.7715	0.7776	0.7838	0.7908	0.7978	0.8050	0.8118	0.8188	0.8259	0.8334	0.8416	0.8499	0.8574	0.8641	0.8703	0.8749	0.8771	0.8788	0.8805
Denmark	0.7747	0.7808	0.7865	0.7919	0.7971	0.8021	0.8069	0.8111	0.8146	0.8178	0.8211	0.8245	0.8282	0.8316	0.8354	0.8398	0.8444	0.8494	0.8544	0.8594	0.8643	0.8685	0.8721	0.8751	0.8781	0.8809	0.8840	0.8872	0.8908	0.8939	0.8972	0.9005	0.9034	0.9059	0.9079	0.9096
Finland	0.7278	0.7338	0.7398	0.7458	0.7519	0.7578	0.7634	0.7689	0.7746	0.7802	0.7851	0.7887	0.7918	0.7948	0.7983	0.8025	0.8072	0.8125	0.8183	0.8243	0.8304	0.8360	0.8411	0.8456	0.8500	0.8541	0.8584	0.8632	0.8682	0.8720	0.8760	0.8802	0.8839	0.8872	0.8901	0.8929
France	0.6884	0.6935	0.6982	0.7026	0.7070	0.7114	0.7159	0.7206	0.7255	0.7307	0.7359	0.7407	0.7453	0.7492	0.7529	0.7562	0.7600	0.7638	0.7679	0.7719	0.7762	0.7804	0.7845	0.7882	0.7920	0.7959	0.8000	0.8042	0.8080	0.8114	0.8152	0.8191	0.8230	0.8268	0.8306	0.8343
Germany	0.7265	0.7334	0.7397	0.7457	0.7515	0.7575	0.7638	0.7703	0.7771	0.7844	0.7919	0.7994	0.8066	0.8129	0.8187	0.8240	0.8288	0.8334	0.8379	0.8425	0.8472	0.8521	0.8568	0.8611	0.8651	0.8689	0.8729	0.8771	0.8811	0.8840	0.8874	0.8910	0.8943	0.8972	0.9000	0.9026
Greece	0.6386	0.6464	0.6539	0.6609	0.6679	0.6749	0.6821	0.6888	0.6957	0.7025	0.7089	0.7154	0.7215	0.7270	0.7324	0.7378	0.7433	0.7492	0.7551	0.7610	0.7667	0.7722	0.7774	0.7824	0.7874	0.7920	0.7971	0.8024	0.8078	0.8126	0.8168	0.8194	0.8211	0.8223	0.8235	0.8246
Iceland	0.7224	0.7306	0.7388	0.7455	0.7522	0.7583	0.7642	0.7710	0.7774	0.7826	0.7866	0.7911	0.7953	0.7992	0.8031	0.8068	0.8109	0.8162	0.8226	0.8292	0.8356	0.8415	0.8457	0.8487	0.8522	0.8565	0.8615	0.8673	0.8730	0.8765	0.8786	0.8812	0.8841	0.8871	0.8901	0.8933
Ireland	0.6418	0.6519	0.6617	0.6713	0.6811	0.6908	0.7003	0.7094	0.7180	0.7266	0.7350	0.7429	0.7506	0.7577	0.7645	0.7717	0.7788	0.7862	0.7936	0.8012	0.8091	0.8168	0.8240	0.8300	0.8355	0.8414	0.8479	0.8543	0.8587	0.8607	0.8631	0.8663	0.8699	0.8742	0.8794	0.8852
Israel	0.7086	0.7143	0.7193	0.7238	0.7276	0.7312	0.7348	0.7386	0.7425	0.7463	0.7509	0.7558	0.7611	0.7661	0.7709	0.7758	0.7809	0.7855	0.7898	0.7940	0.7985	0.8027	0.8063	0.8092	0.8120	0.8147	0.8179	0.8212	0.8240	0.8263	0.8284	0.8307	0.8331	0.8358	0.8389	0.8424
Italy	0.7129	0.7210	0.7276	0.7336	0.7394	0.7448	0.7500	0.7551	0.7603	0.7654	0.7703	0.7751	0.7794	0.7832	0.7868	0.7903	0.7942	0.7982	0.8021	0.8058	0.8095	0.8129	0.8161	0.8193	0.8228	0.8258	0.8289	0.8326	0.8363	0.8393	0.8424	0.8457	0.8486	0.8513	0.8536	0.8559
Luxembourg	0.7312	0.7371	0.7427	0.7482	0.7540	0.7599	0.7667	0.7732	0.7797	0.7866	0.7932	0.8000	0.8062	0.8123	0.8181	0.8238	0.8294	0.8352	0.8411	0.8478	0.8547	0.8603	0.8645	0.8679	0.8707	0.8736	0.8787	0.8841	0.8882	0.8920	0.8955	0.8989	0.9021	0.9052	0.9082	0.9111
Malta	0.6110	0.6195	0.6273	0.6337	0.6390	0.6435	0.6478	0.6518	0.6561	0.6607	0.6652	0.6696	0.6738	0.6778	0.6819	0.6864	0.6934	0.7013	0.7099	0.7189	0.7283	0.7363	0.7438	0.7504	0.7560	0.7611	0.7666	0.7719	0.7769	0.7809	0.7848	0.7891	0.7933	0.7974	0.8017	0.8062
Netherlands	0.7434	0.7486	0.7529	0.7569	0.7611	0.7653	0.7696	0.7738	0.7782	0.7830	0.7882	0.7933	0.7983	0.8031	0.8079	0.8127	0.8173	0.8221	0.8269	0.8317	0.8366	0.8415	0.8458	0.8497	0.8535	0.8572	0.8613	0.8657	0.8703	0.8743	0.8782	0.8819	0.8852	0.8882	0.8909	0.8936
Norway	0.7712	0.7783	0.7844	0.7901	0.7959	0.8015	0.8068	0.8118	0.8163	0.8206	0.8248	0.8292	0.8340	0.8390	0.8443	0.8498	0.8558	0.8623	0.8687	0.8750	0.8812	0.8865	0.8912	0.8950	0.8986	0.9021	0.9057	0.9096	0.9140	0.9178	0.9216	0.9252	0.9287	0.9317	0.9343	0.9365
Portugal	0.5133	0.5215	0.5297	0.5375	0.5449	0.5522	0.5596	0.5673	0.5752	0.5832	0.5913	0.5997	0.6080	0.6157	0.6230	0.6302	0.6374	0.6445	0.6517	0.6588	0.6657	0.6723	0.6791	0.6857	0.6921	0.6983	0.7045	0.7107	0.7167	0.7221	0.7277	0.7330	0.7378	0.7425	0.7473	0.7522

Appendix Table 5. Socio-Demographic Index values for all estimated GBD 2015 locations, 1980–2015

Location	1980	1981	1982	1983	1984	1985	1986	1987	1988	1989	1990	1991	1992	1993	1994	1995	1996	1997	1998	1999	2000	2001	2002	2003	2004	2005	2006	2007	2008	2009	2010	2011	2012	2013	2014	2015
Spain	0.5816	0.5902	0.5987	0.6067	0.6146	0.6222	0.6299	0.6377	0.6456	0.6537	0.6620	0.6704	0.6785	0.6860	0.6934	0.7007	0.7074	0.7141	0.7206	0.7271	0.7338	0.7404	0.7469	0.7532	0.7592	0.7653	0.7717	0.7783	0.7848	0.7899	0.7950	0.8001	0.8050	0.8096	0.8143	0.8192
Sweden	0.7422	0.7473	0.7518	0.7557	0.7596	0.7632	0.7669	0.7706	0.7744	0.7785	0.7832	0.7881	0.7934	0.7990	0.8057	0.8129	0.8198	0.8266	0.8330	0.8390	0.8445	0.8488	0.8524	0.8554	0.8584	0.8611	0.8642	0.8677	0.8708	0.8733	0.8766	0.8799	0.8831	0.8861	0.8891	0.8921
Stockholm	0.7792	0.7861	0.7910	0.7957	0.7990	0.8034	0.8059	0.8091	0.8126	0.8168	0.8219	0.8277	0.8311	0.8358	0.8406	0.8481	0.8543	0.8608	0.8665	0.8723	0.8770	0.8797	0.8817	0.8836	0.8850	0.8874	0.8905	0.8940	0.8970	0.8996	0.9028	0.9064	0.9098	0.9111	0.9148	0.9175
Sweden except Stockholm	0.7339	0.7385	0.7428	0.7465	0.7505	0.7539	0.7577	0.7615	0.7653	0.7695	0.7740	0.7786	0.7844	0.7902	0.7973	0.8043	0.8114	0.8181	0.8246	0.8305	0.8361	0.8408	0.8447	0.8480	0.8514	0.8541	0.8572	0.8606	0.8637	0.8661	0.8692	0.8724	0.8754	0.8789	0.8816	0.8846
Switzerland	0.7779	0.7835	0.7886	0.7935	0.7985	0.8037	0.8090	0.8144	0.8198	0.8255	0.8314	0.8375	0.8430	0.8477	0.8521	0.8563	0.8603	0.8643	0.8685	0.8728	0.8774	0.8821	0.8865	0.8902	0.8935	0.8966	0.8997	0.9033	0.9073	0.9106	0.9138	0.9170	0.9200	0.9229	0.9256	0.9282
United Kingdom	0.7364	0.7414	0.7460	0.7507	0.7556	0.7609	0.7663	0.7721	0.7783	0.7846	0.7909	0.7965	0.8018	0.8069	0.8125	0.8180	0.8236	0.8294	0.8354	0.8416	0.8479	0.8537	0.8586	0.8628	0.8667	0.8701	0.8731	0.8759	0.8781	0.8796	0.8814	0.8835	0.8856	0.8878	0.8903	0.8930
England	0.7408	0.7458	0.7498	0.7544	0.7592	0.7644	0.7698	0.7753	0.7814	0.7874	0.7936	0.7993	0.8045	0.8096	0.8149	0.8205	0.8260	0.8319	0.8378	0.8439	0.8501	0.8559	0.8606	0.8647	0.8685	0.8720	0.8749	0.8777	0.8801	0.8814	0.8831	0.8850	0.8870	0.8890	0.8914	0.8939
East Midlands	0.7368	0.7418	0.7461	0.7509	0.7558	0.7613	0.7668	0.7718	0.7782	0.7835	0.7897	0.7955	0.8008	0.8064	0.8113	0.8172	0.8231	0.8292	0.8344	0.8404	0.8460	0.8521	0.8566	0.8610	0.8653	0.8681	0.8714	0.8738	0.8756	0.8770	0.8781	0.8794	0.8816	0.8837	0.8860	0.8886
East of England	0.6853	0.6901	0.6957	0.7003	0.7047	0.7107	0.7159	0.7212	0.7269	0.7332	0.7399	0.7461	0.7511	0.7559	0.7615	0.7683	0.7749	0.7808	0.7871	0.7935	0.7990	0.8049	0.8096	0.8142	0.8184	0.8220	0.8251	0.8272	0.8289	0.8301	0.8319	0.8337	0.8348	0.8359	0.8372	0.8386
Greater London	0.8159	0.8208	0.8237	0.8281	0.8325	0.8373	0.8425	0.8473	0.8525	0.8587	0.8655	0.8708	0.8745	0.8793	0.8834	0.8879	0.8928	0.8977	0.9032	0.9088	0.9140	0.9191	0.9227	0.9258	0.9293	0.9320	0.9344	0.9368	0.9407	0.9420	0.9439	0.9471	0.9494	0.9525	0.9557	0.9590
North East England	0.6542	0.6591	0.6635	0.6688	0.6742	0.6788	0.6851	0.6928	0.7008	0.7085	0.7147	0.7197	0.7263	0.7325	0.7395	0.7450	0.7520	0.7587	0.7653	0.7722	0.7795	0.7858	0.7904	0.7957	0.7999	0.8031	0.8065	0.8106	0.8133	0.8153	0.8159	0.8183	0.8211	0.8238	0.8267	0.8298
North West England	0.7298	0.7346	0.7382	0.7424	0.7476	0.7525	0.7583	0.7648	0.7709	0.7774	0.7829	0.7885	0.7951	0.8007	0.8069	0.8127	0.8174	0.8237	0.8300	0.8358	0.8421	0.8479	0.8532	0.8576	0.8606	0.8641	0.8675	0.8707	0.8728	0.8741	0.8758	0.8779	0.8800	0.8820	0.8843	0.8869
South East England	0.7633	0.7682	0.7736	0.7780	0.7830	0.7878	0.7932	0.7975	0.8031	0.8086	0.8146	0.8204	0.8249	0.8294	0.8342	0.8399	0.8457	0.8512	0.8561	0.8620	0.8682	0.8740	0.8788	0.8826	0.8868	0.8905	0.8925	0.8951	0.8970	0.8980	0.8994	0.9006	0.9024	0.9045	0.9068	0.9093
South West England	0.7435	0.7487	0.7528	0.7581	0.7628	0.7681	0.7732	0.7786	0.7847	0.7899	0.7966	0.8030	0.8080	0.8126	0.8177	0.8240	0.8288	0.8348	0.8407	0.8469	0.8539	0.8599	0.8641	0.8683	0.8730	0.8769	0.8798	0.8820	0.8838	0.8849	0.8858	0.8869	0.8890	0.8911	0.8935	0.8961
West Midlands	0.7326	0.7374	0.7401	0.7446	0.7494	0.7547	0.7601	0.7659	0.7729	0.7778	0.7837	0.7893	0.7953	0.8005	0.8057	0.8110	0.8157	0.8211	0.8276	0.8333	0.8392	0.8446	0.8495	0.8537	0.8569	0.8606	0.8643	0.8666	0.8693	0.8707	0.8728	0.8737	0.8752	0.8768	0.8786	0.8806
Yorkshire and the Humber	0.7297	0.7347	0.7392	0.7432	0.7485	0.7538	0.7587	0.7651	0.7719	0.7784	0.7841	0.7896	0.7950	0.8000	0.8059	0.8102	0.8161	0.8226	0.8282	0.8340	0.8406	0.8461	0.8516	0.8561	0.8597	0.8633	0.8662	0.8697	0.8715	0.8721	0.8745	0.8768	0.8783	0.8797	0.8814	0.8833
Northern Ireland	0.6186	0.6278	0.6354	0.6409	0.6461	0.6551	0.6604	0.6706	0.6796	0.6916	0.6995	0.7069	0.7149	0.7220	0.7297	0.7357	0.7412	0.7488	0.7565	0.7639	0.7731	0.7773	0.7846	0.7912	0.7955	0.7997	0.8029	0.8053	0.8070	0.8096	0.8113	0.8125	0.8144	0.8165	0.8190	0.8216
Scotland	0.7442	0.7488	0.7572	0.7629	0.7683	0.7735	0.7794	0.7858	0.7922	0.7996	0.8051	0.8090	0.8142	0.8201	0.8261	0.8311	0.8371	0.8417	0.8483	0.8546	0.8606	0.8656	0.8710	0.8757	0.8792	0.8821	0.8855	0.8877	0.8890	0.8911	0.8947	0.8983	0.9015	0.9047	0.9082	0.9118
Wales	0.7128	0.7179	0.7215	0.7271	0.7322	0.7376	0.7433	0.7500	0.7567	0.7641	0.7707	0.7772	0.7825	0.7878	0.7946	0.8000	0.8052	0.8113	0.8187	0.8261	0.8329	0.8394	0.8458	0.8504	0.8548	0.8582	0.8623	0.8655	0.8676	0.8700	0.8708	0.8731	0.8763	0.8796	0.8831	0.8869
Southern Latin America	0.5788	0.5846	0.5894	0.5941	0.5986	0.6023	0.6074	0.6124	0.6171	0.6216	0.6261	0.6318	0.6389	0.6464	0.6543	0.6619	0.6703	0.6786	0.6862	0.6924	0.6979	0.7025	0.7059	0.7096	0.7140	0.7191	0.7248	0.7312	0.7375	0.7434	0.7497	0.7566	0.7631	0.7691	0.7746	0.7797
Argentina	0.5768	0.5819	0.5868	0.5920	0.5969	0.6006	0.6062	0.6114	0.6159	0.6196	0.6234	0.6285	0.6352	0.6425	0.6504	0.6576	0.6660	0.6743	0.6820	0.6881	0.6934	0.6975	0.7001	0.7032	0.7070	0.7117	0.7172	0.7234	0.7298	0.7357	0.7422	0.7493	0.7557	0.7617	0.7671	0.7720
Chile	0.5872	0.5941	0.5986	0.6023	0.6062	0.6100	0.6141	0.6187	0.6241	0.6306	0.6371	0.6444	0.6528	0.6613	0.6697	0.6786	0.6876	0.6960	0.7036	0.7101	0.7164	0.7223	0.7278	0.7332	0.7391	0.7453	0.7519	0.7587	0.7649	0.7703	0.7762	0.7823	0.7885	0.7944	0.7998	0.8048
Uruguay	0.5662	0.5734	0.5787	0.5826	0.5859	0.5887	0.5925	0.5968	0.6008	0.6047	0.6086	0.6128	0.6183	0.6239	0.6301	0.6355	0.6414	0.6476	0.6538	0.6591	0.6636	0.6671	0.6694	0.6719	0.6748	0.6786	0.6831	0.6885	0.6948	0.7013	0.7086	0.7164	0.7238	0.7312	0.7383	0.7450
High-income North America	0.8377	0.8410	0.8431	0.8452	0.8479	0.8508	0.8534	0.8557	0.8579	0.8602	0.8623	0.8641	0.8662	0.8683	0.8706	0.8731	0.8760	0.8790	0.8820	0.8850	0.8881	0.8907	0.8931	0.8954	0.8978	0.9005	0.9035	0.9068	0.9101	0.9131	0.9162	0.9194	0.9227	0.9258	0.9288	0.9317
Canada	0.8144	0.8201	0.8247	0.8290	0.8334	0.8379	0.8421	0.8461	0.8504	0.8546	0.8581	0.8607	0.8633	0.8660	0.8694	0.8733	0.8773	0.8815	0.8857	0.8902	0.8949	0.8990	0.9025	0.9055	0.9082	0.9107	0.9131	0.9156	0.9182	0.9202	0.9226	0.9255	0.9284	0.9314	0.9345	0.9375
Greenland	0.5788	0.5883	0.5985	0.6169	0.6158	0.6079	0.6281	0.6275	0.6094	0.6155	0.6059	0.6154	0.5999	0.6086	0.6148	0.6207	0.6275	0.6089	0.6406	0.6481	0.6620	0.6459	0.6420	0.6688	0.6648	0.6729	0.6906	0.6907	0.7003	0.6893	0.7030	0.7252	0.7439	0.7412	0.7506	0.7575
United States	0.8403	0.8433	0.8451	0.8470	0.8496	0.8522	0.8547	0.8568	0.8588	0.8608	0.8628	0.8646	0.8666	0.8686	0.8708	0.8732	0.8759	0.8788	0.8816	0.8845	0.8874	0.8898	0.8921	0.8943	0.8967	0.8994	0.9025	0.9059	0.9093	0.9123	0.9155	0.9188	0.9221	0.9252	0.9282	0.9311
Alabama	0.8073	0.8121	0.8154	0.8175	0.8202	0.8240	0.8268	0.8299	0.8323	0.8346	0.8384	0.8414	0.8447	0.8476	0.8509	0.8537	0.8566	0.8588	0.8618	0.8646	0.8674	0.8723	0.8760	0.8790	0.8824	0.8851	0.8877	0.8904	0.8930	0.8966	0.9005	0.9034	0.9071	0.9096	0.9124	0.9153
Alaska	0.8805	0.8768	0.8723	0.8714	0.8702	0.8701	0.8738	0.8740	0.8784	0.8770	0.8757	0.8747	0.8724	0.8757	0.8779	0.8801	0.8819	0.8827	0.8849	0.8866	0.8901	0.8899	0.8923	0.8951	0.8964	0.8994	0.9017	0.9062	0.9055	0.9095	0.9113	0.9162	0.9233	0.9243	0.9285	0.9324
Arizona	0.8369	0.8392	0.8412	0.8412	0.8440	0.8443	0.8466	0.8481	0.8498	0.8524	0.8527	0.8548	0.8556	0.8569	0.8586	0.8599	0.8604	0.8646	0.8671	0.8684	0.8698	0.8721	0.8730	0.8743	0.8758	0.8790	0.8817	0.8873	0.8931	0.8995	0.9043	0.9080	0.9101	0.9132	0.9156	0.9179
Arkansas	0.8287	0.8335	0.8355	0.8364	0.8388	0.8412	0.8438	0.8450	0.8459	0.8467	0.8483	0.8508	0.8532	0.8557	0.8564	0.8570	0.8571	0.8593	0.8620	0.8650	0.8666	0.8692	0.8699	0.8725	0.8739	0.8761	0.8783	0.8818	0.8853	0.8881	0.8915	0.8937	0.8980	0.9019	0.9058	0.9095
California	0.8541	0.8553	0.8572	0.8580	0.8594	0.8606	0.8616	0.8619	0.8618	0.8612	0.8591	0.8591	0.8605	0.8617	0.8636	0.8654	0.8686	0.8735	0.8784	0.8824	0.8858	0.8888	0.8909	0.8929	0.8952	0.8976	0.9010	0.9045	0.9085	0.9128	0.9159	0.9195	0.9227	0.9271	0.9296	0.9321
Colorado	0.8725	0.8731	0.8739	0.8746	0.8771	0.8792	0.8794	0.8819	0.8835	0.8866	0.8876	0.8876	0.8878	0.8903	0.8927	0.8947	0.8960	0.8983	0.8992	0.9004	0.9025	0.9035	0.9040	0.9059	0.9090	0.9113	0.9150	0.9196	0.9229	0.9257	0.9291	0.9335	0.9372	0.9409	0.9444	0.9478
Connecticut	0.8718	0.8742	0.8767	0.8782	0.8808	0.8837	0.8862	0.8880	0.8919	0.8955	0.8991	0.9016	0.9034	0.9048	0.9068	0.9094	0.9110	0.9149	0.9166	0.9198	0.9245	0.9261	0.9287	0.9300	0.9333	0.9355	0.9387	0.9415	0.9450	0.9485	0.9511	0.9537	0.9578	0.9608	0.9635	0.9660
Delaware	0.8633	0.8667	0.8684	0.8688	0.8712	0.8729	0.8748	0.8770	0.8778	0.8805	0.8822	0.8831	0.8874	0.8883	0.8898	0.8920	0.8946	0.8957	0.8963	0.8979	0.8993	0.9033	0.9030	0.9054	0.9086	0.9105	0.9139	0.9166	0.9187	0.9229	0.9243	0.9272	0.9318	0.9357	0.9380	0.9403
District of Columbia	0.8673	0.8706	0.8720	0.8731	0.8737	0.8761	0.8778	0.8795	0.8810	0.8770	0.8798	0.8803	0.8876	0.8912	0.8987	0.9065	0.9140	0.9210	0.9283	0.9343	0.9377	0.9406	0.9443	0.9459	0.9454	0.9478	0.9488	0.9507	0.9518	0.9559	0.9571	0.9601	0.9638	0.9688	0.9734	0.9779
Florida	0.8414	0.8439	0.8456	0.8467	0.8488	0.8514	0.8540	0.8558	0.8574	0.8600	0.8628	0.8670	0.8697	0.8706	0.8734	0.8763	0.8791	0.8812	0.8845	0.8876	0.8900	0.8919	0.8951	0.8970	0.8994	0.9016	0.9050	0.9089	0.9132	0.9169	0.9198	0.9229	0.9261	0.9278	0.9291	0.9304
Georgia	0.8166	0.8218	0.8248	0.8271	0.8304	0.8338	0.8371	0.8394	0.8426	0.8454	0.8492	0.8534	0.8558	0.8588	0.8621	0.8646	0.8680	0.8697	0.8730	0.8751	0.8777	0.8799	0.8830	0.8854	0.8868	0.8885	0.8912	0.8948	0.8991	0.9024	0.9064	0.9089	0.9126	0.9152	0.9182	0.9213
Hawaii	0.8516	0.8533	0.8527	0.8510	0.8539	0.8588	0.8600	0.8615	0.8633	0.8667	0.8674	0.8715	0.8732	0.8750	0.8760	0.8806	0.8822	0.8883	0.8892	0.8920	0.8922	0.8946	0.8943	0.8948	0.8979	0.9035	0.9049	0.9083	0.9096	0.9138	0.9147	0.9179	0.9212	0.9241	0.9272	0.9299
Idaho	0.8154	0.8195	0.8203	0.8230	0.8276	0.8320	0.8364	0.8392	0.8422	0.8455	0.8472	0.8485	0.8486	0.8519	0.8554	0.8568	0.8583	0.8624	0.8639	0.8661	0.8700	0.8705	0.8720	0.8732	0.8743	0.8773	0.8806	0.8834	0.8854	0.8909	0.8928	0.8979	0.8981	0.9028	0.9053	0.9076
Illinois	0.8466	0.8505	0.8526	0.8543	0.8563	0.8591	0.8621	0.8631	0.8649	0.8667	0.8687	0.8701	0.8726	0.8737	0.8756	0.8782	0.8820	0.8855	0.8886	0.8918	0.8950	0.8967	0.8997	0.9017	0.9044	0.9076	0.9119	0.9158	0.9194	0.9225	0.9253	0.9288	0.9323	0.9354	0.9385	0.9414
Indiana	0.8272	0.8318	0.8336	0.8358	0.8386	0.8414	0.8443	0.8477	0.8487	0.8515	0.8537	0.8556	0.8587	0.8605	0.8637	0.8653	0.8676	0.8701	0.8725	0.8745	0.8775	0.8800	0.8828	0.8849	0.8866	0.8892	0.8928	0.8951	0.8974	0.8992	0.9016	0.9036	0.9068	0.9094	0.9126	0.9158
Iowa	0.8414	0.8459	0.8482	0.8494	0.8520	0.8557	0.8594	0.8618	0.8633	0.8642	0.8663	0.8671	0.8689	0.8699	0.8722	0.8737	0.8754	0.8785	0.8805	0.8823	0.8847	0.8866	0.8882	0.8900	0.8922	0.8935	0.8962	0.9003	0.9045	0.9070	0.9104	0.9144	0.9172	0.9195	0.9243	0.9284
Kansas	0.8630	0.8649	0.8679	0.8688	0.8714	0.8746	0.8758	0.8784	0.8797	0.8816	0.8826	0.8836	0.8830	0.8834	0.8834	0.8837	0.8859	0.8858	0.8863	0.8874	0.8891	0.8911	0.8912	0.8939	0.8955	0.8975	0.9004	0.9030	0.9062	0.9085	0.9111	0.9160	0.9181	0.9240	0.9275	0.9308
Kentucky	0.7920	0.7969	0.7997	0.8029	0.8071	0.8114	0.8146	0.8180	0.8222	0.8240	0.8276	0.8303	0.8339	0.8378	0.8407	0.8442	0.8476	0.8504	0.8539	0.8577	0.8609	0.8652	0.8685	0.8713	0.8738	0.8764	0.8793	0.8819	0.8852	0.8869	0.8903	0.8931	0.8952	0.8975	0.9003	0.9030
Louisiana	0.8195	0.8240	0.8258	0.8287	0.8312	0.8337	0.8363	0.8391	0.8390	0.8415	0.8441	0.8434	0.8461	0.8479	0.8509	0.8547	0.8575	0.8588	0.8612	0.8631	0.8656	0.8697	0.8722	0.8755	0.8781	0.8867	0.8840	0.8871	0.8926	0.8956	0.9012	0.9051	0.9081	0.9105	0.9144	0.9181
Maine	0.8257	0.8283	0.8300	0.8314	0.8345	0.8386	0.8424	0.8464	0.8508	0.8553	0.8604	0.8628	0.8659	0.8698	0.8729	0.8759	0.8785	0.8811	0.8838	0.8874	0.8919	0.8933	0.8970	0.8997	0.9026	0.9049	0.9094	0.9130	0.9171	0.9184	0.9213	0.9248	0.9269	0.9290	0.9322	0.9352
Maryland	0.8691	0.8706	0.8705	0.8715	0.8735	0.8758	0.8780	0.8794	0.8817	0.8847	0.8875	0.8892	0.8911	0.8939	0.8956	0.8975	0.9000	0.9028	0.9039	0.9064	0.9085	0.9117	0.9142	0.9165	0.9201	0.9233	0.9263	0.9297	0.9325	0.9362	0.9390	0.9425	0.9460	0.9496	0.9514	0.9532
Massachusetts	0.8636	0.8665	0.8686	0.8710	0.8743	0.8777	0.8815	0.8847	0.8876	0.8904	0.8938	0.8974	0.8990	0.9014	0.9034	0.9065	0.9099	0.9122	0.9145	0.9177	0.9217	0.9242	0.9262	0.9293	0.9324	0.9358	0.9395	0.9428	0.9456	0.9487	0.9517	0.9541	0.9583	0.9619	0.9646	0.9670
Michigan	0.8536	0.8567	0.8580	0.8596	0.8598	0.8628	0.8650	0.8661	0.8693	0.8683	0.8690	0.8706	0.8737	0.8756	0.8773	0.8800	0.8828	0.8843	0.8871	0.8894	0.8920	0.8944	0.8973	0.8990	0.9012	0.9041	0.9075	0.9107	0.9129	0.9140	0.9151	0.9169	0.9200	0.9221	0.9260	0.9299
Minnesota	0.8368	0.8406	0.8445	0.8491	0.8521	0.8569	0.8617	0.8662	0.8692	0.8733	0.8771	0.8793	0.8821	0.8842	0.8862	0.8889	0.8914	0.8931	0.8964	0.8991	0.9026	0.9046	0.9062	0.9075	0.9096	0.9123	0.9146	0.9190	0.9226	0.9251	0.9279	0.9304	0.9333	0.9357	0.9389	0.9420
Mississippi	0.8027	0.8080	0.8105	0.8136	0.8158	0.8195	0.8230	0.8254	0.8265	0.8281	0.8308	0.8323	0.8345	0.8369	0.8398	0.8433	0.8472	0.8492	0.8508	0.8543	0.8558	0.8600	0.8630	0.8646	0.8669	0.8709	0.8687	0.8723	0.8770	0.8818	0.8879	0.8904	0.8955	0.8980	0.9016	0.9050
Missouri	0.8267	0.8311	0.8334	0.8356	0.8396	0.8421	0.8461	0.8494	0.8518	0.8542	0.8568	0.8581	0.8614	0.8635	0.8670	0.8691	0.8711	0.8737	0.8760	0.8789	0.8825	0.8851	0.8875	0.8893	0.8915	0.8938	0.8965	0.9001	0.9029	0.9057	0.9084	0.9109	0.9144	0.9169	0.9206	0.9241
Montana	0.8313	0.8326	0.8328	0.8351	0.8356	0.8404	0.8431	0.8454	0.8490	0.8513	0.8536	0.8544	0.8558	0.8583	0.8623	0.8638	0.8683	0.8703	0.8738	0.8764	0.8795	0.8803	0.8818	0.8836	0.8876	0.8912	0.8916	0.8977	0.9002	0.9037	0.9065	0.9097	0.9133	0.9154	0.9211	0.9268
Nebraska	0.8381	0.8413	0.8438	0.8456	0.8481	0.8527	0.8562	0.8593	0.8618	0.8638	0.8665	0.8680	0.8709	0.8718	0.8729	0.8740	0.8769	0.8790	0.8819	0.8834	0.8853	0.8856	0.8853	0.8876	0.8890	0.8929	0.8965	0.9005	0.9029	0.9044	0.9095	0.9131	0.9163	0.9191	0.9221	0.9246
Nevada	0.8819	0.8822	0.8832	0.8845	0.8844	0.8854	0.8842	0.8840	0.8826	0.8818	0.8798	0.8818	0.8829	0.8843	0.8829	0.8827	0.8840	0.8872	0.8877	0.8908	0.8928	0.8939	0.8932	0.8949	0.8962	0.8972	0.8990	0.9027	0.9083	0.9119	0.9147	0.9167	0.9198	0.9214	0.9208	0.9202
New Hampshire	0.8504	0.8556	0.8570	0.8610	0.8644	0.8661	0.8698	0.8714	0.8764	0.8805	0.8853	0.8891	0.8902	0.8919	0.8935	0.8962	0.8991	0.9018	0.9047	0.9094	0.9116	0.9135	0.9165	0.9200	0.9217	0.9250	0.9298	0.9341	0.9379	0.9395	0.9426	0.9446	0.9504	0.9529	0.9564	0.9599
New Jersey	0.8832	0.8862	0.8875	0.8886	0.8907	0.8925	0.8932	0.8939	0.8955	0.8973	0.9008	0.9009	0.9020	0.9029	0.9027	0.9044	0.9061	0.9081	0.9099	0.9120	0.9147	0.9151	0.9170	0.9179	0.9207	0.9235	0.9269	0.9294	0.9332	0.9355	0.9384	0.9411	0.9450	0.9486	0.9523	0.9559
New Mexico	0.8184	0.8200	0.8203	0.8219	0.8253	0.8281	0.8309	0.8328	0.8358	0.8378	0.8406	0.8397	0.8409	0.8424	0.8462	0.8508	0.8529	0.8563	0.8589	0.8618	0.8658	0.8674	0.8685	0.8725	0.8745	0.8770	0.8799	0.8829	0.8862	0.8908	0.8952	0.8993	0.9029	0.9069	0.9090	0.9109
New York	0.8478	0.8501	0.8518	0.8535	0.8568	0.8597	0.8620	0.8640	0.8670	0.8698	0.8735	0.8760	0.8787	0.8810	0.8832	0.8860	0.8903	0.8946	0.8981	0.9016	0.9048	0.9075	0.9098	0.9121	0.9152	0.9186	0.9223	0.9256	0.9289	0.9310	0.9334	0.9373	0.9408	0.9449	0.9482	0.9513
North Carolina	0.8117	0.8155	0.8171	0.8204	0.8234	0.8269	0.8302	0.8328	0.8355	0.8385	0.8425	0.8469	0.8489	0.8539	0.8575	0.8610	0.8637	0.8665	0.8691	0.8723	0.8734	0.8773	0.8803	0.8835	0.8857	0.8875	0.8908	0.8942	0.8971	0.9009	0.9043	0.9075	0.9107	0.9133	0.9155	0.9176
North Dakota	0.8192	0.8200	0.8214	0.8237	0.8295	0.8323	0.8379	0.8416	0.8431	0.8480	0.8528	0.8547	0.8562	0.8579	0.8607	0.8629	0.8677	0.8695	0.8766	0.8810	0.8846	0.8862	0.8869	0.8893	0.8913	0.8933	0.8970	0.9015	0.9052	0.9080	0.9119	0.9143	0.9186	0.9227	0.9302	0.9361
Ohio	0.8313	0.8342	0.8364	0.8388	0.8413	0.8442	0.8471	0.8498	0.8521	0.8546	0.8574	0.8586	0.8612	0.8634	0.8660	0.8681	0.8715	0.8735	0.8766	0.8793	0.8820	0.8852	0.8884	0.8911	0.8938	0.8965	0.8997	0.9028	0.9049	0.9070	0.9099	0.9125	0.9150	0.9174	0.9214	0.9253
Oklahoma	0.8329	0.8355	0.8346	0.8387	0.8431	0.8468	0.8498	0.8528	0.8534	0.8550	0.8563	0.8555	0.8561	0.8581	0.8595	0.8600	0.8614	0.8603	0.8620	0.8657	0.8687	0.8698	0.8719	0.8749	0.8774	0.8805	0.8839	0.8874	0.8911	0.8930	0.8965	0.9013	0.9051	0.9080	0.9116	0.9149
Oregon	0.8594	0.8610	0.8640	0.8640	0.8654	0.8672	0.8682	0.8696	0.8699	0.8711	0.8721	0.8744	0.8763	0.8788	0.8801	0.8805	0.8824	0.8854	0.8870	0.8905	0.8940	0.8965	0.8990	0.9009	0.9033	0.9059	0.9072	0.9106	0.9133	0.9174	0.9205	0.9234	0.9267	0.9292	0.9336	0.9380
Pennsylvania	0.8506	0.8527	0.8541	0.8558	0.8583	0.8601	0.8615	0.8632	0.8653	0.8677	0.8700	0.8719	0.8742	0.8761	0.8784	0.8812	0.8844	0.8877	0.8895	0.8918	0.8949	0.8973	0.8992	0.9009	0.9041	0.9067	0.9099	0.9131	0.9160	0.9186	0.9212	0.9240	0.9277	0.9312	0.9350	0.9387
Rhode Island	0.8363	0.8392	0.8421	0.8439	0.8480	0.8520	0.8543	0.8567	0.8612	0.8644	0.8673	0.8709	0.8729	0.8761	0.8799	0.8847	0.8879	0.8913	0.8945	0.8986	0.9021	0.9032	0.9050	0.9073	0.9124	0.9151	0.9207	0.9237	0.9269	0.9311	0.9330	0.9362	0.9395	0.9424	0.9470	0.9513
South Carolina	0.8022	0.8059	0.8087	0.8112	0.8149	0.8184	0.8217	0.8246	0.8264	0.8292	0.8332	0.8374	0.8414	0.8464	0.8515	0.8556	0.8589	0.8613	0.8642	0.8668	0.8704	0.8734	0.8778	0.8805	0.8829	0.8852	0.8857	0.8899	0.8925	0.8969	0.9004	0.9035	0.9067	0.9096	0.9126	0.9156
South Dakota	0.8102	0.8154	0.8165	0.8182	0.8212	0.8263	0.8300	0.8324	0.8366	0.8399	0.8437	0.8450	0.8457	0.8497	0.8542	0.8562	0.8599	0.8647	0.8677	0.8693	0.8763	0.8765	0.8771	0.8790	0.8807	0.8838	0.8860	0.8894	0.8942	0.8970	0.9004	0.9036	0.9065	0.9090	0.9137	0.9174
Tennessee	0.8022	0.8071	0.8097	0.8124	0.8164	0.8198	0.8237	0.8268	0.8293	0.8325	0.8363	0.8397	0.8438	0.8479	0.8517	0.8555	0.8595	0.8630	0.8658	0.8697	0.8733	0.8767	0.8801	0.8827	0.8853	0.8869	0.8901	0.8922	0.8950	0.8988	0.9019	0.9040	0.9066	0.9095	0.9127	0.9158
Texas	0.8223	0.8258	0.8276	0.8309	0.8333	0.8357	0.8379	0.8405	0.8421	0.8447	0.8460	0.8465	0.8469	0.8478	0.8501	0.8518	0.8531	0.8561	0.8596	0.8623	0.8651	0.8677	0.8690	0.8721	0.8745	0.8774	0.8817	0.8855	0.8894	0.8919	0.8970	0.9025	0.9060	0.9088	0.9117	0.9143
Utah	0.8021	0.8073	0.8108	0.8164	0.8225	0.8289	0.8327	0.8367	0.8367	0.8417	0.8446	0.8480	0.8482	0.8514	0.8524	0.8526	0.8517	0.8542	0.8552	0.8572	0.8613	0.8623	0.8627	0.8657	0.8675	0.8703	0.8747	0.8786	0.8812	0.8857	0.8894	0.8938	0.8966	0.9007	0.9046	0.9084
Vermont	0.8500	0.8525	0.8543	0.8558	0.8581	0.8622	0.8638	0.8675	0.8722	0.8739	0.8794	0.8814	0.8830	0.8850	0.8860	0.8917	0.8929	0.8957	0.8984	0.9008	0.9051	0.9074	0.9098	0.9103	0.9129	0.9186	0.9213	0.9250	0.9287	0.9322	0.9319	0.9359	0.9395	0.9429	0.9467	0.9502
Virginia	0.8601	0.8630	0.8642	0.8662	0.8683	0.8706	0.8730	0.8748	0.8775	0.8796	0.8820	0.8840	0.8851	0.8874	0.8883	0.8909	0.8931	0.8954	0.8965	0.8986	0.9008	0.9028	0.9046	0.9074	0.9091	0.9127	0.9164	0.9205	0.9246	0.9275	0.9306	0.9338	0.9371	0.9405	0.9434	0.9461
Washington	0.8626	0.8647	0.8674	0.8686	0.8707	0.8729	0.8754	0.8771	0.8787	0.8805	0.8815	0.8828	0.8850	0.8870	0.8897	0.8913	0.8929	0.8956	0.8986	0.9020	0.9056	0.9088	0.9115	0.9134	0.9154	0.9177	0.9199	0.9230	0.9245	0.9269	0.9302	0.9325	0.9360	0.9398	0.9432	0.9467
West Virginia	0.7910	0.7966	0.7995	0.8026	0.8069	0.8105	0.8135	0.8170	0.8208	0.8231	0.8261	0.8288	0.8326	0.8362	0.8401	0.8429	0.8472	0.8500	0.8535	0.8561	0.8595	0.8625	0.8644	0.8676	0.8707	0.8738	0.8785	0.8788	0.8823	0.8845	0.8883	0.8902	0.8929	0.8951	0.8990	0.9027
Wisconsin	0.8274	0.8311	0.8335	0.8359	0.8384	0.8420	0.8455	0.8500	0.8542	0.8576	0.8610	0.8636	0.8669	0.8695	0.8732	0.8760	0.8795	0.8827	0.8855	0.8877	0.8911	0.8928	0.8955	0.8974	0.9000	0.9020	0.9055	0.9090	0.9113	0.9136	0.9167	0.9193	0.9225	0.9254	0.9276	0.9295
Wyoming	0.8565	0.8587	0.8590	0.8622	0.8640	0.8666	0.8697	0.8752	0.8769	0.8797	0.8795	0.8809	0.8799	0.8816	0.8834	0.8855	0.8862	0.8859	0.8903	0.8941	0.8966	0.8991	0.8971	0.8995	0.9026	0.9022	0.9060	0.9111	0.9141	0.9184	0.9233	0.9282	0.9314	0.9343	0.9373	0.9400
Latin America and Caribbean	0.4475	0.4566	0.4652	0.4732	0.4810	0.4888	0.4967	0.5044	0.5119	0.5189	0.5256	0.5322	0.5387	0.5451	0.5515	0.5578	0.5642	0.5706	0.5768	0.5827	0.5885	0.5946	0.6004	0.6060	0.6119	0.6180	0.6246	0.6313	0.6380	0.6439	0.6498	0.6559	0.6618	0.6675	0.6731	0.6781
Caribbean	0.5033	0.5099	0.5158	0.5213	0.5268	0.5322	0.5375	0.5429	0.5483	0.5534	0.5580	0.5621	0.5657	0.5687	0.5715	0.5745	0.5780	0.5818	0.5858	0.5903	0.5951	0.6000	0.6049	0.6095	0.6139	0.6186	0.6239	0.6293	0.6343	0.6391	0.6434	0.6480	0.6524	0.6568	0.6612	0.6655
Antigua and Barbuda	0.6574	0.6643	0.6704	0.6773	0.6850	0.6935	0.7022	0.7111	0.7210	0.7299	0.7374	0.7445	0.7500	0.7546	0.7589	0.7616	0.7665	0.7713	0.7760	0.7803	0.7842	0.7874	0.7908	0.7945	0.7989	0.8039	0.8097	0.8164	0.8227	0.8270	0.8297	0.8319	0.8342	0.8363	0.8386	0.8410
The Bahamas	0.6491	0.6558	0.6623	0.6691	0.6772	0.6860	0.6941	0.7019	0.7088	0.7151	0.7205	0.7251	0.7289	0.7328	0.7373	0.7426	0.7488	0.7558	0.7636	0.7721	0.7805	0.7889	0.7966	0.8027	0.8075	0.8116	0.8149	0.8178	0.8201	0.8217	0.8236	0.8254	0.8276	0.8299	0.8323	0.8348
Barbados	0.6178	0.6249	0.6310	0.6363	0.6422	0.6482	0.6548	0.6618	0.6691	0.6765	0.6829	0.6882	0.6924	0.6964	0.7004	0.7044	0.7090	0.7141	0.7191	0.7239	0.7288	0.7338	0.7384	0.7431	0.7474	0.7521	0.7569	0.7614	0.7652	0.7683	0.7708	0.7734	0.7758	0.7780	0.7802	0.7824
Belize	0.3743	0.3863	0.3971	0.4073	0.4171	0.4266	0.4347	0.4442	0.4540	0.4641	0.4745	0.4833	0.4918	0.5000	0.5077	0.5153	0.5257	0.5356	0.5449	0.5537	0.5626	0.5714	0.5801	0.5891	0.5981	0.6068	0.6143	0.6213	0.6276	0.6332	0.6385	0.6443	0.6498	0.6549	0.6600	0.6652
Bermuda	0.7780	0.7853	0.7922	0.7989	0.8051	0.8112	0.8175	0.8237	0.8297	0.8352	0.8393	0.8405	0.8448	0.8542	0.8562	0.8590	0.8615	0.8632	0.8679	0.8707	0.8740	0.8789	0.8828	0.8864	0.8902	0.8933	0.8995	0.9033	0.9069	0.9094	0.9114	0.9135	0.9151	0.9166	0.9182	0.9164
Cuba	0.5976	0.6063	0.6142	0.6216	0.6290	0.6358	0.6422	0.6481	0.6541	0.6597	0.6647	0.6680	0.6695	0.6690	0.6683	0.6677	0.6681	0.6689	0.6702	0.6730	0.6771	0.6821	0.6873	0.6927	0.6983	0.7049	0.7126	0.7203	0.7275	0.7339	0.7398	0.7458	0.7515	0.7570	0.7619	0.7662
Dominica	0.5220	0.5210	0.5397	0.5468	0.5547	0.5629	0.5712	0.5781	0.5866	0.5933	0.5992	0.6140	0.6062	0.6141	0.6257	0.6372	0.6462	0.6550	0.6635	0.6712	0.6784	0.6836	0.6884	0.6938	0.6991	0.7039	0.7087	0.7140	0.7195	0.7247	0.7300	0.7351	0.7394	0.7434	0.7479	0.7526

Appendix Table 5. Socio-Demographic Index values for all estimated GBD 2015 locations, 1980–2015

Location	1980	1981	1982	1983	1984	1985	1986	1987	1988	1989	1990	1991	1992	1993	1994	1995	1996	1997	1998	1999	2000	2001	2002	2003	2004	2005	2006	2007	2008	2009	2010	2011	2012	2013	2014	2015
Dominican Republic	0.4344	0.4437	0.4523	0.4607	0.4685	0.4756	0.4826	0.4899	0.4971	0.5039	0.5096	0.5151	0.5213	0.5281	0.5348	0.5418	0.5491	0.5568	0.5647	0.5726	0.5803	0.5875	0.5946	0.6007	0.6065	0.6130	0.6202	0.6280	0.6353	0.6422	0.6495	0.6565	0.6632	0.6699	0.6768	0.6837
Grenada	0.4761	0.4858	0.4958	0.5055	0.5149	0.5242	0.5279	0.5322	0.5362	0.5411	0.5468	0.5614	0.5762	0.5898	0.6024	0.6136	0.6236	0.6329	0.6423	0.6521	0.6618	0.6706	0.6791	0.6879	0.6954	0.7035	0.7102	0.7169	0.7231	0.7278	0.7323	0.7365	0.7404	0.7444	0.7488	0.7532
Guyana	0.4605	0.4661	0.4696	0.4715	0.4735	0.4762	0.4795	0.4832	0.4866	0.4892	0.4917	0.4946	0.4996	0.5066	0.5149	0.5239	0.5335	0.5432	0.5512	0.5582	0.5640	0.5699	0.5751	0.5794	0.5841	0.5885	0.5939	0.6000	0.6058	0.6112	0.6165	0.6243	0.6320	0.6397	0.6472	0.6546
Haiti	0.2545	0.2580	0.2617	0.2661	0.2710	0.2766	0.2820	0.2876	0.2933	0.2986	0.3035	0.3085	0.3127	0.3163	0.3185	0.3218	0.3254	0.3295	0.3341	0.3392	0.3444	0.3494	0.3542	0.3590	0.3629	0.3666	0.3705	0.3748	0.3790	0.3836	0.3871	0.3914	0.3960	0.4012	0.4065	0.4118
Jamaica	0.5301	0.5345	0.5393	0.5447	0.5507	0.5565	0.5622	0.5690	0.5755	0.5821	0.5888	0.5961	0.6032	0.6104	0.6172	0.6240	0.6300	0.6355	0.6405	0.6455	0.6504	0.6554	0.6603	0.6652	0.6699	0.6744	0.6800	0.6855	0.6908	0.6954	0.6998	0.7039	0.7078	0.7116	0.7153	0.7189
Puerto Rico	0.7052	0.7130	0.7197	0.7258	0.7320	0.7380	0.7444	0.7505	0.7567	0.7627	0.7684	0.7742	0.7801	0.7860	0.7919	0.7978	0.8039	0.8097	0.8157	0.8214	0.8265	0.8326	0.8382	0.8433	0.8482	0.8528	0.8571	0.8608	0.8641	0.8670	0.8696	0.8719	0.8740	0.8763	0.8790	0.8820
Saint Lucia	0.4802	0.4929	0.5042	0.5139	0.5212	0.5284	0.5378	0.5466	0.5556	0.5645	0.5736	0.5837	0.5938	0.6030	0.6116	0.6199	0.6293	0.6381	0.6473	0.6562	0.6642	0.6710	0.6772	0.6836	0.6903	0.6962	0.7006	0.7052	0.7099	0.7147	0.7188	0.7240	0.7287	0.7329	0.7369	0.7408
Saint Vincent and the Grenadines	0.4918	0.5023	0.5125	0.5226	0.5329	0.5430	0.5525	0.5609	0.5702	0.5782	0.5855	0.5940	0.6031	0.6113	0.6186	0.6264	0.6340	0.6415	0.6488	0.6561	0.6631	0.6704	0.6776	0.6847	0.6916	0.6979	0.7049	0.7114	0.7176	0.7231	0.7279	0.7321	0.7361	0.7401	0.7437	0.7473
Suriname	0.4876	0.4958	0.5025	0.5082	0.5132	0.5186	0.5242	0.5292	0.5353	0.5416	0.5476	0.5529	0.5581	0.5625	0.5667	0.5710	0.5767	0.5829	0.5891	0.5943	0.5996	0.6058	0.6121	0.6189	0.6262	0.6335	0.6408	0.6482	0.6557	0.6630	0.6703	0.6776	0.6847	0.6914	0.6977	0.7037
Trinidad and Tobago	0.6293	0.6364	0.6430	0.6485	0.6539	0.6596	0.6643	0.6688	0.6731	0.6770	0.6813	0.6863	0.6909	0.6953	0.7002	0.7053	0.7111	0.7173	0.7237	0.7308	0.7385	0.7457	0.7531	0.7615	0.7698	0.7775	0.7860	0.7939	0.8014	0.8068	0.8115	0.8164	0.8209	0.8251	0.8290	0.8327
Virgin Islands, U.S.	0.6970	0.7072	0.7156	0.7225	0.7282	0.7336	0.7384	0.7437	0.7496	0.7559	0.7625	0.7716	0.7810	0.7903	0.7996	0.8088	0.8173	0.8256	0.8331	0.8393	0.8441	0.8490	0.8527	0.8554	0.8577	0.8607	0.8632	0.8663	0.8685	0.8707	0.8735	0.8767	0.8798	0.8823	0.8843	0.8861
Andean Latin America	0.4513	0.4600	0.4683	0.4758	0.4830	0.4899	0.4967	0.5036	0.5099	0.5148	0.5197	0.5247	0.5298	0.5352	0.5415	0.5482	0.5550	0.5622	0.5690	0.5753	0.5815	0.5874	0.5933	0.5992	0.6054	0.6119	0.6190	0.6262	0.6338	0.6406	0.6477	0.6550	0.6622	0.6694	0.6761	0.6824
Bolivia	0.3764	0.3825	0.3884	0.3939	0.3995	0.4050	0.4098	0.4146	0.4193	0.4244	0.4300	0.4363	0.4426	0.4494	0.4565	0.4641	0.4719	0.4798	0.4879	0.4955	0.5028	0.5100	0.5169	0.5237	0.5306	0.5377	0.5451	0.5524	0.5600	0.5673	0.5746	0.5822	0.5898	0.5975	0.6049	0.6120
Ecuador	0.4627	0.4724	0.4811	0.4888	0.4962	0.5034	0.5101	0.5158	0.5222	0.5283	0.5347	0.5411	0.5476	0.5539	0.5602	0.5662	0.5722	0.5783	0.5842	0.5893	0.5944	0.5995	0.6049	0.6102	0.6163	0.6227	0.6295	0.6359	0.6425	0.6485	0.6544	0.6607	0.6671	0.6735	0.6797	0.6852
Peru	0.4702	0.4792	0.4880	0.4959	0.5035	0.5106	0.5181	0.5260	0.5328	0.5371	0.5410	0.5449	0.5488	0.5534	0.5594	0.5663	0.5731	0.5806	0.5875	0.5942	0.6007	0.6066	0.6126	0.6185	0.6246	0.6311	0.6384	0.6461	0.6543	0.6614	0.6690	0.6768	0.6844	0.6918	0.6987	0.7050
Central Latin America	0.4575	0.4682	0.4778	0.4865	0.4949	0.5029	0.5106	0.5180	0.5250	0.5318	0.5387	0.5457	0.5530	0.5601	0.5673	0.5738	0.5802	0.5869	0.5934	0.5996	0.6060	0.6120	0.6176	0.6230	0.6288	0.6349	0.6416	0.6484	0.6550	0.6606	0.6662	0.6720	0.6778	0.6834	0.6887	0.6938
Colombia	0.4681	0.4766	0.4849	0.4929	0.5007	0.5083	0.5160	0.5234	0.5305	0.5374	0.5442	0.5510	0.5578	0.5647	0.5718	0.5791	0.5860	0.5927	0.5988	0.6039	0.6088	0.6136	0.6183	0.6231	0.6282	0.6339	0.6402	0.6471	0.6542	0.6608	0.6673	0.6742	0.6809	0.6874	0.6938	0.6998
Costa Rica	0.5156	0.5205	0.5246	0.5284	0.5325	0.5364	0.5406	0.5447	0.5490	0.5538	0.5587	0.5641	0.5704	0.5770	0.5837	0.5904	0.5973	0.6047	0.6126	0.6210	0.6289	0.6357	0.6422	0.6486	0.6547	0.6608	0.6673	0.6742	0.6807	0.6868	0.6930	0.6992	0.7056	0.7117	0.7176	0.7232
El Salvador	0.3758	0.3824	0.3882	0.3939	0.3995	0.4050	0.4109	0.4168	0.4228	0.4288	0.4353	0.4424	0.4502	0.4585	0.4671	0.4759	0.4843	0.4929	0.5016	0.5102	0.5186	0.5269	0.5349	0.5425	0.5499	0.5572	0.5646	0.5721	0.5791	0.5852	0.5910	0.5969	0.6026	0.6082	0.6135	0.6187
Guatemala	0.3117	0.3167	0.3218	0.3274	0.3333	0.3394	0.3452	0.3510	0.3568	0.3625	0.3680	0.3739	0.3798	0.3859	0.3924	0.3993	0.4063	0.4136	0.4212	0.4289	0.4366	0.4443	0.4521	0.4597	0.4671	0.4745	0.4820	0.4896	0.4969	0.5038	0.5104	0.5170	0.5235	0.5299	0.5363	0.5427
Honduras	0.3092	0.3170	0.3247	0.3321	0.3395	0.3468	0.3535	0.3602	0.3669	0.3736	0.3800	0.3863	0.3930	0.4001	0.4068	0.4140	0.4210	0.4285	0.4362	0.4433	0.4511	0.4590	0.4670	0.4751	0.4834	0.4921	0.5012	0.5104	0.5193	0.5273	0.5350	0.5424	0.5495	0.5563	0.5625	0.5684
Mexico	0.4661	0.4800	0.4921	0.5027	0.5127	0.5223	0.5311	0.5394	0.5473	0.5549	0.5626	0.5705	0.5783	0.5860	0.5936	0.5999	0.6063	0.6130	0.6198	0.6265	0.6335	0.6400	0.6460	0.6519	0.6578	0.6638	0.6704	0.6767	0.6827	0.6875	0.6924	0.6975	0.7028	0.7078	0.7127	0.7176
Aguascalientes	0.4838	0.4985	0.5112	0.5222	0.5326	0.5429	0.5528	0.5620	0.5706	0.5791	0.5867	0.5939	0.6011	0.6082	0.6151	0.6208	0.6268	0.6334	0.6401	0.6467	0.6538	0.6611	0.6684	0.6754	0.6825	0.6896	0.6972	0.7045	0.7113	0.7170	0.7227	0.7285	0.7343	0.7400	0.7455	0.7510
Baja California	0.5383	0.5522	0.5643	0.5748	0.5846	0.5929	0.5992	0.6051	0.6105	0.6160	0.6217	0.6280	0.6343	0.6406	0.6469	0.6519	0.6579	0.6649	0.6721	0.6793	0.6862	0.6931	0.7004	0.7073	0.7141	0.7209	0.7282	0.7352	0.7418	0.7472	0.7528	0.7586	0.7645	0.7702	0.7758	0.7815
Baja California Sur	0.5165	0.5329	0.5473	0.5599	0.5716	0.5837	0.5959	0.6075	0.6186	0.6294	0.6385	0.6459	0.6532	0.6604	0.6676	0.6735	0.6789	0.6839	0.6889	0.6937	0.6991	0.7050	0.7108	0.7165	0.7224	0.7284	0.7351	0.7417	0.7479	0.7531	0.7584	0.7640	0.7698	0.7753	0.7807	0.7862
Campeche	0.4470	0.4607	0.4726	0.4829	0.4926	0.5029	0.5133	0.5229	0.5321	0.5410	0.5492	0.5568	0.5645	0.5720	0.5794	0.5855	0.5913	0.5970	0.6029	0.6085	0.6150	0.6222	0.6295	0.6363	0.6432	0.6499	0.6571	0.6639	0.6702	0.6753	0.6804	0.6856	0.6910	0.6960	0.7010	0.7059
Chiapas	0.3174	0.3315	0.3437	0.3543	0.3643	0.3747	0.3852	0.3952	0.4048	0.4141	0.4231	0.4320	0.4407	0.4490	0.4572	0.4641	0.4711	0.4786	0.4861	0.4934	0.5011	0.5083	0.5149	0.5212	0.5275	0.5336	0.5401	0.5462	0.5518	0.5561	0.5603	0.5647	0.5691	0.5731	0.5771	0.5810
Chihuahua	0.5067	0.5201	0.5319	0.5421	0.5517	0.5603	0.5676	0.5744	0.5807	0.5870	0.5931	0.5992	0.6054	0.6115	0.6176	0.6225	0.6279	0.6343	0.6407	0.6469	0.6533	0.6596	0.6659	0.6721	0.6785	0.6850	0.6922	0.6992	0.7058	0.7112	0.7169	0.7227	0.7286	0.7344	0.7401	0.7457
Coahuila	0.5324	0.5474	0.5606	0.5720	0.5827	0.5928	0.6021	0.6108	0.6191	0.6273	0.6347	0.6416	0.6485	0.6553	0.6621	0.6677	0.6732	0.6791	0.6850	0.6909	0.6973	0.7038	0.7100	0.7161	0.7224	0.7289	0.7360	0.7430	0.7496	0.7552	0.7609	0.7668	0.7729	0.7788	0.7846	0.7904
Colima	0.4874	0.5020	0.5149	0.5261	0.5366	0.5479	0.5594	0.5702	0.5803	0.5902	0.5986	0.6059	0.6132	0.6205	0.6277	0.6336	0.6395	0.6455	0.6516	0.6578	0.6643	0.6702	0.6755	0.6806	0.6860	0.6915	0.6976	0.7036	0.7093	0.7138	0.7185	0.7235	0.7287	0.7337	0.7385	0.7434
Distrito Federal	0.6447	0.6559	0.6656	0.6739	0.6817	0.6882	0.6930	0.6974	0.7014	0.7053	0.7116	0.7205	0.7294	0.7384	0.7474	0.7552	0.7627	0.7701	0.7777	0.7852	0.7925	0.7980	0.8023	0.8068	0.8118	0.8172	0.8234	0.8298	0.8362	0.8417	0.8476	0.8539	0.8604	0.8669	0.8734	0.8799
Durango	0.4585	0.4729	0.4854	0.4962	0.5064	0.5168	0.5268	0.5364	0.5453	0.5542	0.5623	0.5700	0.5777	0.5853	0.5928	0.5989	0.6047	0.6101	0.6156	0.6210	0.6276	0.6350	0.6422	0.6490	0.6558	0.6625	0.6697	0.6766	0.6829	0.6880	0.6933	0.6987	0.7042	0.7095	0.7147	0.7199
Guanajuato	0.3999	0.4149	0.4281	0.4397	0.4506	0.4616	0.4726	0.4831	0.4930	0.5025	0.5114	0.5199	0.5282	0.5361	0.5440	0.5505	0.5568	0.5631	0.5692	0.5753	0.5822	0.5897	0.5970	0.6036	0.6101	0.6165	0.6231	0.6293	0.6351	0.6396	0.6441	0.6488	0.6535	0.6579	0.6622	0.6665
Guerrero	0.3573	0.3706	0.3823	0.3924	0.4021	0.4119	0.4216	0.4308	0.4395	0.4479	0.4554	0.4624	0.4692	0.4758	0.4822	0.4872	0.4930	0.4996	0.5061	0.5123	0.5195	0.5272	0.5345	0.5417	0.5488	0.5559	0.5633	0.5702	0.5764	0.5811	0.5859	0.5907	0.5955	0.5998	0.6041	0.6082
Hidalgo	0.4023	0.4176	0.4310	0.4428	0.4539	0.4655	0.4770	0.4878	0.4980	0.5078	0.5164	0.5242	0.5320	0.5395	0.5469	0.5530	0.5590	0.5651	0.5714	0.5776	0.5845	0.5910	0.5966	0.6021	0.6077	0.6134	0.6195	0.6254	0.6308	0.6351	0.6394	0.6439	0.6484	0.6527	0.6568	0.6609
Jalisco	0.4703	0.4847	0.4973	0.5082	0.5185	0.5283	0.5373	0.5458	0.5537	0.5615	0.5693	0.5774	0.5855	0.5935	0.6014	0.6079	0.6147	0.6218	0.6290	0.6361	0.6434	0.6504	0.6572	0.6636	0.6702	0.6766	0.6835	0.6902	0.6965	0.7015	0.7067	0.7121	0.7175	0.7228	0.7280	0.7331
MÃ©xico	0.4501	0.4678	0.4832	0.4965	0.5090	0.5193	0.5274	0.5349	0.5419	0.5487	0.5564	0.5651	0.5739	0.5825	0.5910	0.5981	0.6055	0.6134	0.6214	0.6292	0.6366	0.6422	0.6472	0.6520	0.6570	0.6622	0.6680	0.6737	0.6791	0.6832	0.6876	0.6922	0.6970	0.7016	0.7061	0.7105
MichoacÃ¡n de Ocampo	0.3912	0.4048	0.4166	0.4269	0.4367	0.4473	0.4583	0.4687	0.4785	0.4880	0.4968	0.5049	0.5130	0.5209	0.5286	0.5350	0.5413	0.5477	0.5542	0.5607	0.5680	0.5753	0.5820	0.5881	0.5943	0.6003	0.6065	0.6125	0.6180	0.6222	0.6265	0.6309	0.6354	0.6397	0.6439	0.6480
Morelos	0.4707	0.4850	0.4976	0.5086	0.5189	0.5294	0.5397	0.5493	0.5584	0.5672	0.5748	0.5812	0.5878	0.5942	0.6006	0.6057	0.6110	0.6168	0.6225	0.6281	0.6343	0.6402	0.6458	0.6512	0.6569	0.6626	0.6690	0.6753	0.6811	0.6857	0.6905	0.6956	0.7007	0.7056	0.7105	0.7152
Nayarit	0.4403	0.4548	0.4674	0.4784	0.4888	0.4993	0.5095	0.5193	0.5285	0.5376	0.5460	0.5539	0.5618	0.5695	0.5772	0.5835	0.5893	0.5947	0.6002	0.6056	0.6121	0.6187	0.6245	0.6301	0.6358	0.6417	0.6481	0.6542	0.6600	0.6645	0.6692	0.6741	0.6791	0.6839	0.6886	0.6932
Nuevo LeÃ³n	0.5741	0.5898	0.6036	0.6156	0.6270	0.6367	0.6446	0.6519	0.6588	0.6655	0.6724	0.6796	0.6869	0.6942	0.7014	0.7073	0.7129	0.7183	0.7236	0.7288	0.7347	0.7410	0.7472	0.7532	0.7594	0.7657	0.7727	0.7795	0.7861	0.7916	0.7974	0.8034	0.8096	0.8157	0.8218	0.8278
Oaxaca	0.3500	0.3620	0.3725	0.3815	0.3900	0.3995	0.4095	0.4189	0.4279	0.4367	0.4449	0.4528	0.4607	0.4683	0.4758	0.4819	0.4885	0.4956	0.5029	0.5102	0.5182	0.5256	0.5322	0.5384	0.5445	0.5506	0.5571	0.5632	0.5688	0.5731	0.5774	0.5819	0.5863	0.5905	0.5945	0.5985
Puebla	0.4101	0.4239	0.4358	0.4461	0.4558	0.4655	0.4749	0.4836	0.4918	0.4999	0.5079	0.5161	0.5243	0.5323	0.5401	0.5466	0.5536	0.5614	0.5691	0.5767	0.5845	0.5917	0.5983	0.6045	0.6109	0.6173	0.6241	0.6307	0.6367	0.6415	0.6464	0.6514	0.6565	0.6613	0.6660	0.6706
QuerÃ©taro	0.4258	0.4435	0.4589	0.4724	0.4851	0.4984	0.5119	0.5244	0.5362	0.5477	0.5579	0.5672	0.5764	0.5854	0.5941	0.6015	0.6089	0.6167	0.6245	0.6322	0.6400	0.6473	0.6541	0.6605	0.6669	0.6730	0.6796	0.6858	0.6916	0.6960	0.7006	0.7052	0.7098	0.7142	0.7185	0.7227
Quintana Roo	0.4308	0.4538	0.4733	0.4900	0.5052	0.5198	0.5337	0.5468	0.5592	0.5712	0.5813	0.5899	0.5982	0.6062	0.6141	0.6207	0.6276	0.6350	0.6423	0.6494	0.6565	0.6629	0.6689	0.6746	0.6805	0.6864	0.6929	0.6991	0.7048	0.7094	0.7141	0.7189	0.7236	0.7282	0.7326	0.7369
San Luis PotosÃ-	0.4188	0.4331	0.4456	0.4566	0.4671	0.4781	0.4895	0.5003	0.5104	0.5203	0.5290	0.5367	0.5444	0.5518	0.5591	0.5650	0.5712	0.5778	0.5843	0.5906	0.5977	0.6063	0.6154	0.6236	0.6310	0.6380	0.6451	0.6516	0.6575	0.6619	0.6664	0.6710	0.6755	0.6799	0.6843	0.6885
Sinaloa	0.4717	0.4873	0.5011	0.5131	0.5245	0.5350	0.5445	0.5534	0.5618	0.5700	0.5778	0.5853	0.5928	0.6001	0.6074	0.6133	0.6192	0.6252	0.6311	0.6369	0.6432	0.6497	0.6561	0.6620	0.6681	0.6741	0.6807	0.6870	0.6929	0.6975	0.7023	0.7072	0.7123	0.7171	0.7219	0.7265
Sonora	0.5285	0.5428	0.5553	0.5662	0.5764	0.5860	0.5945	0.6025	0.6101	0.6177	0.6248	0.6317	0.6386	0.6455	0.6523	0.6578	0.6632	0.6685	0.6737	0.6786	0.6843	0.6905	0.6967	0.7029	0.7092	0.7156	0.7227	0.7296	0.7362	0.7416	0.7472	0.7530	0.7589	0.7647	0.7704	0.7761
Tabasco	0.4356	0.4535	0.4692	0.4829	0.4959	0.5095	0.5235	0.5368	0.5493	0.5614	0.5724	0.5825	0.5924	0.6019	0.6113	0.6193	0.6271	0.6349	0.6428	0.6507	0.6586	0.6654	0.6713	0.6768	0.6825	0.6882	0.6943	0.7003	0.7059	0.7103	0.7149	0.7196	0.7244	0.7289	0.7333	0.7376
Tamaulipas	0.5234	0.5380	0.5508	0.5620	0.5725	0.5824	0.5914	0.5998	0.6078	0.6157	0.6227	0.6292	0.6357	0.6421	0.6486	0.6537	0.6589	0.6644	0.6700	0.6755	0.6817	0.6879	0.6937	0.6994	0.7053	0.7113	0.7180	0.7246	0.7309	0.7362	0.7416	0.7473	0.7531	0.7588	0.7644	0.7699
Tlaxcala	0.4191	0.4335	0.4461	0.4570	0.4673	0.4788	0.4910	0.5025	0.5132	0.5236	0.5327	0.5408	0.5488	0.5567	0.5644	0.5707	0.5772	0.5844	0.5916	0.5988	0.6061	0.6125	0.6182	0.6236	0.6293	0.6350	0.6414	0.6475	0.6532	0.6576	0.6623	0.6671	0.6720	0.6766	0.6812	0.6856
Veracruz de Ignacio de la Llave	0.4391	0.4526	0.4644	0.4746	0.4844	0.4936	0.5019	0.5099	0.5173	0.5247	0.5321	0.5398	0.5474	0.5549	0.5624	0.5685	0.5748	0.5816	0.5886	0.5955	0.6025	0.6087	0.6143	0.6195	0.6249	0.6303	0.6362	0.6420	0.6474	0.6516	0.6559	0.6605	0.6652	0.6696	0.6740	0.6782
YucatÃ¡n	0.4533	0.4661	0.4773	0.4869	0.4960	0.5048	0.5130	0.5207	0.5279	0.5351	0.5428	0.5511	0.5595	0.5678	0.5759	0.5827	0.5898	0.5974	0.6051	0.6128	0.6202	0.6265	0.6321	0.6376	0.6433	0.6492	0.6557	0.6620	0.6680	0.6728	0.6777	0.6830	0.6883	0.6934	0.6985	0.7035
Zacatecas	0.4020	0.4163	0.4287	0.4395	0.4496	0.4617	0.4751	0.4878	0.4999	0.5115	0.5211	0.5290	0.5368	0.5443	0.5518	0.5579	0.5633	0.5682	0.5730	0.5778	0.5841	0.5907	0.5965	0.6021	0.6079	0.6138	0.6201	0.6263	0.6321	0.6367	0.6414	0.6463	0.6513	0.6560	0.6607	0.6652
Nicaragua	0.3450	0.3499	0.3547	0.3606	0.3666	0.3726	0.3786	0.3848	0.3896	0.3945	0.3993	0.4043	0.4092	0.4145	0.4202	0.4265	0.4335	0.4409	0.4485	0.4567	0.4649	0.4729	0.4802	0.4870	0.4940	0.5007	0.5074	0.5141	0.5206	0.5260	0.5315	0.5376	0.5439	0.5503	0.5567	0.5631
Panama	0.5293	0.5382	0.5467	0.5541	0.5611	0.5680	0.5749	0.5813	0.5857	0.5898	0.5940	0.5990	0.6045	0.6100	0.6152	0.6198	0.6250	0.6307	0.6370	0.6432	0.6490	0.6544	0.6595	0.6648	0.6701	0.6757	0.6817	0.6883	0.6952	0.7017	0.7084	0.7157	0.7236	0.7316	0.7393	0.7467
Venezuela	0.5327	0.5400	0.5468	0.5527	0.5583	0.5634	0.5687	0.5743	0.5802	0.5852	0.5909	0.5974	0.6043	0.6108	0.6166	0.6224	0.6278	0.6333	0.6384	0.6426	0.6472	0.6518	0.6553	0.6580	0.6627	0.6688	0.6759	0.6836	0.6915	0.6977	0.7035	0.7095	0.7155	0.7210	0.7254	0.7283
Tropical Latin America	0.4223	0.4306	0.4389	0.4468	0.4549	0.4633	0.4723	0.4813	0.4899	0.4982	0.5055	0.5127	0.5193	0.5257	0.5322	0.5389	0.5457	0.5522	0.5583	0.5640	0.5694	0.5757	0.5818	0.5879	0.5942	0.6005	0.6070	0.6137	0.6206	0.6266	0.6328	0.6392	0.6449	0.6506	0.6565	0.6611
Brazil	0.4225	0.4309	0.4392	0.4471	0.4553	0.4638	0.4729	0.4819	0.4906	0.4989	0.5063	0.5135	0.5201	0.5266	0.5331	0.5397	0.5465	0.5530	0.5590	0.5647	0.5700	0.5764	0.5825	0.5886	0.5949	0.6013	0.6077	0.6145	0.6214	0.6274	0.6336	0.6399	0.6457	0.6513	0.6571	0.6616
Acre	0.2930	0.3030	0.3132	0.3231	0.3332	0.3434	0.3556	0.3674	0.3785	0.3890	0.3983	0.4057	0.4123	0.4186	0.4248	0.4310	0.4381	0.4449	0.4512	0.4573	0.4636	0.4682	0.4690	0.4756	0.4804	0.4858	0.4969	0.5052	0.5062	0.5172	0.5258	0.5286	0.5398	0.5448	0.5512	0.5557
Alagoas	0.2781	0.2867	0.2953	0.3035	0.3120	0.3209	0.3309	0.3408	0.3503	0.3593	0.3674	0.3740	0.3800	0.3859	0.3917	0.3977	0.4044	0.4107	0.4165	0.4219	0.4278	0.4289	0.4370	0.4437	0.4530	0.4578	0.4650	0.4704	0.4768	0.4835	0.4899	0.4961	0.5025	0.5073	0.5076	0.5116
AmapÃ¡	0.3376	0.3492	0.3612	0.3730	0.3849	0.3970	0.4115	0.4256	0.4386	0.4508	0.4616	0.4718	0.4808	0.4894	0.4977	0.5060	0.5134	0.5203	0.5268	0.5330	0.5285	0.5352	0.5462	0.5509	0.5655	0.5736	0.5774	0.5869	0.5935	0.6044	0.6083	0.6172	0.6255	0.6289	0.6364	0.6416
Amazonas	0.3448	0.3559	0.3672	0.3783	0.3897	0.4011	0.4157	0.4298	0.4429	0.4551	0.4660	0.4724	0.4780	0.4833	0.4884	0.4934	0.5014	0.5090	0.5162	0.5231	0.5381	0.5419	0.5479	0.5558	0.5633	0.5695	0.5735	0.5832	0.5914	0.5971	0.6059	0.6129	0.6187	0.6242	0.6304	0.6355
Bahia	0.3123	0.3205	0.3289	0.3369	0.3453	0.3541	0.3659	0.3775	0.3883	0.3986	0.4078	0.4151	0.4217	0.4282	0.4347	0.4413	0.4490	0.4563	0.4631	0.4695	0.4808	0.4849	0.4894	0.4944	0.5011	0.5076	0.5143	0.5196	0.5261	0.5315	0.5378	0.5435	0.5493	0.5552	0.5550	0.5590
CearÃ¡	0.3161	0.3232	0.3303	0.3372	0.3444	0.3520	0.3608	0.3696	0.3781	0.3862	0.3935	0.4008	0.4074	0.4139	0.4204	0.4270	0.4339	0.4405	0.4466	0.4522	0.4649	0.4663	0.4737	0.4805	0.4875	0.4928	0.4993	0.5054	0.5125	0.5182	0.5247	0.5313	0.5371	0.5428	0.5441	0.5483
Distrito Federal	0.5742	0.5837	0.5931	0.6022	0.6115	0.6212	0.6324	0.6435	0.6541	0.6643	0.6735	0.6811	0.6881	0.6949	0.7018	0.7089	0.7160	0.7229	0.7294	0.7356	0.7302	0.7350	0.7432	0.7503	0.7592	0.7675	0.7754	0.7849	0.7949	0.8025	0.8102	0.8210	0.8288	0.8352	0.8486	0.8546
EspÃ-rito Santo	0.4432	0.4525	0.4617	0.4704	0.4794	0.4886	0.4979	0.5072	0.5160	0.5244	0.5320	0.5398	0.5470	0.5540	0.5610	0.5682	0.5750	0.5815	0.5875	0.5931	0.5948	0.6010	0.6081	0.6154	0.6218	0.6292	0.6348	0.6410	0.6476	0.6531	0.6586	0.6646	0.6704	0.6747	0.6809	0.6851
GoiÃ¡s	0.3900	0.4024	0.4144	0.4257	0.4368	0.4479	0.4570	0.4661	0.4746	0.4829	0.4903	0.4972	0.5036	0.5099	0.5163	0.5228	0.5292	0.5352	0.5407	0.5458	0.5514	0.5560	0.5603	0.5667	0.5730	0.5790	0.5859	0.5926	0.5985	0.6033	0.6089	0.6144	0.6179	0.6223	0.6292	0.6330
MaranhÃ£o	0.2667	0.2716	0.2769	0.2820	0.2877	0.2940	0.3051	0.3161	0.3265	0.3365	0.3454	0.3533	0.3605	0.3674	0.3742	0.3810	0.3890	0.3966	0.4037	0.4104	0.4359	0.4355	0.4364	0.4376	0.4447	0.4498	0.4558	0.4618	0.4688	0.4756	0.4828	0.4888	0.4959	0.5014	0.4980	0.5020
Mato Grosso	0.3947	0.4063	0.4179	0.4289	0.4400	0.4512	0.4613	0.4712	0.4807	0.4899	0.4980	0.5058	0.5129	0.5198	0.5266	0.5334	0.5410	0.5483	0.5551	0.5615	0.5684	0.5742	0.5793	0.5839	0.5869	0.5923	0.6008	0.6092	0.6141	0.6206	0.6260	0.6307	0.6362	0.6397	0.6469	0.6509
Mato Grosso do Sul	0.4227	0.4327	0.4425	0.4517	0.4612	0.4708	0.4794	0.4881	0.4963	0.5043	0.5113	0.5182	0.5244	0.5304	0.5366	0.5428	0.5496	0.5560	0.5619	0.5675	0.5739	0.5779	0.5831	0.5899	0.5926	0.5997	0.6076	0.6150	0.6192	0.6258	0.6322	0.6369	0.6427	0.6485	0.6560	0.6601
Minas Gerais	0.4267	0.4352	0.4437	0.4517	0.4599	0.4685	0.4769	0.4853	0.4933	0.5010	0.5078	0.5149	0.5213	0.5276	0.5340	0.5406	0.5475	0.5540	0.5601	0.5658	0.5734	0.5776	0.5848	0.5899	0.5970	0.6031	0.6097	0.6165	0.6234	0.6297	0.6350	0.6410	0.6464	0.6520	0.6551	0.6595
ParÃ¡	0.3317	0.3398	0.3481	0.3562	0.3647	0.3735	0.3846	0.3955	0.4059	0.4157	0.4243	0.4318	0.4385	0.4449	0.4513	0.4578	0.4647	0.4712	0.4772	0.4830	0.5059	0.5042	0.5091	0.5110	0.5190	0.5253	0.5287	0.5353	0.5436	0.5527	0.5604	0.5679	0.5758	0.5814	0.5819	0.5868
ParaÃ-ba	0.3029	0.3109	0.3188	0.3264	0.3342	0.3424	0.3521	0.3618	0.3710	0.3799	0.3878	0.3948	0.4011	0.4074	0.4136	0.4201	0.4276	0.4349	0.4416	0.4478	0.4615	0.4592	0.4648	0.4701	0.4773	0.4817	0.4893	0.4953	0.5004	0.5064	0.5127	0.5190	0.5253	0.5300	0.5311	0.5349
ParanÃ¡	0.4400	0.4500	0.4599	0.4693	0.4787	0.4883	0.4965	0.5046	0.5123	0.5198	0.5265	0.5333	0.5395	0.5457	0.5518	0.5581	0.5650	0.5715	0.5775	0.5832	0.5853	0.5928	0.5979	0.6058	0.6106	0.6163	0.6231	0.6306	0.6365	0.6419	0.6466	0.6531	0.6582	0.6628	0.6704	0.6746
Pernambuco	0.3480	0.3565	0.3650	0.3730	0.3814	0.3901	0.3997	0.4093	0.4184	0.4272	0.4350	0.4423	0.4490	0.4555	0.4621	0.4688	0.4755	0.4818	0.4875	0.4929	0.4974	0.5005	0.5083	0.5152	0.5218	0.5272	0.5339	0.5405	0.5470	0.5530	0.5604	0.5660	0.5709	0.5760	0.5806	0.5848
Piaui	0.2732	0.2807	0.2883	0.2955	0.3030	0.3108	0.3210	0.3312	0.3407	0.3499	0.3581	0.3650	0.3713	0.3775	0.3837	0.3900	0.3972	0.4040	0.4103	0.4162	0.4231	0.4267	0.4338	0.4402	0.4461	0.4501	0.4556	0.4625	0.4694	0.4749	0.4810	0.4862	0.4923	0.4978	0.4988	0.5026
Rio de Janeiro	0.5202	0.5285	0.5366	0.5442	0.5520	0.5602	0.5680	0.5759	0.5833	0.5906	0.5969	0.6033	0.6091	0.6149	0.6207	0.6268	0.6331	0.6390	0.6444	0.6494	0.6485	0.6573	0.6646	0.6699	0.6767	0.6850	0.6911	0.6976	0.7059	0.7112	0.7180	0.7245	0.7302	0.7362	0.7477	0.7529
Rio Grande do Norte	0.3393	0.3472	0.3550	0.3626	0.3706	0.3789	0.3883	0.3977	0.4067	0.4153	0.4230	0.4308	0.4379	0.4448	0.4518	0.4589	0.4660	0.4727	0.4789	0.4847	0.4898	0.4957	0.5011	0.5075	0.5151	0.5198	0.5285	0.5342	0.5398	0.5455	0.5522	0.5584	0.5646	0.5699	0.5709	0.5749
Rio Grande do Sul	0.5034	0.5100	0.5164	0.5225	0.5289	0.5357	0.5430	0.5504	0.5574	0.5642	0.5702	0.5769	0.5830	0.5891	0.5953	0.6017	0.6081	0.6141	0.6197	0.6250	0.6256	0.6344	0.6409	0.6484	0.6528	0.6613	0.6683	0.6771	0.6841	0.6899	0.6963	0.7022	0.7080	0.7130	0.7212	0.7262
RondÃ´nia	0.3377	0.3512	0.3646	0.3771	0.3895	0.4017	0.4119	0.4219	0.4313	0.4402	0.4481	0.4555	0.4621	0.4685	0.4748	0.4811	0.4887	0.4959	0.5027	0.5091	0.5110	0.5231	0.5261	0.5328	0.5389	0.5473	0.5584	0.5677	0.5680	0.5743	0.5806	0.5843	0.5917	0.5960	0.5997	0.6040
Roraima	0.3680	0.3800	0.3920	0.4035	0.4151	0.4268	0.4364	0.4460	0.4551	0.4638	0.4715	0.4813	0.4881	0.4947	0.5013	0.5079	0.5140	0.5196	0.5249	0.5299	0.5189	0.5300	0.5445	0.5410	0.5549	0.5653	0.5732	0.5813	0.5874	0.5979	0.6047	0.6125	0.6150	0.6206	0.6329	0.6381
Santa Catarina	0.4770	0.4857	0.4942	0.5023	0.5106	0.5192	0.5272	0.5352	0.5428	0.5502	0.5568	0.5641	0.5707	0.5773	0.5839	0.5906	0.5975	0.6041	0.6102	0.6159	0.6185	0.6281	0.6345	0.6417	0.6464	0.6535	0.6590	0.6664	0.6723	0.6789	0.6847	0.6908	0.6960	0.7016	0.7103	0.7149
SÃ£o Paulo	0.5197	0.5274	0.5351	0.5423	0.5499	0.5578	0.5664	0.5751	0.5834	0.5914	0.5985	0.6060	0.6128	0.6196	0.6265	0.6335	0.6400	0.6461	0.6518	0.6571	0.6561	0.6657	0.6716	0.6785	0.6841	0.6908	0.6974	0.7042	0.7116	0.7173	0.7233	0.7302	0.7356	0.7421	0.7518	0.7567
Sergipe	0.3167	0.3270	0.3372	0.3471	0.3572	0.3675	0.3780	0.3885	0.3985	0.4080	0.4166	0.4241	0.4309	0.4375	0.4442	0.4509	0.4578	0.4644	0.4706	0.4763	0.4799	0.4845	0.4942	0.5002	0.5080	0.5122	0.5175	0.5250	0.5313	0.5383	0.5454	0.5510	0.5574	0.5624	0.5666	0.5709
Tocantins	0.3736	0.3802	0.3868	0.3932	0.3999	0.4071	0.4129	0.4188	0.4245	0.4299	0.4345	0.4425	0.4497	0.4567	0.4635	0.4705	0.4784	0.4860	0.4931	0.4999	0.5044	0.5085	0.5168	0.5241	0.5323	0.5381	0.5445	0.5534	0.5600	0.5673	0.5746	0.5809	0.5881	0.5944	0.5960	0.6006
Paraguay	0.4137	0.4214	0.4283	0.4344	0.4404	0.4464	0.4522	0.4582	0.4646	0.4712	0.4779	0.4842	0.4903	0.4966	0.5032	0.5102	0.5176	0.5254	0.5330	0.5404	0.5470	0.5531	0.5589	0.5649	0.5708	0.5762	0.5819	0.5880	0.5946	0.6004	0.6077	0.6150	0.6215	0.6293	0.6367	0.6436

Appendix Table 5. Socio-Demographic Index values for all estimated GBD 2015 locations, 1980–2015

Location	1980	1981	1982	1983	1984	1985	1986	1987	1988	1989	1990	1991	1992	1993	1994	1995	1996	1997	1998	1999	2000	2001	2002	2003	2004	2005	2006	2007	2008	2009	2010	2011	2012	2013	2014	2015
North Africa and Middle East	0.3300	0.3378	0.3462	0.3550	0.3641	0.3737	0.3829	0.3924	0.4025	0.4124	0.4225	0.4325	0.4421	0.4514	0.4602	0.4689	0.4779	0.4868	0.4956	0.5040	0.5121	0.5195	0.5268	0.5337	0.5408	0.5479	0.5550	0.5620	0.5685	0.5745	0.5803	0.5858	0.5908	0.5956	0.6001	0.6045
North Africa and Middle East	0.3300	0.3378	0.3462	0.3550	0.3641	0.3737	0.3829	0.3924	0.4025	0.4124	0.4225	0.4325	0.4421	0.4514	0.4602	0.4689	0.4779	0.4868	0.4956	0.5040	0.5121	0.5195	0.5268	0.5337	0.5408	0.5479	0.5550	0.5620	0.5685	0.5745	0.5803	0.5858	0.5908	0.5956	0.6001	0.6045
Afghanistan	0.1291	0.1314	0.1336	0.1355	0.1373	0.1392	0.1407	0.1413	0.1418	0.1427	0.1440	0.1473	0.1514	0.1536	0.1530	0.1526	0.1499	0.1464	0.1432	0.1415	0.1416	0.1439	0.1524	0.1625	0.1716	0.1812	0.1892	0.1984	0.2072	0.2186	0.2303	0.2428	0.2560	0.2682	0.2791	0.2888
Algeria	0.3173	0.3301	0.3435	0.3563	0.3694	0.3823	0.3947	0.4067	0.4188	0.4311	0.4434	0.4558	0.4682	0.4805	0.4924	0.5042	0.5142	0.5236	0.5324	0.5404	0.5474	0.5537	0.5592	0.5641	0.5680	0.5714	0.5735	0.5750	0.5762	0.5772	0.5784	0.5800	0.5822	0.5846	0.5872	0.5900
Bahrain	0.5059	0.5172	0.5267	0.5350	0.5429	0.5498	0.5568	0.5651	0.5750	0.5856	0.5969	0.6062	0.6165	0.6278	0.6392	0.6503	0.6585	0.6664	0.6735	0.6792	0.6849	0.6920	0.6981	0.7042	0.7110	0.7187	0.7301	0.7409	0.7501	0.7569	0.7621	0.7642	0.7662	0.7687	0.7721	0.7764
Egypt	0.3470	0.3541	0.3616	0.3694	0.3777	0.3865	0.3961	0.4063	0.4176	0.4292	0.4409	0.4526	0.4643	0.4754	0.4864	0.4970	0.5078	0.5177	0.5269	0.5357	0.5439	0.5517	0.5590	0.5660	0.5726	0.5788	0.5843	0.5895	0.5942	0.5980	0.6012	0.6045	0.6075	0.6107	0.6144	0.6191
Iran	0.3456	0.3485	0.3535	0.3606	0.3698	0.3814	0.3940	0.4088	0.4249	0.4418	0.4600	0.4783	0.4958	0.5133	0.5300	0.5461	0.5617	0.5764	0.5897	0.6020	0.6134	0.6238	0.6343	0.6446	0.6543	0.6636	0.6720	0.6802	0.6868	0.6923	0.6977	0.7029	0.7066	0.7095	0.7126	0.7154
Iraq	0.3234	0.3327	0.3420	0.3507	0.3589	0.3664	0.3726	0.3792	0.3857	0.3921	0.3997	0.4041	0.4093	0.4162	0.4227	0.4282	0.4345	0.4424	0.4544	0.4676	0.4800	0.4913	0.5002	0.5047	0.5116	0.5172	0.5223	0.5263	0.5296	0.5331	0.5376	0.5438	0.5517	0.5606	0.5685	0.5756
Jordan	0.3625	0.3818	0.4007	0.4194	0.4374	0.4531	0.4633	0.4727	0.4814	0.4891	0.4967	0.5130	0.5287	0.5426	0.5549	0.5657	0.5714	0.5770	0.5826	0.5887	0.5952	0.6021	0.6090	0.6157	0.6228	0.6302	0.6379	0.6456	0.6535	0.6606	0.6669	0.6728	0.6784	0.6839	0.6894	0.6949
Kuwait	0.5264	0.5424	0.5599	0.5785	0.5979	0.6181	0.6346	0.6524	0.6680	0.6820	0.6911	0.6972	0.7048	0.7121	0.7173	0.7216	0.7260	0.7333	0.7438	0.7548	0.7652	0.7710	0.7751	0.7793	0.7840	0.7890	0.7973	0.8066	0.8165	0.8264	0.8359	0.8420	0.8479	0.8534	0.8582	0.8624
Lebanon	0.5012	0.5084	0.5126	0.5184	0.5278	0.5394	0.5494	0.5596	0.5656	0.5677	0.5698	0.5763	0.5829	0.5894	0.5959	0.6026	0.6093	0.6166	0.6249	0.6335	0.6423	0.6503	0.6593	0.6686	0.6781	0.6870	0.6941	0.7005	0.7067	0.7135	0.7202	0.7277	0.7353	0.7424	0.7489	0.7547
Libya	0.3584	0.3715	0.3843	0.3958	0.4069	0.4182	0.4294	0.4404	0.4509	0.4623	0.4747	0.4879	0.5007	0.5140	0.5259	0.5358	0.5452	0.5537	0.5616	0.5688	0.5756	0.5817	0.5873	0.5935	0.5997	0.6068	0.6138	0.6211	0.6282	0.6347	0.6413	0.6399	0.6429	0.6444	0.6439	0.6430
Morocco	0.2522	0.2591	0.2669	0.2749	0.2831	0.2916	0.3005	0.3088	0.3180	0.3265	0.3347	0.3434	0.3512	0.3587	0.3669	0.3743	0.3830	0.3908	0.3986	0.4058	0.4123	0.4188	0.4248	0.4307	0.4362	0.4411	0.4468	0.4521	0.4574	0.4626	0.4677	0.4734	0.4789	0.4846	0.4902	0.4959
Palestine	0.3528	0.3610	0.3696	0.3795	0.3884	0.3959	0.4023	0.4075	0.4123	0.4173	0.4229	0.4298	0.4381	0.4475	0.4586	0.4713	0.4854	0.5008	0.5156	0.5294	0.5410	0.5481	0.5511	0.5519	0.5518	0.5512	0.5503	0.5490	0.5476	0.5466	0.5471	0.5494	0.5531	0.5575	0.5622	0.5670
Oman	0.2905	0.2932	0.2995	0.3084	0.3186	0.3306	0.3421	0.3555	0.3716	0.3893	0.4089	0.4336	0.4575	0.4798	0.5005	0.5201	0.5386	0.5576	0.5757	0.5930	0.6098	0.6219	0.6332	0.6430	0.6514	0.6583	0.6663	0.6720	0.6771	0.6812	0.6863	0.6953	0.7049	0.7142	0.7226	0.7301
Qatar	0.4963	0.5107	0.5244	0.5375	0.5503	0.5630	0.5742	0.5862	0.5977	0.6078	0.6162	0.6283	0.6375	0.6453	0.6512	0.6571	0.6606	0.6668	0.6728	0.6789	0.6850	0.6973	0.7081	0.7175	0.7258	0.7330	0.7434	0.7528	0.7615	0.7692	0.7760	0.7820	0.7879	0.7937	0.7992	0.8045
Saudi Arabia	0.4005	0.4179	0.4355	0.4522	0.4684	0.4832	0.4909	0.4985	0.5065	0.5149	0.5245	0.5333	0.5426	0.5524	0.5624	0.5726	0.5883	0.6034	0.6176	0.6308	0.6433	0.6530	0.6623	0.6716	0.6809	0.6901	0.6991	0.7075	0.7158	0.7227	0.7299	0.7369	0.7435	0.7495	0.7547	0.7593
'Asir	0.3632	0.3824	0.4015	0.4189	0.4355	0.4507	0.4588	0.4670	0.4750	0.4833	0.4929	0.5017	0.5112	0.5208	0.5309	0.5413	0.5581	0.5740	0.5885	0.6017	0.6139	0.6239	0.6333	0.6426	0.6517	0.6605	0.6694	0.6777	0.6862	0.6938	0.7021	0.7118	0.7210	0.7295	0.7342	0.7383
Bahah	0.3956	0.4101	0.4240	0.4366	0.4486	0.4595	0.4654	0.4712	0.4770	0.4828	0.4899	0.4968	0.5047	0.5127	0.5211	0.5298	0.5435	0.5566	0.5683	0.5790	0.5891	0.5977	0.6058	0.6142	0.6227	0.6310	0.6397	0.6480	0.6567	0.6644	0.6729	0.6828	0.6923	0.7011	0.7061	0.7104
Eastern Province	0.4236	0.4407	0.4579	0.4743	0.4901	0.5045	0.5124	0.5207	0.5293	0.5383	0.5486	0.5578	0.5677	0.5772	0.5868	0.5966	0.6117	0.6264	0.6404	0.6535	0.6659	0.6756	0.6848	0.6939	0.7031	0.7112	0.7193	0.7268	0.7344	0.7411	0.7483	0.7573	0.7660	0.7738	0.7790	0.7837
Ha'il	0.4064	0.4227	0.4388	0.4535	0.4676	0.4806	0.4878	0.4950	0.5022	0.5096	0.5182	0.5265	0.5356	0.5447	0.5541	0.5639	0.5793	0.5938	0.6071	0.6191	0.6304	0.6399	0.6488	0.6577	0.6666	0.6753	0.6841	0.6924	0.7010	0.7086	0.7169	0.7258	0.7343	0.7420	0.7469	0.7512
Jawf	0.2983	0.3293	0.3592	0.3855	0.4098	0.4315	0.4426	0.4540	0.4650	0.4764	0.4893	0.5010	0.5135	0.5258	0.5384	0.5515	0.5721	0.5914	0.6089	0.6248	0.6393	0.6507	0.6614	0.6719	0.6821	0.6927	0.7034	0.7135	0.7238	0.7331	0.7430	0.7549	0.7663	0.7766	0.7825	0.7879
Jizan	0.3172	0.3320	0.3464	0.3590	0.3709	0.3816	0.3870	0.3923	0.3974	0.4027	0.4092	0.4155	0.4227	0.4304	0.4387	0.4473	0.4613	0.4746	0.4865	0.4971	0.5072	0.5154	0.5232	0.5310	0.5391	0.5475	0.5562	0.5643	0.5727	0.5797	0.5873	0.5969	0.6062	0.6147	0.6190	0.6228
Madinah	0.3047	0.3317	0.3586	0.3832	0.4062	0.4269	0.4374	0.4483	0.4591	0.4702	0.4827	0.4940	0.5059	0.5176	0.5296	0.5419	0.5610	0.5791	0.5957	0.6110	0.6252	0.6362	0.6467	0.6571	0.6673	0.6763	0.6852	0.6933	0.7015	0.7086	0.7162	0.7246	0.7326	0.7398	0.7452	0.7500
Makkah	0.4182	0.4341	0.4500	0.4651	0.4796	0.4929	0.4999	0.5071	0.5146	0.5225	0.5317	0.5403	0.5496	0.5588	0.5683	0.5780	0.5928	0.6071	0.6205	0.6329	0.6446	0.6541	0.6630	0.6720	0.6811	0.6884	0.6958	0.7025	0.7095	0.7153	0.7217	0.7286	0.7353	0.7414	0.7468	0.7518
Najran	0.2531	0.2835	0.3118	0.3362	0.3581	0.3770	0.3868	0.3968	0.4065	0.4162	0.4271	0.4358	0.4452	0.4547	0.4648	0.4753	0.4938	0.5114	0.5276	0.5422	0.5556	0.5657	0.5752	0.5844	0.5933	0.6019	0.6106	0.6186	0.6266	0.6336	0.6413	0.6509	0.6601	0.6686	0.6732	0.6771
Northern Borders	0.4254	0.4370	0.4491	0.4609	0.4724	0.4828	0.4865	0.4909	0.4958	0.5014	0.5083	0.5144	0.5212	0.5301	0.5399	0.5498	0.5655	0.5808	0.5952	0.6086	0.6214	0.6311	0.6403	0.6494	0.6586	0.6748	0.6922	0.7074	0.7187	0.7235	0.7246	0.7215	0.7177	0.7128	0.7183	0.7233
Qassim	0.3603	0.3804	0.4016	0.4221	0.4422	0.4613	0.4727	0.4846	0.4963	0.5084	0.5212	0.5320	0.5433	0.5543	0.5656	0.5772	0.5951	0.6121	0.6278	0.6423	0.6558	0.6664	0.6765	0.6863	0.6960	0.7051	0.7130	0.7203	0.7274	0.7349	0.7429	0.7517	0.7601	0.7679	0.7727	0.7771
Riyadh	0.5005	0.5099	0.5209	0.5331	0.5457	0.5578	0.5632	0.5672	0.5723	0.5781	0.5854	0.5918	0.5988	0.6081	0.6182	0.6281	0.6422	0.6562	0.6698	0.6829	0.6955	0.7051	0.7143	0.7235	0.7327	0.7418	0.7502	0.7576	0.7644	0.7692	0.7734	0.7775	0.7815	0.7850	0.7902	0.7944
Tabuk	0.3509	0.3679	0.3870	0.4065	0.4252	0.4420	0.4479	0.4552	0.4631	0.4717	0.4819	0.4893	0.4974	0.5088	0.5215	0.5344	0.5559	0.5766	0.5961	0.6141	0.6306	0.6426	0.6540	0.6649	0.6755	0.7008	0.7254	0.7448	0.7571	0.7609	0.7594	0.7546	0.7490	0.7423	0.7480	0.7531
Sudan	0.2318	0.2363	0.2416	0.2459	0.2493	0.2519	0.2543	0.2565	0.2600	0.2634	0.2667	0.2710	0.2756	0.2803	0.2847	0.2889	0.2921	0.2972	0.3033	0.3105	0.3188	0.3270	0.3351	0.3428	0.3506	0.3582	0.3663	0.3745	0.3823	0.3898	0.3971	0.4046	0.4107	0.4166	0.4224	0.4282
Syria	0.2946	0.3037	0.3130	0.3226	0.3316	0.3410	0.3499	0.3588	0.3692	0.3785	0.3881	0.3980	0.4084	0.4190	0.4293	0.4394	0.4487	0.4575	0.4664	0.4746	0.4830	0.4903	0.4980	0.5054	0.5143	0.5253	0.5376	0.5498	0.5613	0.5716	0.5807	0.5844	0.5836	0.5819	0.5801	0.5790
Tunisia	0.3408	0.3516	0.3622	0.3726	0.3831	0.3936	0.4045	0.4157	0.4269	0.4384	0.4503	0.4629	0.4761	0.4891	0.5017	0.5140	0.5253	0.5359	0.5457	0.5552	0.5642	0.5726	0.5802	0.5875	0.5944	0.6007	0.6072	0.6135	0.6194	0.6248	0.6298	0.6342	0.6388	0.6432	0.6475	0.6515
Turkey	0.4416	0.4494	0.4571	0.4649	0.4730	0.4811	0.4897	0.4988	0.5077	0.5159	0.5242	0.5318	0.5393	0.5468	0.5530	0.5594	0.5665	0.5738	0.5807	0.5869	0.5935	0.5993	0.6053	0.6112	0.6178	0.6247	0.6320	0.6392	0.6460	0.6518	0.6581	0.6650	0.6716	0.6780	0.6842	0.6900
United Arab Emirates	0.5343	0.5449	0.5553	0.5657	0.5765	0.5874	0.5944	0.6019	0.6104	0.6205	0.6324	0.6509	0.6704	0.6902	0.7094	0.7273	0.7391	0.7506	0.7622	0.7725	0.7813	0.7904	0.7982	0.8064	0.8149	0.8237	0.8335	0.8440	0.8538	0.8622	0.8691	0.8724	0.8746	0.8742	0.8742	0.8747
Yemen	0.0960	0.0975	0.0993	0.1022	0.1053	0.1082	0.1103	0.1126	0.1172	0.1236	0.1329	0.1464	0.1599	0.1723	0.1845	0.1964	0.2094	0.2226	0.2354	0.2476	0.2595	0.2713	0.2826	0.2946	0.3067	0.3196	0.3321	0.3441	0.3555	0.3662	0.3766	0.3845	0.3918	0.3989	0.4052	0.4080
South Asia	0.2421	0.2471	0.2522	0.2580	0.2640	0.2704	0.2771	0.2842	0.2922	0.3008	0.3096	0.3179	0.3262	0.3346	0.3431	0.3519	0.3609	0.3697	0.3784	0.3874	0.3963	0.4050	0.4136	0.4224	0.4314	0.4407	0.4506	0.4608	0.4707	0.4806	0.4907	0.5005	0.5099	0.5190	0.5277	0.5362
South Asia	0.2421	0.2471	0.2522	0.2580	0.2640	0.2704	0.2771	0.2842	0.2922	0.3008	0.3096	0.3179	0.3262	0.3346	0.3431	0.3519	0.3609	0.3697	0.3784	0.3874	0.3963	0.4050	0.4136	0.4224	0.4314	0.4407	0.4506	0.4608	0.4707	0.4806	0.4907	0.5005	0.5099	0.5190	0.5277	0.5362
Bangladesh	0.1990	0.2050	0.2116	0.2190	0.2268	0.2349	0.2427	0.2505	0.2582	0.2663	0.2746	0.2825	0.2905	0.2983	0.3060	0.3139	0.3216	0.3292	0.3367	0.3439	0.3512	0.3585	0.3659	0.3734	0.3810	0.3889	0.3971	0.4056	0.4141	0.4225	0.4308	0.4391	0.4474	0.4555	0.4636	0.4716
Bhutan	0.2037	0.2081	0.2125	0.2177	0.2233	0.2296	0.2375	0.2479	0.2591	0.2704	0.2818	0.2909	0.2994	0.3078	0.3166	0.3266	0.3382	0.3504	0.3624	0.3744	0.3861	0.3978	0.4094	0.4203	0.4306	0.4405	0.4498	0.4602	0.4703	0.4802	0.4901	0.4995	0.5084	0.5167	0.5245	0.5321
India	0.2518	0.2567	0.2617	0.2674	0.2733	0.2795	0.2863	0.2935	0.3018	0.3105	0.3195	0.3280	0.3364	0.3448	0.3535	0.3625	0.3716	0.3806	0.3895	0.3989	0.4081	0.4171	0.4260	0.4352	0.4447	0.4544	0.4650	0.4759	0.4865	0.4970	0.5080	0.5186	0.5286	0.5383	0.5476	0.5564
Andhra Pradesh	0.2137	0.2194	0.2250	0.2314	0.2373	0.2435	0.2498	0.2575	0.2674	0.2778	0.2890	0.3002	0.3104	0.3222	0.3345	0.3476	0.3606	0.3724	0.3857	0.3989	0.4128	0.4263	0.4391	0.4520	0.4647	0.4768	0.4903	0.5054	0.5195	0.5327	0.5479	0.5619	0.5748	0.5870	0.5979	0.6079
Andhra Pradesh, Rural	0.1747	0.1796	0.1845	0.1902	0.1954	0.2009	0.2066	0.2136	0.2227	0.2323	0.2428	0.2534	0.2633	0.2748	0.2870	0.3001	0.3134	0.3256	0.3395	0.3534	0.3680	0.3822	0.3956	0.4090	0.4220	0.4342	0.4476	0.4623	0.4758	0.4882	0.5024	0.5154	0.5277	0.5393	0.5501	0.5598
Andhra Pradesh, Urban	0.3473	0.3527	0.3579	0.3639	0.3691	0.3746	0.3800	0.3870	0.3963	0.4061	0.4167	0.4272	0.4364	0.4474	0.4588	0.4711	0.4831	0.4938	0.5062	0.5183	0.5310	0.5428	0.5534	0.5638	0.5738	0.5831	0.5939	0.6065	0.6182	0.6293	0.6429	0.6559	0.6685	0.6810	0.6932	0.7046
ArunÄ chal Pradesh	0.1910	0.1991	0.2079	0.2167	0.2263	0.2371	0.2481	0.2586	0.2695	0.2796	0.2906	0.3017	0.3134	0.3254	0.3366	0.3491	0.3606	0.3723	0.3830	0.3933	0.4037	0.4144	0.4238	0.4337	0.4448	0.4557	0.4662	0.4771	0.4880	0.5028	0.5169	0.5301	0.5420	0.5534	0.5623	0.5707
ArunÄ chal Pradesh, Rural	0.1836	0.1910	0.1991	0.2072	0.2160	0.2259	0.2361	0.2456	0.2555	0.2647	0.2747	0.2847	0.2953	0.3063	0.3165	0.3279	0.3384	0.3491	0.3590	0.3683	0.3779	0.3879	0.3968	0.4062	0.4171	0.4277	0.4379	0.4485	0.4589	0.4732	0.4865	0.4985	0.5090	0.5186	0.5266	0.5340
ArunÄ chal Pradesh, Urban	0.2973	0.3085	0.3201	0.3315	0.3433	0.3561	0.3691	0.3809	0.3929	0.4036	0.4149	0.4261	0.4375	0.4490	0.4592	0.4706	0.4806	0.4905	0.4991	0.5071	0.5154	0.5243	0.5318	0.5400	0.5498	0.5594	0.5688	0.5787	0.5886	0.6031	0.6168	0.6296	0.6413	0.6524	0.6626	0.6724
Assam	0.2347	0.2416	0.2488	0.2569	0.2661	0.2750	0.2836	0.2923	0.3002	0.3091	0.3181	0.3252	0.3319	0.3383	0.3448	0.3516	0.3580	0.3641	0.3712	0.3811	0.3908	0.4001	0.4096	0.4185	0.4274	0.4357	0.4447	0.4534	0.4614	0.4694	0.4784	0.4869	0.4946	0.5024	0.5103	0.5182
Assam, Rural	0.2190	0.2257	0.2327	0.2407	0.2496	0.2583	0.2668	0.2753	0.2831	0.2919	0.3008	0.3078	0.3144	0.3207	0.3270	0.3337	0.3400	0.3460	0.3530	0.3628	0.3725	0.3818	0.3913	0.4002	0.4091	0.4175	0.4264	0.4351	0.4430	0.4508	0.4595	0.4676	0.4748	0.4821	0.4894	0.4967
Assam, Urban	0.3788	0.3861	0.3936	0.4019	0.4113	0.4201	0.4284	0.4366	0.4437	0.4518	0.4598	0.4657	0.4710	0.4760	0.4810	0.4862	0.4908	0.4951	0.5002	0.5083	0.5163	0.5238	0.5316	0.5389	0.5463	0.5535	0.5616	0.5697	0.5773	0.5853	0.5946	0.6040	0.6130	0.6226	0.6329	0.6437
BihÄ r	0.1901	0.1939	0.1983	0.2029	0.2089	0.2150	0.2221	0.2288	0.2369	0.2444	0.2524	0.2594	0.2659	0.2693	0.2726	0.2742	0.2776	0.2811	0.2801	0.2813	0.2851	0.2888	0.2947	0.2997	0.3067	0.3133	0.3250	0.3375	0.3515	0.3637	0.3774	0.3912	0.4051	0.4183	0.4308	0.4421
BihÄ r, Rural	0.1722	0.1757	0.1797	0.1838	0.1894	0.1950	0.2015	0.2077	0.2153	0.2223	0.2300	0.2369	0.2435	0.2473	0.2511	0.2532	0.2572	0.2612	0.2608	0.2624	0.2667	0.2708	0.2771	0.2823	0.2894	0.2961	0.3080	0.3205	0.3345	0.3467	0.3604	0.3740	0.3878	0.4008	0.4132	0.4243
BihÄ r, Urban	0.3349	0.3396	0.3447	0.3500	0.3568	0.3634	0.3710	0.3781	0.3865	0.3942	0.4024	0.4096	0.4163	0.4198	0.4233	0.4251	0.4289	0.4327	0.4323	0.4338	0.4373	0.4402	0.4448	0.4482	0.4532	0.4579	0.4674	0.4778	0.4901	0.5009	0.5135	0.5264	0.5398	0.5527	0.5651	0.5767
ChhattÄ«sgarh	0.2506	0.2560	0.2610	0.2661	0.2710	0.2758	0.2810	0.2865	0.2927	0.2992	0.3061	0.3123	0.3190	0.3257	0.3321	0.3388	0.3458	0.3533	0.3609	0.3686	0.3750	0.3831	0.3911	0.4009	0.4107	0.4214	0.4337	0.4469	0.4598	0.4707	0.4806	0.4908	0.4999	0.5085	0.5162	0.5235
ChhattÄ«sgarh, Rural	0.2194	0.2246	0.2295	0.2343	0.2391	0.2438	0.2488	0.2541	0.2601	0.2664	0.2731	0.2792	0.2858	0.2923	0.2986	0.3052	0.3122	0.3197	0.3272	0.3349	0.3412	0.3491	0.3569	0.3665	0.3759	0.3863	0.3983	0.4111	0.4237	0.4342	0.4437	0.4536	0.4623	0.4705	0.4779	0.4848
ChhattÄ«sgarh, Urban	0.3942	0.3992	0.4039	0.4086	0.4132	0.4177	0.4227	0.4280	0.4340	0.4404	0.4470	0.4530	0.4594	0.4656	0.4716	0.4778	0.4843	0.4912	0.4980	0.5050	0.5104	0.5174	0.5242	0.5327	0.5411	0.5505	0.5615	0.5734	0.5853	0.5952	0.6043	0.6140	0.6229	0.6316	0.6405	0.6492
Delhi	0.4357	0.4416	0.4479	0.4533	0.4588	0.4656	0.4730	0.4806	0.4886	0.4967	0.5044	0.5131	0.5216	0.5300	0.5385	0.5463	0.5536	0.5630	0.5717	0.5802	0.5891	0.5974	0.6058	0.6146	0.6239	0.6338	0.6441	0.6549	0.6661	0.6785	0.6907	0.7034	0.7158	0.7282	0.7405	0.7529
Delhi, Rural	0.3536	0.3602	0.3674	0.3740	0.3808	0.3890	0.3979	0.4073	0.4172	0.4274	0.4373	0.4481	0.4584	0.4686	0.4789	0.4885	0.4978	0.5091	0.5197	0.5302	0.5410	0.5511	0.5610	0.5708	0.5809	0.5913	0.6016	0.6122	0.6228	0.6342	0.6450	0.6557	0.6656	0.6751	0.6840	0.6927
Delhi, Urban	0.4436	0.4492	0.4553	0.4605	0.4659	0.4726	0.4799	0.4875	0.4955	0.5038	0.5115	0.5201	0.5281	0.5359	0.5438	0.5511	0.5580	0.5671	0.5756	0.5839	0.5926	0.6008	0.6089	0.6173	0.6264	0.6361	0.6462	0.6568	0.6678	0.6801	0.6922	0.7049	0.7173	0.7298	0.7424	0.7550
Goa	0.4344	0.4383	0.4435	0.4478	0.4538	0.4580	0.4637	0.4694	0.4755	0.4822	0.4886	0.4956	0.5037	0.5128	0.5215	0.5311	0.5409	0.5505	0.5633	0.5743	0.5850	0.5946	0.6041	0.6123	0.6210	0.6307	0.6399	0.6494	0.6612	0.6723	0.6826	0.6944	0.7032	0.7116	0.7207	0.7298
Goa, Rural	0.4210	0.4242	0.4287	0.4324	0.4376	0.4411	0.4460	0.4510	0.4563	0.4624	0.4681	0.4744	0.4819	0.4904	0.4986	0.5077	0.5171	0.5263	0.5387	0.5492	0.5594	0.5686	0.5776	0.5852	0.5934	0.6023	0.6109	0.6196	0.6306	0.6408	0.6502	0.6611	0.6689	0.6763	0.6849	0.6932
Goa, Urban	0.4665	0.4705	0.4758	0.4802	0.4861	0.4903	0.4959	0.5015	0.5074	0.5140	0.5202	0.5268	0.5346	0.5432	0.5515	0.5606	0.5699	0.5789	0.5912	0.6016	0.6117	0.6208	0.6297	0.6372	0.6453	0.6543	0.6629	0.6718	0.6831	0.6937	0.7036	0.7150	0.7234	0.7315	0.7410	0.7504
GujarÄ t	0.2808	0.2873	0.2935	0.3017	0.3092	0.3160	0.3239	0.3304	0.3401	0.3496	0.3588	0.3663	0.3764	0.3861	0.3978	0.4091	0.4205	0.4310	0.4415	0.4506	0.4584	0.4664	0.4747	0.4838	0.4927	0.5030	0.5131	0.5235	0.5329	0.5431	0.5534	0.5632	0.5719	0.5801	0.5880	0.5957
GujarÄ t, Rural	0.2355	0.2414	0.2470	0.2546	0.2615	0.2679	0.2754	0.2816	0.2909	0.3001	0.3089	0.3163	0.3261	0.3356	0.3470	0.3581	0.3694	0.3798	0.3903	0.3994	0.4072	0.4151	0.4230	0.4317	0.4401	0.4498	0.4594	0.4692	0.4781	0.4877	0.4973	0.5062	0.5141	0.5212	0.5283	0.5349
GujarÄ t, Urban	0.3817	0.3882	0.3942	0.4024	0.4095	0.4158	0.4232	0.4289	0.4380	0.4469	0.4552	0.4617	0.4709	0.4797	0.4906	0.5011	0.5117	0.5213	0.5309	0.5390	0.5457	0.5524	0.5592	0.5668	0.5741	0.5830	0.5920	0.6015	0.6101	0.6200	0.6302	0.6401	0.6492	0.6581	0.6674	0.6766
HaryÄ na	0.2694	0.2765	0.2844	0.2921	0.3000	0.3093	0.3183	0.3276	0.3390	0.3503	0.3625	0.3744	0.3852	0.3967	0.4086	0.4204	0.4331	0.4451	0.4568	0.4688	0.4813	0.4936	0.5053	0.5168	0.5281	0.5396	0.5513	0.5633	0.5750	0.5864	0.5976	0.6081	0.6178	0.6268	0.6349	0.6427
HaryÄ na, Rural	0.2385	0.2452	0.2527	0.2600	0.2675	0.2763	0.2848	0.2936	0.3045	0.3154	0.3271	0.3387	0.3493	0.3606	0.3725	0.3843	0.3972	0.4094	0.4214	0.4336	0.4462	0.4584	0.4699	0.4809	0.4917	0.5027	0.5141	0.5258	0.5374	0.5488	0.5598	0.5700	0.5788	0.5867	0.5938	0.6003
HaryÄ na, Urban	0.3938	0.4001	0.4071	0.4137	0.4203	0.4284	0.4360	0.4438	0.4538	0.4636	0.4743	0.4847	0.4937	0.5034	0.5134	0.5233	0.5339	0.5434	0.5525	0.5617	0.5713	0.5804	0.5888	0.5969	0.6050	0.6137	0.6233	0.6338	0.6446	0.6559	0.6673	0.6782	0.6881	0.6973	0.7067	0.7159
Himachal Pradesh	0.2884	0.2939	0.2990	0.3046	0.3090	0.3152	0.3220	0.3294	0.3392	0.3493	0.3595	0.3693	0.3795	0.3898	0.4007	0.4116	0.4246	0.4373	0.4506	0.4653	0.4798	0.4932	0.5054	0.5169	0.5279	0.5383	0.5486	0.5587	0.5687	0.5785	0.5894	0.5998	0.6095	0.6187	0.6275	0.6360
Himachal Pradesh, Rural	0.2777	0.2831	0.2881	0.2935	0.2979	0.3039	0.3107	0.3180	0.3277	0.3377	0.3479	0.3577	0.3679	0.3782	0.3890	0.4000	0.4131	0.4259	0.4392	0.4540	0.4685	0.4821	0.4945	0.5060	0.5172	0.5276	0.5380	0.5480	0.5579	0.5676	0.5782	0.5883	0.5974	0.6061	0.6143	0.6221
Himachal Pradesh, Urban	0.4217	0.4273	0.4322	0.4374	0.4411	0.4466	0.4525	0.4589	0.4677	0.4766	0.4853	0.4935	0.5019	0.5103	0.5190	0.5278	0.5387	0.5492	0.5603	0.5729	0.5853	0.5967	0.6073	0.6172	0.6271	0.6367	0.6466	0.6567	0.6673	0.6783	0.6911	0.7040	0.7169	0.7301	0.7437	0.7578
Jammu and KashmÄ«r	0.2588	0.2639	0.2696	0.2757	0.2824	0.2892	0.2965	0.3020	0.3093	0.3160	0.3221	0.3273	0.3326	0.3438	0.3544	0.3656	0.3767	0.3929	0.4072	0.4246	0.4398	0.4531	0.4652	0.4753	0.4849	0.4930	0.5008	0.5079	0.5158	0.5234	0.5313	0.5398	0.5483	0.5568	0.5657	0.5745
Jammu and KashmÄ«r, Rural	0.2289	0.2336	0.2389	0.2446	0.2508	0.2573	0.2643	0.2695	0.2765	0.2831	0.2891	0.2941	0.2994	0.3106	0.3212	0.3324	0.3436	0.3599	0.3745	0.3920	0.4076	0.4211	0.4333	0.4436	0.4533	0.4613	0.4691	0.4761	0.4837	0.4909	0.4984	0.5063	0.5140	0.5217	0.5300	0.5381
Jammu and KashmÄ«r, Urban	0.3757	0.3810	0.3870	0.3932	0.3999	0.4067	0.4139	0.4190	0.4258	0.4318	0.4370	0.4411	0.4452	0.4553	0.4645	0.4741	0.4834	0.4977	0.5100	0.5254	0.5386	0.5499	0.5600	0.5685	0.5766	0.5835	0.5904	0.5967	0.6041	0.6114	0.6194	0.6283	0.6375	0.6472	0.6574	0.6678
Jharkhand	0.2816	0.2855	0.2890	0.2925	0.2960	0.2996	0.3037	0.3083	0.3137	0.3195	0.3256	0.3309	0.3365	0.3419	0.3470	0.3519	0.3562	0.3635	0.3703	0.3765	0.3807	0.3860	0.3917	0.3979	0.4064	0.4149	0.4231	0.4321	0.4395	0.4482	0.4580	0.4660	0.4731	0.4801	0.4876	0.4949
Jharkhand, Rural	0.2662	0.2698	0.2729	0.2760	0.2791	0.2822	0.2858	0.2900	0.2949	0.3003	0.3061	0.3112	0.3168	0.3223	0.3277	0.3328	0.3374	0.3451	0.3522	0.3587	0.3631	0.3686	0.3744	0.3806	0.3890	0.3974	0.4054	0.4143	0.4213	0.4298	0.4392	0.4468	0.4536	0.4601	0.4673	0.4743
Jharkhand, Urban	0.4063	0.4110	0.4153	0.4196	0.4238	0.4279	0.4326	0.4377	0.4435	0.4498	0.4564	0.4624	0.4690	0.4755	0.4821	0.4886	0.4946	0.5037	0.5122	0.5202	0.5259	0.5322	0.5386	0.5450	0.5535	0.5619	0.5700	0.5791	0.5869	0.5964	0.6070	0.6160	0.6242	0.6324	0.6410	0.6497
KarnÄ taka	0.2598	0.2646	0.2698	0.2762	0.2830	0.2894	0.2970	0.3051	0.3139	0.3232	0.3325	0.3432	0.3532	0.3636	0.3744	0.3854	0.3966	0.4076	0.4202	0.4324	0.4444	0.4550	0.4651	0.4746	0.4851	0.4966	0.5078	0.5195	0.5305	0.5403	0.5515	0.5616	0.5709	0.5795	0.5877	0.5957
KarnÄ taka, Rural	0.2158	0.2201	0.2247	0.2305	0.2368	0.2428	0.2499	0.2576	0.2659	0.2748	0.2838	0.2940	0.3036	0.3136	0.3239	0.3346	0.3456	0.3564	0.3690	0.3813	0.3937	0.4050	0.4159	0.4263	0.4376	0.4496	0.4608	0.4721	0.4821	0.4907	0.5003	0.5090	0.5172	0.5250	0.5327	0.5402
KarnÄ taka, Urban	0.3750	0.3796	0.3846	0.3908	0.3974	0.4035	0.4107	0.4183	0.4265	0.4351	0.4436	0.4533	0.4620	0.4710	0.4801	0.4896	0.4992	0.5087	0.5201	0.5311	0.5421	0.5517	0.5609	0.5695	0.5791	0.5897	0.5995	0.6099	0.6191	0.6272	0.6370	0.6464	0.6558	0.6655	0.6756	0.6857
Kerala	0.3417	0.3455	0.3507	0.3569	0.3636	0.3699	0.3774	0.3851	0.3927	0.4009	0.4091	0.4178	0.4262	0.4353	0.4450	0.4559	0.4664	0.4765	0.4872	0.4982	0.5083	0.5170	0.5253	0.5332	0.5415	0.5509	0.5600	0.5695	0.5789	0.5890	0.5987	0.6089	0.6187	0.6282	0.6382	0.6484
Kerala, Rural	0.3316	0.3353	0.3403	0.3464	0.3530	0.3591	0.3666	0.3742	0.3819	0.3900	0.3982	0.4069	0.4153	0.4245	0.4342	0.4453	0.4560	0.4664	0.4774	0.4888	0.4994	0.5082	0.5166	0.5243	0.5324	0.5413	0.5497	0.5584	0.5668	0.5757	0.5841	0.5931	0.6018	0.6104	0.6197	0.6291
Kerala, Urban	0.3798	0.3832	0.3879	0.3936	0.3996	0.4052	0.4120	0.4189	0.4258	0.4331	0.4404	0.4482	0.4557	0.4639	0.4728	0.4830	0.4928	0.5023	0.5125	0.5231	0.5329	0.5412	0.5491	0.5565	0.5644	0.5732	0.5816	0.5903	0.5988	0.6081	0.6172	0.6273	0.6374	0.6478	0.6595	0.6716
Madhya Pradesh	0.2066	0.2114	0.2168	0.2228	0.2282	0.2345	0.2406	0.2486	0.2575	0.2666	0.2770	0.2857	0.2943	0.3025	0.3105	0.3188	0.3273	0.3357	0.3433	0.3519	0.3595	0.3683	0.3759	0.3853	0.3941	0.4025	0.4117	0.4211	0.4307	0.4416	0.4519	0.4626	0.4735	0.4851	0.4958	0.5058
Madhya Pradesh, Rural	0.1639	0.1679	0.1723	0.1773	0.1819	0.1872	0.1924	0.1994	0.2073	0.2157	0.2253	0.2335	0.2419	0.2500	0.2580	0.2663	0.2748	0.2829	0.2902	0.2982	0.3051	0.3132	0.3202	0.3291	0.3375	0.3457	0.3551	0.3649	0.3753	0.3870	0.3980	0.4091	0.4198	0.4306	0.4407	0.4498
Madhya Pradesh, Urban	0.3873	0.3916	0.3965	0.4021	0.4067	0.4123	0.4173	0.4247	0.4329	0.4413	0.4511	0.4589	0.4666	0.4738	0.4807	0.4878	0.4949	0.5013	0.5064	0.5121	0.5163	0.5216	0.5253	0.5310	0.5363	0.5418	0.5490	0.5571	0.5666	0.5781	0.5892	0.6007	0.6120	0.6235	0.6350	0.6458
MahÄ rÄshtra	0.3133	0.3186	0.3238	0.3295	0.3351	0.3415	0.3480	0.3554	0.3640	0.3743	0.3844	0.3935	0.4040	0.4149	0.4253	0.4365	0.4473	0.4568	0.4657	0.4752	0.4836	0.4922	0.5009	0.5099	0.5193	0.5295	0.5402	0.5514	0.5615	0.5715	0.5821	0.5919	0.6007	0.6091	0.6172	0.6251

Appendix Table 5. Socio-Demographic Index values for all estimated GBD 2015 locations, 1980–2015																																				
**Location**	**1980**	**1981**	**1982**	**1983**	**1984**	**1985**	**1986**	**1987**	**1988**	**1989**	**1990**	**1991**	**1992**	**1993**	**1994**	**1995**	**1996**	**1997**	**1998**	**1999**	**2000**	**2001**	**2002**	**2003**	**2004**	**2005**	**2006**	**2007**	**2008**	**2009**	**2010**	**2011**	**2012**	**2013**	**2014**	**2015**
MahÄ rÄshtra, Rural	0.2509	0.2557	0.2605	0.2657	0.2710	0.2770	0.2833	0.2905	0.2987	0.3086	0.3184	0.3273	0.3376	0.3483	0.3586	0.3699	0.3809	0.3911	0.4008	0.4113	0.4209	0.4306	0.4405	0.4504	0.4607	0.4715	0.4825	0.4938	0.5038	0.5134	0.5233	0.5323	0.5401	0.5472	0.5540	0.5605
MahÄ rÄshtra, Urban	0.4320	0.4367	0.4411	0.4459	0.4505	0.4559	0.4611	0.4674	0.4746	0.4835	0.4919	0.4990	0.5072	0.5157	0.5235	0.5323	0.5406	0.5479	0.5546	0.5622	0.5688	0.5755	0.5823	0.5893	0.5969	0.6055	0.6147	0.6247	0.6339	0.6434	0.6538	0.6640	0.6735	0.6829	0.6928	0.7027
Manipur	0.2974	0.3036	0.3091	0.3157	0.3229	0.3297	0.3378	0.3484	0.3586	0.3674	0.3758	0.3841	0.3918	0.4000	0.4071	0.4145	0.4221	0.4300	0.4384	0.4497	0.4590	0.4685	0.4764	0.4839	0.4925	0.5019	0.5096	0.5168	0.5228	0.5300	0.5364	0.5444	0.5520	0.5597	0.5675	0.5757
Manipur, Rural	0.2835	0.2892	0.2942	0.3002	0.3068	0.3130	0.3205	0.3305	0.3401	0.3484	0.3564	0.3643	0.3719	0.3800	0.3872	0.3947	0.4025	0.4105	0.4191	0.4305	0.4399	0.4494	0.4573	0.4646	0.4729	0.4820	0.4893	0.4961	0.5015	0.5080	0.5136	0.5208	0.5275	0.5341	0.5411	0.5485
Manipur, Urban	0.3634	0.3692	0.3742	0.3801	0.3865	0.3925	0.3996	0.4091	0.4182	0.4258	0.4331	0.4404	0.4474	0.4549	0.4616	0.4686	0.4758	0.4833	0.4914	0.5023	0.5110	0.5200	0.5274	0.5343	0.5424	0.5514	0.5587	0.5656	0.5715	0.5786	0.5850	0.5931	0.6009	0.6088	0.6174	0.6266
MeghÄ laya	0.2307	0.2341	0.2376	0.2416	0.2463	0.2516	0.2577	0.2648	0.2715	0.2807	0.2897	0.2978	0.3052	0.3133	0.3205	0.3290	0.3368	0.3453	0.3541	0.3634	0.3733	0.3832	0.3918	0.3998	0.4078	0.4161	0.4243	0.4324	0.4403	0.4487	0.4563	0.4641	0.4704	0.4767	0.4836	0.4905
MeghÄ laya, Rural	0.2008	0.2036	0.2066	0.2100	0.2142	0.2189	0.2244	0.2309	0.2371	0.2456	0.2542	0.2617	0.2688	0.2766	0.2836	0.2918	0.2994	0.3077	0.3162	0.3253	0.3351	0.3448	0.3533	0.3612	0.3691	0.3771	0.3851	0.3930	0.4005	0.4084	0.4154	0.4224	0.4276	0.4327	0.4387	0.4444
MeghÄ laya, Urban	0.3849	0.3892	0.3935	0.3983	0.4038	0.4097	0.4164	0.4242	0.4312	0.4411	0.4507	0.4588	0.4662	0.4742	0.4810	0.4893	0.4966	0.5048	0.5133	0.5223	0.5318	0.5413	0.5495	0.5571	0.5651	0.5736	0.5823	0.5911	0.6000	0.6096	0.6189	0.6289	0.6374	0.6466	0.6566	0.6670
Mizoram	0.2869	0.2902	0.2938	0.2989	0.3072	0.3195	0.3325	0.3479	0.3585	0.3674	0.3746	0.3835	0.3918	0.4007	0.4071	0.4154	0.4258	0.4328	0.4392	0.4466	0.4556	0.4641	0.4723	0.4793	0.4856	0.4914	0.4968	0.5027	0.5088	0.5164	0.5245	0.5316	0.5395	0.5472	0.5557	0.5642
Mizoram, Rural	0.2380	0.2405	0.2433	0.2477	0.2553	0.2670	0.2792	0.2939	0.3039	0.3122	0.3190	0.3274	0.3353	0.3437	0.3498	0.3578	0.3678	0.3746	0.3808	0.3880	0.3967	0.4048	0.4127	0.4192	0.4250	0.4302	0.4350	0.4403	0.4455	0.4522	0.4591	0.4650	0.4714	0.4775	0.4846	0.4916
Mizoram, Urban	0.3512	0.3548	0.3587	0.3640	0.3724	0.3849	0.3980	0.4137	0.4244	0.4331	0.4401	0.4488	0.4568	0.4653	0.4712	0.4791	0.4891	0.4954	0.5011	0.5080	0.5165	0.5245	0.5325	0.5392	0.5453	0.5511	0.5564	0.5625	0.5686	0.5766	0.5851	0.5927	0.6012	0.6097	0.6193	0.6290
NÄ gÄland	0.3069	0.3125	0.3196	0.3267	0.3344	0.3416	0.3489	0.3585	0.3687	0.3783	0.3879	0.3965	0.4048	0.4159	0.4257	0.4351	0.4439	0.4527	0.4587	0.4643	0.4734	0.4823	0.4916	0.4994	0.5074	0.5151	0.5222	0.5312	0.5405	0.5493	0.5575	0.5667	0.5750	0.5829	0.5907	0.5987
NÄ gÄland, Rural	0.2973	0.3024	0.3092	0.3158	0.3230	0.3298	0.3367	0.3459	0.3556	0.3648	0.3741	0.3824	0.3906	0.4015	0.4113	0.4207	0.4295	0.4383	0.4443	0.4499	0.4588	0.4673	0.4760	0.4830	0.4900	0.4965	0.5022	0.5098	0.5174	0.5244	0.5308	0.5382	0.5446	0.5507	0.5574	0.5644
NÄ gÄland, Urban	0.3805	0.3865	0.3941	0.4014	0.4091	0.4162	0.4234	0.4327	0.4425	0.4517	0.4609	0.4690	0.4768	0.4873	0.4965	0.5054	0.5135	0.5217	0.5271	0.5323	0.5410	0.5497	0.5589	0.5667	0.5750	0.5830	0.5906	0.6004	0.6104	0.6201	0.6293	0.6396	0.6490	0.6583	0.6682	0.6786
Orissa	0.2042	0.2086	0.2127	0.2192	0.2242	0.2308	0.2377	0.2435	0.2516	0.2604	0.2668	0.2747	0.2820	0.2899	0.2988	0.3093	0.3170	0.3263	0.3367	0.3480	0.3579	0.3676	0.3763	0.3872	0.3989	0.4095	0.4224	0.4368	0.4493	0.4603	0.4720	0.4819	0.4916	0.5005	0.5088	0.5167
Orissa, Rural	0.1857	0.1897	0.1934	0.1994	0.2039	0.2100	0.2164	0.2217	0.2292	0.2375	0.2434	0.2509	0.2578	0.2653	0.2738	0.2840	0.2916	0.3007	0.3111	0.3223	0.3324	0.3423	0.3512	0.3623	0.3742	0.3849	0.3978	0.4120	0.4242	0.4348	0.4459	0.4553	0.4646	0.4731	0.4813	0.4889
Orissa, Urban	0.3645	0.3690	0.3731	0.3797	0.3844	0.3908	0.3973	0.4023	0.4098	0.4179	0.4233	0.4302	0.4364	0.4432	0.4510	0.4604	0.4668	0.4748	0.4840	0.4940	0.5027	0.5110	0.5183	0.5279	0.5384	0.5477	0.5595	0.5729	0.5845	0.5947	0.6059	0.6156	0.6255	0.6350	0.6447	0.6542
Punjab	0.3218	0.3285	0.3355	0.3425	0.3502	0.3584	0.3668	0.3763	0.3861	0.3970	0.4073	0.4175	0.4279	0.4382	0.4481	0.4581	0.4682	0.4779	0.4873	0.4973	0.5075	0.5172	0.5257	0.5341	0.5420	0.5505	0.5592	0.5691	0.5788	0.5886	0.5981	0.6074	0.6160	0.6241	0.6319	0.6397
Punjab, Rural	0.2883	0.2947	0.3015	0.3083	0.3158	0.3239	0.3322	0.3416	0.3512	0.3621	0.3723	0.3823	0.3926	0.4025	0.4123	0.4221	0.4321	0.4419	0.4515	0.4617	0.4724	0.4826	0.4918	0.5010	0.5096	0.5188	0.5278	0.5379	0.5476	0.5570	0.5660	0.5747	0.5826	0.5900	0.5971	0.6039
Punjab, Urban	0.4152	0.4215	0.4281	0.4345	0.4415	0.4490	0.4566	0.4651	0.4739	0.4839	0.4930	0.5019	0.5109	0.5196	0.5278	0.5361	0.5444	0.5523	0.5599	0.5682	0.5767	0.5846	0.5914	0.5981	0.6044	0.6114	0.6185	0.6271	0.6357	0.6445	0.6533	0.6625	0.6714	0.6804	0.6898	0.6994
RÄ jasthÄn	0.1817	0.1862	0.1912	0.1981	0.2035	0.2092	0.2156	0.2219	0.2312	0.2400	0.2511	0.2604	0.2703	0.2795	0.2906	0.3014	0.3131	0.3237	0.3337	0.3454	0.3558	0.3669	0.3760	0.3876	0.3985	0.4091	0.4211	0.4333	0.4451	0.4560	0.4695	0.4827	0.4943	0.5046	0.5140	0.5226
RÄ jasthÄn, Rural	0.1452	0.1488	0.1530	0.1590	0.1636	0.1684	0.1739	0.1793	0.1878	0.1957	0.2060	0.2147	0.2241	0.2329	0.2437	0.2542	0.2656	0.2761	0.2857	0.2972	0.3075	0.3185	0.3280	0.3400	0.3513	0.3623	0.3746	0.3869	0.3987	0.4095	0.4228	0.4357	0.4470	0.4568	0.4660	0.4743
RÄ jasthÄn, Urban	0.3283	0.3336	0.3395	0.3474	0.3537	0.3600	0.3672	0.3740	0.3841	0.3934	0.4051	0.4149	0.4252	0.4348	0.4463	0.4575	0.4695	0.4806	0.4908	0.5028	0.5132	0.5237	0.5317	0.5419	0.5511	0.5599	0.5700	0.5804	0.5905	0.6000	0.6124	0.6249	0.6363	0.6469	0.6572	0.6671
Sikkim	0.2633	0.2674	0.2727	0.2784	0.2866	0.2959	0.3065	0.3169	0.3264	0.3353	0.3440	0.3511	0.3567	0.3654	0.3735	0.3818	0.3908	0.4001	0.4094	0.4184	0.4284	0.4384	0.4487	0.4589	0.4691	0.4792	0.4890	0.4988	0.5091	0.5311	0.5494	0.5657	0.5798	0.5927	0.6033	0.6125
Sikkim, Rural	0.2537	0.2577	0.2627	0.2681	0.2760	0.2850	0.2953	0.3054	0.3146	0.3233	0.3317	0.3385	0.3438	0.3522	0.3600	0.3679	0.3766	0.3855	0.3944	0.4030	0.4124	0.4218	0.4313	0.4406	0.4498	0.4587	0.4672	0.4756	0.4844	0.5045	0.5210	0.5355	0.5479	0.5591	0.5690	0.5775
Sikkim, Urban	0.3666	0.3713	0.3771	0.3832	0.3920	0.4020	0.4134	0.4245	0.4345	0.4437	0.4525	0.4596	0.4648	0.4734	0.4813	0.4893	0.4981	0.5072	0.5162	0.5249	0.5346	0.5442	0.5540	0.5636	0.5733	0.5829	0.5921	0.6015	0.6116	0.6348	0.6543	0.6722	0.6882	0.7034	0.7174	0.7303
Tamil NÄ du	0.2800	0.2857	0.2903	0.2957	0.3026	0.3102	0.3187	0.3283	0.3378	0.3483	0.3587	0.3688	0.3794	0.3915	0.4037	0.4157	0.4276	0.4395	0.4525	0.4647	0.4769	0.4872	0.4964	0.5051	0.5144	0.5250	0.5360	0.5466	0.5564	0.5682	0.5800	0.5911	0.6010	0.6107	0.6201	0.6290
Tamil NÄ du, Rural	0.2369	0.2423	0.2467	0.2520	0.2589	0.2666	0.2751	0.2847	0.2943	0.3046	0.3148	0.3244	0.3343	0.3454	0.3565	0.3674	0.3784	0.3897	0.4024	0.4148	0.4276	0.4389	0.4495	0.4597	0.4705	0.4822	0.4939	0.5047	0.5140	0.5249	0.5354	0.5454	0.5545	0.5636	0.5725	0.5809
Tamil NÄ du, Urban	0.3685	0.3744	0.3791	0.3847	0.3918	0.3996	0.4081	0.4177	0.4269	0.4367	0.4460	0.4543	0.4625	0.4718	0.4810	0.4897	0.4986	0.5078	0.5184	0.5287	0.5395	0.5487	0.5572	0.5654	0.5741	0.5839	0.5938	0.6029	0.6109	0.6207	0.6308	0.6408	0.6507	0.6612	0.6721	0.6829
Telangana	0.2724	0.2780	0.2833	0.2887	0.2942	0.2997	0.3059	0.3125	0.3199	0.3277	0.3360	0.3441	0.3526	0.3613	0.3705	0.3805	0.3909	0.4014	0.4121	0.4231	0.4341	0.4449	0.4553	0.4658	0.4759	0.4866	0.4968	0.5068	0.5163	0.5238	0.5313	0.5381	0.5435	0.5479	0.5522	0.5568
Telangana, Rural	0.2282	0.2331	0.2378	0.2427	0.2476	0.2527	0.2584	0.2645	0.2714	0.2788	0.2867	0.2945	0.3030	0.3117	0.3210	0.3313	0.3422	0.3533	0.3648	0.3767	0.3886	0.4002	0.4114	0.4224	0.4330	0.4439	0.4541	0.4639	0.4729	0.4796	0.4864	0.4925	0.4974	0.5015	0.5057	0.5103
Telangana, Urban	0.4243	0.4288	0.4328	0.4368	0.4407	0.4446	0.4491	0.4540	0.4597	0.4660	0.4726	0.4789	0.4858	0.4927	0.5001	0.5085	0.5174	0.5263	0.5356	0.5450	0.5543	0.5630	0.5708	0.5785	0.5856	0.5933	0.6006	0.6077	0.6146	0.6196	0.6252	0.6306	0.6353	0.6397	0.6447	0.6505
Tripura	0.2541	0.2592	0.2646	0.2696	0.2746	0.2795	0.2849	0.2907	0.2993	0.3073	0.3142	0.3198	0.3244	0.3316	0.3372	0.3448	0.3531	0.3622	0.3710	0.3878	0.4043	0.4200	0.4329	0.4450	0.4561	0.4660	0.4747	0.4822	0.4901	0.4975	0.5048	0.5127	0.5198	0.5269	0.5339	0.5412
Tripura, Rural	0.2381	0.2428	0.2480	0.2527	0.2574	0.2621	0.2672	0.2729	0.2812	0.2890	0.2958	0.3012	0.3058	0.3129	0.3186	0.3261	0.3344	0.3435	0.3522	0.3689	0.3852	0.4004	0.4127	0.4241	0.4343	0.4433	0.4509	0.4572	0.4638	0.4700	0.4760	0.4826	0.4885	0.4944	0.5006	0.5071
Tripura, Urban	0.3649	0.3696	0.3748	0.3792	0.3837	0.3880	0.3927	0.3978	0.4057	0.4128	0.4187	0.4231	0.4265	0.4326	0.4371	0.4435	0.4506	0.4584	0.4660	0.4818	0.4974	0.5123	0.5243	0.5355	0.5457	0.5549	0.5628	0.5695	0.5768	0.5839	0.5911	0.5994	0.6072	0.6155	0.6248	0.6347
Uttar Pradesh	0.2103	0.2137	0.2182	0.2226	0.2274	0.2329	0.2390	0.2455	0.2533	0.2618	0.2709	0.2795	0.2876	0.2951	0.3028	0.3105	0.3191	0.3271	0.3351	0.3433	0.3518	0.3606	0.3704	0.3809	0.3917	0.4031	0.4144	0.4260	0.4373	0.4488	0.4602	0.4713	0.4818	0.4921	0.5017	0.5109
Uttar Pradesh, Rural	0.1855	0.1883	0.1921	0.1958	0.1999	0.2047	0.2101	0.2159	0.2230	0.2308	0.2393	0.2474	0.2550	0.2622	0.2695	0.2770	0.2854	0.2931	0.3009	0.3090	0.3175	0.3265	0.3367	0.3477	0.3590	0.3709	0.3826	0.3946	0.4061	0.4179	0.4295	0.4407	0.4513	0.4614	0.4711	0.4802
Uttar Pradesh, Urban	0.3361	0.3401	0.3453	0.3503	0.3556	0.3616	0.3681	0.3749	0.3829	0.3915	0.4007	0.4093	0.4173	0.4248	0.4324	0.4401	0.4489	0.4570	0.4653	0.4736	0.4817	0.4892	0.4969	0.5046	0.5124	0.5208	0.5293	0.5385	0.5478	0.5578	0.5679	0.5780	0.5877	0.5975	0.6072	0.6168
Uttarakhand	0.2942	0.3009	0.3071	0.3133	0.3195	0.3255	0.3320	0.3388	0.3463	0.3542	0.3622	0.3695	0.3772	0.3847	0.3935	0.4021	0.4104	0.4185	0.4264	0.4356	0.4464	0.4568	0.4681	0.4792	0.4912	0.5044	0.5179	0.5317	0.5447	0.5616	0.5767	0.5905	0.6022	0.6130	0.6225	0.6314
Uttarakhand, Rural	0.2691	0.2756	0.2817	0.2877	0.2937	0.2996	0.3059	0.3125	0.3198	0.3275	0.3354	0.3425	0.3500	0.3573	0.3659	0.3743	0.3824	0.3903	0.3980	0.4071	0.4177	0.4280	0.4391	0.4500	0.4619	0.4749	0.4882	0.5018	0.5144	0.5311	0.5457	0.5592	0.5704	0.5806	0.5896	0.5980
Uttarakhand, Urban	0.4130	0.4179	0.4224	0.4268	0.4311	0.4353	0.4400	0.4451	0.4508	0.4571	0.4635	0.4692	0.4753	0.4812	0.4885	0.4956	0.5023	0.5088	0.5151	0.5227	0.5319	0.5409	0.5507	0.5604	0.5710	0.5829	0.5952	0.6078	0.6197	0.6357	0.6500	0.6632	0.6744	0.6848	0.6951	0.7049
West Bengal	0.2703	0.2748	0.2796	0.2850	0.2913	0.2970	0.3029	0.3101	0.3167	0.3235	0.3302	0.3363	0.3416	0.3474	0.3538	0.3615	0.3692	0.3775	0.3875	0.3976	0.4077	0.4178	0.4272	0.4369	0.4460	0.4548	0.4635	0.4728	0.4816	0.4905	0.4991	0.5079	0.5165	0.5250	0.5334	0.5417
West Bengal, Rural	0.2218	0.2260	0.2304	0.2356	0.2416	0.2471	0.2528	0.2599	0.2664	0.2732	0.2799	0.2861	0.2917	0.2977	0.3045	0.3124	0.3204	0.3291	0.3392	0.3495	0.3597	0.3697	0.3790	0.3884	0.3973	0.4058	0.4144	0.4235	0.4323	0.4412	0.4498	0.4583	0.4665	0.4744	0.4820	0.4895
West Bengal, Urban	0.4086	0.4129	0.4175	0.4227	0.4288	0.4341	0.4396	0.4463	0.4522	0.4583	0.4642	0.4694	0.4738	0.4786	0.4842	0.4910	0.4979	0.5054	0.5146	0.5237	0.5326	0.5409	0.5481	0.5552	0.5616	0.5677	0.5742	0.5815	0.5889	0.5969	0.6051	0.6139	0.6230	0.6324	0.6424	0.6527
The Six Minor Territories	0.4068	0.4123	0.4173	0.4222	0.4270	0.4318	0.4370	0.4426	0.4487	0.4552	0.4618	0.4678	0.4742	0.4803	0.4875	0.4956	0.5047	0.5161	0.5268	0.5374	0.5481	0.5586	0.5693	0.5801	0.5905	0.6018	0.6130	0.6240	0.6348	0.6457	0.6551	0.6644	0.6732	0.6818	0.6903	0.6988
The Six Minor Territories, Rural	0.3487	0.3530	0.3567	0.3603	0.3638	0.3673	0.3711	0.3753	0.3802	0.3855	0.3913	0.3967	0.4028	0.4089	0.4164	0.4249	0.4346	0.4466	0.4579	0.4691	0.4803	0.4912	0.5022	0.5132	0.5235	0.5346	0.5454	0.5559	0.5658	0.5754	0.5833	0.5906	0.5969	0.6027	0.6085	0.6140
The Six Minor Territories, Urban	0.4757	0.4795	0.4826	0.4855	0.4882	0.4908	0.4937	0.4970	0.5009	0.5053	0.5100	0.5143	0.5193	0.5243	0.5306	0.5380	0.5464	0.5572	0.5671	0.5769	0.5867	0.5961	0.6058	0.6154	0.6246	0.6347	0.6448	0.6549	0.6648	0.6749	0.6836	0.6924	0.7006	0.7088	0.7177	0.7268
Nepal	0.1738	0.1776	0.1818	0.1856	0.1903	0.1952	0.2003	0.2053	0.2111	0.2173	0.2238	0.2308	0.2380	0.2455	0.2539	0.2622	0.2707	0.2792	0.2873	0.2953	0.3036	0.3115	0.3186	0.3256	0.3326	0.3396	0.3473	0.3551	0.3634	0.3718	0.3806	0.3896	0.3985	0.4070	0.4152	0.4227
Pakistan	0.2139	0.2191	0.2245	0.2301	0.2359	0.2421	0.2482	0.2549	0.2622	0.2702	0.2786	0.2868	0.2956	0.3043	0.3129	0.3218	0.3308	0.3396	0.3483	0.3571	0.3656	0.3736	0.3811	0.3885	0.3957	0.4029	0.4102	0.4171	0.4238	0.4301	0.4363	0.4424	0.4487	0.4550	0.4613	0.4676
Sub-Saharan Africa	0.2327	0.2359	0.2390	0.2422	0.2455	0.2490	0.2519	0.2549	0.2587	0.2626	0.2666	0.2699	0.2728	0.2757	0.2783	0.2810	0.2837	0.2870	0.2900	0.2932	0.2966	0.2999	0.3033	0.3072	0.3122	0.3180	0.3243	0.3315	0.3387	0.3458	0.3534	0.3610	0.3686	0.3763	0.3839	0.3914
Central Sub-Saharan Africa	0.2324	0.2333	0.2343	0.2357	0.2386	0.2410	0.2421	0.2424	0.2438	0.2458	0.2468	0.2471	0.2472	0.2459	0.2442	0.2423	0.2391	0.2355	0.2311	0.2270	0.2217	0.2151	0.2088	0.2048	0.2032	0.2039	0.2094	0.2184	0.2277	0.2359	0.2466	0.2574	0.2681	0.2788	0.2897	0.3007
Angola	0.2266	0.2286	0.2313	0.2345	0.2381	0.2415	0.2432	0.2455	0.2481	0.2513	0.2553	0.2593	0.2639	0.2671	0.2706	0.2746	0.2776	0.2811	0.2844	0.2891	0.2935	0.2972	0.3028	0.3088	0.3154	0.3234	0.3320	0.3421	0.3531	0.3629	0.3732	0.3827	0.3921	0.4015	0.4105	0.4191
Central African Republic	0.1917	0.1946	0.1973	0.1992	0.2020	0.2053	0.2078	0.2096	0.2118	0.2148	0.2177	0.2208	0.2231	0.2251	0.2280	0.2311	0.2321	0.2341	0.2368	0.2402	0.2437	0.2467	0.2502	0.2526	0.2557	0.2590	0.2629	0.2673	0.2726	0.2774	0.2824	0.2876	0.2931	0.2895	0.2853	0.2817
Congo	0.3246	0.3329	0.3418	0.3512	0.3609	0.3699	0.3768	0.3835	0.3903	0.3976	0.4042	0.4103	0.4159	0.4209	0.4245	0.4283	0.4324	0.4361	0.4396	0.4428	0.4473	0.4506	0.4543	0.4579	0.4623	0.4679	0.4745	0.4801	0.4861	0.4918	0.4983	0.5035	0.5087	0.5142	0.5204	0.5265
Democratic Republic of the Congo	0.2261	0.2258	0.2254	0.2255	0.2276	0.2291	0.2294	0.2284	0.2290	0.2301	0.2295	0.2280	0.2262	0.2226	0.2184	0.2136	0.2073	0.1997	0.1912	0.1826	0.1724	0.1604	0.1486	0.1401	0.1348	0.1324	0.1369	0.1462	0.1556	0.1639	0.1756	0.1879	0.2000	0.2125	0.2256	0.2388
Equatorial Guinea	0.2270	0.2422	0.2539	0.2637	0.2703	0.2738	0.2710	0.2675	0.2649	0.2631	0.2611	0.2588	0.2626	0.2683	0.2752	0.2860	0.3075	0.3487	0.3758	0.3990	0.4234	0.4488	0.4705	0.4879	0.5062	0.5222	0.5344	0.5458	0.5572	0.5663	0.5743	0.5818	0.5895	0.5965	0.6030	0.6086
Gabon	0.4257	0.4290	0.4325	0.4366	0.4410	0.4457	0.4501	0.4543	0.4590	0.4650	0.4715	0.4785	0.4854	0.4925	0.5001	0.5080	0.5162	0.5245	0.5323	0.5392	0.5461	0.5525	0.5587	0.5648	0.5709	0.5776	0.5835	0.5895	0.5957	0.6013	0.6079	0.6144	0.6213	0.6285	0.6359	0.6435
Eastern Sub-Saharan Africa	0.1682	0.1710	0.1738	0.1768	0.1799	0.1829	0.1859	0.1899	0.1947	0.1994	0.2041	0.2073	0.2098	0.2126	0.2151	0.2181	0.2212	0.2258	0.2301	0.2346	0.2392	0.2441	0.2490	0.2537	0.2594	0.2660	0.2732	0.2814	0.2901	0.2988	0.3078	0.3169	0.3257	0.3345	0.3431	0.3515
Burundi	0.1371	0.1399	0.1420	0.1440	0.1458	0.1485	0.1508	0.1536	0.1568	0.1607	0.1649	0.1675	0.1705	0.1730	0.1752	0.1762	0.1753	0.1748	0.1739	0.1732	0.1728	0.1753	0.1782	0.1819	0.1860	0.1897	0.1933	0.1970	0.2028	0.2077	0.2129	0.2181	0.2235	0.2295	0.2357	0.2395
Comoros	0.2041	0.2071	0.2104	0.2143	0.2187	0.2237	0.2290	0.2347	0.2408	0.2465	0.2522	0.2574	0.2630	0.2689	0.2734	0.2782	0.2825	0.2870	0.2914	0.2959	0.3007	0.3056	0.3109	0.3159	0.3207	0.3254	0.3300	0.3342	0.3381	0.3419	0.3455	0.3491	0.3529	0.3570	0.3611	0.3650
Djibouti	0.2970	0.2976	0.2963	0.2942	0.2934	0.2945	0.3001	0.3064	0.3125	0.3178	0.3228	0.3252	0.3287	0.3332	0.3378	0.3435	0.3494	0.3553	0.3608	0.3661	0.3710	0.3754	0.3796	0.3842	0.3896	0.3951	0.4014	0.4085	0.4164	0.4234	0.4301	0.4366	0.4430	0.4491	0.4553	0.4615
Eritrea	0.1461	0.1486	0.1508	0.1532	0.1561	0.1596	0.1631	0.1675	0.1724	0.1775	0.1825	0.1865	0.1920	0.1995	0.2093	0.2202	0.2342	0.2478	0.2600	0.2703	0.2786	0.2860	0.2928	0.2983	0.3025	0.3059	0.3071	0.3082	0.3076	0.3077	0.3084	0.3106	0.3139	0.3173	0.3207	0.3238
Ethiopia	0.1078	0.1093	0.1114	0.1148	0.1178	0.1197	0.1217	0.1236	0.1263	0.1286	0.1309	0.1314	0.1304	0.1305	0.1304	0.1313	0.1334	0.1366	0.1386	0.1419	0.1467	0.1526	0.1581	0.1623	0.1686	0.1774	0.1876	0.1994	0.2120	0.2249	0.2380	0.2513	0.2642	0.2771	0.2899	0.3022
Kenya	0.2295	0.2395	0.2499	0.2599	0.2698	0.2801	0.2908	0.3021	0.3140	0.3261	0.3378	0.3488	0.3586	0.3673	0.3753	0.3820	0.3866	0.3901	0.3932	0.3962	0.3989	0.4011	0.4032	0.4056	0.4084	0.4120	0.4163	0.4215	0.4263	0.4312	0.4375	0.4443	0.4510	0.4578	0.4646	0.4716
Baringo	0.1624	0.1673	0.1722	0.1769	0.1816	0.1866	0.1920	0.1978	0.2041	0.2106	0.2178	0.2245	0.2301	0.2346	0.2388	0.2421	0.2441	0.2453	0.2463	0.2472	0.2473	0.2467	0.2459	0.2455	0.2459	0.2472	0.2495	0.2529	0.2561	0.2596	0.2640	0.2688	0.2738	0.2789	0.2842	0.2901
Bomet	0.1949	0.2070	0.2186	0.2300	0.2412	0.2529	0.2647	0.2773	0.2901	0.3024	0.3150	0.3265	0.3369	0.3462	0.3544	0.3610	0.3650	0.3680	0.3712	0.3749	0.3787	0.3826	0.3871	0.3924	0.3980	0.4041	0.4107	0.4174	0.4227	0.4282	0.4347	0.4417	0.4487	0.4560	0.4634	0.4708
Bungoma	0.1602	0.1724	0.1843	0.1959	0.2074	0.2195	0.2317	0.2448	0.2588	0.2733	0.2879	0.3011	0.3130	0.3234	0.3328	0.3406	0.3458	0.3501	0.3544	0.3584	0.3624	0.3662	0.3698	0.3733	0.3771	0.3818	0.3873	0.3935	0.3991	0.4046	0.4106	0.4171	0.4237	0.4305	0.4373	0.4444
Busia	0.1866	0.1957	0.2047	0.2135	0.2223	0.2314	0.2407	0.2506	0.2610	0.2714	0.2823	0.2924	0.3012	0.3085	0.3149	0.3200	0.3232	0.3251	0.3263	0.3273	0.3284	0.3285	0.3288	0.3298	0.3315	0.3340	0.3377	0.3432	0.3486	0.3541	0.3601	0.3665	0.3729	0.3796	0.3863	0.3933
Elgeyo-Marakwet	0.2278	0.2352	0.2426	0.2500	0.2575	0.2656	0.2742	0.2835	0.2936	0.3043	0.3156	0.3261	0.3354	0.3433	0.3505	0.3565	0.3603	0.3634	0.3663	0.3686	0.3702	0.3711	0.3721	0.3738	0.3762	0.3798	0.3847	0.3911	0.3970	0.4026	0.4086	0.4151	0.4217	0.4286	0.4354	0.4427
Embu	0.2885	0.2964	0.3042	0.3118	0.3195	0.3274	0.3359	0.3447	0.3542	0.3638	0.3739	0.3834	0.3923	0.4010	0.4097	0.4173	0.4230	0.4276	0.4317	0.4358	0.4398	0.4437	0.4471	0.4499	0.4529	0.4568	0.4617	0.4676	0.4733	0.4788	0.4852	0.4919	0.4987	0.5056	0.5125	0.5194
Garissa	0.2424	0.2473	0.2522	0.2570	0.2618	0.2668	0.2721	0.2777	0.2837	0.2896	0.2969	0.3038	0.3100	0.3155	0.3205	0.3245	0.3271	0.3289	0.3309	0.3327	0.3336	0.3336	0.3337	0.3343	0.3355	0.3376	0.3399	0.3431	0.3463	0.3495	0.3529	0.3565	0.3602	0.3641	0.3680	0.3731
HomaBay	0.1562	0.1677	0.1788	0.1895	0.2002	0.2112	0.2224	0.2344	0.2466	0.2583	0.2704	0.2817	0.2918	0.3008	0.3088	0.3153	0.3194	0.3224	0.3259	0.3299	0.3333	0.3352	0.3367	0.3377	0.3391	0.3419	0.3462	0.3527	0.3597	0.3674	0.3756	0.3838	0.3910	0.3980	0.4051	0.4125
Isiolo	0.1255	0.1289	0.1321	0.1349	0.1376	0.1403	0.1435	0.1467	0.1503	0.1538	0.1580	0.1618	0.1646	0.1668	0.1691	0.1707	0.1716	0.1718	0.1719	0.1720	0.1715	0.1705	0.1694	0.1686	0.1685	0.1696	0.1721	0.1760	0.1791	0.1820	0.1859	0.1902	0.1946	0.1991	0.2036	0.2087
Kajiado	0.2513	0.2616	0.2718	0.2819	0.2920	0.3024	0.3131	0.3243	0.3359	0.3474	0.3589	0.3698	0.3795	0.3879	0.3954	0.4017	0.4059	0.4088	0.4110	0.4132	0.4159	0.4187	0.4218	0.4256	0.4300	0.4353	0.4417	0.4494	0.4565	0.4628	0.4693	0.4763	0.4835	0.4908	0.4980	0.5052
Kakamega	0.1987	0.2073	0.2159	0.2245	0.2332	0.2424	0.2520	0.2624	0.2736	0.2853	0.2975	0.3088	0.3188	0.3277	0.3358	0.3427	0.3473	0.3503	0.3522	0.3541	0.3556	0.3563	0.3569	0.3582	0.3604	0.3637	0.3680	0.3741	0.3804	0.3862	0.3920	0.3985	0.4050	0.4116	0.4184	0.4254
Kericho	0.1863	0.1976	0.2087	0.2195	0.2304	0.2419	0.2536	0.2661	0.2795	0.2933	0.3077	0.3208	0.3326	0.3436	0.3537	0.3623	0.3683	0.3731	0.3777	0.3820	0.3850	0.3865	0.3880	0.3902	0.3931	0.3970	0.4015	0.4074	0.4138	0.4200	0.4263	0.4330	0.4399	0.4469	0.4541	0.4613
Kiambu	0.3041	0.3158	0.3275	0.3389	0.3504	0.3621	0.3741	0.3865	0.3994	0.4123	0.4252	0.4374	0.4485	0.4585	0.4679	0.4760	0.4821	0.4871	0.4915	0.4955	0.4991	0.5022	0.5054	0.5093	0.5137	0.5189	0.5246	0.5306	0.5364	0.5432	0.5508	0.5588	0.5668	0.5749	0.5831	0.5908
Kilifi	0.2235	0.2301	0.2367	0.2433	0.2499	0.2569	0.2644	0.2724	0.2808	0.2893	0.2986	0.3073	0.3153	0.3225	0.3292	0.3346	0.3380	0.3407	0.3435	0.3457	0.3473	0.3483	0.3497	0.3517	0.3544	0.3578	0.3617	0.3662	0.3704	0.3747	0.3792	0.3842	0.3891	0.3941	0.3991	0.4049
Kirinyaga	0.2748	0.2838	0.2928	0.3017	0.3107	0.3199	0.3295	0.3394	0.3499	0.3606	0.3712	0.3812	0.3906	0.3997	0.4087	0.4166	0.4227	0.4276	0.4317	0.4353	0.4387	0.4422	0.4459	0.4501	0.4549	0.4603	0.4661	0.4722	0.4776	0.4831	0.4894	0.4962	0.5029	0.5098	0.5167	0.5235
Kisii	0.1756	0.1934	0.2099	0.2253	0.2400	0.2545	0.2688	0.2833	0.2978	0.3117	0.3258	0.3389	0.3507	0.3611	0.3703	0.3779	0.3832	0.3872	0.3905	0.3945	0.3985	0.4018	0.4050	0.4082	0.4117	0.4162	0.4217	0.4284	0.4349	0.4419	0.4497	0.4579	0.4659	0.4741	0.4822	0.4898
Kisumu	0.2338	0.2460	0.2580	0.2698	0.2815	0.2936	0.3059	0.3188	0.3323	0.3459	0.3603	0.3737	0.3853	0.3945	0.4021	0.4081	0.4118	0.4145	0.4170	0.4198	0.4229	0.4250	0.4265	0.4275	0.4287	0.4312	0.4352	0.4419	0.4502	0.4590	0.4676	0.4764	0.4848	0.4931	0.5015	0.5095
Kitui	0.1946	0.2011	0.2077	0.2142	0.2207	0.2277	0.2351	0.2431	0.2517	0.2600	0.2686	0.2765	0.2839	0.2913	0.2986	0.3048	0.3090	0.3123	0.3155	0.3183	0.3206	0.3227	0.3252	0.3287	0.3330	0.3380	0.3435	0.3495	0.3547	0.3593	0.3643	0.3699	0.3756	0.3815	0.3874	0.3938
Kwale	0.2364	0.2433	0.2503	0.2570	0.2638	0.2709	0.2784	0.2862	0.2945	0.3028	0.3119	0.3204	0.3275	0.3330	0.3374	0.3407	0.3424	0.3434	0.3445	0.3461	0.3478	0.3488	0.3498	0.3512	0.3534	0.3565	0.3604	0.3653	0.3700	0.3750	0.3801	0.3858	0.3915	0.3974	0.4034	0.4099
Laikipia	0.3290	0.3371	0.3453	0.3535	0.3617	0.3704	0.3795	0.3892	0.3994	0.4094	0.4202	0.4303	0.4402	0.4504	0.4605	0.4696	0.4764	0.4814	0.4848	0.4877	0.4905	0.4928	0.4954	0.4988	0.5029	0.5079	0.5134	0.5203	0.5273	0.5337	0.5400	0.5468	0.5537	0.5607	0.5678	0.5750
Lamu	0.2833	0.2917	0.3001	0.3084	0.3168	0.3254	0.3343	0.3436	0.3530	0.3616	0.3697	0.3775	0.3845	0.3909	0.3968	0.4018	0.4051	0.4078	0.4108	0.4139	0.4173	0.4202	0.4234	0.4271	0.4312	0.4360	0.4407	0.4455	0.4501	0.4553	0.4609	0.4670	0.4732	0.4795	0.4858	0.4924
Machakos	0.2535	0.2622	0.2711	0.2798	0.2887	0.2980	0.3077	0.3179	0.3290	0.3407	0.3531	0.3647	0.3753	0.3848	0.3937	0.4013	0.4068	0.4109	0.4145	0.4181	0.4213	0.4242	0.4278	0.4326	0.4385	0.4451	0.4524	0.4607	0.4682	0.4747	0.4813	0.4884	0.4956	0.5029	0.5102	0.5175
Makueni	0.2029	0.2124	0.2219	0.2312	0.2404	0.2500	0.2600	0.2704	0.2811	0.2915	0.3022	0.3122	0.3213	0.3295	0.3370	0.3432	0.3475	0.3510	0.3542	0.3565	0.3569	0.3561	0.3569	0.3601	0.3648	0.3702	0.3762	0.3829	0.3891	0.3952	0.4018	0.4090	0.4162	0.4236	0.4310	0.4384

Appendix Table 5. Socio-Demographic Index values for all estimated GBD 2015 locations, 1980–2015

Location	1980	1981	1982	1983	1984	1985	1986	1987	1988	1989	1990	1991
Mandera	0.1477	0.1511	0.1543	0.1572	0.1600	0.1628	0.1660	0.1694	0.1732	0.1773	0.1817	0.1857
Marsabit	0.1771	0.1805	0.1839	0.1870	0.1901	0.1932	0.1967	0.2003	0.2041	0.2076	0.2123	0.2166
Meru	0.2487	0.2574	0.2661	0.2748	0.2834	0.2922	0.3015	0.3111	0.3213	0.3320	0.3440	0.3554
Migori	0.0898	0.1091	0.1407	0.1646	0.1852	0.2045	0.2226	0.2407	0.2583	0.2748	0.2912	0.3062
Mombasa	0.3803	0.3875	0.3947	0.4017	0.4086	0.4158	0.4234	0.4314	0.4400	0.4490	0.4589	0.4682
Murangâ€™a	0.2624	0.2703	0.2783	0.2862	0.2942	0.3026	0.3115	0.3209	0.3312	0.3421	0.3531	0.3634
Nairobi	0.3892	0.4039	0.4185	0.4329	0.4472	0.4615	0.4759	0.4905	0.5050	0.5193	0.5332	0.5463
Nakuru	0.2941	0.3035	0.3129	0.3224	0.3319	0.3418	0.3521	0.3629	0.3741	0.3851	0.3964	0.4072
Nandi	0.1993	0.2076	0.2159	0.2242	0.2326	0.2415	0.2509	0.2609	0.2716	0.2825	0.2938	0.3042
Narok	0.1503	0.1587	0.1671	0.1753	0.1834	0.1919	0.2007	0.2100	0.2197	0.2291	0.2384	0.2470
Nyamira	0.2612	0.2693	0.2773	0.2852	0.2930	0.3012	0.3099	0.3190	0.3284	0.3377	0.3476	0.3568
Nyandarua	0.2608	0.2695	0.2782	0.2868	0.2955	0.3046	0.3141	0.3241	0.3350	0.3465	0.3578	0.3682
Nyeri	0.2890	0.3000	0.3110	0.3219	0.3327	0.3437	0.3551	0.3668	0.3794	0.3930	0.4065	0.4191
Samburu	0.1781	0.1842	0.1902	0.1961	0.2020	0.2081	0.2146	0.2213	0.2281	0.2341	0.2400	0.2455
Siaya	0.2090	0.2156	0.2223	0.2288	0.2353	0.2424	0.2499	0.2580	0.2669	0.2761	0.2861	0.2954
TaitaTaveta	0.2894	0.2979	0.3064	0.3148	0.3232	0.3320	0.3413	0.3510	0.3615	0.3722	0.3834	0.3939
TanaRiver	0.1967	0.2025	0.2085	0.2143	0.2202	0.2265	0.2332	0.2403	0.2478	0.2551	0.2631	0.2705
TharakaNithi	0.3002	0.3063	0.3123	0.3182	0.3242	0.3304	0.3371	0.3442	0.3518	0.3592	0.3676	0.3756
TransNzoia	0.2135	0.2225	0.2314	0.2401	0.2490	0.2584	0.2681	0.2786	0.2900	0.3022	0.3149	0.3266
Turkana	0.0625	0.0644	0.0663	0.0678	0.0691	0.0703	0.0718	0.0731	0.0746	0.0759	0.0795	0.0827
UasinGishu	0.2419	0.2510	0.2603	0.2695	0.2787	0.2884	0.2984	0.3089	0.3200	0.3311	0.3424	0.3530
Vihiga	0.2198	0.2281	0.2363	0.2444	0.2525	0.2610	0.2698	0.2792	0.2889	0.2983	0.3079	0.3171
Wajir	0.1230	0.1256	0.1280	0.1302	0.1322	0.1343	0.1368	0.1393	0.1423	0.1456	0.1504	0.1549
WestPokot	0.1638	0.1694	0.1750	0.1806	0.1861	0.1920	0.1981	0.2047	0.2118	0.2191	0.2269	0.2342
Madagascar	0.2353	0.2402	0.2443	0.2478	0.2505	0.2524	0.2541	0.2552	0.2565	0.2585	0.2614	0.2632
Malawi	0.1568	0.1564	0.1554	0.1547	0.1557	0.1594	0.1673	0.1759	0.1835	0.1895	0.1938	0.1942
Mozambique	0.0886	0.0901	0.0901	0.0860	0.0809	0.0729	0.0617	0.0604	0.0608	0.0614	0.0622	0.0645
Rwanda	0.1339	0.1359	0.1368	0.1388	0.1425	0.1489	0.1600	0.1731	0.1858	0.1964	0.2050	0.2083
Somalia	0.1098	0.1101	0.1094	0.1070	0.1052	0.1050	0.1056	0.1080	0.1108	0.1137	0.1158	0.1157
South Sudan	0.1117	0.1119	0.1123	0.1127	0.1149	0.1202	0.1249	0.1297	0.1345	0.1392	0.1435	0.1461
Tanzania	0.2463	0.2487	0.2513	0.2541	0.2572	0.2609	0.2629	0.2646	0.2678	0.2713	0.2757	0.2798
Uganda	0.1963	0.1946	0.1945	0.1954	0.1960	0.1962	0.1959	0.1965	0.1987	0.2016	0.2049	0.2071
Zambia	0.2811	0.2850	0.2884	0.2942	0.2998	0.3053	0.3092	0.3137	0.3177	0.3213	0.3257	0.3283
Southern Sub-Saharan Africa	0.4784	0.4852	0.4918	0.4981	0.5045	0.5106	0.5163	0.5220	0.5281	0.5343	0.5401	0.5456
Botswana	0.2931	0.3028	0.3131	0.3246	0.3365	0.3492	0.3626	0.3766	0.3910	0.4058	0.4202	0.4345
Lesotho	0.2808	0.2858	0.2906	0.2949	0.2997	0.3047	0.3087	0.3125	0.3175	0.3232	0.3289	0.3363
Namibia	0.3574	0.3640	0.3701	0.3759	0.3820	0.3887	0.3997	0.4110	0.4219	0.4320	0.4408	0.4478
South Africa	0.5446	0.5511	0.5569	0.5622	0.5675	0.5724	0.5767	0.5810	0.5855	0.5898	0.5937	0.5971
Eastern Cape	0.5165	0.5232	0.5290	0.5340	0.5392	0.5436	0.5473	0.5511	0.5550	0.5589	0.5620	0.5645
Free State	0.5479	0.5538	0.5593	0.5643	0.5693	0.5738	0.5779	0.5821	0.5862	0.5903	0.5940	0.5973
Gauteng	0.6152	0.6207	0.6260	0.6309	0.6358	0.6403	0.6446	0.6488	0.6530	0.6571	0.6609	0.6644
KwaZulu-Natal	0.5319	0.5382	0.5438	0.5489	0.5541	0.5588	0.5630	0.5672	0.5715	0.5757	0.5794	0.5826
Limpopo	0.4966	0.5030	0.5085	0.5133	0.5182	0.5223	0.5258	0.5293	0.5329	0.5364	0.5393	0.5415
Mpumalanga	0.5533	0.5591	0.5644	0.5692	0.5740	0.5783	0.5822	0.5861	0.5901	0.5940	0.5974	0.6004
North-West	0.5397	0.5457	0.5511	0.5560	0.5610	0.5654	0.5694	0.5733	0.5774	0.5812	0.5847	0.5877
Northern Cape	0.5448	0.5505	0.5557	0.5605	0.5653	0.5697	0.5737	0.5776	0.5816	0.5855	0.5890	0.5921
Western Cape	0.6019	0.6078	0.6133	0.6185	0.6236	0.6284	0.6329	0.6373	0.6417	0.6461	0.6501	0.6538
Swaziland	0.3211	0.3274	0.3328	0.3381	0.3441	0.3512	0.3615	0.3743	0.3878	0.4017	0.4159	0.4262
Zimbabwe	0.3064	0.3168	0.3283	0.3412	0.3533	0.3657	0.3773	0.3884	0.4007	0.4129	0.4245	0.4350
Western Sub-Saharan Africa	0.2265	0.2296	0.2327	0.2356	0.2386	0.2423	0.2450	0.2476	0.2507	0.2542	0.2581	0.2618
Benin	0.1635	0.1668	0.1704	0.1740	0.1779	0.1820	0.1856	0.1887	0.1919	0.1948	0.1984	0.2028
Burkina Faso	0.0719	0.0721	0.0723	0.0724	0.0724	0.0732	0.0740	0.0754	0.0804	0.0852	0.0897	0.0943
Cameroon	0.2646	0.2693	0.2744	0.2800	0.2864	0.2933	0.2996	0.3058	0.3116	0.3173	0.3227	0.3273
Cape Verde	0.2256	0.2356	0.2452	0.2554	0.2647	0.2738	0.2810	0.2876	0.2936	0.2998	0.3056	0.3142
Chad	0.1235	0.1238	0.1243	0.1257	0.1272	0.1302	0.1322	0.1343	0.1379	0.1414	0.1447	0.1480
Cote d'Ivoire	0.2075	0.2131	0.2191	0.2249	0.2305	0.2361	0.2408	0.2453	0.2500	0.2552	0.2602	0.2649
The Gambia	0.2015	0.2024	0.2049	0.2083	0.2116	0.2151	0.2196	0.2244	0.2291	0.2340	0.2388	0.2400
Ghana	0.3018	0.3061	0.3101	0.3138	0.3180	0.3225	0.3268	0.3313	0.3367	0.3424	0.3486	0.3555
Guinea	0.1425	0.1454	0.1486	0.1519	0.1549	0.1578	0.1598	0.1616	0.1638	0.1666	0.1700	0.1745
Guinea-Bissau	0.1478	0.1490	0.1505	0.1520	0.1541	0.1563	0.1587	0.1618	0.1656	0.1698	0.1744	0.1794
Liberia	0.1925	0.1949	0.1982	0.2016	0.2045	0.2073	0.2087	0.2106	0.2134	0.2164	0.2152	0.2122
Mali	0.0768	0.0807	0.0846	0.0885	0.0923	0.0965	0.0997	0.1026	0.1052	0.1085	0.1122	0.1170
Mauritania	0.2279	0.2324	0.2364	0.2404	0.2440	0.2476	0.2509	0.2543	0.2579	0.2617	0.2654	0.2693
Niger	0.0804	0.0802	0.0801	0.0800	0.0790	0.0782	0.0769	0.0787	0.0807	0.0826	0.0846	0.0864
Nigeria	0.2793	0.2821	0.2847	0.2870	0.2894	0.2928	0.2947	0.2959	0.2978	0.3002	0.3036	0.3066
Sao Tome and Principe	0.2622	0.2666	0.2720	0.2769	0.2809	0.2849	0.2895	0.2926	0.2952	0.2987	0.3028	0.3067
Senegal	0.1600	0.1633	0.1677	0.1722	0.1770	0.1821	0.1873	0.1928	0.1984	0.2042	0.2099	0.2154
Sierra Leone	0.1459	0.1486	0.1513	0.1535	0.1560	0.1587	0.1617	0.1657	0.1702	0.1748	0.1796	0.1832
Togo	0.2028	0.2067	0.2107	0.2146	0.2187	0.2228	0.2265	0.2298	0.2341	0.2390	0.2447	0.2500

Location	1992	1993	1994	1995	1996	1997	1998	1999	2000	2001	2002	2003
Mandera	0.1888	0.1913	0.1936	0.1951	0.1958	0.1958	0.1956	0.1952	0.1949	0.1941	0.1929	0.1920
Marsabit	0.2202	0.2232	0.2261	0.2285	0.2300	0.2309	0.2317	0.2322	0.2332	0.2342	0.2356	0.2375
Meru	0.3660	0.3757	0.3846	0.3922	0.3977	0.4020	0.4057	0.4090	0.4118	0.4144	0.4171	0.4201
Migori	0.3199	0.3320	0.3426	0.3513	0.3569	0.3615	0.3663	0.3723	0.3792	0.3851	0.3889	0.3902
Mombasa	0.4765	0.4838	0.4906	0.4964	0.5004	0.5033	0.5055	0.5077	0.5102	0.5129	0.5160	0.5195
Murangâ€™a	0.3726	0.3810	0.3889	0.3958	0.4009	0.4050	0.4087	0.4120	0.4150	0.4181	0.4216	0.4260
Nairobi	0.5580	0.5683	0.5776	0.5856	0.5915	0.5963	0.6005	0.6049	0.6102	0.6157	0.6209	0.6259
Nakuru	0.4171	0.4261	0.4345	0.4416	0.4467	0.4508	0.4547	0.4583	0.4612	0.4633	0.4654	0.4679
Nandi	0.3139	0.3231	0.3319	0.3393	0.3446	0.3484	0.3514	0.3542	0.3575	0.3608	0.3638	0.3666
Narok	0.2545	0.2610	0.2669	0.2716	0.2746	0.2766	0.2785	0.2802	0.2817	0.2825	0.2834	0.2848
Nyamira	0.3651	0.3725	0.3795	0.3853	0.3894	0.3914	0.3925	0.3954	0.4005	0.4062	0.4107	0.4129
Nyandarua	0.3774	0.3854	0.3928	0.3991	0.4035	0.4069	0.4101	0.4133	0.4160	0.4183	0.4210	0.4243
Nyeri	0.4303	0.4401	0.4493	0.4573	0.4635	0.4686	0.4730	0.4770	0.4805	0.4839	0.4873	0.4910
Samburu	0.2503	0.2544	0.2584	0.2616	0.2637	0.2652	0.2665	0.2671	0.2672	0.2670	0.2671	0.2679
Siaya	0.3036	0.3110	0.3177	0.3233	0.3270	0.3292	0.3307	0.3320	0.3338	0.3351	0.3367	0.3391
TaitaTaveta	0.4035	0.4123	0.4207	0.4280	0.4334	0.4375	0.4412	0.4448	0.4483	0.4518	0.4559	0.4607
TanaRiver	0.2771	0.2830	0.2883	0.2926	0.2952	0.2971	0.2984	0.2984	0.2970	0.2945	0.2927	0.2926
TharakaNithi	0.3828	0.3893	0.3954	0.4007	0.4044	0.4073	0.4100	0.4121	0.4135	0.4148	0.4168	0.4202
TransNzoia	0.3369	0.3459	0.3540	0.3608	0.3653	0.3685	0.3711	0.3737	0.3760	0.3772	0.3783	0.3799
Turkana	0.0851	0.0869	0.0889	0.0904	0.0915	0.0921	0.0925	0.0924	0.0918	0.0911	0.0903	0.0898
UasinGishu	0.3626	0.3711	0.3788	0.3852	0.3894	0.3931	0.3974	0.4021	0.4074	0.4127	0.4169	0.4197
Vihiga	0.3253	0.3328	0.3398	0.3456	0.3498	0.3531	0.3562	0.3588	0.3607	0.3620	0.3631	0.3646
Wajir	0.1585	0.1615	0.1644	0.1665	0.1680	0.1687	0.1697	0.1707	0.1707	0.1699	0.1689	0.1681
WestPokot	0.2403	0.2450	0.2492	0.2523	0.2542	0.2550	0.2554	0.2557	0.2560	0.2557	0.2555	0.2557
Madagascar	0.2655	0.2688	0.2718	0.2747	0.2779	0.2815	0.2856	0.2905	0.2961	0.3024	0.3065	0.3116
Malawi	0.1921	0.1921	0.1919	0.1951	0.1997	0.2064	0.2121	0.2173	0.2218	0.2233	0.2248	0.2270
Mozambique	0.0669	0.0691	0.0710	0.0727	0.0740	0.0893	0.1065	0.1206	0.1310	0.1424	0.1540	0.1650
Rwanda	0.2110	0.2127	0.2093	0.2112	0.2144	0.2210	0.2273	0.2317	0.2347	0.2391	0.2451	0.2509
Somalia	0.1137	0.1115	0.1083	0.1059	0.1046	0.1040	0.1036	0.1035	0.1034	0.1031	0.1036	0.1054
South Sudan	0.1480	0.1494	0.1513	0.1541	0.1587	0.1648	0.1713	0.1775	0.1830	0.1877	0.1921	0.1966
Tanzania	0.2836	0.2873	0.2907	0.2939	0.2967	0.2997	0.3030	0.3066	0.3106	0.3147	0.3193	0.3244
Uganda	0.2096	0.2129	0.2187	0.2253	0.2311	0.2370	0.2433	0.2500	0.2566	0.2631	0.2699	0.2769
Zambia	0.3311	0.3336	0.3348	0.3368	0.3387	0.3413	0.3440	0.3473	0.3511	0.3544	0.3580	0.3627
Southern Sub-Saharan Africa	0.5503	0.5547	0.5579	0.5611	0.5666	0.5718	0.5763	0.5814	0.5868	0.5922	0.5978	0.6030
Botswana	0.4478	0.4609	0.4733	0.4854	0.4952	0.5051	0.5148	0.5250	0.5346	0.5437	0.5527	0.5612
Lesotho	0.3439	0.3515	0.3589	0.3657	0.3731	0.3796	0.3865	0.3936	0.4014	0.4092	0.4167	0.4246
Namibia	0.4552	0.4621	0.4695	0.4772	0.4852	0.4935	0.5024	0.5109	0.5190	0.5256	0.5322	0.5385
South Africa	0.6001	0.6026	0.6032	0.6040	0.6084	0.6124	0.6156	0.6198	0.6245	0.6298	0.6357	0.6416
Eastern Cape	0.5663	0.5677	0.5674	0.5678	0.5719	0.5756	0.5784	0.5822	0.5869	0.5922	0.5982	0.6044
Free State	0.6003	0.6031	0.6035	0.6023	0.6054	0.6113	0.6160	0.6203	0.6250	0.6299	0.6353	0.6407
Gauteng	0.6675	0.6697	0.6704	0.6711	0.6740	0.6767	0.6798	0.6841	0.6886	0.6934	0.6982	0.7031
KwaZulu-Natal	0.5855	0.5881	0.5889	0.5900	0.5947	0.5987	0.6016	0.6055	0.6100	0.6151	0.6207	0.6264
Limpopo	0.5432	0.5445	0.5435	0.5428	0.5452	0.5463	0.5476	0.5514	0.5557	0.5604	0.5658	0.5713
Mpumalanga	0.6031	0.6051	0.6043	0.6037	0.6074	0.6099	0.6119	0.6158	0.6201	0.6247	0.6297	0.6349
North-West	0.5902	0.5921	0.5921	0.5927	0.5971	0.6011	0.6039	0.6072	0.6111	0.6156	0.6207	0.6259
Northern Cape	0.5948	0.5966	0.5960	0.5960	0.6015	0.6066	0.6089	0.6113	0.6145	0.6188	0.6240	0.6292
Western Cape	0.6569	0.6593	0.6600	0.6614	0.6674	0.6736	0.6769	0.6794	0.6829	0.6876	0.6929	0.6982
Swaziland	0.4364	0.4470	0.4577	0.4693	0.4810	0.4922	0.5028	0.5125	0.5210	0.5275	0.5333	0.5364
Zimbabwe	0.4434	0.4514	0.4602	0.4681	0.4761	0.4838	0.4909	0.4976	0.5031	0.5072	0.5095	0.5101
Western Sub-Saharan Africa	0.2656	0.2696	0.2736	0.2777	0.2817	0.2861	0.2906	0.2953	0.3003	0.3053	0.3106	0.3166
Benin	0.2074	0.2125	0.2172	0.2223	0.2269	0.2319	0.2370	0.2427	0.2488	0.2555	0.2625	0.2696
Burkina Faso	0.0986	0.1030	0.1070	0.1113	0.1160	0.1211	0.1268	0.1328	0.1386	0.1443	0.1501	0.1563
Cameroon	0.3316	0.3355	0.3393	0.3435	0.3478	0.3526	0.3581	0.3639	0.3700	0.3759	0.3820	0.3881
Cape Verde	0.3232	0.3325	0.3427	0.3532	0.3638	0.3746	0.3862	0.3990	0.4120	0.4249	0.4373	0.4494
Chad	0.1507	0.1527	0.1552	0.1576	0.1592	0.1612	0.1638	0.1667	0.1696	0.1729	0.1772	0.1832
Cote d'Ivoire	0.2696	0.2745	0.2793	0.2844	0.2896	0.2951	0.3012	0.3072	0.3126	0.3169	0.3209	0.3245
The Gambia	0.2412	0.2428	0.2446	0.2469	0.2496	0.2530	0.2570	0.2619	0.2672	0.2713	0.2747	0.2787
Ghana	0.3626	0.3696	0.3765	0.3829	0.3887	0.3945	0.3999	0.4056	0.4111	0.4163	0.4214	0.4272
Guinea	0.1794	0.1846	0.1894	0.1941	0.1982	0.2018	0.2050	0.2085	0.2121	0.2158	0.2197	0.2235
Guinea-Bissau	0.1842	0.1895	0.1952	0.2013	0.2074	0.2142	0.2181	0.2224	0.2267	0.2310	0.2347	0.2387
Liberia	0.2056	0.1957	0.1833	0.1682	0.1498	0.1409	0.1404	0.1520	0.1764	0.1944	0.2093	0.2113
Mali	0.1217	0.1268	0.1317	0.1365	0.1409	0.1455	0.1501	0.1547	0.1585	0.1629	0.1673	0.1718
Mauritania	0.2733	0.2778	0.2819	0.2864	0.2914	0.2957	0.2999	0.3046	0.3090	0.3135	0.3180	0.3230
Niger	0.0882	0.0902	0.0924	0.0943	0.0957	0.0971	0.0991	0.1010	0.1026	0.1045	0.1068	0.1094
Nigeria	0.3099	0.3138	0.3179	0.3221	0.3266	0.3315	0.3364	0.3412	0.3467	0.3523	0.3583	0.3659
Sao Tome and Principe	0.3112	0.3162	0.3209	0.3252	0.3301	0.3347	0.3394	0.3443	0.3496	0.3551	0.3609	0.3671
Senegal	0.2209	0.2266	0.2319	0.2372	0.2420	0.2467	0.2519	0.2574	0.2629	0.2685	0.2738	0.2793
Sierra Leone	0.1851	0.1880	0.1917	0.1952	0.1971	0.1982	0.1995	0.1997	0.2018	0.2057	0.2133	0.2217
Togo	0.2550	0.2579	0.2620	0.2673	0.2727	0.2788	0.2835	0.2882	0.2922	0.2957	0.2990	0.3026

Location	2004	2005	2006	2007	2008	2009	2010	2011	2012	2013	2014	2015
Mandera	0.1919	0.1927	0.1940	0.1960	0.1978	0.2002	0.2028	0.2057	0.2087	0.2118	0.2151	0.2195
Marsabit	0.2402	0.2434	0.2471	0.2516	0.2552	0.2585	0.2618	0.2654	0.2691	0.2729	0.2767	0.2814
Meru	0.4236	0.4277	0.4324	0.4373	0.4414	0.4462	0.4521	0.4584	0.4647	0.4713	0.4779	0.4845
Migori	0.3910	0.3933	0.3970	0.4024	0.4081	0.4148	0.4224	0.4307	0.4389	0.4472	0.4555	0.4635
Mombasa	0.5236	0.5282	0.5334	0.5388	0.5434	0.5484	0.5545	0.5611	0.5678	0.5746	0.5814	0.5885
Murangâ€™a	0.4312	0.4372	0.4437	0.4506	0.4564	0.4619	0.4682	0.4749	0.4816	0.4884	0.4952	0.5021
Nairobi	0.6311	0.6370	0.6434	0.6504	0.6574	0.6653	0.6737	0.6827	0.6916	0.7008	0.7099	0.7182
Nakuru	0.4711	0.4751	0.4801	0.4865	0.4930	0.4993	0.5061	0.5134	0.5207	0.5282	0.5357	0.5432
Nandi	0.3695	0.3733	0.3776	0.3825	0.3870	0.3920	0.3976	0.4036	0.4096	0.4159	0.4222	0.4289
Narok	0.2868	0.2897	0.2930	0.2965	0.2996	0.3038	0.3090	0.3146	0.3202	0.3261	0.3321	0.3386
Nyamira	0.4142	0.4164	0.4195	0.4240	0.4292	0.4360	0.4439	0.4516	0.4590	0.4666	0.4742	0.4817
Nyandarua	0.4283	0.4331	0.4382	0.4438	0.4489	0.4540	0.4598	0.4659	0.4721	0.4785	0.4849	0.4915
Nyeri	0.4952	0.5000	0.5058	0.5126	0.5191	0.5255	0.5325	0.5399	0.5473	0.5547	0.5622	0.5694
Samburu	0.2691	0.2709	0.2732	0.2765	0.2798	0.2840	0.2890	0.2945	0.3000	0.3056	0.3113	0.3174
Siaya	0.3424	0.3466	0.3516	0.3575	0.3635	0.3701	0.3772	0.3845	0.3909	0.3971	0.4034	0.4102
TaitaTaveta	0.4662	0.4725	0.4789	0.4852	0.4909	0.4969	0.5036	0.5108	0.5181	0.5255	0.5328	0.5401
TanaRiver	0.2933	0.2945	0.2961	0.2984	0.3009	0.3044	0.3087	0.3135	0.3184	0.3235	0.3287	0.3348
TharakaNithi	0.4248	0.4301	0.4361	0.4431	0.4494	0.4548	0.4606	0.4669	0.4732	0.4797	0.4862	0.4930
TransNzoia	0.3822	0.3853	0.3894	0.3945	0.3997	0.4053	0.4115	0.4181	0.4248	0.4318	0.4387	0.4459
Turkana	0.0898	0.0908	0.0930	0.0969	0.1001	0.1030	0.1063	0.1099	0.1134	0.1171	0.1207	0.1249
UasinGishu	0.4223	0.4256	0.4300	0.4362	0.4424	0.4484	0.4548	0.4616	0.4686	0.4757	0.4828	0.4900
Vihiga	0.3667	0.3698	0.3742	0.3808	0.3874	0.3931	0.3991	0.4056	0.4122	0.4190	0.4259	0.4328
Wajir	0.1679	0.1685	0.1699	0.1726	0.1748	0.1769	0.1796	0.1826	0.1856	0.1887	0.1920	0.1962
WestPokot	0.2563	0.2576	0.2599	0.2641	0.2683	0.2725	0.2772	0.2823	0.2875	0.2929	0.2983	0.3044
Madagascar	0.3170	0.3225	0.3281	0.3338	0.3399	0.3445	0.3489	0.3531	0.3574	0.3615	0.3656	0.3698
Malawi	0.2299	0.2331	0.2374	0.2444	0.2522	0.2610	0.2703	0.2792	0.2871	0.2947	0.3019	0.3086
Mozambique	0.1755	0.1860	0.1960	0.2058	0.2152	0.2244	0.2334	0.2422	0.2510	0.2600	0.2690	0.2780
Rwanda	0.2583	0.2664	0.2762	0.2864	0.2978	0.3092	0.3204	0.3315	0.3426	0.3528	0.3624	0.3713
Somalia	0.1078	0.1108	0.1142	0.1183	0.1226	0.1270	0.1315	0.1356	0.1397	0.1439	0.1475	0.1506
South Sudan	0.2015	0.2066	0.2120	0.2176	0.2234	0.2293	0.2352	0.2405	0.2459	0.2513	0.2565	0.2616
Tanzania	0.3300	0.3364	0.3424	0.3493	0.3567	0.3642	0.3719	0.3798	0.3874	0.3952	0.4032	0.4114
Uganda	0.2841	0.2920	0.3000	0.3084	0.3174	0.3268	0.3361	0.3451	0.3534	0.3614	0.3692	0.3768
Zambia	0.3681	0.3743	0.3814	0.3902	0.3998	0.4096	0.4204	0.4307	0.4407	0.4501	0.4590	0.4670
Southern Sub-Saharan Africa	0.6084	0.6138	0.6192	0.6249	0.6298	0.6346	0.6398	0.6456	0.6516	0.6577	0.6636	0.6688
Botswana	0.5696	0.5774	0.5854	0.5931	0.6006	0.6053	0.6107	0.6162	0.6217	0.6281	0.6346	0.6411
Lesotho	0.4323	0.4399	0.4480	0.4563	0.4643	0.4719	0.4803	0.4883	0.4965	0.5050	0.5135	0.5217
Namibia	0.5456	0.5526	0.5596	0.5664	0.5727	0.5782	0.5837	0.5897	0.5959	0.6026	0.6096	0.6170
South Africa	0.6478	0.6545	0.6614	0.6686	0.6757	0.6819	0.6881	0.6945	0.7007	0.7065	0.7119	0.7164
Eastern Cape	0.6111	0.6184	0.6263	0.6346	0.6426	0.6492	0.6558	0.6627	0.6692	0.6752	0.6806	0.6850
Free State	0.6464	0.6525	0.6589	0.6656	0.6721	0.6778	0.6836	0.6896	0.6952	0.7005	0.7055	0.7096
Gauteng	0.7082	0.7136	0.7192	0.7250	0.7307	0.7359	0.7412	0.7466	0.7519	0.7569	0.7618	0.7659
KwaZulu-Natal	0.6325	0.6390	0.6459	0.6530	0.6600	0.6660	0.6721	0.6784	0.6843	0.6899	0.6950	0.6992
Limpopo	0.5773	0.5838	0.5908	0.5981	0.6050	0.6107	0.6163	0.6225	0.6283	0.6335	0.6382	0.6419
Mpumalanga	0.6403	0.6461	0.6523	0.6587	0.6648	0.6702	0.6756	0.6812	0.6866	0.6916	0.6962	0.7000
North-West	0.6314	0.6373	0.6435	0.6500	0.6563	0.6618	0.6674	0.6732	0.6786	0.6838	0.6885	0.6923
Northern Cape	0.6348	0.6406	0.6467	0.6532	0.6594	0.6650	0.6706	0.6765	0.6819	0.6872	0.6920	0.6961
Western Cape	0.7038	0.7096	0.7157	0.7221	0.7283	0.7340	0.7397	0.7455	0.7512	0.7566	0.7618	0.7663
Swaziland	0.5396	0.5455	0.5523	0.5599	0.5686	0.5772	0.5857	0.5935	0.6014	0.6089	0.6162	0.6232
Zimbabwe	0.5102	0.5091	0.5071	0.5060	0.5021	0.5018	0.5036	0.5076	0.5141	0.5219	0.5302	0.5384
Western Sub-Saharan Africa	0.3239	0.3316	0.3386	0.3459	0.3529	0.3598	0.3668	0.3736	0.3804	0.3872	0.3941	0.4009
Benin	0.2767	0.2835	0.2899	0.2964	0.3028	0.3089	0.3146	0.3201	0.3257	0.3318	0.3381	0.3446
Burkina Faso	0.1625	0.1691	0.1755	0.1821	0.1887	0.1952	0.2023	0.2093	0.2165	0.2236	0.2306	0.2374
Cameroon	0.3944	0.4005	0.4063	0.4123	0.4186	0.4248	0.4311	0.4372	0.4436	0.4501	0.4570	0.4641
Cape Verde	0.4609	0.4715	0.4819	0.4923	0.5019	0.5100	0.5176	0.5251	0.5318	0.5379	0.5434	0.5485
Chad	0.1931	0.2037	0.2133	0.2222	0.2307	0.2388	0.2478	0.2554	0.2634	0.2713	0.2792	0.2871
Cote d'Ivoire	0.3280	0.3317	0.3355	0.3393	0.3432	0.3475	0.3519	0.3558	0.3607	0.3668	0.3735	0.3807
The Gambia	0.2836	0.2878	0.2909	0.2941	0.2979	0.3019	0.3064	0.3095	0.3134	0.3176	0.3218	0.3266
Ghana	0.4332	0.4393	0.4452	0.4512	0.4577	0.4641	0.4710	0.4788	0.4869	0.4951	0.5031	0.5110
Guinea	0.2273	0.2313	0.2353	0.2397	0.2447	0.2493	0.2539	0.2586	0.2636	0.2685	0.2733	0.2777
Guinea-Bissau	0.2427	0.2469	0.2508	0.2552	0.2597	0.2646	0.2695	0.2749	0.2798	0.2845	0.2893	0.2943
Liberia	0.2134	0.2160	0.2200	0.2251	0.2306	0.2359	0.2415	0.2485	0.2570	0.2672	0.2756	0.2827
Mali	0.1763	0.1810	0.1854	0.1901	0.1954	0.2008	0.2064	0.2112	0.2159	0.2205	0.2255	0.2309
Mauritania	0.3280	0.3335	0.3412	0.3483	0.3548	0.3608	0.3673	0.3740	0.3808	0.3877	0.3947	0.4014
Niger	0.1116	0.1141	0.1164	0.1187	0.1215	0.1239	0.1270	0.1297	0.1335	0.1375	0.1420	0.1465
Nigeria	0.3760	0.3867	0.3963	0.4062	0.4156	0.4246	0.4336	0.4421	0.4503	0.4582	0.4661	0.4740
Sao Tome and Principe	0.3735	0.3799	0.3872	0.3940	0.4015	0.4086	0.4153	0.4224	0.4291	0.4352	0.4415	0.4481
Senegal	0.2848	0.2902	0.2950	0.2996	0.3037	0.3076	0.3114	0.3152	0.3193	0.3237	0.3286	0.3341
Sierra Leone	0.2299	0.2375	0.2446	0.2521	0.2597	0.2676	0.2757	0.2836	0.2932	0.3051	0.3167	0.3230
Togo	0.3061	0.3093	0.3132	0.3174	0.3213	0.3257	0.3307	0.3361	0.3421	0.3484	0.3550	0.3617

Appendix Figure 1. Co−evolution of the proportion of DALYs due to YLDs with SDI for the globe and GBD regions from 1990 to 2015, with comparison to the value of expected proportion of DALYs due to YLDs based on SDI alone.

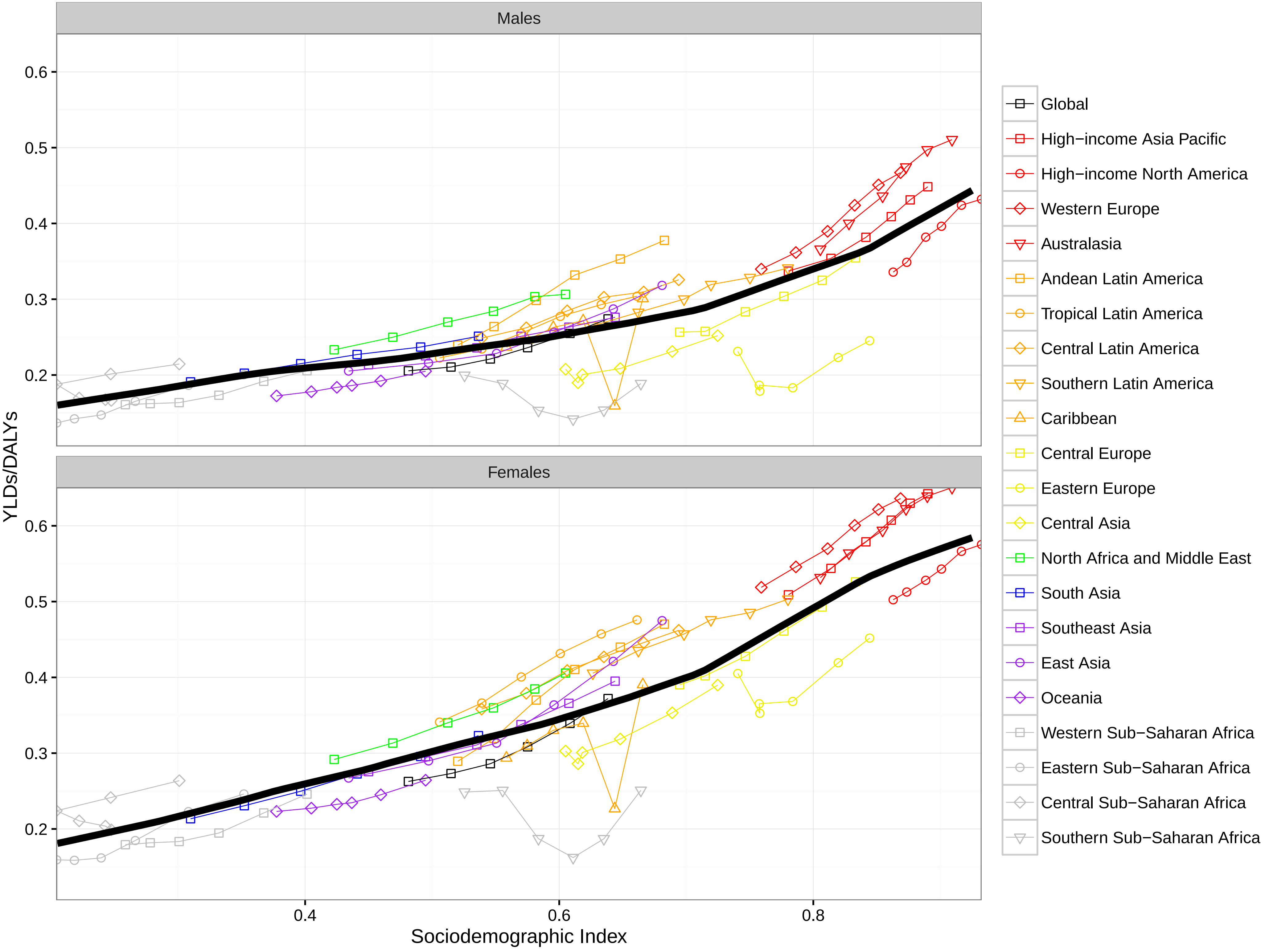

Appendix Figure 2. Years of functional health lost computed as life expectancy at birth minus HALE at birth for both sexes combined, 2015. SDI = Socio−demographic Index. HALE = healthy life expectancy. LE = life expectancy.

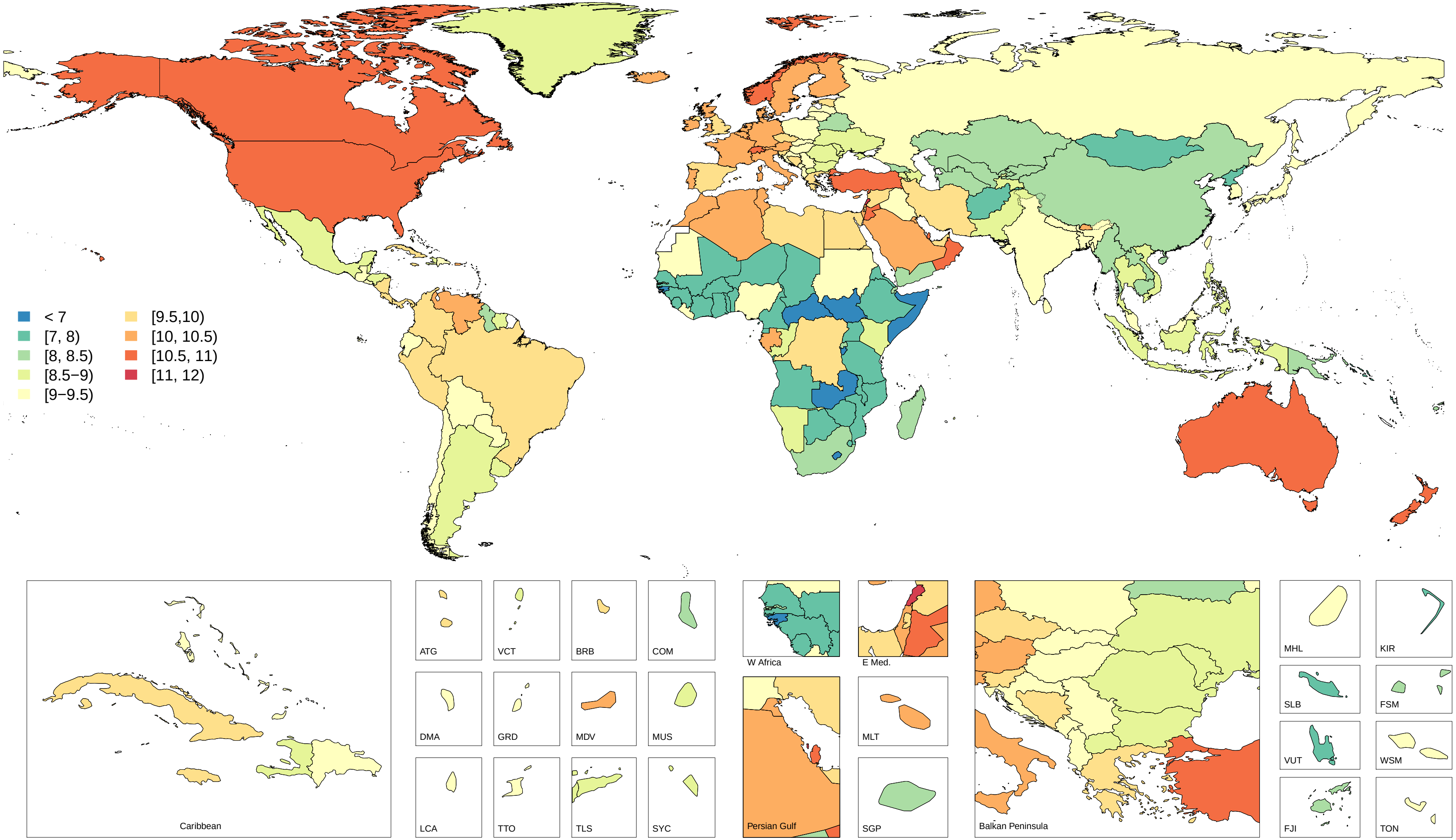

Appendix Figure 3. Observed HALE at birth minus expected HALE at birth for both sexes combined, 2015. SDI = Socio−demographic Index. HALE = healthy life expectancy. LE = life expectancy.

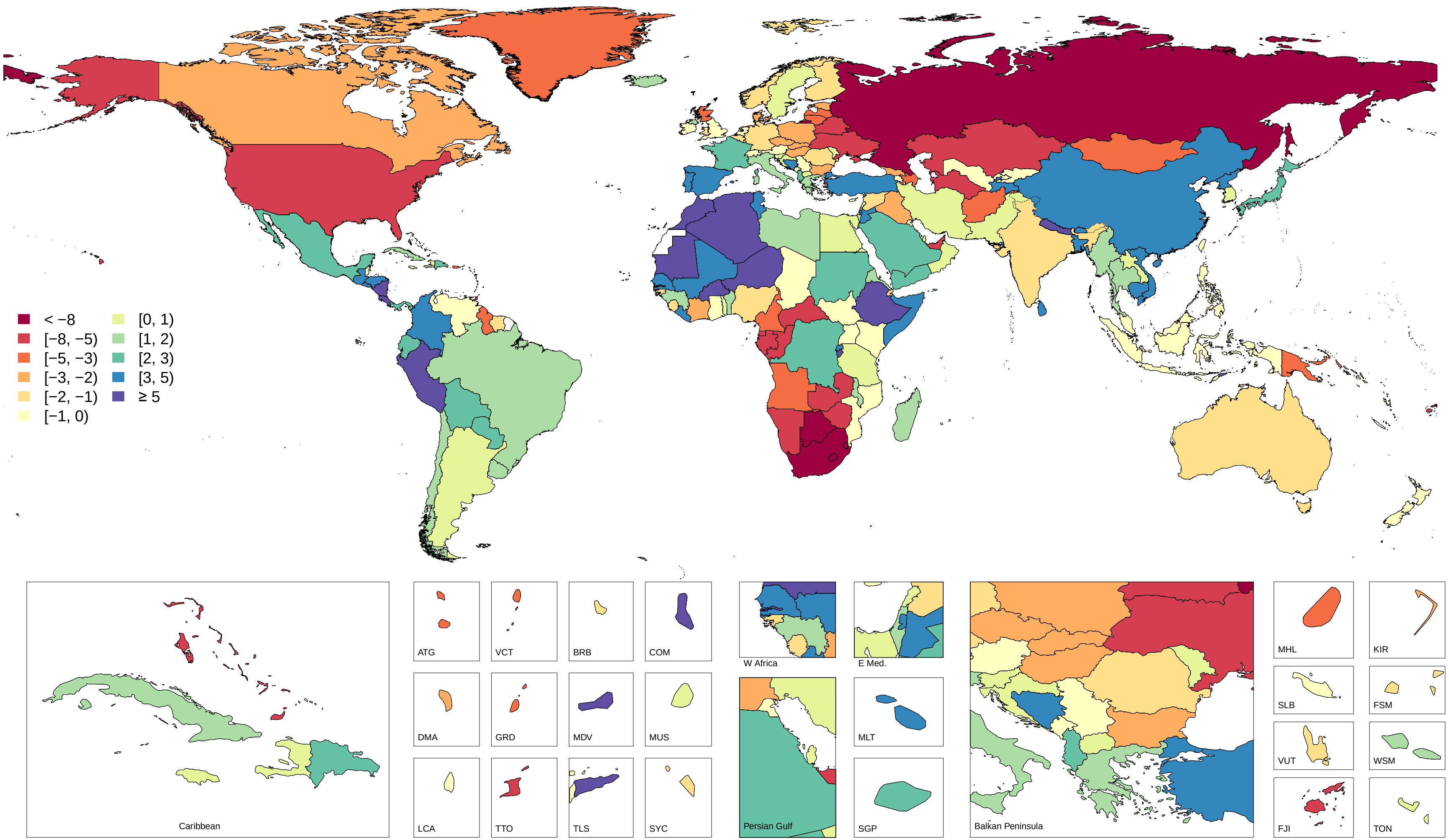

Appendix Figure 4. Ratio of observed versus expected age−standardised DALY rates (per 100,000) on the basis of SDI alone for both sexes combined, 2005. SDI = Socio−demographic Index. DALY = disability−adjusted life year.

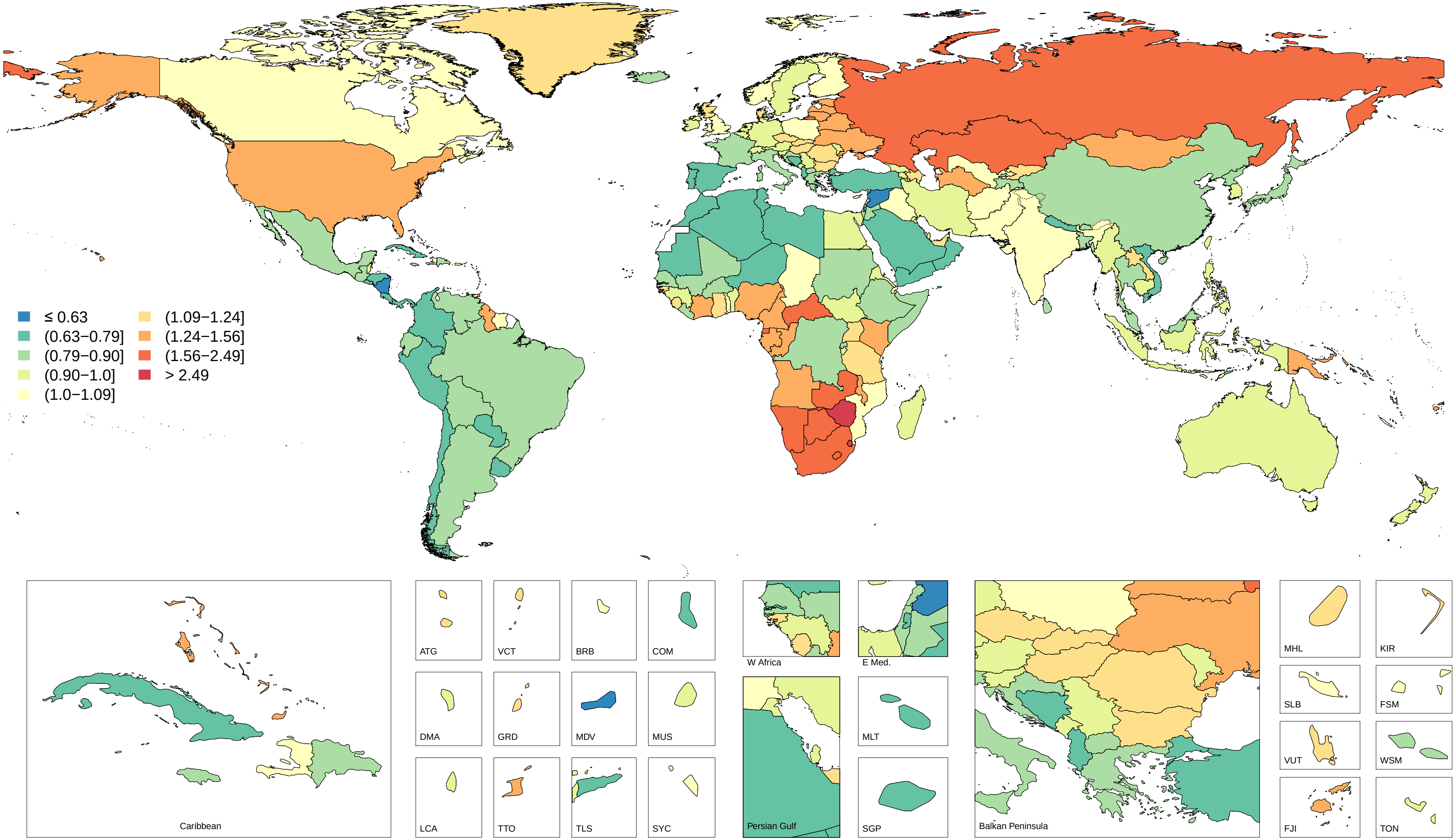

Appendix Figure 5A. Comparisons of GBD 2015 HALE at birth with WHO 2015 HALE at birth.

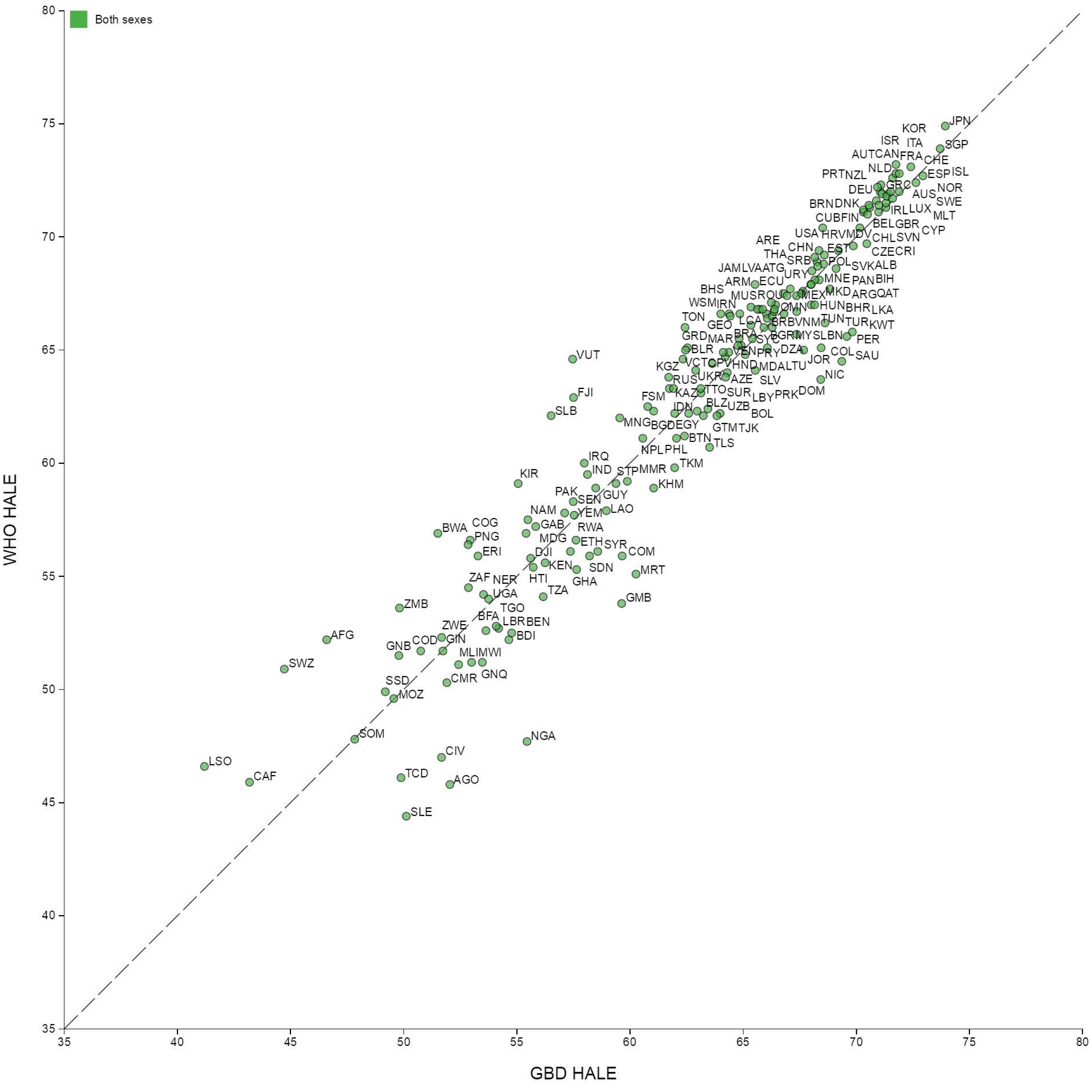

Appendix Figure 5B. Comparisons of GBD 2014 HALE at birth with EC 2014 HALE at birth.

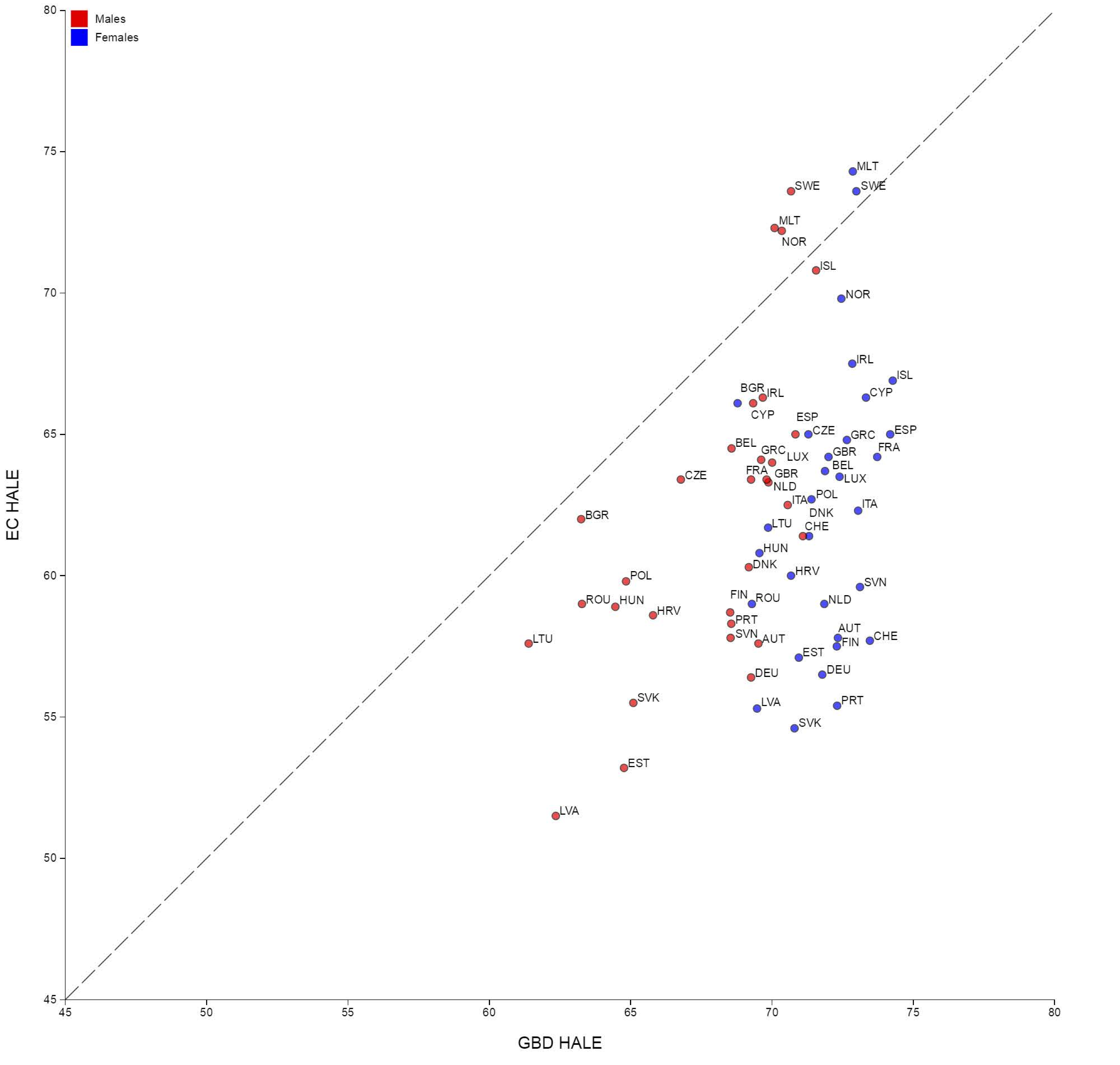

Appendix Figure 5C. Comparisons of GBD 2014 HALE at age 65 with EC 2014 HALE at age 65.

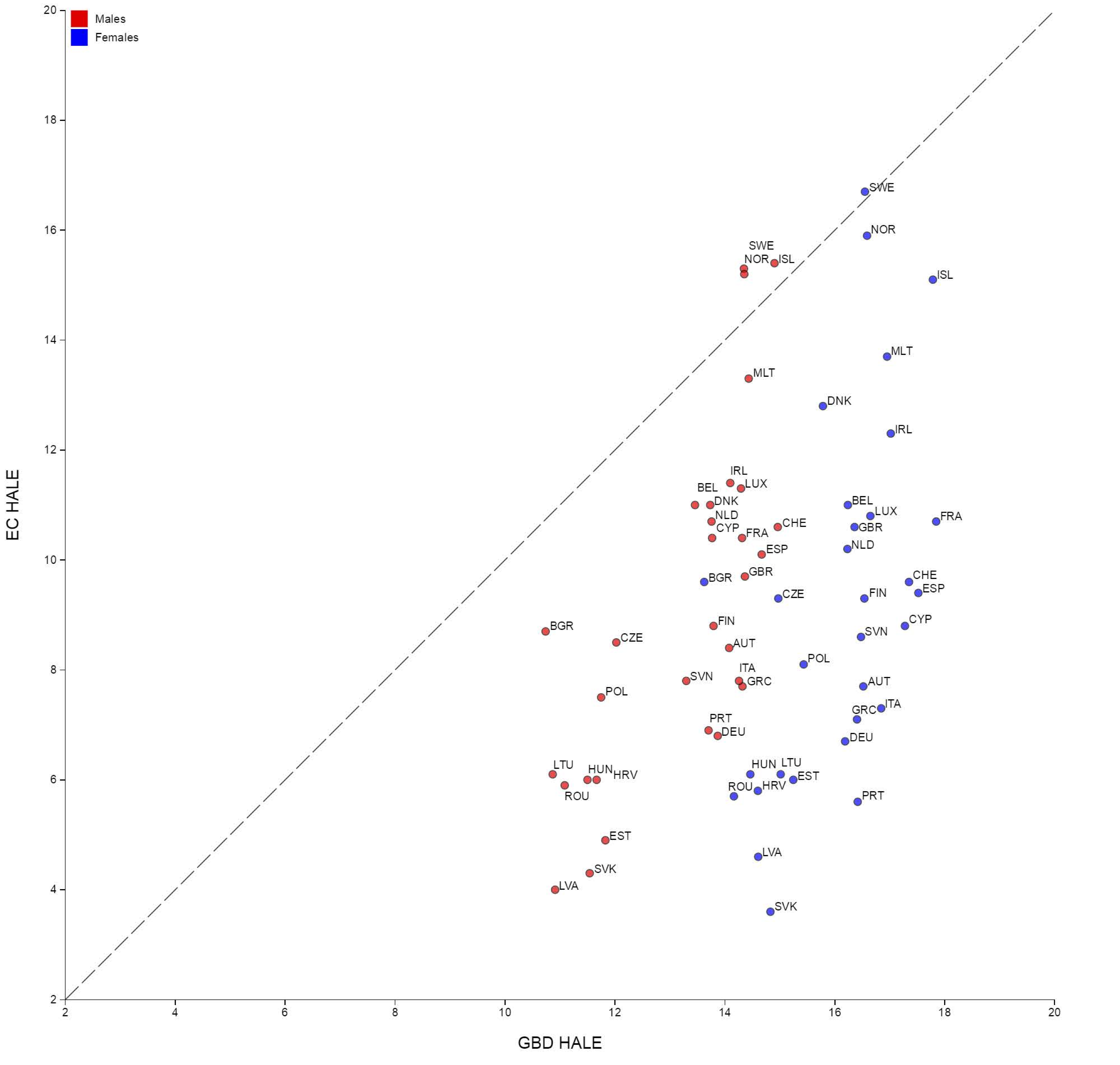

Supplement: Supplementary appendix [file mmc1.pdf]
